# Discovery of VU6083859, a TAOK1 Selective Inhibitor, and VU6080195, a *pan*-TAOK Activator

**DOI:** 10.1021/acschemneuro.5c00906

**Published:** 2026-01-14

**Authors:** Daniel C. Schultz, Lauren C. Parr, Hunter Sweet, Sean Lamb, Julie L. Engers, Nathaniel C. Napier, Hallie G. McKinnie, David Whomble, Valerie Kramlinger, Olivier Boutaud, Craig W. Lindsley

**Affiliations:** 1 Warren Center for Neuroscience Drug Discovery, 5718 Vanderbilt University, Nashville, Tennessee 37232, United States; 2 Department of Pharmacology, 12327 Vanderbilt University School of Medicine, Nashville, Tennessee 37232, United States; 3 Department of Chemistry, 5718 Vanderbilt University, Nashville, Tennessee 37232, United States; 4 Vanderbilt Institute for Therapeutic Advances, Vanderbilt University, Nashville, Tennessee 37232, United States

**Keywords:** medicinal chemistry, neurodegeneration, neurodevelopmental disorder, structure−activity relationship (SAR), thousand and one kinase (TAOK)

## Abstract

The thousand and one (TAO) kinases, TAOK1, TAOK2, and TAOK3, have garnered great interest for their role in, and therapeutic potential for, breast cancer, neurodegeneration in human tauopathies, and a large number of neurodevelopmental disorders (NDDs). However, only one *pan*-TAO kinase inhibitor, referred to as compound 43, has been employed to pharmacologically validate this important family of kinases despite a poor pharmacokinetic­(PK) profile and off-target liabilities. In order to understand the isoenzyme-specific role of TAOKs in NDDs and in regulating tau pathology, isoenzyme-selective inhibitors and activators are required. Here, we report on an iterative medicinal chemistry exercise to expand the chemical space around compound 43, which resulted in the first TAOK1 selective inhibitor VU6083859 (TAOK1 IC_50_ = 158 nM; TAOK2/TAOK1 = 22; TAOK3/TAOK1 > 63; selective versus the Cerep 360 kinase panel) and quite unexpectedly, by virtue of a ‘magic methyl,’ a *pan*-TAOK activator, VU6080195 (TAOK1 EC_50_ = 270 nM, TAOK2 EC_50_ = 1,376 nM, TAOK3 EC_50_ = 503 nM; note: the *des*-methyl congener is a TAOK1-preferring inhibitor). Both new kinase ligands showed modest rat PK, central nervous system (CNS) penetration (*K*_p_s > 0.15) and therefore provide a foundation to further optimize this chemotype to probe and validate the role(s) of TAO kinase modulation in the CNS.

## Graphical abstract

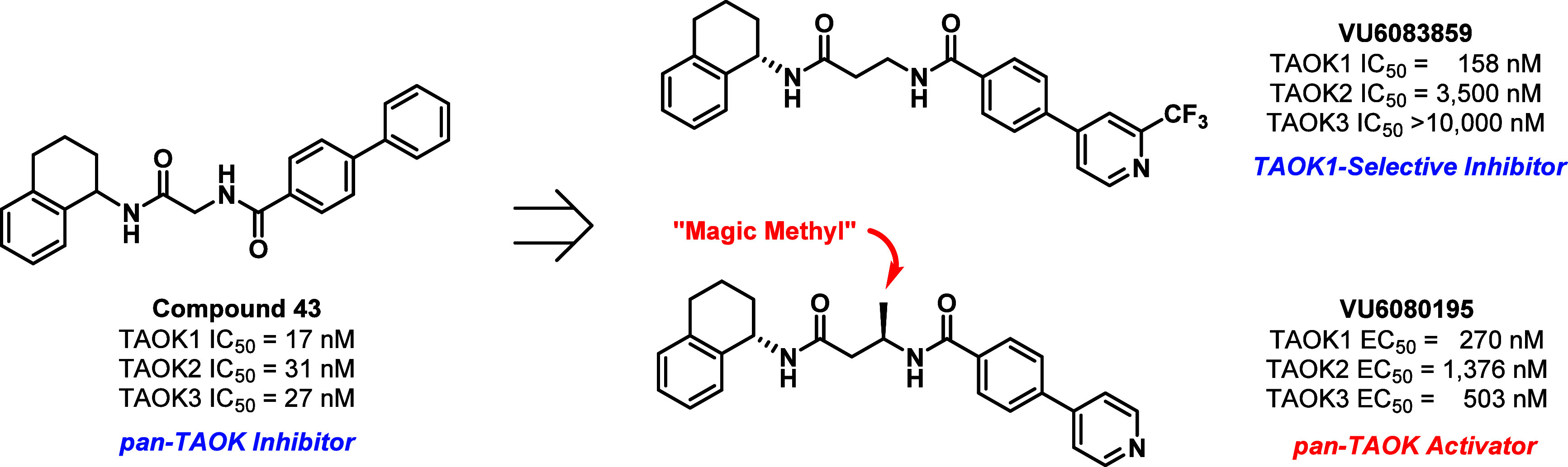

## Introduction

Thousand and one kinases (TAOKs), consisting of three family members (TAOK1, TAOK2, and TAOK3), are serine/threonine kinases within the STE20 kinase family. Each kinase shares a conserved *N*-terminal kinase domain, coiled-coil motifs, and a serine-rich domain, whereas TAOK2 is distinguished by an additional *C*-terminal leucine-rich repeat domain.^1–4^ These structural elements support TAOK involvement in diverse biological processes, including cytoskeletal remodeling, mitotic progression, MAPK signaling, and neuronal development. TAOK1 and TAOK2 are enriched in the central nervous system, where they regulate microtubule stability and neuronal morphology.^1–4^ These kinases are especially important in regulating tau dynamics and are linked to Alzheimer’s disease, frontotemporal lobar dementia (FTLD), and other tauopathies. TAOK3, by contrast, is more broadly expressed and is emerging as a context-dependent modulator of cancer biology, with reported functions in Hippo pathway regulation, chemotherapy resistance, and cell survival.^1–5^ Recently, data has emerged linking genetic variants in TAOKs to several neurodevelopmental disorders (NDDs) including developmental delays, microencephaly, autism spectrum disorder, intellectual disability, attention deficit hyperactivity disorder, epilepsy, and schizophrenia.^5–7^ From the genetic data, each TAOK isoenzyme plays distinct roles in NDDs, and selective inhibitors and activators of each isoenzyme (e.g., TAOK1, TAOK2, and TAOK3) are required to dissect the varying contributions and to afford therapeutic intervention.^4–7^ Recently, TAOK1 was associated with neurodevelopmental disorders and found to be essential for neuronal maturation and cortical development; thus, selective, TAOK1 small-molecule tools are desperately needed.^8^

**Figure 1:**
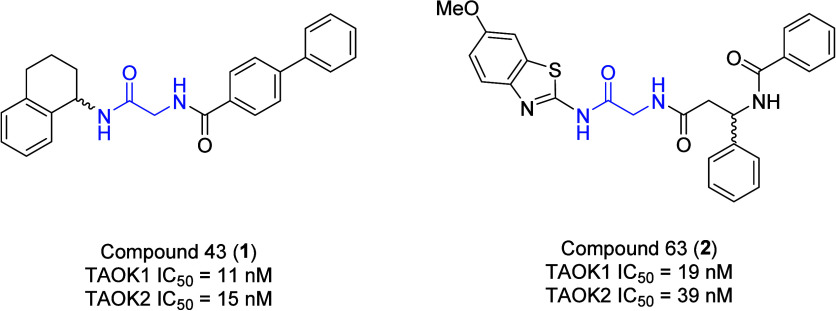
Structures of the first reported *pan*-TAOK inhibitors compound 43 (**1**) and compound 63 (**2**).

A breakthrough in the field occurred in 2017 when Morris and co-workers^9^ published the structures and activities of two *pan*-TAOK inhibitors provided to them by Exelixis, denoted as compound 43 (**1**, TAOK1 IC_50_ = 11 nM, TAOK2 IC_50_ = 15 nM) and compound 63 (**2**, TAOK1 IC_50_ = 19 nM, TAOK2 IC_50_ = 39 nM) (Figure 1) based on an atypical, racemic glycine-centered kinase inhibitor scaffold, which proved to be ATP-competitive and moderately selective versus the kinome. Then, they demonstrated that **1** delays mitosis and induces mitotic cell death in centrosome-amplified breast cancer cells. In the following year, Morris and co-workers published that **1** reduces tau phosphorylation at sites (e.g., T123, T427, S262/S356, and S202/T205/S208) associated with neurodegeneration in human tauopathies, as well as decreasing tau phosphorylation in neurons from FTLD brain tissue and in cortical neurons in the Tau35 transgenic mouse model of tauopathy.^10^ Overall, these data suggest that TAOK inhibition is a novel mechanism by which to reduce and/or prevent tau-associated neurodegeneration. While critical for the TAOK field, deeper characterization of **1** is required, as well as further optimization of **1**, to determine if isoenzyme-selective TAOK inhibitors, or activators,^11–14^ could be developed to further advance the field with respect to neurodegenerative and neurodevelopmental disorders.

## Results and Discussion

We first synthesized **1** (Scheme 1) and profiled it in a battery of biochemical, pharmacological, and *in vitro*/*in vivo* DMPK assays. Racemic **1** and its single enantiomers ((*R*)-**1** and (*S*)-**1**) were prepared in three steps starting from biphenyl-4-carboxylic acid **3**. An HATU-mediated coupling between **3** and glycine methyl ester proceeded smoothly, followed by ester hydrolysis to give **4**. A second HATU-mediated amide coupling between **4** and either racemic, (*R*)- or (*S*)-1,2,3,4-tetrahydronaphthalen-1-amine, delivered racemic **1** (or single enantiomers (*R*)-**1** and (*S*)-**1**). To understand the prior art, we first characterized the racemic **1** employed by Morrison and co-workers. In agreement with the prior art, **1** was a potent *pan*-inhibitor of the three TAOKs (TAOK1 IC_50_ = 17 nM, TAOK2 IC_50_ = 31 nM, and TAOK3 IC_50_ = 27 nM). Across a panel of 378 kinases, **1** exhibited a fairly clean profile, returning <80% kinase activity at 10 μM for only six kinases (Table 1, see the Supporting Information for full results). Furthermore, a lead profiling panel of 68 GPRCS, ion channels, and transporters returned >50% inhibition at 10 μM for six targets (see the Supporting Information for full results), which notably included 100% inhibition of the β_2_ adrenergic receptor and 91% inhibition at the 5-HT_2B_ receptor. As a first-generation tool compound, these activities did not preclude its value or the results obtained. The *pan*-TAOK inhibitor **1** was next profiled, for the first time, in a battery of *in vitro* DMPK assays, where it showed moderate predicted hepatic clearance in both human (CL_hep_ = 13 mL/min/kg) and rat (CL_hep_ = 36 mL/min/kg) and low unbound fraction in human (*f*_u_ = 0.016) and rat (*f*_u_ = 0.011) plasma as well as rat brain (*f*_u_ = 0.018). In a rat plasma:brain level (PBL) PK cassette study, there was a clear *in vitro:in vivo* correlation (IVIVC) disconnect, with **1** displaying low clearance (CL_p_ = 13.9 mL/min/kg) and low volume (*V*_ss_ = 0.45 L/kg), resulting in a short half-life (*t*_1/2_ = 0.45 h). However, **1** was CNS-penetrant with a *K*_p_ of 0.61 and a *K*_p,uu_ of 1.0, highlighting **1** as a viable lead for further optimization. Another key question was with respect to the single enantiomers of **1** and would there be enantioselective TAOK inhibition. The majority of the TAOK activity resided in the (*S*)-**1** enantiomer (TAOK1 IC_50_ = 0.9 nM, TAOK2 IC_50_ = 3.2 nM), whereas the (*R*)-**1** enantiomer was weak (TAOK1 IC_50_ = 564 nM, TAOK2 IC_50_ = 2,740 nM). Thus, future optimization would have to take stereochemistry into consideration.

**Scheme 1:**
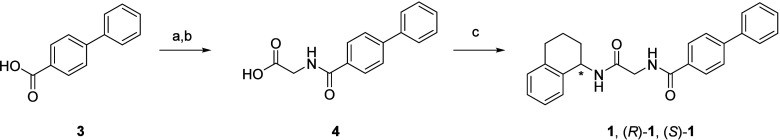
Synthesis of Racemic Compound 43 (**1**) and Its Single Enantiomers (*R*)-**1** and (*S*)-**1** ^a^

a Reagents and conditions: (a) glycine methyl ester hydrochloride, HATU, DIPEA, DMF, rt, 82%; (b) 2*N* NaOH, 1,4-dioxane, 80 °C, quant; (c) racemic, (*R*)- or (*S*)-1,2,3,4-tetrahydronaphthalen-1-amine, HATU, DIPEA, DMF, rt, 29−32%.

With **1** as a *bona fide* lead compound, we envisioned a multidimensional SAR exploration of **1** that could be readily accomplished through a series of iterative libraries (Figure 2) with an eye toward improving physiochemical/DMPK properties and engendering isoenzyme selectivity profiles as well as a hope to identify ‘molecular switches’ that could engender TAOK activation. To expedite the characterization of a large number of analogs, our screening paradigm first evaluated percent TAOK enzyme activity at a single 10 μM concentration at TAOK1 and TAOK2 (as these are the isoenzymes we were most interested in for NDD dissection), followed by full IC_50_ determination for active compounds. In many instances, we would first assess racemic compounds and then screen both single enantiomers based on a potency cutoff, as enantiopreference might change with significant structural changes. Analog **8** of TAOK inhibitor **1** is prepared in three steps from commercial materials, according to Scheme 2. Natural and unnatural *N*-Boc amino acid **5** was coupled to various racemic or chiral primary amines via an HATU-mediated sequence to provide **6**, followed by deprotection to yield derivative **7**. A subsequent HATU-mediated coupling between **7** and various carboxylic acids afforded the final analogs **8−11** in good to modest overall yield. SAR highlights from this endeavor for TAOK1 and TAOK2 inhibitions are shown in Table 1.

**Figure 2:**
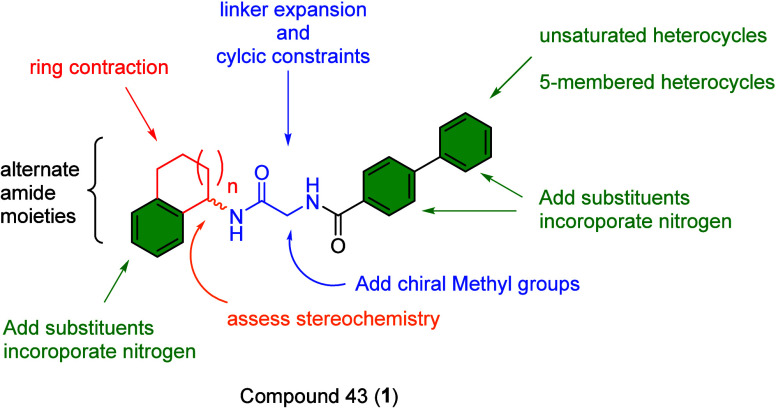
Multidimensional chemical optimization plan for compound 43 (**1**).

**Scheme 2:**
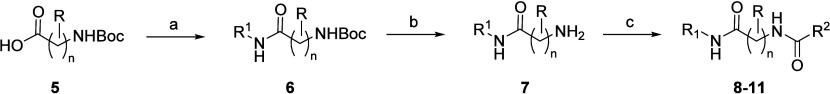
Synthesis of TAOK Inhibitor Analog **8** ^a^

a Reagents and conditions: (a) R^1^NH_2_, HATU, TEA, DMF, rt, 43−79%; (b) TFA, DCM, rt, quant; (c) aryl carboxylic acid, HATU, DIPEA, DMF, rt, 12−71%.

**Table 1:**
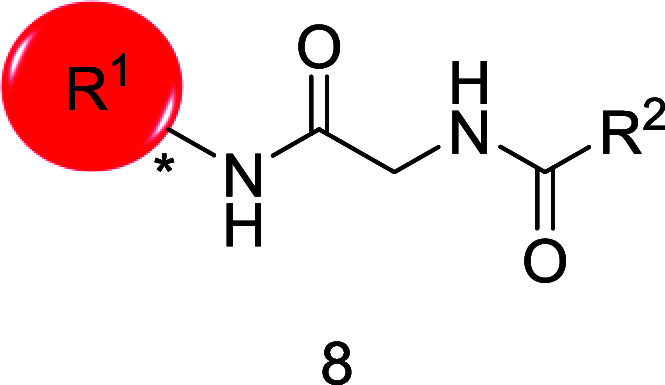

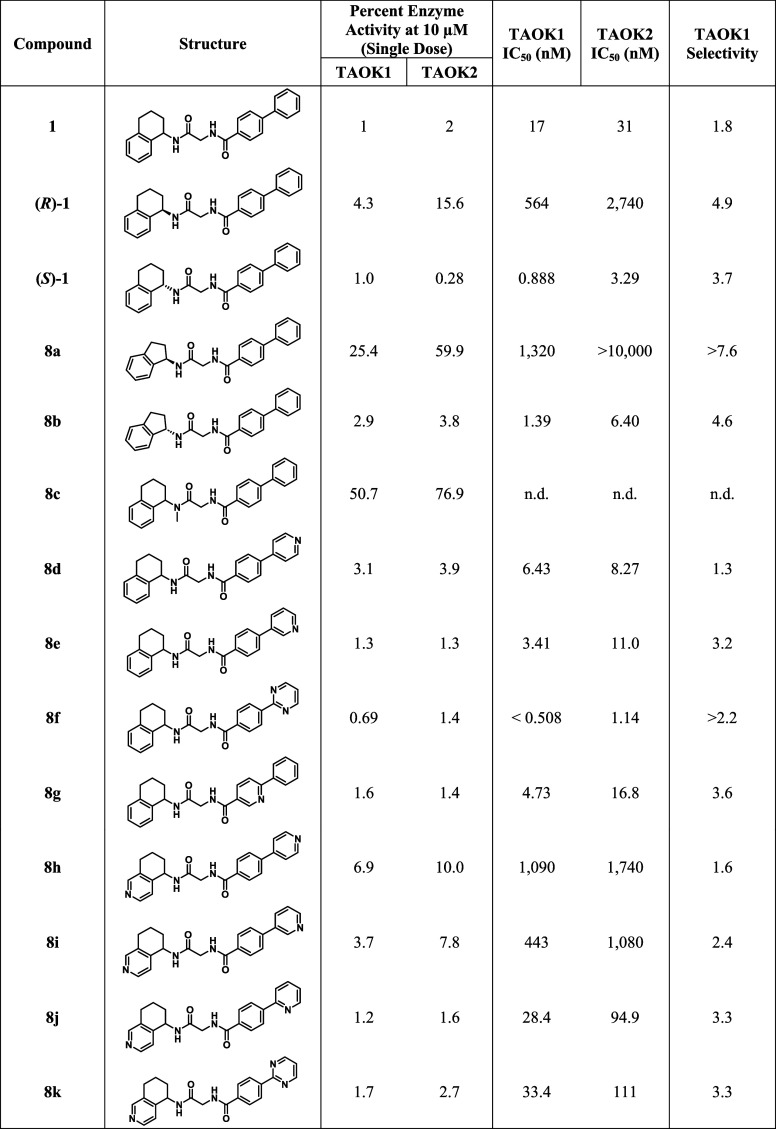
TAOK1 and TAOK2 Inhibitory Activities of Glycine-Based Analog **8** ^a^

a Inactive = No observed activity up to 10 μM (% activity, ≥85% at the highest dose). n.d. = not determined.

Several interesting SAR trends emerged from this initial library of glycine-based analog **8**. Contraction of the six-membered 1,2,3,4-tetrahydronaphthalen-1-amine in **1** to the 2,3-dihydro-1*H*-indene-1-amine congener retained activity and the same enantiopreference (e.g., **8a**, the (*R*)-enantiomer, TAOK1 IC_50_ = 1,329 nM, TAOK2 IC_50_ > 10,000 nM; **8b**, the (*S*)-enantiomer, TAOK1 IC_50_ = 1.3 nM, TAOK2 IC_50_ = 6.4 nM). Conversion of **1** to the tertiary amide **8c** led to a complete loss of TAOK inhibitory activity. The distal phenyl ring of the 4-biphenyl amide in **1** could be replaced with basic amines, such as a 4-pyridyl congener **8d** (TAOK1 IC_50_ = 6.4 nM, TAOK2 IC_50_ = 8.2 nM), a 3-pyridyl moiety **8e** (TAOK1 IC_50_ = 3.4 nM, TAOK2 IC_50_ = 11 nM), or a nonbasic 2-pyrimidine **8f** affording a very potent analog (TAOK1 IC_50_ < 0.5 nM, TAOK2 IC_50_ = 1.1 nM). Moving the basic nitrogen into the benzamide ring as in **8g** was also tolerated (TAOK1 IC_50_ = 4.7 nM, TAOK2 IC_50_ = 16.3 nM), providing multiple positions to impart solubility and improve properties. In addition, the western 1,2,3,4-tetrahydronaphthalen-1-amine moiety could also tolerate the addition of a pyridine nitrogen (eg., **8h**−**k**), with varying degrees of success; however, this change in combination with the 2-pyrimidine of **8f** affords a reasonable TAOK inhibitor (TAOK1 IC_50_ = 33.4 nM, TAOK2 IC_50_ = 111 nM).

We next explored the impact of homologating the glycine linker to a β-alanine motif as in analog **9** (Table 2, highlighted in blue) to assess the impact of greater conformational flexibility. The direct analog **9a** of **1** was inactive at both TAOK1 and TAOK2, as was a 3-biphenyl regioisomer **9b**, synthesized to determine if the flexible linker altered the trajectory of the classic 4-biphenyl. The removal of the distal phenyl ring to afford the truncated 4-toluyl analog **9c** was also inactive at both TAOK isoenzymes.

**Table 2:**
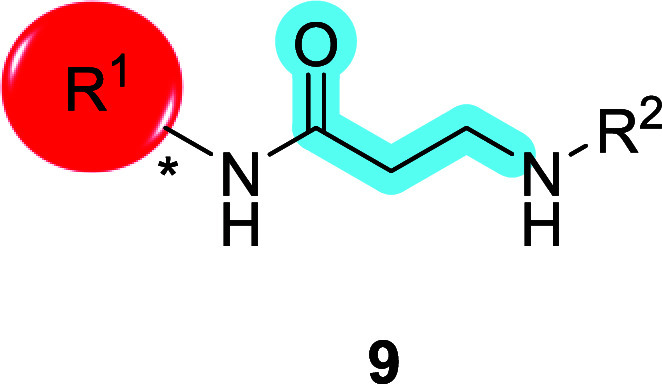

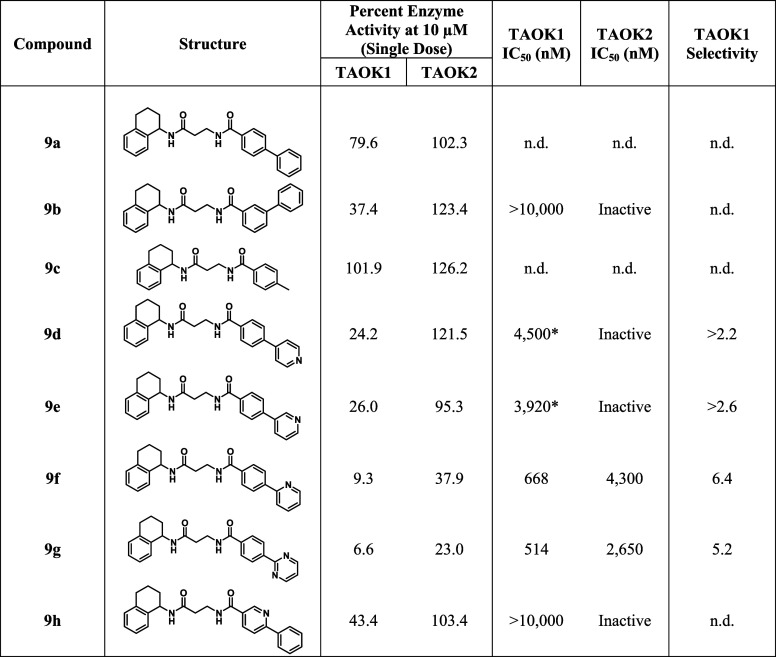
TAOK1 and TAOK2 Inhibitory Activities of β-Alanine-Based Analog **9** ^a^

a Inactive = No observed activity up to 10 μM (% activity, ≥85% at the highest dose). n.d. = not determined.

The addition of a pyridyl moiety, as in **9d** and **9e**, rescued TAOK1 activity (TAOK1 IC_50_s of 4.5 and 3.9 μM, respectively) with no inhibitory activity measured at TAOK2. The addition of nitrogen atoms into the 4-biphenyl motif as with **9f** (TAOK1 IC_50_ = 668 nM, TAOK2 IC_50_ = 4,300 nM) and **9g** (TAOK1 IC_50_ = 514 nM, TAOK2 IC_50_ = 2,650 nM) enhanced TAOK inhibitory activity, with TAOK1 preference, in the context of the β-alanine linker. Meanwhile, attempts at installing a linker between the right-hand biaryl rings (e.g., internal alkyne, methylene, or ether) led to abolished activity, as did the replacement of the right-hand amide with a sulfonamide (**9i−9l**, see the Supporting Information). We elected to initiate deeper profiling of **9d** and found that it was also inactive at TAOK3. Inhibitor **9d** displayed high predicted hepatic clearance for both human (CL_hep_ = 17 mL/min/kg) and rat (CL_hep_ = 59 mL/min/kg) but showed good unbound fraction in human plasma (*f*_u_ = 0.07), rat plasma (*f*_u_ = 0.05), and rat brain (*f*_u_ = 0.45). *In vivo* rat PK demonstrated a good IVIVC, with moderate plasma clearance (CL_p_ = 46.3 mL/min/kg) and with a moderate volume (*V*_ss_ = 0.72 L/kg), leading to a short half-life (*t*_1/2_ = 0.25 h); however, **9d** also showed moderate CNS penetration (*K*_p_ = 0.10, *K*_p,uu_ = 0.09). We then screened **9d** against a panel of 70 kinases to determine if these structural changes impacted kinome selectivity. Inhibitor **9d** only returned <80% kinase activity relative to control for 10 kinases at 10 μM (aurora A (78.1%), CDK7 (75.9%), CLK2 (29.6%), DYRK1a (21.1%), FLT3 (68.8%), HIPK2 (65%), MNK2 (67.2%), PIM3 (78%), PKA (74.5%), and ROCK1 (57%)), and thus the kinome selectivity was maintained; however, the two most potent kinase activities (CLK2 (29.6%) and DYRK1/DYRK1A (21.1%)) were inactive against the lead **1** and have been engendered by the homologation of the linker. The synthesis of the single enantiomers of **9d**, (*S*)-**9d** (TAOK1 IC_50_ = 1.5 μM) and (*R*)-**9d** (TAOK1 IC_50_ = 6.5 μM), indicated that the preference for the (*S*)-enantiomer was maintained in the homologated β-alanine series, and both enantiomers were inactive at TAOK2 and TAOK3 (Figure 3). Excited by these findings, we wondered if further modification to **9d**, with functionalized pyridine and other five- and six-membered heterocycles, could provide the first example of a TAOK1 selective inhibitor.

**Figure 3:**
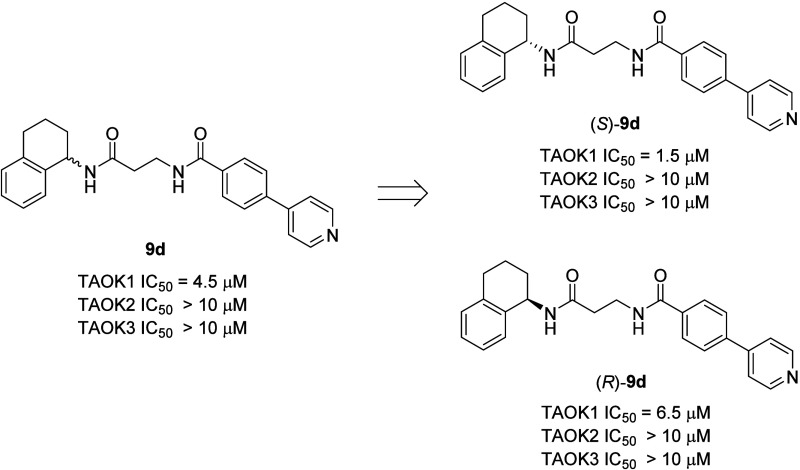
Single enantiomers of **9d** once again indicated that the (*S*)-enantiomer, (*S*)-**9d**, was a more potent and selective TAOK1 inhibitor than the racemate **9d** or the (*R*)-enantiomer, (*R*)-**9d**.

**Table 3:**
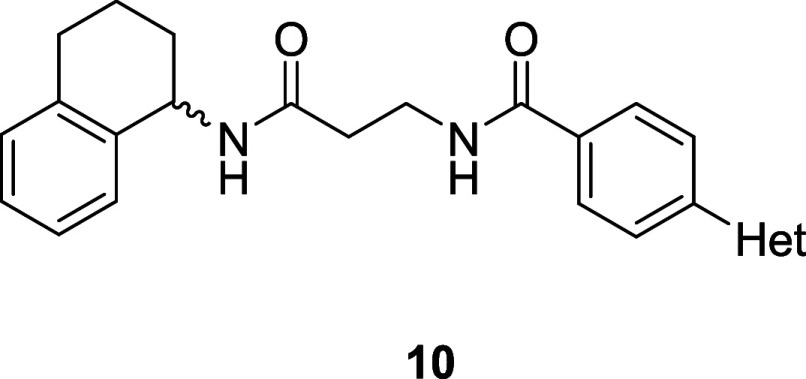

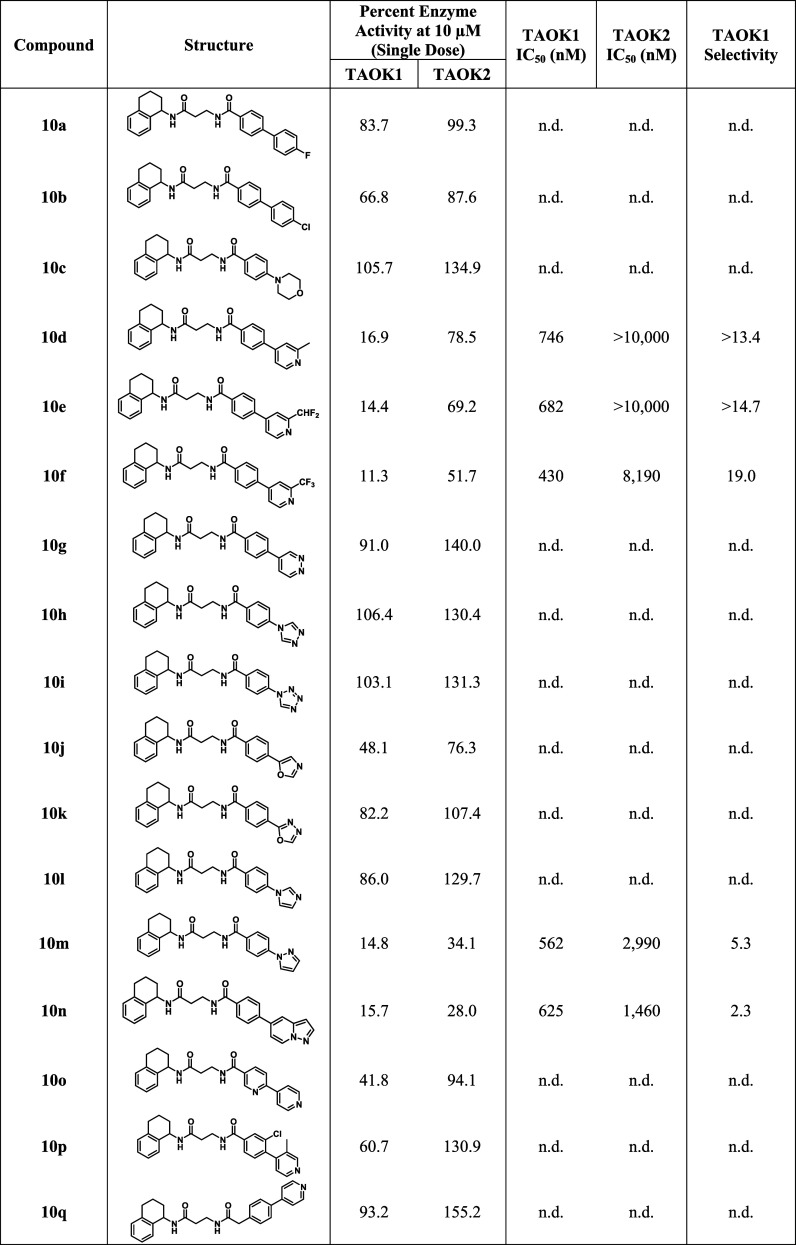
TAOK1 and TAOK2 Inhibitory Activities of Analog **10** ^a^

a n.d. = not determined.

As shown in Table 3, this exercise led to mostly inactive derivatives, wherein functionalized aryl rings (**10a** and **10b**) and diverse five-membered heterocycles (**10h**−**l**) were devoid of TAOK inhibitory activity. Notably, the addition of either a CH_3_ (**10d**), CHF_2_ (**10e**), or CF_3_ (**10f**) moiety to the pyridine ring enhanced TAOK1 activity (TAOK1 IC_50_s of 746, 682, and 430 nM, respectively) with minimal activity at TAOK2 (13- to 19-fold TAOK1 selectivity). Deeper profiling of **10f** across the TAOK isoenzymes indicated that **10f** was highly selective for TAOK1 (TAOK1 IC_50_ = 430 nM, TAOK2 IC_50_ = 8.2 μM (19-fold TAOK1 selectivity), TAOK3 IC_50_ = 8.8 μM (20-fold TAOK1 selectivity)). In our tier 1 *in vitro* DMPK panel, **10f** displayed high predicted hepatic clearance for both human (CL_hep_ = 19.9 mL/min/kg) and rat (CL_hep_ = 52.9 mL/min/kg) but showed good unbound fraction in human plasma (*f*_u_ = 0.022) and rat plasma (*f*_u_ = 0.038) and low in rat brain (*f*_u_ = 0.008). *In vivo* rat PK demonstrated a good IVIVC, with high plasma clearance (CL_p_ = 89.9 mL/min/kg) and a high volume (*V*_ss_ = 5.7 L/kg), leading to a moderate half-life (*t*_1/2_ = 0.88 h); however, **10f** also showed CNS penetration (*K*_p_ = 0.22, *K*_p,uu_ = 0.05).

**Figure 4:**
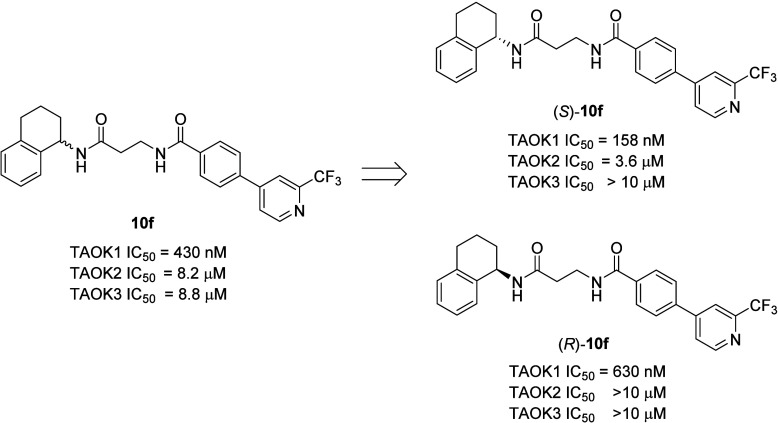
Single enantiomers of **10f** once again indicated that the (*S*)-enantiomer, (*S*)-**10f**, was a more potent and selective TAOK1 inhibitor than the racemate **10f** or the (*R*)-enantiomer, (*R*)-**10f**. (*S*)-**10f** is the most potent and selective (22.7-fold versus TAOK2 and >63.2-fold versus TAOK3).

The synthesis of the single enantiomers of **10f**, (*S*)-**10f** (TAOK1 IC_50_ = 158 nM) and (*R*)-**10f** (TAOK1 IC_50_ = 630 nM), indicated that the preference for the (*S*)-enantiomer (VU6083859) was maintained here as well, and both enantiomers were weak to inactivity at TAOK2 and TAOK3 (Figure 4), affording >20- to >60-fold selectivity for TAOK1. In tier 1 *in vitro* DMPK assays, (*S*)-**10f** was comparable to **10f**, showing high predicted hepatic clearance for human (CL_hep_ = 19.6 mL/min/kg) and for rat (CL_hep_ = 52.5 mL/min/kg), with low unbound fraction in human plasma (*f*_u_ = 0.012), moderate in rat plasma (*f*_u_ = 0.023), and low in rat brain (*f*_u_ = 0.007). (*S*)-**10f** displayed a robust IVIVC with a rat CL_p_ of 53.7

**Figure 5:**
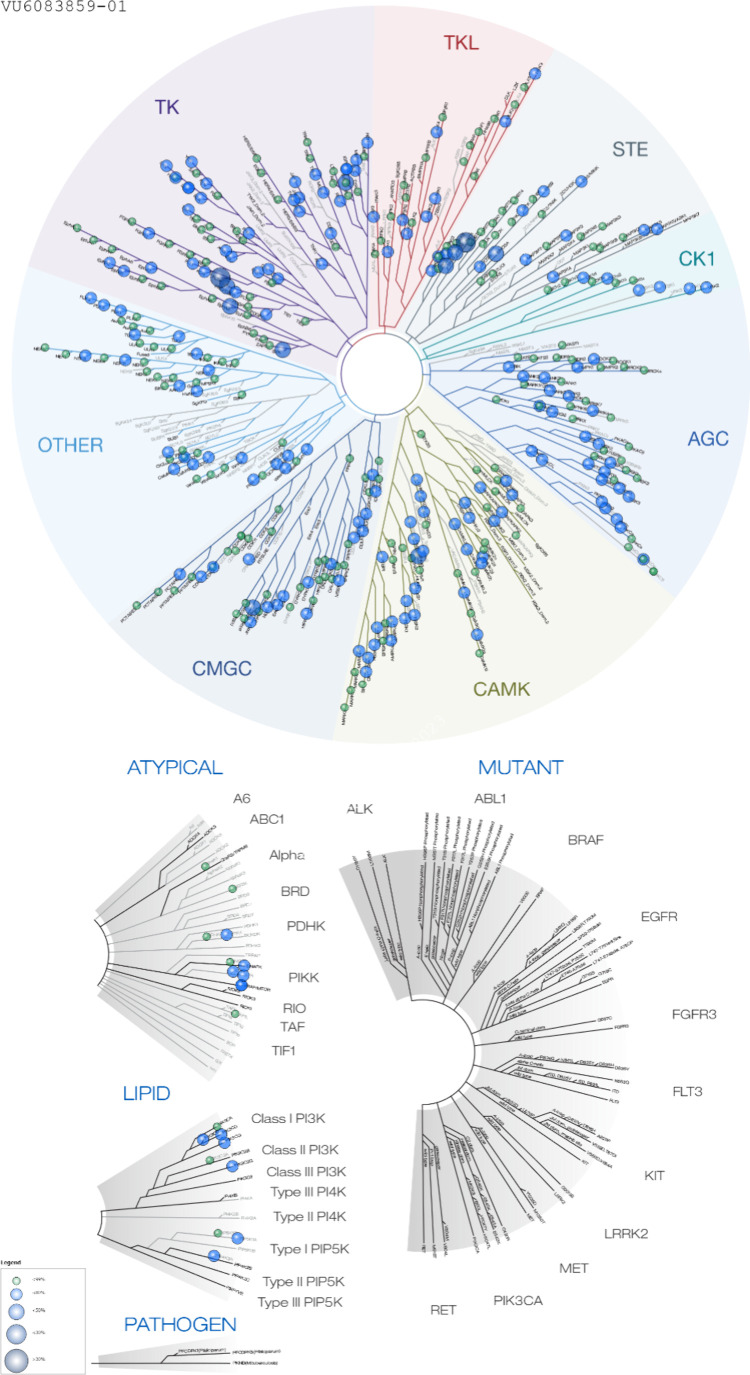
Kinome selectivity of (*S*)-**10f** (VU6083859) showing a clean kinome ancillary panel. The full data is in the Supporting Information.

mL/min/kg, a *t*_1/2_ of 0.94 h, and a *K*_p_ of 0.15 (*K*_p,uu_ of 0.05). TAOK1 inhibitor (*S*)-**10f** was then profiled at Reaction Biology in a 390 kinase panel monitoring kinase activity at 10 μM, and we found that inhibitor (*S*)-**10f** only returned <80% kinase activity relative to control for 4 kinases out of 390 at 10 μM (EphA2 (79%), MYO3A (76%), SAPK2a (75%), and Syk (79%)), in addition to TAOK1 (32%), TAOK2 (55%), and TAOK3 (73%), as expected (Figure 5). Interestingly, (*S*)-**10f** did not inhibit CLK2 or DYRK1a as did **9d**; moreover, exquisite kinome selectivity was maintained. Thus, the first TAOK1 inhibitor ((*S*)-**10f**, VU6083859) suitable for *in vitro* and *in vivo* studies has been developed.

In parallel, we were evaluating the impact of substituents and cyclic constraints within the β-alanine linker. Thus, employing the basic core of **9d** for this exercise, we generated analog **11** (Table 4). Further homologation of the β-alanine to a propyl linker as in **11a** (TAOK1 IC_50_ = 65 nM, TAOK2 IC_50_ = 402 nM) was an order of magnitude more potent than the β-alanine comparator **9d** (TAOK1 IC_50_ = 4.5 μM, TAOK2 IC_50_ > 10 μM). Cyclic constraints **11b**−**k** were generally not tolerated, but diastereoselective inhibition was noted for cyclopentane pairs **11h**−**k**. Incorporation of either an (*R*)- or (*S*)-methyl group at the two-position of the β-alanine linker in either (*S*)-**9d** or (*R*)-**9d**, generating four pairs of diastereomers **11l**−**o**, unexpectedly provided a *pan*-TAOK activator **11n**, with the previously preferred (*S*)-1,2,3,4-tetrahydronaphthalen-1-amine and the (*R*)-stereochemistry at the 2-methyl (eg., *N*-((*R*)-4-oxo-4-(((*S*)-1,2,3,4-tetrahydronaphthalen-1-yl)­amino)­butan-2-yl)-4-(pyridin-4-yl) benzamide or VU6080195). Activator **11n** displayed an EC_50_ of 270 nM (156% *E*_max_) at TAOK1, an EC_50_ of 1.3 μM (274% *E*_max_) at TAOK2, and an EC_50_ of 504 nM (372% *E*_max_) at TAOK3. This ‘magic methyl’ effect is profound, as the *des*-methyl congener (*S*)-**9d** was a modest but selective (TAOK1 IC_50_ = 1.5 μM, TAOK2/TAOK3 IC_50_ *s* > 10 μM) inhibitor. The effect of methyl groups on conformational change and protein binding has been studied extensively.^15–25^ While the binding modes of any compounds within the literature or present manuscript to any TAOK isoform are not yet known, it is quite likely that the chiral methyl in **11n** induces a significant change in its lowest energy conformation, yielding a preferred binding pose quite different to that of (*S*)-**9d** and **1**. Activator **11n** also displayed high predicted hepatic clearance for human (CL_hep_ = 18.8 mL/min/kg) and modest for rat (CL_hep_ = 52.5 mL/min/kg), with good unbound fraction in human plasma (*f*_u_ = 0.058), rat plasma (*f*_u_ = 0.028), and rat brain (*f*_u_ = 0.041). *In vivo* rat PK demonstrated a good IVIVC, with high plasma clearance (CL_p_ = 65.9 mL/min/kg) and a high volume (*V*_ss_ = 10.9 L/kg), leading to a long half-life (*t*_1/2_ = 4.7 h); moreover, **11n** also showed acceptable CNS penetration (*K*_p_ = 0.15, *K*_p,uu_ = 0.22) for first-generation tool compounds. Thus, an iterative parallel synthesis, multidimensional optimization of *pan*-TAOK inhibitor **1** not only afforded the first TAOK1 selective inhibitor ((*S*)-**10f**, VU6083859) and *pan*-TAOK activator (**11n**, VU608019) tool compounds but also displayed CNS penetration, DMPK properties, and kinome selectivity suitable for early proof-of-concept studies. Clearly, there is work to be done with rat hepatocyte metabolite identification to identify metabolic ‘hot spots’ in these ligands to improve rat PK, and **11a** is a new lead from which we launched a next-generation optimization effort.

**Table 4:**
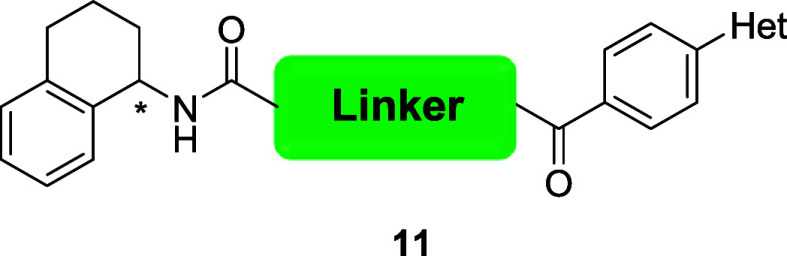

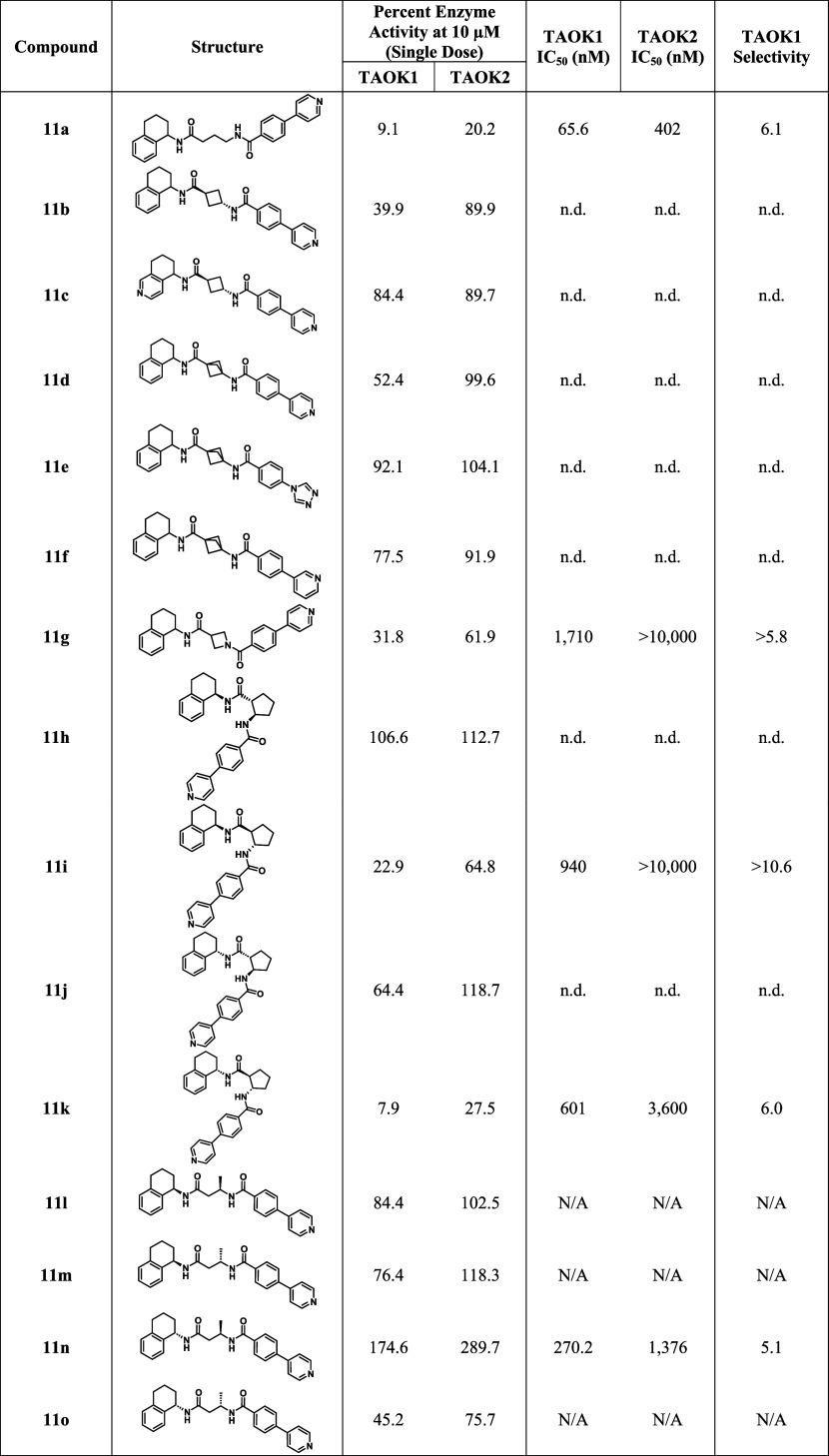
TAOK1 and TAOK2 Inhibitory Activities of Analog **11** ^a^

a n.d. = not determined.

## Conclusions

In summary, the work summarized herein describes our efforts at optimizing the activity and PK profile of *pan*-TAOK inhibitor **1** (compound 43), which quickly pivoted to the development of TAOK subtype-specific analogs, yielding the first TAOK1 selective inhibitor ((*S*)-**10f**, VU6083859) relative to TAOK2, TAOK3, and the kinome. The left-hand side of the scaffold exhibited a steep SAR, tolerating few changes to the tetrahydronaphthalene moiety. Modifications to the right-hand side of the scaffold were better tolerated, though these demonstrated significant effects on subtype specificity. Linker SAR proved to be quite sensitive to stereoisomerism, with the (1*S*,1*S*)-cyclopentyl analogs demonstrating superior activity compared to their (1*R*,1*R*)-cyclopentyl isomers. The incorporation of a single chiral methyl on TAOK inhibitor **9d**, however, led to a drastic and unexpected change in activity, with **11n** (VU608019) acting as an activator of all three TAOK isoforms, a notable example of the “magic methyl” effect. *In vitro* and *in vivo* DMPK data render both novel ligands suitable for further development as a CNS-penetrant tool compounds. Further optimization and profiling efforts are underway and will be disclosed in due course.

## Supplementary Material



## Notes

Additional experimental details, methods for the synthesis and characterization of all compounds, *in vitro* and *in vivo* DMPK protocols, and supplemental figures (PDF)

## Abbreviations

NDD: neurodevelopmental disorder
PK: pharmacokinetics
TAO: thousand and one kinase
