# Supplementary material for: Discovery of VU6083859, a TAOK1 Selective Inhibitor, and VU6080195, a pan-TAOK Activator
Source: ACS Chem Neurosci. 2026 Jan 14;17(3):624–34. doi: 10.1021/acschemneuro.5c00906 (PMC12879741; doi:10.1021/acschemneuro.5c00906)
Supplement: Supplementary file 1 [file cn5c00906_si_001.pdf]

Supporting Information

Discovery of VU6083859, a TAOK1 Selective Inhibitor and VU6080195, a *pan*-TAOK Activator

Daniel C. Schultz[*†1], Lauren C. Parr[†2], Hunter Sweet[1], Sean Lamb[1], Julie L. Engers[1,2,4], Nathaniel C. Napier[1,2,4], Hallie G. McKinnie[1,2,4], David Whomble[1,2,4], Valerie Kramlinger[1], Olivier Boutaud[1,2,4], Craig W. Lindsley[*1,2,3,4]

Affiliation:

[1]Warren Center for Neuroscience Drug Discovery, Vanderbilt University, Nashville, TN 37232, USA
[2]Department of Pharmacology, Vanderbilt University School of Medicine, Nashville, TN 37232, USA
[3]Department of Chemistry, Vanderbilt University, Nashville TN 37232, USA
[4]Vanderbilt Institute for Therapeutic Advances, Vanderbilt University, Nashville TN 37232, USA

[†]co-first authors, contributed equally

*To whom correspondence should be addressed at daniel.schultz13@yahoo.com and craig.lindsley@vanderbilt.edu

Chemistry S2

General Experimental Procedures S2

Synthesis S3

NMR Spectra of Final Compounds S43

TAOK1 and 2 Activities of Selected Compounds S123

DMPK S125

HotSpot Kinase Assay Protocol S129

Eurofins Profiling Data S131

Chemistry - General Experimental Procedures

All reactions were carried out employing standard chemical techniques. Solvents used for extraction, washing, and flash column chromatography were ACS reagent grade. Solvents for purification via HPLC were HPLC grade. All reagents were purchased from commercial sources and were used without further purification.

All NMR spectra were recorded on a 400 MHz Bruker AV-400 instrument. ^1H chemical shifts are reported as δ values in ppm relative to the residual solvent peak ($CDCl_3$ = 7.26, MeOD-d_4 = 3.31, DMSO-d_6 = 2.50, Acetone-d_6 = 2.05). Data are reported as follows: chemical shift, multiplicity (br = broad, s = singlet, d = doublet, t = triplet, q = quartet, q = quintet, dd = doublet of doublets, ddd = doublet of doublet of doublets, td = triplet of doublets, m = multiplet), coupling constant, and integration. ^{13}C chemical shifts are reported as δ values in ppm relative to the residual solvent peak ($CDCl_3$ = 77.16, MeOD-d_4 = 49.00, DMSO-d_6 = 39.52, Acetone-d_6 = 29.84).

Low resolution mass spectra were obtained on a Waters QDa (Performance) SQ MS with ESI source. *Acidic Method (Waters QDa (Performance) SQ MS)*: MS parameters were as follows: cone voltage: 15 V, capillary voltage: 0.8 kV, probe temperature: 600° C. Samples were introduced via an Acquity I-Class PLUS UPLC comprised of a BSM, FL-SM, CH-A, and PDA. UV absorption was generally observed at 215 nm and 254 nm; 4 nm bandwidth. Column: Waters Acquity BEH C18, 1.0 x 50 mm, 1.7 um. Column temperature: 55° C. Flow rate: 0.35 mL/min. Default gradient: 5% to 95% CH_3CN (0.05% TFA) in H_2O (0.05% TFA) over 1.4 min , hold at 95% CH_3CN for 0.1 min. *Basic Method (Waters QDa (Performance) SQ MS):* MS parameters were as follows: cone voltage: 15 V, capillary voltage: 0.8 kV, probe temperature: 600° C. Samples were introduced via an Acquity I-Class PLUS UPLC comprised of a BSM, FL-SM, CH-A, and PDA. UV absorption was generally observed at 215 nm and 254 nm with a 4 nm bandwidth. Column: Waters Acquity BEH C18, 2.1 x 50 mm, 1.7 um. Column temperature: 55° C. Flow rate: 1.0 mL/min. Default gradient: 5% to 95% CH_3CN in H_2O (5 mM NH_4HCO_3) over 1.4 min, hold at 95% CH_3CN for 0.1 min. Data was acquired and analyzed using MassLynx and OpenLynx software.

High resolution mass spectra were obtained on an Agilent 6540 UHD Q-TOF with ESI source. MS parameters were as follows: fragmentor: 150, capillary voltage: 3500 V, nebulizer pressure: 60 psig, drying gas flow: 13 L/min, drying gas temperature: 275° C. Samples were introduced via an Agilent 1290 UHPLC comprised of a G4220A binary pump, G4226A ALS, G1316C TCC, and G4212A DAD with ULD flow cell. UV absorption was observed at 215 nm and 254 nm with a 4 nm bandwidth. Column: Agilent Zorbax Extend C18, 1.8 μm, 2.1 x 50 mm. Gradient conditions: 5% to 95% CH_3CN in H_2O (0.1% formic Acid) over 1 min, hold at 95% CH_3CN for 0.1 min, 0.5 mL/min, 40° C.

Automated flash column chromatography was performed on a Teledyne ISCO CombiFlash system.

Final compounds were purified using a Gilson preparative reversed-phase HPLC system comprised of a 333 aqueous pump with solvent-selection valve, 334 organic pump, GX-271 or GX-281 liquid hander, two column switching valves, and a 155 UV detector. UV wavelength for fraction collection was user-defined, with absorbance at 254 nm always monitored. Column: Phenomenex Axia-packed Gemini C18, 30 x 50 mm, 5 um.

Mobile phase: CH_3CN in H_2O (0.05% v/v NH_4OH). Gradient conditions: 2.25 min equilibration, followed by user-defined gradient (starting organic percentage, ending organic percentage, duration), hold at 95% CH_3CN in H_2O (0.05% v/v NH_4OH) for 1 min, 50 mL/min, 23° C.

Synthesis

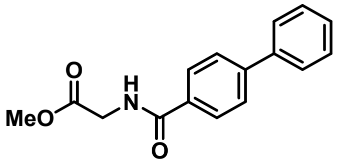

Preparation of methyl ([1,1'-biphenyl]-4-carbonyl)glycinate. To a vial was added 4-phenylbenzoic acid (379.3 mg, 1.91 mmol, 1.2 eq.), HATU (725.4 mg, 1.91 mmol, 1.2 eq.), anhydrous DMF (4 mL), and *N*,*N*-diisopropylethylamine (832 µL, 4.78 mmol, 3 eq.). The solution was stirred at room temperature for 10 minutes, then glycine methyl ester hydrochloride (205.4 mg, 1.64 mmol, 1.0 eq.) was added. The reaction was stirred at room temperature for 80 minutes. The solution was then diluted with 25 mL EtOAc and washed with 25 mL H_2O followed by 25 mL brine. The organic layer was concentrated, and the crude material was purified via automated flash column chromatography (Teledyne ISCO, 24 g column, solid loading on celite, 0-70% EtOAc/Hex) to afford the title compound as an off-white solid (362.9 mg,1.35 mmol, 82% yield).
^{1}H NMR (400 MHz, $CDCl_3$) δ 7.92 – 7.87 (m, 2H), 7.70 – 7.65 (m, 2H), 7.64 – 7.59 (m, 2H), 7.50 – 7.44 (m, 2H), 7.42 – 7.37 (m, 1H), 6.68 (t, *J* = 4.5 Hz, 1H), 4.29 (d, *J* = 5.0 Hz, 2H), 3.82 (s, 3H).
^{13}C NMR (101 MHz, $CDCl_3$) δ 170.7, 167.3, 144.8, 140.1, 132.5, 129.1, 128.2, 127.8, 127.5, 127.4, 52.7, 41.9.
HRMS (ESI-TOF) calculated for $C_{16}H_{16}NO_3$ $[M+H]^+$ = 270.1125, found = 270.1125.

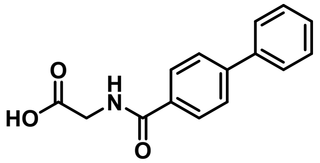

Preparation of ([1,1'-biphenyl]-4-carbonyl)glycine (4). To a vial containing ([1,1'-biphenyl]-4-carbonyl)glycinate (362.9 mg, 1.35 mmol, 1.0 eq.) was added 1,4-dioxane (3.4 mL) and 2N NaOH (2.7 mL, 5.4 mmol, 4.0 eq.). The solution was heated to 60 °C for 1 hour, by which time the reaction was complete. The reaction was concentrated, then 1N HCl was added, followed by DCM. The insoluble material was collected via vacuum filtration, washing with 2N HCl and DCM, then dried in a hi-vac oven to afford the desired product as a white solid in quantitative yield. The product was taken directly to the next step without purification.
^{1}H NMR (400 MHz, DMSO-*d*$_6$) δ 8.79 – 8.66 (m, 1H), 7.98 – 7.93 (m, 2H), 7.82 – 7.76 (m, 2H), 7.76 – 7.71 (m, 2H), 7.53 – 7.46 (m, 2H), 7.44 – 7.38 (m, 1H), 3.90 – 3.84 (m, 2H).
^{13}C NMR (101 MHz, DMSO-*d*$_6$) δ 171.2, 165.9, 142.8, 139.1, 132.8, 129.0, 128.1, 127.9, 126.9, 126.6, 41.8.
HRMS (ESI-TOF) calculated for $C_{15}H_{14}NO_3$ $[M+H]^+$ = 256.0968, found = 256.0967.

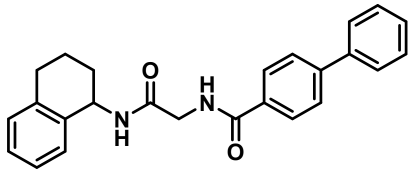

***N*-(2-oxo-2-((1,2,3,4-tetrahydronaphthalen-1-yl)amino)ethyl)-[1,1'-biphenyl]-4-carboxamide (Compound 43, VU6063661, 1).** To a test tube was added ([1,1'-biphenyl]-4-carbonyl)glycine (322.0 mg, 1.26 mmol, 1 eq.), HATU (575.5 mg, 1.51 mmol, 1.2 eq.), 1,2,3,4-tetrahydro-1-naphthylamine hydrochloride (278.0 mg, 1.51 mmol, 1.2 eq.), anhydrous DMF (6.3 mL), and DIPEA (879 µL, 5.04 mmol, 4 eq.). The reaction was stirred at room temperature. Upon completion, the crude material was purified via RP-HPLC (20-70% MeCN in 0.5 mL/L AQ NH_4OH) to afford the title compound as a white solid (154.0 mg, 0.401 mmol, 32% yield).
^{1}H NMR (400 MHz, DMSO-*d*$_6$) δ 8.79 (t, *J* = 5.9 Hz, 1H), 8.30 (d, *J* = 8.7 Hz, 1H), 8.02 – 7.96 (m, 2H), 7.82 – 7.76 (m, 2H), 7.76 – 7.71 (m, 2H), 7.53 – 7.45 (m, 2H), 7.44 – 7.38 (m, 1H), 7.24 – 7.05 (m, 4H), 5.06 – 4.96 (m, 1H), 3.95 (d, *J* = 5.9 Hz, 2H), 2.81 – 2.65 (m, 2H), 1.95 – 1.81 (m, 2H), 1.78 – 1.64 (m, 2H).

^{13}C NMR (101 MHz, DMSO-*d_6*) δ 168.4, 166.1, 142.8, 139.2, 137.5, 137.0, 132.9, 129.1, 128.7, 128.1, 126.9, 126.7, 126.5, 125.8, 46.6, 42.7, 29.9, 28.8, 20.2. Note: 2 aromatic signals not present, likely buried or overlapping.
HRMS (ESI-TOF) calculated for $C_{25}H_{25}N_2O_2$ $[M+H]^+$ = 385.1911, found = 385.1910.

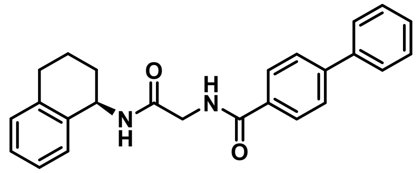

General Procedure A: Preparation (*R*)-*N*-(2-oxo-2-((1,2,3,4-tetrahydronaphthalen-1-yl)amino)ethyl)-[1,1'-biphenyl]-4-carboxamide ((*R*)-1). To a vial was added ([1,1'-biphenyl]-4-carbonyl)glycine (25.8 mg, 0.101 mmol, 1.1 eq.), HATU (41.3 mg, 0.109 mmol, 1.1 eq.), anhydrous DMF (1 mL), and DIPEA (49.8 µL, 0.286 mmol, 3.0 eq.). The solution was stirred at room temperature for 10 minutes, then (*R*)-(-)-1,2,3,4-tetrahydronaphthalen-1-amine (13.5 µL, 95.4 µmol, 1.0 eq.) was added. The reaction was stirred at room temperature for 4 hours, then was quenched with H_2O, extracted with several portions of EtOAc and DCM, and concentrated. The crude material was purified via automated flash column chromatography (0-100% EtOAc/Hex) to afford the title compound as a white solid (9.4 mg, 24.4 µmol, 26% yield).
^{1}H NMR (400 MHz, DMSO-*d6*) δ 8.79 (t, *J* = 5.9 Hz, 1H), 8.30 (d, *J* = 8.7 Hz, 1H), 8.02 – 7.96 (m, 2H), 7.82 – 7.77 (m, 2H), 7.76 – 7.71 (m, 2H), 7.53 – 7.46 (m, 2H), 7.44 – 7.38 (m, 1H), 7.24 – 7.06 (m, 4H), 5.06 – 4.97 (m, 1H), 3.95 (d, *J* = 5.9 Hz, 2H), 2.81 – 2.65 (m, 2H), 1.95 – 1.81 (m, 2H), 1.79 – 1.64 (m, 2H).
^{13}C NMR (101 MHz, DMSO-*d6*) δ 168.4, 166.1, 142.8, 139.2, 137.5, 137.0, 132.9, 129.1, 128.7, 128.1, 126.9, 126.7, 126.5, 125.8, 46.6, 42.7, 29.9, 28.8, 20.2. Note: 2 aromatic signals not present, likely buried or overlapping.
HRMS (ESI-TOF) calculated for $C_{25}H_{25}N_2O_2$ $[M+H]^+$ = 385.1911, found = 385.1908.

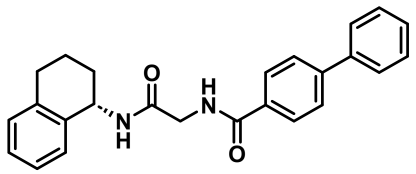

(*S*)-*N*-(2-oxo-2-((1,2,3,4-tetrahydronaphthalen-1-yl)amino)ethyl)-[1,1'-biphenyl]-4-carboxamide ((*S*)-1). Prepared in a similar manner as General Procedure A using (*S*)-(+)-1,2,3,4-tetrahydro-1-naphthylamine (13.5 µL, 95.4 µmol, 1.0 eq.) to afford the title compound as an off-white solid (6.0 mg, 15.6 µmol, 16% yield).
^{1}H NMR (400 MHz, DMSO-*d_6*) δ 8.78 (t, *J* = 5.9 Hz, 1H), 8.30 (d, *J* = 8.7 Hz, 1H), 8.02 – 7.96 (m, 2H), 7.82 – 7.76 (m, 2H), 7.76 – 7.71 (m, 2H), 7.53 – 7.46 (m, 2H), 7.44 – 7.38 (m, 1H), 7.24 – 7.05 (m, 4H), 5.06 – 4.97 (m, 1H), 3.94 (d, *J* = 5.9 Hz, 2H), 2.81 – 2.65 (m, 2H), 1.94 – 1.82 (m, 2H), 1.79 – 1.65 (m, 2H).
^{13}C NMR (101 MHz, DMSO-*d_6*) δ 168.4, 166.1, 142.8, 139.2, 137.5, 137.0, 132.9, 129.0, 128.7, 128.1, 126.9, 126.7, 126.5, 125.8, 46.6, 42.7, 29.9, 28.8, 20.2. Note: 2 aromatic signals not present, likely buried or overlapping.
HRMS (ESI-TOF) calculated for $C_{25}H_{25}N_2O_2$ $[M+H]^+$ = 385.1911, found = 385.1911.

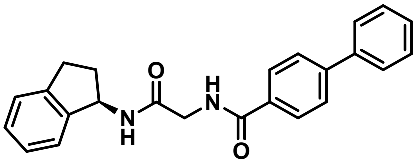

(*R*)-*N*-(2-((2,3-Dihydro-1*H*-inden-1-yl)amino)-2-oxoethyl)-[1,1'-biphenyl]-4-carboxamide (8a): Prepared in a similar manner as General Procedure A using (*R*)-2,3-dihydro-1*H*-inden-1-amine hydrochloride (20.6 mg, 0.121 mmol, 1.0 eq.) to afford the title compound as a white solid (4.4 mg, 11.9 µmol, 10% yield).

^{1}H NMR (400 MHz, DMSO-d_6) δ 8.79 (t, *J* = 5.9 Hz, 1H), 8.32 (d, *J* = 8.4 Hz, 1H), 8.03 – 7.96 (m, 2H), 7.82 – 7.77 (m, 2H), 7.77 – 7.71 (m, 2H), 7.53 – 7.46 (m, 2H), 7.44 – 7.38 (m, 1H), 7.27 – 7.16 (m, 4H), 5.33 (q, *J* = 8.1 Hz, 1H), 3.95 (d, *J* = 6.0 Hz, 2H), 2.93 (ddd, *J* = 15.8, 8.8, 3.1 Hz, 1H), 2.81 (dt, *J* = 15.8, 8.4 Hz, 1H), 2.38 (dtd, *J* = 12.5, 7.8, 3.2 Hz, 1H), 1.83 (dq, *J* = 12.5, 8.8 Hz, 1H).
^{13}C NMR (101 MHz, DMSO-d_6) δ 168.8, 166.1, 144.0, 142.81, 142.77, 139.2, 132.9, 129.0, 128.1, 127.4, 126.9, 126.5, 126.3, 124.5, 123.9, 53.6, 42.7, 32.9, 29.7. Note: 1 aromatic C missing, presumably buried.
HRMS (ESI-TOF) calc. for $C_{24}H_{23}N_2O_2$ $[M+H]^+$ = 371.1754, found = 371.1754.

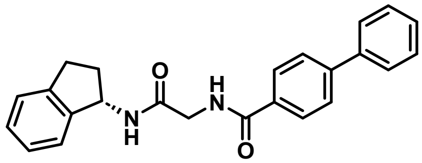

(*S*)-*N*-(2-((2,3-Dihydro-1*H*-inden-1-yl)amino)-2-oxoethyl)-[1,1'-biphenyl]-4-carboxamide (8b):
Prepared in a similar manner as General Procedure A using (*S*)-(+)-1-aminoindan (14 µL, 0.109 mmol, 1.0 eq.) to afford the title compound as a white solid (9.0 mg, 24.3 µmol, 22% yield).
^{1}H NMR (400 MHz, DMSO-d_6) δ 8.79 (t, *J* = 5.9 Hz, 1H), 8.32 (d, *J* = 8.4 Hz, 1H), 8.03 – 7.96 (m, 2H), 7.82 – 7.77 (m, 2H), 7.77 – 7.71 (m, 2H), 7.53 – 7.46 (m, 2H), 7.44 – 7.38 (m, 1H), 7.28 – 7.16 (m, 4H), 5.33 (q, *J* = 8.1 Hz, 1H), 3.95 (d, *J* = 5.9 Hz, 2H), 2.93 (ddd, *J* = 15.8, 8.8, 3.1 Hz, 1H), 2.81 (dt, *J* = 15.8, 8.4 Hz, 1H), 2.38 (dtd, *J* = 12.5, 7.8, 3.2 Hz, 1H), 1.83 (dq, *J* = 12.5, 8.8 Hz, 1H).
13C NMR (101 MHz, DMSO-d6) δ 168.7, 166.1, 144.0, 142.81, 142.77, 139.2, 132.9, 129.0, 128.1, 127.4, 126.9, 126.5, 126.3, 124.5, 123.9, 53.6, 42.7, 32.9, 29.7. Note: 1 aromatic C missing, presumably buried.
HRMS (ESI-TOF) calc for $C_{24}H_{23}N_2O_2$ $[M+H]^+$ = 371.1754, found = 371.1755.

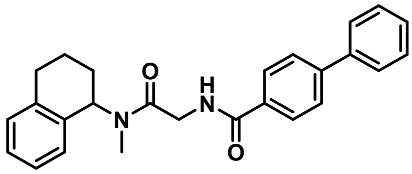

***N*-(2-(methyl(1,2,3,4-tetrahydronaphthalen-1-yl)amino)-2-oxoethyl)-[1,1'-biphenyl]-4-carboxamide (8c):**
Prepared in a similar manner as General Procedure A using *N*-methyl-1,2,3,4-tetrahydronaphthalene-1-amine hydrochloride (22.3 mg, 0.113 mmol, 1.0 eq.) to afford the title compound as an orange solid (1.7 mg, 4.3 µmol, 4% yield).
^{1}H NMR (400 MHz, DMSO-d_6): Note: a mixture of rotamers was observed. Variable NMR did not result in signal coalescence.
HRMS (ESI-TOF) calc for $C_{26}H_{27}N_2O_2$ $[M+H]^+$ = 399.2067, found = 399.2068.

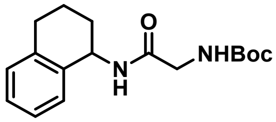

General Procedure B: Preparation of *tert*-butyl (2-oxo-2-((1,2,3,4-tetrahydronaphthalen-1-yl)amino)ethyl)carbamate. To a test tube was added *N*-(*tert*-butoxycarbonyl)glycine (98.3 mg, 0.561 mmol, 1.1 eq.) and HATU (217.5 mg, 0.572 mmol, 1.1 eq.), followed by anhydrous DMF (2 mL) and TEA (213 µL, 1.53 mmol, 3.0 eq.). The mixture was stirred at room temperature for 10 minutes, then 1,2,3,4-tetrahydro-1-naphthylamine (73.1 µL, 0.509 mmol, 1.0 eq.) was added. The reaction was stirred at room temperature for 20 hours, then was quenched with 200 µL H_2O, diluted with DMSO, and filtered through PTFE. The crude solution was purified via RP-HPLC (5-70% MeCN in 0.5 mL/L AQ NH_4OH) to afford the title compound as a clear residue (66.3 mg, 0.218 mmol, 43% yield).
^{1}H NMR (400 MHz, $CDCl_3$) δ 7.25 – 7.20 (m, 1H), 7.19 – 7.10 (m, 2H), 7.10 – 7.06 (m, 1H), 6.43 (d, *J* = 8.2 Hz, 1H), 5.30 – 5.09 (m, 2H), 3.80 (d, *J* = 5.6 Hz, 2H), 2.85 – 2.70 (m, 2H), 2.10 – 1.99 (m, 1H), 1.87 – 1.72 (m, 3H), 1.41 (s, 9H).

^{13}C NMR (101 MHz, CDCl$_3$) δ 168.8, 156.1, 137.7, 136.5, 129.3, 128.6, 127.5, 126.4, 80.4, 47.6, 44.7, 30.3, 29.3, 28.4, 20.1.
HRMS (ESI-TOF) calculated for $C_{17}H_{25}N_2O_3$ $[M+H]^+$ = 305.1860, found = 305.1863.

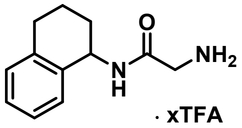

General Procedure C: Preparation of 2-amino-N-(1,2,3,4-tetrahydronaphthalen-1-yl)acetamide trifluoroacetic acid: A vial was charged with *tert*-butyl (2-oxo-2-((1,2,3,4-tetrahydronaphthalen-1-yl)amino)ethyl)carbamate (66.3 mg, 0.128 mmol, 1.0 eq.), which was then dissolved in anhydrous DCM (2 mL). To the stirring solution was added TFA (167 µL, 2.18 mmol, 10 eq.). The reaction was stirred at room temperature for 16 hours, then was concentrated to afford the title compound as a white solid in quantitative yield. Material taken to the next step without purification.
^{1}H NMR (400 MHz, DMSO-*d$_6$*) δ 8.68 (d, *J* = 8.4 Hz, 1H), 8.03 (s, 3H), 7.21 – 7.14 (m, 3H), 7.14 – 7.10 (m, 1H), 5.05 – 4.98 (m, 1H), 3.61 (d, *J* = 15.9 Hz, 1H), 3.54 (d, J = 15.9 Hz, 1H), 2.83 – 2.65 (m, 2H), 1.97 – 1.64 (m, 4H).
HRMS (ESI-TOF) calculated for $C_{12}H_{17}N_2O$ $[M+H]^+$ = 205.1335, found = 205.1333.

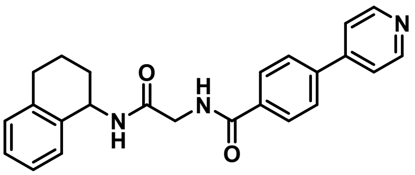

General Procedure D: Preparation of *N*-(2-oxo-2-((1,2,3,4-tetrahydronaphthalen-1-yl)amino)ethyl)-4-(pyridin-4-yl)benzamide (8d): To a test tube was added 4-pyridin-4-yl-benzoic acid (27.9 mg, 0.140 mmol, 1.3 eq.), HATU (59.0 mg, 0.155 mmol, 1.4 eq.), anhydrous DMF (1 mL), and DIPEA (95.0 µL, 0.545 mmol, 5.0 eq.). The mixture was stirred at room temp for 15 minutes, then a solution of 2-amino-*N*-(1,2,3,4-tetrahydronaphthalen-1-yl)acetamide trifluoroacetic acid (34.7 mg, 0.109 mmol, 1.0 eq.) in anhydrous DMF (1 mL) was added. The reaction was stirred at room temperature for 1.5 hours, then was quenched with 100 µL H_2O and purified via RP-HPLC (5-62% MeCN in 0.5 mL/L AQ NH_4OH) to afford the title compound as a white residue (19.3 mg, 50.1 µmol, 46% yield).
^{1}H NMR (400 MHz, DMSO-*d$_6$*) δ 8.86 (t, *J* = 5.9 Hz, 1H), 8.69 – 8.65 (m, 2H), 8.31 (d, *J* = 8.7 Hz, 1H), 8.07 – 8.01 (m, 2H), 7.97 – 7.91 (m, 2H), 7.81 – 7.76 (m, 2H), 7.24 – 7.05 (m, 4H), 5.06 – 4.96 (m, 1H), 3.95 (d, *J* = 6.0 Hz, 2H), 2.81 – 2.65 (m, 2H), 1.96 – 1.81 (m, 2H), 1.79 – 1.63 (m, 2H).
^{13}C NMR (101 MHz, DMSO-*d$_6$*) δ 168.3, 165.9, 150.4, 146.0, 139.7, 137.5, 137.0, 134.6, 128.7, 128.2, 128.1, 126.8, 126.7, 125.8, 121.3, 46.8, 42.7, 29.9, 28.8, 20.2.
HRMS (ESI-TOF) calculated for $C_{24}H_{24}N_3O_2$ $[M+H]^+$ = 386.1863, found = 386.1866.

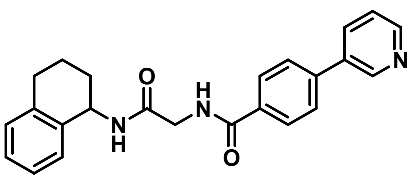

***N*-(2-oxo-2-((1,2,3,4-tetrahydronaphthalen-1-yl)amino)ethyl)-4-(pyridin-3-yl)benzamide (8e).**
Prepared according to General Procedure D using 4-pyridin-3-yl-benzoic acid (9.4 mg, 47 µmol, 1.2 eq.) to afford the title compound as a clear residue (9.0 mg, 23.3 µmol, 60% yield).
^{1}H NMR (400 MHz, DMSO-*d$_6$*) δ 8.97 (dd, *J* = 2.4, 0.6 Hz, 1H), 8.83 (t, *J* = 5.9 Hz, 1H), 8.61 (dd, *J* = 4.8, 1.5 Hz, 1H), 8.31 (d, *J* = 8.7 Hz, 1H), 8.16 (ddd, *J* = 8.0, 2.4, 1.7 Hz, 1H), 8.05 – 8.00 (m, 2H), 7.89 – 7.84 (m, 2H), 7.52 (ddd, *J* = 8.0, 4.8, 0.7 Hz, 1H), 7.24 – 7.05 (m, 4H), 5.05 – 4.97 (m, 1H), 3.95 (d, J = 5.9 Hz, 2H), 2.81 – 2.65 (m, 2H), 1.95 – 1.82 (m, 2H), 1.79 – 1.65 (m, 2H).

^{13}C NMR (101 MHz, DMSO-d_6) δ 168.4, 166.0, 149.0, 147.8, 139.7, 137.5, 137.0, 134.6, 134.3, 133.6, 128.7, 128.2, 128.1, 126.7, 126.7, 125.8, 124.0, 46.6, 42.7, 29.9, 28.8, 20.2.
HRMS (ESI-TOF) calculated for $C_{24}H_{24}N_3O_2$ $[M+H]^+$ = 386.1863, found = 386.1865.

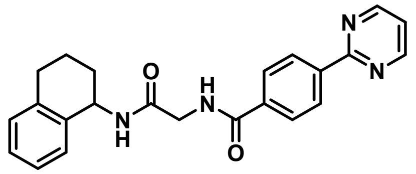

***N*-(2-oxo-2-((1,2,3,4-tetrahydronaphthalen-1-yl)amino)ethyl)-4-(pyrimidin-2-yl)benzamide (8f).** Prepared according to General Procedure D using 4-pyrimidin-2-yl-benzoic acid (25 mg, 0.12 mmol, 1.1 eq.) to afford the title compound as a white solid (30.6 mg, 79.2 µmol, 71% yield).
^{1}H NMR (400 MHz, DMSO-d_6) δ 8.95 (d, *J* = 4.9 Hz, 2H), 8.86 (t, *J* = 5.9 Hz, 1H), 8.50 – 8.45 (m, 2H), 8.32 (d, *J* = 8.7 Hz, 1H), 8.07 – 8.02 (m, 2H), 7.50 (t, *J* = 4.9 Hz, 1H), 7.24 – 7.18 (m, 1H), 7.18 – 7.12 (m, 2H), 7.11 – 7.06 (m, 1H), 5.05 – 4.97 (m, 1H), 3.95 (d, *J* = 5.9 Hz, 2H), 2.81 – 2.65 (m, 2H), 1.95 – 1.82 (m, 2H), 1.80 – 1.64 (m, 2H).
^{13}C NMR (101 MHz, DMSO-d_6) δ 168.3, 166.0, 162.6, 157.9, 139.6, 137.5, 137.0, 136.0, 128.7, 128.1, 127.9, 127.5, 126.7, 125.8, 120.4, 46.6, 42.8, 29.9, 28.8, 20.2.
HRMS (ESI-TOF) calculated for $C_{23}H_{23}N_4O_2$ 387.1816, found 387.1819.

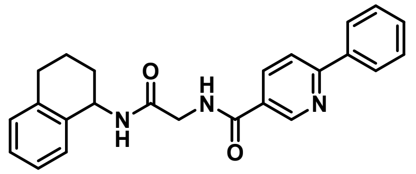

***N*-(2-oxo-2-((1,2,3,4-tetrahydronaphthalen-1-yl)amino)ethyl)-6-phenylnicotinamide (8g).** Prepared according to General Procedure D using 6-phenylnicotinic acid (9.4 mg, 47 µmol, 1.2 eq.) to afford the title compound as a white solid (3.5 mg, 8.9 µmol, 23% yield).
^{1}H NMR (400 MHz, DMSO-d_6) δ 9.13 (dd, *J* = 2.3, 0.6 Hz, 1H), 9.00 (t, *J* = 5.9 Hz, 1H), 8.37 – 8.30 (m, 2H), 8.18 – 8.13 (m, 2H), 8.10 (dd, *J* = 8.3, 0.6 Hz, 1H), 7.56 – 7.45 (m, 3H), 7.23 – 7.06 (m, 4H), 5.06 – 4.98 (m, 1H), 3.97 (d, *J* = 5.9 Hz, 2H), 2.81 – 2.65 (m, 2H), 1.94 – 1.83 (m, 2H), 1.79 – 1.66 (m, 2H).
^{13}C NMR (101 MHz, DMSO-d_6) δ 168.2, 164.9, 158.1, 148.7, 137.8, 137.5, 137.0, 136.2, 129.8, 128.9, 128.7, 128.0, 126.9, 126.7, 125.8, 119.6, 46.6, 42.7, 29.9, 28.8, 20.2. Note: 1 aromatic signal not present, likely buried or overlapping.
HRMS (ESI-TOF) calculated for $C_{24}H_{24}N_3O_2$ $[M+H]^+$ = 386.1863, found = 386.1862.

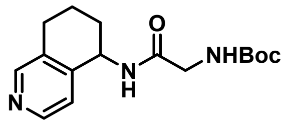

***Tert*-butyl (2-oxo-2-((5,6,7,8-tetrahydroisoquinolin-5-yl)amino)ethyl)carbamate:** Prepared according to General Procedure B using 5,6,7,8-tetrahydro-5-isoquinolinamine hydrochloride (86.2 mg, 0.467 mmol, 1.0 eq.) to afford the title compound as a yellow oil (113.2 mg, 0.371 mmol, 79% yield).
^{1}H NMR (400 MHz, CDCl$_3$) δ 8.55 – 8.28 (m, 2H), 7.77 – 7.61 (m, 2H), 5.56 (s, 1H), 5.27 – 5.16 (m, 1H), 3.92 – 3.77 (m, 2H), 2.95 – 2.82 (m, 2H), 2.21 – 2.11 (m, 1H), 2.10 – 1.99 (m, 1H), 1.96 – 1.74 (m, 2H), 1.39 (s, 9H).
^{13}C NMR (101 MHz, CDCl$_3$) δ 170.3, 156.5, 156.1, 142.5, 139.7, 136.9, 125.3, 80.5, 47.5, 44.6, 28.6, 28.4, 26.2, 20.2.
HRMS (ESI-TOF) calculated for $C_{16}H_{24}N_3O_3$ $[M+H]^+$ = 306.1812, found = 306.1810.

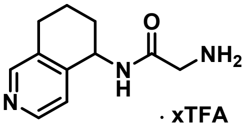

2-Amino-*N*-(5,6,7,8-tetrahydroisoquinolin-5-yl)acetamide trifluoroacetic acid: Prepared according to General Procedure C using *tert*-butyl (2-oxo-2-((5,6,7,8-tetrahydroisoquinolin-5-yl)amino)ethyl)carbamate (113.2 mg, 0.371 mmol, 1.0 eq.) to afford the title compound as a gold oil in quantitative yield. Product taken to the next step without purification.
^{1}H NMR (400 MHz, DMSO-*d₆*) δ 8.95 (d, *J* = 8.3 Hz, 1H), 8.67 (s, 1H), 8.61 (d, *J* = 5.7 Hz, 1H), 8.16 – 8.03 (m, 3H), 7.63 (d, *J* = 5.8 Hz, 1H), 5.14 – 5.06 (m, 1H), 3.71 – 3.62 (m, 2H, overlapping with H_2O signal), 2.88 (t, *J* = 6.2 Hz, 2H), 2.06 – 1.81 (m, 3H), 1.79 – 1.67 (m, 1H).
HRMS (ESI-TOF) calculated for $C_{11}H_{16}N_3O$ $[M+H]^+$ = 206.1288, found = 206.1283.

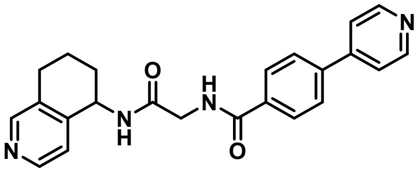

***N*-(2-oxo-2-((5,6,7,8-tetrahydroisoquinolin-5-yl)amino)ethyl)-4-(pyridin-4-yl)benzamide (8h).** Prepared according to General Procedure D using 2-amino-*N*-(5,6,7,8-tetrahydroisoquinolin-5-yl)acetamide trifluoroacetic acid (39.5 mg, 0.124 mmol, 1.0 eq.) and 4-pyridin-4-yl-benzoic acid (31.6 mg, 0.159 mmol, 1.3 eq.) to afford the title compound as a clear residue (16.3 mg, 42.2 µmol, 34% yield).
^{1}H NMR (400 MHz, DMSO-*d₆*) δ 8.91 (t, *J* = 5.8 Hz, 1H), 8.69 – 8.65 (m, 2H), 8.41 (d, *J* = 8.7 Hz, 1H), 8.34 – 8.30 (m, 2H), 8.07 – 8.02 (m, 2H), 7.96 – 7.91 (m, 2H), 7.80 – 7.76 (m, 2H), 7.19 (d, *J* = 5.1 Hz, 1H), 5.03 – 4.95 (m, 1H), 3.96 (d, *J* = 5.9 Hz, 2H), 2.76 – 2.69 (m, 2H), 1.98 – 1.87 (m, 2H), 1.83 – 1.63 (m, 2H).
^{13}C NMR (101 MHz, DMSO-*d₆*) δ 168.8, 166.0, 150.4, 149.9, 146.7, 146.5, 146.0, 139.8, 134.5, 132.7, 128.3, 126.8, 122.1, 121.4, 46.1, 42.9, 29.1, 25.5, 20.3.
HRMS (ESI-TOF) calculated for $C_{23}H_{23}N_4O_2$ = 387.1816, found = 387.1814.

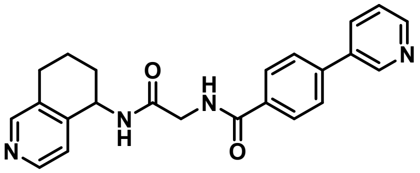

***N*-(2-oxo-2-((5,6,7,8-tetrahydroisoquinolin-5-yl)amino)ethyl)-4-(pyridin-3-yl)benzamide (8i).**
Prepared according to General Procedure D using 2-amino-*N*-(5,6,7,8-tetrahydroisoquinolin-5-yl)acetamide trifluoroacetic acid (39.5 mg, 0.124 mmol, 1.0 eq.) and 4-pyridin-3-yl-benzoic acid (30.9 mg, 0.155 mmol, 1.3 eq.) to afford the title compound as a clear residue (19.6 mg, 50.7 µmol, 41% yield).
^{1}H NMR (400 MHz, DMSO-*d₆*) δ 8.97 (dd, *J* = 2.4, 0.6 Hz, 1H), 8.88 (t, *J* = 5.9 Hz, 1H), 8.61 (dd, *J* = 4.7, 1.6 Hz, 1H), 8.40 (d, *J* = 8.8 Hz, 1H), 8.34 – 8.30 (m, 2H), 8.16 (ddd, *J* = 8.0, 2.4, 1.7 Hz, 1H), 8.06 – 8.01 (m, 2H), 7.90 – 7.85 (m, 2H), 7.52 (ddd, *J* = 8.0, 4.8, 0.7 Hz, 1H), 7.19 (d, *J* = 5.1 Hz, 1H), 5.03 – 4.94 (m, 1H), 3.96 (d, *J* = 5.8 Hz, 2H), 2.77 – 2.70 (m, 2H), 1.98 – 1.88 (m, 2H), 1.82 – 1.63 (m, 2H).
^{13}C NMR (101 MHz, DMSO-*d₆*) δ 168.8, 166.1, 149.9, 149.0, 147.8, 146.7, 146.4, 139.8, 134.6, 134.3, 133.5, 132.7, 128.2, 126.7, 124.0, 122.1, 46.1, 42.9, 29.1, 25.5, 20.3.
HRMS (ESI-TOF) calculated for $\mathbf{C_{23}H_{23}N_4O_2}$ $[M+H]^+$ = 387.1816, found = 387.1820.

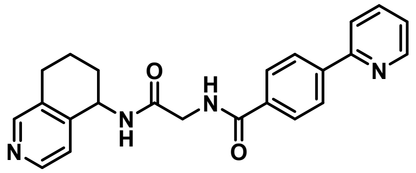

***N*-(2-oxo-2-((5,6,7,8-tetrahydroisoquinolin-5-yl)amino)ethyl)-4-(pyridin-2-yl)benzamide (8j).** Prepared according to General Procedure D using 2-amino-*N*-(5,6,7,8-tetrahydroisoquinolin-5-yl)acetamide trifluoroacetic acid (39.5 mg, 0.124 mmol, 1.0 eq.) and 4-pyridin-2-yl-benzoic acid (30.4 mg, 0.153 mmol, 1.2 eq.) to afford the title compound as a clear residue (10.9 mg, 28.2 µmol, 23% yield).
^{1}H NMR (400 MHz, DMSO-*d_6*) δ 8.88 (t, *J* = 5.8 Hz, 1H), 8.70 (ddd, *J* = 4.7, 1.6, 0.8 Hz, 1H), 8.40 (d, *J* = 8.7 Hz, 1H), 8.34 – 8.29 (m, 2H), 8.23 – 8.17 (m, 2H), 8.08 – 8.00 (m, 3H), 7.92 (td, *J* = 7.7, 1.8 Hz, 1H), 7.40 (ddd, *J* = 7.5, 4.8, 0.9 Hz, 1H), 7.19 (d, *J* = 5.1 Hz, 1H), 5.03 – 4.94 (m, 1H), 3.96 (d, *J* = 5.8 Hz, 2H), 2.76 – 2.69 (m, 2H), 1.98 – 1.88 (m, 2H), 1.82 – 1.62 (m, 2H).
^{13}C NMR (101 MHz, DMSO-*d_6*) δ 168.9, 166.2, 155.0, 149.9, 149.7, 146.8, 146.4, 141.2, 137.4, 134.3, 132.7, 127.9, 126.3, 123.2, 122.1, 120.7, 46.1, 42.9, 29.1, 25.5, 20.3.
HRMS (ESI-TOF) calculated for $C_{23}H_{23}N_4O_2$ $[M+H]^+$ = 387.1816, found = 387.1815.

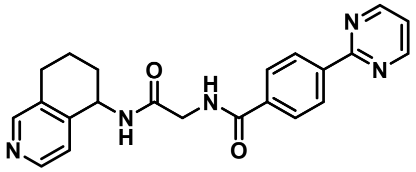

***N*-(2-oxo-2-((5,6,7,8-tetrahydroisoquinolin-5-yl)amino)ethyl)-4-(pyrimidin-2-yl)benzamide (8k).** Prepared according to General Procedure D using 2-amino-N-(5,6,7,8-tetrahydroisoquinolin-5-yl)acetamide hydrogen chloride (8 mg, 33.1 µmol, 1.0 eq.) 4-pyrimidin-2-yl-benzoic acid (9.4 mg, 46.8 µmol, 1.4 eq.) to afford the title compound as an off-white solid (8.5 mg, 21.9 µmol, 66% yield)
^{1}H NMR (400 MHz, DMSO-*d_6*) δ 8.95 (d, *J* = 4.9 Hz, 2H), 8.93 (t, *J* = 5.9 Hz, 1H), 8.51 – 8.46 (m, 2H), 8.42 (d, *J* = 8.7 Hz, 1H), 8.34 – 8.30 (m, 2H), 8.08 – 8.03 (m, 2H), 7.50 (t, *J* = 4.9 Hz, 1H), 7.21 – 7.18 (m, 1H), 5.03 – 4.94 (m, 1H), 3.96 (d, *J* = 5.8 Hz, 2H), 2.77 – 2.69 (m, 2H), 1.98 – 1.88 (m, 2H), 1.82 – 1.63 (m, 2H).
^{13}C NMR (101 MHz, DMSO-*d_6*) δ 168.8, 166.2, 162.6, 157.9, 149.9, 146.7, 146.5, 139.7, 136.0, 132.7, 127.9, 127.5, 122.1, 120.4, 46.2, 42.9, 29.1, 25.5, 20.3.
HRMS (ESI-TOF) calculated for $C_{22}H_{22}N_5O_2$ $[M+H]^+$ = 388.1768, found = 388.1765.

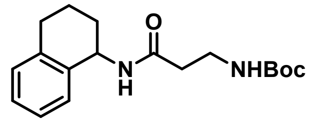

***Tert*-butyl (3-oxo-3-((1,2,3,4-tetrahydronaphthalen-1-yl)amino)propyl)carbamate.** Prepared according to General Procedure B using boc-β-alanine (325.4 mg, 1.72 mmol, 1.0 eq.) to afford the title compound as a white solid (283 mg, 0.889 mmol, 52%).
^{1}H NMR (400 MHz, $CDCl_3$) δ 7.20 – 7.15 (m, 1H), 7.14 – 7.07 (m, 2H), 7.06 – 7.00 (m, 1H), 6.37 (d, *J* = 7.9 Hz, 1H), 5.29 (s, 1H), 5.14 – 5.06 (m, 1H), 3.32 (t, *J* = 6.1 Hz, 2H), 2.81 – 2.64 (m, 2H), 2.41 – 2.28 (m, 2H), 2.02 – 1.91 (m, 1H), 1.87 – 1.68 (m, 3H), 1.38 (s, 9H).
^{13}C NMR (101 MHz, $CDCl_3$) δ 170.7, 156.1, 137.5, 136.6, 129.1, 128.5, 127.2, 126.2, 79.2, 47.4, 36.9, 36.3, 30.2, 29.2, 28.4, 20.0.
HRMS (ESI-TOF) calculated for $C_{18}H_{27}N_2O_3$ $[M+H]^+$ = 319.2016, found = 319.2023.

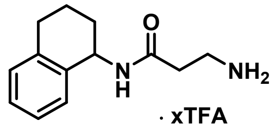

3-Amino-*N*-(1,2,3,4-tetrahydronaphthalen-1-yl)propenamide trifluoroacetic acid: Prepared according to General Procedure C using *tert*-butyl (3-oxo-3-((1,2,3,4-tetrahydronaphthalen-1-yl)amino)propyl)carbamate (283 mg, 0.889 mmol, 1.0 eq.) to afford the title compound as a clear solid in quantitative yield. Product taken to the next step without purification.
^{1}H NMR (400 MHz, DMSO-*d$_6$*) δ 8.47 (d, *J* = 8.6 Hz, 1H), 7.79 (s, 3H), 7.21 – 7.07 (m, 4H), 5.03 – 4.95 (m, 1H), 3.10 - 3.00 (m, 2H), 2.81 – 2.64 (m, 2H), 2.58 – 2.42 (m, 2H, overlapping with DMSO signal), 1.94 – 1.79 (m, 2H), 1.79– 1.62 (m, 2H).
^{13}C NMR (101 MHz, DMSO-*d6*) δ 168.7, 158.2 (q, $J_{C\text{-}F}$ = 35.5Hz), 137.1, 128.8, 128.3, 126.9, 125.9, 115.9 (q, $J_{C\text{-}F}$ = 292.9 Hz), 46.4, 35.4, 32.0, 29.9, 28.7, 19.9. Note: One aromatic C not observed.
HRMS (ESI-TOF) calculated for $C_{13}H_{19}N_2O$ [M+H]$^+$ = 219.1492, found = 219.1491.
Note: The analogous hydrogen chloride salt was prepared in a similar manner using 4M HCl in 1,4-dioxane to afford the desired compound in quantitative yield as a white solid.

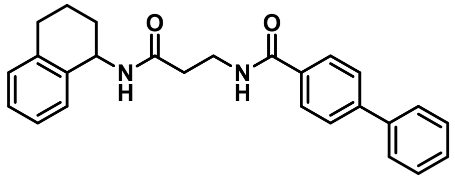

***N*-(3-oxo-3-((1,2,3,4-tetrahydronaphthalen-1-yl)amino)propyl)-[1,1'-biphenyl]-4-carboxamide (9a):** Prepared according to General Procedure D using 4-phenylbenzoic acid (25.5 mg, 0.129 mmol, 1.3 eq.) and 3-amino-*N*-(1,2,3,4-tetrahydronaphthalen-1-yl)propenamide trifluoroacetic acid (33.9 mg, 0.102 mmol, 1.0 eq.) to afford the title compound as a white solid (10.8 mg, 27.1 µmol, 27% yield).
^{1}H NMR (400 MHz, DMSO-*d$_6$*) δ 8.61 (t, *J* = 5.5 Hz, 1H), 8.30 (d, *J* = 8.7 Hz, 1H), 7.97 – 7.90 (m, 2H), 7.80 – 7.70 (m, 4H), 7.53 – 7.46 (m, 2H), 7.44 – 7.38 (m, 1H), 7.18 – 6.99 (m, 4H), 5.04 – 4.96 (m, 1H), 3.58 – 3.50 (m, 2H), 2.79 – 2.62 (m, 2H), 2.54 – 2.44 (m, 1H, partially buried under DMSO signal), 2.40 (dt, *J* = 14.2, 6.7 Hz, 1H), 1.92 – 1.79 (m, 2H), 1.75 – 1.60 (m, 2H).
^{13}C NMR (101 MHz, DMSO-*d$_6$*) δ 169.8, 165.8, 142.6, 139.2, 137.6, 137.0, 133.3, 129.0, 128.6, 128.2, 128.0, 127.9, 126.9, 126.6, 126.4, 125.8, 46.2, 36.3, 35.5, 29.9, 28.8, 20.0.
HRMS (ESI-TOF) calculated for $C_{26}H_{27}N_2O_2$ [M+H]$^+$ = 399.2067, found = 399.2072.

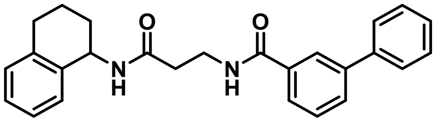

***N*-(3-oxo-3-((1,2,3,4-tetrahydronaphthalen-1-yl)amino)propyl)-[1,1'-biphenyl]-3-carboxamide (9b).** Prepared according to General Procedure D using 3-amino-*N*-(1,2,3,4-tetrahydronaphthalen-1-yl)propenamide hydrogen chloride (10 mg, 39.3 µmol, 1.0 eq.) and biphenyl-3-carboxylic acid (10.8 mg, 54.7 µmol, 1.4 eq.) to afford the title compound as a white solid (7.9 mg, 19.8 µmol, 50% yield).
^{1}H NMR (400 MHz, DMSO-*d$_6$*) δ 8.72 (t, *J* = 5.5 Hz, 1H), 8.30 (d, *J* = 8.7 Hz, 1H), 8.15 (t, *J* = 1.6 Hz, 1H), 7.86 – 7.80 (m, 2H), 7.76 – 7.71 (m, 2H), 7.56 (t, *J* = 7.7 Hz, 1H), 7.53 – 7.47 (m, 2H), 7.43 – 7.38 (m, 1H), 7.13 (d, *J* = 7.7 Hz, 1H), 7.08 – 7.01 (m, 2H), 6.92 – 6.86 (m, 1H), 5.04 – 4.95 (m, 1H), 3.60 – 3.51 (m, 2H), 2.76 – 2.61 (m, 2H), 2.55 – 2.34 (m, 2H), 1.92 – 1.76 (m, 2H), 1.74 – 1.58 (m, 2H).
^{13}C NMR (101 MHz, DMSO-*d$_6$*) δ 169.8, 166.0, 140.2, 139.6, 137.6, 137.0, 135.1, 129.3, 129.0, 129.0, 128.6, 128.2, 127.8, 126.9, 126.6, 126.4, 125.7, 125.4, 46.2, 36.4, 35.5, 29.9, 28.8, 20.0.
HRMS (ESI-TOF) calculated for $C_{26}H_{27}N_2O_2$ [M+H]$^+$ = 399.2067, found = 399.2066.

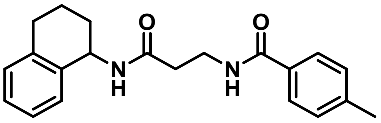

4-Methyl-*N*-(3-oxo-3-((1,2,3,4-tetrahydronaphthalen-1-yl)amino)propyl)benzamide (9c). Prepared according to General Procedure D using 3-amino-*N*-(1,2,3,4-tetrahydronaphthalen-1-yl)propenamide hydrogen chloride (10 mg, 39.3 μmol, 1.0 eq.) and 4-methylbenzoic acid (7.5 mg, 54.7 μmol, 1.4 eq.) to afford the title compound as a white solid (6.2 mg, 18.4 μmol, 47% yield).
^{1}H NMR (400 MHz, DMSO-*d*$_6$) δ 8.45 (t, *J* = 5.6 Hz, 1H), 8.28 (d, *J* = 8.7 Hz, 1H), 7.77 – 7.71 (m, 2H), 7.29 – 7.23 (m, 2H), 7.16 – 6.97 (m, 4H), 5.03 – 4.94 (m, 1H), 3.55 – 3.45 (m, 2H), 2.78 – 2.63 (m, 2H), 2.50 – 2.32 (m, 2H), 2.35 (s, 3H), 1.92 – 1.77 (m, 2H), 1.75 – 1.58 (m, 2H).
^{13}C NMR (101 MHz, DMSO-*d*$_6$) δ 169.9, 166.0, 140.9, 137.6, 137.0, 131.7, 128.8, 128.6, 128.2, 127.2, 126.6, 125.7, 46.2, 36.2, 35.5, 29.9, 28.8, 21.0, 20.0.
HRMS (ESI-TOF) calculated for $C_{21}H_{25}N_2O_2$ $[M+H]^+$ = 337.1911, found = 337.1910.

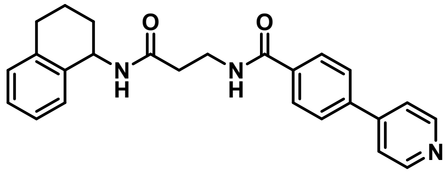

***N*-(3-oxo-3-((1,2,3,4-tetrahydronaphthalen-1-yl)amino)propyl)-4-(pyridin-4-yl)benzamide (VU6071680, 9d).** Prepared according to General Procedure D using 3-amino-*N*-(1,2,3,4-tetrahydronaphthalen-1-yl)propenamide hydrogen chloride (10 mg, 39.3 μmol, 1.0 eq.) and 4-pyridin-4-yl-benzoic acid (10.9 mg, 54.7 μmol, 1.4 eq.) to afford the title compound as a white solid (9.7 mg, 24.3 μmol, 62% yield).
^{1}H NMR (400 MHz, DMSO-*d*$_6$) δ 8.72 – 8.64 (m, 3H), 8.30 (d, *J* = 8.7 Hz, 1H), 8.02 – 7.96 (m, 2H), 7.94 – 7.89 (m, 2H), 7.80 – 7.75 (m, 2H), 7.17 – 6.99 (m, 4H), 5.04 – 4.96 (m, 1H), 3.59 – 3.51 (m, 2H), 2.78 – 2.62 (m, 2H), 2.55 – 2.35 (m, 2H), 1.93 – 1.78 (m, 2H), 1.75 – 1.59 (m, 2H).
^{13}C NMR (101 MHz, DMSO-*d*$_6$) δ 169.8, 165.6, 150.4, 146.0, 139.5, 137.6, 137.0, 134.9, 128.6, 128.2, 128.0, 126.7, 126.6, 125.8, 121.3, 46.2, 36.4, 35.4, 29.9, 28.8, 20.0.
HRMS (ESI-TOF) calculated for $C_{25}H_{26}N_3O_2$ $[M+H]^+$ = 400.2020, found = 400.2022.

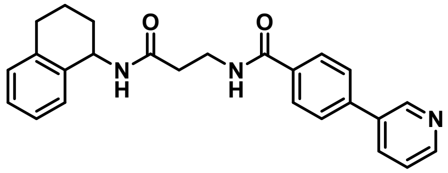

***N*-(3-oxo-3-((1,2,3,4-tetrahydronaphthalen-1-yl)amino)propyl)-4-(pyridin-3-yl)benzamide (9e).** Prepared according to General Procedure D using 3-amino-*N*-(1,2,3,4-tetrahydronaphthalen-1-yl)propenamide hydrogen chloride (10 mg, 39.3 μmol, 1.0 eq.) and 4-pyridin-3-yl-benzoic acid (9.1 mg, 45.6 μmol, 1.2 eq.) to afford the title compound as a white solid (9.1 mg, 22.8 μmol, 58% yield).
^{1}H NMR (400 MHz, DMSO-*d*$_6$) δ 8.96 (dd, *J* = 2.4, 0.5 Hz, 1H), 8.65 (t, *J* = 5.5 Hz, 1H), 8.61 (dd, *J* = 4.7, 1.5 Hz, 1H), 8.30 (d, *J* = 8.7 Hz, 1H), 8.15 (ddd, *J* = 8.0, 2.3, 1.7 Hz, 1H), 8.00 – 7.95 (m, 2H), 7.88 – 7.82 (m, 2H), 7.52 (ddd, *J* = 7.9, 4.8, 0.6 Hz, 1H), 7.18 – 7.00 (m, 4H), 5.04 – 4.96 (m, 1H), 3.60 – 3.50 (m, 2H), 2.78 – 2.62 (m, 2H), 2.55 – 2.35 (m, 2H), 1.93 – 1.78 (m, 2H), 1.75 – 1.59 (m, 2H).
^{13}C NMR (101 MHz, DMSO-*d*$_6$) δ 169.8, 165.7, 149.0, 147.8, 139.6, 137.6, 137.0, 134.7, 134.3, 134.0, 128.7, 128.2, 128.0, 126.7, 126.6, 125.8, 124.0, 46.2, 36.4, 35.5, 29.9, 28.8, 20.0.
HRMS (ESI-TOF) calculated for $C_{25}H_{26}N_3O_2$ $[M+H]^+$ = 400.2020, found = 400.2020.

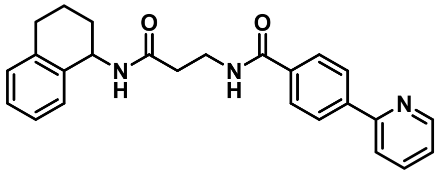

***N*-(3-oxo-3-((1,2,3,4-tetrahydronaphthalen-1-yl)amino)propyl)-4-(pyridin-2-yl)benzamide (9f).** Prepared according to General Procedure D using 3-amino-*N*-(1,2,3,4-tetrahydronaphthalen-1-yl)propenamide hydrogen chloride (10 mg, 39.3 μmol, 1.0 eq.) and 4-(pyridine-2-yl)benzoic acid (10.9 mg, 54.7 μmol, 1.4 eq.) to afford the title compound as a white solid (5.9 mg, 14.8 μmol, 38% yield).
^{1}H NMR (400 MHz, DMSO-*d$_6$*) δ 8.70 (ddd, *J* = 4.7, 1.7, 0.8 Hz, 1H), 8.65 (t, *J* = 5.5 Hz, 1H), 8.30 (d, *J* = 8.7 Hz, 1H), 8.21 – 8.15 (m, 2H), 8.08 - 8.03 (m, 1H), 8.00 – 7.94 (m, 2H), 7.92 (td, *J* = 7.8, 1.8 Hz, 1H), 7.40 (ddd, *J* = 7.5, 4.8, 0.8 Hz, 1H), 7.18 – 7.13 (m, 1H), 7.13 – 6.99 (m, 3H), 5.04 – 4.96 (m, 1H), 3.58 – 3.51 (m, 2H), 2.79 – 2.62 (m, 2H), 2.53 – 2.36 (m, 2H), 1.93 – 1.78 (m, 2H), 1.76 – 1.60 (m, 2H).
^{13}C NMR (101 MHz, DMSO-*d$_6$*) δ 169.8, 165.8, 155.1, 149.7, 141.0, 137.6, 137.4, 137.0, 134.7, 128.7, 128.2, 127.7, 126.6, 126.3, 125.8, 123.2, 120.7, 46.2, 36.4, 35.5, 29.9, 28.8, 20.0.
HRMS (ESI-TOF) calculated for $C_{25}H_{26}N_3O_2$ $[M+H]^+$ = 400.2020, found = 400.2018.

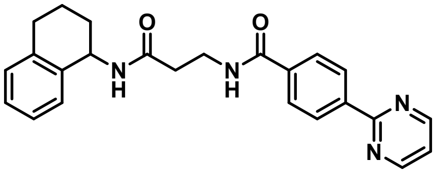

***N*-(3-oxo-3-((1,2,3,4-tetrahydronaphthalen-1-yl)amino)propyl)-4-(pyrimidin-2-yl)benzamide (9g).** Prepared according to General Procedure D using 3-amino-*N*-(1,2,3,4-tetrahydronaphthalen-1-yl)propenamide hydrogen chloride (10 mg, 39.3 μmol, 1.0 eq.) and 4-pyrimidin-2-yl-benzoic acid (11 mg, 54.7 μmol, 1.4 eq.) to afford the title compound as a white solid (7.3 mg, 18.2 μmol, 46% yield).
^{1}H NMR (400 MHz, DMSO-*d$_6$*) δ 8.95 (d, *J* = 4.9 Hz, 2H), 8.69 (t, *J* = 5.6 Hz, 1H), 8.48 – 8.43 (m, 2H), 8.31 (d, *J* = 8.7 Hz, 1H), 8.02 – 7.97 (m, 2H), 7.50 (t, *J* = 4.9 Hz, 1H), 7.16 (d, *J* = 7.6 Hz, 1H), 7.13 – 6.97 (m, 3H), 5.04 – 4.96 (m, 1H), 3.60 – 3.50 (m, 2H), 2.79 – 2.62 (m, 2H), 2.55 – 2.36 (m, 2H), 1.93 – 1.78 (m, 2H), 1.76 – 1.59 (m, 2H).
^{13}C NMR (101 MHz, DMSO-*d$_6$*) δ 169.8, 165.7, 162.6, 157.9, 139.5, 137.6, 137.0, 136.4, 128.7, 128.3, 127.7, 127.5, 126.6, 125.7, 120.4, 46.2, 36.4, 35.5, 29.9, 28.8, 20.0.
HRMS (ESI-TOF) calculated for $C_{24}H_{25}N_4O_2$ $[M+H]^+$ = 401.1972, found = 401.1980.

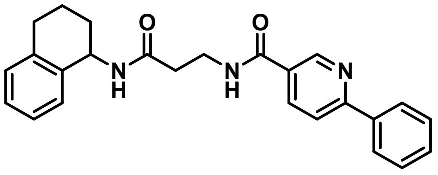

***N*-(3-oxo-3-((1,2,3,4-tetrahydronaphthalen-1-yl)amino)propyl)-6-phenylnicotinamide (9h).** Prepared according to General Procedure D using 3-amino-*N*-(1,2,3,4-tetrahydronaphthalen-1-yl)propenamide hydrogen chloride (10 mg, 39.3 μmol, 1.0 eq.) and 6-phenylnicotinic acid (10.9 mg, 54.7 μmol, 1.4 eq.) to afford the title compound as a white solid (6.1 mg, 15.3 μmol, 39% yield).
^{1}H NMR (400 MHz, DMSO-*d$_6$*) δ 9.09 (dd, *J* = 2.3, 0.5 Hz, 1H), 8.81 (t, *J* = 5.6 Hz, 1H), 8.31 (d, *J* = 8.7 Hz, 1H), 8.28 (dd, *J* = 8.4, 2.3 Hz, 1H), 8.18 – 8.13 (m, 2H), 8.09 (dd, *J* = 8.3, 0.5 Hz, 1H), 7.56 – 7.45 (m, 3H), 7.15 (d, *J* = 7.7 Hz, 1H), 7.12 – 6.98 (m, 3H), 5.04 – 4.96 (m, 1H), 3.60 – 3.52 (m, 2H), 2.78 – 2.62 (m, 2H), 2.55 – 2.36 (m, 2H), 1.93 – 1.77 (m, 2H), 1.75 – 1.59 (m, 2H).
^{13}C NMR (101 MHz, DMSO-*d$_6$*) δ 169.7, 164.5, 158.0, 148.6, 137.8, 137.6, 137.0, 136.0, 129.7, 128.9, 128.7, 128.3, 128.2, 126.9, 126.6, 125.7, 119.6, 46.2, 36.3, 35.4, 29.9, 28.8, 20.20.
HRMS (ESI-TOF) calculated for $C_{25}H_{26}N_3O_2$ $[M+H]^+$ = 400.2020, found = 400.2018.

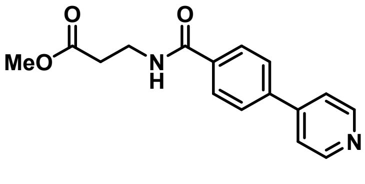

Methyl 3-(4-(pyridin-4-yl)benzamido)propanoate. To a vial was added 4-pyridin-4-yl-benzoic acid (202.8 mg, 1.02 mmol, 1.0 eq.) and anhydrous DCM (5 mL). Oxalyl chloride (700 µL, 8.02 mmol, 7.9 eq.) was added, followed by 2 drops of anhydrous DMF. The mixture was stirred at room temperature for 2.5 hours, then was concentrated to afford 4-(pyridin-4-yl)benzoyl chloride as a light yellow solid in quantitative yield. To a separate vial was added β-alanine methyl ester hydrochloride (141.2 mg, 1.01 mmol, 1.0 eq.), anhydrous DCM (5 mL), and triethylamine (700 µL, 5.02 mmol, 4.9 eq.). The mixture was stirred and a solution of 4-(pyridin-4-yl)benzoyl chloride in anhydrous DCM (3 mL) was added slowly. The mixture was stirred at room temperature overnight, then was quenched with H_2O, extracted with 3 portions of DCM, and concentrated. The crude material was purified via automated flash column chromatography (Teledyne ISCO, 24 g column, 0-10% MeOH in DCM) to afford the title compound as a white solid (223.7 mg, 0.787 mmol, 77% yield).
^{1}H NMR (400 MHz, $CDCl_3$) δ 8.71 – 8.67 (m, 2H), 7.91 – 7.86 (m, 2H), 7.72 – 7.67 (m, 2H), 7.53 – 7.50 (m, 2H), 6.94 (t, *J* = 5.5 Hz, 1H), 3.79 - 3.73 (m, 2H), 3.73 (s, 3H), 2.69 (t, *J* = 5.8 Hz, 2H).
^{13}C NMR (101 MHz, $CDCl_3$) δ 173.6, 166.7, 150.6, 147.4, 141.3, 134.9, 127.9, 127.4, 121.8, 52.1, 35.5, 33.8.
HRMS (ESI-TOF) calculated for $C_{16}H_{17}N_2O_3$ $[M+H]^+$ = 285.1234, found = 285.1236.

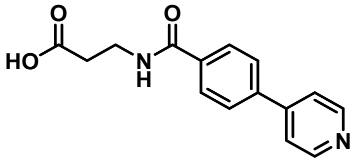

General Procedure E: Preparation of 3-(4-(Pyridin-4-yl)benzamido)propanoic acid hydrogen chloride. To a vial was added methyl 3-(4-(pyridin-4-yl)benzamido)propanoate (217.3 mg, 0.764 mmol, 1 eq.) and THF (5 mL), followed by 1M LiOH (1.55 mL, 1.55 mmol, 2.0 eq.). The mixture was heated to 50 °C for 1.75 hours, then was cooled to room temperature, acidified to pH ~3 with 2N HCl, and concentrated to afford the title compound as a pale-yellow solid in quantitative yield. Material taken directly to the next step without purification.
^{1}H NMR (400 MHz, DMSO-*d_6*): δ 8.99 – 8.93 (m, 2H), 8.87 (t, *J* = 5.4 Hz, 1H), 8.41 – 8.35 (m, 2H), 8.14 – 8.04 (m, 4H), 3.53 – 3.44 (m, 2H), 2.55 (t, *J* = 7.1 Hz, 2H).
^{13}C NMR (101 MHz, DMSO-*d_6*): δ 172.9, 165.3, 153.5, 143.1, 137.0, 136.5, 128.3, 127.9, 123.9, 35.7, 33.7.
HRMS (ESI-TOF) calculated for $C_{15}H_{15}N_2O_3$ $[M+H]^+$ = 271.1077, found = 271.1077.

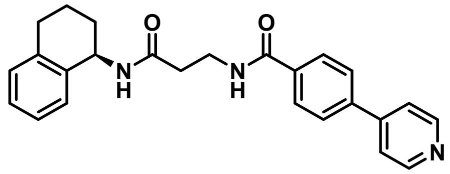

(*R*)-*N*-(3-oxo-3-((1,2,3,4-tetrahydronaphthalen-1-yl)amino)propyl)-4-(pyridin-4-yl)benzamide ((*R*)-9d): Prepared in a similar manner as General Procedure A using 3-(4-(pyridin-4-yl)benzamido)propanoic acid hydrogen chloride (34.4 mg, 0.115 mmol, 1.1 eq.) and (*R*)-(-)-1,2,3,4-tetrahydronaphthalen-1-amine (15.4 µL, 0.109 mmol, 1.0 eq.) to afford the title compound as a white solid (24.5 mg, 61.3 µmol, 56% yield).
^{1}H NMR (400 MHz, DMSO-*d_6*) δ 8.73 – 8.62 (m, 3H), 8.30 (d, *J* = 8.6 Hz, 1H), 8.03 – 7.95 (m, 2H), 7.95 – 7.88 (m, 2H), 7.81 – 7.75 (m, 2H), 7.18 – 6.99 (m, 4H), 5.05 – 4.95 (m, 1H), 3.59 - 3.50 (m, 2H), 2.79 – 2.62 (m, 2H), 2.54 – 2.45 (m, 1H, partially buried under DMSO signal), 2.40 (dt, *J* = 14.4, 6.7 Hz, 1H), 1.93 – 1.78 (m, 2H), 1.76 – 1.59 (m, 2H).
^{13}C NMR (101 MHz, DMSO-*d_6*) δ 169.8, 165.5, 150.3, 146.0, 139.5, 137.6, 137.0, 134.9, 128.6, 128.2, 128.0, 126.7, 126.6, 125.8, 121.3, 46.2, 36.4, 35.4, 29.9, 28.8, 20.0.
HRMS (ESI-TOF) calculated for $C_{25}H_{26}N_3O_2$ $[M+H]^+$ = 400.2020, found = 400.2025.

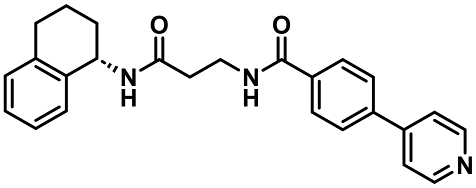

(*S*)-*N*-(3-oxo-3-((1,2,3,4-tetrahydronaphthalen-1-yl)amino)propyl)-4-(pyridin-4-yl)benzamide ((*S*)-9d): Prepared in a similar manner as General Procedure A using 3-(4-(pyridin-4-yl)benzamido)propanoic acid hydrogen chloride (34.4 mg, 0.114 mmol, 1.1 eq.) and (*S*)-(+)-1,2,3,4-tetrahydronaphthalen-1-amine (15.9 µL, 0.109 mmol, 1.0 eq.) to afford the title compound as a white solid (26.1 mg, 65.3 µmol, 60% yield).
^{1}H NMR (400 MHz, DMSO-*d₆*) δ 8.71 – 8.64 (m, 3H), 8.30 (d, *J* = 8.7 Hz, 1H), 8.01 – 7.96 (m, 2H), 7.94 – 7.89 (m, 2H), 7.80 – 7.76 (m, 2H), 7.17 – 6.99 (m, 4H), 5.04 – 4.96 (m, 1H), 3.59 - 3.50 (m, 2H), 2.79 – 2.62 (m, 2H), 2.54 – 2.45 (m, 1H, partially buried under DMSO signal), 2.40 (dt, *J* = 14.3, 6.8 Hz, 1H), 1.92 – 1.78 (m, 2H), 1.75 – 1.60 (m, 2H).
^{13}C NMR (101 MHz, DMSO-*d₆*) δ 169.8, 165.5, 150.3, 146.0, 139.5, 137.6, 137.0, 134.9, 128.6, 128.2, 128.0, 126.7, 126.6, 125.7, 121.3, 46.2, 36.4, 35.4, 29.9, 28.8, 20.0.
HRMS (ESI-TOF) calculated for $C_{25}H_{26}N_3O_2$ [M+H]$^+$ = 400.2020, found = 400.2024.

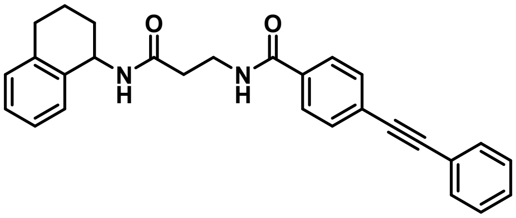

***N*-(3-oxo-3-((1,2,3,4-tetrahydronaphthalen-1-yl)amino)propyl)-4-(phenylethynyl)benzamide (9i):** Prepared according to General Procedure D using 3-amino-*N*-(1,2,3,4-tetrahydronaphthalen-1-yl)propenamide trifluoroacetic acid (28.5 mg, 85.6 µmol, 1.0 eq.) and 4-(phenylethynyl)benzoic acid (20.9 mg, 94.0 µmol, 1.1 eq.) to afford the title compound as a white solid (4.3 mg, 10.2 µmol, 12% yield).
^{1}H NMR (400 MHz, DMSO-*d₆*) δ 8.67 (t, *J* = 5.6 Hz, 1H), 8.30 (d, *J* = 8.7 Hz, 1H), 7.93 – 7.86 (m, 2H), 7.68 – 7.62 (m, 2H), 7.62 – 7.56 (m, 2H), 7.49 – 7.42 (m, 3H), 7.17 – 7.08 (m, 2H), 7.08 – 6.98 (m, 2H), 5.04 – 4.96 (m, 1H), 3.58 – 3.49 (m, 2H), 2.78 – 2.62 (m, 2H), 2.53 – 2.44 (m, 1H, partially buried under DMSO signal), 2.39 (dt, *J* = 14.3, 6.7 Hz, 1H), 1.92 – 1.77 (m, 2H), 1.75 – 1.59 (m, 2H).
^{13}C NMR (101 MHz, DMSO-*d₆*) δ 169.8, 165.4, 137.6, 137.0, 134.3, 131.5, 131.3, 129.2, 128.9, 128.7, 128.2, 127.6, 126.6, 125.8, 124.9, 122.0, 91.2, 88.8, 46.2, 36.4, 35.4, 29.9, 28.8, 20.0.
HRMS (ESI-TOF) calculated for $C_{28}H_{27}N_2O_2$ [M+H]$^+$ = 423.2067, found = 423.2067.

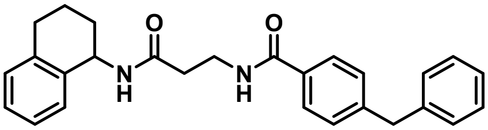

4-Benzyl-*N*-(3-oxo-3-((1,2,3,4-tetrahydronaphthalen-1-yl)amino)propyl)benzamide (9j): Prepared according to General Procedure D using 3-amino-*N*-(1,2,3,4-tetrahydronaphthalen-1-yl)propenamide trifluoroacetic acid (28.5 mg, 85.6 µmol, 1.0 eq.) and 4-benzylbenzoic acid (20.5 mg, 96.6 µmol, 1.1 eq.) to afford the title compound as a white solid (11.0 mg, 26.7 µmol, 31% yield).
^{1}H NMR (400 MHz, DMSO-*d₆*) δ 8.46 (t, *J* = 5.6 Hz, 1H), 8.26 (d, *J* = 8.7 Hz, 1H), 7.79 – 7.74 (m, 2H), 7.34 – 7.26 (m, 4H), 7.26 – 7.16 (m, 3H), 7.13 – 7.02 (m, 3H), 6.96 – 6.90 (m, 1H), 5.01 – 4.93 (m, 1H), 3.99 (s, 2H), 3.53 – 3.45 (m, 2H), 2.77 – 2.61 (m, 2H), 2.51 – 2.41 (m, 1H, partially buried under DMSO signal), 2.35 (dt, *J* = 14.3, 6.7 Hz, 1H), 1.92 – 1.76 (m, 2H), 1.74 – 1.56 (m, 2H).
^{13}C NMR (101 MHz, DMSO-*d₆*) δ 169.8, 166.0, 144.6, 140.9, 137.5, 137.0, 132.3, 128.7, 128.6, 128.54, 128.49, 128.2, 127.4, 126.6, 126.1, 125.7, 46.2, 40.8, 36.3, 35.5, 29.9, 28.8, 20.0.
HRMS (ESI-TOF) calculated for $C_{27}H_{29}N_2O_2$ [M+H]$^+$ = 413.2224, found = 413.2224.

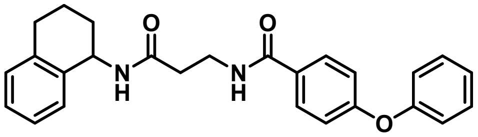

***N*-(3-oxo-3-((1,2,3,4-tetrahydronaphthalen-1-yl)amino)propyl)-4-phenoxybenzamide (9k):** Prepared according to General Procedure D using 3-amino-*N*-(1,2,3,4-tetrahydronaphthalen-1-yl)propenamide trifluoroacetic acid (28.5 mg, 85.6 μmol, 1.0 eq.) and 4-phenoxybenzoic acid (20.4 mg, 95.2 μmol, 1.1 eq.) to afford the title compound as a white solid (14.1 mg, 34.0 μmol, 40% yield).
^{1}H NMR (400 MHz, DMSO-*d₆*) δ 8.50 (t, *J* = 5.6 Hz, 1H), 8.28 (d, *J* = 8.7 Hz, 1H), 7.91 – 7.84 (m, 2H), 7.47 – 7.40 (m, 2H), 7.21 (tt, *J* = 7.4, 1.0 Hz, 1H), 7.15 – 6.97 (m, 8H), 5.02 – 4.95 (m, 1H), 3.55 – 3.47 (m, 2H), 2.78 – 2.63 (m, 2H), 2.50 – 2.42 (m, 1H), 2.37 (dt, *J* = 14.3, 6.7 Hz, 1H), 1.91 – 1.77 (m, 2H), 1.75 – 1.58 (m, 2H).
^{13}C NMR (101 MHz, DMSO-*d₆*) δ 169.8, 165.4, 159.3, 155.8, 137.6, 137.0, 130.3, 129.4, 129.3, 128.7, 128.2, 126.6, 125.7, 124.2, 119.4, 117.5, 46.2, 36.3, 35.5, 29.9, 28.8, 20.0.
HRMS (ESI-TOF) calculated for $C_{26}H_{27}N_2O_3$ $[M+H]^+$ = 415.2016, found = 415.2015.

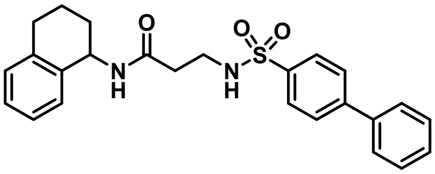

3-([1,1'-Biphenyl]-4-sulfonamido)-*N*-(1,2,3,4-tetrahydronaphthalen-1-yl)propenamide (9l). To a test tube was added a solution of 3-amino-N-(1,2,3,4-tetrahydronaphthalen-1-yl)propenamide trifluoroacetic acid (33.9 mg, 0.102 mmol, 1.0 eq.) in anhydrous DMF (1 mL), followed by TEA (44.4 μL, 0.319 mmol, 3.1 eq.). To the stirring solution was added biphenyl-4-sulfonyl chloride (31.1 mg, 0.123 mmol, 1.2 eq.). The reaction was stirred at room temperature for 3.5 hours, then was quenched with 150 μL H_2O and purified via RP-HPLC (5-72% MeCN in 0.5 mL/L AQ NH_4OH) to afford the title compound as a white solid (13.2 mg, 30.4 μmol, 30% yield).
^{1}H NMR (400 MHz, DMSO-*d₆*) δ 8.26 (d, *J* = 8.6 Hz, 1H), 7.94 – 7.84 (m, 4H), 7.78 – 7.73 (m, 2H), 7.71 (t, *J* = 5.1 Hz, 1H), 7.55 – 7.48 (m, 2H), 7.47 – 7.41 (m, 1H), 7.17 – 7.04 (m, 4H), 4.99 – 4.89 (m, 1H), 3.09 – 2.96 (m, 2H), 2.79 – 2.63 (m, 2H), 2.40 – 2.25 (m, 2H), 1.90 – 1.78 (m, 2H), 1.74 – 1.57 (m, 2H).
^{13}C NMR (101 MHz, DMSO-*d₆*) δ 169.0, 143.9, 139.1, 138.6, 137.4, 137.0, 129.1, 128.7, 128.5, 128.3, 127.4, 127.2, 127.1, 126.7, 125.8, 46.2, 35.6, 29.8, 28.8, 19.9. Note: 1 aliphatic carbon absent, likely buried under DMSO.
HRMS (ESI-TOF) calculated for $C_{25}H_{27}N_2O_3S$ $[M+H]^+$ = 435.1737, found = 435.1735.

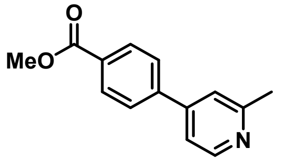

General Procedure F: Preparation of methyl 4-(2-methylpyridin-4-yl)benzoate. To a vial was added potassium trifluoro(4-(methoxycarbonyl)phenyl)borate (126.7 mg, 0.523 mmol, 1.0 eq.), Cs_2CO_3 (438.6 mg, 1.34 mmol, 2.6 eq.), and Pd(dppf)Cl_2 (41.6 mg, 56.7 μmol, 0.1 eq.), followed by 1,4-dioxane (2.2 mL), H_2O (0.4 mL), and 4-bromo-2-methylpyridine (91.7 μL, 0.773 mmol, 1.5 eq.). The mixture was degassed with N2 for 5 minutes, then was heated to 100 °C for 16 hours. The reaction was then cooled to room temperature, diluted with H_2O, extracted multiple times with DCM and 3:1 $CHCl_3$:IPA, and concentrated. The crude material was purified via automated flash column chromatography (Teledyne ISCO, 0-100% EtOAc in Hexanes) to afford the title compound as a white solid (68.0 mg, 0.299 mmol, 57% yield).
^{1}H NMR (400 MHz, $CDCl_3$) δ 8.58 (d, *J* = 5.2 Hz, 1H), 8.17 – 8.12 (m, 2H), 7.72 – 7.66 (m, 2H), 7.40 – 7.38 (m, 1H), 7.35 – 7.32 (m, 1H), 3.95 (s, 3H), 2.64 (s, 3H).
^{13}C NMR (101 MHz, $CDCl_3$) δ 166.8, 159.3, 149.9, 147.7, 143.0, 130.6, 130.5, 127.2, 121.4, 119.1, 52.4, 24.7.
HRMS (ESI-TOF) calculated for $C_{14}H_{14}NO_2$ $[M+H]^+$ = 228.1019, found = 228.1022.

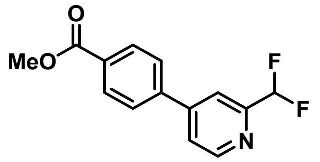

Methyl 4-(2-(difluoromethyl)pyridin-4-yl)benzoate. Prepared according to General Procedure F using potassium trifluoro(4-(methoxycarbonyl)phenyl)borate (128.6 mg, 0.531 mmol, 1.0 eq.) and 4-bromo-2-(difluoromethyl)pyridine (158.4 mg, 0.762 mmol, 1.4 eq.) to afford the title compound as a white solid (91.4 mg, 0.347 mmol, 65% yield).
^{1}H NMR (400 MHz, $CDCl_3$) δ 8.75 - 8.73 (m, 1H), 8.21 – 8.16 (m, 2H), 7.89 – 7.86 (m, 1H), 7.76 – 7.72 (m, 2H), 7.66 - 7.63 (m, 1H), 6.71 (t, *J* = 55.4 Hz, 1H), 3.97 (s, 3H).
^{13}C NMR (101 MHz, CDCl3) δ 166.6, 153.8 (t, $J_{C\text{-}F}$ = 25.6 Hz), 150.3, 149.0, 141.8, 131.2, 130.7, 127.3, 123.6, 118.3 (t, $J_{C\text{-}F}$ = 3.0 Hz), 114.1 (t, $J_{C\text{-}F}$ = 240.8 Hz), 52.5.
HRMS (ESI-TOF) calculated for $C_{14}H_{12}F_2NO_2$ $[M+H]^+$ = 264.0831, found = 264.0832.

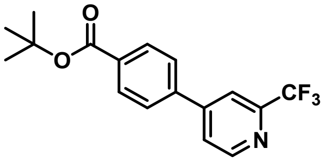

***Tert*-butyl 4-(2-(trifluoromethyl)pyridin-4-yl)benzoate.** To a vial was added 4-chloro-(2-trifluoromethyl)pyridine (176.4 µL, 1.38 mmol, 1.0 eq.), (4-(*tert*-butoxycarbonyl)phenyl)boronic acid (461.9 mg, 2.08 mmol, 1.5 eq.), Cs_2CO_3 (1121.9 mg, 3.42 mmol, 2.5 eq.), and Pd(dppf)Cl_2 (103.6 mg, 0.141 mmol, 0.1 eq.), followed by 1,4-dioxane (5.9 mL) and H_2O (1 mL). The mixture was degassed with N_2 for 5 minutes, then was heated to 100 °C for 19 hours. The mixture was cooled to room temperature, filtered over celite, diluted with H_2O, extracted 3x with DCM, and concentrated. The crude material was purified via automated flash column chromatography (Teledyne ISCO, 24g column, 0-40% EtOAc in hexanes) to afford the title compound as a white solid (397.1 mg, 1.23 mmol, 89% yield).
^{1}H NMR (400 MHz, $CDCl_3$) δ 8.81 (d, *J* = 5.1 Hz, 1H), 8.16 – 8.10 (m, 2H), 7.91 – 7.89 (m, 1H), 7.73 – 7.67 (m, 3H), 1.62 (s, 9H).
^{13}C NMR (101 MHz, $CDCl_3$) δ 165.1, 150.8, 149.4, 149.2 (q, $J_{C\text{-}F}$ = 34.5 Hz), 140.7, 133.4, 130.6, 127.1, 124.4, 121.7 (q, $J_{C\text{-}F}$ = 274 Hz), 118.7 (q, $J_{C\text{-}F}$ = 2.9 Hz) 81.8, 28.3.
HRMS (ESI-TOF) calculated for $C_{17}H_{17}F_3NO_2$ $[M+H]^+$ = 324.1206, found = 324.1208.

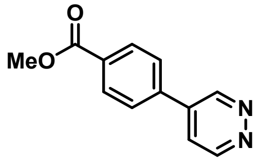

Methyl 4-(pyridazin-4-yl)benzoate. Prepared according to General Procedure F using potassium trifluoro(4-(methoxycarbonyl)phenyl)borate (126.9 mg, 0.524 mmol, 1.0 eq.) and 3-bromopyridazine hydrobromide (126.2 mg, 0.526 mmol, 1.0 eq.) to afford the title compound as a tan solid (44.8 mg, 0.209 mmol, 40% yield).
^{1}H NMR (400 MHz, $CDCl_3$) δ 9.50 (dd, *J* = 2.4, 1.2 Hz, 1H), 9.29 (dd, *J* = 5.4, 1.1 Hz, 1H), 8.24 – 8.19 (m, 2H), 7.78 – 7.73 (m, 2H), 7.69 (dd, *J* = 5.4, 2.5 Hz, 1H), 3.97 (s, 3H).
^{13}C NMR (101 MHz, $CDCl_3$) δ 166.4, 151.7, 149.9, 139.0, 137.6, 131.8, 130.9, 127.4, 123.6, 52.6.
HRMS (ESI-TOF) calculated for $C_{12}H_{11}N_2O_2$ $[M+H]^+$ = 215.0815, found = 215.0815.

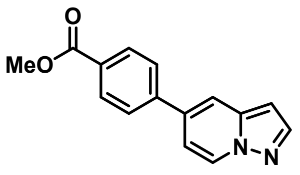

Methyl 4-(pyrazolo[1,5-*a*]pyridin-5-yl)benzoate: Prepared according to General Procedure F using potassium trifluoro(4-(methoxycarbonyl)phenyl)borate (80.9 mg, 0.334 mmol, 1.0 eq.) and 5-bromopyrazolo[1,5-*a*]pyridine (99.1 mg, 0.503 mmol, 1.5 eq.) to afford the title compound as a yellow solid (36.4 mg, 0.144 mmol, 43% yield).
^{1}H NMR (400 MHz, CDCl$_3$) δ 8.56 – 8.51 (m, 1H), 8.16 – 8.10 (m, 2H), 7.99 (d, *J* = 2.3 Hz, 1H), 7.79 – 7.76 (m, 1H), 7.73 – 7.67 (m, 2H), 7.03 (dd, *J* = 7.3, 2.0 Hz, 1H), 6.61 – 6.58 (m, 1H), 3.95 (s, 3H).
^{13}C NMR (101 MHz, CDCl$_3$) δ 166.8, 143.3, 142.8, 140.2, 135.2, 130.4, 129.8, 128.9, 126.9, 115.9, 111.3, 98.0, 52.4.
HRMS (ESI-TOF) calculated for $C_{15}H_{13}N_2O_2$ $[M+H]^+$ = 253.0972, found = 253.0974.

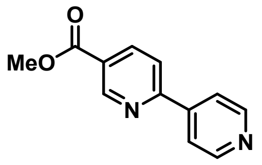

Methyl [2,4'-bipyridine]-5-carboxylate (76). Prepared according to General Procedure F using methyl 6-bromonicotinate (200 mg, 0.926 mmol, 1.0 eq.) and 4-pyridineboronic acid pinacol ester (189.8 mg, 0.926 mmol, 1.0 eq.) to afford the title compound as a tan solid (96.3 mg, 0.450 mmol, 49% yield).
^{1}H NMR (400 MHz, CDCl$_3$) δ 9.29 (dd, *J* = 2.1, 0.9 Hz, 1H), 8.75 – 8.72 (m, 2H), 8.38 (dd, *J* = 8.2, 2.2 Hz, 1H), 7.93 – 7.90 (m, 2H), 7.86 (dd, *J* = 8.3, 0.7 Hz, 1H), 3.96 (s, 3H).
^{13}C NMR (101 MHz, CDCl$_3$) δ 165.5, 158.2, 151.3, 150.7, 145.3, 138.3, 125.9, 121.4, 120.4, 52.6.
HRMS (ESI-TOF) calculated for $C_{12}H_{11}N_2O_2$ $[M+H]^+$ = 215.0815, found = 215.0819.

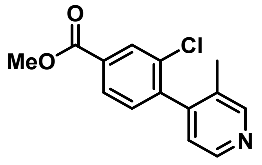

Methyl 3-chloro-4-(3-methylpyridin-4-yl)benzoate (77). Prepared according to General Procedure F using (2-chloro-4-(methoxycarbonyl)phenyl)boronic acid (57.0 mg, 0.266 mmol, 1.0 eq.) and 4-bromo-3-methylpyridine hydrochloride (59.7 mg, 0.286 mmol, 1.1 eq.) to afford the title compound as a clear oil (42.0 mg, 0.161 mmol, 60% yield).
^{1}H NMR (400 MHz, CDCl$_3$) δ 8.60 – 8.45 (m, 2H), 8.16 (d, *J* = 1.6 Hz, 1H), 8.00 (dd, *J* = 7.9, 1.6 Hz, 1H), 7.27 (d, *J* = 7.9 Hz, 1H), 7.07 (d, *J* = 4.6 Hz, 1H), 3.95 (s, 3H), 2.11 (s, 3H).
^{13}C NMR (101 MHz, CDCl$_3$) δ 165.7, 151.1, 147.4, 146.3, 142.4, 133.1, 131.7, 131.4, 131.0, 130.4, 128.1, 123.6, 52.7, 16.7.
HRMS (ESI-TOF) calculated for $C_{14}H_{13}ClNO_2$ $[M+H]^+$ = 262.0629, found = 262.0634.

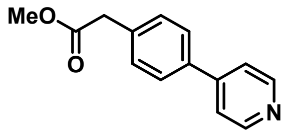

Methyl 2-(4-(pyridin-4-yl)phenyl)acetate. Prepared according to General Procedure F using methyl 2-(4-bromophenyl)acetate (57.1 mg, 0.249 mmol, 1.0 eq.) and 4-pyridineboronic acid pinacol ester (61.3 mg, 0.299 mmol, 1.2 eq.) to afford the title compound as a pale yellow solid (39.7 mg, 0.175 mmol, 70% yield).
^{1}H NMR (400 MHz, CDCl$_3$) δ 8.70 – 8.60 (m, 2H), 7.62 – 7.58 (m, 2H), 7.51 – 7.46 (m, 2H), 7.42 – 7.38 (m, 2H), 3.71 (s, 3H), 3.69 (s, 2H).

^{13}C NMR (101 MHz, CDCl$_3$) δ 171.8, 150.4, 148.0, 137.1, 135.1, 130.2, 127.3, 121.7, 52.3, 40.9.
HRMS (ESI-TOF) calculated for $C_{14}H_{14}NO_2$ $[M+H]^+$ = 228.1019, found = 228.1025.

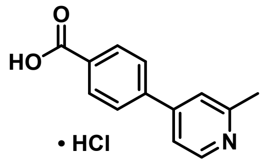

4-(2-Methylpyridin-4-yl)benzoic acid hydrochloride. Prepared in a similar manner as General Procedure E using methyl 4-(2-methylpyridin-4-yl)benzoate (66.8 mg, 0.294 mmol, 1.0 eq.) to afford the title compound as a light-yellow solid in quantitative yield. Product taken forward without purification.
^{1}H NMR (400 MHz, DMSO-*d$_6$*) δ 8.82 (d, *J* = 6.2 Hz, 1H), 8.31 – 8.28 (m, 1H), 8.20 – 8.15 (m, 1H), 8.15 – 8.08 (m, 4H), 2.77 (s, 3H).
HRMS (ESI-TOF) calculated for $C_{13}H_{12}NO_2$ $[M+H]^+$ = 214.0863, found = 214.0862.

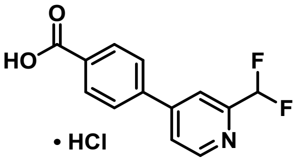

4-(2-(Difluoromethyl)pyridin-4-yl)benzoic acid hydrochloride. Prepared in a similar manner as General Procedure E using methyl 4-(2-(difluoromethyl)pyridin-4-yl)benzoate (87.9 mg, 0.334 mmol, 1.0 eq.) to afford the title compound as a white solid in quantitative yield. Product taken forward without purification.
^{1}H NMR (400 MHz, DMSO-*d$_6$*) δ 8.81 – 8.78 (m, 1H), 8.11 – 8.06 (m, 2H), 8.06 – 8.04 (m, 1H), 8.02 – 7.95 (m, 3H), 7.03 (t, *J* = 54.8 Hz, 1H).
HRMS (ESI-TOF) calculated for $C_{13}H_{10}F_2NO_2$ $[M+H]^+$ = 250.0674, found = 250.0674.

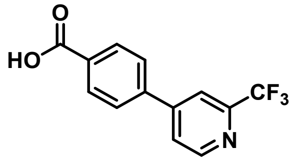

4-(2-(Trifluoromethyl)pyridin-4-yl)benzoic acid. To a vial was added *tert*-butyl 4-(2-(trifluoromethyl)pyridin-4-yl)benzoate (100.8 mg, 0.312 mmol, 1.0 eq.), which was dissolved in anhydrous DCM (2 mL). Trifluoroacetic acid (474 µL, 6.19 mmol, 20 eq.) was added, and the reaction was stirred at room temperature for 17.5 hours. The solution was then concentrated to afford the title compound as a white solid in quantitative yield.
^{1}H NMR (400 MHz, DMSO-*d$_6$*) δ 8.89 – 8.85 (m, 1H), 8.26 – 8.24 (m, 1H), 8.13 – 8.03 (m, 5H).
^{13}C NMR (101 MHz, DMSO-*d$_6$*) δ 166.8, 151.0, 148.2, 147.5 (q, $J_{C\text{-}F}$ = 33.8 Hz), 139.9, 131.9, 130.1, 127.6, 125.0, 121.7 (q, $J_{C\text{-}F}$ = 274 Hz), 118.4 (q, $J_{C\text{-}F}$ = 2.8 Hz).
HRMS (ESI-TOF) calculated for $C_{13}H_9F_3NO_2$ $[M+H]^+$ = 268.0580, found = 268.0584.

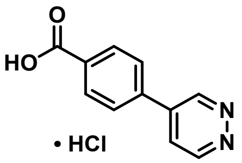

4-(Pyridazin-4-yl)benzoic acid hydrochloride. Prepared according to General Procedure E using methyl 4-(pyridazin-4-yl)benzoate (43.3 mg, 0.202 mmol, 1.0 eq.) to afford the title compound as a tan solid in quantitative yield. Product taken forward without purification.
^{1}H NMR (400 MHz, DMSO-*d$_6$*) δ 9.72 (dd, *J* = 2.4, 1.1 Hz, 1H), 9.36 (dd, *J* = 5.5, 1.0 Hz, 1H), 8.14 (dd, *J* = 5.5, 2.5 Hz, 1H), 8.12 – 8.05 (m, 4H).

HRMS (ESI-TOF) calculated for $C_{11}H_9N_2O_2$ $[M+H]^+$ = 201.0659, found = 201.0659.

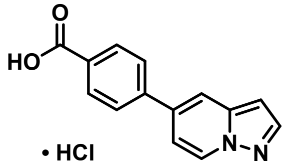

4-(Pyrazolo[1,5-*a*]pyridin-5-yl)benzoic acid hydrochloride: Prepared according to General Procedure E using methyl 4-(pyrazolo[1,5-a]pyridin-5-yl)benzoate (36.4 mg, 0.144 mmol, 1.0 eq.) to afford the title compound as a yellow solid in quantitative yield. Product taken forward without purification.
^{1}H NMR (400 MHz, DMSO-*d₆*) δ 8.78 (dt, *J* = 7.4, 0.8 Hz, 1H), 8.14-8.11 (m, 1H), 8.07-8.02 (m, 3H), 7.96 – 7.91 (m, 2H), 7.29 (dd, *J* = 7.4, 2.1 Hz, 1H), 6.71 (dd, *J* = 2.2, 0.6 Hz, 1H).
HRMS (ESI-TOF) calculated for $C_{14}H_{11}N_2O_2$ $[M+H]^+$ = 239.0815, found = 239.0818.

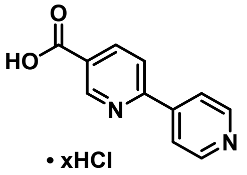

[2,4'-Bipyridine]-5-carboxylic acid hydrochloride: Prepared according to General Procedure E using methyl [2,4'-bipyridine]-5-carboxylate (96.3 mg, 0.450 mmol, 1.0 eq.) to afford the title compound as a tan solid in quantitative yield. Product taken forward without purification.
^{1}H NMR (400 MHz, DMSO-d_6) δ 9.26 (t, *J* = 1.5 Hz, 1H), 9.03 – 8.98 (m, 2H), 8.64 – 8.59 (m, 2H), 8.51 – 8.46 (m, 2H).
HRMS (ESI-TOF) calculated for $C_{11}H_9N_2O_2$ $[M+H]^+$ = 201.0659, found = 201.0661.

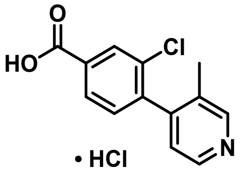

3-Chloro-4-(3-methylpyridin-4-yl)benzoic acid hydrochloride. Prepared according to General Procedure E using methyl 3-chloro-4-(3-methylpyridin-4-yl)benzoate (42.0 mg, 0.161 mmol, 1.0 eq.) to afford the title compound as an off-white solid in quantitative yield. Product taken forward without purification.
^{1}H NMR (400 MHz, DMSO-d_6) δ 8.90 (s, 1H), 8.80 (d, *J* = 5.6 Hz, 1H), 8.12 - 8.09 (m, 1H), 8.04 (dd, *J* = 8.0, 1.6 Hz, 1H), 7.76 (d, *J* = 5.6 Hz, 1H), 7.56 (d, *J* = 8.0 Hz, 1H), 2.18 (s, 3H).
^{13}C NMR (101 MHz, DMSO-d_6) δ 165.7, 151.9, 144.8, 141.8, 139.7, 134.7, 133.4, 131.3, 130.7, 130.1, 128.4, 126.2, 16.3.
HRMS (ESI-TOF) calculated for $C_{13}H_{11}ClNO_2$ $[M+H]^+$ = 248.0473, found = 248.0479.

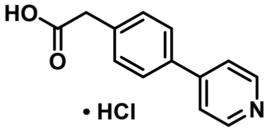

2-(4-(Pyridin-4-yl)phenyl)acetic acid hydrochloride: Prepared according to General Procedure E using methyl 2-(4-(pyridin-4-yl)phenyl)acetate (39.7 mg, 0.175 mmol, 1.0 eq.) to afford the title compound as an off-white solid in quantitative yield. Product taken forward without purification.
^{1}H NMR (400 MHz, DMSO-d_6) δ 8.94 – 8.88 (m, 2H), 8.35 – 8.29 (m, 2H), 8.00 – 7.95 (m, 2H), 7.53 – 7.48 (m, 2H), 3.71 (s, 2H).
HRMS (ESI-TOF) calculated for $C_{13}H_{12}NO_2$ $[M+H]^+$ = 214.0863, found = 214.0859.

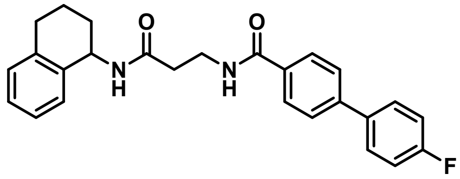

4'-Fluoro-*N*-(3-oxo-3-((1,2,3,4-tetrahydronaphthalen-1-yl)amino)propyl)-[1,1'-biphenyl]-4-carboxamide (10a): Prepared according to General Procedure D using 3-amino-*N*-(1,2,3,4-tetrahydronaphthalen-1-yl)propenamide trifluoroacetic acid (28.5 mg, 85.6 µmol, 1.0 eq.) and 4'-fluoro-[1,1'-biphenyl]-4-carboxylic acid (21.0 mg, 97.1 µmol, 1.1 eq.) to afford the title compound as a white solid (7.4 mg, 17.8 µmol, 21% yield).
^{1}H NMR (400 MHz, DMSO-*d$_6$*) δ 8.61 (t, *J* = 5.6 Hz, 1H), 8.29 (d, *J* = 8.7 Hz, 1H), 7.96 – 7.90 (m, 2H), 7.82 – 7.72 (m, 4H), 7.36 – 7.28 (m, 2H), 7.17 – 6.99 (m, 4H), 5.04 – 4.96 (m, 1H), 3.57 – 3.50 (m, 2H), 2.78 – 2.63 (m, 2H), 2.53 – 2.44 (m, 1H, partially buried under DMSO signal), 2.39 (dt, *J* = 14.3, 6.7 Hz, 1H), 1.91 – 1.81 (m, 2H), 1.75 – 1.60 (m, 2H).
^{13}C NMR (101 MHz, DMSO-*d$_6$*) δ 170.0, 165.8, 162.2 (d, $J_{C\text{-}F}$ = 245 Hz), 141.6, 137.6, 137.0, 135.7 (d, $J_{C\text{-}F}$ = 3.2 Hz), 133.3, 128.96 (d, $J_{C\text{-}F}$ = 8.1 Hz), 128.7, 128.2, 127.9, 126.6, 126.4, 125.8, 115.9 (d, $J_{C\text{-}F}$ = 21.6 Hz), 46.2, 36.4, 35.5, 30.0, 28.8, 20.0.
HRMS (ESI-TOF) calculated for $C_{26}H_{26}FN_2O_2$ [M+H]$^+$ = 417.1973, found = 417.1972.

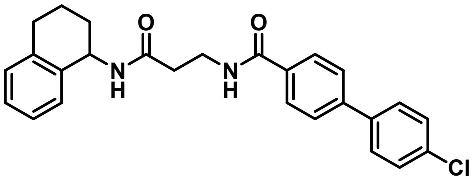

4'-Chloro-*N*-(3-oxo-3-((1,2,3,4-tetrahydronaphthalen-1-yl)amino)propyl)-[1,1'-biphenyl]-4-carboxamide (10b): Prepared according to General Procedure D using 3-amino-*N*-(1,2,3,4-tetrahydronaphthalen-1-yl)propenamide trifluoroacetic acid (28.5 mg, 85.6 µmol, 1.0 eq.) and 4'-chloro-[1,1'-biphenyl]-4-carboxylic acid (21.9 mg, 94.1 µmol, 1.1 eq.) to afford the title compound as a white solid (10.5 mg, 24.3 µmol, 28% yield).
^{1}H NMR (400 MHz, DMSO-*d$_6$*) δ 8.62 (t, *J* = 5.6 Hz, 1H), 8.30 (d, *J* = 8.7 Hz, 1H), 7.97 – 7.92 (m, 2H), 7.81 – 7.74 (m, 4H), 7.58 – 7.52 (m, 2H), 7.17 – 6.99 (m, 4H), 5.04 – 4.95 (m, 1H), 3.58 – 3.50 (m, 2H), 2.78 – 2.62 (m, 2H), 2.53 – 2.44 (m, 1H, partially buried under DMSO signal), 2.40 (dt, *J* = 14.4, 6.7 Hz, 1H), 1.91 – 1.79 (m, 2H), 1.75 – 1.59 (m, 2H).
^{13}C NMR (101 MHz, DMSO-*d$_6$*) δ 169.8, 165.7, 141.2, 138.0, 137.6, 137.0, 133.6, 133.0, 129.0, 128.7, 128.2, 127.9, 126.6, 126.4, 125.8, 46.2, 36.4, 35.5, 29.9, 28.8, 20.0. Note: 1 aromatic C buried.
HRMS (ESI-TOF) calculated for $C_{26}H_{26}ClN_2O_2$ [M+H]$^+$ = 433.1677, found = 433.1678.

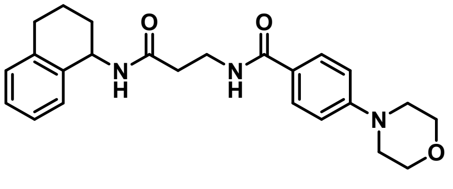

4-Morpholino-*N*-(3-oxo-3-((1,2,3,4-tetrahydronaphthalen-1-yl)amino)propyl)benzamide (10c): Prepared according to General Procedure D using 3-amino-*N*-(1,2,3,4-tetrahydronaphthalen-1-yl)propenamide trifluoroacetic acid (28.5 mg, 85.6 µmol, 1.0 eq.) and 4-(4-morpholinyl)benzoic acid (20.1 mg, 97.0 µmol, 1.1 eq.) to afford the title compound as a white solid (7.2 mg, 17.7 µmol, 21% yield).
^{1}H NMR (400 MHz, DMSO-*d$_6$*) δ 8.31 – 8.23 (m, 2H), 7.77 – 7.71 (m, 2H), 7.16 – 7.00 (m, 4H), 6.98 – 6.93 (m, 2H), 5.02 – 4.94 (m, 1H), 3.76 – 3.70 (m, 4H), 3.52 – 3.44 (m, 2H), 3.23 – 3.17 (m, 4H), 2.78 – 2.62 (m, 2H), 2.45 (dt, *J* = 14.4, 7.2 Hz, 1H), 2.36 (dt, *J* = 14.3, 6.8 Hz, 1H), 1.91 – 1.78 (m, 2H), 1.75 – 1.58 (m, 2H).
^{13}C NMR (101 MHz, DMSO-*d$_6$*) δ 169.9, 165.8, 152.8, 137.6, 137.0, 128.6, 128.4, 128.2, 126.6, 125.8, 124.2, 113.4, 65.9, 47.4, 46.2, 36.2, 35.7, 29.9, 28.8, 20.0.
HRMS (ESI-TOF) calculated for $C_{24}H_{30}N_3O_3$ [M+H]$^+$ = 408.2282, found = 408.2282.

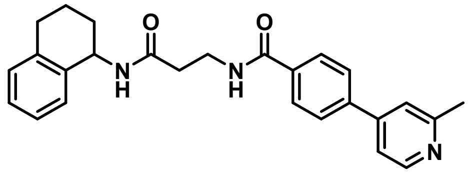

4-(2-Methylpyridin-4-yl)-*N*-(3-oxo-3-((1,2,3,4-tetrahydronaphthalen-1-yl)amino)propyl)benzamide (10d): Prepared according to General Procedure D using 3-amino-*N*-(1,2,3,4-tetrahydronaphthalen-1-yl)propenamide trifluoroacetic acid (32.8 mg, 98.8 μmol, 1.0 eq.) and 4-(2-methylpyridin-4-yl)benzoic acid hydrochloride (28.7 mg, 0.115 mmol, 1.2 eq.) to afford the title compound as a white residue (13.7 mg, 33.1 μmol, 34% yield).
^{1}H NMR (400 MHz, DMSO-*d$_6$*) δ 8.68 (t, *J* = 5.6 Hz, 1H), 8.53 (d, *J* = 5.2 Hz, 1H), 8.30 (d, *J* = 8.7 Hz, 1H), 8.01 – 7.95 (m, 2H), 7.92 – 7.87 (m, 2H), 7.66 (d, *J* = 1.3 Hz, 1H), 7.56 (dd, *J* = 5.2, 1.5 Hz, 1H), 7.18 – 6.98 (m, 4H), 5.04 – 4.96 (m, 1H), 3.58 – 3.51 (m, 2H), 2.78 – 2.62 (m, 2H), 2.55 (s, 3H), 2.54 – 2.36 (m, 2H), 1.92 – 1.79 (m, 2H), 1.75 - 1.60 (m, 2H).
^{13}C NMR (101 MHz, DMSO-*d$_6$*) δ 169.8, 165.6, 158.7, 149.6, 146.3, 139.8, 137.6, 137.0, 134.8, 128.6, 128.2, 128.0, 126.7, 126.6, 125.8, 120.6, 118.5, 46.2, 36.4, 35.4, 29.9, 28.8, 24.1, 20.0.
HRMS (ESI-TOF) calculated for $C_{26}H_{28}N_3O_2$ [M+H]$^+$ = 414.2176, found = 414.2177.

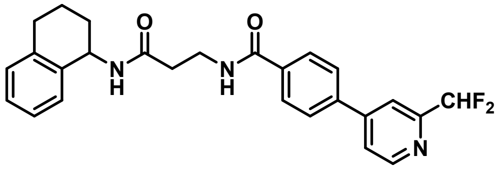

4-(2-(Difluoromethyl)pyridin-4-yl)-N-(3-oxo-3-((1,2,3,4-tetrahydronaphthalen-1-yl)amino)propyl)benzamide (10e): Prepared according to General Procedure D using 3-amino-*N*-(1,2,3,4-tetrahydronaphthalen-1-yl)propenamide trifluoroacetic acid (32.8 mg, 98.8 μmol, 1.0 eq.) and 4-(2-(difluoromethyl)pyridin-4-yl)benzoic acid hydrochloride (33.4 mg, 0.117 mmol, 1.2 eq.) to afford the title compound as a clear residue (13.3 mg, 29.6 μmol, 30% yield).
^{1}H NMR (400 MHz, DMSO-*d$_6$*) δ 8.78 (d, *J* = 5.2 Hz, 1H), 8.72 (t, *J* = 5.6 Hz, 1H), 8.31 (d, *J* = 8.7 Hz, 1H), 8.07 - 8.05 (m, 1H), 8.04 – 7.96 (m, 5H), 7.18 - 6.99 (m, 4H), 7.03 (t, *J* = 54.8 Hz, 1H), 5.05 – 4.96 (m, 1H), 3.60 – 3.51 (m, 2H), 2.78 – 2.62 (m, 2H), 2.55 – 2.36 (m, 2H), 1.92 – 1.78 (m, 2H), 1.76 – 1.59 (m, 2H).
^{13}C NMR (101 MHz, DMSO-*d$_6$*) δ 169.8, 165.5, 152.8 (t, J_{C-F} = 24.2 Hz), 150.5, 147.7, 138.7, 137.6, 137.0, 135.4, 128.6, 128.2, 128.1, 127.0, 126.6, 125.8, 123.4, 118.0 (t, J_{C-F} = 3.9 Hz), 113.8 (t, J_{C-F} = 239 Hz) 46.2, 36.4, 35.4, 29.9, 28.8, 20.0.
HRMS (ESI-TOF) calculated for $C_{26}H_{26}F_2N_3O_2$ [M+H]$^+$ = 450.1988, found = 450.1986.

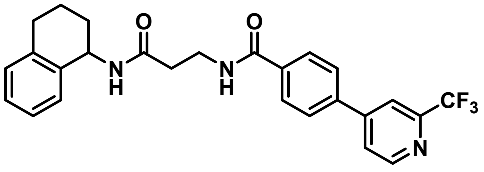

***N*-(3-oxo-3-((1,2,3,4-tetrahydronaphthalen-1-yl)amino)propyl)-4-(2-(trifluoromethyl)pyridin-4-yl)benzamide (VU6080099, 10f):** Prepared according to General Procedure D using 3-amino-*N*-(1,2,3,4-tetrahydronaphthalen-1-yl)propenamide trifluoroacetic acid (32.8 mg, 98.8 μmol, 1.0 eq.) and 4-(2-(trifluoromethyl)pyridin-4-yl)benzoic acid (31.6 mg, 0.118 mmol, 1.2 eq.) to afford the title compound as a clear residue (10.7 mg, 22.9 μmol, 23% yield).
^{1}H NMR (400 MHz, DMSO-*d$_6$*) δ 8.86 (d, *J* = 5.3 Hz, 1H), 8.74 (t, *J* = 5.6 Hz, 1H), 8.31 (d, *J* = 8.7 Hz, 1H), 8.28 – 8.25 (m, 1H), 8.17 – 8.08 (m, 1H), 8.08 – 7.99 (m, 4H), 7.19 – 6.99 (m, 4H), 5.05 – 4.96 (m, 1H), 3.60 – 3.50 (m, 2H), 2.78 – 2.62 (m, 2H), 2.55 – 2.36 (m, 2H), 1.93 – 1.78 (m, 2H), 1.76 – 1.59 (m, 2H).

^{13}C NMR (101 MHz, DMSO-*d$_6$*) δ 169.8, 165.4, 150.9, 148.3, 147.5 (q, $J_{C\text{-}F}$ = 33.7 Hz), 138.1, 137.6, 137.0, 135.6, 128.7, 128.2, 128.1, 127.2, 126.6, 125.8, 124.8, 121.7 (q, $J_{C\text{-}F}$ = 274 Hz), 118.2 (q, $J_{C\text{-}F}$ = 3.2 Hz), 46.2, 36.4, 35.4, 29.9, 28.8, 20.0.
HRMS (ESI-TOF) calculated for $C_{26}H_{25}F_3N_3O_2$ $[M+H]^+$ = 468.1893, found = 468.1890.

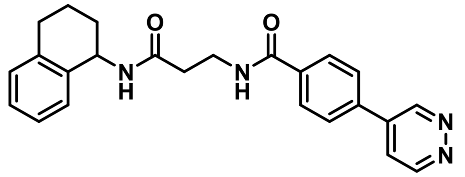

***N*-(3-oxo-3-((1,2,3,4-tetrahydronaphthalen-1-yl)amino)propyl)-4-(pyridazin-4-yl)benzamide (10g):** Prepared according to General Procedure D using 3-amino-*N*-(1,2,3,4-tetrahydronaphthalen-1-yl)propenamide trifluoroacetic acid (32.8 mg, 98.8 µmol, 1.0 eq.) and 4-(pyridazin-4-yl)benzoic acid hydrochloride (29.6 mg, 0.125 mmol, 1.3 eq.) to afford the title compound as a clear residue (7.2 mg, 18.0 µmol, 18% yield).
^{1}H NMR (400 MHz, DMSO-*d$_6$*) δ 9.71 (dd, *J* = 2.5, 1.2 Hz, 1H), 9.32 (dd, *J* = 5.5, 1.2 Hz, 1H), 8.73 (t, *J* = 5.6 Hz, 1H), 8.30 (d, *J* = 8.7 Hz, 1H), 8.09 (dd, *J* = 5.5, 2.6 Hz, 1H), 8.07 – 8.00 (m, 4H), 7.17 – 7.00 (m, 4H), 5.05 – 4.96 (m, 1H), 3.60 – 3.50 (m, 2H), 2.78 – 2.62 (m, 2H), 2.55 – 2.36 (m, 2H), 1.93 – 1.78 (m, 2H), 1.76 – 1.59 (m, 2H).
^{13}C NMR (101 MHz, DMSO-*d$_6$*) δ 169.8, 165.4, 151.7, 149.4, 137.6, 137.0, 136.4, 136.3, 135.7, 128.6, 128.2, 128.1, 127.2, 126.6, 125.8, 123.4, 46.2, 36.4, 35.4, 29.9, 28.8, 20.0.
HRMS (ESI-TOF) calculated for $C_{24}H_{25}N_4O_2$ $[M+H]^+$ = 401.1972, found = 401.1972.

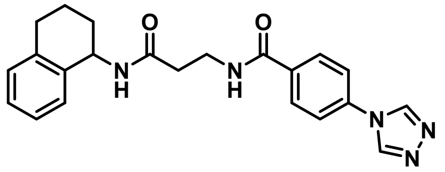

***N*-(3-oxo-3-((1,2,3,4-tetrahydronaphthalen-1-yl)amino)propyl)-4-(4*H*-1,2,4-triazol-4-yl)benzamide (10h):** Prepared according to General Procedure D using 3-amino-*N*-(1,2,3,4-tetrahydronaphthalen-1-yl)propenamide trifluoroacetic acid (28.5 mg, 85.6 µmol, 1.0 eq.) and 4-(4*H*-1,2,4-triazol-4-yl)benzoic acid (18.2 mg, 96.2 µmol, 1.1 eq.) to afford the title compound as a white solid (8.0 mg, 20.5 µmol, 24% yield).
^{1}H NMR (400 MHz, DMSO-*d$_6$*) δ 9.23 (s, 2H), 8.71 (t, *J* = 5.6 Hz, 1H), 8.30 (d, *J* = 8.7 Hz, 1H), 8.05 – 7.99 (m, 2H), 7.87 – 7.81 (m, 2H), 7.17 – 6.99 (m, 4H), 5.04 – 4.96 (m, 1H), 3.58 – 3.50 (m, 2H), 2.78 – 2.63 (m, 2H), 2.53 – 2.44 (m, 1H, partially buried under DMSO signal), 2.40 (dt, *J* = 14.4, 6.7 Hz, 1H), 1.91 – 1.79 (m, 2H), 1.75 – 1.59 (m, 2H).
^{13}C NMR (101 MHz, DMSO-*d$_6$*) δ 169.8, 165.0, 141.2, 137.6, 137.0, 135.9, 133.6, 129.0, 128.7, 128.2, 126.7, 125.8, 120.5, 46.2, 36.4, 35.4, 29.9, 28.8, 20.0.
HRMS (ESI-TOF) calculated for $C_{22}H_{24}N_5O_2$ $[M+H]^+$ = 390.1925, found = 390.1923.

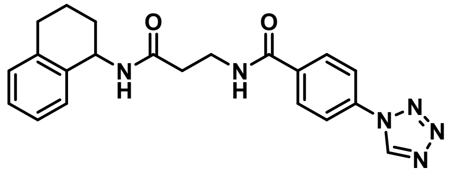

***N*-(3-oxo-3-((1,2,3,4-tetrahydronaphthalen-1-yl)amino)propyl)-4-(1*H*-tetrazol-1-yl)benzamide (10i):** Prepared according to General Procedure D using 3-amino-*N*-(1,2,3,4-tetrahydronaphthalen-1-yl)propenamide trifluoroacetic acid (28.5 mg, 85.6 µmol, 1.0 eq.) and 4-(1*H*-tetrazol-1-yl)benzoic acid (18.3 mg, 96.2 µmol, 1.1 eq.) to afford the title compound as a white solid (6.9 mg, 17.7 µmol, 21% yield).
^{1}H NMR (400 MHz, DMSO-*d$_6$*) δ 10.19 (s, 1H), 8.78 (t, *J* = 5.6 Hz, 1H), 8.31 (d, *J* = 8.7 Hz, 1H), 8.13 – 8.07 (m, 2H), 8.07 – 8.02 (m, 2H), 7.18 – 6.99 (m, 4H), 5.04 – 4.96 (m, 1H), 3.59 – 3.51 (m, 2H), 2.78 – 2.63 (m, 2H),

2.53 – 2.45 (m, 1H, partially buried under DMSO signal), 2.41 (dt, *J* = 14.4, 6.7 Hz, 1H), 1.91 – 1.78 (m, 2H), 1.76 – 1.59 (m, 2H).
^{13}C NMR (101 MHz, DMSO-*d₆*) δ 169.7, 164.9, 142.4, 137.6, 137.0, 135.5, 135.3, 129.0, 128.7, 128.2, 126.6, 125.8, 120.7, 46.2, 36.4, 35.4, 29.9, 28.8, 20.0.
HRMS (ESI-TOF) calculated for $C_{21}H_{23}N_6O_2$ [M+H]$^+$ = 391.1877, found = 391.1876.

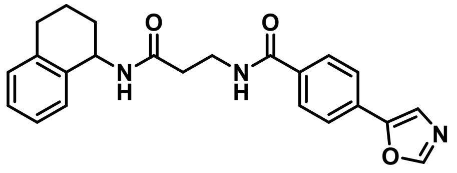

4-(Oxazol-5-yl)-*N*-(3-oxo-3-((1,2,3,4-tetrahydronaphthalen-1-yl)amino)propyl)benzamide (10j): Prepared according to General Procedure D using 3-amino-*N*-(1,2,3,4-tetrahydronaphthalen-1-yl)propenamide trifluoroacetic acid (28.5 mg, 85.6 μmol, 1.0 eq.) and 4-(5-oxazolyl)benzoic acid (18.3 mg, 96.7 μmol, 1.1 eq.) to afford the title compound as a white solid (9.4 mg, 24.1 μmol, 28% yield).
^{1}H NMR (400 MHz, DMSO-*d₆*) δ 8.65 (t, *J* = 5.6 Hz, 1H), 8.51 (s, 1H), 8.30 (d, *J* = 8.7 Hz, 1H), 7.98 – 7.92 (m, 2H), 7.85 – 7.80 (m, 3H), 7.17 – 6.98 (m, 4H), 5.04 – 4.95 (m, 1H), 3.57 – 3.49 (m, 2H), 2.78 – 2.62 (m, 2H), 2.53 – 2.44 (m, 1H, partially buried under DMSO signal), 2.39 (dt, *J* = 14.3, 6.7 Hz, 1H), 1.93 – 1.77 (m, 2H), 1.76 – 1.58 (m, 2H).
^{13}C NMR (101 MHz, DMSO-*d₆*) δ 169.8, 165.4, 152.4, 149.9, 137.6, 137.0, 134.1, 129.7, 128.7, 128.2, 128.1, 126.6, 125.7, 123.8, 123.4, 46.2, 36.4, 35.5, 29.9, 28.8, 20.0.
HRMS (ESI-TOF) calculated for $C_{23}H_{24}N_3O_3$ [M+H]$^+$ = 390.1812, found = 390.1810.

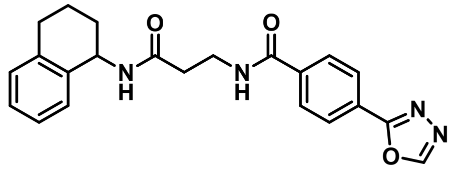

4-(1,3,4-Oxadiazol-2-yl)-*N*-(3-oxo-3-((1,2,3,4-tetrahydronaphthalen-1-yl)amino)propyl)benzamide (10k): Prepared according to General Procedure D using 3-amino-*N*-(1,2,3,4-tetrahydronaphthalen-1-yl)propenamide trifluoroacetic acid (32.8 mg, 98.8 μmol, 1.0 eq.) and 4-(1,3,4-oxadiazol-2-yl)benzoic acid (22.5 mg, 0.118 mmol, 1.2 eq.) to afford the title compound as a white solid (12.5 mg, 32.0 μmol, 32% yield).
^{1}H NMR (400 MHz, DMSO-*d₆*) δ 9.41 (s, 1H), 8.79 (t, *J* = 5.6 Hz, 1H), 8.31 (d, *J* = 8.7 Hz, 1H), 8.15 – 8.10 (m, 2H), 8.07 – 8.03 (m, 2H), 7.18 – 6.98 (m, 4H), 5.04 – 4.96 (m, 1H), 3.59 - 3.51 (m, 2H), 2.78 - 2.62 (m, 2H), 2.54 – 2.36 (m, 2H), 1.92 – 1.77 (m, 2H), 1.76 – 1.58 (m, 2H).
^{13}C NMR (101 MHz, DMSO-*d₆*) δ 169.7, 165.2, 163.2, 154.9, 137.6, 137.4, 137.0, 128.7, 128.3, 128.2, 126.7, 126.6, 125.7, 125.3, 46.2, 36.4, 35.4, 29.9, 28.8, 20.0.
HRMS (ESI-TOF) calculated for $C_{22}H_{23}N_4O_3$ [M+H]$^+$ = 391.1765, found = 391.1770.

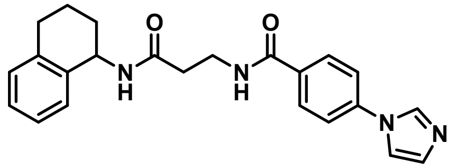

4-(1*H*-imidazol-1-yl)-*N*-(3-oxo-3-((1,2,3,4-tetrahydronaphthalen-1-yl)amino)propyl)benzamide (10l): Prepared according to General Procedure D using 3-amino-*N*-(1,2,3,4-tetrahydronaphthalen-1-yl)propenamide trifluoroacetic acid (32.8 mg, 98.8 μmol, 1.0 eq.) and 4-(1-imidazolyl)benzoic acid (22.1 mg, 0.117 mmol, 1.2 eq.) to afford the title compound as a white solid (18.3 mg, 47.1 μmol, 48% yield).
^{1}H NMR (400 MHz, DMSO-*d₆*) δ 8.66 (t, *J* = 5.6 Hz, 1H), 8.39 (t, *J* = 1.0 Hz, 1H), 8.30 (d, *J* = 8.7 Hz, 1H), 8.02 – 7.96 (m, 2H), 7.86 (t, *J* = 1.3 Hz, 1H), 7.82 – 7.76 (m, 2H), 7.17 – 6.99 (m, 5H), 5.04 – 4.95 (m, 1H), 3.60 – 3.48 (m, 2H), 2.79 – 2.62 (m, 2H), 2.54 – 2.34 (m, 2H), 1.92 – 1.78 (m, 2H), 1.76 – 1.59 (m, 2H).

^{13}C NMR (101 MHz, DMSO-*d$_6$*) δ 169.8, 165.1, 138.8, 137.6, 137.0, 135.6, 132.4, 130.2, 128.9, 128.6, 128.2, 126.6, 125.8, 119.4, 117.8, 46.2, 36.4, 35.5, 29.9, 28.8, 20.0.
HRMS (ESI-TOF) calculated for $C_{23}H_{25}N_4O_2$ $[M+H]^+$ = 389.1972, found = 389.1971.

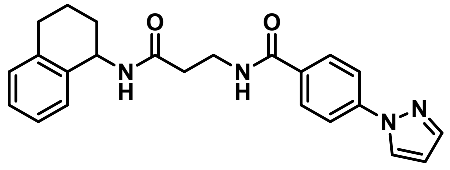

***N*-(3-oxo-3-((1,2,3,4-tetrahydronaphthalen-1-yl)amino)propyl)-4-(1*H*-pyrazol-1-yl)benzamide (10m):** Prepared according to General Procedure D using 3-amino-*N*-(1,2,3,4-tetrahydronaphthalen-1-yl)propenamide trifluoroacetic acid (32.8 mg, 98.8 µmol, 1.0 eq.) and 4-(1*H*-pyrazol-1-yl)benzoic acid (23.0 mg, 0.122 mmol, 1.2 eq.) to afford the title compound as an off-white solid (16.5 mg, 42.5 µmol, 43% yield).
^{1}H NMR (400 MHz, DMSO-*d$_6$*) δ 8.66 – 8.58 (m, 2H), 8.30 (d, *J* = 8.7 Hz, 1H), 8.01 – 7.96 (m, 2H), 7.96 – 7.92 (m, 2H), 7.80 (d, *J* = 1.6 Hz, 1H), 7.18 – 6.99 (m, 4H), 6.59 (dd, *J* = 2.4, 1.8 Hz, 1H), 5.05 – 4.95 (m, 1H), 3.59 – 3.48 (m, 2H), 2.78 – 2.62 (m, 2H), 2.53 – 2.34 (m, 2H), 1.92 – 1.78 (m, 2H), 1.76 – 1.59 (m, 2H).
^{13}C NMR (101 MHz, DMSO-*d$_6$*) δ 169.8, 165.3, 141.6, 141.5, 137.6, 137.0, 131.8, 128.7, 128.6, 128.2, 128.1, 126.6, 125.7, 117.6, 108.4, 46.2, 36.3, 35.5, 29.9, 28.8, 20.0.
HRMS (ESI-TOF) calculated for $C_{23}H_{25}N_4O_2$ $[M+H]^+$ = 389.1972, found = 389.1974.

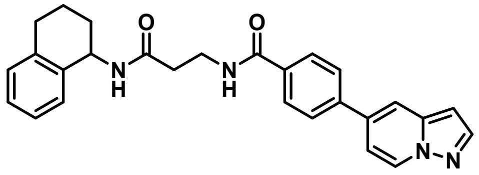

***N*-(3-oxo-3-((1,2,3,4-tetrahydronaphthalen-1-yl)amino)propyl)-4-(pyrazolo[1,5-*a*]pyridin-5-yl)benzamide (10n):** Prepared according to General Procedure D using 3-amino-*N*-(1,2,3,4-tetrahydronaphthalen-1-yl)propenamide trifluoroacetic acid (32.8 mg, 98.8 µmol, 1.0 eq.) and 4-(pyrazolo[1,5-*a*]pyridin-5-yl)benzoic acid hydrochloride (32.1 mg, 0.117 mmol, 1.2 eq.) to yield the title compound as an off-white residue (8.8 mg, 20.1 µmol, 20% yield).
^{1}H NMR (400 MHz, DMSO-*d$_6$*) δ 8.77 (dt, *J* = 7.3, 0.8 Hz, 1H), 8.65 (t, *J* = 5.6 Hz, 1H), 8.30 (d, *J* = 8.7 Hz, 1H), 8.12 (dd, *J* = 2.0, 0.8 Hz, 1H), 8.04 (d, *J* = 2.2 Hz, 1H), 8.00 – 7.95 (m, 2H), 7.94 – 7.89 (m, 2H), 7.30 (dd, *J* = 7.3, 2.0 Hz, 1H), 7.18 – 7.00 (m, 4H), 6.69 (dd, *J* = 2.3, 0.7 Hz, 1H), 5.04 – 4.96 (m, 1H), 3.59 – 3.51 (m, 2H), 2.79 – 2.63 (m, 2H), 2.54 – 2.36 (m, 2H), 1.92 – 1.80 (m, 2H), 1.75 – 1.60 (m, 2H).
^{13}C NMR (101 MHz, DMSO-*d$_6$*) δ 169.8, 165.6, 142.4, 140.2, 139.7, 137.6, 137.0, 134.1, 133.9, 129.0, 128.6, 128.2, 127.9, 126.6, 126.3, 125.8, 115.1, 111.0, 97.9, 46.2, 36.4, 35.5, 29.9, 28.8, 20.0.
HRMS (ESI-TOF) calculated for $C_{27}H_{27}N_4O_2$ $[M+H]^+$ = 439.2129, found = 439.2128.

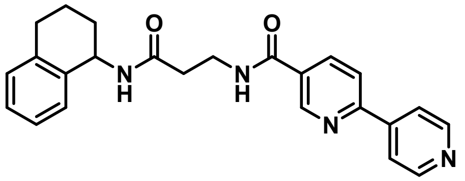

***N*-(3-oxo-3-((1,2,3,4-tetrahydronaphthalen-1-yl)amino)propyl)-[2,4'-bipyridine]-5-carboxamide (10o):** Prepared according to General Procedure D using 3-amino-*N*-(1,2,3,4-tetrahydronaphthalen-1-yl)propenamide trifluoroacetic acid (32.8 mg, 98.8 µmol, 1.0 eq.) and [2,4'-bipyridine]-5-carboxylic acid hydrochloride (27.4 mg, 0.116 mmol, 1.2 eq.) to yield the title compound as a white residue (14.2 mg, 35.5 µmol, 36% yield).
^{1}H NMR (400 MHz, DMSO-*d$_6$*) δ 9.15 (dd, *J* = 2.2, 0.6 Hz, 1H), 8.89 (t, *J* = 5.6 Hz, 1H), 8.76 – 8.71 (m, 2H), 8.35 (dd, *J* = 8.3, 2.3 Hz, 1H), 8.32 (d, *J* = 8.7 Hz, 1H), 8.25 (dd, *J* = 8.4, 0.5 Hz, 1H), 8.13 – 8.10 (m, 2H), 7.17 – 6.97 (m, 4H), 5.05 – 4.95 (m, 1H), 3.62 – 3.52 (m, 2H), 2.78 – 2.62 (m, 2H), 2.56 – 2.37 (m, 2H), 1.93 – 1.78 (m, 2H), 1.75 – 1.58 (m, 2H).

^{13}C NMR (101 MHz, DMSO-*d$_6$*) δ 169.7, 164.3, 155.4, 150.5, 148.9, 144.7, 137.5, 137.0, 136.3, 129.9, 128.7, 128.2, 126.6, 125.7, 121.0, 120.6, 46.2, 36.4, 35.4, 29.9, 28.8, 20.0.
HRMS (ESI-TOF) calculated for $C_{24}H_{25}N_4O_2$ $[M+H]^+$ = 401.1972, found = 401.1971.

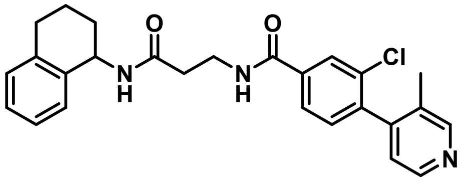

3-Chloro-4-(3-methylpyridin-4-yl)-*N*-(3-oxo-3-((1,2,3,4-tetrahydronaphthalen-1-yl)amino)propyl)benzamide (10p): Prepared according to General Procedure D using 3-amino-*N*-(1,2,3,4-tetrahydronaphthalen-1-yl)propenamide trifluoroacetic acid (33.9 mg, 0.102 mmol, 1.0 eq.) and 3-chloro-4-(3-methylpyridin-4-yl)benzoic acid hydrochloride (39.8 mg, 0.140 mmol, 1.4 eq.) to afford the title compound as a white residue (9.0 mg, 20.1 μmol, 20% yield).
^{1}H NMR (400 MHz, DMSO-*d$_6$*) δ 8.80 (t, *J* = 5.2 Hz, 1H), 8.59 – 8.55 (m, 1H), 8.50 (d, *J* = 4.9 Hz, 1H), 8.30 (d, *J* = 8.8 Hz, 1H), 8.07 (d, *J* = 9.6 Hz, 1H), 7.95 – 7.89 (m, 1H), 7.45 (dd, *J* = 7.8, 1.5 Hz, 1H), 7.20 (d, *J* = 4.9 Hz, 1H), 7.17 – 7.03 (m, 3H), 7.02 – 6.95 (m, 1H), 5.05 – 4.96 (m, 1H), 3.55 (q, *J* = 6.3 Hz, 2H), 2.78 – 2.63 (m, 2H), 2.55 – 2.45 (m, 1H, overlapping with DMSO signal), 2.44 – 2.35 (m, 1H), 2.07 (s, 3H), 1.93 – 1.77 (m, 2H), 1.76 – 1.69 (m, 2H).
^{13}C NMR (101 MHz, DMSO-*d$_6$*) δ 169.7, 164.3, 150.7, 147.3, 145.6, 139.7, 137.6, 137.0, 136.0, 131.5, 130.9, 130.6, 128.7, 128.2, 128.1, 126.6, 126.4, 126.3, 125.7, 123.6, 46.2, 36.5, 35.4, 29.9, 28.8, 20.0, 16.1. Note: Doubling/broadening of some peaks (128.1 ppm - broad; 126.4 ppm, 126.3 ppm - split) likely due to slowly interconverting atropisomers.
HRMS (ESI-TOF) calculated for $C_{26}H_{27}ClN_3O_2$ $[M+H]^+$ = 448.1786, found = 448.1790.

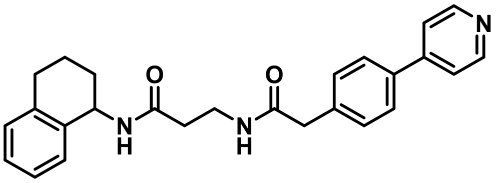

3-(2-(4-(Pyridin-4-yl)phenyl)acetamido)-*N*-(1,2,3,4-tetrahydronaphthalen-1-yl)propenamide (10q): Prepared according to General Procedure D using 3-amino-*N*-(1,2,3,4-tetrahydronaphthalen-1-yl)propenamide trifluoroacetic acid (33.9 mg, 0.102 mmol, 1.0 eq.) and 2-(4-(pyridin-4-yl)phenyl)acetic acid hydrochloride (43.6 mg, 0.175 mmol, 1.7 eq.) to afford the title compound as a white solid (8.6 mg, 20.8 μmol, 20% yield).
^{1}H NMR (400 MHz, DMSO-*d$_6$*) δ 8.65 – 8.59 (m, 2H), 8.24 (d, *J* = 8.7 Hz, 1H), 8.19 (t, *J* = 5.6 Hz, 1H), 7.75 – 7.69 (m, 2H), 7.69 – 7.65 (m, 2H), 7.42 – 7.36 (m, 2H), 7.19 – 7.04 (m, 4H), 5.03 – 4.94 (m, 1H), 3.52 – 3.42 (m, 2H), 3.33 – 3.27 (m, 2H, partially buried under H_2O signal), 2.79 – 2.63 (m, 2H), 2.38 – 2.23 (m, 2H), 1.90 – 1.78 (m, 2H), 1.75 – 1.58 (m, 2H).
^{13}C NMR (101 MHz, DMSO-*d$_6$*) δ 169.8, 169.7, 150.2, 146.8, 137.8, 137.6, 137.0, 135.1, 129.8, 128.7, 128.2, 126.64, 126.61, 125.8, 121.0, 46.2, 42.0, 35.6, 35.4, 29.9, 28.8, 20.0.
HRMS (ESI-TOF) calculated for $C_{26}H_{28}N_3O_2$ $[M+H]^+$ = 414.2176, found = 414.2181.

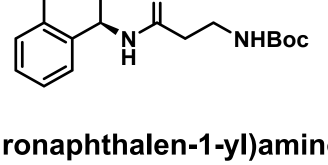

***Tert*-Butyl (*R*)-(3-oxo-3-((1,2,3,4-tetrahydronaphthalen-1-yl)amino)propyl)carbamate.** Prepared according to General Procedure B using (*R*)-1,2,3,4-tetrahydronaphthalen-1-amine (147 mg, 1.0 mmol, 1.0 eq.) and boc-*β*-alanine (208 mg, 1.1 mmol, 1.1 eq.) to afford the title compound as a white solid (272 mg, 85% yield).

^{1}H NMR (400 MHz, DMSO-d_6) δ 8.21 (d, *J* = 8.7 Hz, 1H), 7.19 – 7.09 (m, 3H), 7.09 – 7.05 (m, 1H), 6.75 (t, *J* = 5.7 Hz, 1H), 4.98 - 4.93 (m, 1H), 3.16 (q, *J* = 6.5 Hz, 2H), 2.79 – 2.62 (m, 2H), 2.35 – 2.19 (m, 2H), 1.90 – 1.80 (m, 2H), 1.76 – 1.60 (m, 2H), 1.37 (s, 9H).

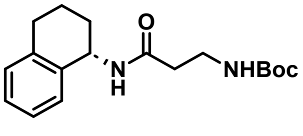

***Tert*-Butyl (*S*)-(3-oxo-3-((1,2,3,4-tetrahydronaphthalen-1-yl)amino)propyl)carbamate.** Prepared according to General Procedure B using (*S*)-1,2,3,4-tetrahydronaphthalen-1-amine (147 mg, 1.0 mmol, 1.0 eq.) and Boc-β-alanine (208 mg, 1.1 mmol, 1.1 eq.) to afford the title compound as a white solid (300 mg, 94% yield).

^{1}H NMR (400 MHz, DMSO-d_6) δ 8.21 (d, *J* = 8.6 Hz, 1H), 7.18 – 7.11 (m, 3H), 7.11 – 7.05 (m, 1H), 6.75 (t, *J* = 5.7 Hz, 1H), 4.98 - 4.93 (m, 1H), 3.16 (q, *J* = 6.7 Hz, 2H), 2.79 – 2.67 (m, 2H), 2.36 – 2.18 (m, 2H), 1.88 - 1.82 (m, 2H), 1.73 - 1.60 (m, 2H), 1.37 (s, 9H).

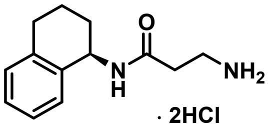

(*R*)-3-amino-*N*-(1,2,3,4-tetrahydronaphthalen-1-yl)propanamide: Prepared according to General Procedure C with 4M HCl in 1,4-dioxane using *tert*-butyl (*R*)-(3-oxo-3-((1,2,3,4-tetrahydronaphthalen-1-yl)amino)propyl)carbamate (245 mg, 0.77 mmol, 1.0 eq.) to afford the title compound as a white powder . Product taken to the next step without purification.

^{1}H NMR (400 MHz, DMSO-d_6) δ 8.48 (d, *J* = 8.6 Hz, 1H), 7.20 – 7.05 (m, 4H), 5.04 – 4.94 (m, 1H), 3.03 (t, *J* = 6.8 Hz, 2H), 2.82 – 2.63 (m, 2H), 2.54 - 2.50 (m, 2H, overlapping with DMSO signal), 1.94 – 1.79 (m, 2H), 1.79 – 1.61 (m, 2H), NH_2 not observable.

^{13}C NMR (101 MHz, DMSO-d_6) δ 168.7, 137.3, 137.1, 128.8, 128.3, 126.8, 125.8, 46.3, 35.3, 32.1, 29.8, 28.8, 19.9.

HRMS (ESI-TOF) calculated for $C_{13}H_{19}N_2O$ $[M+H]^+$ = 219.1492, found = 219.1497.

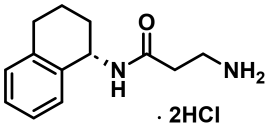

(*S*)-3-amino-*N*-(1,2,3,4-tetrahydronaphthalen-1-yl)propanamide: Prepared according to General Procedure C with 4M HCl in 1,4-dioxane using *tert*-butyl (*S*)-(3-oxo-3-((1,2,3,4-tetrahydronaphthalen-1-yl)amino)propyl)carbamate (265 mg, 0.83 mmol, 1.0 eq.) to afford the title compound as a white powder. Product taken to the next step without purification.

^{1}H NMR (400 MHz, DMSO-d_6) δ 8.48 (d, *J* = 8.6 Hz, 1H), 7.21 – 7.06 (m, 4H), 5.04 – 4.94 (m, 1H), 3.03 (t, *J* = 6.9 Hz, 2H), 2.82 – 2.63 (m, 2H), 2.54 - 2.50 (m, 2H, overlapping with DMSO signal), 1.94 – 1.79 (m, 2H), 1.79 – 1.60 (m, 2H), NH_2 not observable.

^{13}C NMR (101 MHz, DMSO-d_6) δ 168.7, 137.3, 137.1, 128.8, 128.3, 126.8, 125.8, 46.3, 35.3, 32.1, 29.8, 28.8, 19.9.

HRMS (ESI-TOF) calculated for $C_{13}H_{19}N_2O$ $[M+H]^+$ = 219.1492, found = 219.1497.

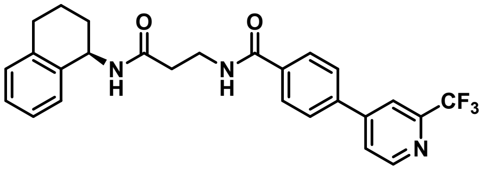

(*R*)-*N*-(3-oxo-3-((1,2,3,4-tetrahydronaphthalen-1-yl)amino)propyl)-4-(2-(trifluoromethyl)pyridin-4-yl)benzamide (VU6083858, (*R*)-10f): Prepared according to General Procedure D (*R*)-3-amino-*N*-(1,2,3,4-tetrahydronaphthalen-1-yl)propenamide HCl salt (29 mg, 0.1 mmol, 1.0 eq.) and 4-(2-(trifluoromethyl)pyridin-4-yl)benzoic acid (29.4 mg, 0.11 mmol, 1.1 eq.) to afford the title compound as a white powder (20 mg, 43% yield).

^{1}H NMR (400 MHz, DMSO-*d$_6$*) δ 8.86 (d, *J* = 5.1 Hz, 1H), 8.74 (t, *J* = 5.6 Hz, 1H), 8.31 (d, *J* = 8.7 Hz, 1H), 8.27 (d, *J* = 1.7 Hz, 1H), 8.13 (dd, *J* = 5.2, 1.7 Hz, 1H), 8.09 – 7.98 (m, 4H), 7.15 (d, J = 7.6 Hz, 1H), 7.13 - 7.00 (m, 3H), 5.03 - 4.98 (m, 1H), 3.60 – 3.52 (m, 2H), 2.77 - 2.64 (m, 2H), 2.50 - 2.37 (m, 2H, partially buried under DMSO), 1.92 – 1.82 (m, 2H), 1.76 – 1.59 (m, 2H).

^{13}C NMR (101 MHz, DMSO-*d$_6$*) δ 169.8, 165.4, 150.9, 148.3, 147.5 (q, J_{C-F} = 33.7 Hz), 138.1, 137.6, 137.0, 135.6, 128.7, 128.2, 128.1, 127.2, 126.6, 125.8, 124.8, 121.7 (q, J_{C-F} = 274 Hz), 118.2 (q, J_{C-F} = 3.2 Hz), 46.2, 36.4, 35.4, 29.9, 28.8, 20.0.

HRMS (ESI-TOF) calculated for $C_{26}H_{25}F_3N_3O_2$ $[M+H]^+$ = 468.1893, found = 468.1899.

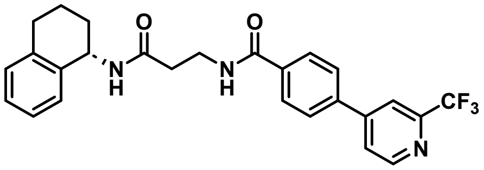

(*S*)-*N*-(3-oxo-3-((1,2,3,4-tetrahydronaphthalen-1-yl)amino)propyl)-4-(2-(trifluoromethyl)pyridin-4-yl)benzamide (VU6083859, (*S*)-10f): Prepared according to General Procedure D (*S*)-3-amino-*N*-(1,2,3,4-tetrahydronaphthalen-1-yl)propenamide HCl salt (29 mg, 0.1 mmol, 1.0 eq.) and 4-(2-(trifluoromethyl)pyridin-4-yl)benzoic acid (29.4 mg, 0.11 mmol, 1.1 eq.) to afford the title compound as a white powder (18 mg, 39% yield).

^{1}H NMR (400 MHz, DMSO-*d$_6$*) 1H NMR (400 MHz, DMSO) δ 8.86 (d, *J* = 5.1 Hz, 1H), 8.74 (t, *J* = 5.6 Hz, 1H), 8.31 (d, *J* = 8.7 Hz, 1H), 8.28 – 8.24 (m, 1H), 8.13 (dd, *J* = 5.1, 1.7 Hz, 1H), 8.09 – 7.98 (m, 4H), 7.15 (d, *J* = 7.6 Hz, 1H), 7.13 - 7.00 (m, 3H), 5.03 - 4.98 (m, 1H), 3.60 – 3.52 (m, 2H), 2.76 - 2.65 (m, 2H), 2.54 - 2.38 (m, 2H, partially buried under DMSO), 1.90 – 1.82 (m, 2H), 1.76 – 1.59 (m, 2H).

^{13}C NMR (101 MHz, DMSO-*d$_6$*) δ 169.8, 165.4, 150.9, 148.3, 147.5 (q, J_{C-F} = 33.7 Hz), 138.1, 137.6, 137.0, 136.0, 128.7, 128.2, 128.1, 127.2, 126.6, 125.8, 124.8, 121.7 (q, J_{C-F} = 274 Hz), 118.2 (q, J_{C-F} = 3.2 Hz), 46.2, 36.4, 35.4, 29.9, 28.8, 20.0.

HRMS (ESI-TOF) calculated for $C_{26}H_{25}F_3N_3O_2$ $[M+H]^+$ = 468.1893, found = 468.1897.

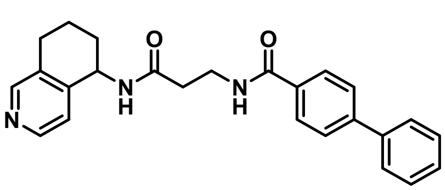

***N*-(3-oxo-3-((5,6,7,8-tetrahydroisoquinolin-5-yl)amino)propyl)-[1,1'-biphenyl]-4-carboxamide (10r).**
To a vial was added biphenyl-4-carboxylic acid (255.6 mg, 1.29 mmol, 1.2 eq.) and HATU (490.3 mg, 1.29 mmol, 1.2 eq.), which were dissolved in DMF (2.5 mL). DIPEA (562 µL, 3.22 mmol, 3 eq.) was added. The solution was

stirred at room temperature for 10 minutes, then β-Alanine methyl ester hydrochloride (150 mg, 1.07 mmol, 1.0 eq.) was added. After 90 minutes, the reaction was quenched with 25 mL H_2O and extracted with 25 mL EtOAc. The organic layer was washed with brine and concentrated. The crude material was purified via automated flash column chromatography (Teledyne ISCO, 0-70% EtOAc in Hexanes) to afford methyl 3-([1,1'-biphenyl]-4-carboxamido)propanoate as a white solid (335.5 mg, 1.02 mmol, 95% yield). ES-MS $[M+H]^+$ = 284.2.

A vial was then charged with methyl 3-([1,1'-biphenyl]-4-carboxamido)propanoate (262.4 mg, 0.926 mmol, 1.0 eq.), which was dissolved in 1,4-dioxane (2.3 mL). NaOH (51.9 mg, 1.30 mmol, 1.4 eq.) was added. The mixture was stirred at room temperature. Upon completion, the reaction was neutralized, then extracted with EtOAc. The organic layers were combined and concentrated to give 3-([1,1'-biphenyl]-4-carboxamido)propanoic acid as a white solid (90 mg, 0.328 mmol, 35% yield). ES-MS $[M+H]^+$ = 270.2.

The title compound was then prepared in a similar manner as General Procedure A using 3-([1,1'-biphenyl]-4-carboxamido)propanoic acid (8.0 mg, 29.7 μmol, 1.0 eq.) and 5,6,7,8-tetrahydro-5-isoquinolinamine hydrochloride (6.6 mg, 35.6 μmol, 1.2 eq.) to afford the desired product as a white residue (3.9 mg, 9.8 μmol, 33% yield).
^{1}H NMR (400 MHz, DMSO-*d₆*) δ 8.66 (t, *J* = 5.6 Hz, 1H), 8.39 (d, *J* = 8.6 Hz, 1H), 8.28 (s, 1H), 8.15 (d, *J* = 5.1 Hz, 1H), 7.98 – 7.93 (m, 2H), 7.80 – 7.70 (m, 4H), 7.53 – 7.46 (m, 2H), 7.44 – 7.38 (m, 1H), 7.10 (d, *J* = 5.1 Hz, 1H), 4.97 (td, *J* = 8.5, 5.0 Hz, 1H), 3.59 – 3.52 (m, 2H), 2.74 – 2.66 (m, 2H), 2.57 – 2.38 (m, 2H), 1.96 – 1.83 (m, 2H), 1.79 – 1.57 (m, 2H).
^{13}C NMR (101 MHz, DMSO-*d₆*) δ 170.3, 165.8, 149.9, 146.7, 146.4, 142.6, 139.2, 133.3, 132.7, 129.1, 128.1, 127.9, 126.9, 126.5, 122.3, 45.7, 36.3, 35.5, 29.2, 25.5, 20.1.
HRMS (ESI-TOF) calculated for $C_{25}H_{26}N_3O_2$ $[M+H]^+$ = 400.2020, found = 400.2019.

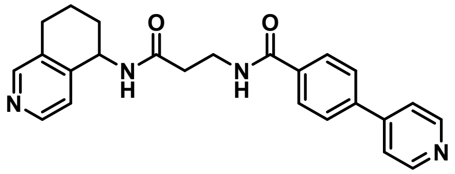

***N*-(3-oxo-3-((5,6,7,8-tetrahydroisoquinolin-5-yl)amino)propyl)-4-(pyridin-4-yl)benzamide (10s):** Prepared in a similar manner as General Procedure A using 3-(4-(pyridin-4-yl)benzamido)propanoic acid hydrogen chloride (66.5 mg, 0.217 mmol, 1.0 eq.) and 5,6,7,8-tetrahydro-5-isoquinolinamine hydrochloride (53.4 mg, 0.289 mmol, 1.3 eq.) to afford the title compound as a clear residue (21.2 mg, 52.9 μmol, 24% yield).
^{1}H NMR (400 MHz, MeOD-*d₄*) δ 8.68 – 8.56 (m, 2H), 8.25 (s, 1H), 8.09 (s, 1H), 7.99 – 7.93 (m, 2H), 7.89 – 7.83 (m, 2H), 7.80 – 7.72 (m, 2H), 7.21 (d, *J* = 5.1 Hz, 1H), 5.10 (dd, *J* = 8.1, 5.4 Hz, 1H), 3.74 (t, *J* = 6.7 Hz, 2H), 2.82 – 2.74 (m, 2H), 2.72 – 2.54 (m, 2H), 2.09 – 1.91 (m, 2H), 1.88 – 1.69 (m, 2H). Note: Amide -NH signals not observed in MeOD-*d₄*.
^{13}C NMR (101 MHz, MeOD-*d₄*) δ 173.5, 169.4, 150.8, 150.6, 149.5, 148.5, 147.2, 141.9, 136.3, 135.2, 129.3, 128.3, 124.1, 123.3, 48.0, 37.9, 36.8, 30.5, 26.9, 21.3.
HRMS (ESI-TOF) calculated for $C_{24}H_{25}N_4O_2$ = 401.1972, found = 401.1971.

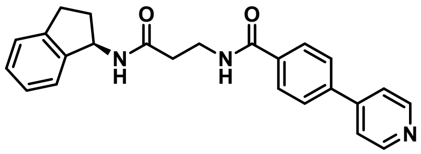

(*R*)-*N*-(3-((2,3-dihydro-1H-inden-1-yl)amino)-3-oxopropyl)-4-(pyridin-4-yl)benzamide (10t): Prepared in a similar manner as General Procedure A using 3-(4-(pyridin-4-yl)benzamido)propanoic acid hydrogen chloride (34.4 mg, 0.115 mmol, 1.0 eq.) and (*R*)-2,3-dihydro-1*H*-inden-1-amine hydrochloride (19.0 mg, 0.112 mmol, 1.0 eq.) to afford the title compound as a white solid (17.3 mg, 44.9 μmol, 40% yield).
^{1}H NMR (400 MHz, DMSO-*d₆*) δ 8.73 – 8.63 (m, 3H), 8.29 (d, *J* = 8.4 Hz, 1H), 8.02 – 7.96 (m, 2H), 7.94 – 7.89 (m, 2H), 7.80 – 7.75 (m, 2H), 7.24 – 7.14 (m, 3H), 7.13 – 7.07 (m, 1H), 5.31 (q, *J* = 8.0 Hz, 1H), 3.61 – 3.49 (m,

2H), 2.90 (ddd, *J* = 15.8, 8.8, 3.1 Hz, 1H), 2.78 (dt, *J* = 15.8, 8.3 Hz, 1H), 2.54 – 2.40 (m, 2H, partially buried under DMSO signal), 2.40 – 2.31 (m, 1H), 1.77 (dq, *J* = 12.6, 8.7 Hz, 1H).
^{13}C NMR (101 MHz, DMSO-*d_6*) δ 170.2, 165.6, 150.4, 146.0, 144.1, 142.8, 139.6, 134.9, 128.0, 127.3, 126.7, 126.3, 124.4, 124.0, 121.3, 53.4, 36.3, 35.4, 32.9, 29.7.
HRMS (ESI-TOF) calculated for $C_{24}H_{24}N_3O_2$ $[M+H]^+$ = 386.1863, found = 386.1868.

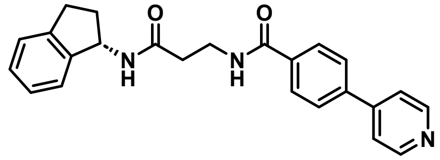

(*S*)-*N*-(3-((2,3-dihydro-1H-inden-1-yl)amino)-3-oxopropyl)-4-(pyridin-4-yl)benzamide (10u): Prepared in a similar manner as General Procedure A using 3-(4-(pyridin-4-yl)benzamido)propanoic acid hydrogen chloride (34.4 mg, 0.115 mmol, 1.1 eq.) and (*S*)-(+)-1-aminoindan (14.0 µL, 0.109 mmol, 1.0 eq.) to afford the title compound as a tan solid (18.1 mg, 47.0 µmol, 43% yield).
^{1}H NMR (400 MHz, DMSO-*d_6*) δ 8.74 – 8.64 (m, 3H), 8.29 (d, *J* = 8.3 Hz, 1H), 8.03 – 7.96 (m, 2H), 7.95 – 7.89 (m, 2H), 7.81 – 7.75 (m, 2H), 7.25 – 7.14 (m, 3H), 7.13 – 7.07 (m, 1H), 5.32 (q, *J* = 8.0 Hz, 1H), 3.60 – 3.51 (m, 2H), 2.90 (ddd, *J* = 15.8, 8.7, 3.1 Hz, 1H), 2.78 (dt, *J* = 15.8, 8.3 Hz, 1H), 2.55 – 2.40 (m, 2H, partially buried under DMSO signal), 2.40 – 2.31 (m, 1H), 1.77 (dq, *J* = 12.6, 8.8 Hz, 1H).
^{13}C NMR (101 MHz, DMSO-*d_6*) δ 170.2, 165.6, 150.4, 146.0, 144.1, 142.8, 139.6, 134.9, 128.0, 127.3, 126.7, 126.3, 124.4, 124.0, 121.3, 53.4, 36.3, 35.4, 32.9, 29.7.
HRMS (ESI-TOF) calculated for $C_{24}H_{24}N_3O_2$ $[M+H]^+$ = 386.1863, found = 386.1868.

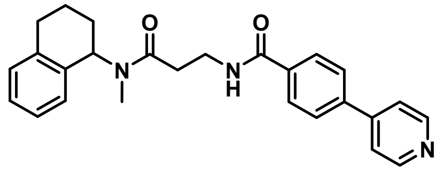

***N*-(3-(methyl(1,2,3,4-tetrahydronaphthalen-1-yl)amino)-3-oxopropyl)-4-(pyridin-4-yl)benzamide (10v):** Prepared in a similar manner as General Procedure A using 3-(4-(pyridin-4-yl)benzamido)propanoic acid hydrogen chloride (34.4 mg, 0.115 mmol, 1.0 eq.) and *N*-methyl-1,2,3,4-tetrahydronaphthalene-1-amine hydrochloride (24.1 mg, 0.122 mmol, 1.1 eq.) to afford the title compound as a yellow residue (10.4 mg, 25.1 µmol, 22% yield).
^{1}H NMR ($CDCl_3$): Note: a mixture of rotamers was observed. Variable NMR did not result in signal coalescence.
HRMS (ESI-TOF) calculated for $C_{26}H_{28}N_3O_2$ $[M+H]^+$ = 414.2176, found = 414.2180.

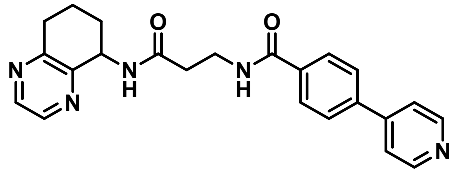

***N*-(3-oxo-3-((5,6,7,8-tetrahydroquinoxalin-5-yl)amino)propyl)-4-(pyridin-4-yl)benzamide (10w):** Prepared in a similar manner as General Procedure A using 3-(4-(pyridin-4-yl)benzamido)propanoic acid hydrogen chloride (24.0 mg, 78.2 µmol, 1.0 eq.) and 5,6,7,8-tetrahydroquinoxalin-5-amine hydrochloride (18.1 mg, 97.7 µmol, 1.2 eq.) to afford the title compound as a white residue (12.5 mg, 31.1 µmol, 40% yield).
^{1}H NMR (400 MHz, DMSO-*d_6*) δ 8.70 – 8.64 (m, 2H), 8.61 (t, *J* = 5.5 Hz, 1H), 8.46 – 8.39 (m, 2H), 8.34 (d, *J* = 8.2 Hz, 1H), 8.00 – 7.94 (m, 2H), 7.94 – 7.88 (m, 2H), 7.81 – 7.75 (m, 2H), 5.08 – 4.98 (m, 1H), 3.56 – 3.46 (m, 2H), 2.96 – 2.79 (m, 2H), 2.42 (t, *J* = 7.2 Hz, 2H), 2.03 – 1.76 (m, 4H).
^{13}C NMR (101 MHz, DMSO-*d_6*) δ 169.6, 165.6, 153.2, 151.8, 150.3, 146.0, 142.8, 142.2, 139.5, 135.0, 128.0, 126.7, 121.3, 48.5, 36.2, 35.4, 31.3, 29.2, 18.7.
HRMS (ESI-TOF) calculated for $C_{23}H_{24}N_5O_2$ $[M+H]^+$ = 402.1925, found = 402.1927.

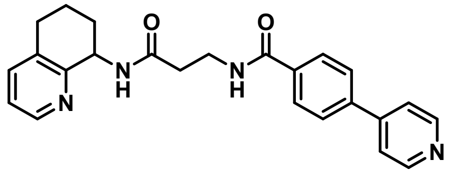

***N*-(3-oxo-3-((5,6,7,8-tetrahydroquinolin-8-yl)amino)propyl)-4-(pyridin-4-yl)benzamide (10x):** Prepared in a similar manner as General Procedure A using 3-(4-(pyridin-4-yl)benzamido)propanoic acid hydrogen chloride (24.0 mg, 78.2 µmol, 1.0 eq.) and 5,6,7,8-tetrahydroquinolin-8-amine (14.5 mg, 97.7 µmol, 1.2 eq.) to afford the title compound as a clear residue (17.6 mg, 43.9 µmol, 56% yield).
^{1}H NMR (400 MHz, MeOD-*d$_4$*) δ 8.69 – 8.55 (m, 2H), 8.34 – 8.27 (m, 1H), 8.00 – 7.92 (m, 2H), 7.89 – 7.81 (m, 2H), 7.80 – 7.72 (m, 2H), 7.60 – 7.54 (m, 1H), 7.23 (dd, *J* = 7.7, 4.7 Hz, 1H), 5.08 (t, *J* = 5.7 Hz, 1H), 3.79 – 3.65 (m, 2H), 2.92 – 2.73 (m, 2H), 2.57 (t, *J* = 6.6 Hz, 2H), 2.13 – 2.02 (m, 1H), 1.99 – 1.76 (m, 3H).
^{13}C NMR (101 MHz, MeOD-*d$_4$*) δ 173.2, 169.4, 156.1, 150.8, 149.5, 148.0, 141.8, 139.4, 136.5, 135.5, 129.3, 128.2, 124.1, 123.3, 50.8, 37.8, 36.9, 31.0, 29.4, 20.4.
HRMS (ESI-TOF) calculated for $C_{24}H_{25}N_4O_2$ [M+H]$^+$ = 401.1972, found = 401.1974.

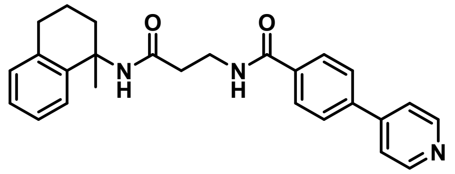

***N*-(3-((1-methyl-1,2,3,4-tetrahydronaphthalen-1-yl)amino)-3-oxopropyl)-4-(pyridin-4-yl)benzamide (10y):** Prepared in a similar manner as General Procedure A using 3-(4-(pyridin-4-yl)benzamido)propanoic acid hydrogen chloride (24.0 mg, 78.2 µmol, 1.0 eq.) and 1-methyl-1,2,3,4-tetrahydronaphthalen-1-amine (15.8 mg, 97.7 µmol, 1.2 eq.) to afford the title compound as a clear residue (12.4 mg, 30 µmol, 38% yield).
^{1}H NMR (400 MHz, DMSO-*d$_6$*) δ 8.69 – 8.64 (m, 2H), 8.52 (t, *J* = 5.5 Hz, 1H), 8.01 – 7.88 (m, 5H), 7.81 – 7.75 (m, 2H), 7.27 – 7.21 (m, 1H), 7.07 – 6.96 (m, 3H), 3.44 – 3.37 (m, 2H, partially buried under H_2O signal), 2.76 – 2.57 (m, 3H), 2.44 – 2.34 (m, 2H), 1.84 – 1.65 (m, 2H), 1.58 (ddd, *J* = 12.8, 5.7, 2.9 Hz, 1H), 1.44 (s, 3H).
^{13}C NMR (101 MHz, DMSO-*d$_6$*) δ 169.3, 165.6, 150.4, 146.1, 142.8, 139.6, 136.1, 135.0, 128.4, 128.0, 126.8, 125.9, 125.82, 125.77, 121.4, 55.0, 53.9, 36.3, 35.8, 34.2, 30.4, 29.4.
HRMS (ESI-TOF) calculated for $C_{26}H_{28}N_3O_2$ [M+H]$^+$ = 414.2176, found = 414.2176.

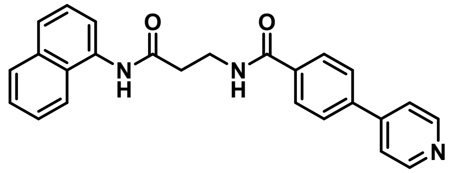

***N*-(3-(naphthalen-1-ylamino)-3-oxopropyl)-4-(pyridin-4-yl)benzamide (10z):** Prepared in a similar manner as General Procedure A using 3-(4-(pyridin-4-yl)benzamido)propanoic acid hydrogen chloride (24.0 mg, 78.2 µmol, 1.0 eq.) and 1-naphthylamine (14 mg, 97.7 µmol, 1.2 eq.) to afford the title compound as a white solid (7.4 mg, 18.7 µmol, 24% yield).
^{1}H NMR (400 MHz, DMSO-*d$_6$*) δ 9.99 (s, 1H), 8.79 (t, *J* = 5.2 Hz, 1H), 8.67 (d, *J* = 4.4 Hz, 2H), 8.07 – 7.99 (m, 3H), 7.96 – 7.89 (m, 3H), 7.81 – 7.73 (m, 3H), 7.68 (d, *J* = 7.4 Hz, 1H), 7.55 – 7.43 (m, 3H), 3.71 – 3.62 (m, 2H), 2.82 (t, *J* = 6.8 Hz, 2H).
^{13}C NMR (101 MHz, DMSO-*d$_6$*) δ 170.3, 165.8, 150.4, 146.1, 139.6, 135.0, 133.7, 133.6, 128.1, 127.9, 126.8, 126.0, 125.7, 125.6, 125.3, 122.9, 122.0, 121.4, 36.3, 36.0. Note: 1 aromatic carbon buried.
HRMS (ESI-TOF) calculated for $C_{25}H_{22}N_3O_2$ [M+H]$^+$ = 396.1707, found = 396.1702.

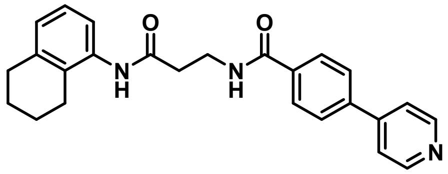

***N*-(3-oxo-3-((5,6,7,8-tetrahydronaphthalen-1-yl)amino)propyl)-4-(pyridin-4-yl)benzamide (10aa):** Prepared in a similar manner as General Procedure A using 3-(4-(pyridin-4-yl)benzamido)propanoic acid hydrogen chloride (24.0 mg, 78.2 µmol, 1.0 eq.) and 5,6,7,8-tetrahydro-1-naphthylamine (14.4 mg, 97.7 µmol, 1.2 eq.) to afford the title compound as a white solid (9.6 mg, 24 µmol, 31% yield).
^{1}H NMR (400 MHz, DMSO-*d_6*) δ 9.20 (s, 1H), 8.72 (t, *J* = 5.5 Hz, 1H), 8.69 – 8.64 (m, 2H), 8.03 – 7.96 (m, 2H), 7.94 – 7.89 (m, 2H), 7.79 – 7.75 (m, 2H), 7.16 (d, *J* = 7.8 Hz, 1H), 7.03 (t, *J* = 7.7 Hz, 1H), 6.89 (d, *J* = 7.5 Hz, 1H), 3.62 – 3.53 (m, 2H), 2.72 – 2.67 (m, 2H), 2.65 (t, *J* = 6.9 Hz, 2H), 2.53 (t, *J* = 5.9 Hz, 2H, partially buried under DMSO signal), 1.69 – 1.58 (m, 4H).
^{13}C NMR (101 MHz, DMSO-*d_6*) δ 169.5, 165.7, 150.4, 146.1, 139.6, 137.3, 136.0, 135.0, 131.2, 128.1, 126.8, 126.2, 125.0, 123.0, 121.4, 36.4, 35.8, 29.2, 24.3, 22.39, 22.35.
HRMS (ESI-TOF) calculated for $C_{25}H_{26}N_3O_2$ [M+H]$^+$ = 400.2020, found = 400.2018.

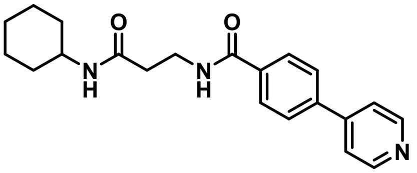

***N*-(3-(cyclohexylamino)-3-oxopropyl)-4-(pyridin-4-yl)benzamide (10ab):** Prepared in a similar manner as General Procedure A using 3-(4-(pyridin-4-yl)benzamido)propanoic acid hydrogen chloride (24.0 mg, 78.2 µmol, 1.0 eq.) and cyclohexylamine (11.2 µL, 97.7 µmol, 1.2 eq.) to afford the title compound as a white solid (5.1 mg, 14.5 µmol, 19% yield).
^{1}H NMR (400 MHz, DMSO-*d_6*) δ 8.68 – 8.64 (m, 2H), 8.61 (t, *J* = 5.6 Hz, 1H), 7.98 – 7.94 (m, 2H), 7.93 – 7.88 (m, 2H), 7.79 – 7.74 (m, 3H), 3.59 – 3.50 (m, 1H), 3.50 – 3.43 (m, 2H), 2.36 (t, *J* = 7.2 Hz, 2H), 1.76 – 1.61 (m, 4H), 1.58 – 1.49 (m, 1H), 1.30 – 1.17 (m, 2H), 1.17 – 1.05 (m, 3H).
^{13}C NMR (101 MHz, DMSO-*d_6*) δ 169.3, 165.6, 150.4, 146.0, 139.6, 135.0, 128.0, 126.8, 121.4, 47.4, 36.3, 35.4, 32.5, 25.2, 24.6.
HRMS (ESI-TOF) calculated for $C_{21}H_{26}N_3O_2$ [M+H]$^+$ = 352.2020, found = 352.2019.

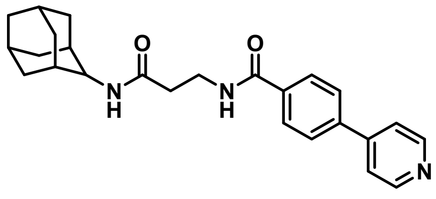

***N*-(3-(((1r,3r,5r,7r)-adamantan-2-yl)amino)-3-oxopropyl)-4-(pyridin-4-yl)benzamide (10ac):** Prepared in a similar manner as General Procedure A using 3-(4-(pyridin-4-yl)benzamido)propanoic acid hydrogen chloride and 2-adamantylamine hydrochloride (18.3 mg, 97.7 µmol, 1.2 eq.) to afford the title compound as a white residue (2.0 mg, 5.0 µmol, 6% yield).
^{1}H NMR (400 MHz, DMSO-*d_6*) δ 8.68 – 8.65 (m, 2H), 8.60 (t, *J* = 5.6 Hz, 1H), 7.98 – 7.94 (m, 2H), 7.93 – 7.88 (m, 2H), 7.80 – 7.75 (m, 3H), 3.90 – 3.84 (m, 1H), 3.52 – 3.44 (m, 2H), 2.50 – 2.44 (m, 2H, partially buried under DMSO signal), 2.00 – 1.92 (m, 2H), 1.83 – 1.64 (m, 10H), 1.48 – 1.41 (m, 2H).
HRMS (ESI-TOF) calculated for $C_{25}H_{30}N_3O_2$ [M+H]$^+$ = 404.2333, found = 404.2331.

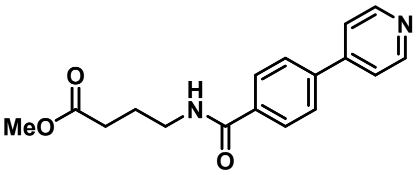

Methyl 4-(4-(pyridin-4-yl)benzamido)butanoate: To a vial was added HATU (136.3 mg, 0.359 mmol, 1.1 eq.), 4-pyridin-4-yl-benzoic acid (72.2 mg, 0.362 mmol, 1.1 eq.), anhydrous DMF (2 mL), and TEA (135.8 µL, 0.975 mmol, 3.0 eq.). The solution was stirred at room temperature for 30 minutes, then methyl 4-aminobutyrate hydrochloride (49.9 mg, 0.325 mmol, 1.0 eq.) was added. The reaction was stirred at room temperature overnight. Upon completion, the reaction was filtered and purified via RP-HPLC (0-60% MeCN in 0.5 mL/L AQ NH_4OH) to yield the title compound as a white solid (46.5 mg, 0.156 mmol, 48% yield).
^{1}H NMR (400 MHz, $CDCl_3$) δ 8.83 – 8.78 (m, 2H), 8.14 – 8.10 (m, 2H), 8.07 – 8.02 (m, 2H), 7.87 – 7.81 (m, 2H), 6.95 (t, *J* = 5.0 Hz, 1H), 3.70 (s, 3H), 3.60 – 3.54 (m, 2H), 2.52 (t, *J* = 6.7 Hz, 2H), 2.02 (quint, *J* = 6.6 Hz, 2H).
^{13}C NMR (101 MHz, $CDCl_3$) δ 174.9, 165.9, 156.9, 141.2, 137.9, 137.0, 128.7, 128.1, 124.5, 52.1, 40.4, 32.2, 24.1.
HRMS (ESI-TOF) calculated for $C_{17}H_{19}N_2O_3$ $[M+H]^+$ = 299.1390, found = 299.1390.

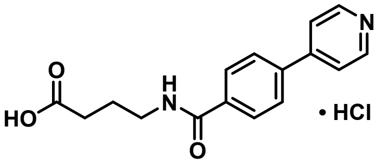

4-(4-(Pyridin-4-yl)benzamido)butanoic acid hydrogen chloride: Prepared according to General Procedure E using methyl 4-(4-(pyridin-4-yl)benzamido)butanoate (46.5 mg, 0.156 mmol, 1.0 eq.) to afford the title compound as a white solid in quantitative yield. Product taken forward without purification.
^{1}H NMR (400 MHz, DMSO-*d6*) δ 8.96 – 8.91 (m, 2H), 8.79 (t, *J* = 5.6 Hz, 1H), 8.36 – 8.30 (m, 2H), 8.12 – 8.04 (m, 4H), 3.34 - 3.27 (m, 2H), 2.29 (t, *J* = 7.4 Hz, 2H), 1.78 (quint, *J* = 7.2 Hz, 2H).
HRMS (ESI-TOF) calc. for $C_{16}H_{17}N_2O_3$ $[M+H]^+$ = 285.1234, found = 285.1230.

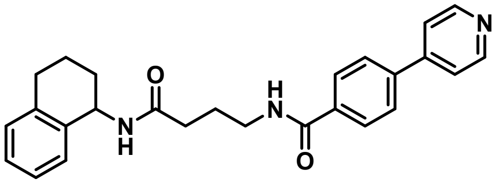

***N*-(4-oxo-4-((1,2,3,4-tetrahydronaphthalen-1-yl)amino)butyl)-4-(pyridin-4-yl)benzamide (11a):** Prepared in a similar manner as General Procedure A using 4-(4-(pyridin-4-yl)benzamido)butanoic acid hydrogen chloride (27.1 mg, 84.5 µmol, 1.2 eq.) and 1,2,3,4-tetrahydro-1-naphthylamine (10 µL, 69.7 µmol, 1.0 eq.) to afford the title compound as a white solid (8.4 mg, 20.3 µmol, 29% yield).
^{1}H NMR (400 MHz, DMSO-d_6) δ 8.69 - 8.64 (m, 2H), 8.60 (t, *J* = 5.5 Hz, 1H), 8.23 (d, *J* = 8.6 Hz, 1H), 8.02 – 7.96 (m, 2H), 7.94 – 7.88 (m, 2H), 7.79 – 7.75 (m, 2H), 7.18 – 7.05 (m, 4H), 5.02 – 4.94 (m, 1H), 3.36 - 3.27 (m, 2H, buried under H_2O signal), 2.80 - 2.64 (m, 2H), 2.28 - 2.13 (m, 2H), 1.91 - 1.77 (m, 4H), 1.76 - 1.60 (m, 2H). Note: Multiplet at 3.36 - 3.27 ppm validated by COSY.
^{13}C NMR (101 MHz, DMSO-d_6) δ 171.3, 165.5, 150.3, 146.1, 139.5, 137.6, 137.0, 135.1, 128.7, 128.2, 128.0, 126.7, 126.6, 125.8, 121.3, 46.2, 39.1, 33.1, 29.9, 28.8, 25.5, 20.0. Note: Peak at 39.1 ppm validated by HSQC.
HRMS (ESI-TOF) calculated for $C_{26}H_{28}N_3O_2$ $[M+H]^+$ = 414.2176, found = 414.2174.

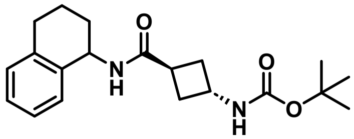

***Tert*-butyl (*trans*-3-((1,2,3,4-tetrahydronaphthalen-1-yl)carbamoyl)cyclobutyl)carbamate:** Prepared in a similar manner as General Procedure B using 1,2,3,4-tetrahydro-1-naphthylamine (73.1 µL, 0.509 mmol, 1.0 eq.) and *trans*-3-(*tert*-butoxycarbonylamino)cyclobutanecarboxylic acid (121.3 mg, 0.564 mmol, 1.1 eq.) to afford the title compound as a white solid (112.2 mg, 0.326 mmol, 64% yield).
^{1}H NMR (400 MHz, DMSO-*d$_6$*) δ 8.08 (d, *J* = 8.7 Hz, 1H), 7.18 – 7.05 (m, 5H), 5.01 – 4.93 (m, 1H), 4.24 – 4.10 (m, 1H), 2.87 – 2.63 (m, 3H), 2.37 – 2.24 (m, 2H), 2.15 – 2.00 (m, 2H), 1.91 – 1.79 (m, 2H), 1.76 – 1.57 (m, 2H), 1.37 (s, 9H).
^{13}C NMR (101 MHz, DMSO-*d$_6$*) δ 174.0, 154.5, 137.8, 137.0, 128.7, 128.1, 126.6, 125.9, 77.6, 46.2, 43.5, 33.2, 33.0, 32.6, 29.9, 28.8, 28.3, 20.0.
HRMS (ESI-TOF) calculated for $C_{20}H_{29}N_2O_3$ $[M+H]^+$ = 345.2173, found = 345.2178.

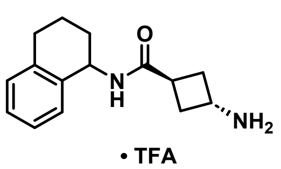

***Trans*-3-amino-*N*-(1,2,3,4-tetrahydronaphthalen-1-yl)cyclobutane-1-carboxamide trifluoroacetic acid:** Prepared according to General Procedure C using *tert*-butyl (*trans*-3-((1,2,3,4-tetrahydronaphthalen-1-yl)carbamoyl)cyclobutyl)carbamate (106.4 mg, 0.309 mmol, 1.0 eq.) to afford the title compound as a yellow solid in quantitative yield. Product taken forward without purification.
^{1}H NMR (400 MHz, DMSO-*d$_6$*) δ 8.23 (d, *J* = 8.7 Hz, 1H), 7.95 (s, 3H), 7.18 – 7.06 (m, 4H), 5.01 - 4.94 (m, 1H), 3.91 – 3.79 (m, 1H), 3.09 - 3.00 (m, 1H), 2.80 – 2.64 (m, 2H), 2.46 – 2.34 (m, 2H), 2.34 – 2.19 (m, 2H), 1.92 – 1.79 (m, 2H), 1.78 – 1.58 (m, 2H).
HRMS (ESI-TOF) calculated for $C_{15}H_{21}N_2O$ $[M+H]^+$ = 245.1648, found = 245.1654.

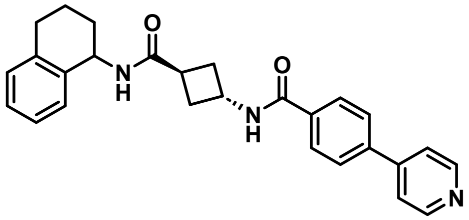

4-(Pyridin-4-yl)-*N*-(*trans*-3-((1,2,3,4-tetrahydronaphthalen-1-yl)carbamoyl)cyclobutyl)benzamide (11b): Prepared according to General Procedure D using *trans*-3-amino-*N*-(1,2,3,4-tetrahydronaphthalen-1-yl)cyclobutane-1-carboxamide trifluoroacetic acid (36.9 mg, 0.103 mmol, 1.0 eq.) and 4-pyridin-4-yl-benzoic acid (24.6 mg, 0.124 mmol, 1.2 eq.) to afford the title compound as a white solid (20.6 mg, 48.4 µmol, 47% yield).
^{1}H NMR (400 MHz, DMSO-*d$_6$*) δ 8.76 (d, *J* = 7.5 Hz, 1H), 8.69 – 8.64 (m, 2H), 8.15 (d, *J* = 8.7 Hz, 1H), 8.03 – 7.97 (m, 2H), 7.94 – 7.89 (m, 2H), 7.80 – 7.76 (m, 2H), 7.18 - 7.12 (m, 3H), 7.12 – 7.06 (m, 1H), 5.06 – 4.97 (m, 1H), 4.68 (sextet, *J* = 7.9 Hz, 1H), 3.02 – 2.91 (m, 1H), 2.81 – 2.64 (m, 2H), 2.51 – 2.40 (m, 2H), 2.40 – 2.25 (m, 2H), 1.94 – 1.81 (m, 2H), 1.80 – 1.60 (m, 2H).
^{13}C NMR (101 MHz, DMSO-*d$_6$*) δ 174.0, 164.8, 150.3, 146.0, 139.6, 137.8, 137.0, 134.9, 128.7, 128.12, 128.08, 126.7, 126.6, 125.9, 121.3, 46.2, 43.0, 32.9, 32.9, 32.6, 29.9, 28.8, 20.0.
HRMS (ESI-TOF) calculated for $C_{27}H_{28}N_3O_2$ $[M+H]^+$ = 426.2176, found = 426.2173.

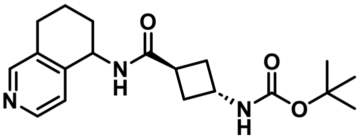

***Tert*-butyl (*trans*-3-((5,6,7,8-tetrahydroisoquinolin-5-yl)carbamoyl)cyclobutyl)carbamate:** Prepared in a similar manner as General Procedure B using 5,6,7,8-tetrahydro-5-isoquinolinamine hydrochloride (41.6 mg, 0.225 mmol, 1.0 eq.) and trans-3-(tert-butoxycarbonylamino)cyclobutanecarboxylic acid (52.0 mg, 0.242 mmol, 1.1 eq.) to afford the title compound as a white solid (33.1 mg, 95.8 µmol, 43% yield).

^{1}H NMR (400 MHz, CDCl$_3$) δ 8.37 – 8.33 (m, 2H), 7.13 (d, *J* = 5.2 Hz, 1H), 5.57 (d, *J* = 8.9 Hz, 1H), 5.24 – 5.15 (m, 1H), 4.74 (br. s, 1H), 4.37 – 4.24 (m, 1H), 2.96 – 2.87 (m, 1H), 2.77 (t, *J* = 6.3 Hz, 2H), 2.71 – 2.60 (m, 2H), 2.31 – 2.16 (m, 2H), 2.16 – 2.07 (m, 1H), 1.95 - 1.80 (m, 2H), 1.75 – 1.64 (m, 1H), 1.44 (s, 9H).
^{13}C NMR (101 MHz, CDCl$_3$) δ 174.5, 155.1, 150.6, 147.5, 146.0, 133.0, 122.4, 79.6, 46.8, 44.4, 34.4, 33.8, 33.7, 30.0, 28.5, 26.1, 20.4. Note: appearance of two peaks at 33.8 and 33.7 could be from conformational equilibrium.
HRMS (ESI-TOF) calculated for $C_{19}H_{28}N_3O_3$ [M+H]$^+$ = 346.2125, found = 346.2125.

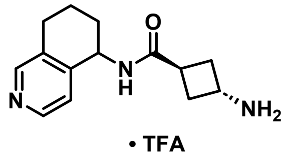

***Trans*-3-amino-*N*-(5,6,7,8-tetrahydroisoquinolin-5-yl)cyclobutane-1-carboxamide:** Prepared according to General Procedure C using *tert*-butyl (*trans*-3-((5,6,7,8-tetrahydroisoquinolin-5-yl)carbamoyl)cyclobutyl)carbamate (33.1 mg, 95.8 μmol, 1.0 eq.) to afford the title compound in quantitative yield. Product taken forward without purification.
^{1}H NMR (400 MHz, DMSO-*d$_6$*) δ 8.61 (s, 1H), 8.52 (d, *J* = 5.7 Hz, 1H), 8.46 (d, *J* = 8.3 Hz, 1H), 8.03-7.89 (m, 3H), 7.51 (d, *J* = 5.7 Hz, 1H), 5.07-4.99 (m, 1H), 3.88 – 3.78 (m, 1H), 3.14 – 3.05 (m, 1H), 2.91 – 2.76 (m, 2H), 2.50 – 2.35 (m, 1H), 2.35 – 2.24 (m, 2H), 2.02 – 1.87 (m, 2H), 1.87 – 1.76 (m, 1H), 1.75 – 1.64 (m, 1H).
HRMS (ESI-TOF) calculated for $C_{14}H_{20}N_3O$ [M+H]$^+$ = 246.1601, found = 246.1606.

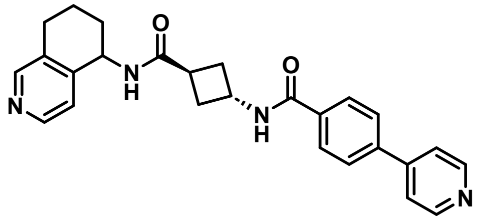

4-(Pyridin-4-yl)-*N*-(*trans*-3-((5,6,7,8-tetrahydroisoquinolin-5-yl)carbamoyl)cyclobutyl)benzamide (11c): Prepared in a similar manner as General Procedure D using *trans*-3-amino-*N*-(5,6,7,8-tetrahydroisoquinolin-5-yl)cyclobutane-1-carboxamide (34.4 mg, 95.7 μmol, 1.0 eq.) and 4-pyridin-4-yl-benzoic acid (28.6 mg, 0.144 mmol, 1.5 eq.) to afford the title compound as a white solid (4.5 mg, 10.6 μmol, 11% yield).
^{1}H NMR (400 MHz, MeOD-*d$_4$*) δ 8.71 – 8.54 (m, 2H), 8.40 – 8.20 (m, 2H), 8.01 – 7.96 (m, 2H), 7.90 – 7.84 (m, 2H), 7.78 (d, *J* = 5.3 Hz, 2H), 7.27 (d, *J* = 3.5 Hz, 1H), 5.16 – 5.09 (m, 1H), 4.78 (quint, *J* = 7.9 Hz, 1H), 3.18 – 3.08 (m, 1H), 2.87 – 2.79 (m, 2H), 2.77 – 2.63 (m, 2H), 2.53 – 2.41 (m, 2H), 2.15 – 1.96 (m, 1H), 1.94 – 1.72 (m, 2H).
^{13}C NMR (101 MHz, MeOD-*d$_4$*) δ 177.6, 168.9, 150.8, 150.6, 149.6, 148.8, 147.3, 141.9, 136.4, 129.3, 128.3, 123.3, 48.1, 45.1, 35.3, 33.9, 33.8, 30.5, 27.0, 21.5. Note: 2 carbon peaks not observed in aromatic region.
HRMS (ESI-TOF) calculated for $C_{26}H_{27}N_4O_2$ [M+H]$^+$ = 427.2129, found = 427.2127.

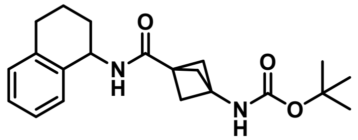

***Tert*-butyl (3-((1,2,3,4-tetrahydronaphthalen-1-yl)carbamoyl)bicyclo[1.1.1]pentan-1-yl)carbamate:** Prepared in a similar manner as General Procedure B using 1,2,3,4-tetrahydro-1-naphthylamine (48.7 μL, 0.340 mmol, 1.0 eq.) and 3-(*boc*-amino)bicyclo[1.1.1]pentane-1-carboxylic acid (115.6 mg, 0.509 mmol, 1.5 eq.) to afford the title compound as a yellow solid (99.1 mg, 0.278 mmol, 82% yield).
^{1}H NMR (400 MHz, CDCl$_3$) δ 7.25 – 7.14 (m, 3H), 7.13 – 7.09 (m, 1H), 5.68 (d, *J* = 8.5 Hz, 1H), 5.20-5.12 (m, 1H), 4.93 (s, 1H), 2.88 – 2.70 (m, 2H), 2.25 (s, 6H), 2.11 – 2.01 (m, 1H), 1.88 – 1.72 (m, 3H), 1.44 (s, 9H).
HRMS (ESI-TOF) calculated for $C_{21}H_{29}N_2O_3$ [M+H]$^+$ = 357.2173, found = 357.2177.

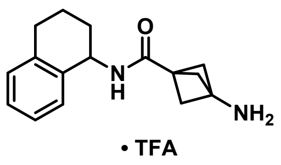

3-Amino-*N*-(1,2,3,4-tetrahydronaphthalen-1-yl)bicyclo[1.1.1]pentane-1-carboxamide trifluoroacetic acid: Prepared according to General Procedure C using *tert*-butyl (3-((1,2,3,4-tetrahydronaphthalen-1-yl)carbamoyl)bicyclo[1.1.1]pentan-1-yl)carbamate (97.8 mg, 0.274 mmol, 1.0 eq.) to afford the title compound as a red oil in quantitative yield. Product taken forward without purification.
^{1}H NMR (400 MHz, DMSO-*d₆*) δ 8.52 (s, 3H), 8.25 (d, *J* = 8.7 Hz, 1H), 7.19 – 7.04 (m, 4H), 5.01 – 4.93 (m, 1H), 2.81 – 2.66 (m, 2H), 2.19 (s, 6H), 1.93 – 1.81 (m, 2H), 1.76 – 1.59 (m, 2H).
HRMS (ESI-TOF) calculated for $C_{16}H_{21}N_2O_3$ $[M+H]^+$ = 257.1648, found = 257.1652.

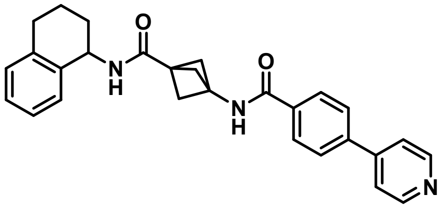

3-(4-(Pyridin-4-yl)benzamido)-*N*-(1,2,3,4-tetrahydronaphthalen-1-yl)bicyclo[1.1.1]pentane-1-carboxamide (11d): Prepared in a similar manner as General Procedure D using 3-amino-*N*-(1,2,3,4-tetrahydronaphthalen-1-yl)bicyclo[1.1.1]pentane-1-carboxamide trifluoroacetic acid (33.9 mg, 91.5 µmol, 1.0 eq.) and 4-pyridin-4-yl-benzoic acid (20.1 mg, 0.101 mmol, 1.1 eq.) to yield the title compound as a white solid (8.9 mg, 20.3 µmol, 22% yield).
^{1}H NMR (400 MHz, DMSO-d_6) δ 9.14 (s, 1H), 8.70 – 8.63 (m, 2H), 8.16 (d, *J* = 8.8 Hz, 1H), 8.01 – 7.95 (m, 2H), 7.93 – 7.87 (m, 2H), 7.80 – 7.74 (m, 2H), 7.19 – 7.06 (m, 4H), 5.04 – 4.96 (m, 1H), 2.82 – 2.66 (m, 2H), 2.31 (s, 6H), 1.97 – 1.82 (m, 2H), 1.77 – 1.64 (m, 2H).
^{13}C NMR (101 MHz, DMSO-d_6) δ 168.3, 166.0, 150.4, 146.0, 139.7, 137.7, 137.2, 134.7, 128.7, 128.1, 127.7, 126.7, 126.6, 125.9, 121.3, 53.5, 46.5, 45.4, 37.7, 29.8, 28.9, 20.5.
HRMS (ESI-TOF) calculated for $C_{28}H_{28}N_3O_2$ $[M+H]^+$ = 438.2176, found = 438.2176.

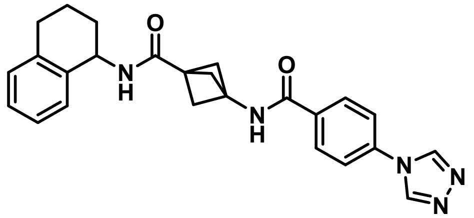

3-(4-(4*H*-1,2,4-triazol-4-yl)benzamido)-*N*-(1,2,3,4-tetrahydronaphthalen-1-yl)bicyclo[1.1.1]pentane-1-carboxamide (11e): Prepared in a similar manner as General Procedure D using 3-amino-N-(1,2,3,4-tetrahydronaphthalen-1-yl)bicyclo[1.1.1]pentane-1-carboxamide trifluoroacetic acid (33.9 mg, 91.5 µmol, 1.0 eq.) and 4-(4*H*-1,2,4-triazol-4-yl)benzoic acid (19.0 mg, 0.101 mmol, 1.1 eq.) to afford the title compound as a white solid (11.1 mg, 26.0 µmol, 28% yield).
^{1}H NMR (400 MHz, DMSO-d_6) δ 9.23 (s, 2H), 9.15 (s, 1H), 8.16 (d, *J* = 8.9 Hz, 1H), 8.04 – 7.98 (m, 2H), 7.86 – 7.80 (m, 2H), 7.19 – 7.06 (m, 4H), 5.04 – 4.95 (m, 1H), 2.82 – 2.64 (m, 2H), 2.30 (s, 6H), 1.98 – 1.82 (m, 2H), 1.77 – 1.63 (m, 2H).
^{13}C NMR (101 MHz, DMSO-d_6) δ 168.2, 165.4, 141.2, 137.7, 137.2, 136.1, 133.3, 129.0, 128.7, 127.6, 126.6, 125.9, 120.5, 53.5, 46.5, 45.3, 37.6, 29.8, 28.9, 20.5.
HRMS (ESI-TOF) calculated for $C_{25}H_{26}N_5O_2$ $[M+H]^+$ = 428.2081, found = 428.2082.

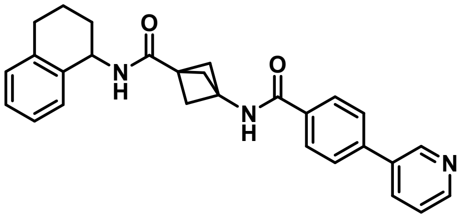

3-(4-(Pyridin-3-yl)benzamido)-*N*-(1,2,3,4-tetrahydronaphthalen-1-yl)bicyclo[1.1.1]pentane-1-carboxamide (11f): Prepared in a similar manner as General Procedure D using 3-amino-N-(1,2,3,4-tetrahydronaphthalen-1-yl)bicyclo[1.1.1]pentane-1-carboxamide trifluoroacetic acid (33.9 mg, 91.5 μmol, 1.0 eq.) and 4-pyridin-3-yl-benzoic acid (20.0 mg, 0.101 mmol, 1.1 eq.) to afford the title compound as a white solid (6.4 mg, 14.6 μmol, 16% yield).
^{1}H NMR (400 MHz, DMSO-*d$_6$*) δ 9.11 (s, 1H), 8.96 (dd, *J* = 2.4, 0.6 Hz, 1H), 8.60 (dd, *J* = 4.7, 1.6 Hz, 1H), 8.18 – 8.12 (m, 2H), 8.00 – 7.94 (m, 2H), 7.86 – 7.81 (m, 2H), 7.51 (ddd, *J* = 8.0, 4.8, 0.7 Hz, 1H), 7.19 – 7.05 (m, 4H), 5.04 – 4.95 (m, 1H), 2.82 – 2.65 (m, 2H), 2.30 (s, 6H), 1.97 – 1.83 (m, 2H), 1.77 – 1.64 (m, 2H).
^{13}C NMR (101 MHz, DMSO-*d$_6$*) δ 168.3, 166.1, 149.1, 147.8, 139.7, 137.7, 137.2, 134.6, 134.3, 133.7, 128.7, 128.1, 127.6, 126.7, 126.6, 125.9, 123.9, 53.5, 46.5, 45.4, 37.7, 29.8, 28.9, 20.5.
HRMS (ESI-TOF) calculated for $C_{28}H_{28}N_3O_2$ [M+H]$^+$ = 438.2176, found = 438.2175.

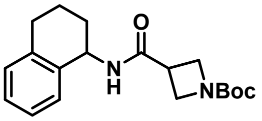

***Tert*-butyl 3-((1,2,3,4-tetrahydronaphthalen-1-yl)carbamoyl)azetidine-1-carboxylate:** Prepared in a similar manner as General Procedure B using 1,2,3,4-tetrahydro-1-naphthylamine (73.1 μL, 0.509 mmol, 1.0 eq.) and 1-(*tert*-butoxycarbonyl)azetidine-3-carboxylic acid (113.9 mg, 0.566 mmol, 1.1 eq.) to afford the title compound as an off-white solid (135.8 mg, 0.411 mmol, 81% yield).
^{1}H NMR (400 MHz, CDCl$_3$) δ 7.18 – 7.06 (m, 3H), 7.06 – 7.00 (m, 1H), 6.37 (d, *J* = 8.5 Hz, 1H), 5.14 – 5.06 (m, 1H), 4.14 – 3.93 (m, 2H), 3.92 (*t*, J = 8.3 Hz, 2H), 3.15 (tt, *J* = 8.7, 6.1 Hz, 1H), 2.80 – 2.65 (m, 2H), 2.03 – 1.90 (m, 1H), 1.84 – 1.69 (m, 3H), 1.38 (s, 9H).
^{13}C NMR (101 MHz, CDCl$_3$) δ 171.0, 156.1, 137.5, 136.4, 129.2, 128.5, 127.3, 126.2, 79.7, 51.7, 47.5, 33.2, 30.0, 29.2, 28.3, 20.0.
HRMS (ESI-TOF) calculated for C19H27N2O3 [M+H]+ = 331.2016, found = 331.2013.

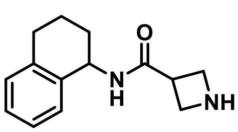

• TFA

***N*-(1,2,3,4-tetrahydronaphthalen-1-yl)azetidine-3-carboxamide trifluoroacetic acid:** Prepared according to General Procedure C using *tert*-butyl 3-((1,2,3,4-tetrahydronaphthalen-1-yl)carbamoyl)azetidine-1-carboxylate (135.8 mg, 0.411 mmol, 1.0 eq.) to afford the title compound as a red solid in quantitative yield. Product taken forward without purification.
^{1}H NMR (400 MHz, DMSO-*d$_6$*) δ 8.74 (s, 2H), 8.47 (d, *J* = 8.5 Hz, 1H), 7.20 – 7.07 (m, 4H), 5.03 – 4.95 (m, 1H), 4.09 – 3.99 (m, 4H), 3.56 (quintet, *J* = 8.2 Hz, 1H), 2.80 – 2.65 (m, 2H), 1.94 – 1.78 (m, 2H), 1.77 – 1.62 (m, 2H).
HRMS (ESI-TOF) calculated for C14H20N2O [M+H]+ = 231.1492, found = 231.1490.

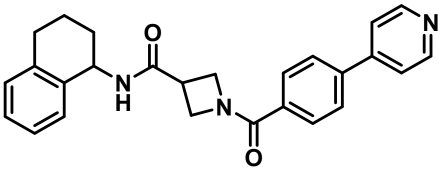

1-(4-(Pyridin-4-yl)benzoyl)-*N*-(1,2,3,4-tetrahydronaphthalen-1-yl)azetidine-3-carboxamide (11g): Prepared in a similar manner as General Procedure D using *N*-(1,2,3,4-tetrahydronaphthalen-1-yl)azetidine-3-carboxamide trifluoroacetic acid (35.4 mg, 0.103 mmol, 1.0 eq.) and 4-pyridin-4-yl-benzoic acid (27.9 mg, 0.140 mmol, 1.4 eq.) to afford the desired product as a clear residue (15.0 mg, 36.5 μmol, 35% yield).
^{1}H NMR (400 MHz, DMSO-*d$_6$*) δ 8.70 – 8.65 (m, 2H), 8.43 (d, *J* = 8.6 Hz, 1H), 7.93 – 7.87 (m, 2H), 7.82 – 7.73 (m, 4H), 7.20 – 7.06 (m, 4H), 5.05 – 4.96 (m, 1H), 4.53 – 4.34 (m, 2H), 4.25 – 4.07 (m, 2H), 3.49 – 3.40 (m, 1H), 2.80 – 2.64 (m, 2H), 1.94 – 1.79 (m, 2H), 1.76 – 1.62 (m, 2H).
^{13}C NMR (101 MHz, DMSO-*d$_6$*) δ 170.62, 170.58, 168.3, 150.4, 146.0, 139.5, 137.18, 137.15, 137.1, 133.4, 128.8, 128.6, 128.3, 127.0, 126.8, 125.95, 125.90, 121.4, 55.30, 55.22, 51.27, 51.22, 46.47, 46.43, 32.7, 29.8, 28.8, 19.8. Note: Peak pairs 170.62, 170.58; 137.18, 137.15; 125.95, 125.90; 55.30, 55.22; 51.27, 51.22; and 46.47, 46.43 appear as such due to slow conformational equilibrium at room temperature.
HRMS (ESI-TOF) calculated for $C_{26}H_{26}N_3O_2$ [M+H]$^+$ = 412.2020, found = 412.2022.

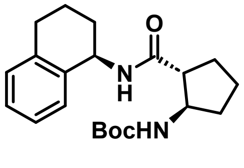

***Tert*-butyl ((1*R*,2*R*)-2-(((*R*)-1,2,3,4-tetrahydronaphthalen-1-yl)carbamoyl)cyclopentyl)carbamate:** Prepared in a similar manner as General Procedure B using (*R*)-(-)-1,2,3,4-tetrahydro-1-naphthylamine (53.3 μL, 0.363 mmol, 1.1 eq.) and (1*R*,2*R*)-2-((*tert*-butoxycarbonyl)amino)cyclopentanecarboxylic acid (74.5 mg, 0.325 mmol, 1.0 eq.) to afford the title compound as a white solid (102.1 mg, 0.285 mmol, 88% yield).
^{1}H NMR (400 MHz, CDCl$_3$) δ 7.53 – 7.36 (m, 2H), 7.30 – 7.22 (m, 2H), 7.22 – 7.16 (m, 1H), 5.35 – 5.25 (m, 1H), 5.03 – 4.89 (m, 1H), 4.14 (quint, *J* = 6.2 Hz, 1H), 3.00 – 2.81 (m, 2H), 2.80 – 2.67 (m, 1H), 2.24 – 2.09 (m, 3H), 2.09 – 1.74 (m, 6H), 1.64 – 1.50 (m, 1H), 1.52 (s, 9H).
^{13}C NMR (101 MHz, CDCl$_3$) δ 173.3, 156.0, 137.4, 129.0, 128.6, 126.9, 126.2, 79.7, 56.3, 52.9, 47.5, 33.4, 30.3, 29.4, 28.4, 28.1, 23.7, 20.3. Note: 1 aromatic C buried.
HRMS (ESI-TOF) calculated for $C_{21}H_{31}N_2O_3$ [M+H]$^+$ = 359.2329, found = 359.2334.

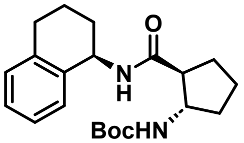

***Tert*-butyl ((1*S*,2*S*)-2-(((*R*)-1,2,3,4-tetrahydronaphthalen-1-yl)carbamoyl)cyclopentyl)carbamate:** Prepared in a similar manner as General Procedure B using (*R*)-(-)-1,2,3,4-tetrahydro-1-naphthylamine (53.3 μL, 0.363 mmol, 1.1 eq.) and (1*S*,2*S*)-2-((*tert*-butoxycarbonyl)amino)cyclopentanecarboxylic acid (75.9 mg, 0.331 mmol, 1.0 eq.) to afford the title compound as a white solid (105.3 mg, 0.294 mmol, 89% yield).
^{1}H NMR (400 MHz, CDCl$_3$) δ 7.57 – 7.47 (m, 1H), 7.43 – 7.36 (m, 1H), 7.30 – 7.17 (m, 3H), 5.36 – 5.28 (m, 1H), 5.14 – 5.04 (m, 1H), 4.11 (quint, *J* = 6.7 Hz, 1H), 3.01 – 2.83 (m, 2H), 2.78 – 2.66 (m, 1H), 2.27 – 1.90 (m, 7H), 1.90 – 1.74 (m, 2H), 1.64 – 1.50 (m, 1H), 1.47 (s, 9H).
^{13}C NMR (101 MHz, CDCl$_3$) δ 173.3, 156.0, 137.4, 129.0, 128.8, 126.9, 126.0, 79.6, 56.7, 52.7, 47.3, 33.5, 30.2, 29.3, 28.3, 27.6, 23.8, 20.1. Note: 1 aromatic C buried.
HRMS (ESI-TOF) calculated for $C_{21}H_{31}N_2O_3$ [M+H]$^+$ = 359.2329, found = 359.2335.

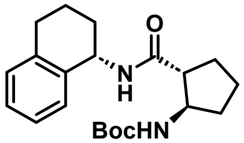

***Tert*-butyl ((1*R*,2*R*)-2-(((*S*)-1,2,3,4-tetrahydronaphthalen-1-yl)carbamoyl)cyclopentyl)carbamate:** Prepared in a similar manner as General Procedure B using (*S*)-(+)-1,2,3,4-tetrahydro-1-naphthylamine (52.4

µL, 0.360 mmol, 1.1 eq.) and (1*R*,2*R*)-2-((*tert*-butoxycarbonyl)amino)cyclopentanecarboxylic acid (75.2 mg, 0.328 mmol, 1.0 eq.) to afford the title compound as an off-white solid (86.6 mg, 0.242 mmol, 74% yield).
^{1}H NMR (400 MHz, $CDCl_3$) δ 7.37 – 7.21 (m, 2H), 7.16 – 7.03 (m, 3H), 5.22 – 5.14 (m, 1H), 4.84 – 4.72 (m, 1H), 3.97 (quint, J = 6.7 Hz, 1H), 2.88 – 2.68 (m, 2H), 2.65 – 2.51 (m, 1H), 2.16 – 1.60 (m, 9H), 1.48 – 1.37 (m, 1H), 1.33 (s, 9H).
^{13}C NMR (101 MHz, $CDCl_3$) δ 173.3, 156.0, 137.4, 129.0, 128.8, 127.0, 126.1, 79.8, 56.8, 52.9, 47.3, 33.5, 30.2, 29.3, 28.4, 27.6, 24.0, 20.1. Note: 1 aromatic signal buried.
HRMS (ESI-TOF) calculated for $C_{21}H_{31}N_2O_3$ [M+H]$^+$ = 359.2329, found = 359.2332.

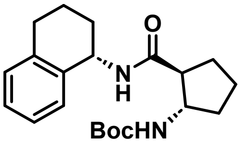

***Tert*-butyl ((1*S*,2*S*)-2-(((*S*)-1,2,3,4-tetrahydronaphthalen-1-yl)carbamoyl)cyclopentyl)carbamate:** Prepared in a similar manner as General Procedure B using (*S*)-(+)-1,2,3,4-tetrahydro-1-naphthylamine (52.4 µL, 0.360 mmol, 1.1 eq.) and (1*S*,2*S*)-2-((*tert*-butoxycarbonyl)amino)cyclopentanecarboxylic acid (73.8 mg, 0.322 mmol, 1.0 eq.) to afford the title compound as a white solid (80.2 mg, 0.224 mmol, 70% yield).
^{1}H NMR (400 MHz, $CDCl_3$) δ 7.38 – 7.23 (m, 2H), 7.17 – 7.09 (m, 2H), 7.09 – 7.04 (m, 1H), 5.21 – 5.13 (m, 1H), 4.77 (d, J = 6.4 Hz, 1H), 4.00 (quint, J = 6.5 Hz, 1H), 2.86 – 2.69 (m, 2H), 2.67 – 2.55 (m, 1H), 2.11 - 2.97 (m, 3H), 1.97 – 1.61 (m, 6H), 1.50 – 1.39 (m, 1H), 1.39 (s, 9H).
^{13}C NMR (101 MHz, $CDCl_3$) δ 173.3, 156.0, 137.5, 129.0, 128.6, 127.0, 126.2, 79.8, 56.3, 53.0, 47.5, 33.4, 30.3, 29.4, 28.5, 28.1, 23.7, 20.3. Note: 1 aromatic signal buried.
HRMS (ESI-TOF) calculated for $C_{21}H_{31}N_2O_3$ [M+H]$^+$ = 359.2329, found = 359.2332.

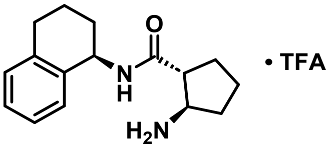

(1*R*,2*R*)-2-amino-*N*-((*R*)-1,2,3,4-tetrahydronaphthalen-1-yl)cyclopentane-1-carboxamide trifluoroacetic acid: Prepare according to General Procedure C using *tert*-butyl ((1*R*,2*R*)-2-(((*R*)-1,2,3,4-tetrahydronaphthalen-1-yl)carbamoyl)cyclopentyl)carbamate (102.1 mg, 0.285 mmol, 1.0 eq.) to afford the title compound as a clear residue in quantitative yield. Product taken forward without purification.
HRMS (ESI-TOF) calculated for $C_{16}H_{23}N_2O$ [M+H]$^+$ = 259.1805, found = 259.1808.

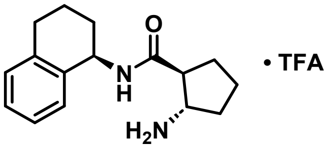

(1*S*,2*S*)-2-amino-*N*-((*R*)-1,2,3,4-tetrahydronaphthalen-1-yl)cyclopentane-1-carboxamide trifluoroacetic acid: Prepared according to General Procedure C using *tert*-butyl ((1*S*,2*S*)-2-(((*R*)-1,2,3,4-tetrahydronaphthalen-1-yl)carbamoyl)cyclopentyl)carbamate (105.3 mg, 0.294 mmol, 1.0 eq.) to afford the title compound as a pink solid in quantitative yield. Product taken forward without purification.
HRMS (ESI-TOF) calculated for $C_{16}H_{23}N_2O$ [M+H]$^+$ = 259.1805, found = 259.1806.

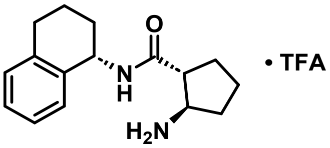

(1*R*,2*R*)-2-amino-*N*-((*S*)-1,2,3,4-tetrahydronaphthalen-1-yl)cyclopentane-1-carboxamide trifluoroacetic acid: Prepared according to General Procedure C using *tert*-butyl ((1*R*,2*R*)-2-(((*S*)-1,2,3,4-tetrahydronaphthalen-1-yl)carbamoyl)cyclopentyl)carbamate (86.6 mg, 0.242 mmol, 1.0 eq.) to afford the title compound as a red-gold oil in quantitative yield. Product taken forward without purification.
HRMS (ESI-TOF) calculated for $C_{16}H_{23}N_2O$ $[M+H]^+$ = 259.1805, found = 259.1808.

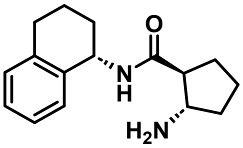

(1*S*,2*S*)-2-amino-*N*-((*S*)-1,2,3,4-tetrahydronaphthalen-1-yl)cyclopentane-1-carboxamide trifluoroacetic acid: Prepared according to General Procedure C using *tert*-butyl ((1*S*,2*S*)-2-(((*S*)-1,2,3,4-tetrahydronaphthalen-1-yl)carbamoyl)cyclopentyl)carbamate (80.2 mg, 0.224 mmol, 1.0 eq.) to afford the title compound as a red-gold oil in quantitative yield. Product taken forward without purification.
HRMS (ESI-TOF) calculated for $C_{16}H_{23}N_2O$ $[M+H]^+$ = 259.1805, found = 259.1805.

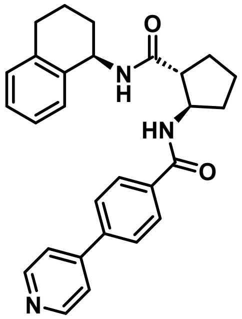

4-(Pyridin-4-yl)-*N*-((1*R*,2*R*)-2-(((*R*)-1,2,3,4-tetrahydronaphthalen-1-yl)carbamoyl)cyclopentyl)benzamide (11h): Prepared in a similar manner as General Procedure D using (1*R*,2*R*)-2-amino-N-((*R*)-1,2,3,4-tetrahydronaphthalen-1-yl)cyclopentane-1-carboxamide trifluoroacetic acid (53.0 mg, 0.142 mmol, 1.1 eq.) and 4-pyridin-4-yl-benzoic acid (25.8 mg, 0.130 mmol, 1.0 eq.) to afford the title compound as a white solid (22.8 mg, 51.9 µmol, 40% yield).
^{1}H NMR (400 MHz, DMSO-*d*$_6$) δ 8.71 – 8.64 (m, 2H), 8.42 (d, *J* = 8.0 Hz, 1H), 8.13 (d, *J* = 8.6 Hz, 1H), 8.01 – 7.95 (m, 2H), 7.93 – 7.87 (m, 2H), 7.81 – 7.75 (m, 2H), 7.09 (d, *J* = 7.7 Hz, 1H), 7.05 – 6.99 (m, 2H), 6.83 – 6.75 (m, 1H), 5.00 – 4.90 (m, 1H), 4.51 (quint, *J* = 7.9 Hz, 1H), 2.78 – 2.62 (m, 3H), 2.09 – 1.97 (m, 1H), 1.96 – 1.56 (m, 9H).
^{13}C NMR (101 MHz, DMSO-*d*$_6$) δ 173.1, 165.3, 150.4, 146.0, 139.5, 137.5, 136.9, 135.1, 128.5, 128.2, 128.1, 126.6, 126.5, 125.6, 121.3, 54.9, 50.5, 46.4, 32.6, 30.1, 28.8, 28.6, 23.4, 20.2.
HRMS (ESI-TOF) calculated for $C_{28}H_{30}N_3O_2$ $[M+H]^+$ = 440.2333, found = 440.2334.

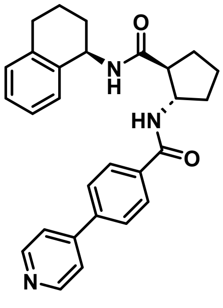

4-(Pyridin-4-yl)-*N*-((1*S*,2*S*)-2-(((*R*)-1,2,3,4-tetrahydronaphthalen-1-yl)carbamoyl)cyclopentyl)benzamide (11i): Prepared in a similar manner as General Procedure D using (1*S*,2*S*)-2-amino-*N*-((*R*)-1,2,3,4-tetrahydronaphthalen-1-yl)cyclopentane-1-carboxamide trifluoroacetic acid (54.7 mg, 0.147 mmol, 1.2 eq.) and

4-pyridin-4-yl-benzoic acid (25.1 mg, 0.126 mmol, 1.0 eq.) to afford the title compound as a light yellow solid (23.7 mg, 53.9 μmol, 43% yield).
^{1}H NMR (400 MHz, DMSO-*d$_6$*) δ 8.70 – 8.64 (m, 2H), 8.37 (d, *J* = 7.8 Hz, 1H), 8.12 (d, *J* = 8.7 Hz, 1H), 8.01 – 7.94 (m, 2H), 7.94 - 7.88 (m, 2H), 7.80 – 7.75 (m, 2H), 7.17 – 7.09 (m, 3H), 7.09 – 7.03 (m, 1H), 5.00 – 4.91 (m, 1H), 4.43 (quint, *J* = 7.7 Hz, 1H), 2.76 – 2.59 (m, 3H), 2.08 – 1.97 (m, 1H), 1.97 – 1.53 (m, 9H).
^{13}C NMR (101 MHz, DMSO-*d$_6$*) δ 172.8, 165.4, 150.3, 146.0, 139.5, 137.6, 137.0, 135.2, 128.7, 128.22, 128.16, 126.64, 126.59, 125.8, 121.3, 54.7, 50.3, 46.2, 32.7, 29.7, 28.8, 28.4, 23.3, 19.8.
HRMS (ESI-TOF) calculated for $C_{28}H_{30}N_3O_2$ $[M+H]^+$ = 440.2333, found = 440.2335.

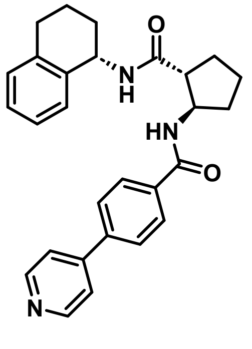

4-(Pyridin-4-yl)-*N*-((1*R*,2*R*)-2-(((*S*)-1,2,3,4-tetrahydronaphthalen-1-yl)carbamoyl)cyclopentyl)benzamide (11j): Prepared in a similar manner as General Procedure D using (1*R*,2*R*)-2-amino-*N*-((*S*)-1,2,3,4-tetrahydronaphthalen-1-yl)cyclopentane-1-carboxamide trifluoroacetic acid (45.0 mg, 0.121 mmol, 1.2 eq.) and 4-pyridin-4-yl-benzoic acid (19.8 mg, 99.4 μmol, 1.0 eq.) to afford the title compound as a light yellow solid (26.5 mg, 60.3 μmol, 61% yield).
^{1}H NMR (400 MHz, DMSO-*d$_6$*) δ 8.71 – 8.63 (m, 2H), 8.37 (d, *J* = 7.8 Hz, 1H), 8.12 (d, *J* = 8.6 Hz, 1H), 8.00 – 7.94 (m, 2H), 7.94 – 7.88 (m, 2H), 7.80 – 7.75 (m, 2H), 7.17 – 7.09 (m, 3H), 7.09 – 7.04 (m, 1H), 5.00 – 4.90 (m, 1H), 4.43 (quint, *J* = 7.7 Hz, 1H), 2.76 – 2.59 (m, 3H), 2.08 - 1.97 (m, 1H), 1.96 – 1.53 (m, 9H).
^{13}C NMR (101 MHz, DMSO-*d$_6$*) δ 172.8, 165.4, 150.3, 146.0, 139.5, 137.6, 137.0, 135.2, 128.7, 128.22, 128.16, 126.64, 126.59, 125.8, 121.3, 54.7, 50.3, 46.2, 32.7, 29.7, 28.8, 28.4, 23.3, 19.8.
HRMS (ESI-TOF) calculated for $C_{28}H_{30}N_3O_2$ $[M+H]^+$ = 440.2333, found = 440.2334.

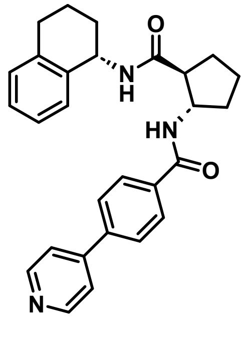

4-(Pyridin-4-yl)-*N*-((1*S*,2*S*)-2-(((*S*)-1,2,3,4-tetrahydronaphthalen-1-yl)carbamoyl)cyclopentyl)benzamide (11k): Prepared in a similar manner as General Procedure D using (1*S*,2*S*)-2-amino-*N*-((*S*)-1,2,3,4-tetrahydronaphthalen-1-yl)cyclopentane-1-carboxamide trifluoroacetic acid (41.7 mg, 0.112 mmol, 1.1 eq.) and 4-pyridin-4-yl-benzoic acid (20.2 mg, 0.101 mmol, 1.0 eq.) to afford the title compound as an off-white solid (18.5 mg, 42.1 μmol, 42% yield).
^{1}H NMR (400 MHz, DMSO-*d$_6$*) δ 8.70 – 8.64 (m, 2H), 8.42 (d, *J* = 8.0 Hz, 1H), 8.13 (d, *J* = 8.7 Hz, 1H), 8.02 – 7.94 (m, 2H), 7.93 – 7.86 (m, 2H), 7.81 – 7.74 (m, 2H), 7.09 (d, *J* = 7.7 Hz, 1H), 7.04 – 6.99 (m, 2H), 6.83 - 6.75 (m, 1H), 5.00 – 4.90 (m, 1H), 4.51 (quint, *J* = 7.8 Hz, 1H), 2.77 – 2.61 (m, 3H), 2.07 – 1.97 (m, 1H), 1.96 – 1.57 (m, 9H).
^{13}C NMR (101 MHz, DMSO-*d$_6$*) δ 173.1, 165.3, 150.3, 146.0, 139.5, 137.5, 136.9, 135.1, 128.5, 128.2, 128.1, 126.6, 126.5, 125.6, 121.3, 54.9, 50.5, 46.4, 32.6, 30.1, 28.8, 28.6, 23.4, 20.2.
HRMS (ESI-TOF) calculated for $C_{28}H_{30}N_3O_2$ $[M+H]^+$ = 440.2333, found = 440.2334.

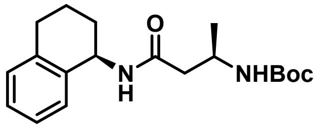

***Tert*-butyl ((*R*)-4-oxo-4-(((*R*)-1,2,3,4-tetrahydronaphthalen-1-yl)amino)butan-2-yl)carbamate:** Prepared in a similar manner as General Procedure B using (*R*)-(-)-1,2,3,4-tetrahydro-1-naphthylamine (53.3 µL, 0.363 mmol, 1.0 eq.) and (*R*)-3-((*tert*-butoxycarbonyl)amino)butanoic acid (71.0 mg, 0.349 mmol, 1.0 eq.) to afford the title compound as a white solid (107.4 mg, 0.323 mmol, 92% yield).
^{1}H NMR (400 MHz, $CDCl_3$) δ 7.23 – 7.17 (m, 1H), 7.16 – 7.09 (m, 2H), 7.08 – 7.03 (m, 1H), 6.29 (d, *J* = 8.3 Hz, 1H), 5.33 (s, 1H), 5.18 – 5.09 (m, 1H), 3.93 (quint, *J* = 6.0 Hz, 1H), 2.83 – 2.66 (m, 2H), 2.42 – 2.28 (m, 2H), 2.04 – 1.93 (m, 1H), 1.88 – 1.69 (m, 3H), 1.39 (s, 9H), 1.20 (d, *J* = 6.6 Hz, 3H).
^{13}C NMR (101 MHz, CDCl3) δ 170.2, 155.4, 137.6, 136.6, 129.2, 128.7, 127.3, 126.3, 79.2, 47.4, 44.3, 42.8, 30.2, 29.3, 28.5, 20.8, 20.1.
HRMS (ESI-TOF) calculated for $C_{19}H_{29}N_2O_3$ [M+H]$^+$ = 333.2173, found = 333.2175.

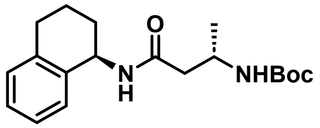

***Tert*-butyl ((*S*)-4-oxo-4-(((*R*)-1,2,3,4-tetrahydronaphthalen-1-yl)amino)butan-2-yl)carbamate:** Prepared in a similar manner as General Procedure B using (*R*)-(-)-1,2,3,4-tetrahydro-1-naphthylamine (53.3 µL, 0.363 mmol, 1.1 eq.) and (*S*)-3-((*tert*-butoxycarbonyl)amino)butanoic acid (66.9 mg, 0.329 mmol, 1.0 eq.) to afford the title compound as a white solid (95.8 mg, 0.288 mmol, 88% yield).
^{1}H NMR (400 MHz, $CDCl_3$) δ 7.25 – 7.19 (m, 1H), 7.18 – 7.10 (m, 2H), 7.10 – 7.04 (m, 1H), 6.19 – 6.07 (m, 1H), 5.39 – 5.17 (m, 1H), 5.21 – 5.10 (m, 1H), 4.01 – 3.88 (m, 1H), 2.85 – 2.68 (m, 2H), 2.46 – 2.29 (m, 2H), 2.08 – 1.94 (m, 1H), 1.88 – 1.74 (m, 3H), 1.38 (s, 9H), 1.22 (d, *J* = 6.6 Hz, 3H).
^{13}C NMR (101 MHz, $CDCl_3$) δ 170.2, 155.5, 137.7, 136.6, 129.3, 128.8, 127.4, 126.4, 79.4, 47.5, 44.5, 42.9, 30.3, 29.3, 28.5, 20.7, 20.0.
HRMS (ESI-TOF) calculated for $C_{19}H_{29}N_2O_3$ [M+H]$^+$ = 333.2173, found = 333.2176.

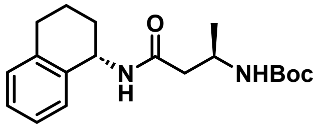

***Tert*-butyl ((*R*)-4-oxo-4-(((*S*)-1,2,3,4-tetrahydronaphthalen-1-yl)amino)butan-2-yl)carbamate:** Prepared in a similar manner as General Procedure B using (*S*)-(+)-1,2,3,4-tetrahydro-1-naphthylamine (59.2 µL, 0.406 mmol, 1.1 eq.) and (*R*)-3-((*tert*-butoxycarbonyl)amino)butanoic acid (76.6 mg, 0.377 mmol, 1.0 eq.) to afford the title compound as a pink solid (112.3 mg, 0.338 mmol, 90% yield).
^{1}H NMR (400 MHz, $CDCl_3$) δ 7.25 – 7.20 (m, 1H), 7.19 – 7.11 (m, 2H), 7.11 – 7.05 (m, 1H), 6.14 – 6.00 (m, 1H), 5.27 (s, 1H), 5.21 – 5.11 (m, 1H), 4.01 - 3.89 (m, 1H), 2.85 – 2.68 (m, 2H), 2.47 - 2.30 (m, 2H), 2.09 - 1.94 (m, 1H), 1.89 – 1.74 (m, 3H), 1.39 (s, 9H), 1.23 (d, *J* = 6.6 Hz, 3H).
^{13}C NMR (101 MHz, $CDCl_3$) δ 170.2, 155.5, 137.7, 136.6, 129.3, 128.8, 127.4, 126.4, 79.4, 47.5, 44.5, 42.9, 30.3, 29.3, 28.5, 20.7, 20.0.
HRMS (ESI-TOF) calculated for $C_{19}H_{29}N_2O_3$ [M+H]$^+$ = 333.2173, found = 333.2178.

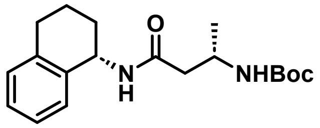

***Tert*-butyl ((*S*)-4-oxo-4-(((*S*)-1,2,3,4-tetrahydronaphthalen-1-yl)amino)butan-2-yl)carbamate:** Prepared in a similar manner as General Procedure B using (*S*)-(+)-1,2,3,4-tetrahydro-1-naphthylamine (59.2 μL, 0.406 mmol, 1.1 eq.) and (*S*)-3-((*tert*-butoxycarbonyl)amino)butanoic acid (77.3 mg, 0.380 mmol, 1.0 eq.) to afford the title compound as a pink solid (106.5 mg, 0.320 mmol, 84% yield).
^{1}H NMR (400 MHz, CDCl$_3$) δ 7.26 – 7.21 (m, 1H), 7.20 – 7.13 (m, 2H), 7.12 – 7.06 (m, 1H), 6.02 – 5.89 (m, 1H), 5.36 – 5.09 (m, 2H), 4.03 – 3.91 (m, 1H), 2.86 – 2.68 (m, 2H), 2.50 – 2.32 (m, 2H), 2.09 – 1.96 (m, 1H), 1.88 – 1.74 (m, 3H), 1.42 (s, 9H), 1.26 (d, *J* = 6.7 Hz, 3H).
^{13}C NMR (101 MHz, CDCl$_3$) δ 170.1, 155.5, 137.7, 136.6, 129.3, 128.8, 127.4, 126.5, 79.4, 47.5, 44.5, 43.0, 30.2, 29.3, 28.5, 20.8, 20.1.
HRMS (ESI-TOF) calculated for $C_{19}H_{29}N_2O_3$ [M+H]$^+$ = 333.2173, found = 333.2177.

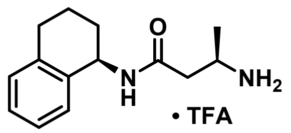

(*R*)-3-amino-*N*-((*R*)-1,2,3,4-tetrahydronaphthalen-1-yl)butanamide trifluoroacetic acid: Prepared according to General Procedure C using *tert*-butyl ((*R*)-4-oxo-4-(((*R*)-1,2,3,4-tetrahydronaphthalen-1-yl)amino)butan-2-yl)carbamate (107.4 mg, 0.323 mmol, 1.0 eq.) to afford the title compound as a clear residue in quantitative yield. Product taken forward without purification.
HRMS (ESI-TOF) calculated for $C_{14}H_{21}N_2O$ [M+H]$^+$ = 233.1648, found = 233.1648.

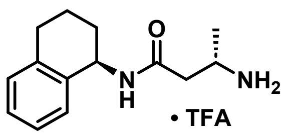

(*S*)-3-amino-*N*-((*R*)-1,2,3,4-tetrahydronaphthalen-1-yl)butanamide trifluoroacetic acid: Prepared according to General Procedure C using *tert*-butyl ((*S*)-4-oxo-4-(((*R*)-1,2,3,4-tetrahydronaphthalen-1-yl)amino)butan-2-yl)carbamate (95.8 mg, 0.288 mmol, 1.0 eq.) to afford the title compound as a yellow residue in quantitative yield. Product taken forward without purification.
HRMS (ESI-TOF) calculated for $C_{14}H_{21}N_2O$ [M+H]$^+$ = 233.1648, found = 233.1650.

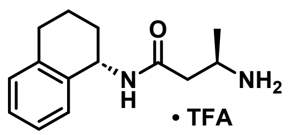

(*R*)-3-amino-*N*-((*S*)-1,2,3,4-tetrahydronaphthalen-1-yl)butanamide trifluoroacetic acid: Prepared according to General Procedure C using *tert*-butyl ((*R*)-4-oxo-4-(((*S*)-1,2,3,4-tetrahydronaphthalen-1-yl)amino)butan-2-yl)carbamate (112.3 mg, 0.338 mmol, 1.0 eq.) to afford the title compound as a red-gold oil in quantitative yield. Product taken forward without purification.
HRMS (ESI-TOF) calculated for $C_{14}H_{21}N_2O$ [M+H]$^+$ = 233.1648, found = 233.1649.

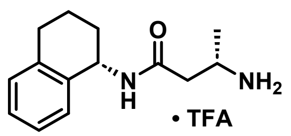

(*S*)-3-amino-*N*-((*S*)-1,2,3,4-tetrahydronaphthalen-1-yl)butanamide trifluoroacetic acid: Prepared according to General Procedure C using *tert*-butyl ((*S*)-4-oxo-4-(((*S*)-1,2,3,4-tetrahydronaphthalen-1-yl)amino)butan-2-yl)carbamate (106.5 mg, 0.320 mmol, 1.0 eq.) to afford the title compound as a red-gold oil in quantitative yield. Product taken forward without purification.
HRMS (ESI-TOF) calculated for $C_{14}H_{21}N_2O$ [M+H]$^+$ = 233.1648, found = 233.1651.

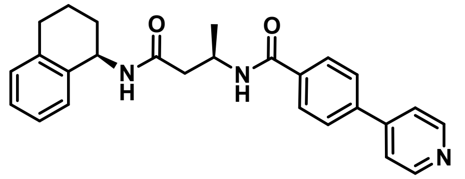

N-((R)-4-oxo-4-(((R)-1,2,3,4-tetrahydronaphthalen-1-yl)amino)butan-2-yl)-4-(pyridin-4-yl)benzamide (11l): Prepared in a similar manner as General Procedure D using (*R*)-3-amino-*N*-((*R*)-1,2,3,4-tetrahydronaphthalen-1-yl)butanamide trifluoroacetic acid (55.9 mg, 0.162 mmol, 1.2 eq.) and 4-pyridin-4-yl-benzoic acid (26.8 mg, 0.135 mmol, 1.0 eq.) to afford the title compound as a white solid (10.6 mg, 25.6 µmol, 19% yield).
^{1}H NMR (400 MHz, DMSO-*d6*) δ 8.70 – 8.65 (m, 2H), 8.42 (d, *J* = 8.1 Hz, 1H), 8.27 (d, *J* = 8.6 Hz, 1H), 8.00 – 7.94 (m, 2H), 7.94 – 7.88 (m, 2H), 7.81 – 7.74 (m, 2H), 7.13 – 7.04 (m, 3H), 7.00 – 6.93 (m, 1H), 5.02 – 4.93 (m, 1H), 4.49 – 4.36 (m, 1H), 2.81 – 2.62 (m, 2H), 2.56 – 2.45 (m, 1H, partially buried under DMSO signal), 2.35 (dd, *J* = 13.7, 7.2 Hz, 1H), 1.92 – 1.79 (m, 2H), 1.76 – 1.62 (m, 2H), 1.21 (d, *J* = 6.6 Hz, 3H).
13C NMR (101 MHz, DMSO-*d6*) δ 169.5, 164.7, 150.4, 146.1, 139.5, 137.4, 137.0, 135.2, 128.7, 128.2, 128.1, 126.7, 126.6, 125.7, 121.4, 46.2, 43.3, 42.1, 29.9, 28.8, 20.3, 19.9.
HRMS (ESI-TOF) calculated for $C_{26}H_{28}N_3O_2$ $[M+H]^+$ = 414.2176, found = 414.2178.

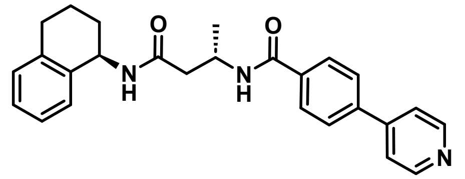

N-((S)-4-oxo-4-(((R)-1,2,3,4-tetrahydronaphthalen-1-yl)amino)butan-2-yl)-4-(pyridin-4-yl)benzamide (11m): Prepared in a similar manner as General Procedure D using (*S*)-3-amino-*N*-((*R*)-1,2,3,4-tetrahydronaphthalen-1-yl)butanamide trifluoroacetic acid (49.9 mg, 0.144 mmol, 1.2 eq.) and 4-pyridin-4-yl-benzoic acid (23.6 mg, 0.119 mmol, 1.0 eq.) to afford the title compound as a white solid (23.3 mg, 56.3 µmol, 48% yield).
^{1}H NMR (400 MHz, DMSO-*d6*) δ 8.70 – 8.64 (m, 2H), 8.41 (d, *J* = 8.0 Hz, 1H), 8.29 (d, *J* = 8.6 Hz, 1H), 8.01 – 7.95 (m, 2H), 7.95 – 7.89 (m, 2H), 7.80 – 7.75 (m, 2H), 7.19 – 7.05 (m, 4H), 5.03 – 4.93 (m, 1H), 4.49 – 4.37 (m, 1H), 2.78 - 2.62 (m, 2H), 2.46 (dd, *J* = 13.7, 6.5 Hz, 1H, partially buried under DMSO signal), 2.38 (dd, *J* = 13.7, 7.4 Hz, 1H), 1.90 – 1.76 (m, 2H), 1.73 – 1.58 (m, 2H), 1.21 (d, *J* = 6.6 Hz, 3H).
^{13}C NMR (101 MHz, DMSO-*d6*) δ 169.5, 164.7, 150.3, 146.1, 139.5, 137.5, 137.1, 135.2, 128.7, 128.3, 128.1, 126.7, 125.8, 121.3, 46.2, 43.3, 42.1, 29.9, 28.8, 20.4, 19.8. Note: 1 aromatic C signal buried.
HRMS (ESI-TOF) calculated for $C_{26}H_{28}N_3O_2$ $[M+H]^+$ = 414.2176, found = 414.2180.

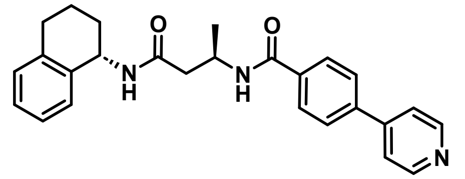

N-((R)-4-oxo-4-(((S)-1,2,3,4-tetrahydronaphthalen-1-yl)amino)butan-2-yl)-4-(pyridin-4-yl)benzamide (VU6080195, 11n): Prepared in a similar manner as General Procedure D using (*R*)-3-amino-*N*-((*S*)-1,2,3,4-tetrahydronaphthalen-1-yl)butanamide trifluoroacetic acid (58.5 mg, 0.169 mmol, 1.2 eq.) and 4-pyridin-4-yl-benzoic acid (27.8 mg, 0.140 mmol, 1.0 eq.) to afford the title compound as a white solid (22.1 mg, 53.4 µmol, 38% yield).
^{1}H NMR (400 MHz, DMSO-*d6*) δ 8.70 – 8.65 (m, 2H), 8.41 (d, *J* = 8.1 Hz, 1H), 8.29 (d, *J* = 8.6 Hz, 1H), 8.01 – 7.95 (m, 2H), 7.95 – 7.89 (m, 2H), 7.81 – 7.75 (m, 2H), 7.18 – 7.04 (m, 4H), 5.02 – 4.93 (m, 1H), 4.49 - 4.37 (m, 1H), 2.78 - 2.62 (m, 2H), 2.46 (dd, *J* = 13.7, 6.5 Hz, 1H), 2.37 (dd, *J* = 13.7, 7.4 Hz, 1H), 1.90 – 1.76 (m, 2H), 1.72 – 1.58 (m, 2H), 1.21 (d, *J* = 6.6 Hz, 3H).

^{13}C NMR (101 MHz, DMSO-*d$_6$*) δ 169.5, 164.8, 150.4, 146.1, 139.5, 137.5, 137.1, 135.2, 128.7, 128.3, 128.1, 126.7, 125.8, 121.4, 46.2, 43.3, 42.1, 29.9, 28.8, 20.4, 19.9. Note: 1 Aromatic C buried.
HRMS (ESI-TOF) calculated for $C_{26}H_{28}N_3O_2$ $[M+H]^+$ = 414.2176, found = 414.2180.

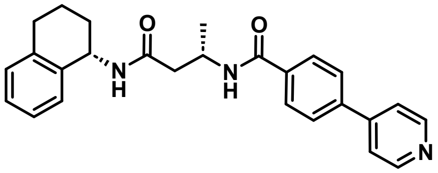

***N*-((*S*)-4-oxo-4-(((*S*)-1,2,3,4-tetrahydronaphthalen-1-yl)amino)butan-2-yl)-4-(pyridin-4-yl)benzamide (11o):** Prepared in a similar manner as General Procedure D using (*S*)-3-amino-*N*-((*S*)-1,2,3,4-tetrahydronaphthalen-1-yl)butanamide trifluoroacetic acid (55.5 mg, 0.160 mmol, 1.2 eq.) and 4-pyridin-4-yl-benzoic acid (26.7 mg, 0.134 mmol, 1.0 eq.) to afford the title compound as a white solid (22.7 mg, 54.9 μmol, 41% yield).
^{1}H NMR (400 MHz, DMSO-*d$_6$*) δ 8.72 – 8.62 (m, 2H), 8.42 (d, *J* = 8.0 Hz, 1H), 8.27 (d, *J* = 8.5 Hz, 1H), 8.00 – 7.94 (m, 2H), 7.94 – 7.87 (m, 2H), 7.80 – 7.74 (m, 2H), 7.13 – 7.02 (m, 3H), 7.00 - 6.94 (m, 1H), 5.02 – 4.93 (m, 1H), 4.49 - 4.35 (m, 1H), 2.80 – 2.62 (m, 2H), 2.55 – 2.44 (m, 1H, partially buried under DMSO signal), 2.35 (dd, *J* = 13.7, 7.3 Hz, 1H), 1.92 – 1.79 (m, 2H), 1.76 – 1.61 (m, 2H), 1.21 (d, *J* = 6.6 Hz, 3H).
^{13}C NMR (101 MHz, DMSO-*d$_6$*) δ 169.5, 164.7, 150.3, 146.1, 139.5, 137.4, 137.0, 135.2, 128.7, 128.2, 128.1, 126.7, 126.6, 125.7, 121.4, 46.2, 43.3, 42.1, 29.9, 28.8, 20.3, 19.9.
HRMS (ESI-TOF) calculated for $C_{26}H_{28}N_3O_2$ $[M+H]^+$ = 414.2176, found = 414.2181.

NMR Spectra of Final Compounds

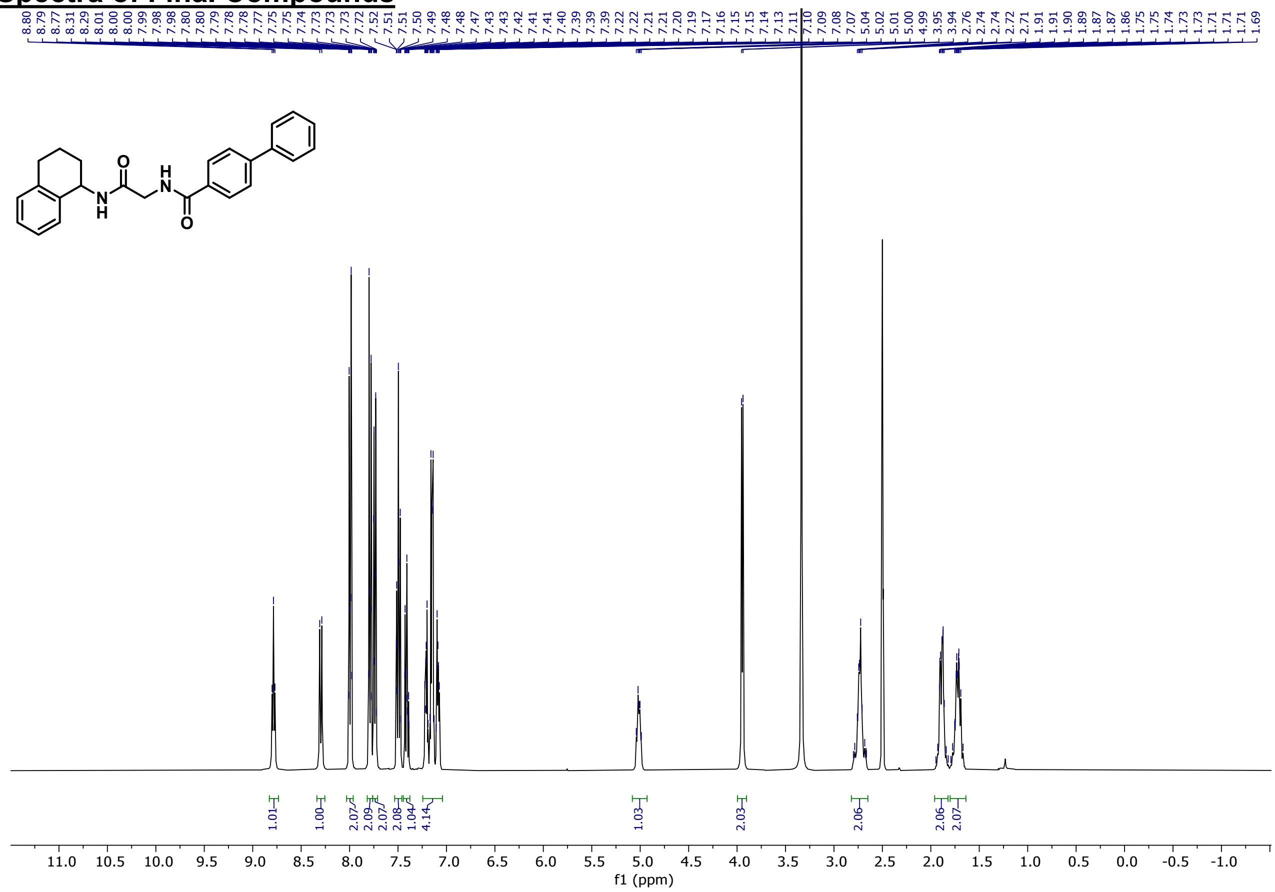

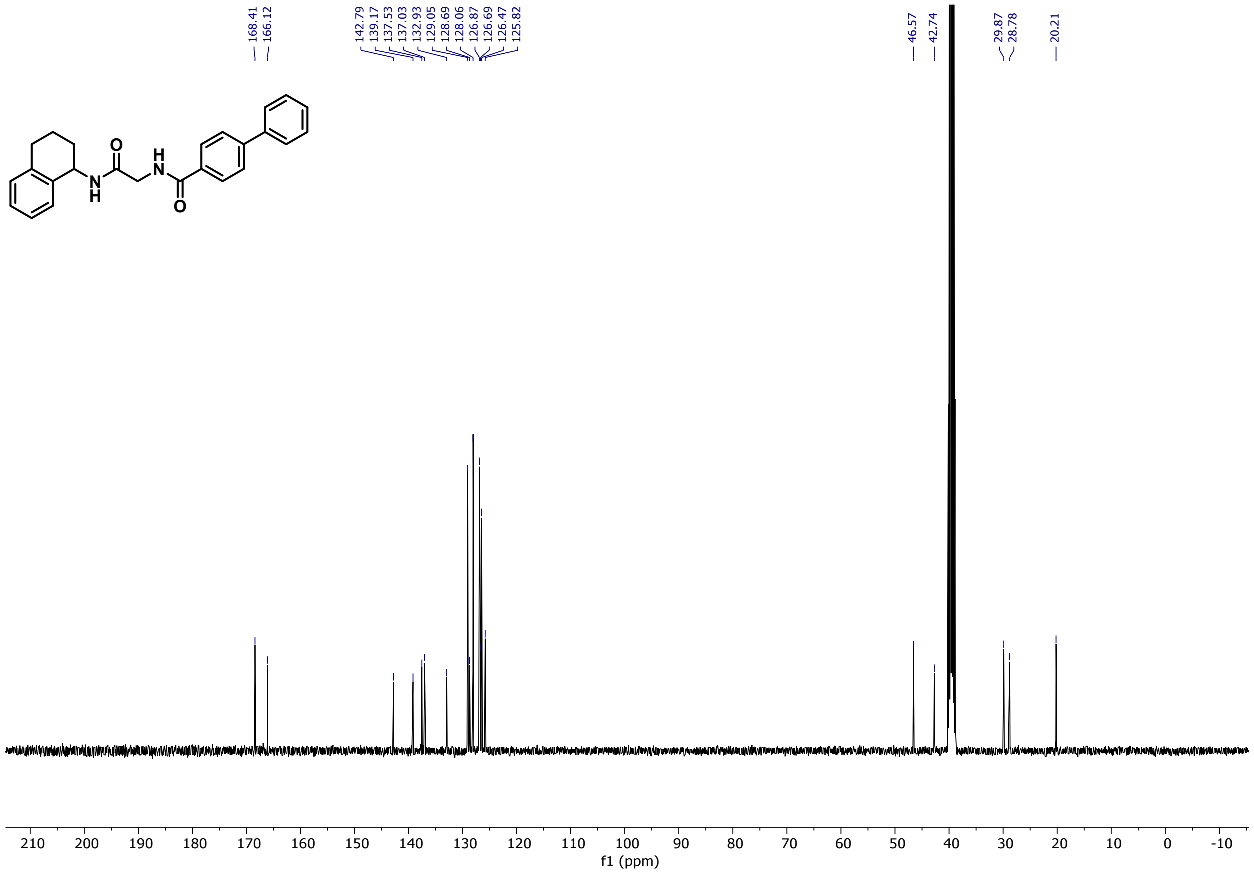

NMR spectra of Compound 43 (VU6063661, 1) in DMSO-*d*$_6$.

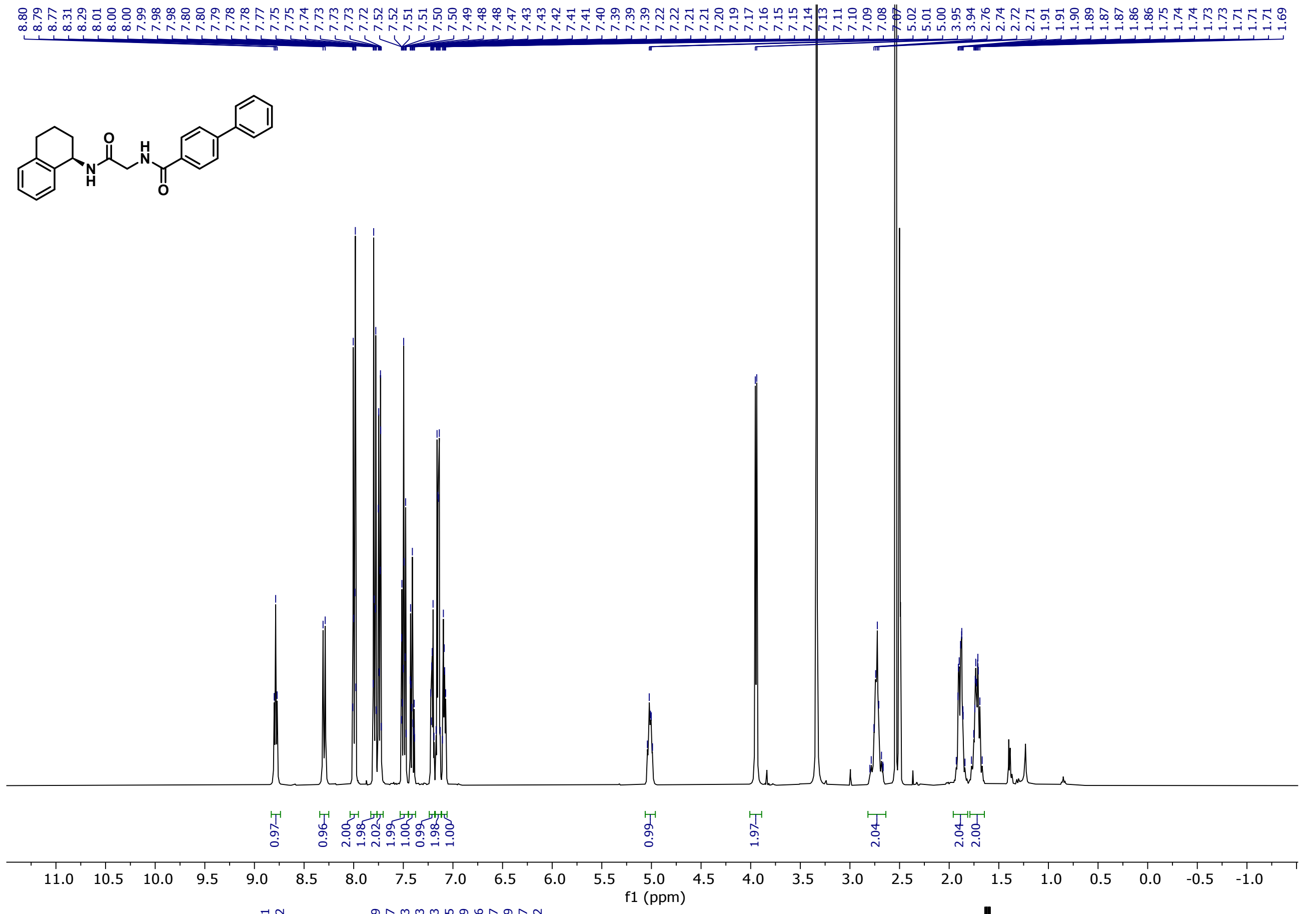

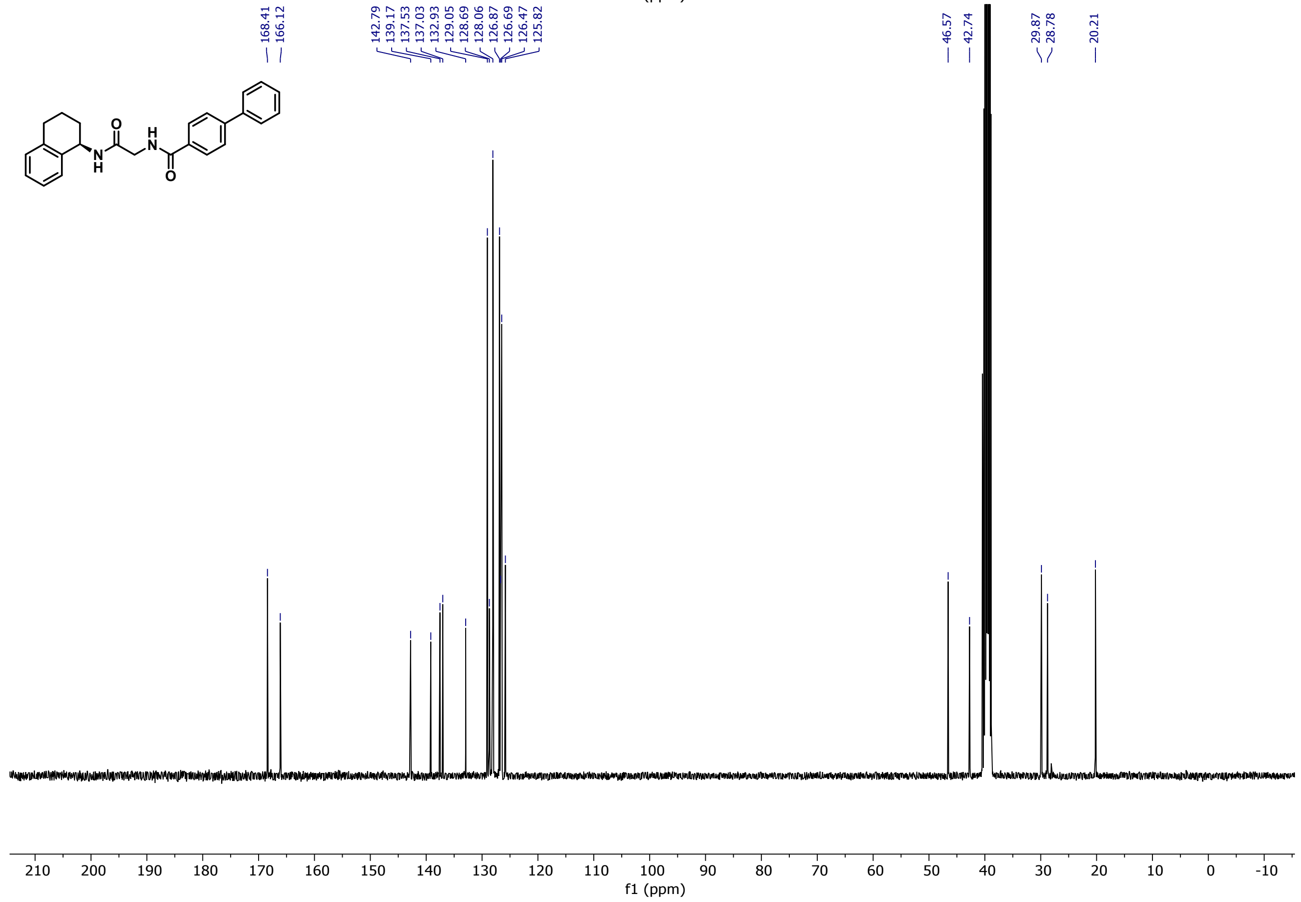

NMR spectra of (*R*)-1 in DMSO-d_6.

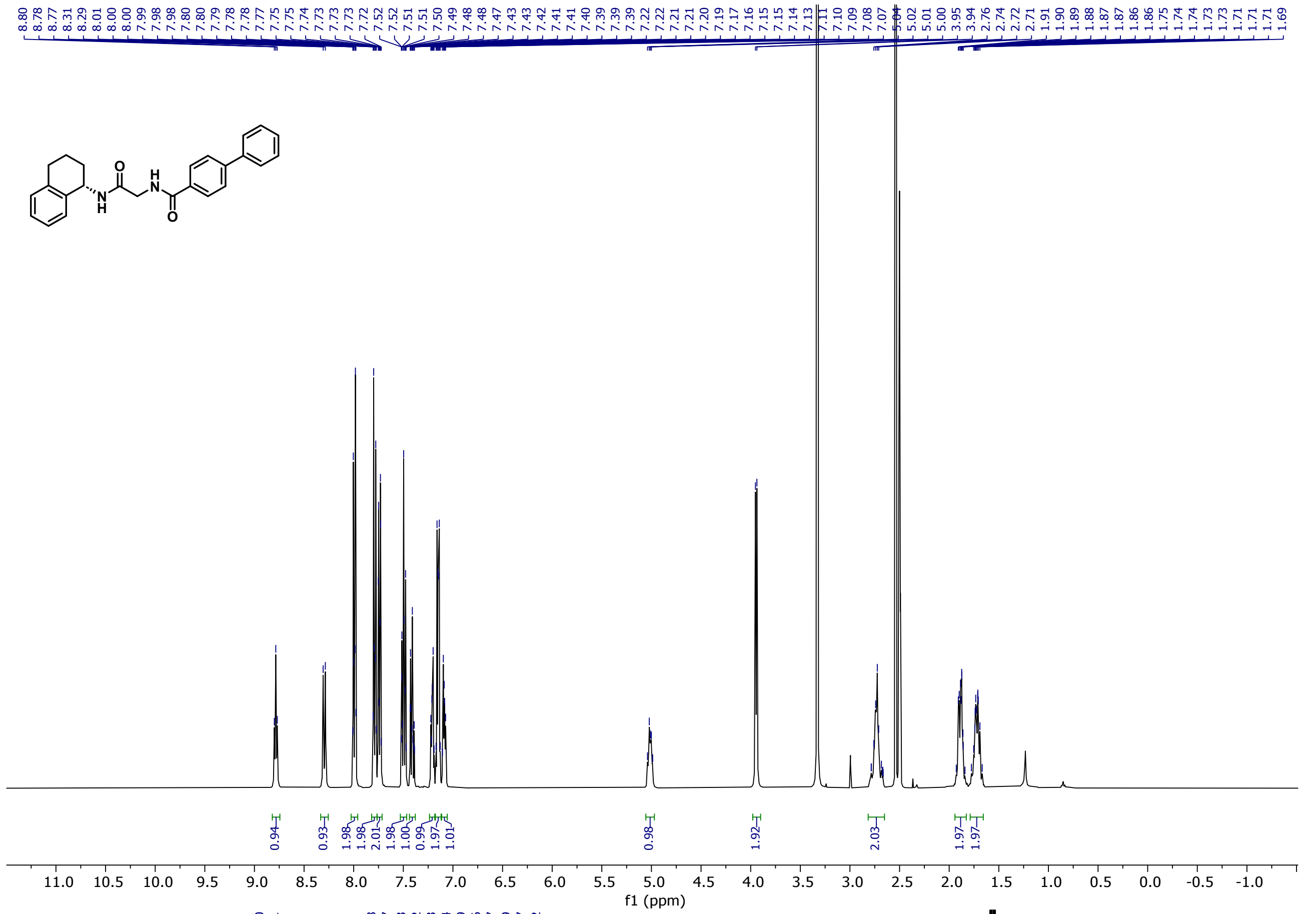

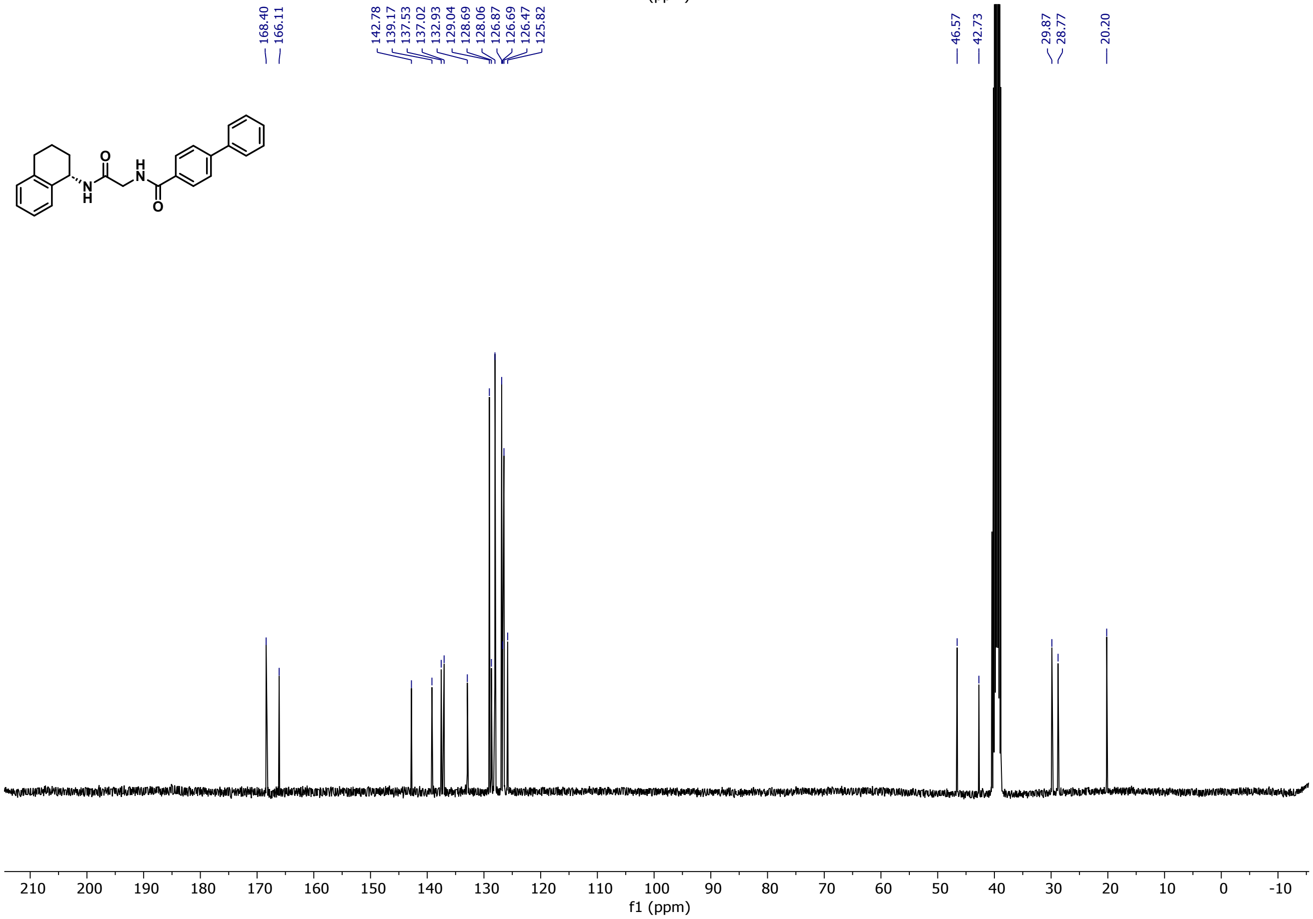

NMR spectra of (*S*)-1 in DMSO-d_6.

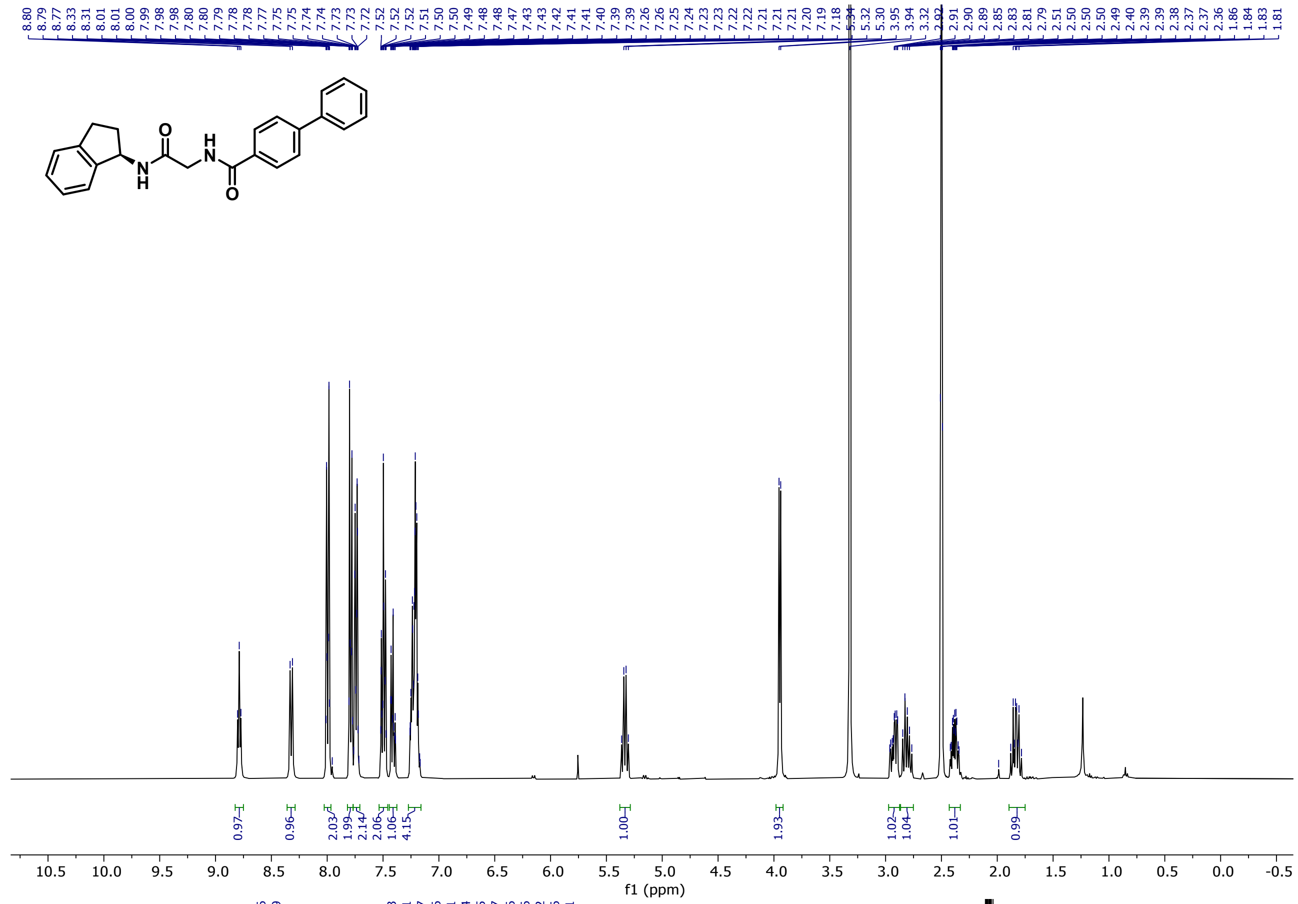

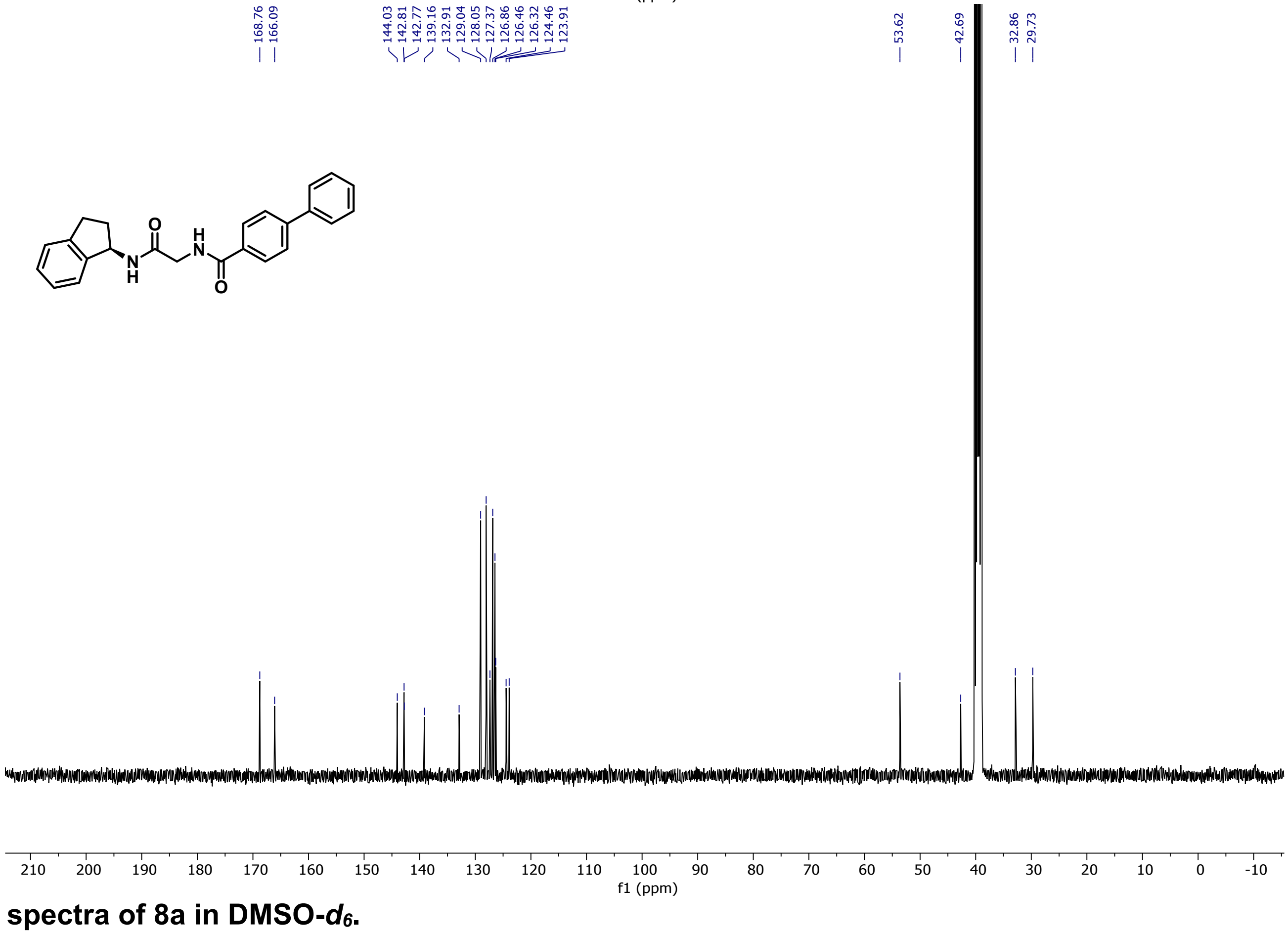

NMR spectra of 8a in DMSO-d_6.

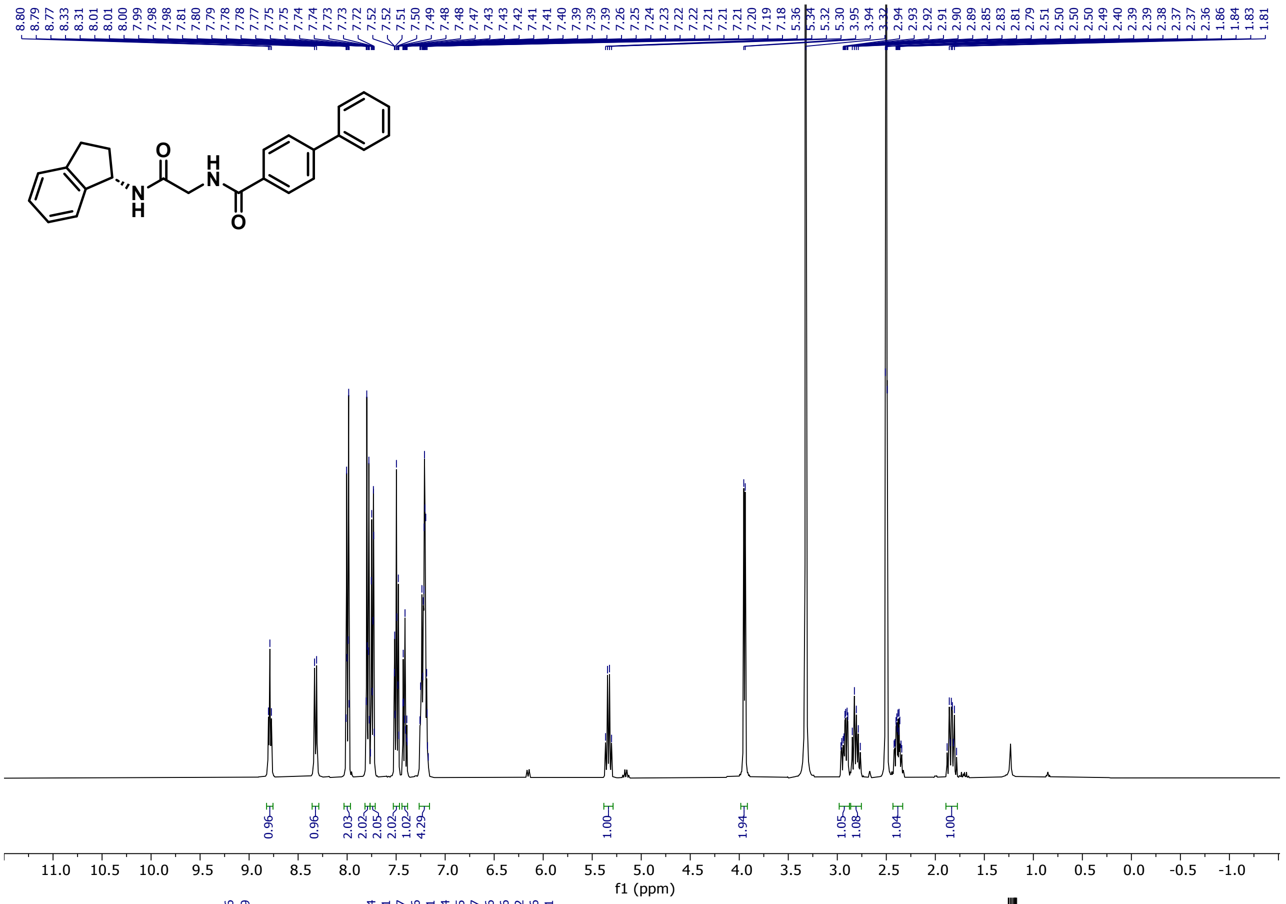

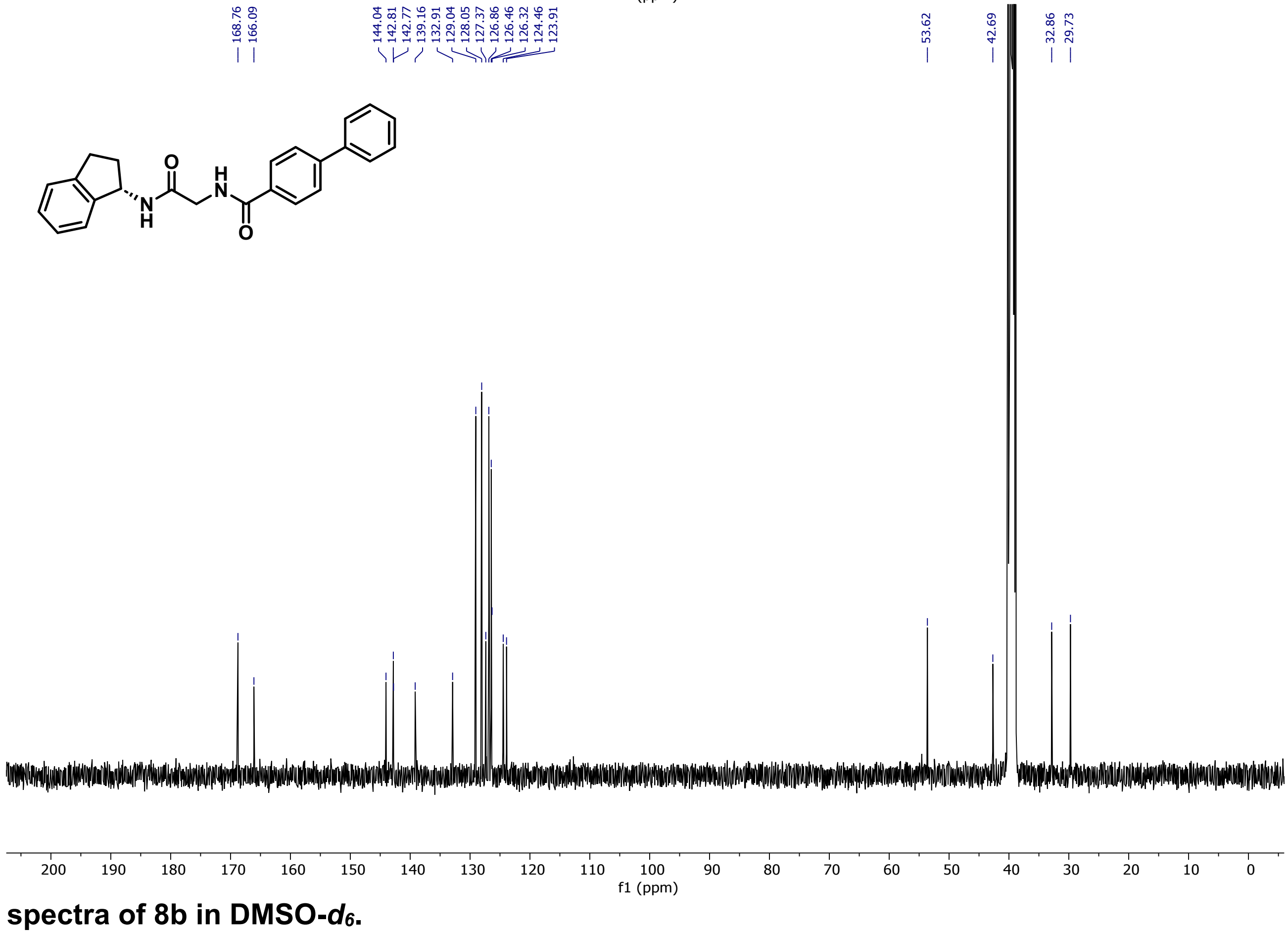

NMR spectra of 8b in DMSO-*d*$_6$.

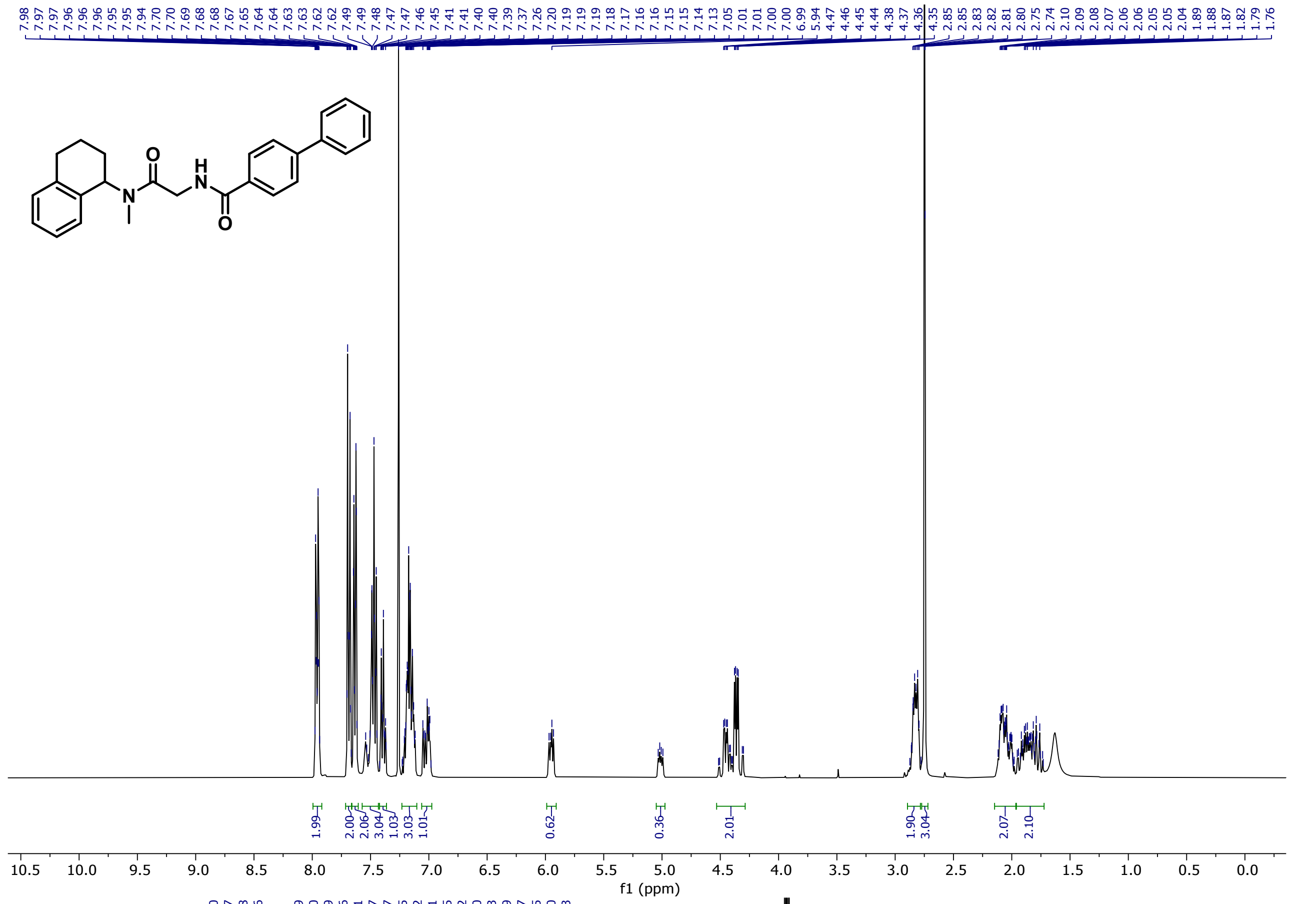

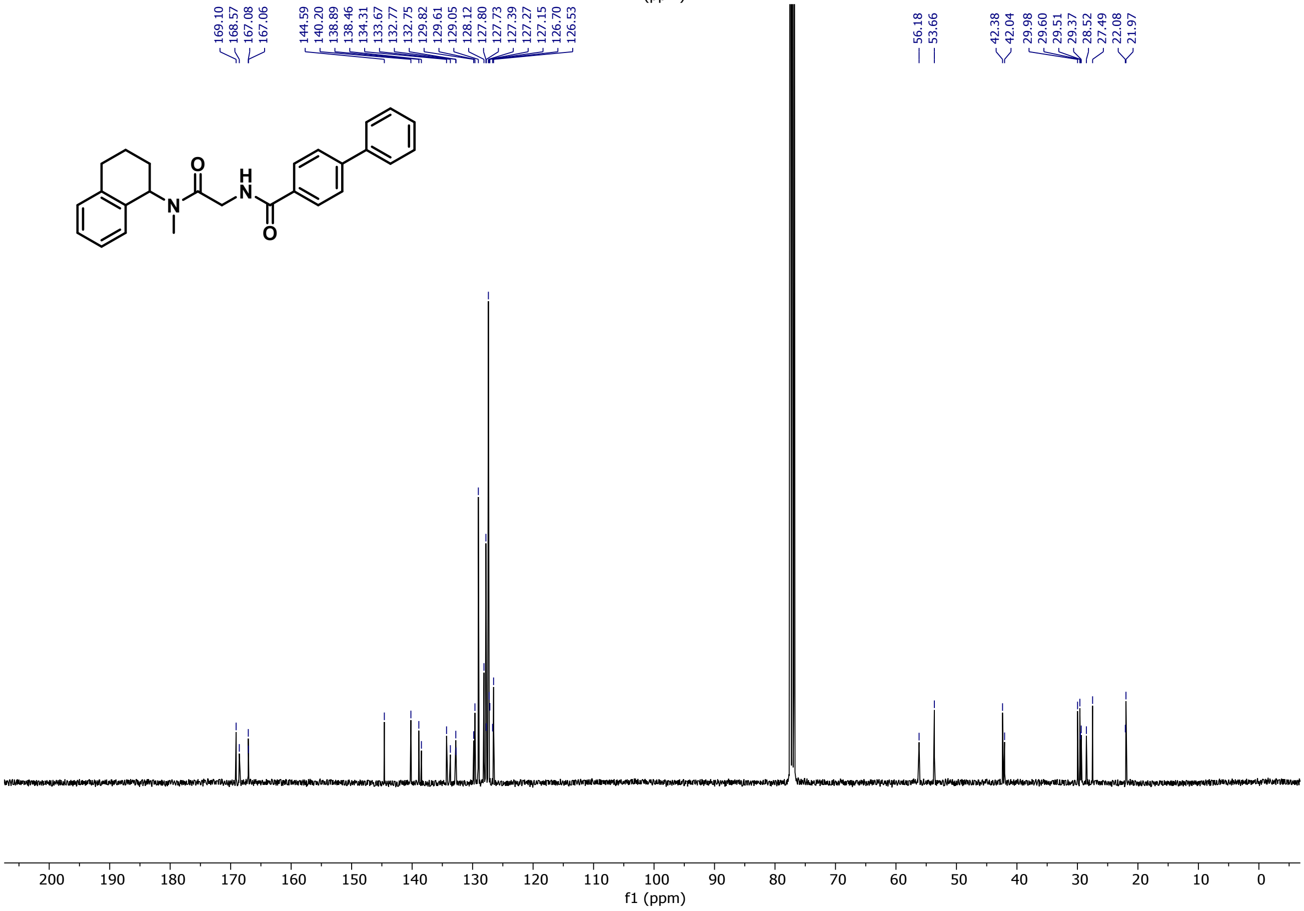

NMR spectra of 8c in $CDCl_3$.

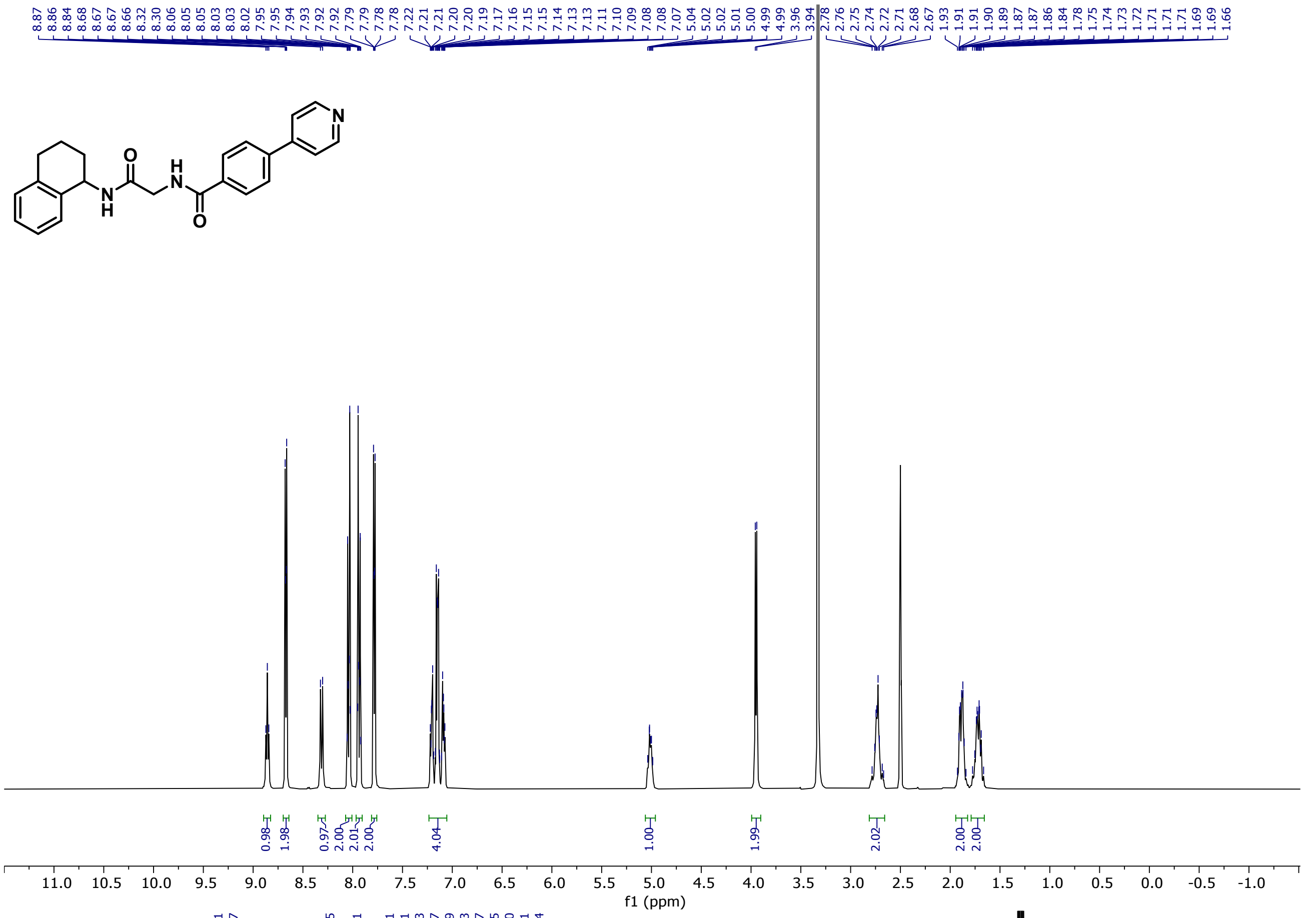

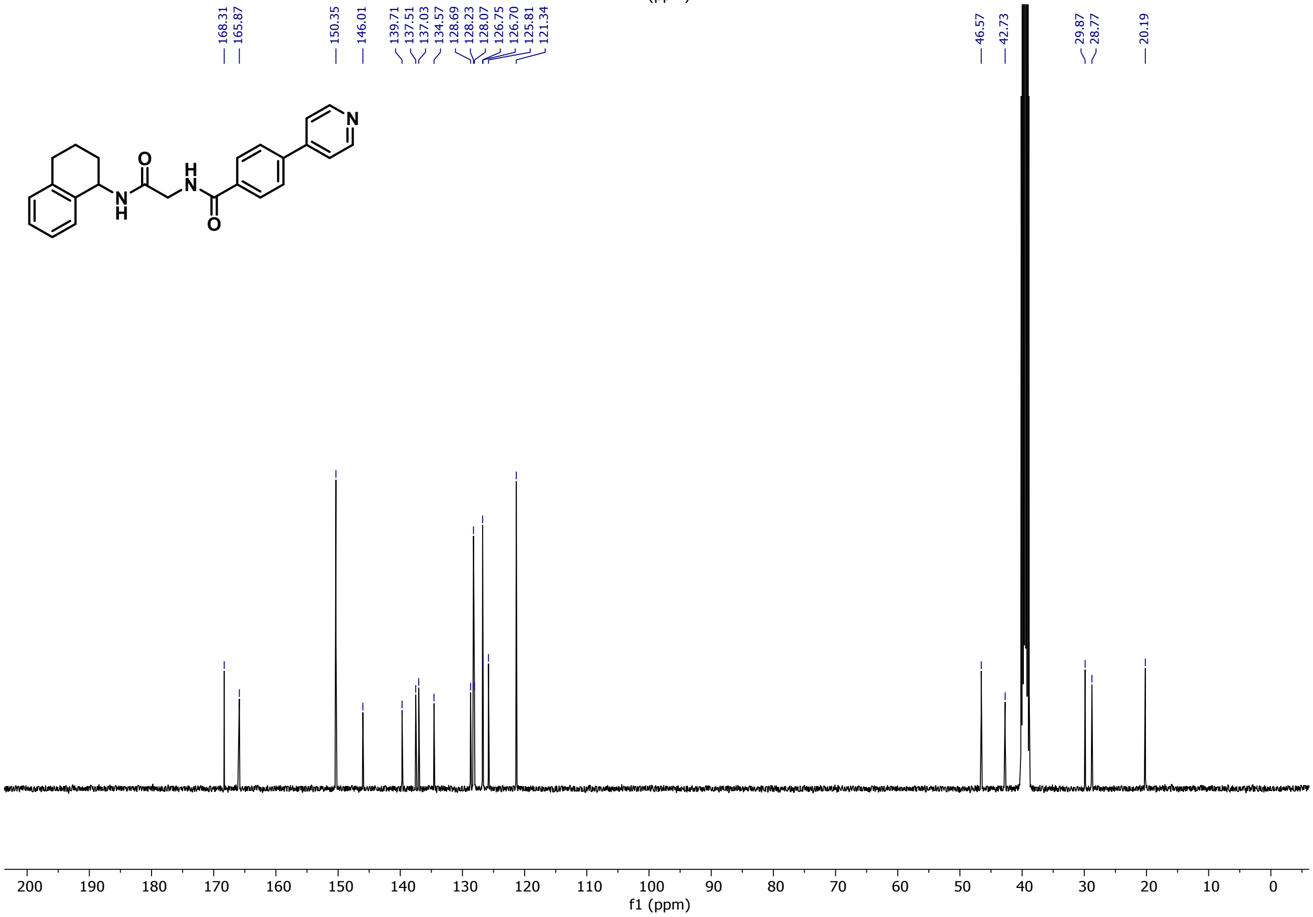

NMR spectra of 8d in DMSO-*d*$_6$.

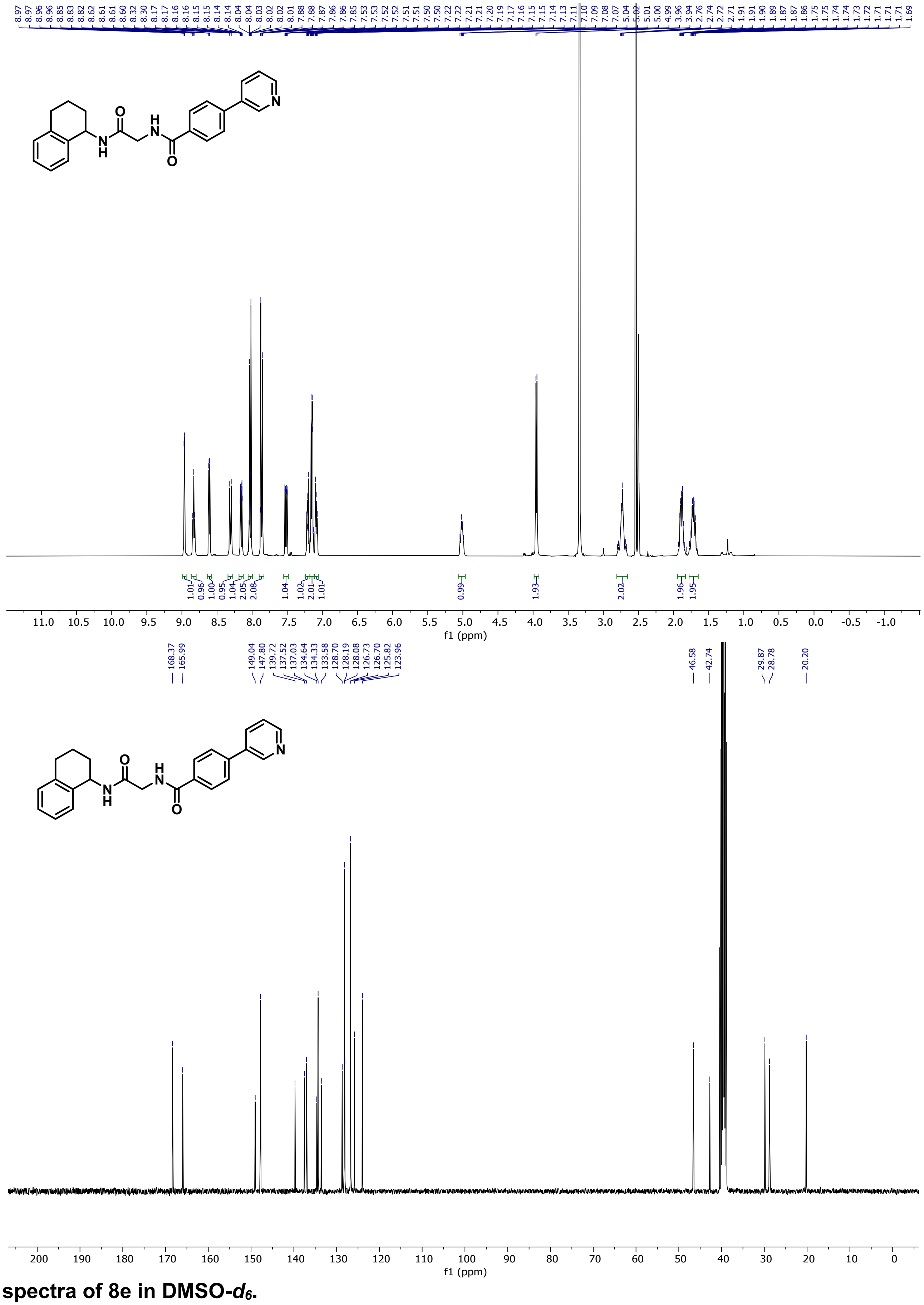

NMR spectra of 8e in DMSO-*d*$_6$.

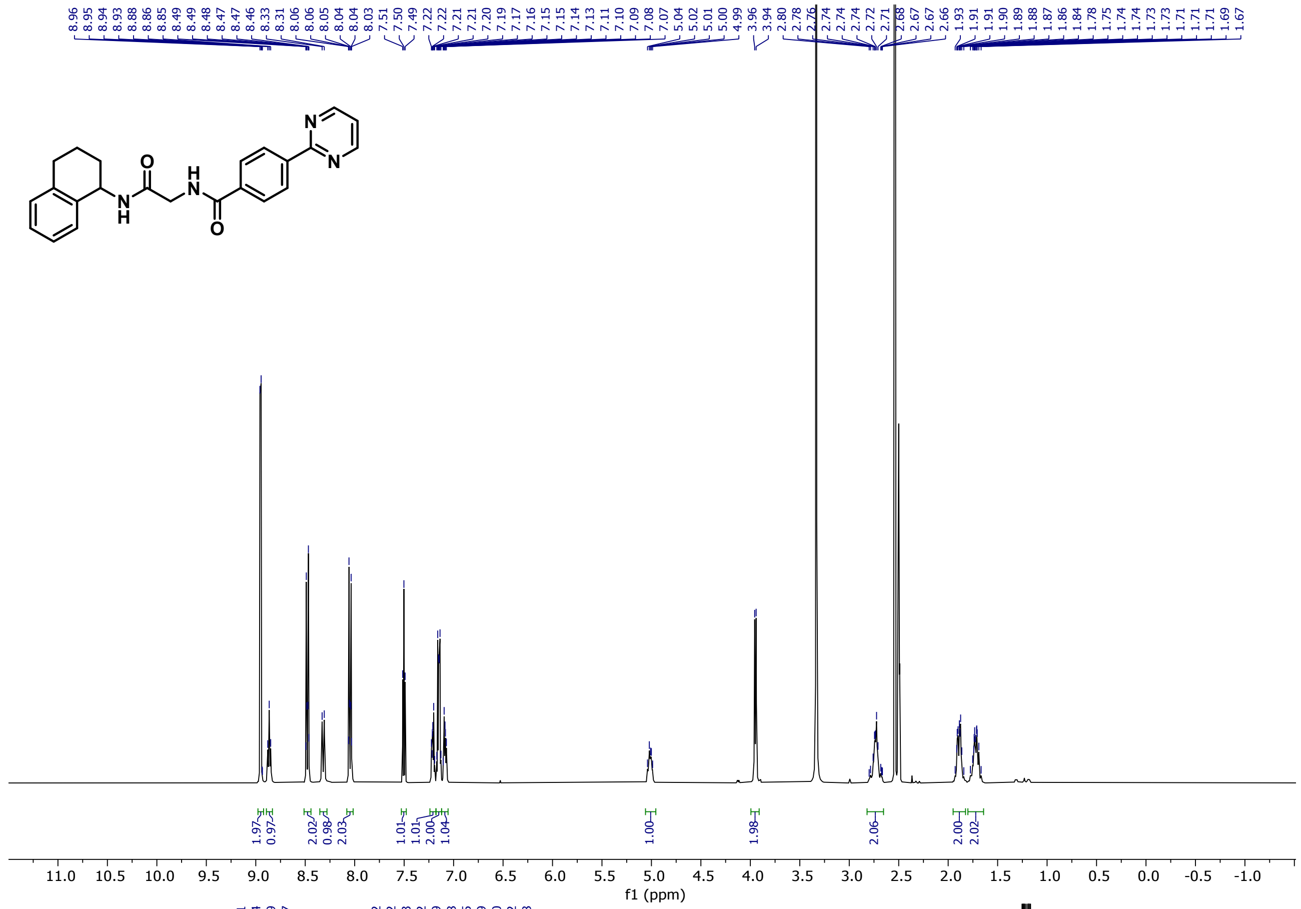

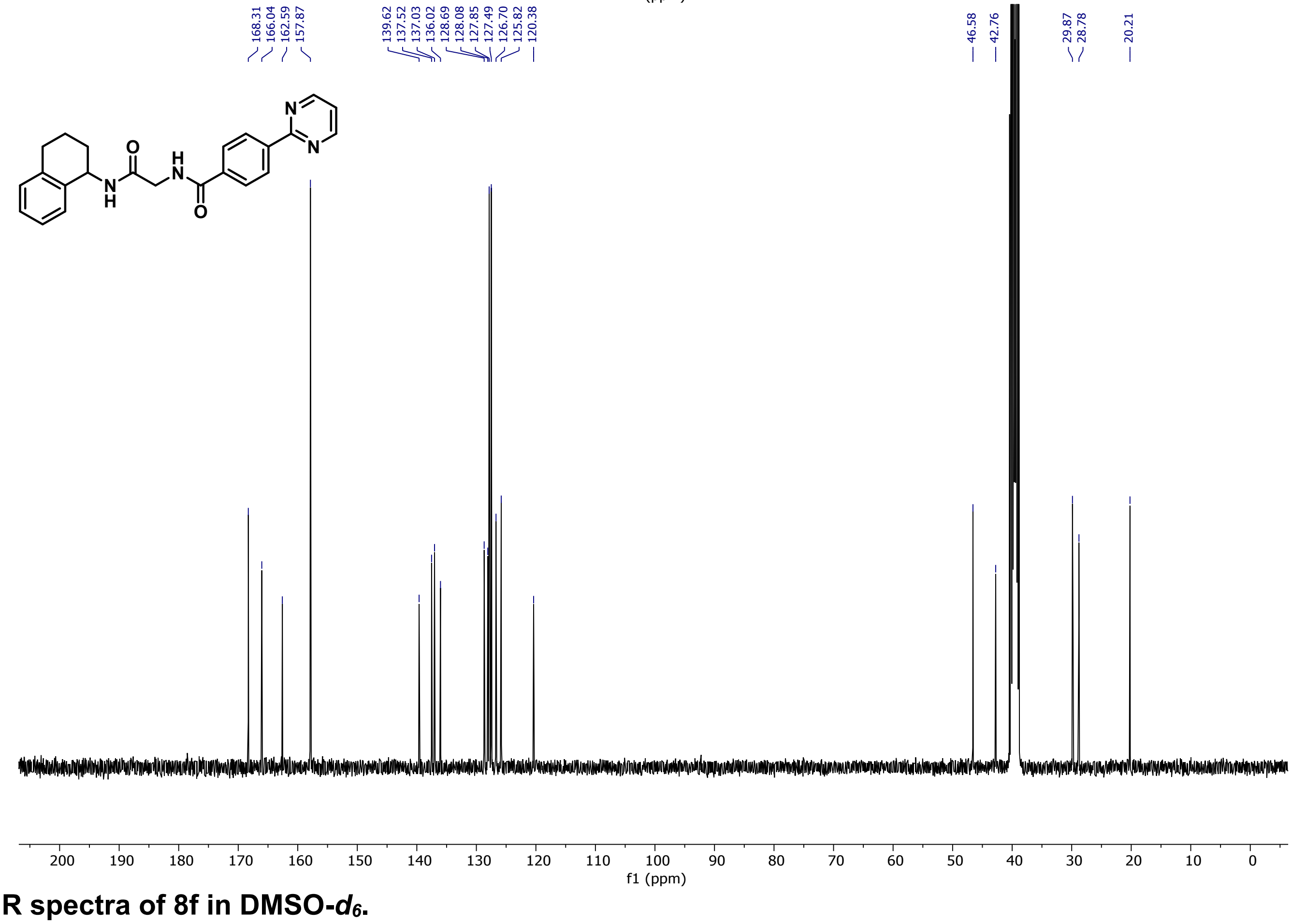

NMR spectra of 8f in DMSO-d_6.

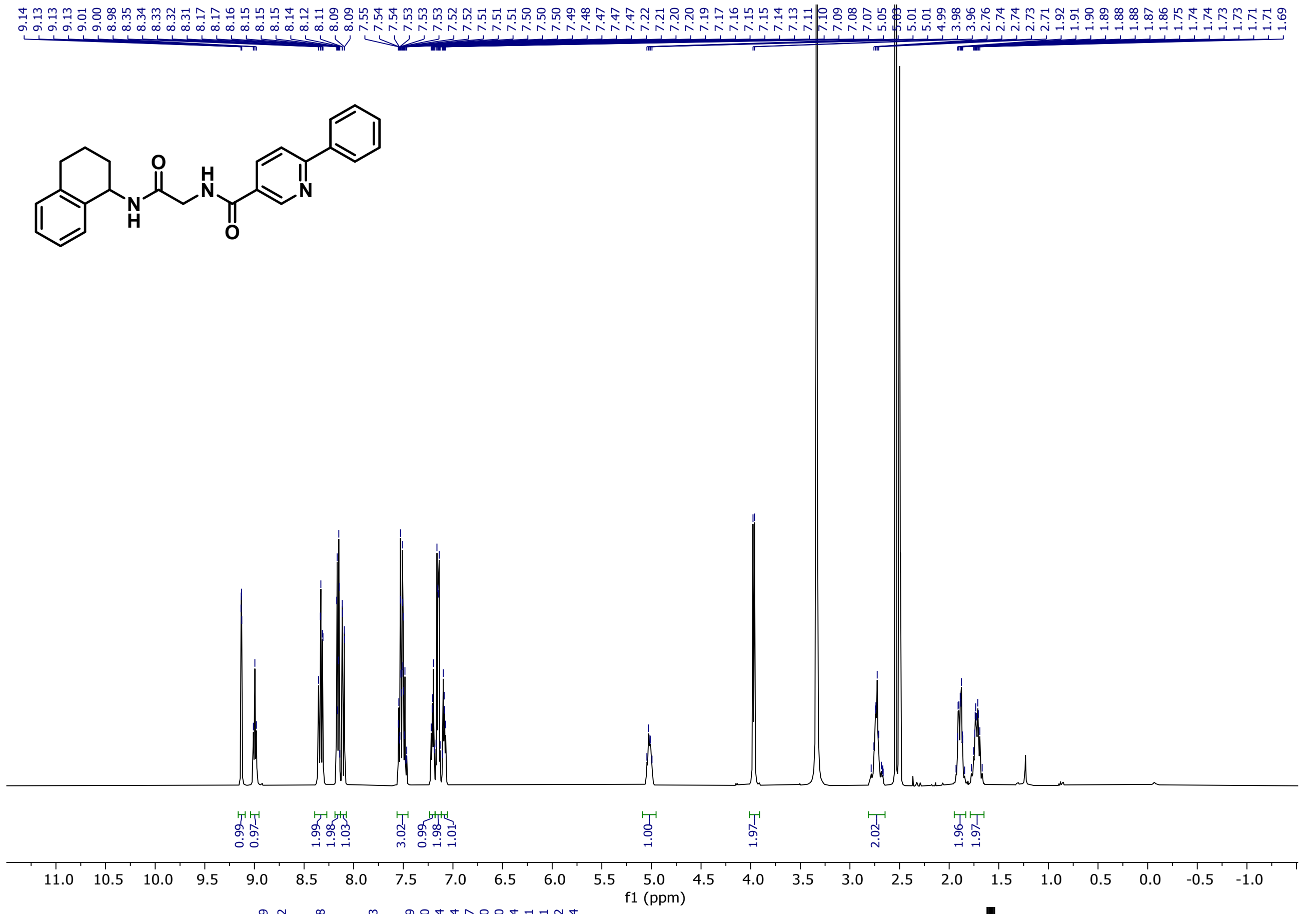

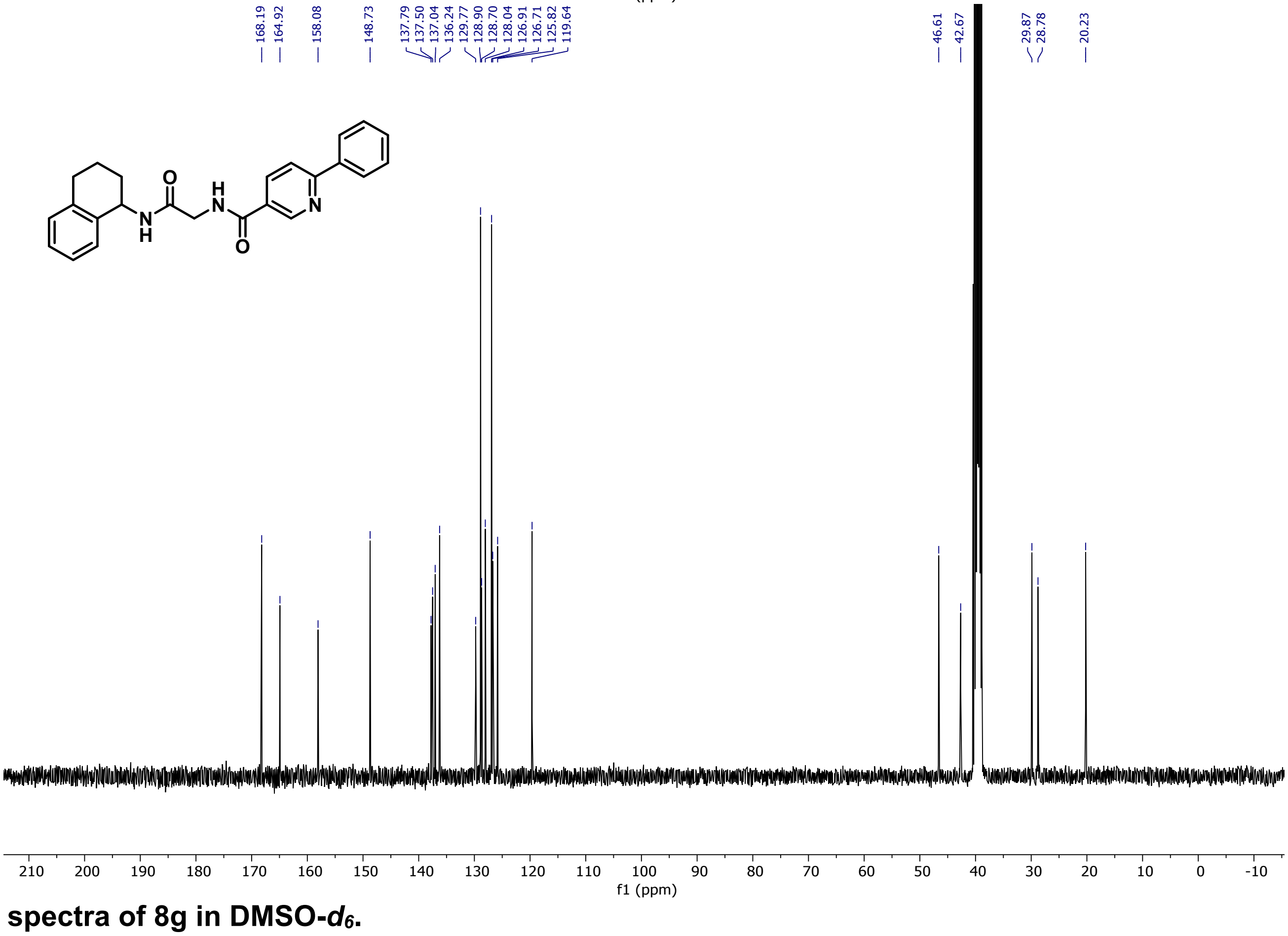

NMR spectra of 8g in DMSO-d_6.

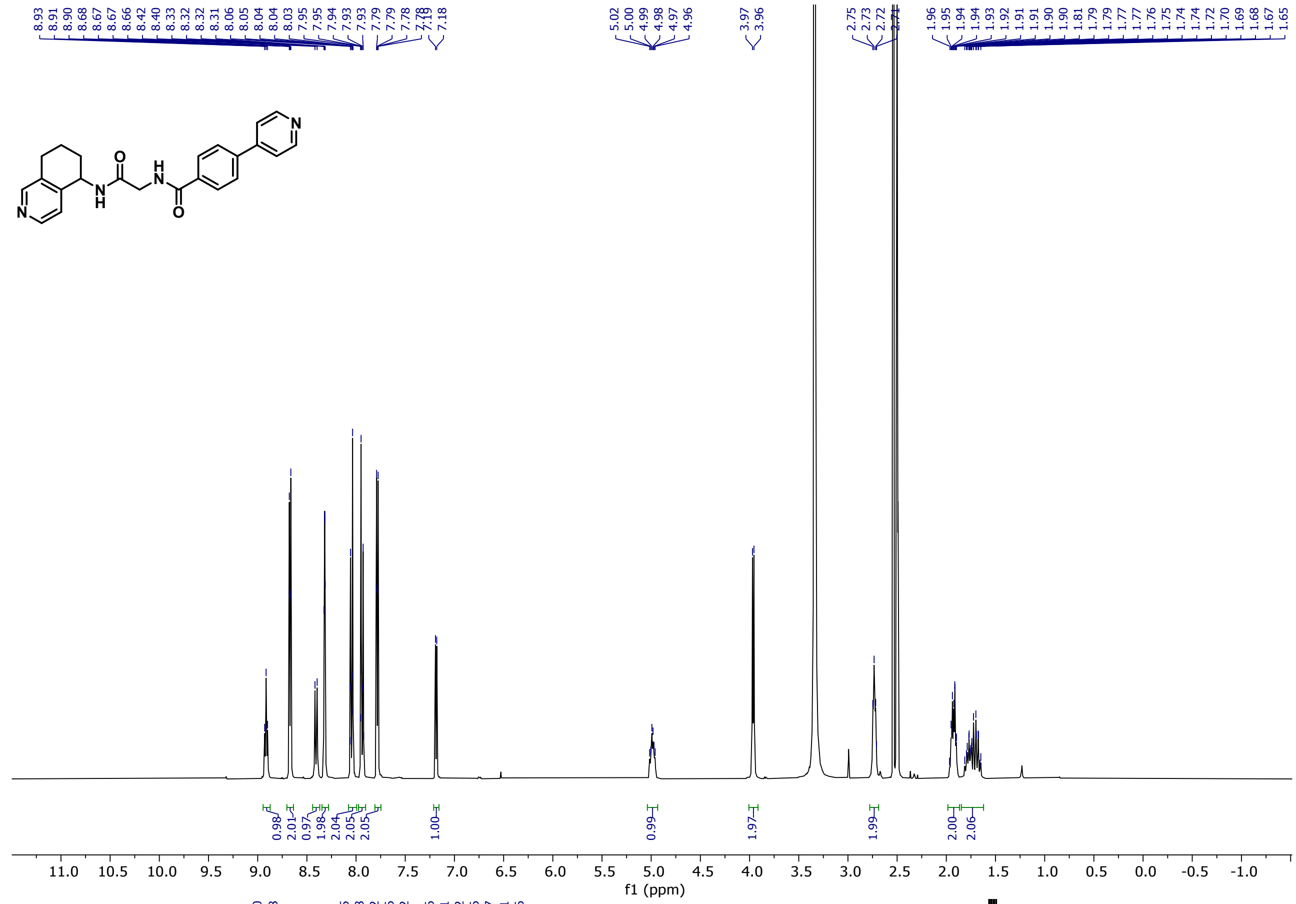

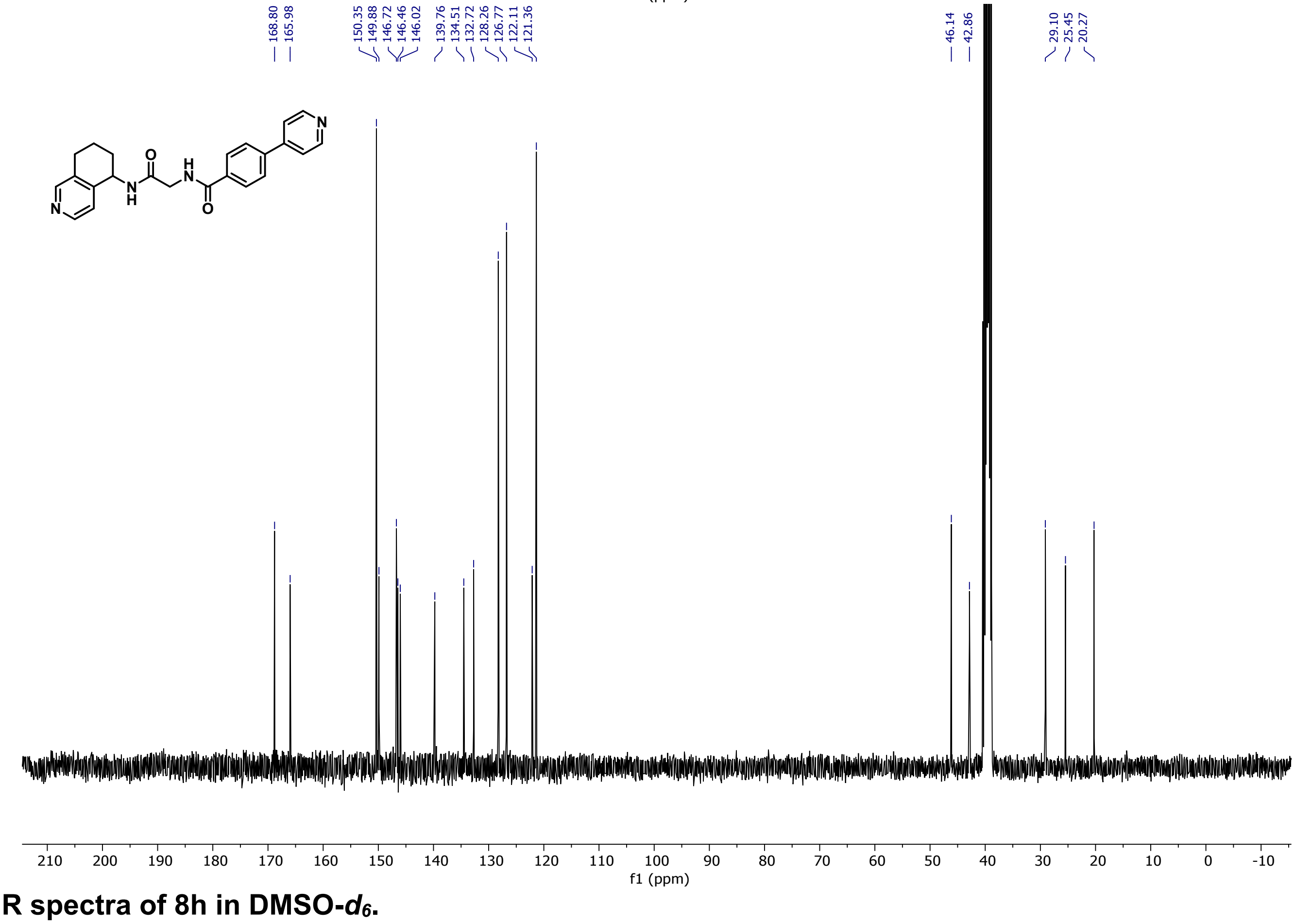

NMR spectra of 8h in DMSO-d_6.

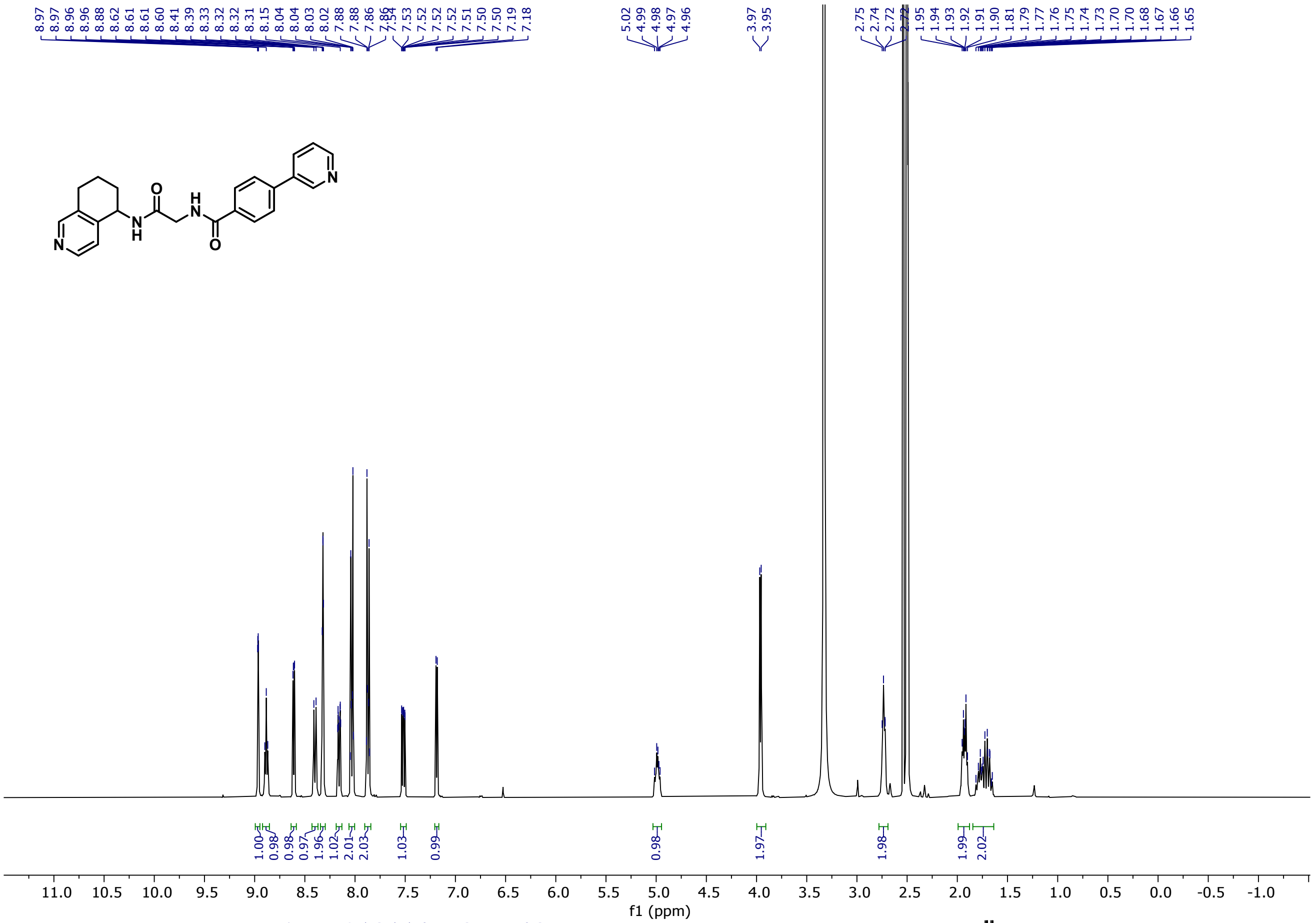

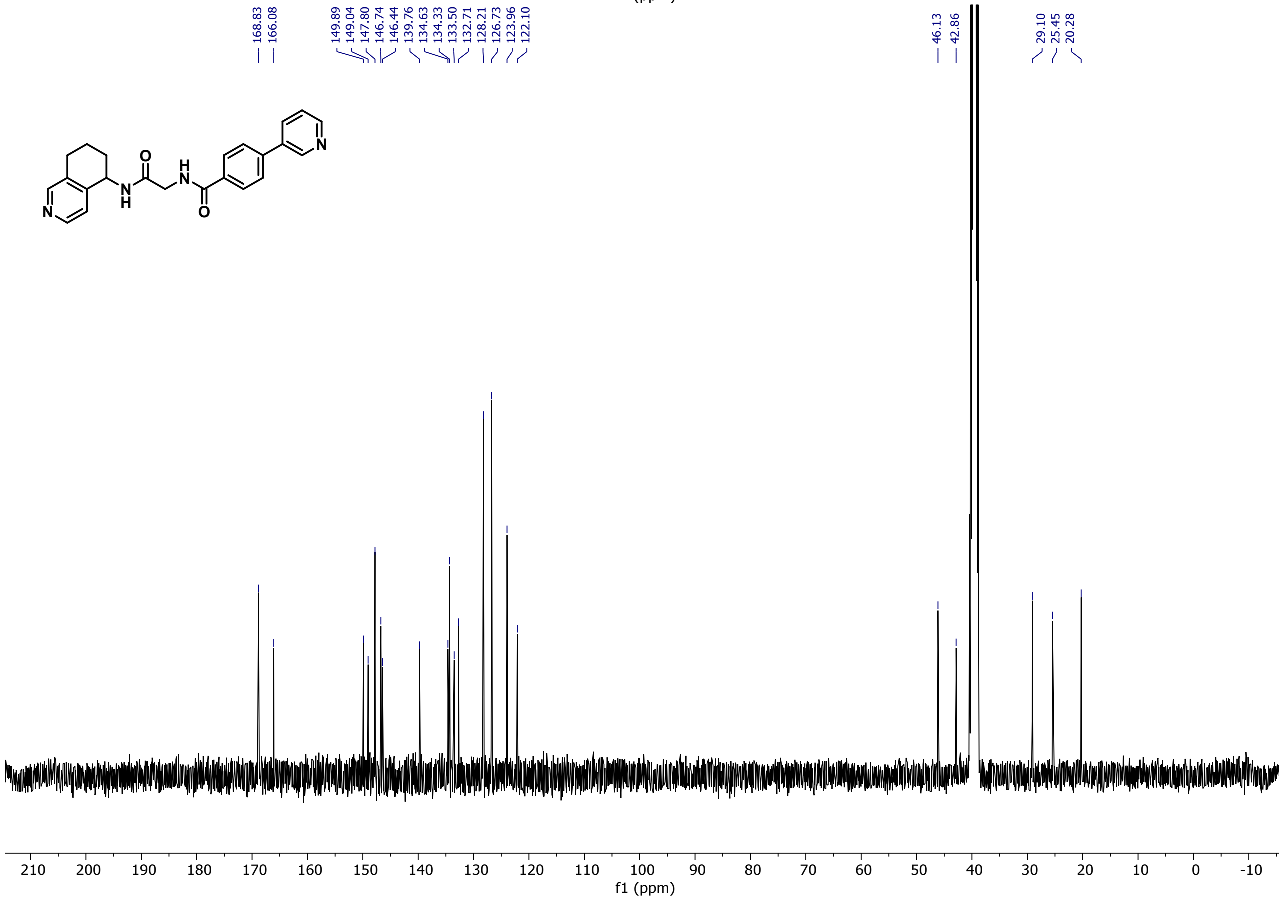

NMR spectra of 8i in DMSO-d_6.

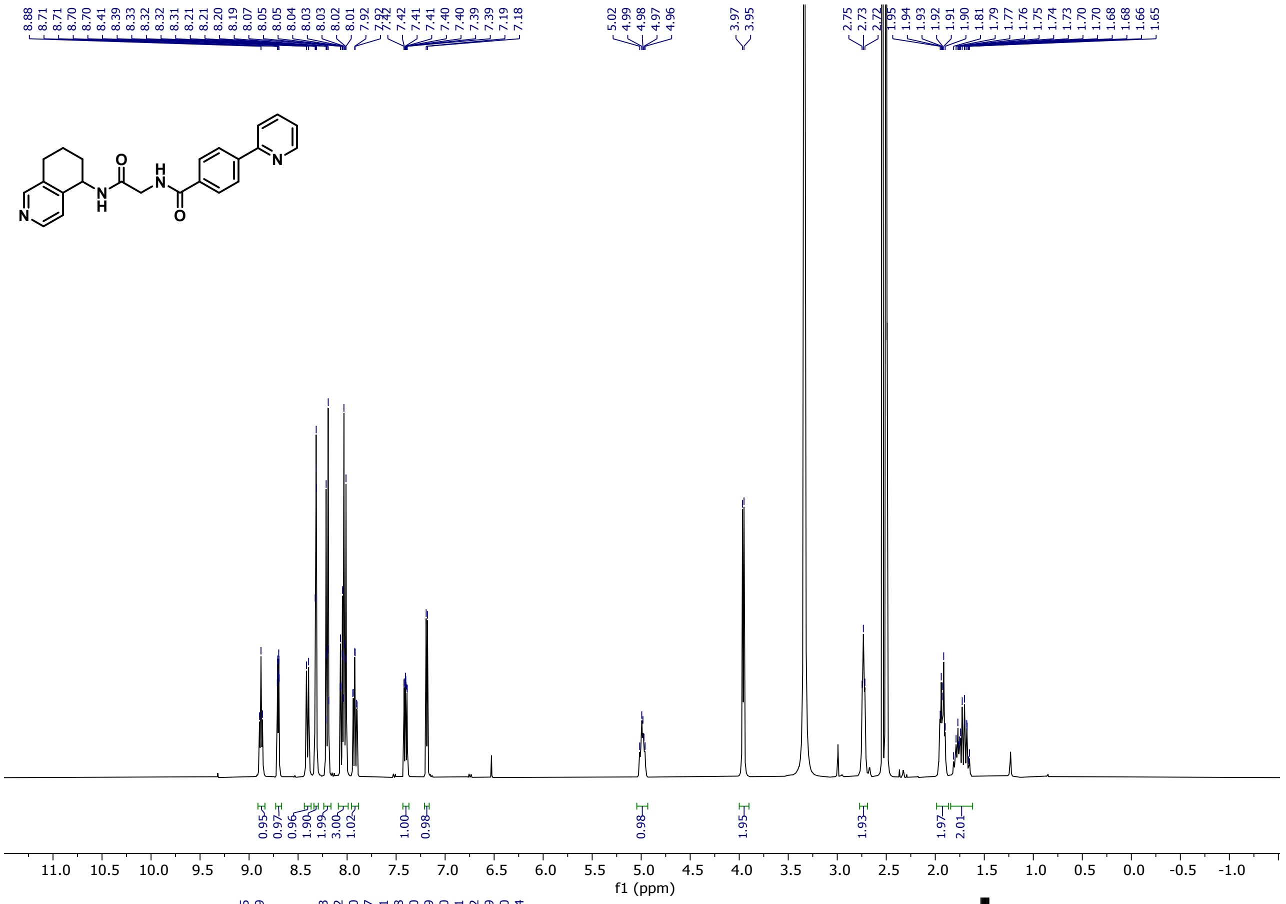

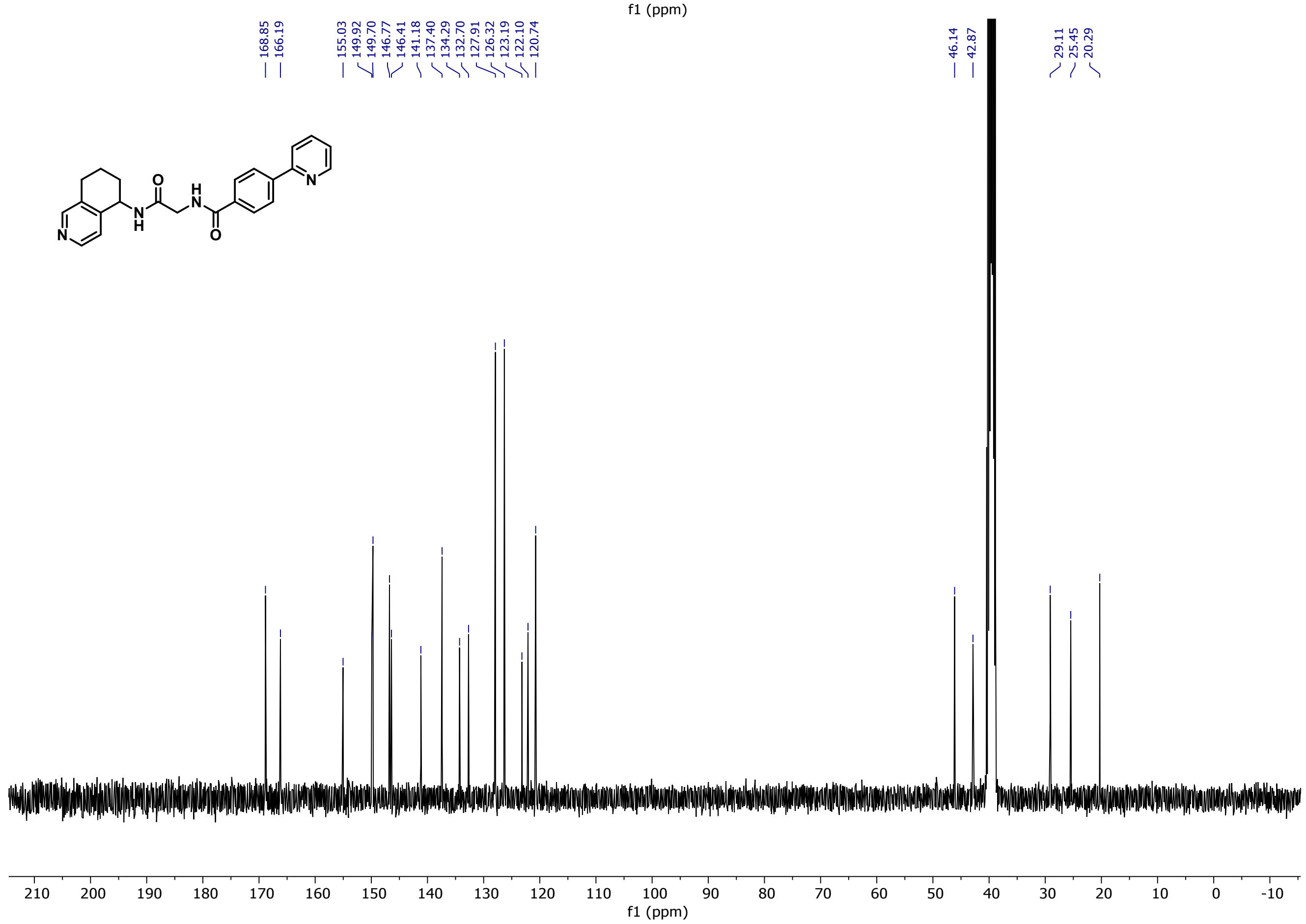

NMR spectra of 8j in DMSO-d_6.

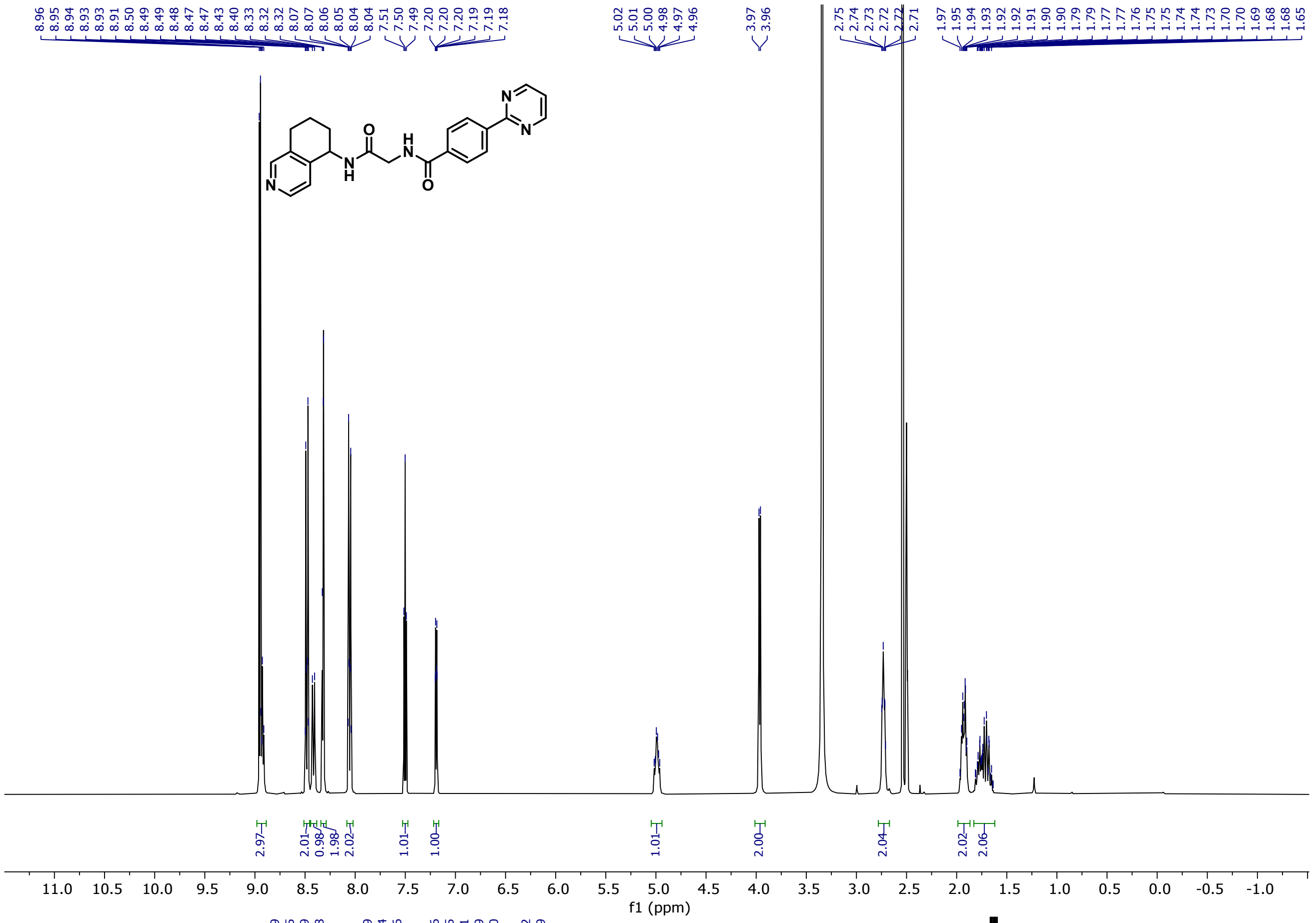

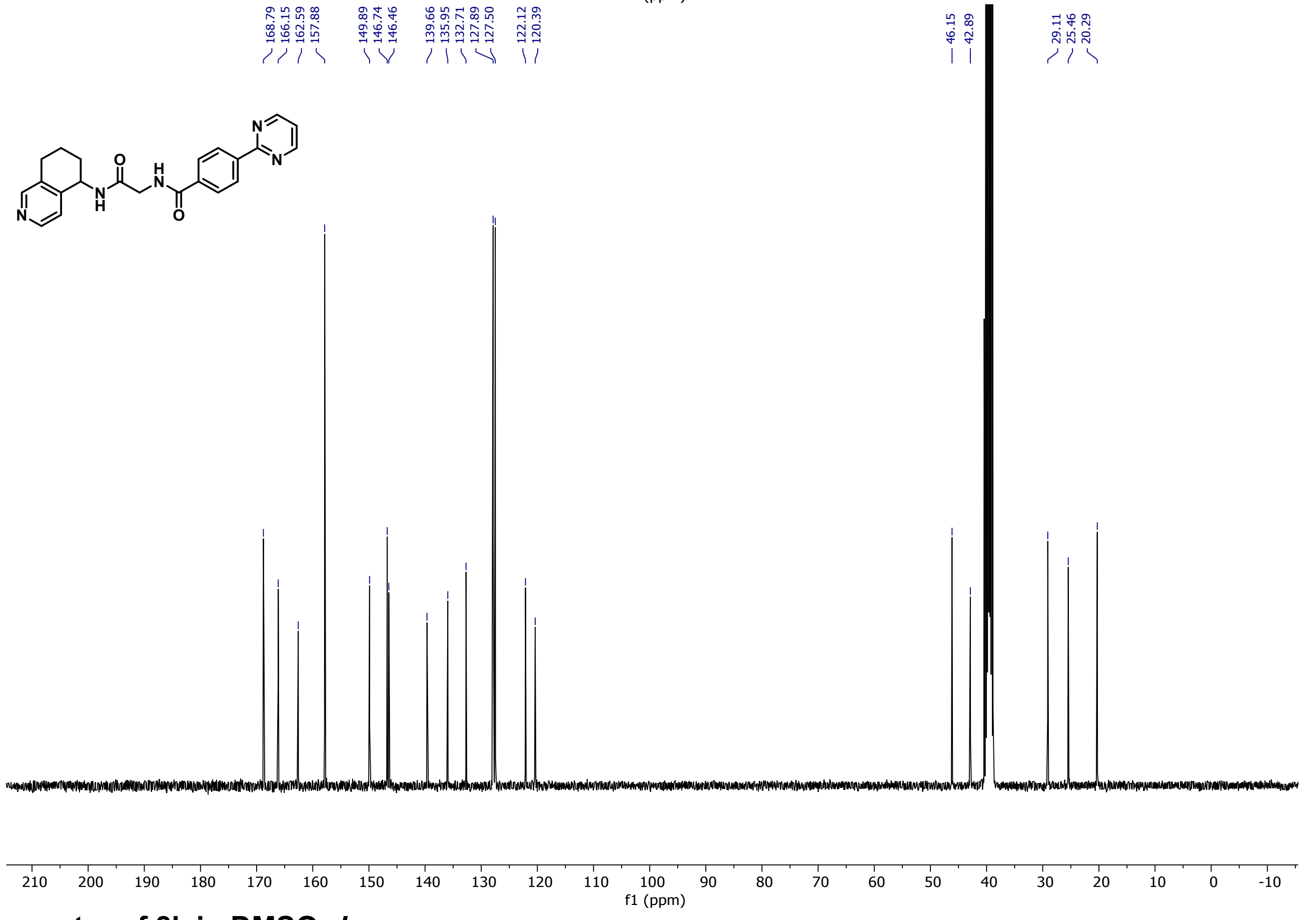

NMR spectra of 8k in DMSO-d_6.

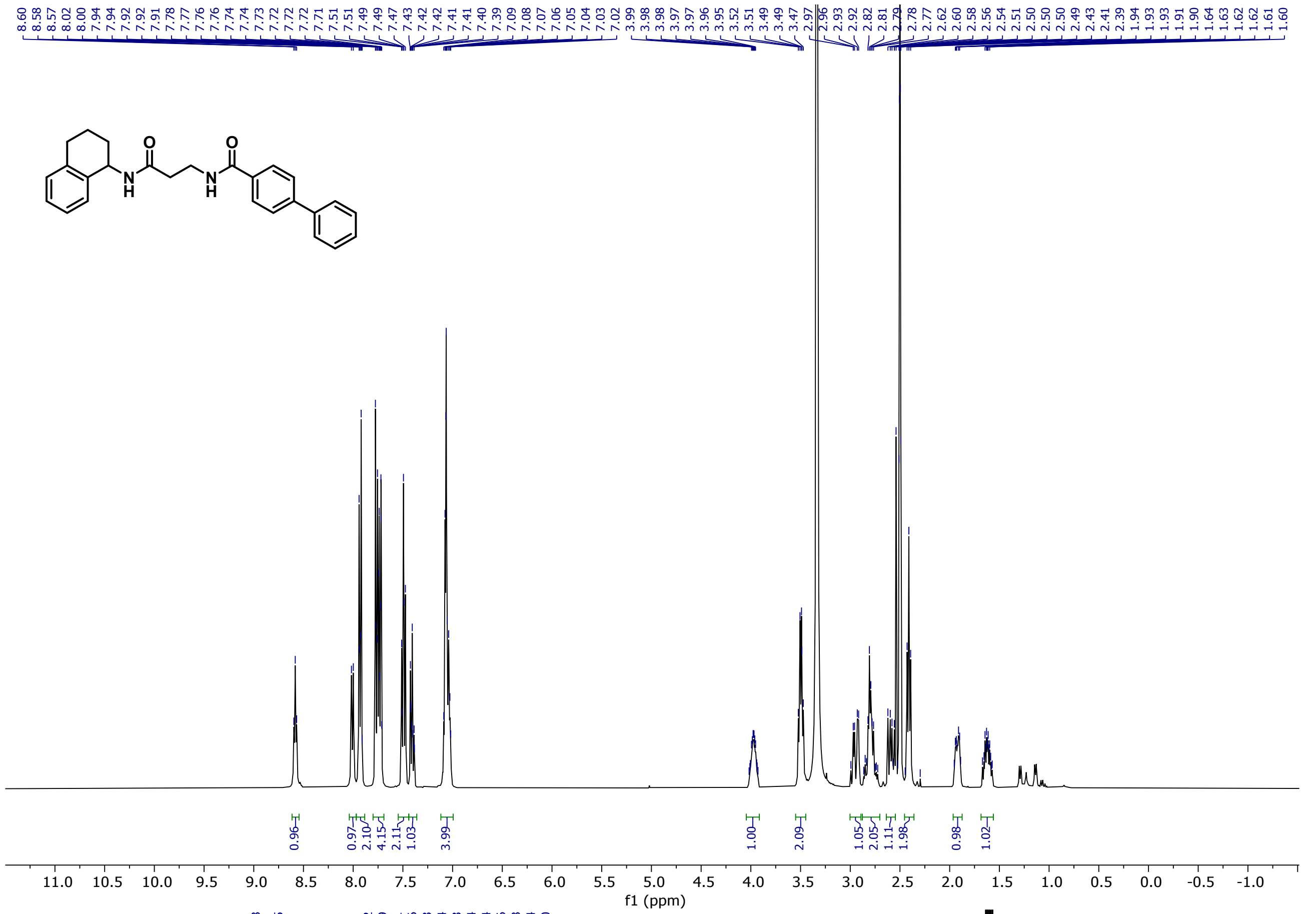

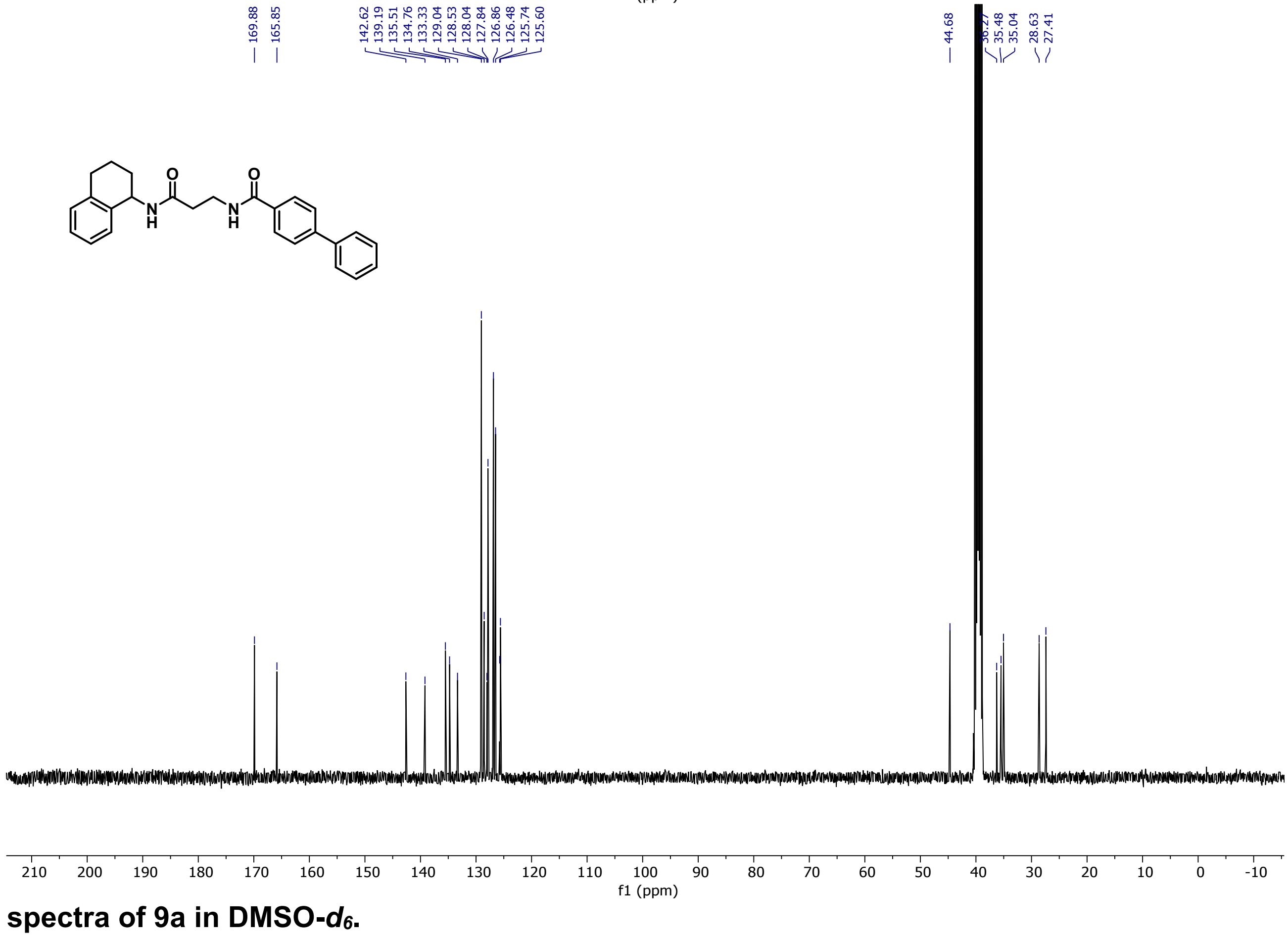

NMR spectra of 9a in DMSO-d_6.

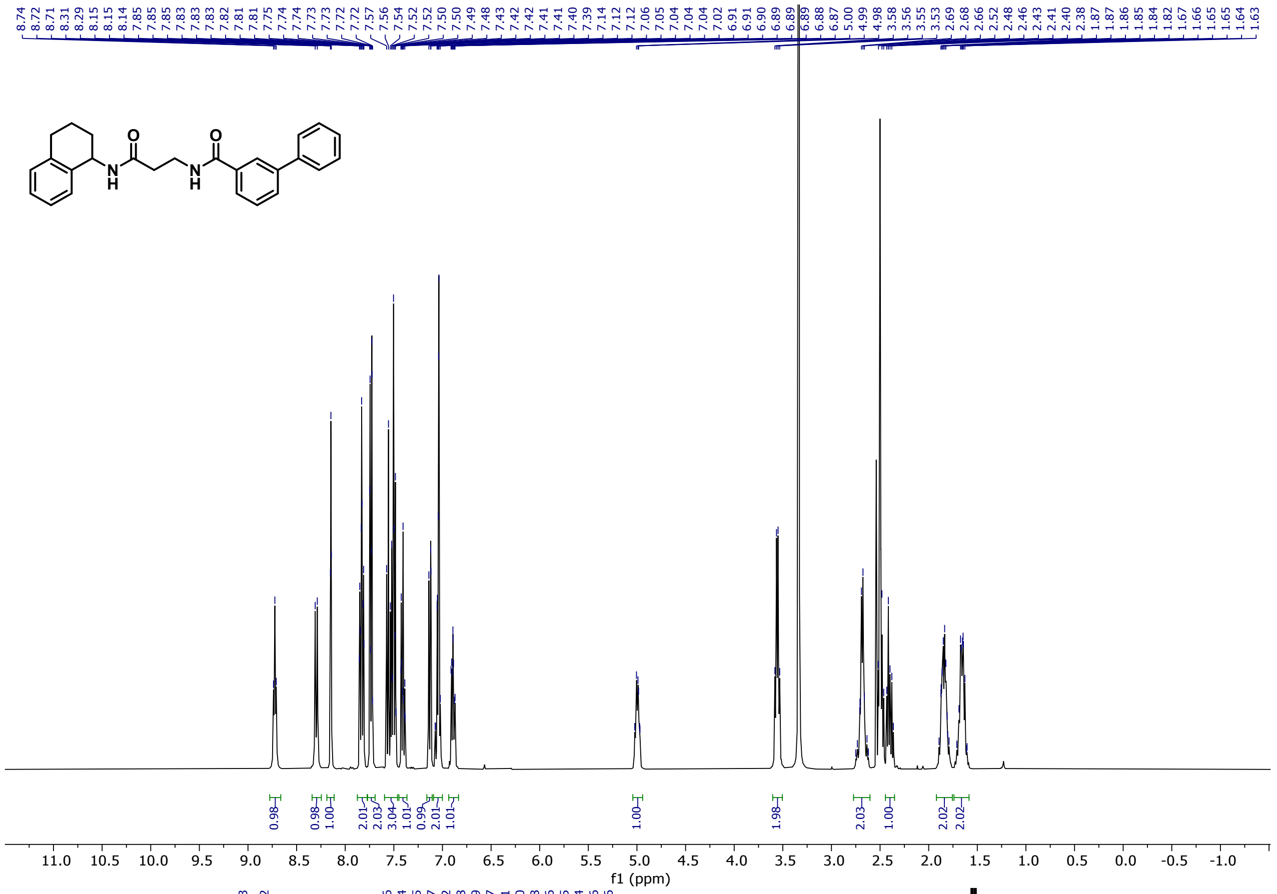

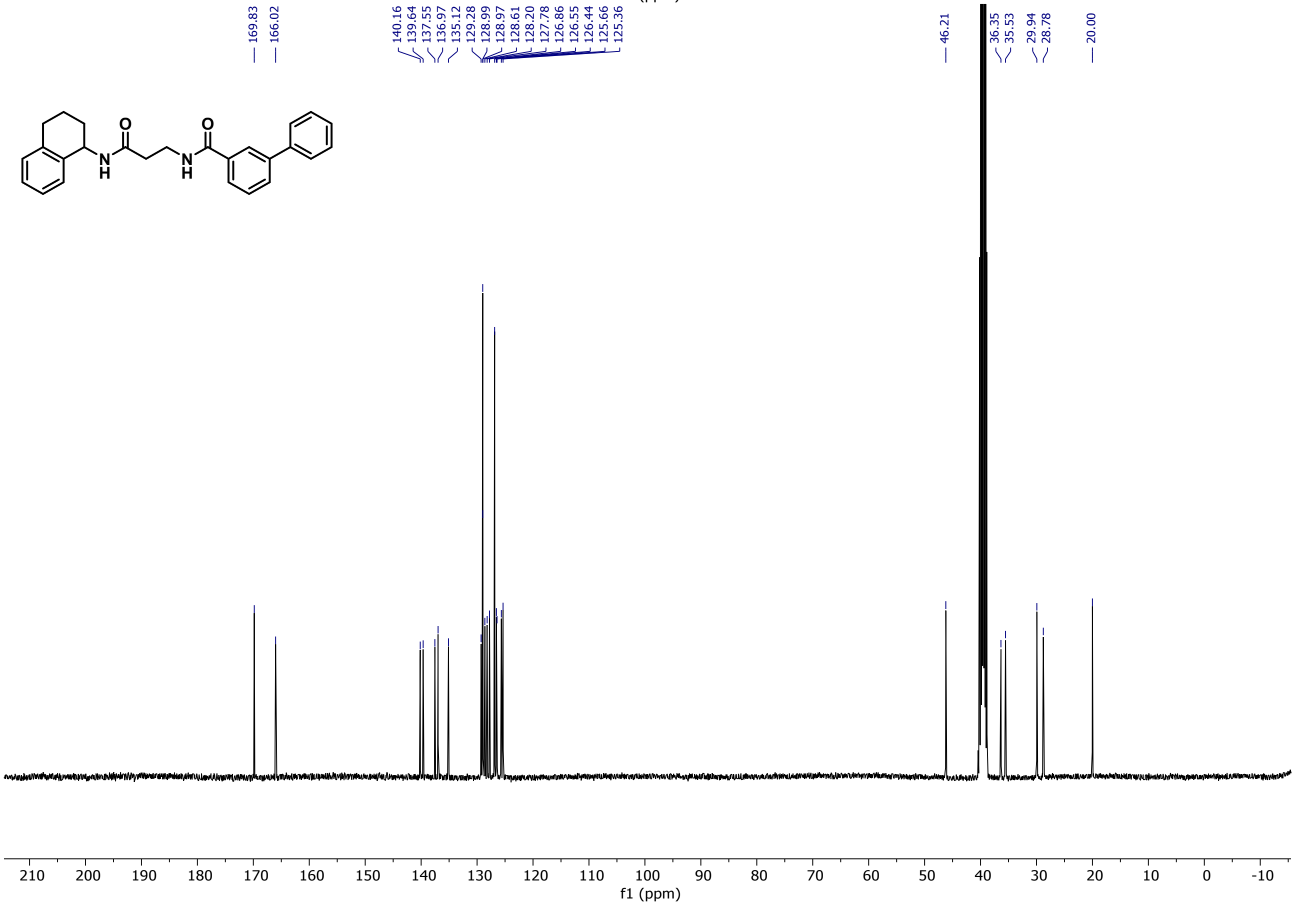

NMR spectra of 9b in DMSO-d_6.

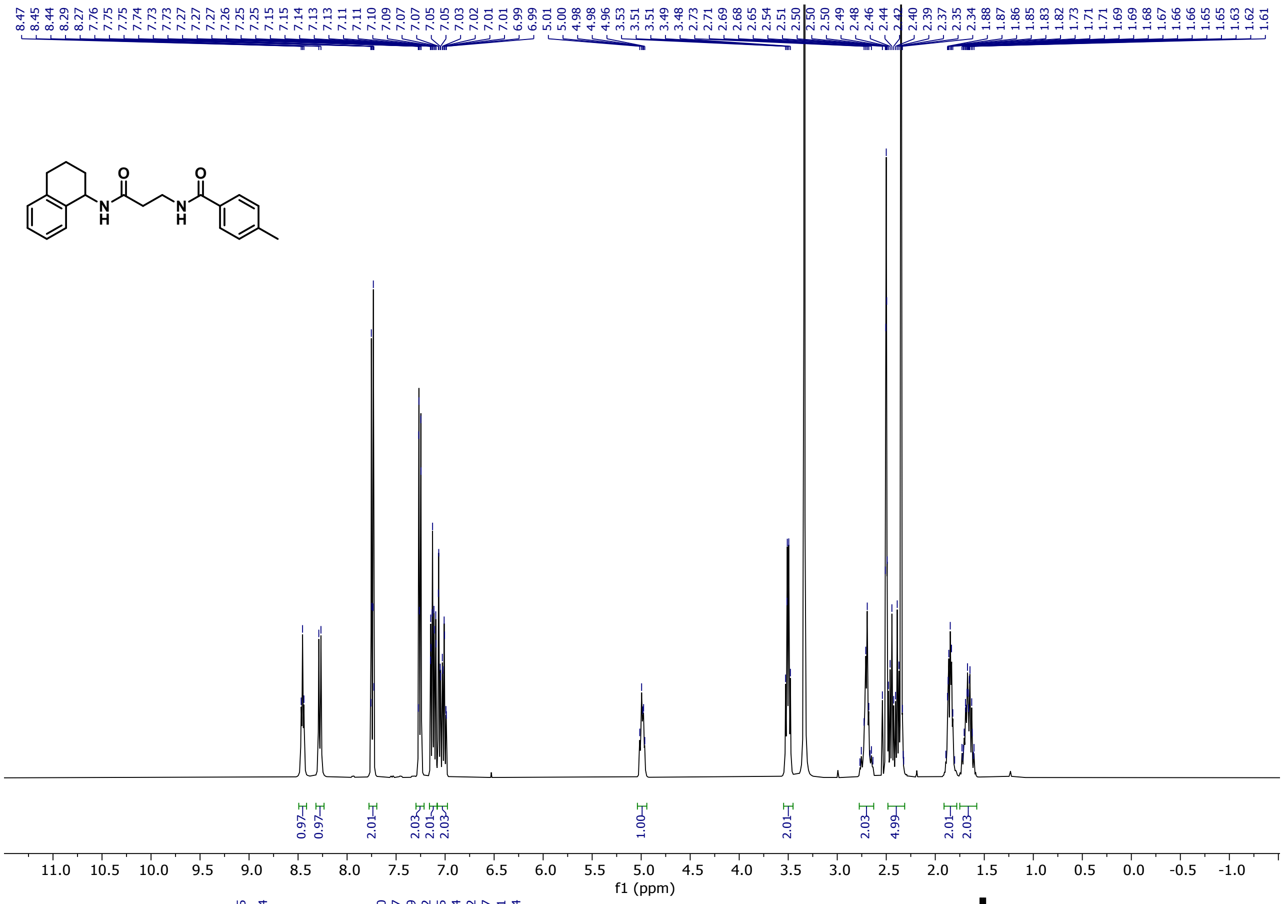

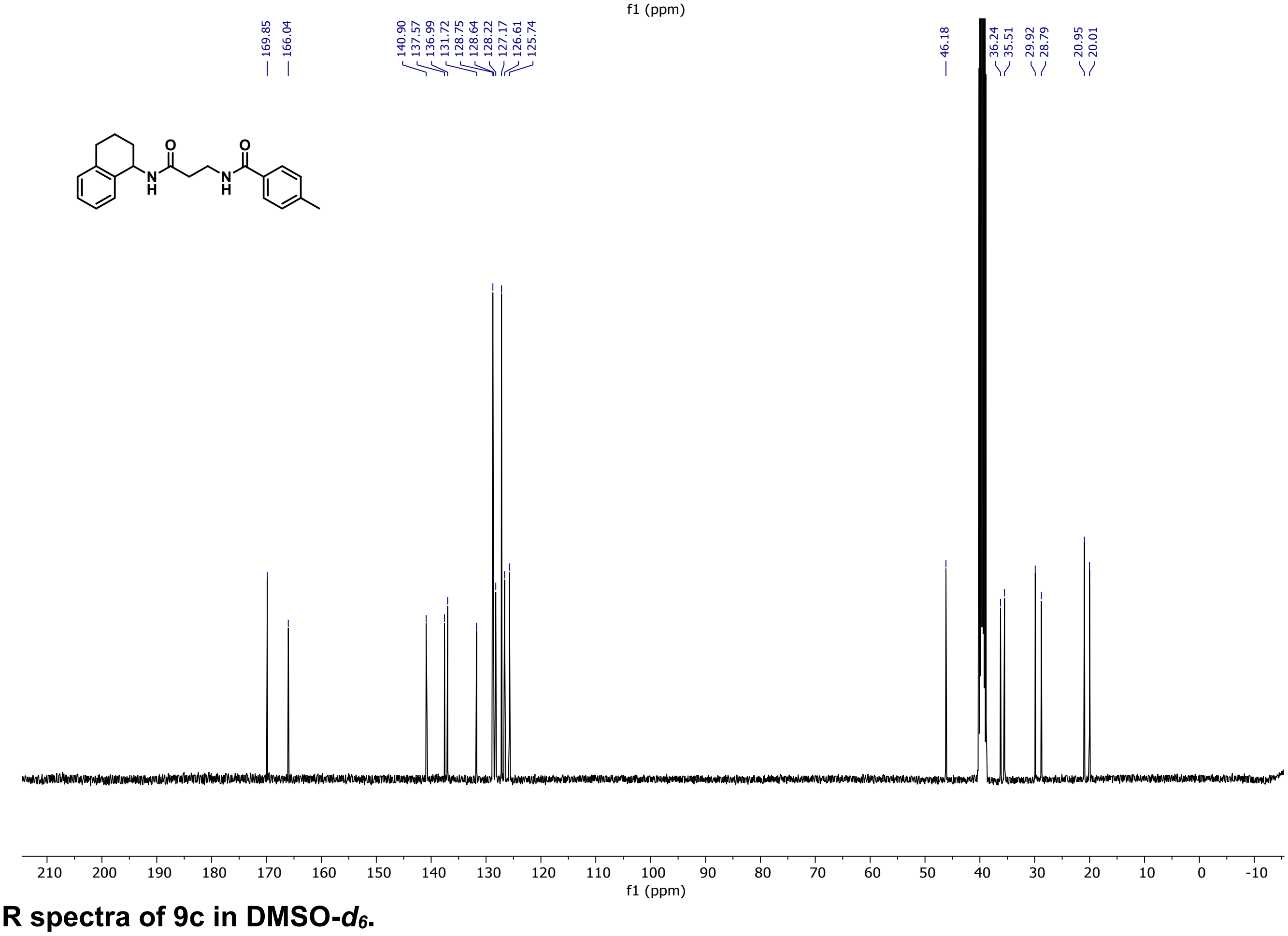

NMR spectra of 9c in DMSO-d_6.

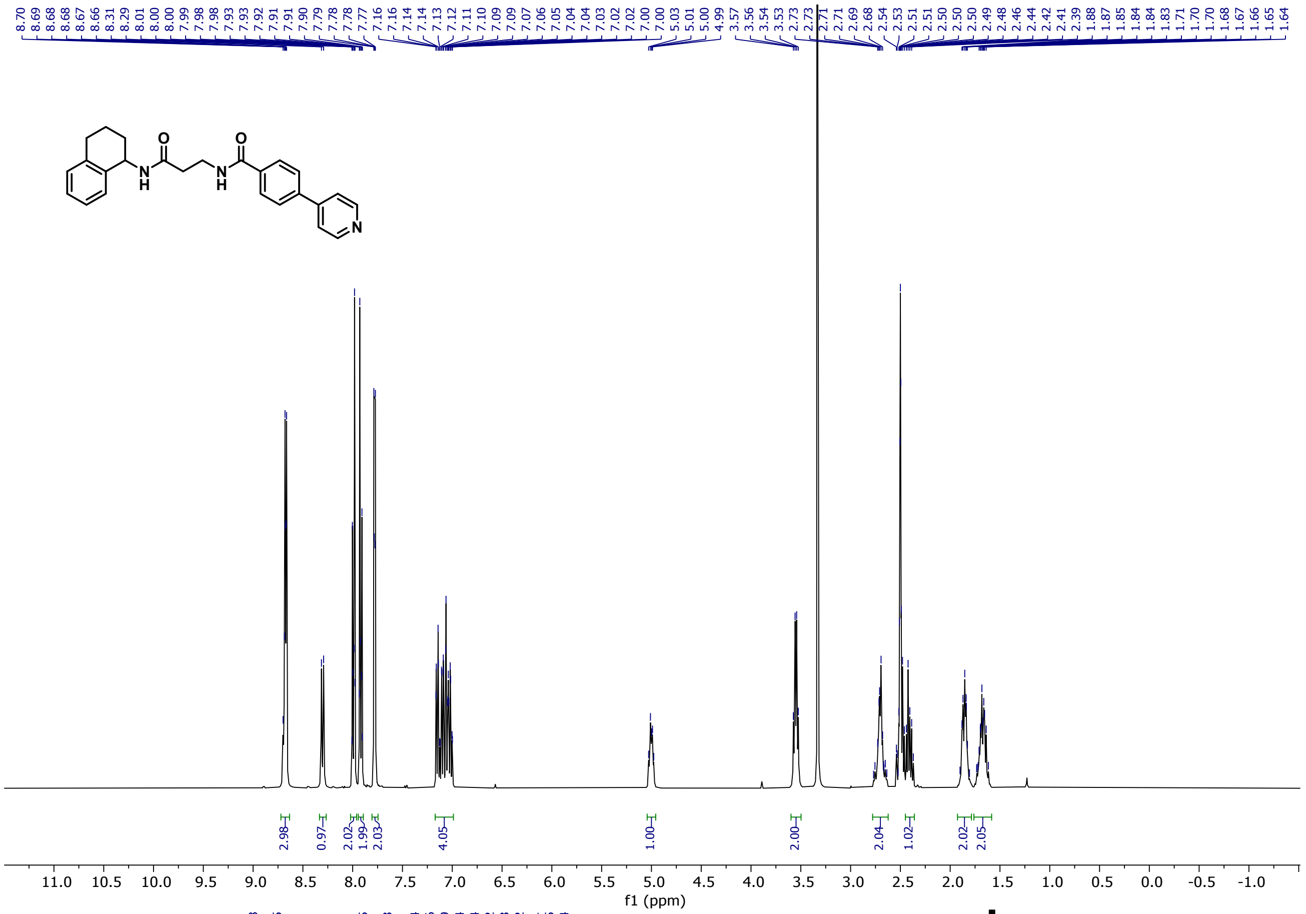

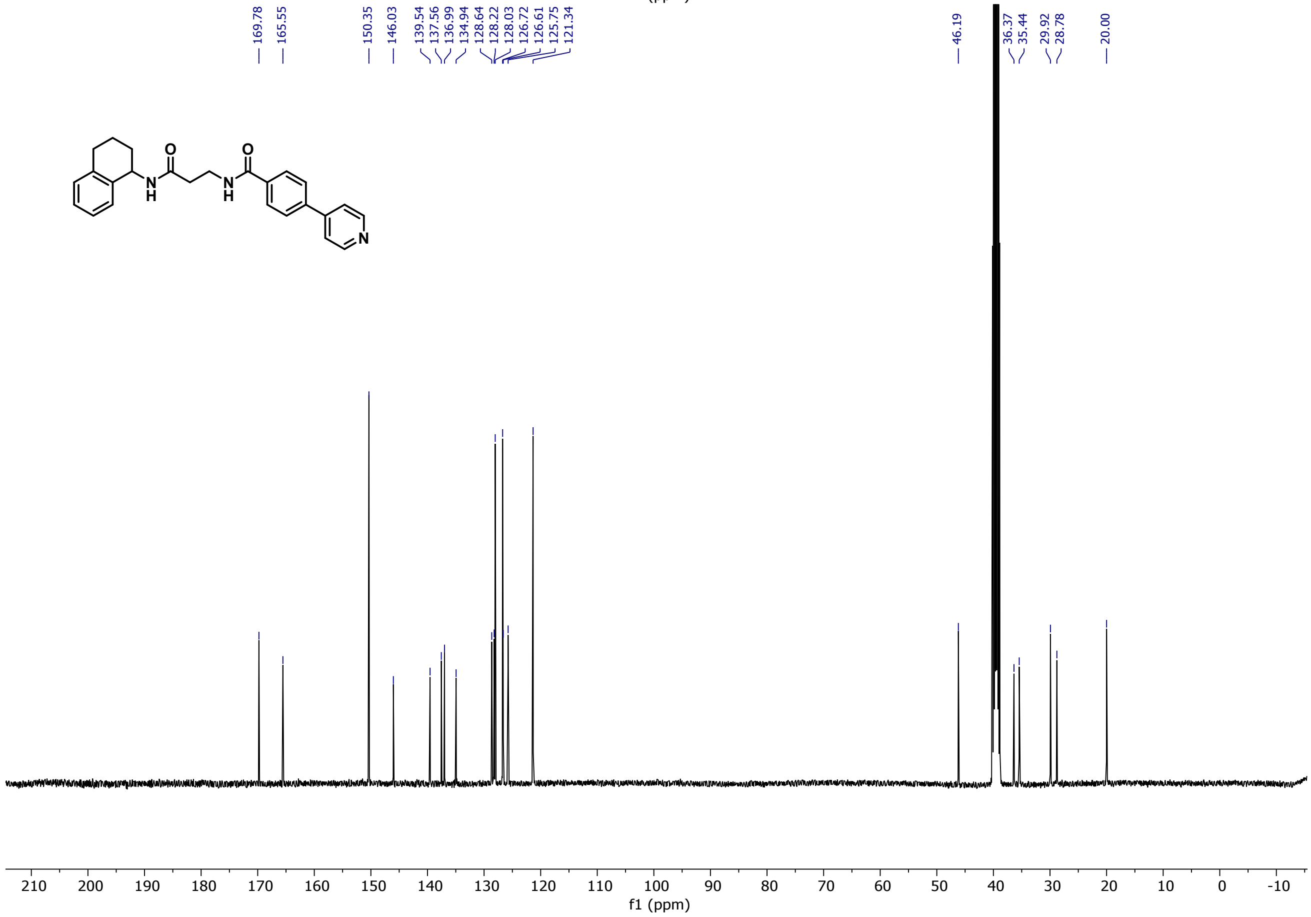

NMR spectra of 9d (VU6071680) in DMSO-*d₆*.

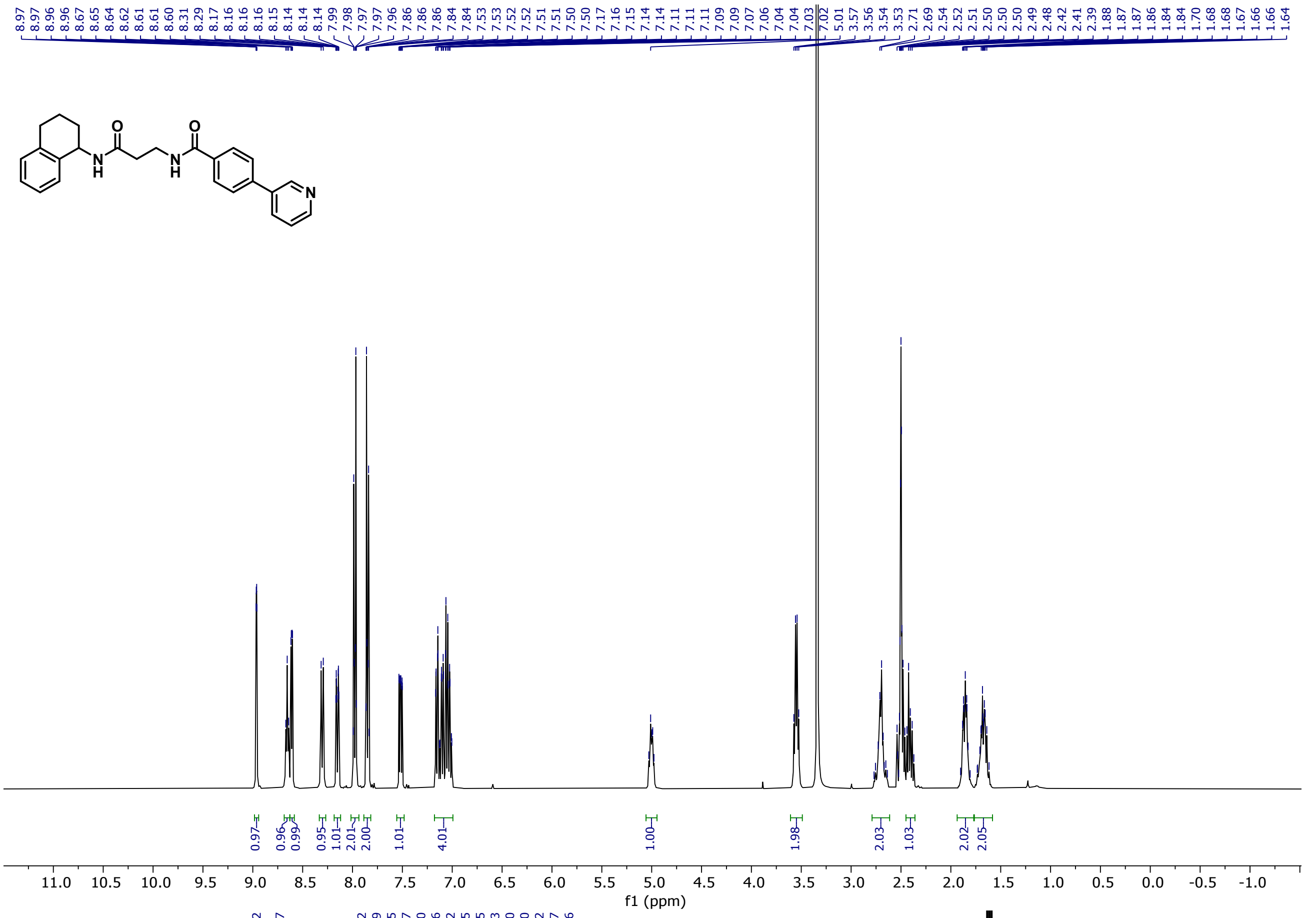

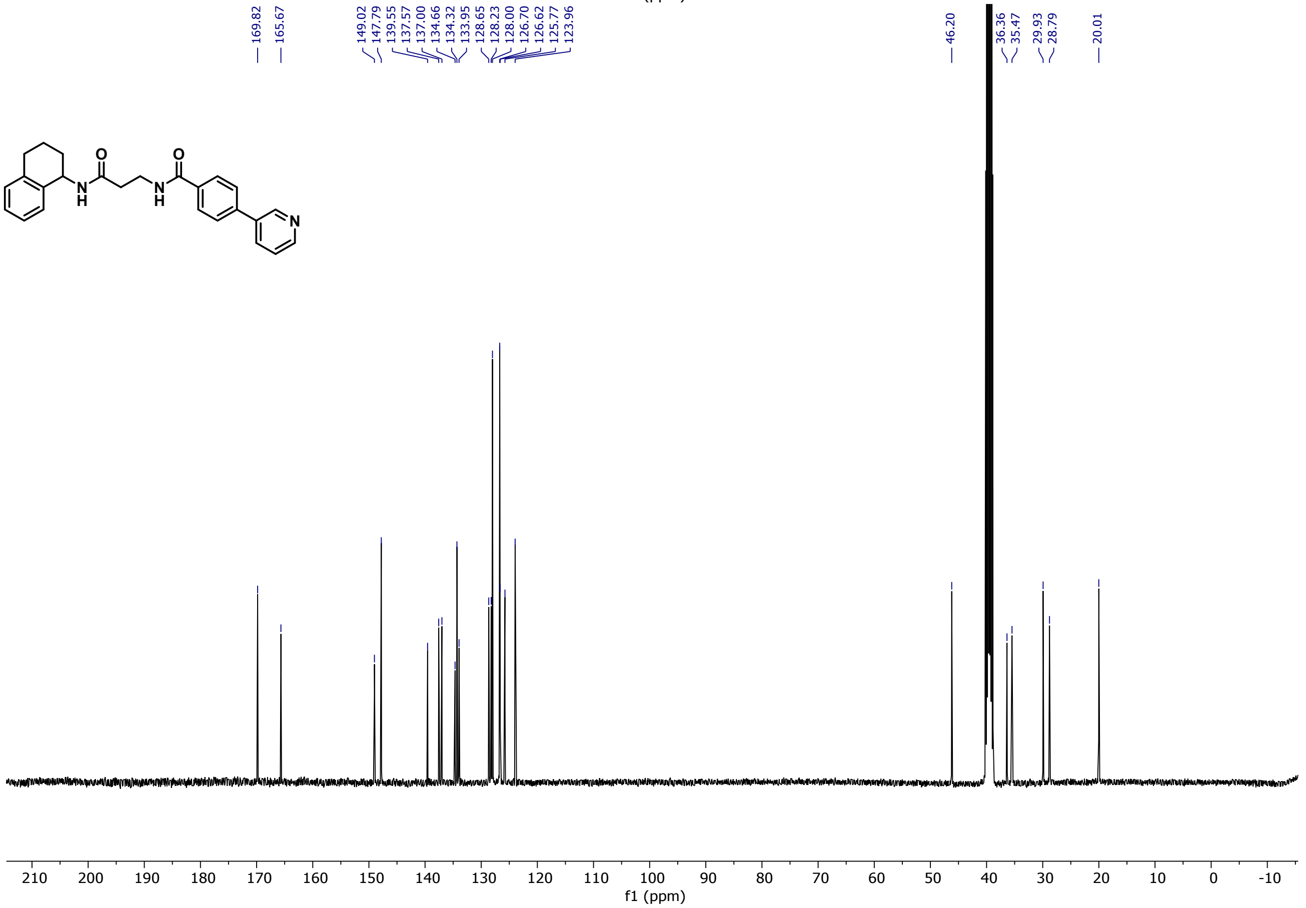

NMR spectra of 9e in DMSO-d_6.

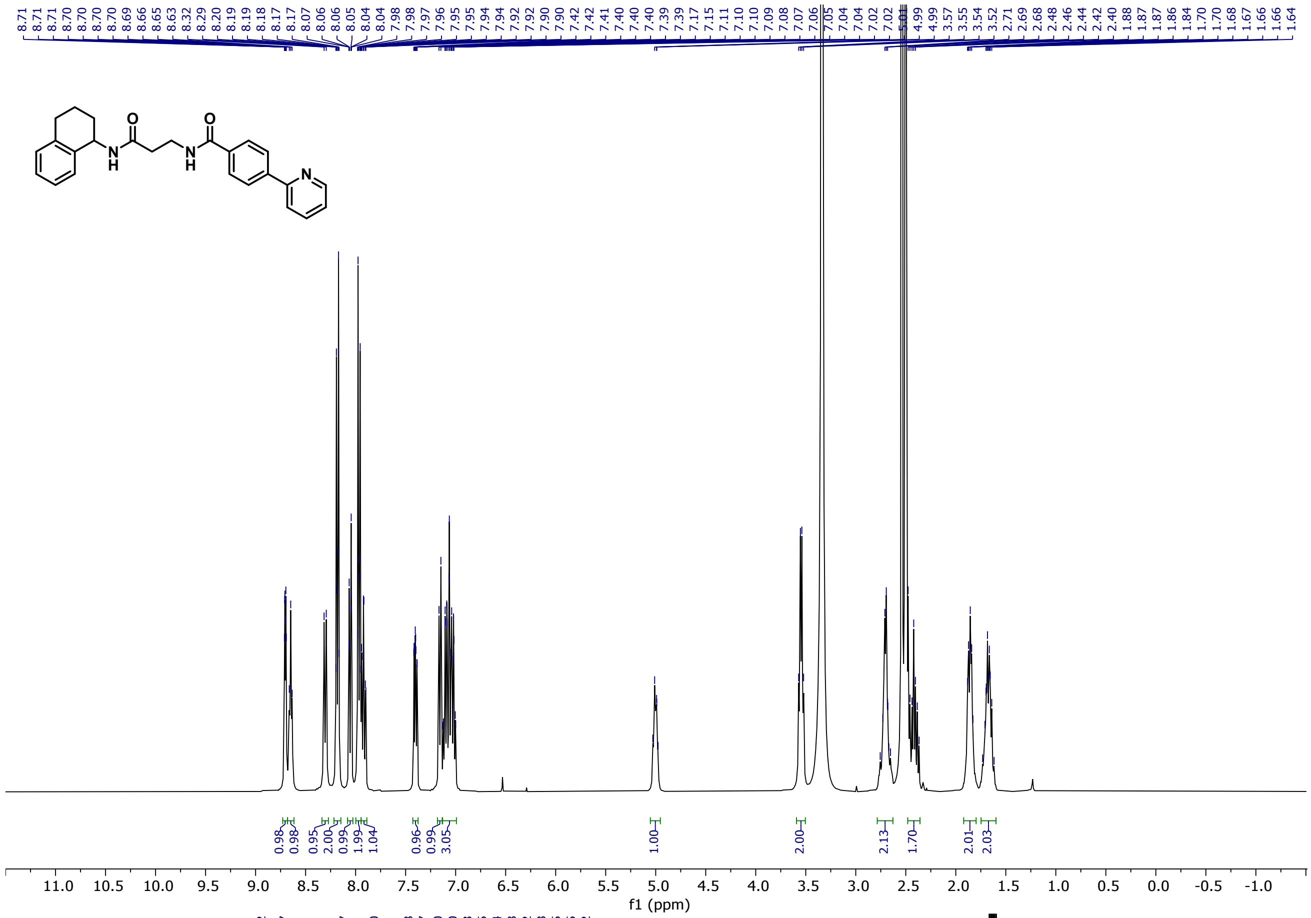

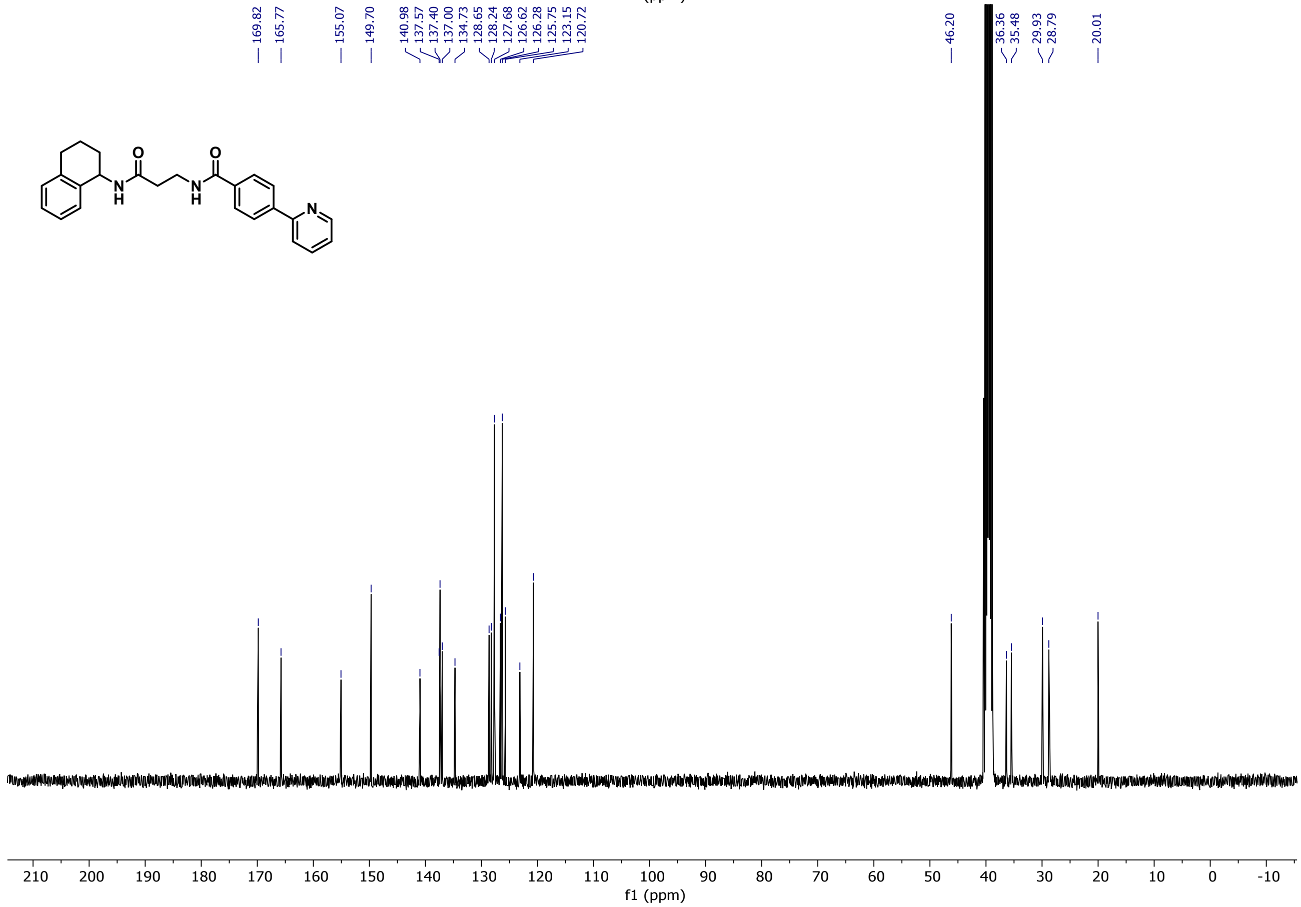

NMR spectra of 9f in DMSO-d_6.

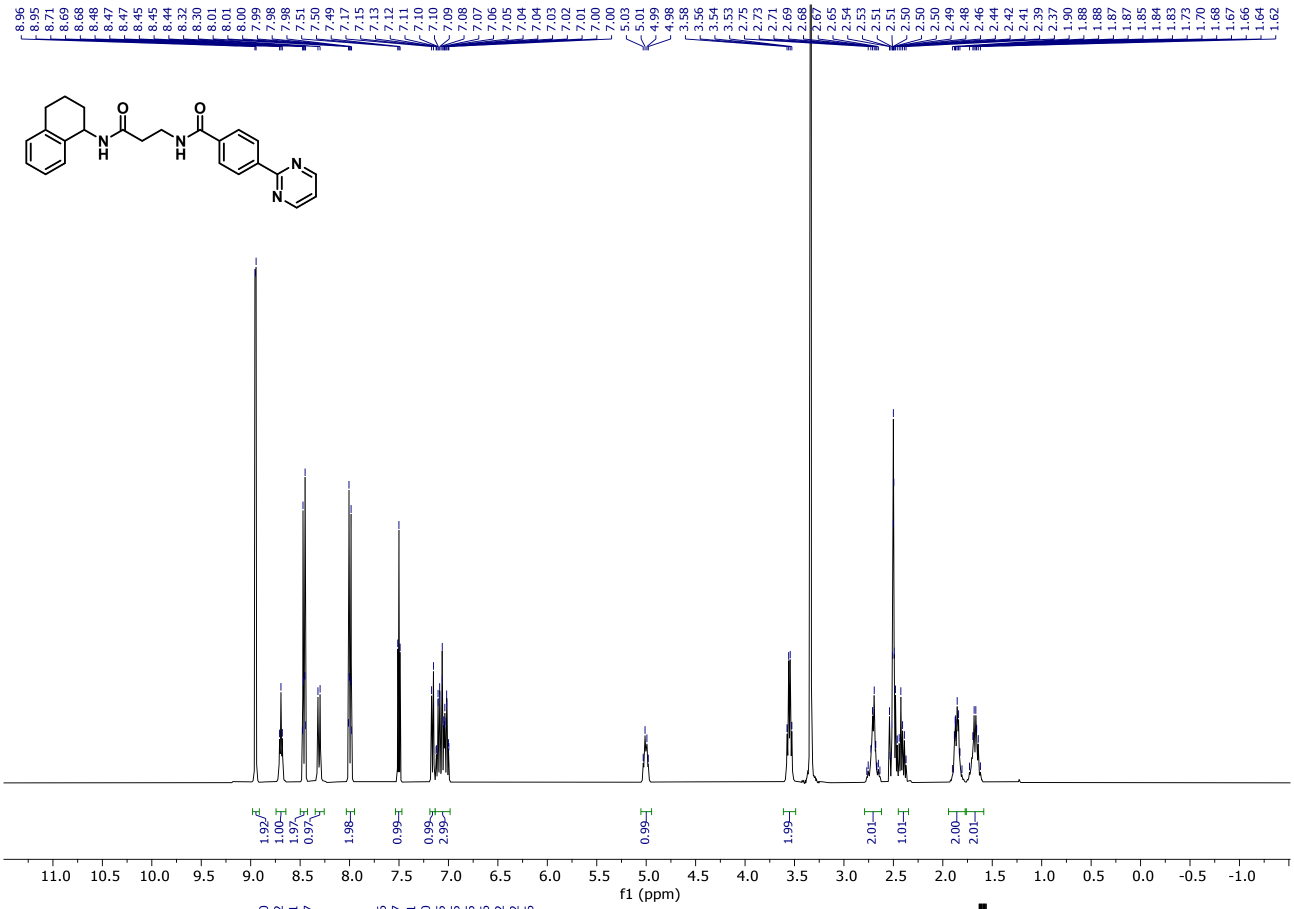

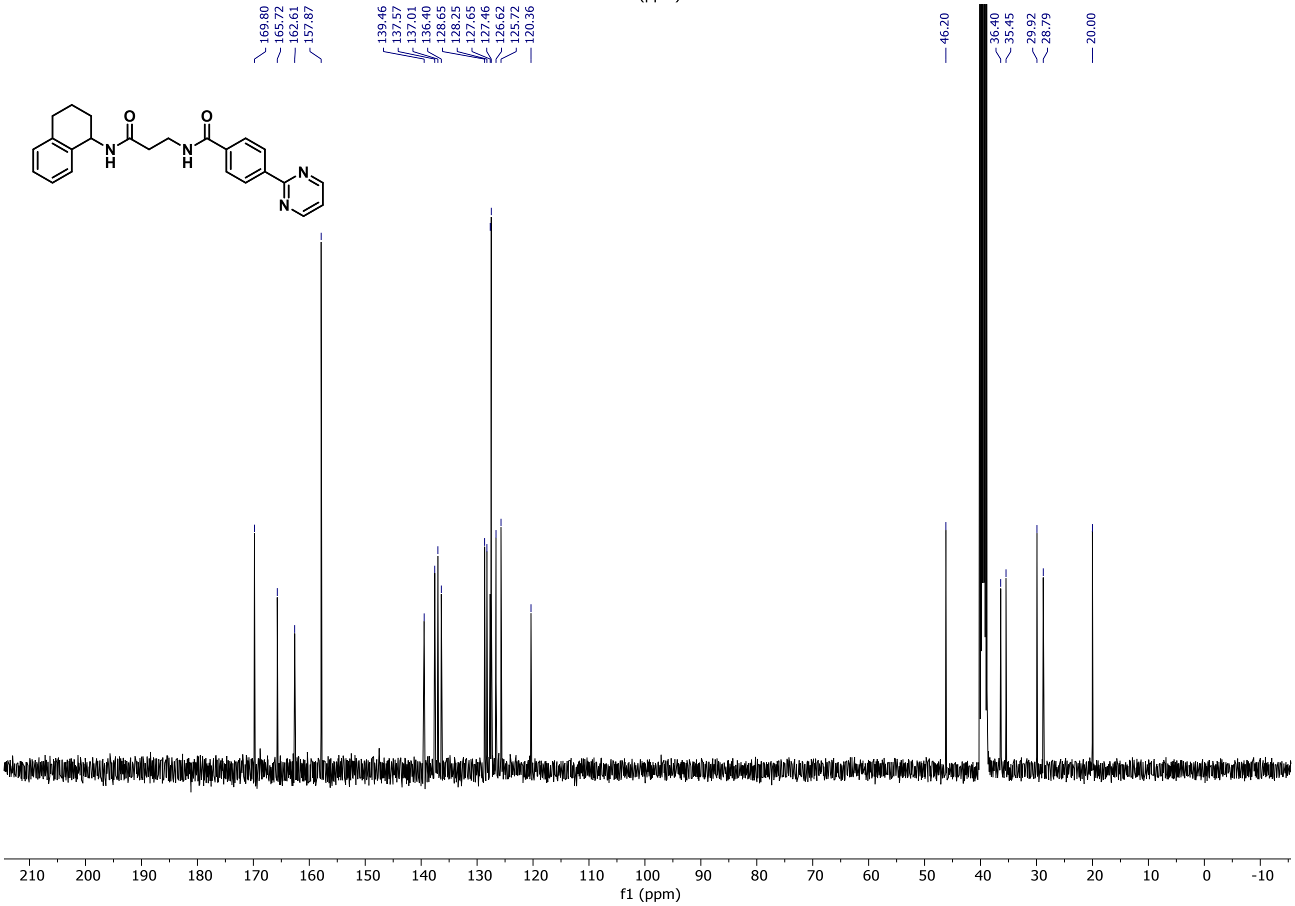

NMR spectra of 9g in DMSO-d_6.

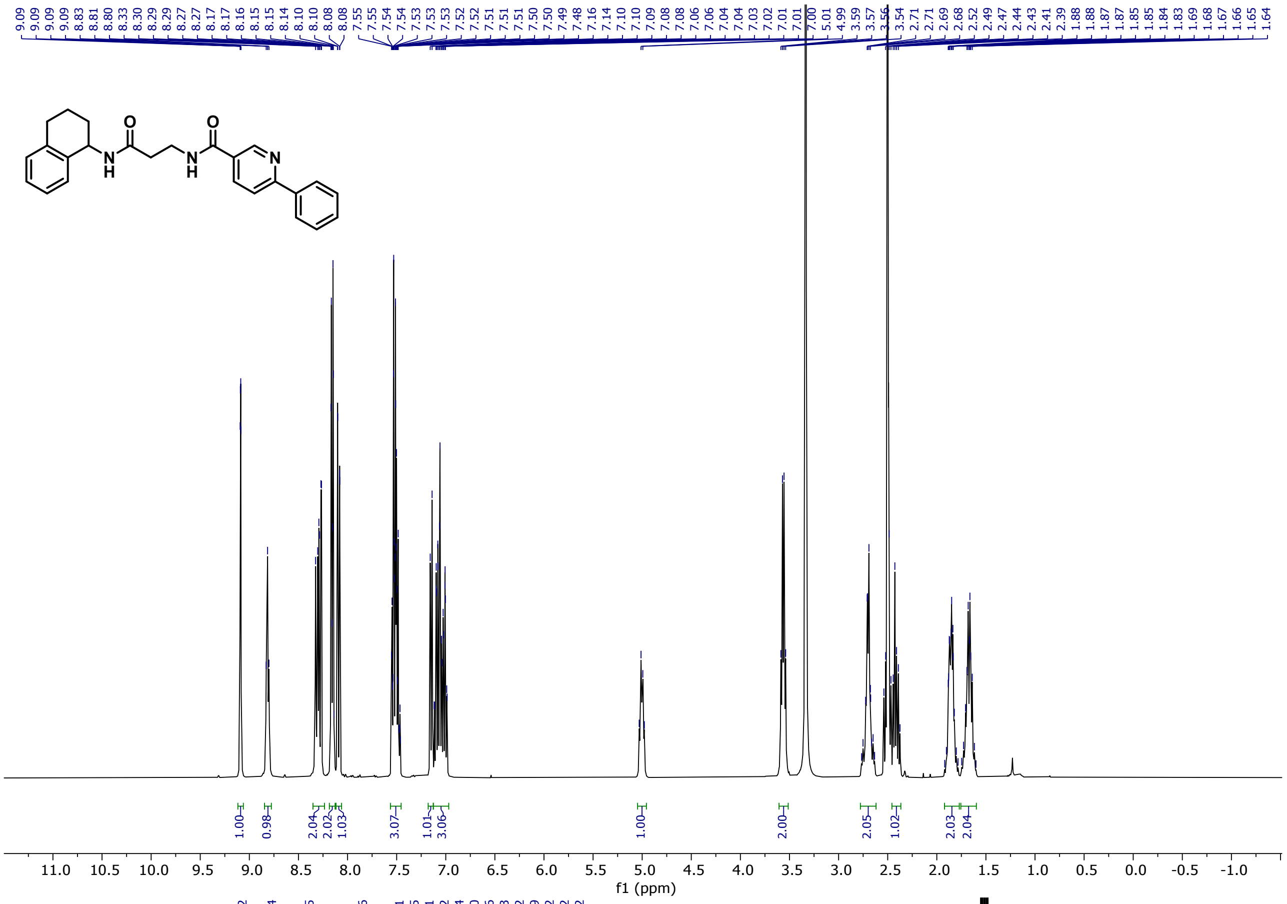

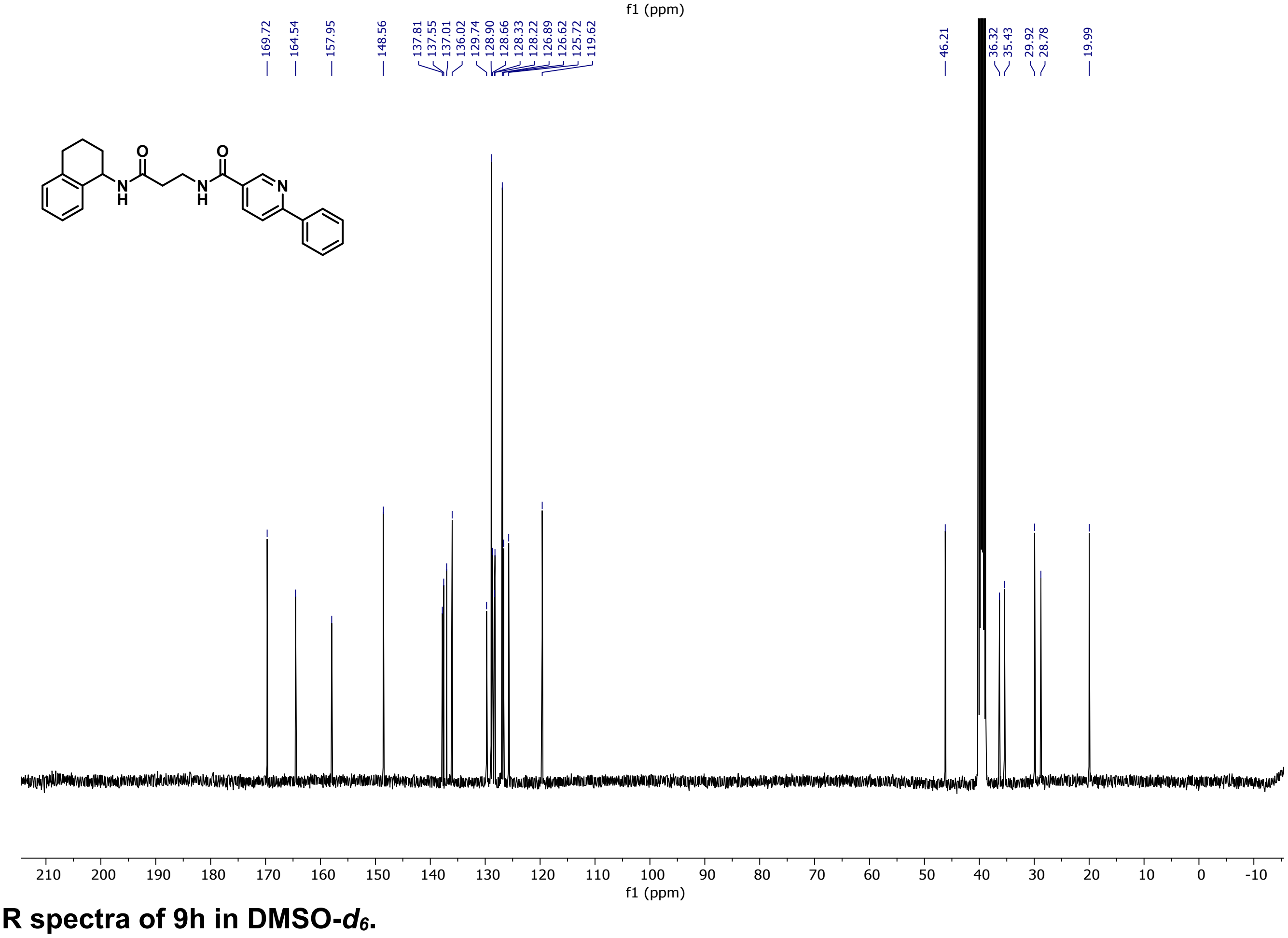

NMR spectra of 9h in DMSO-d_6.

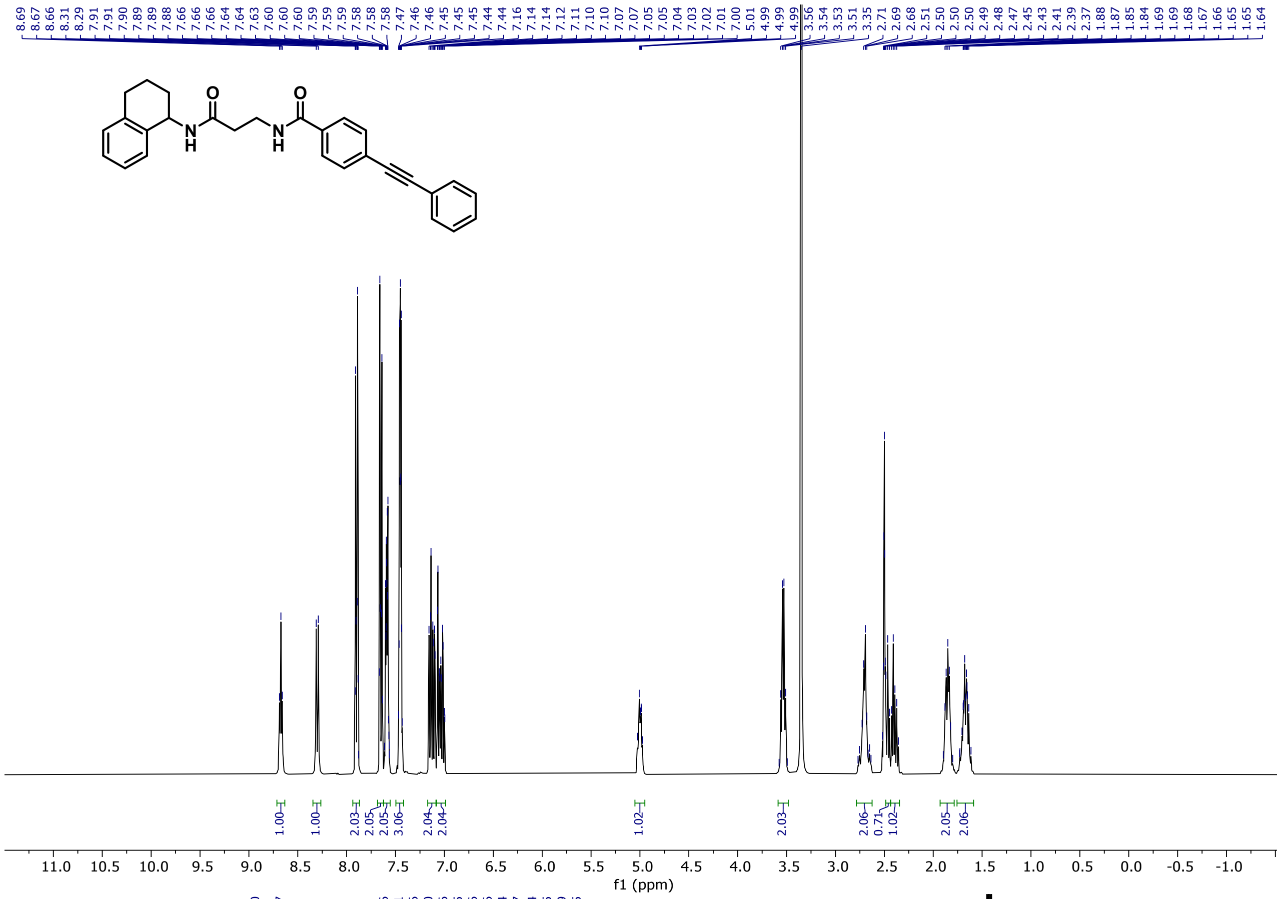

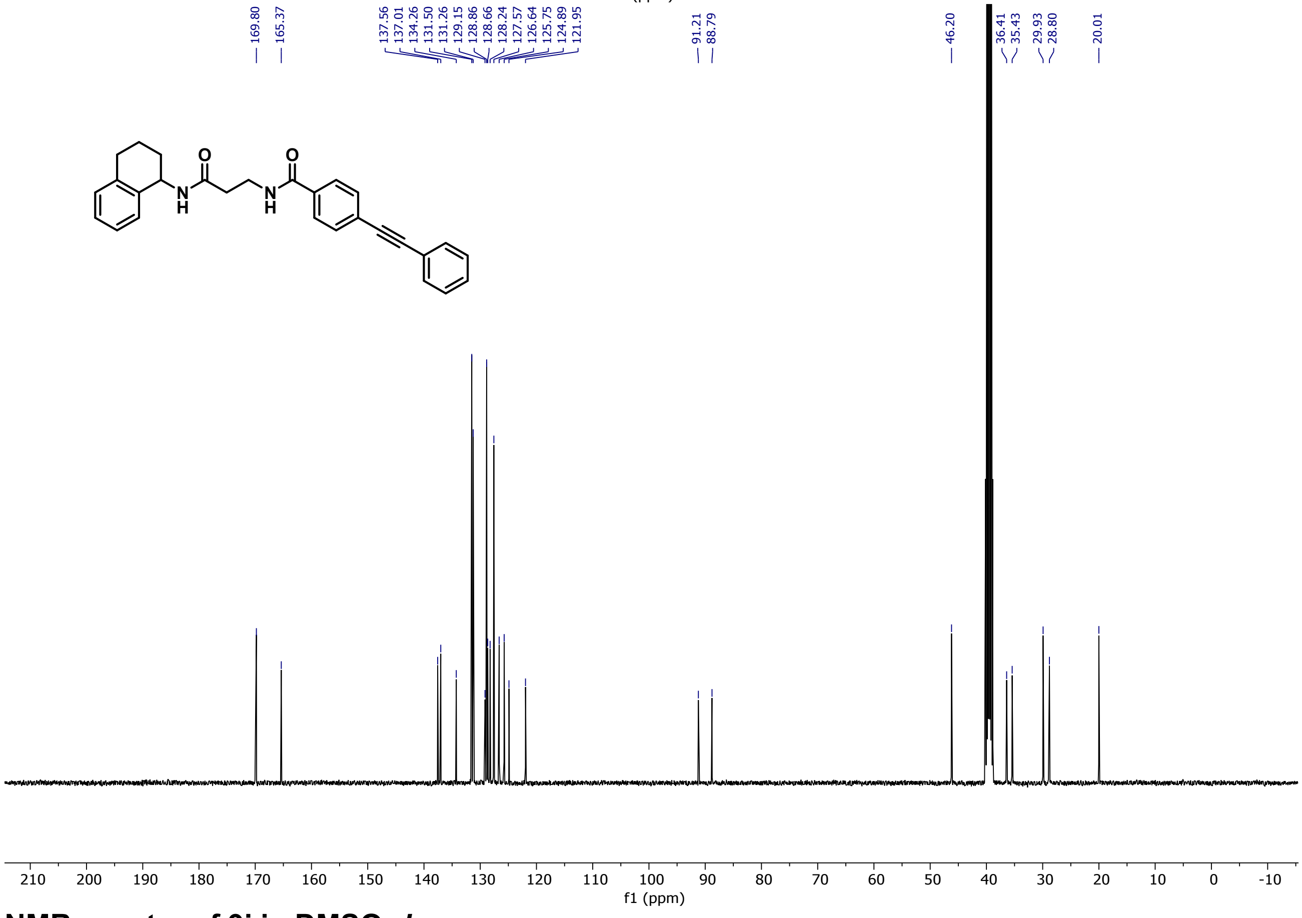

NMR spectra of 9i in DMSO-d_6.

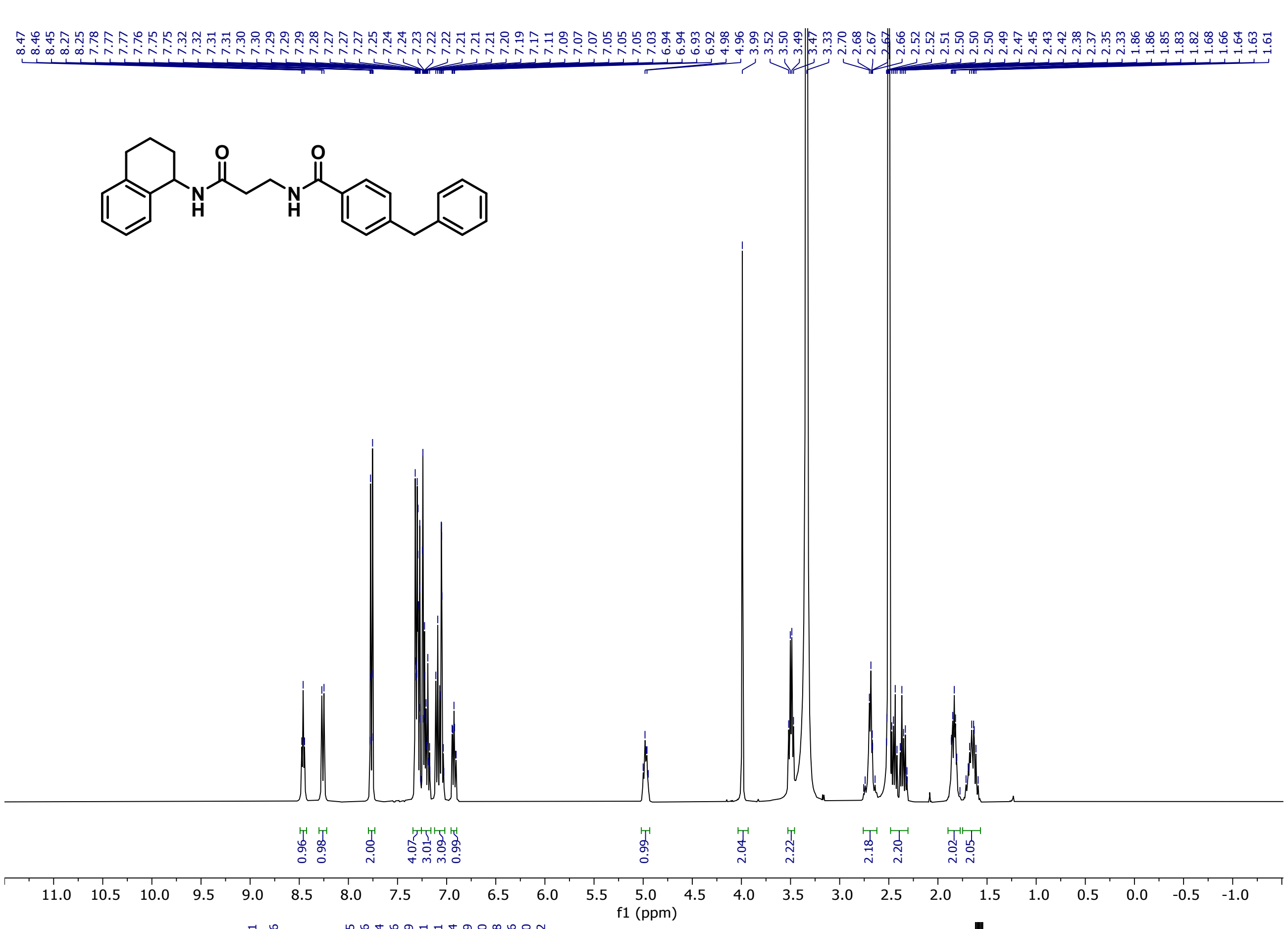

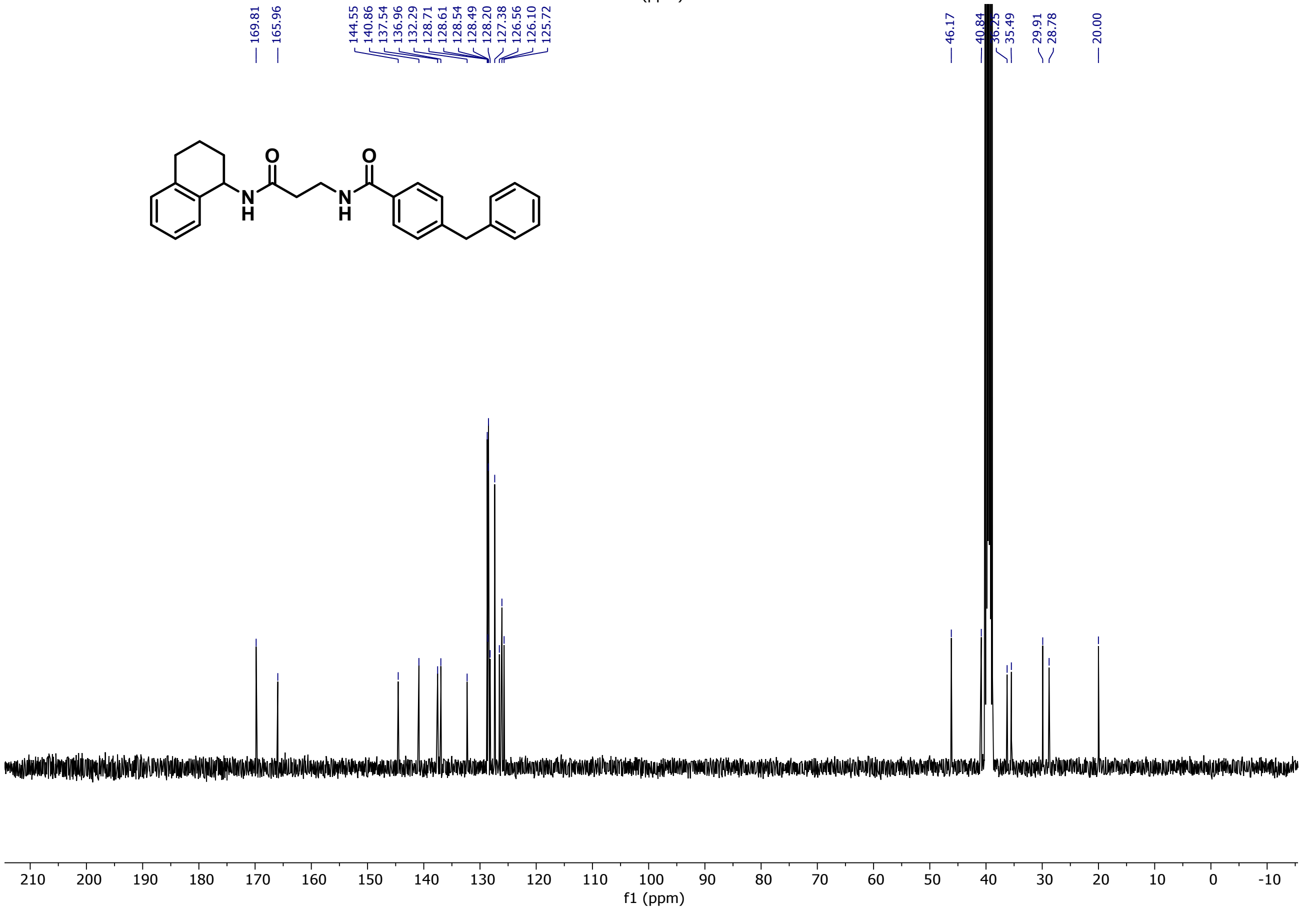

NMR spectra of 9j in DMSO-d_6.

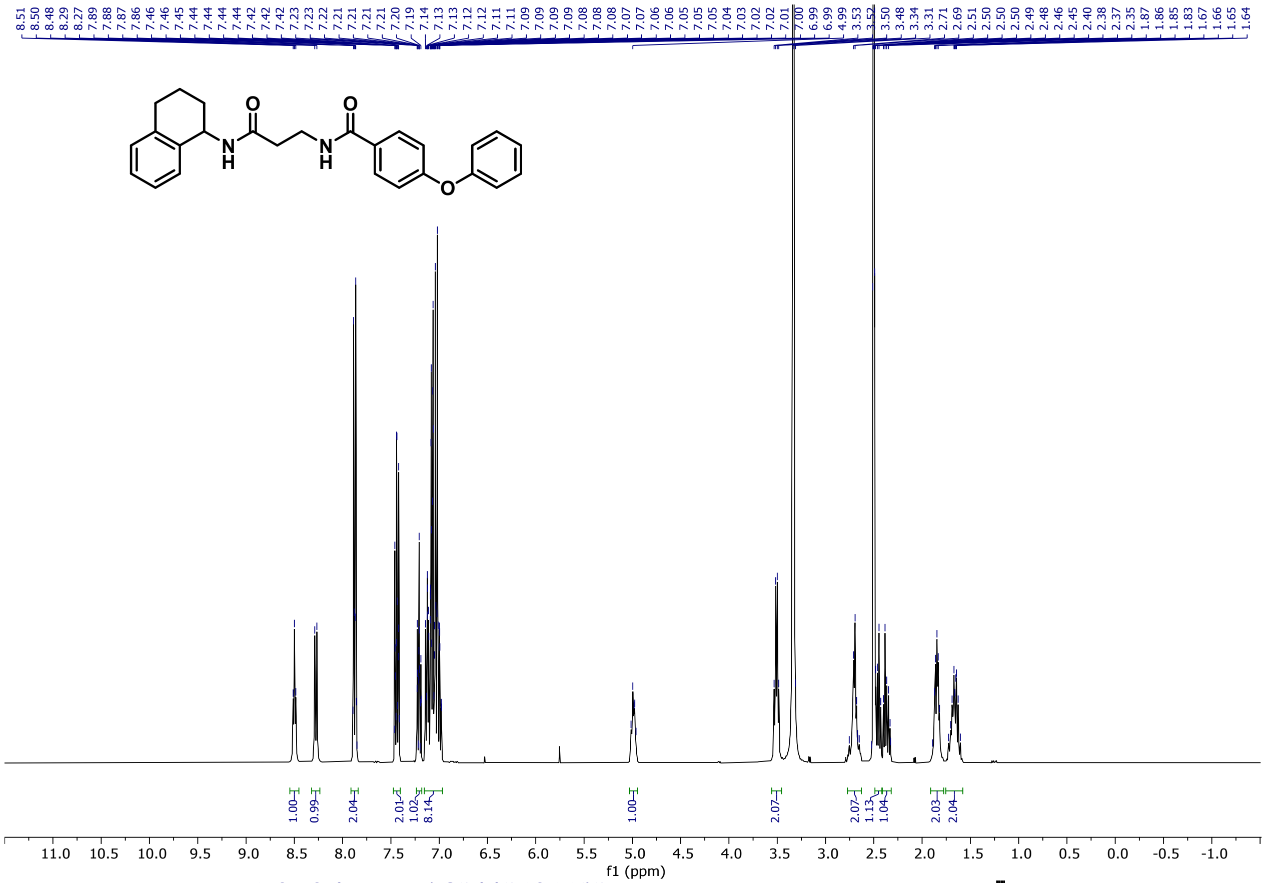

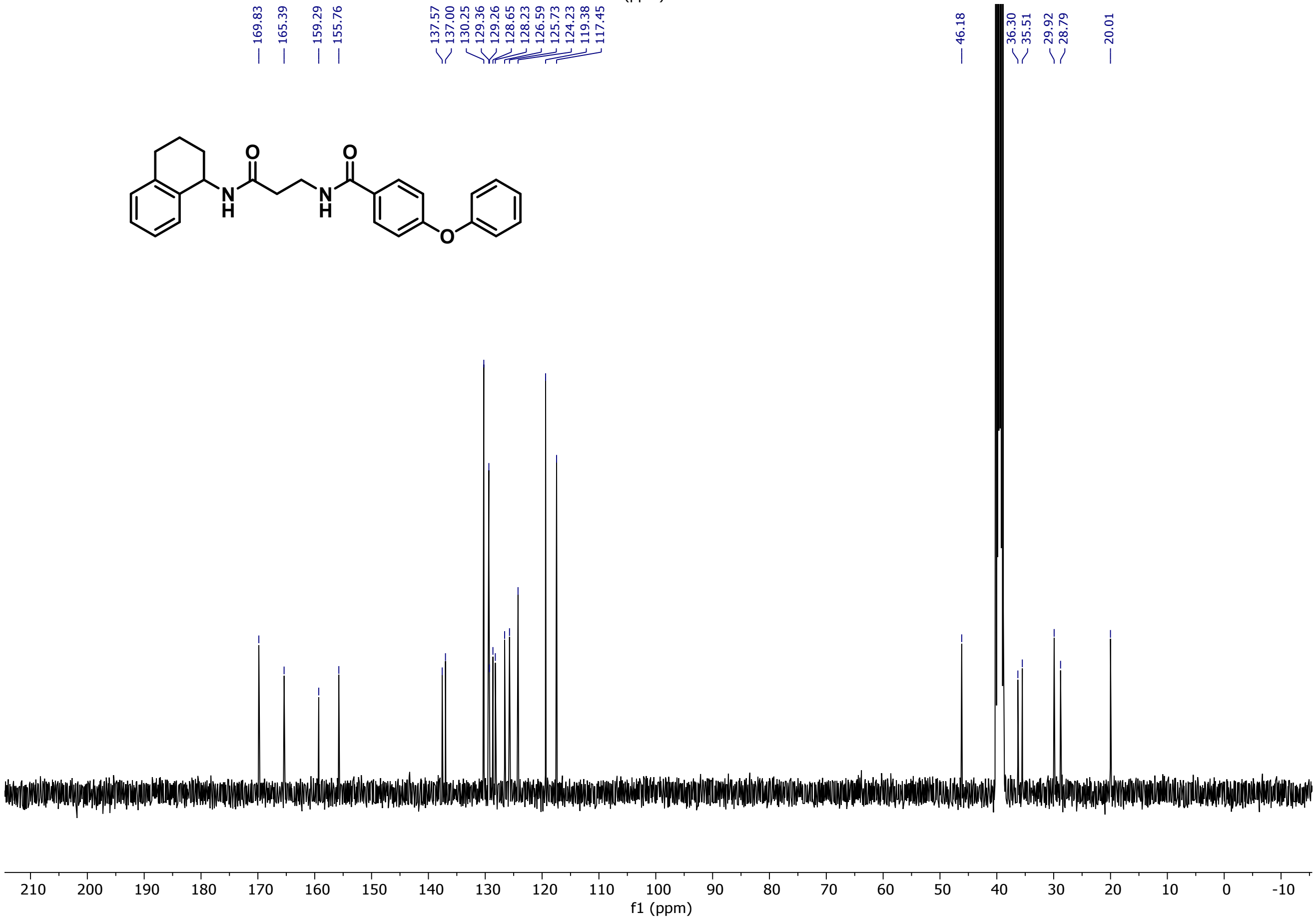

NMR spectra of 9k in DMSO-d_6.

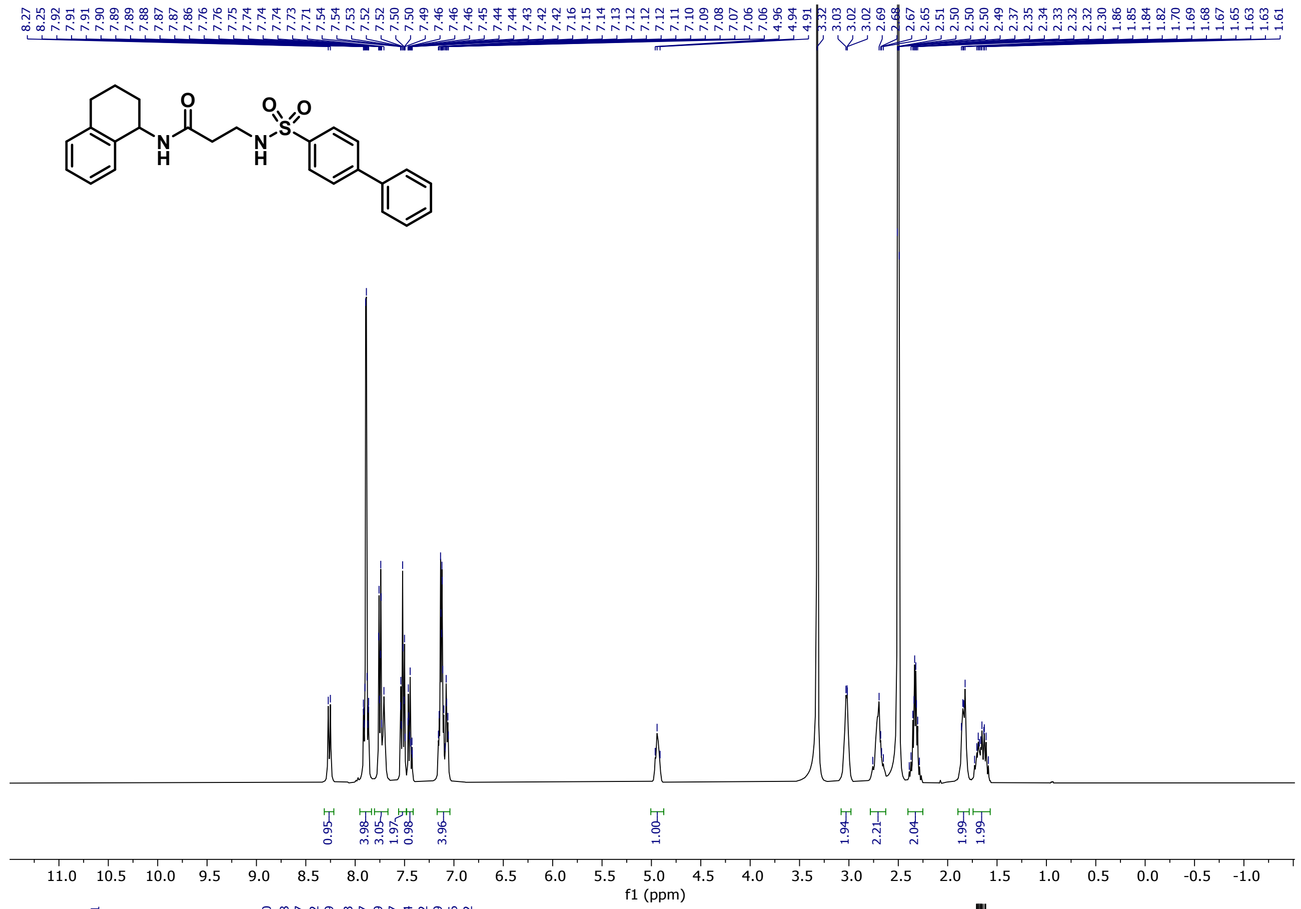

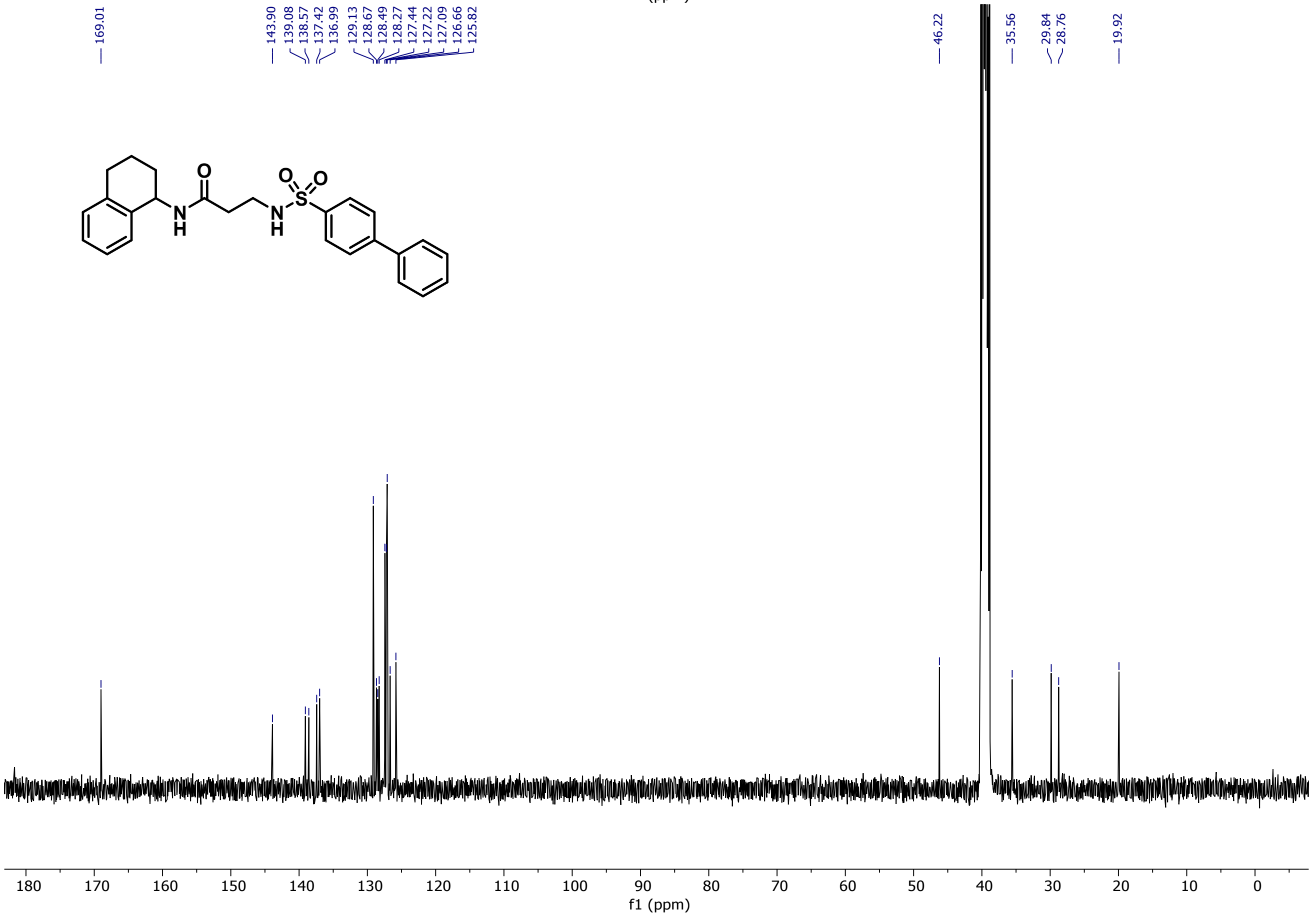

NMR spectra of 9l in DMSO-d_6.

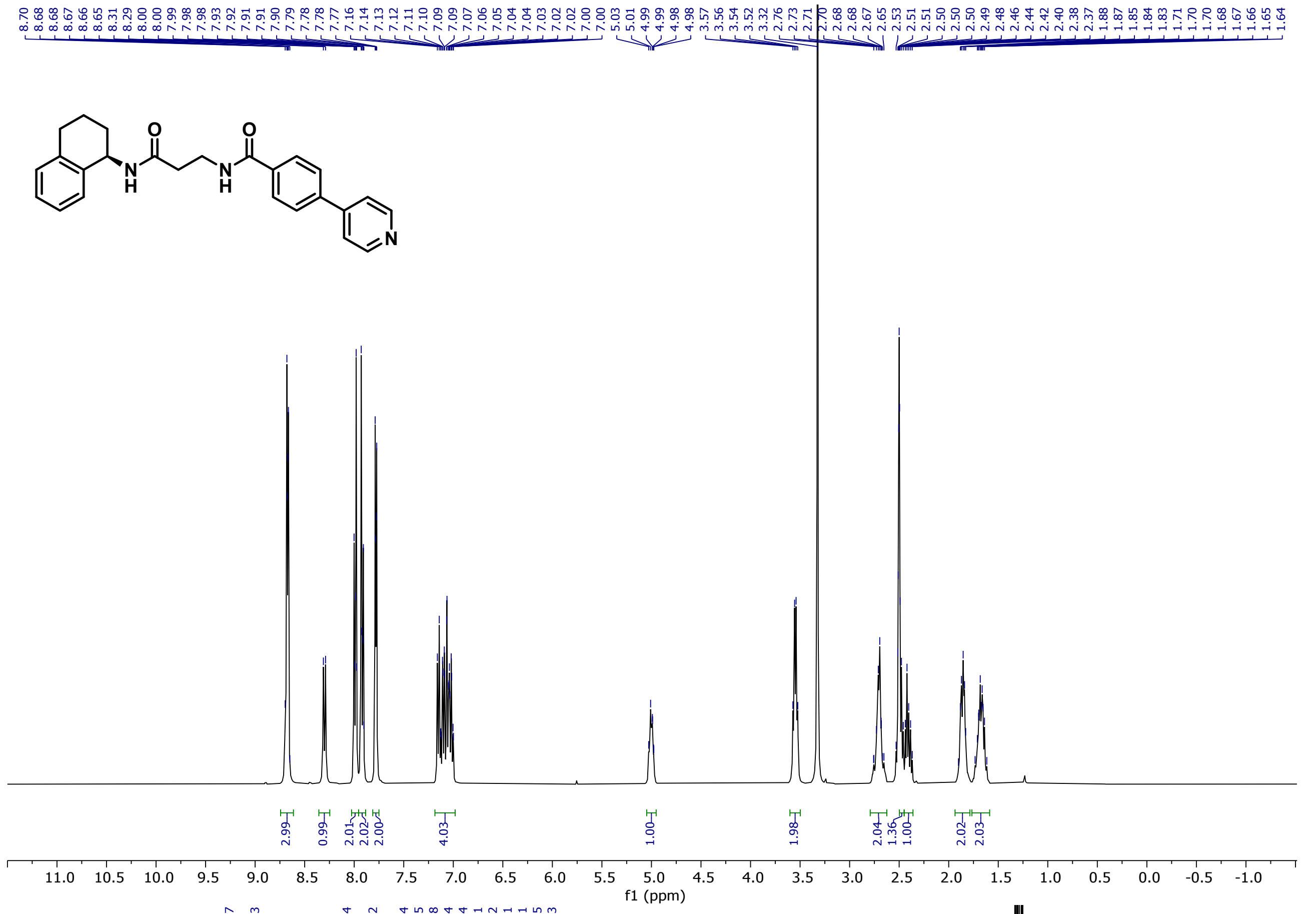

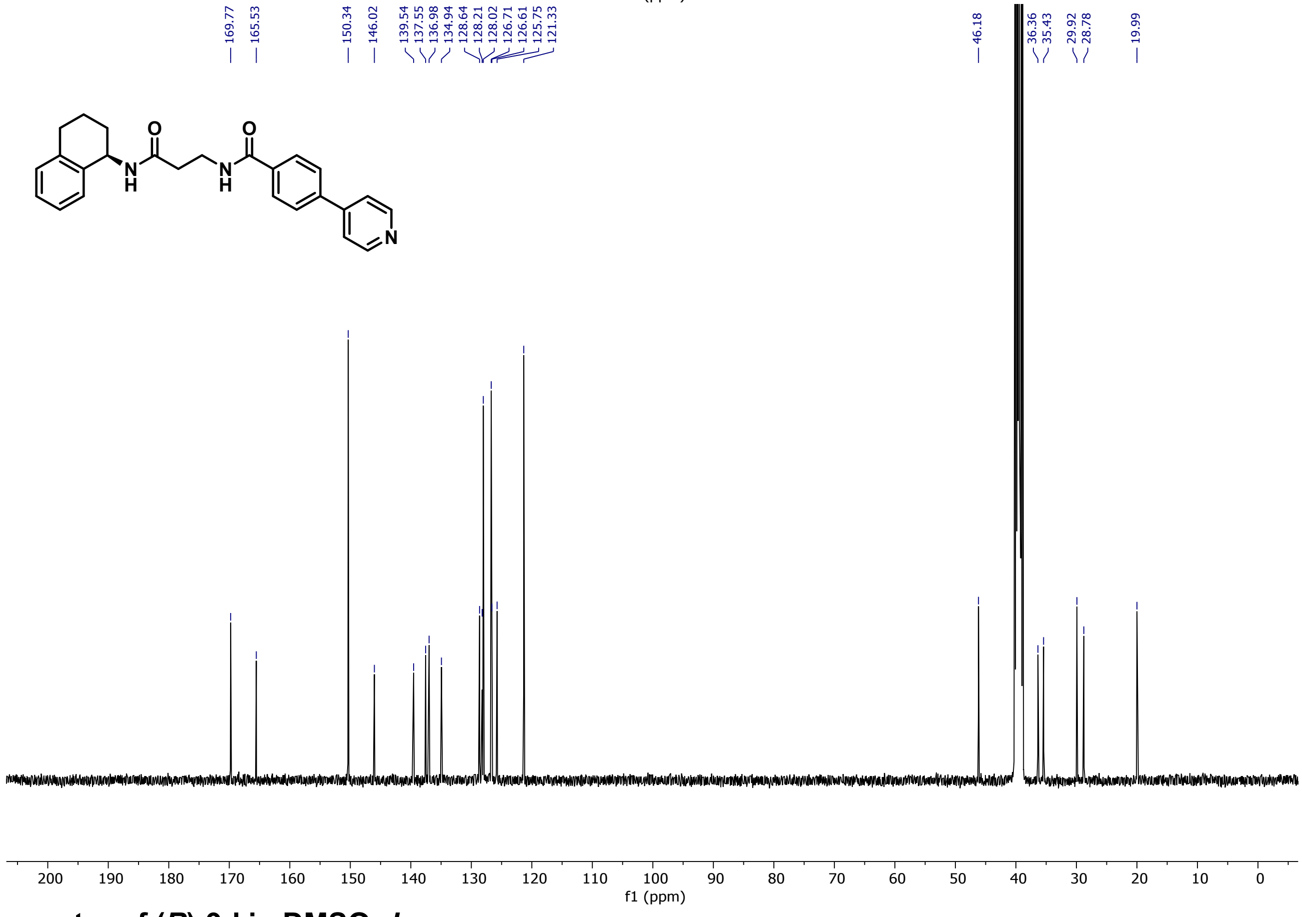

NMR spectra of (*R*)-9d in DMSO-*d*$_6$.

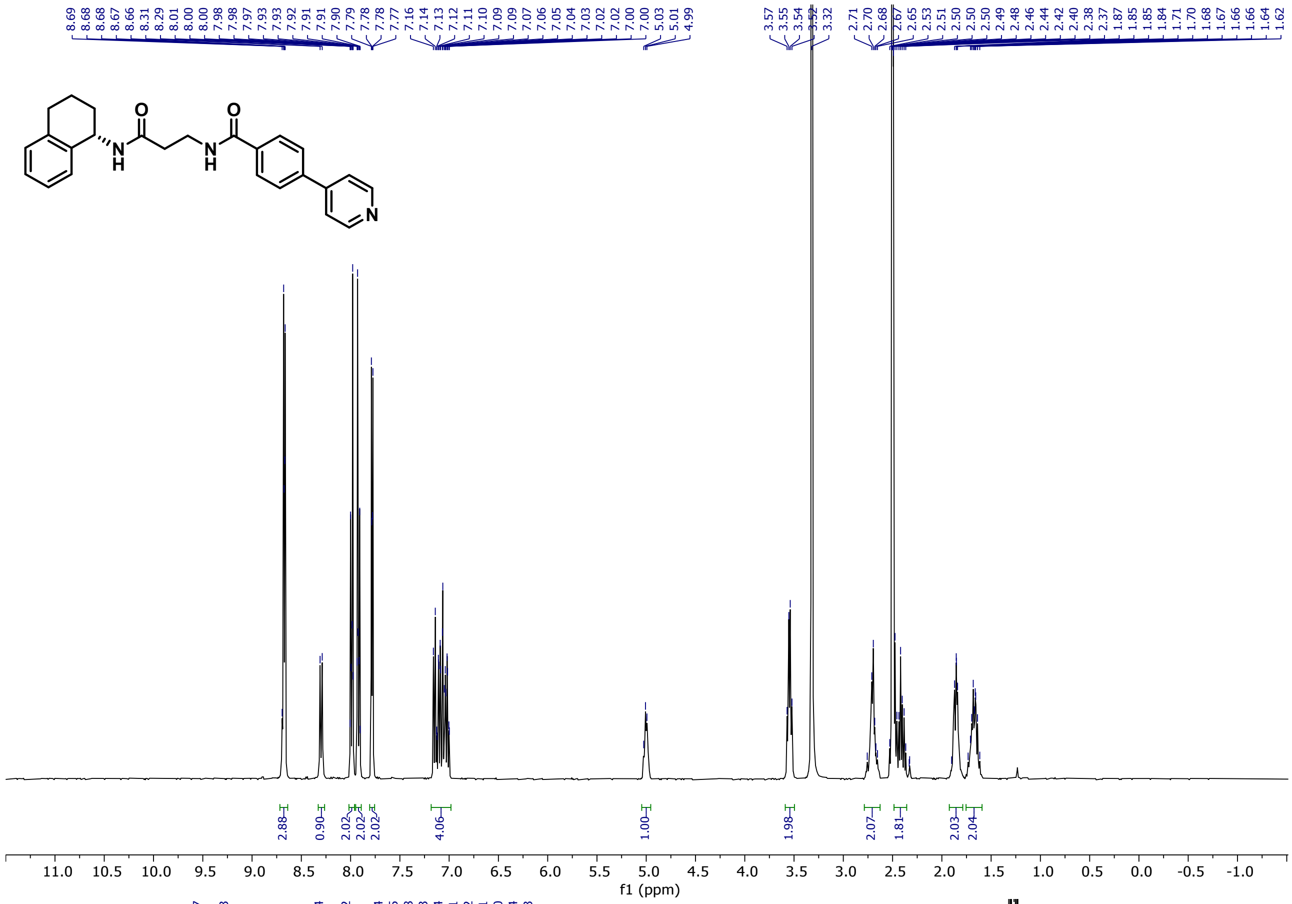

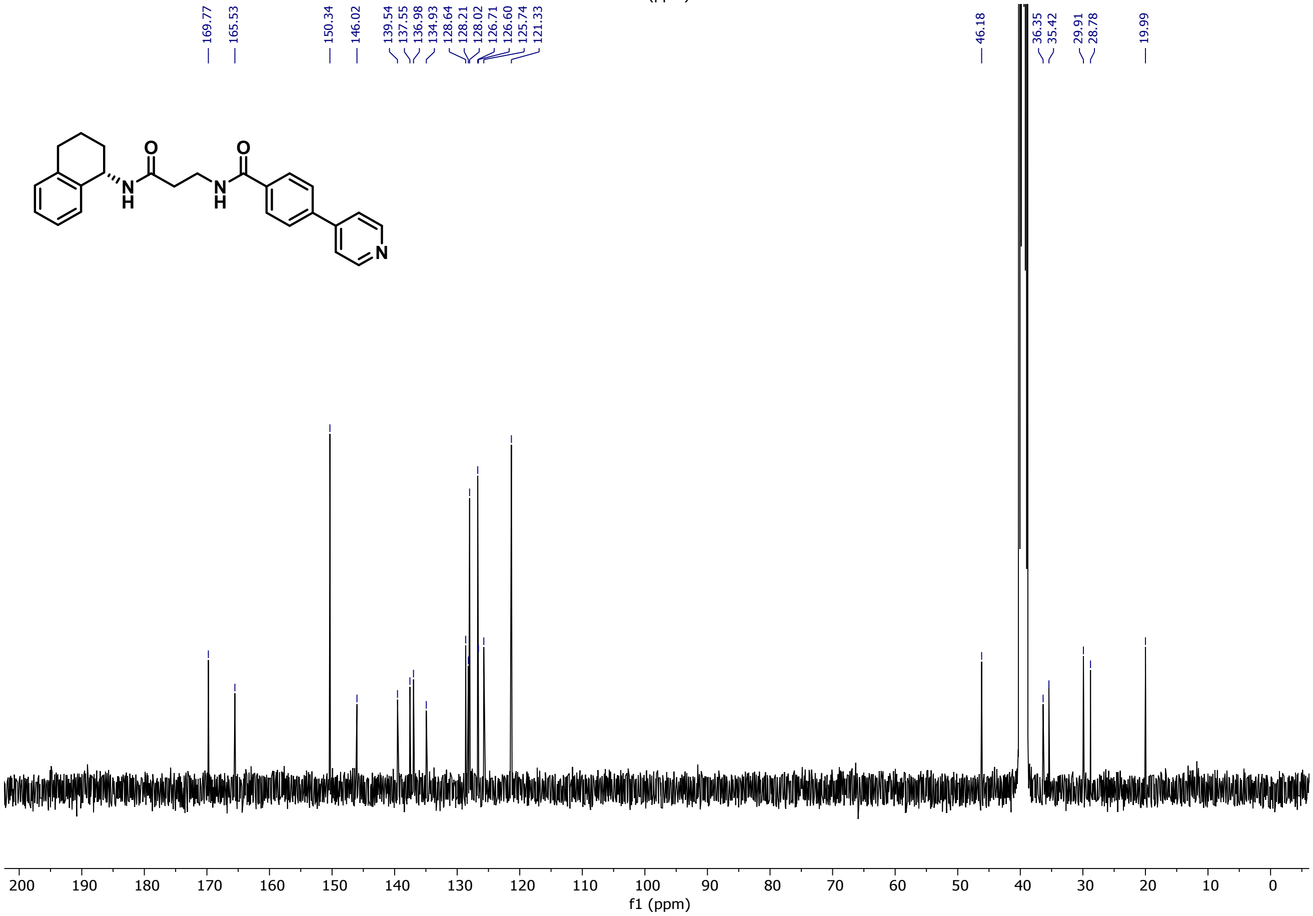

NMR spectra of (*S*)-9d in DMSO-d_6.

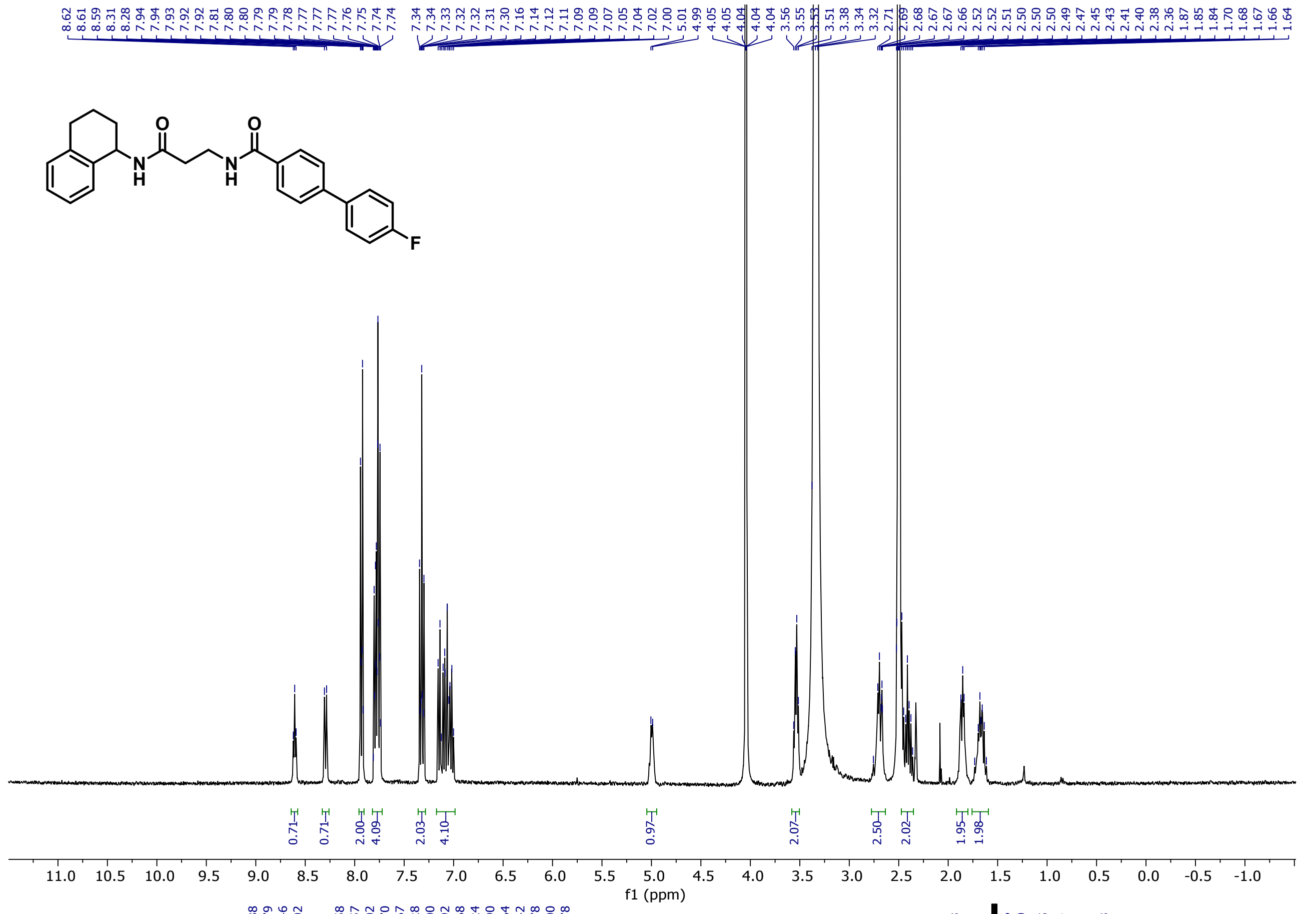

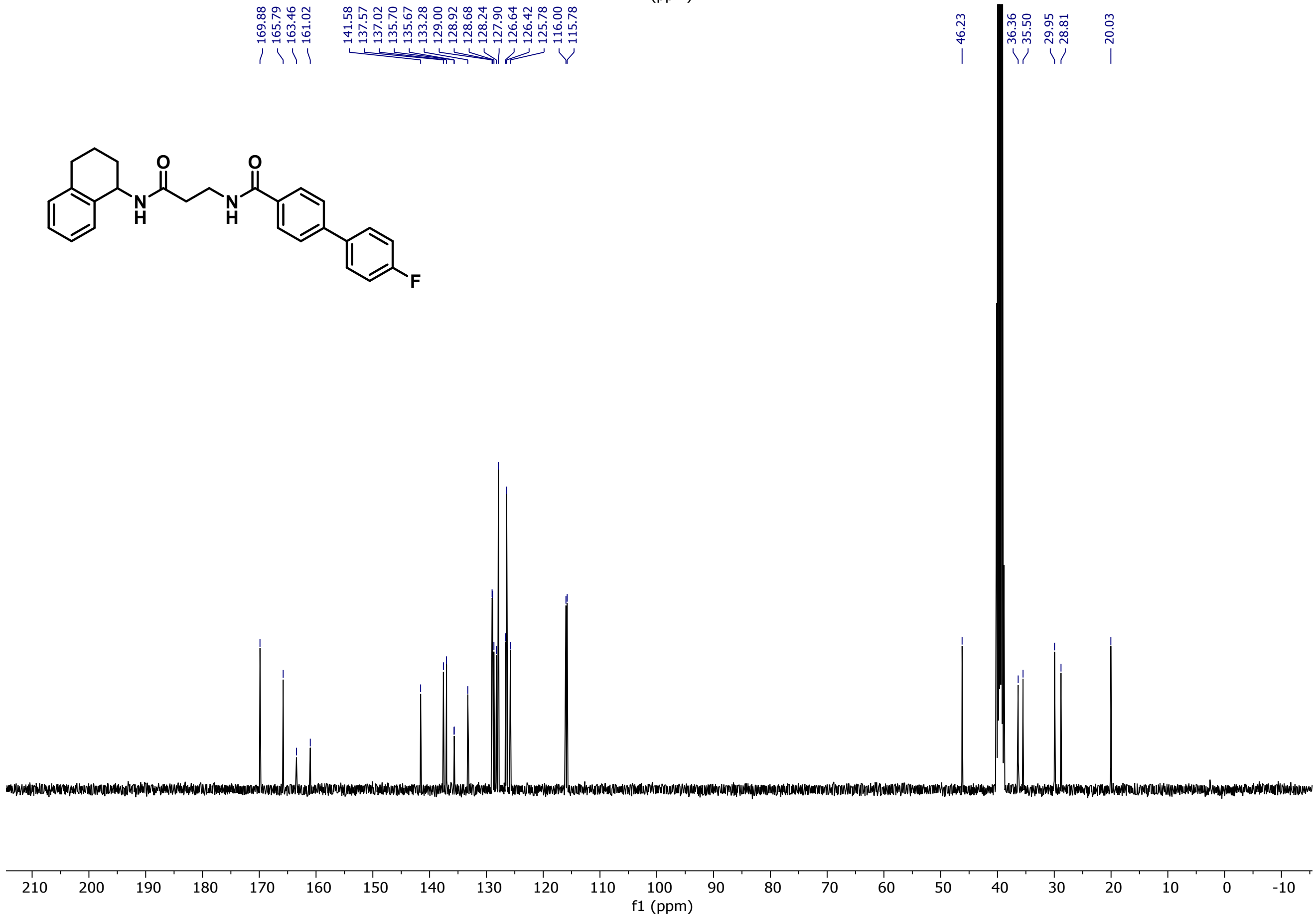

NMR spectra of 10a in DMSO-*d*$_6$.

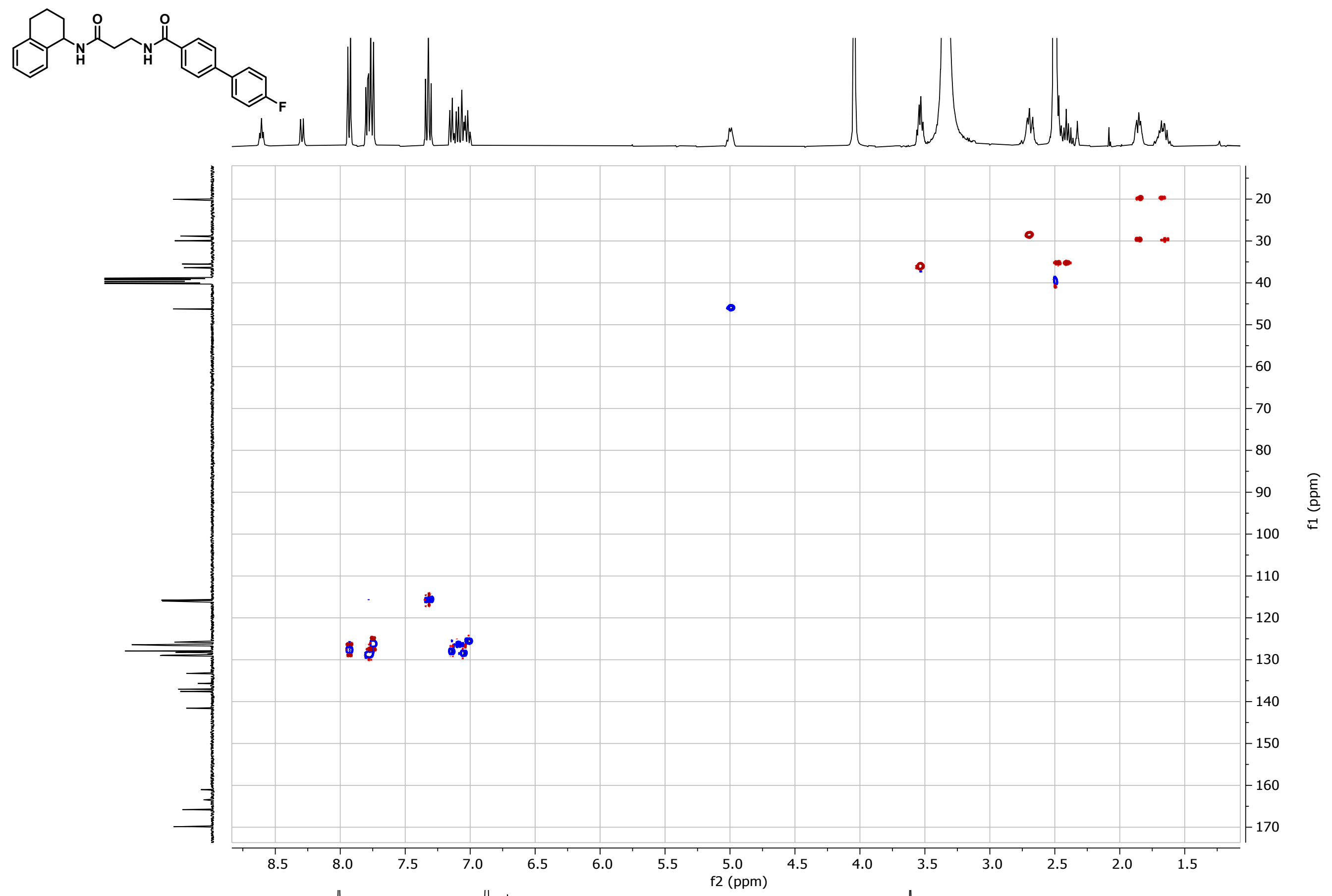

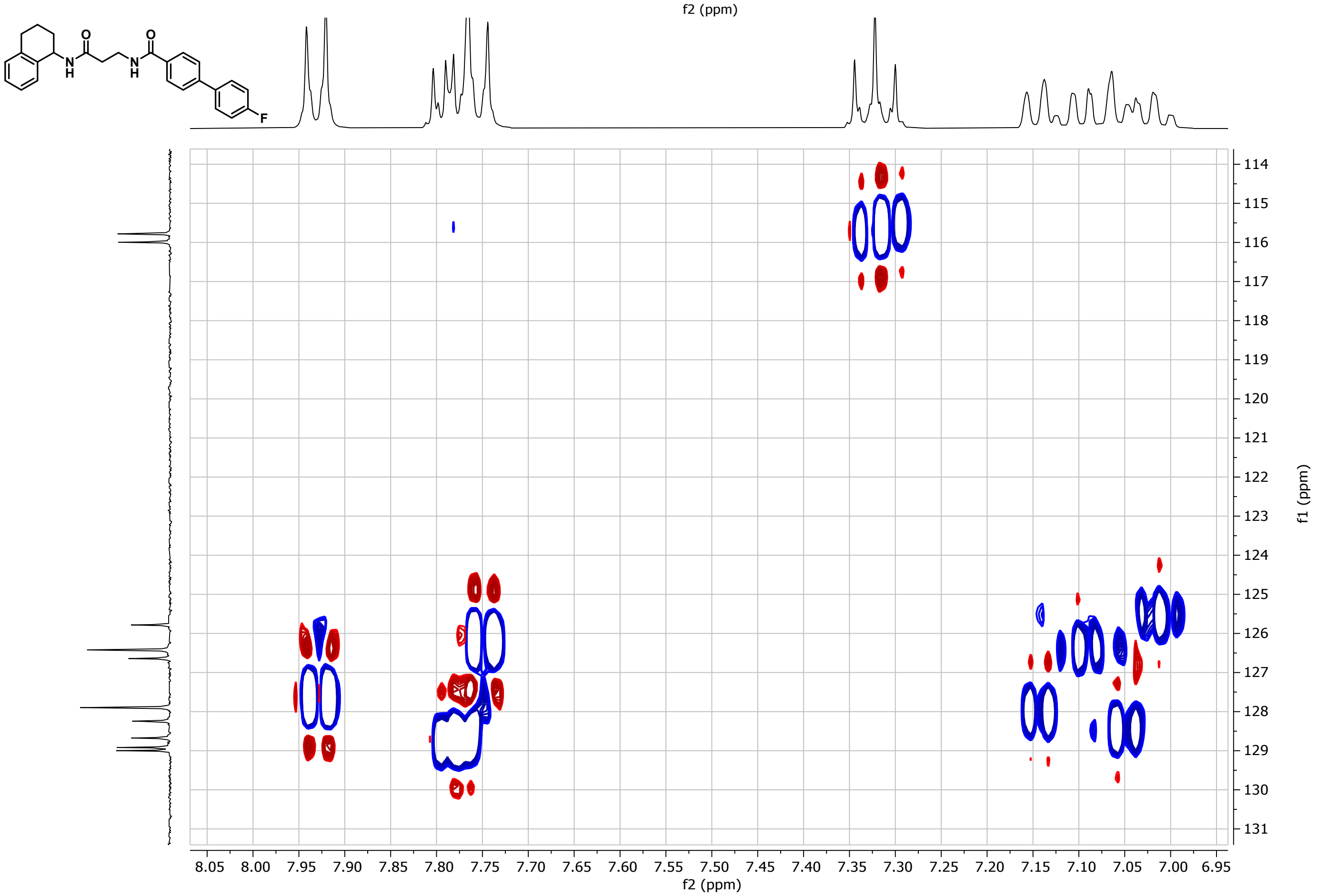

Full (top) and zoomed-in (bottom) HSQC spectrum of 10a in DMSO-d_6.

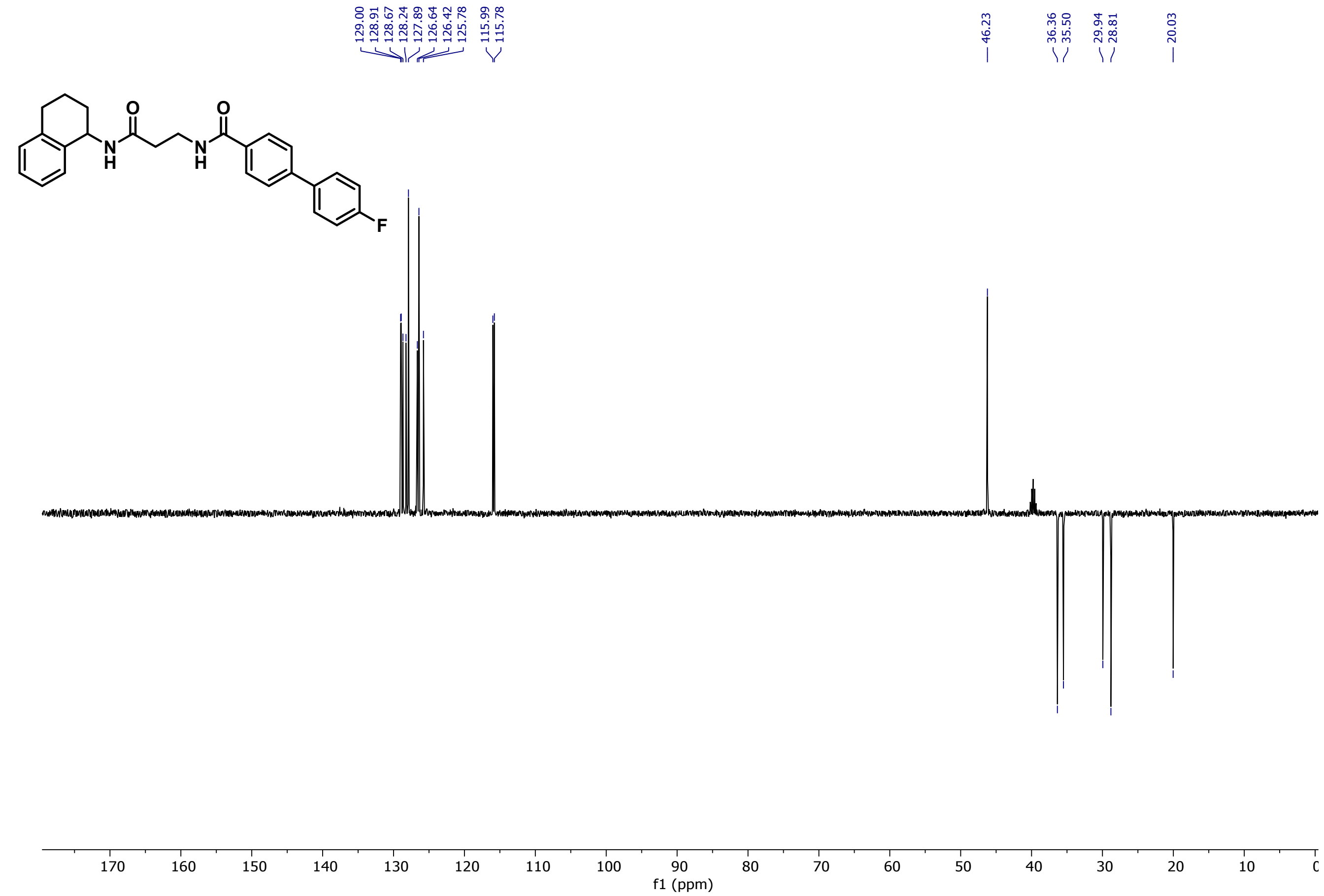

DEPT-135 spectrum of 10a in DMSO-d_6.

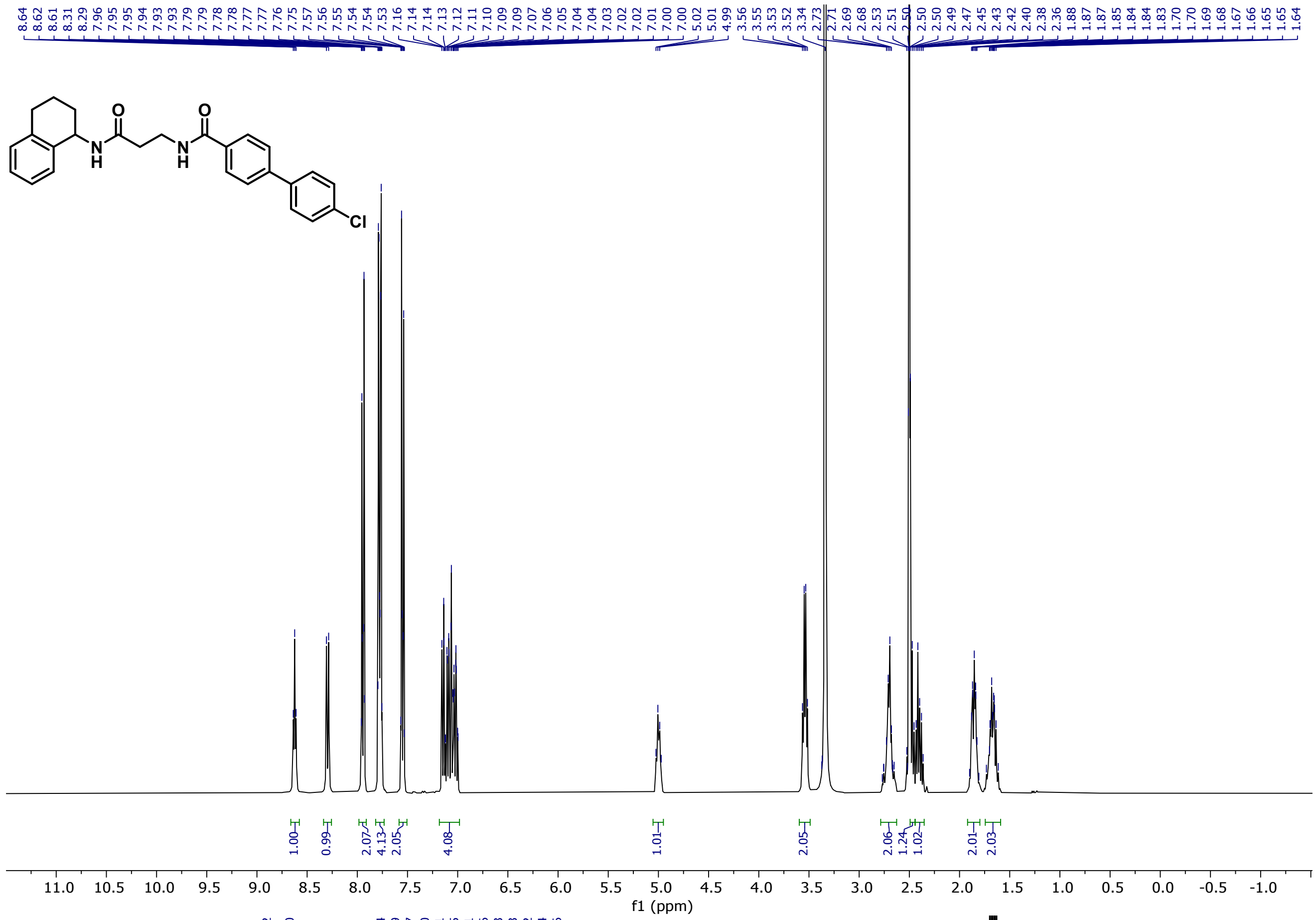

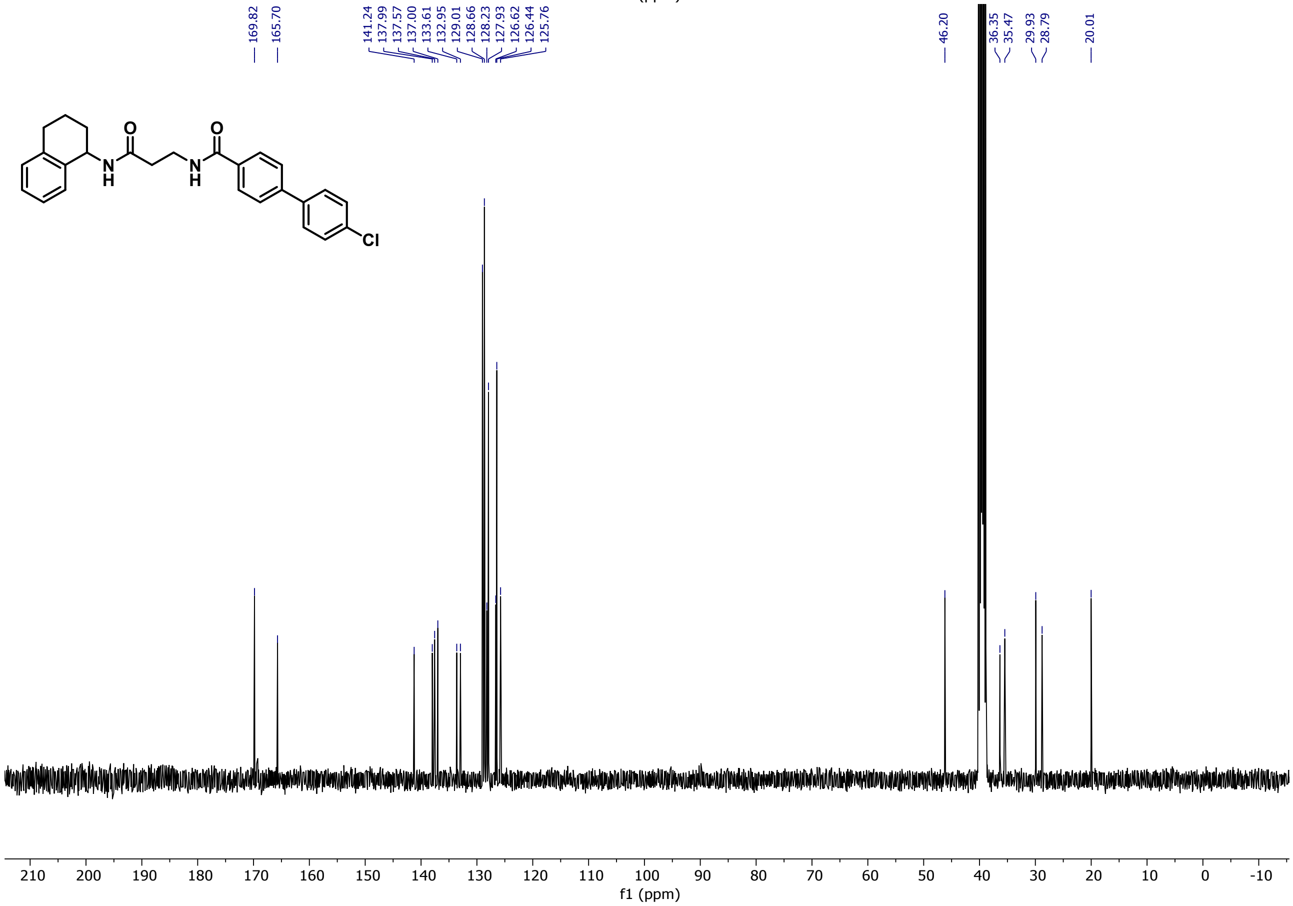

NMR spectra of 10b in DMSO-d_6.

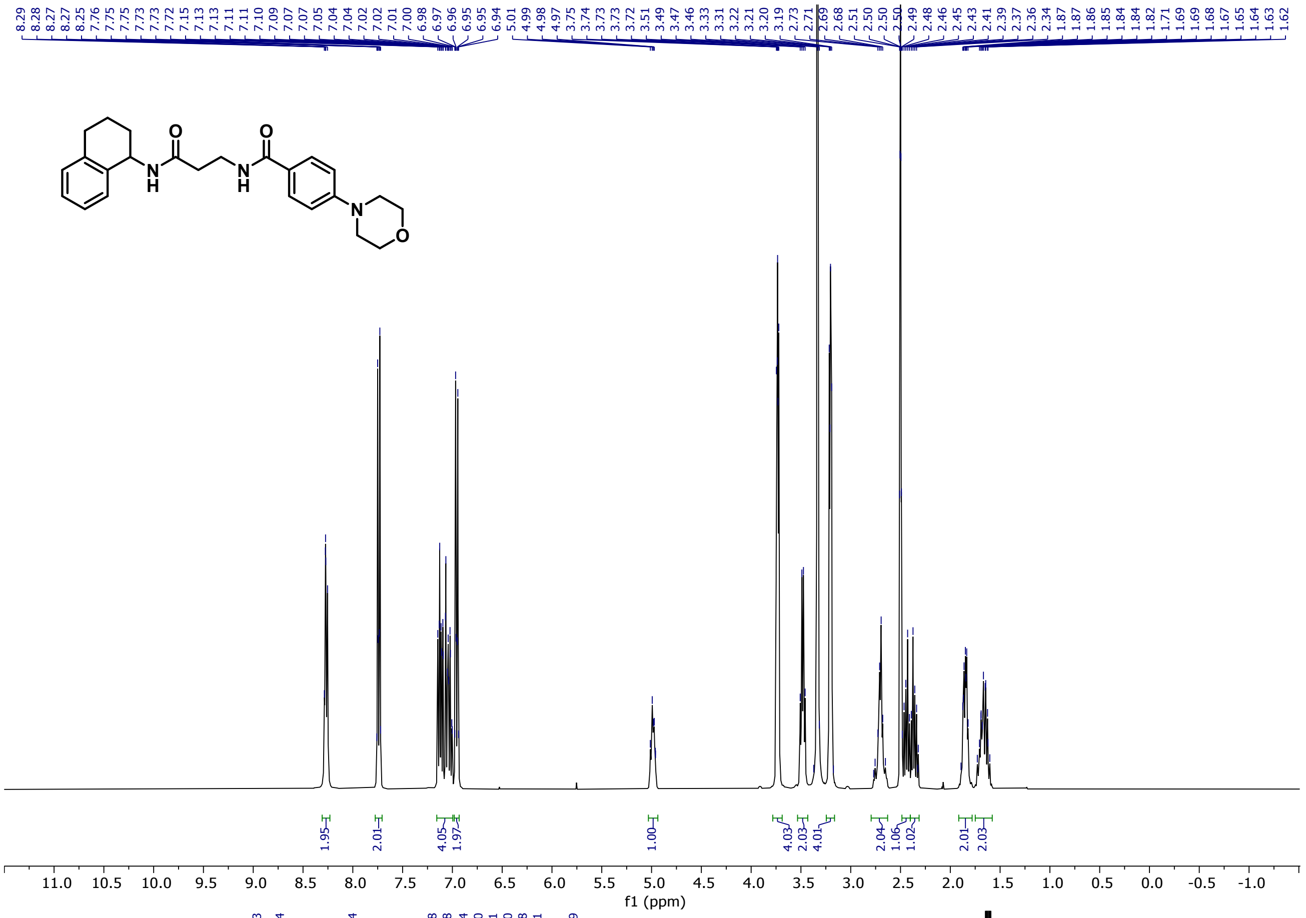

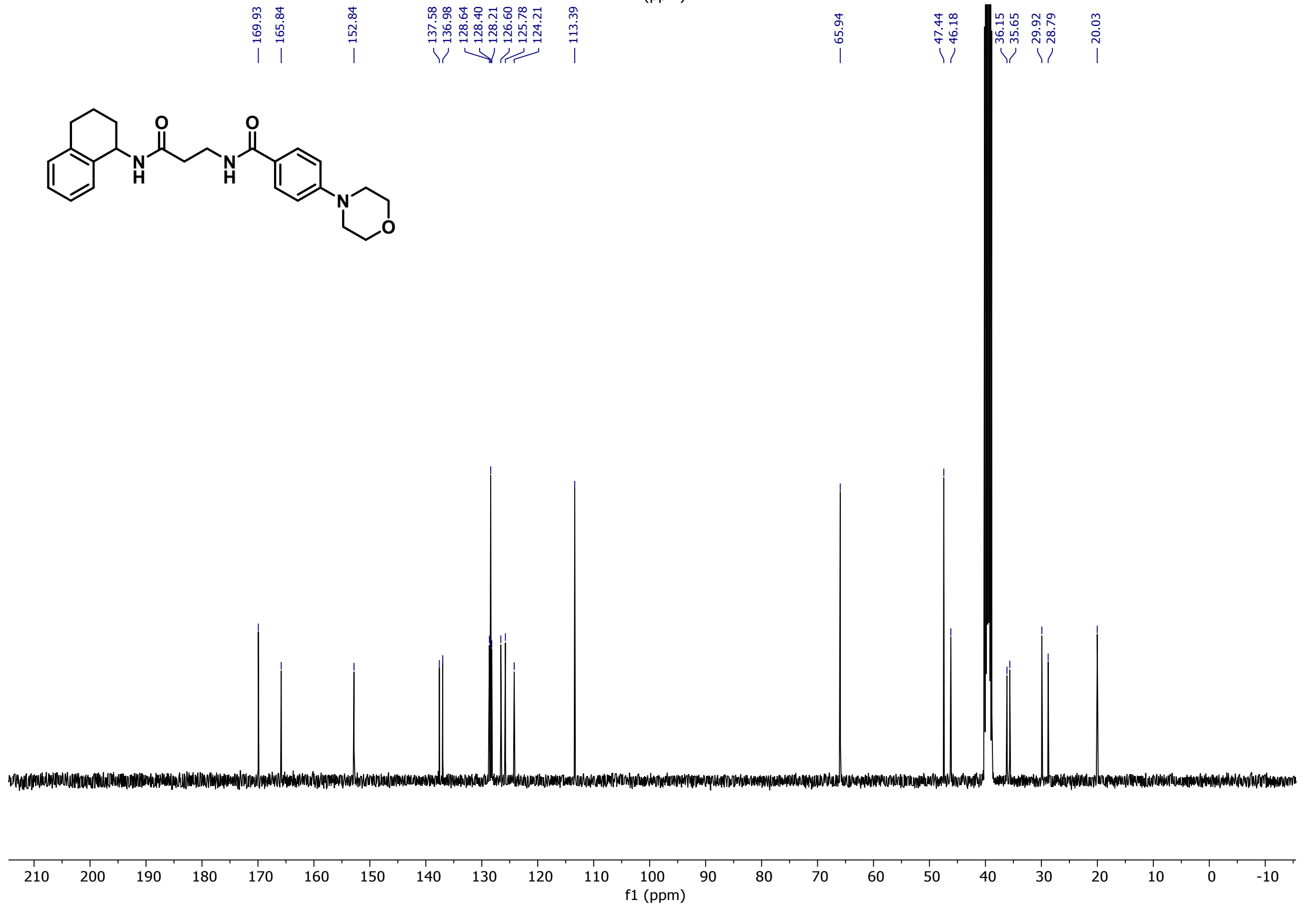

NMR spectra of 10c in DMSO-d_6.

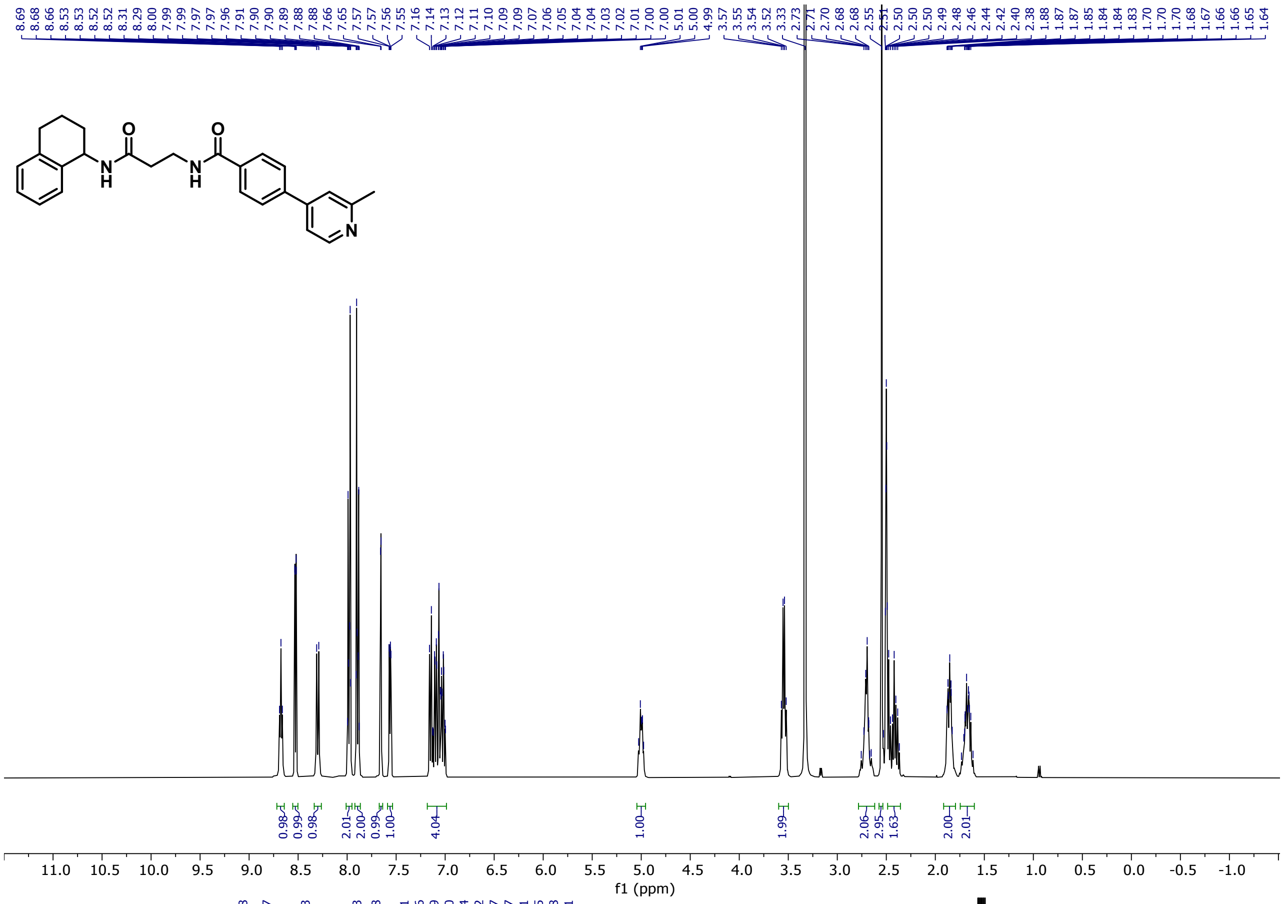

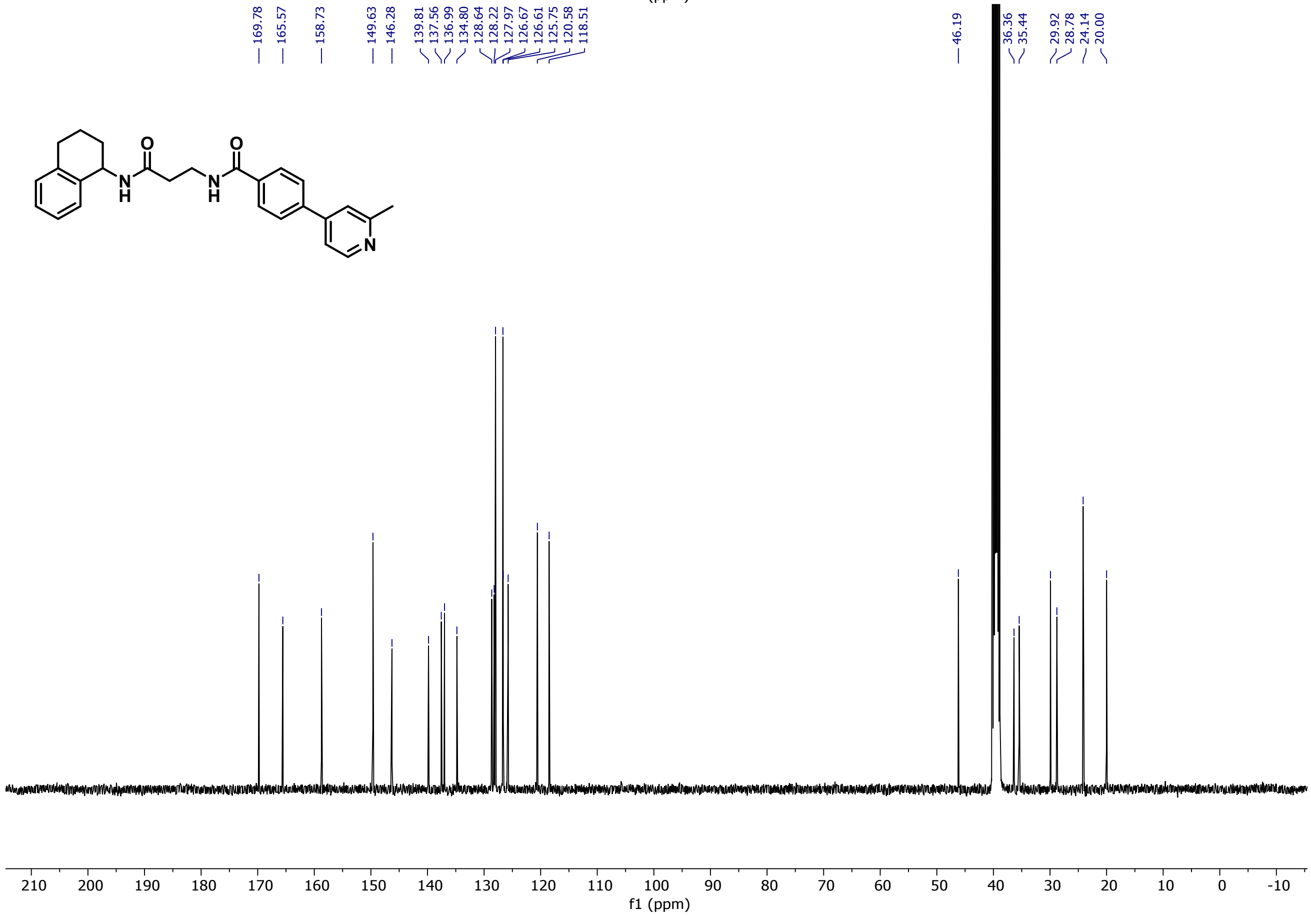

NMR spectra of 10d in DMSO-d_6.

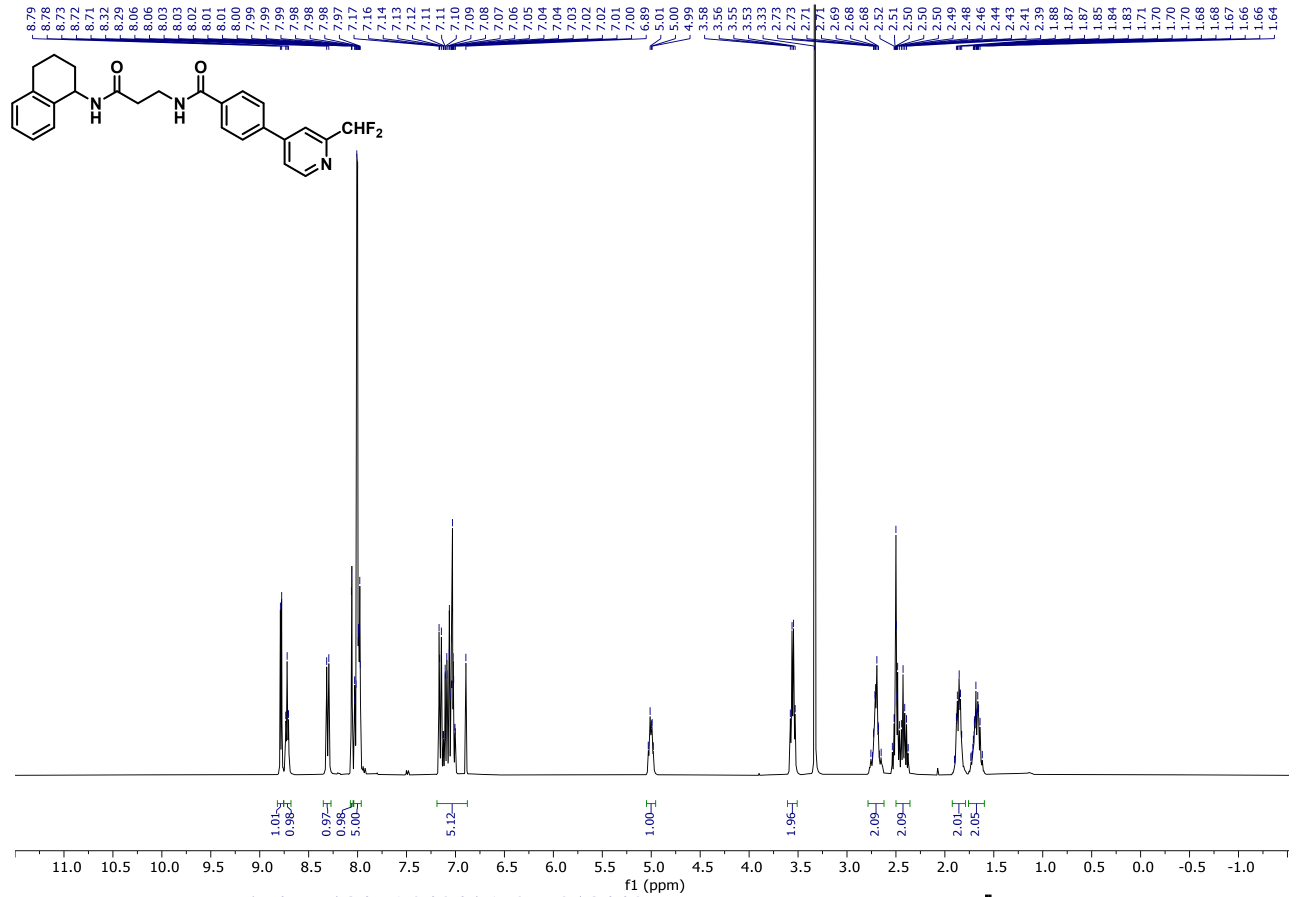

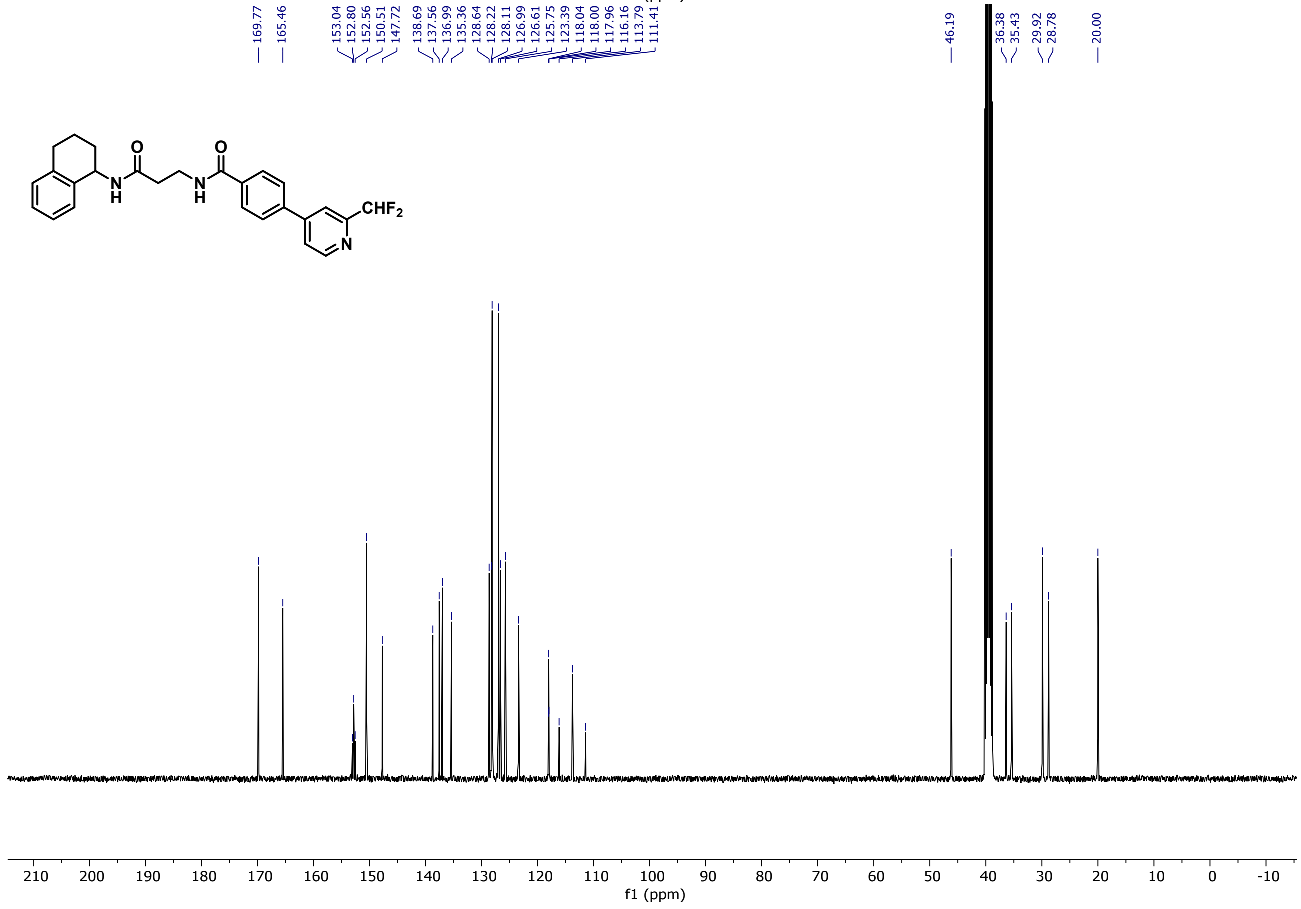

NMR spectra of 10e in DMSO-d_6.

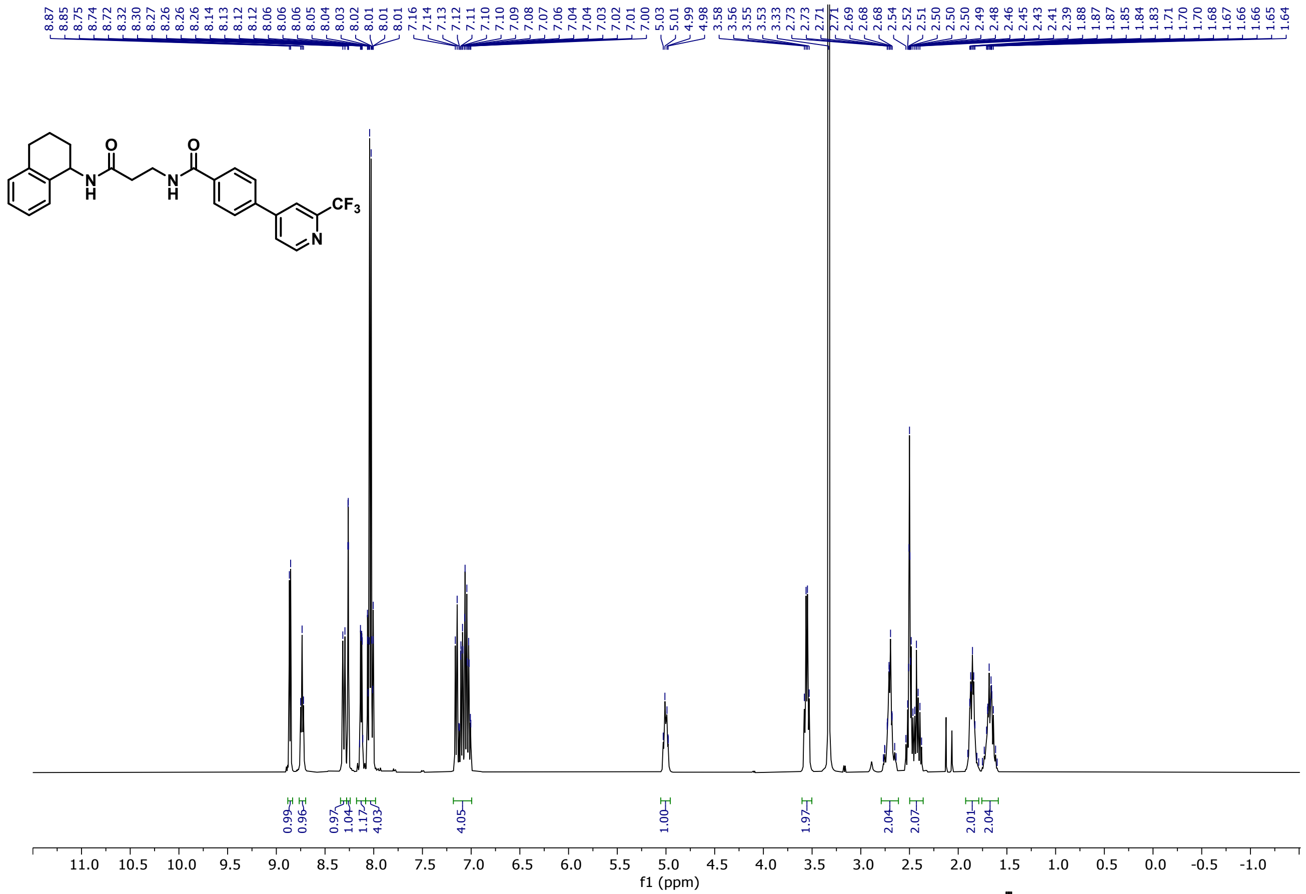

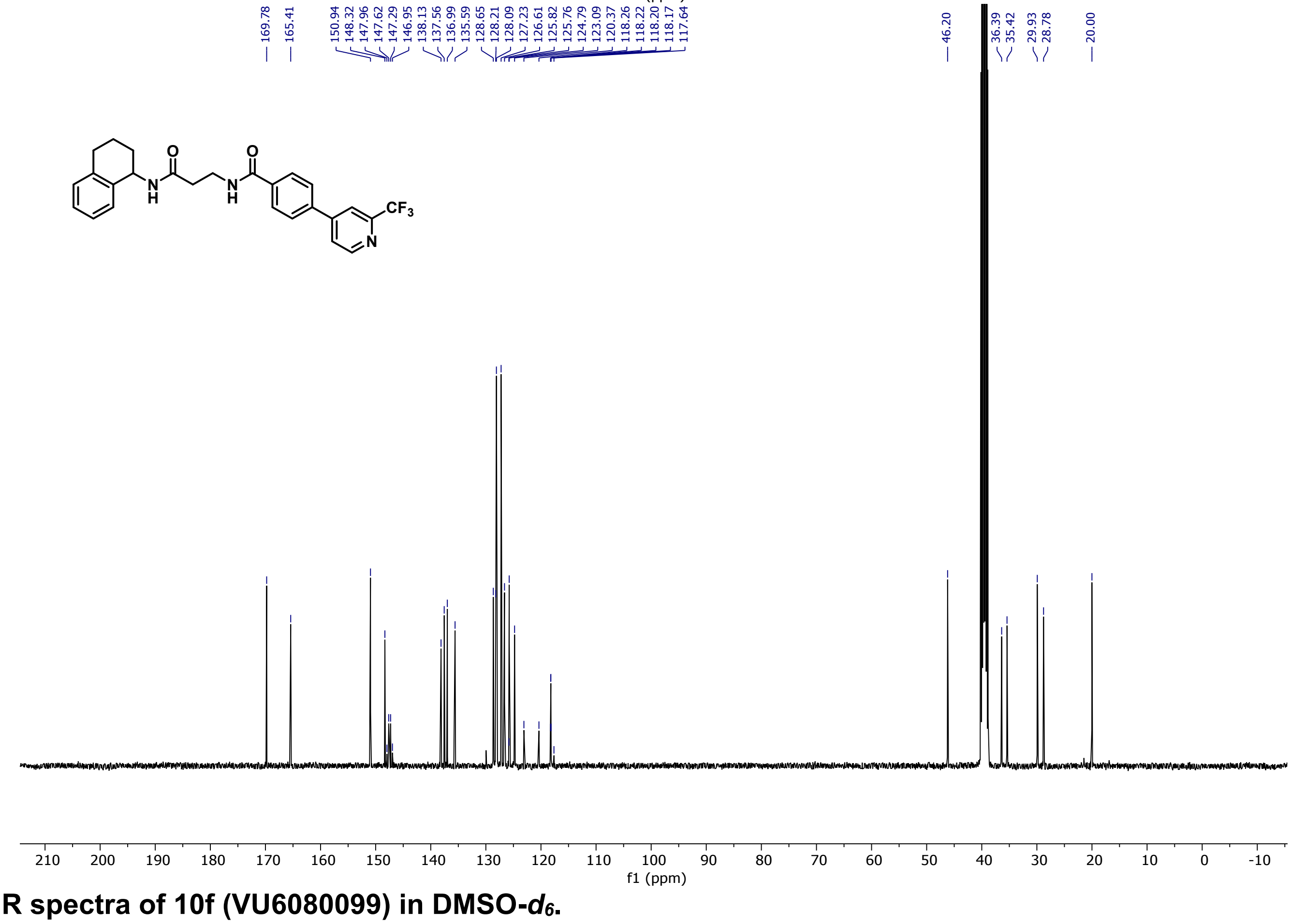

NMR spectra of 10f (VU6080099) in DMSO-d_6.

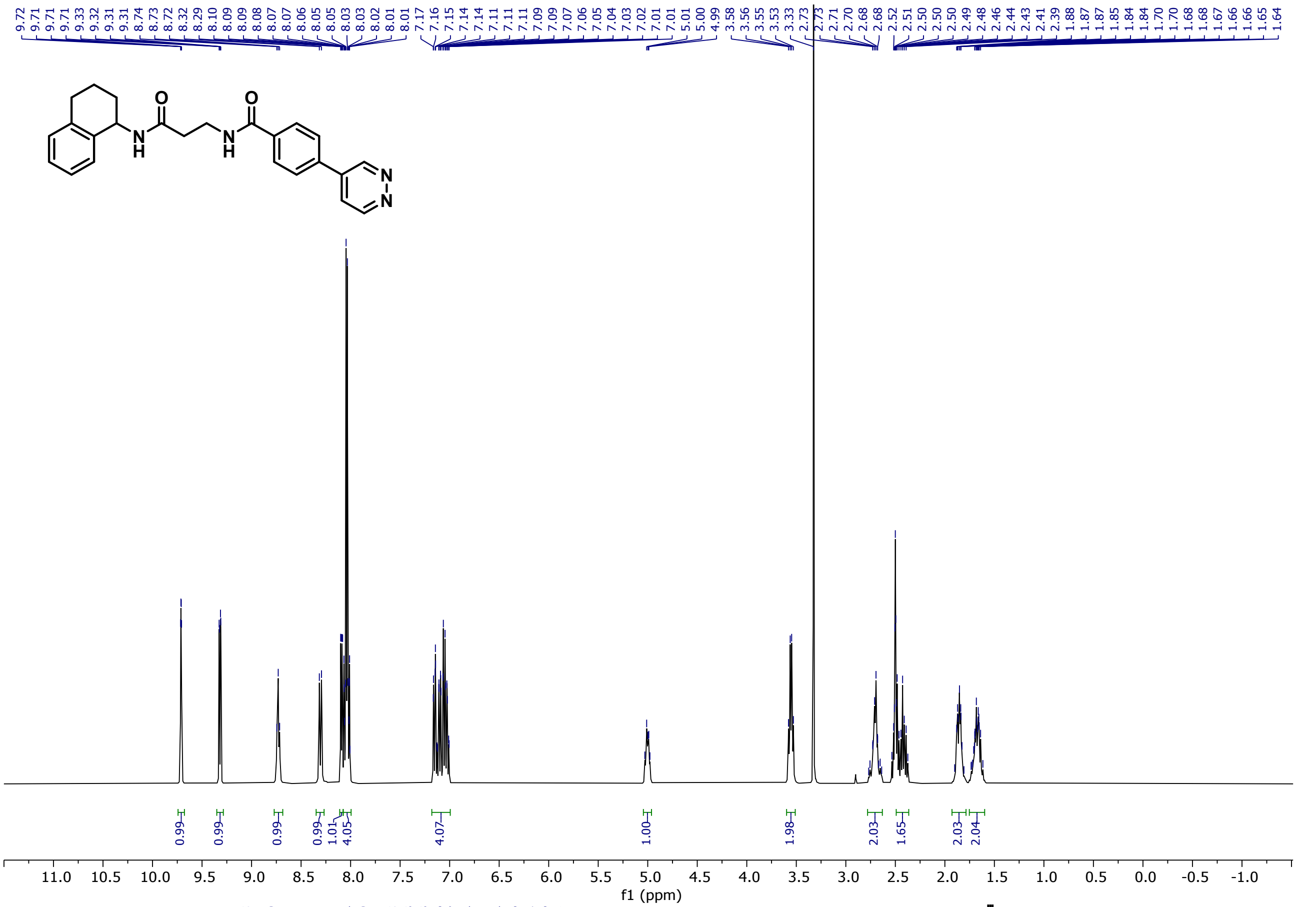

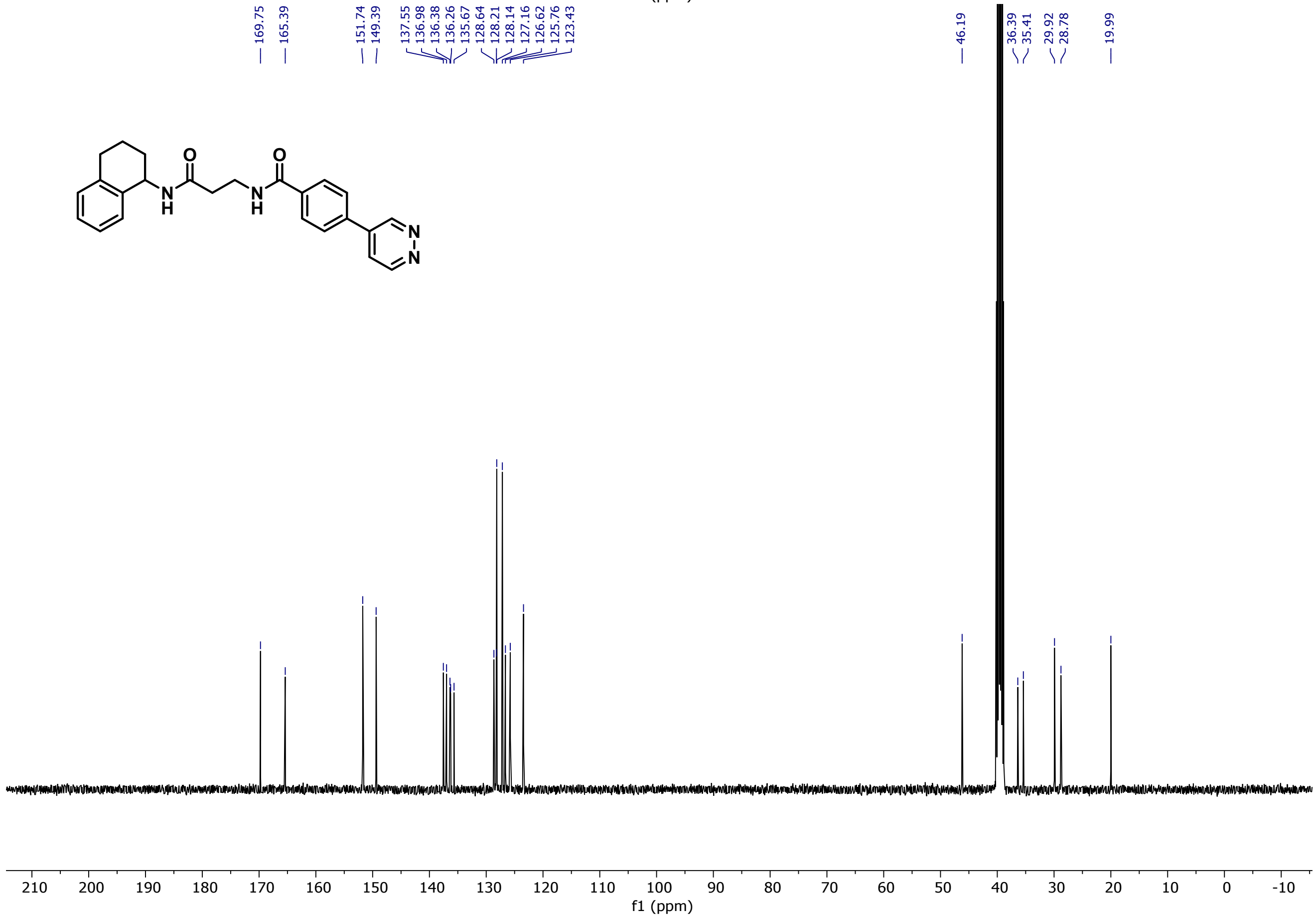

NMR spectra of 10g in DMSO-d_6.

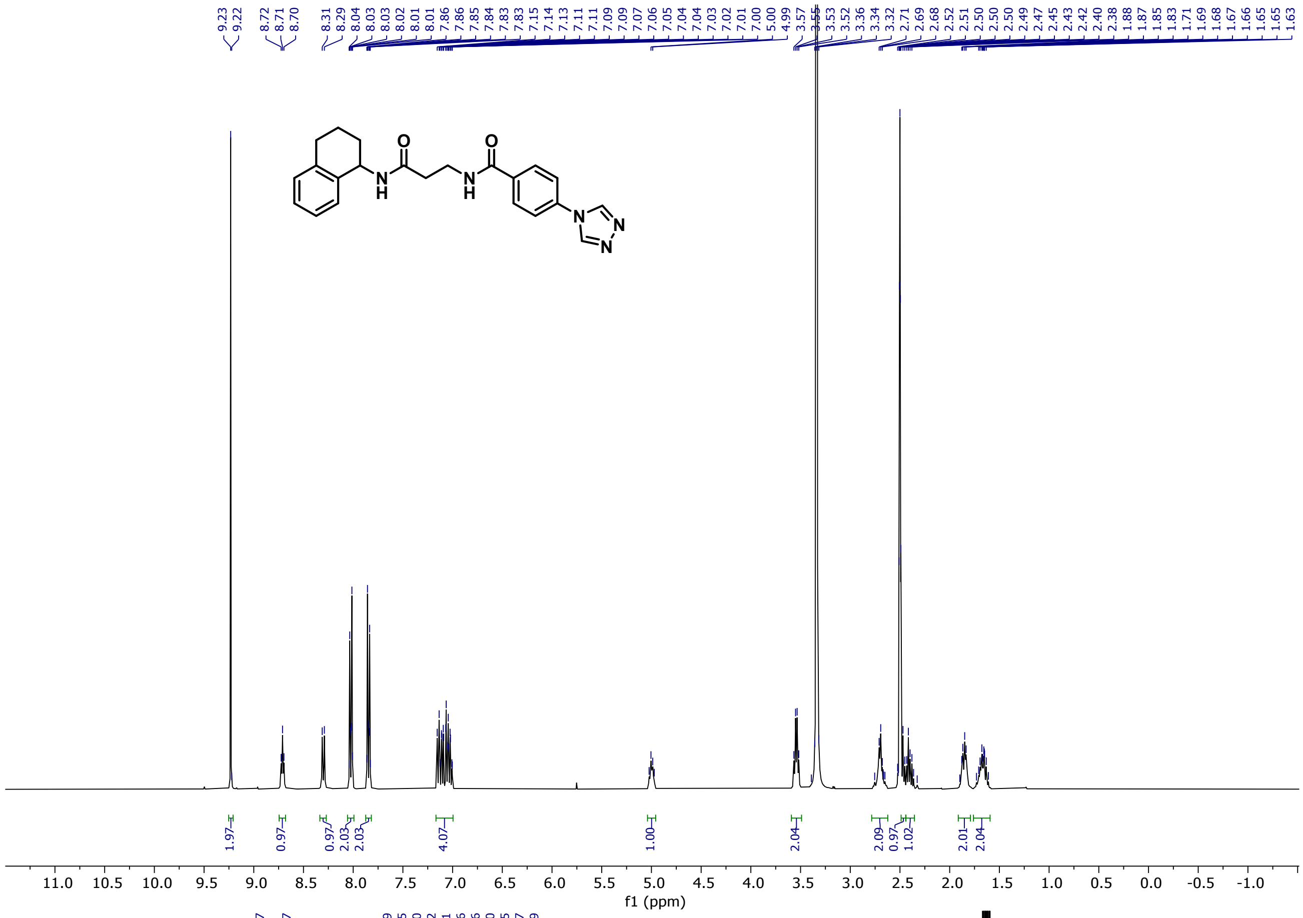

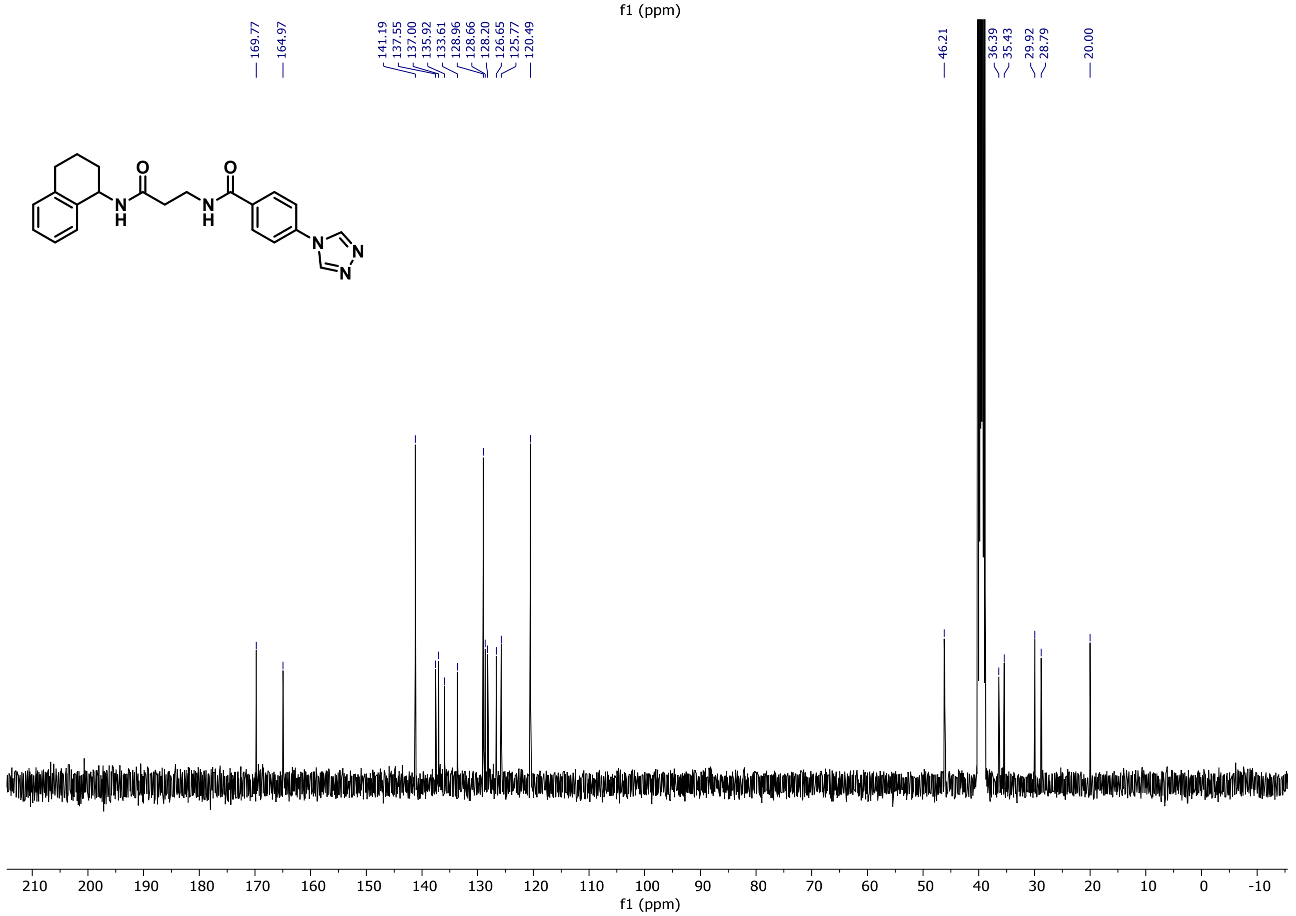

NMR spectra of 10h in DMSO-d_6.

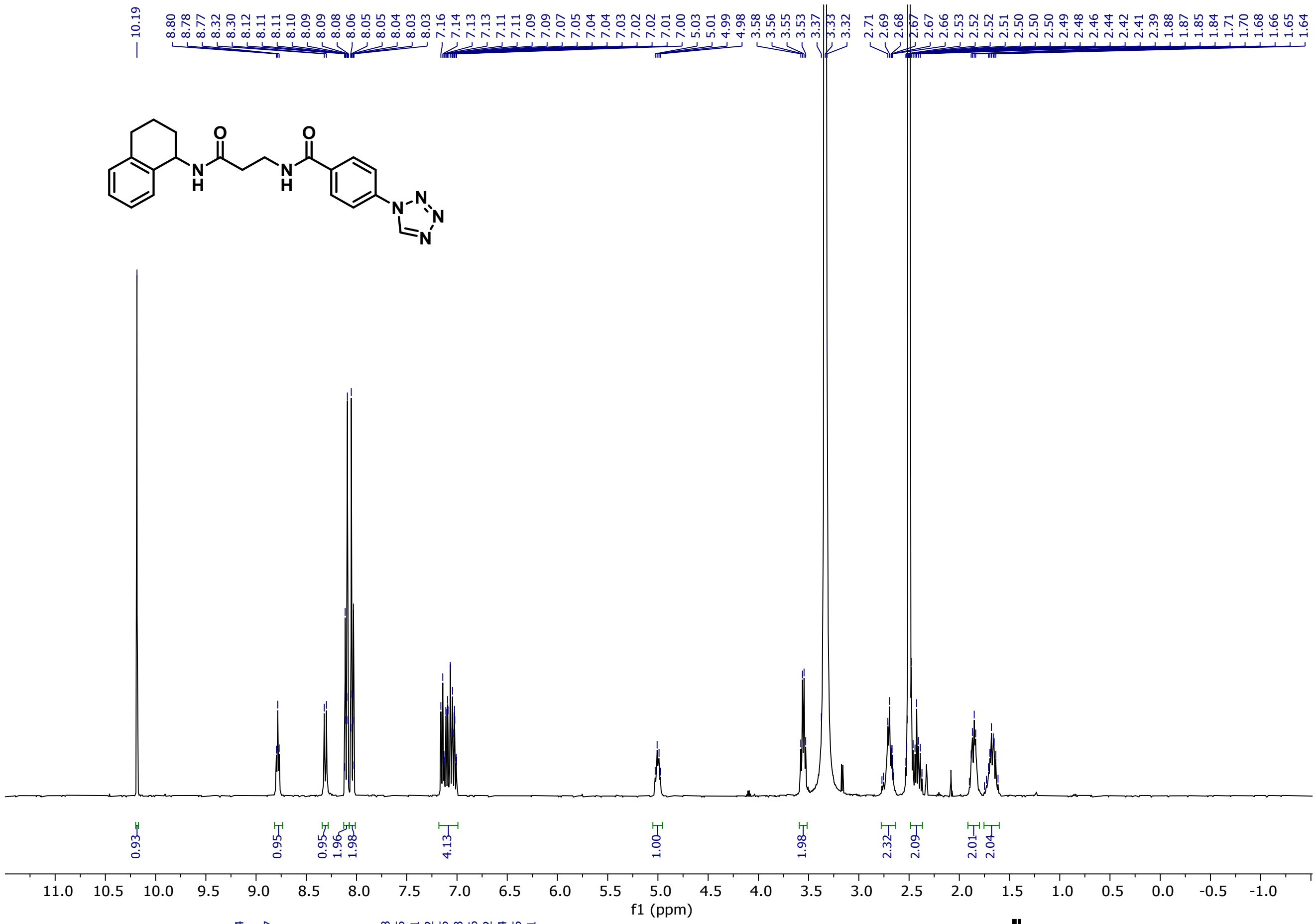

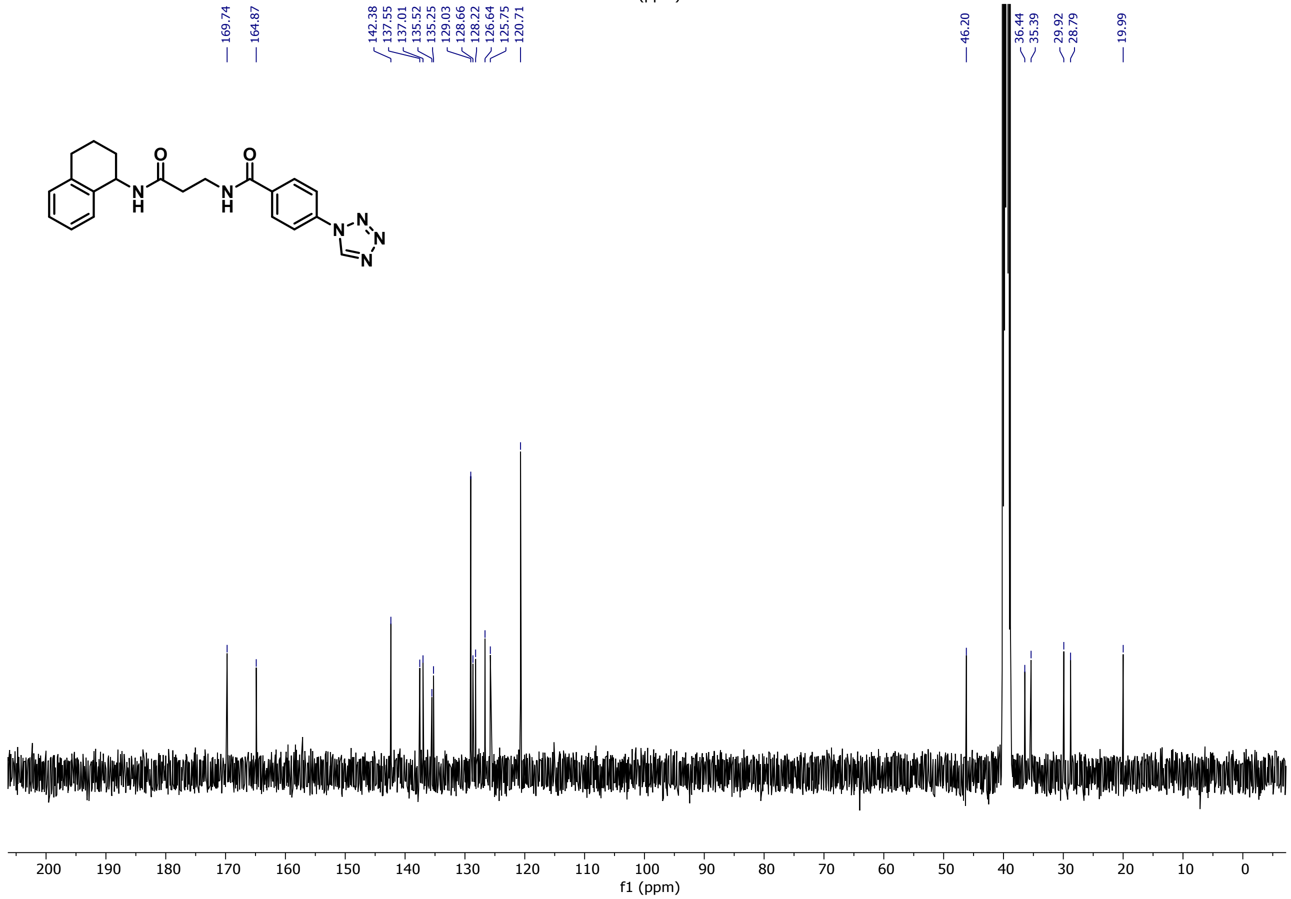

NMR spectra of 10i in DMSO-d_6.

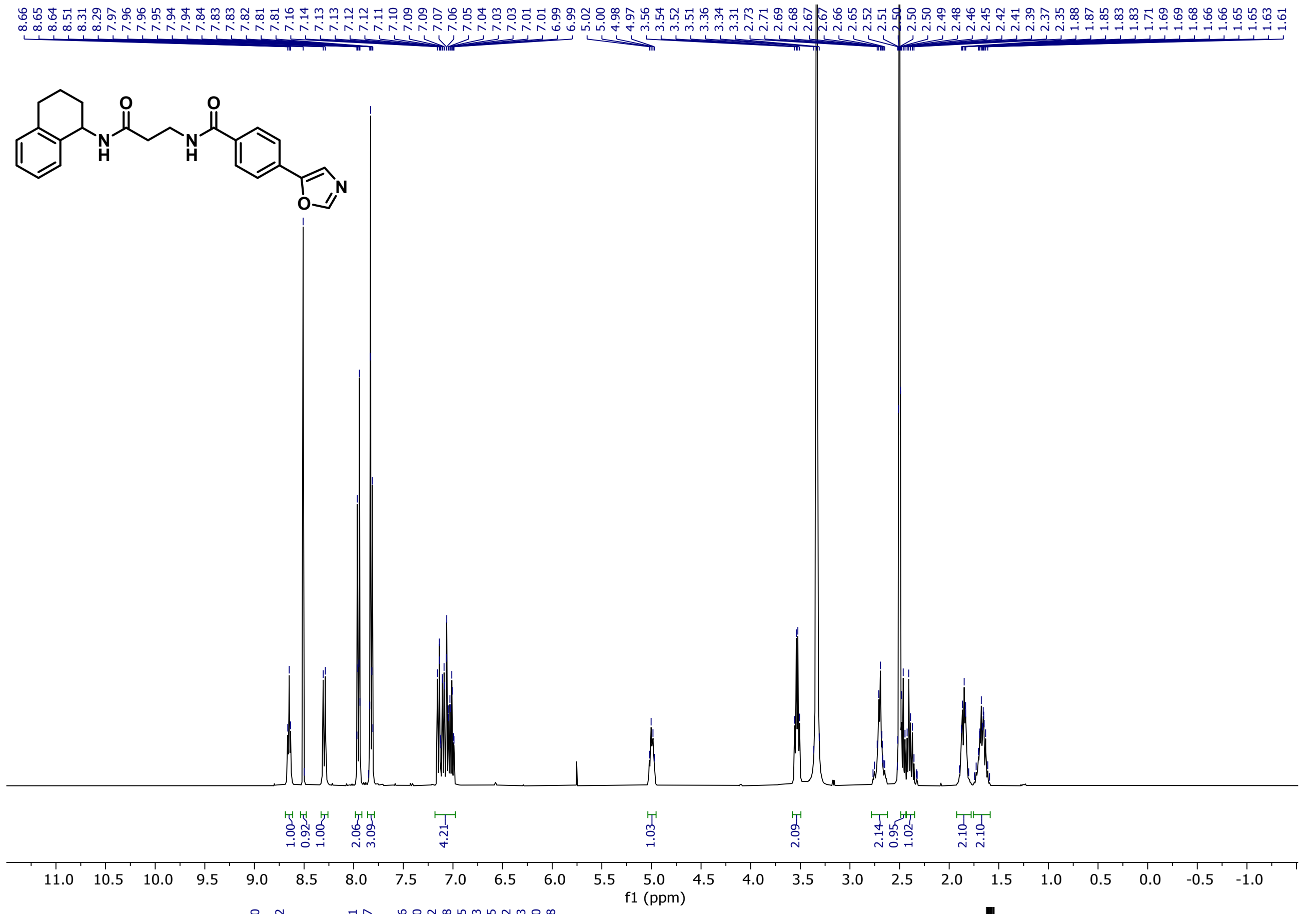

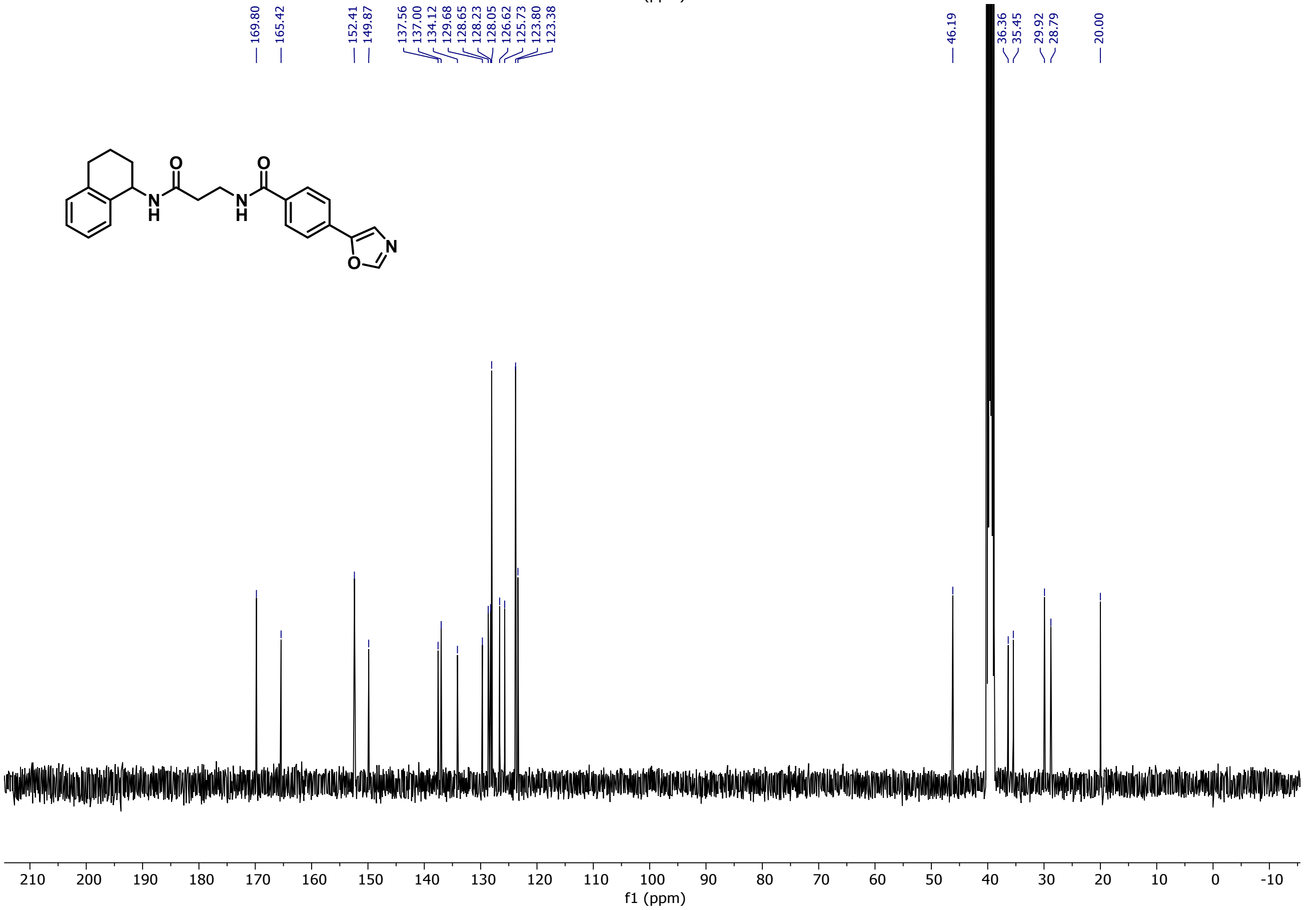

NMR spectra of 10j in DMSO-*d*$_6$.

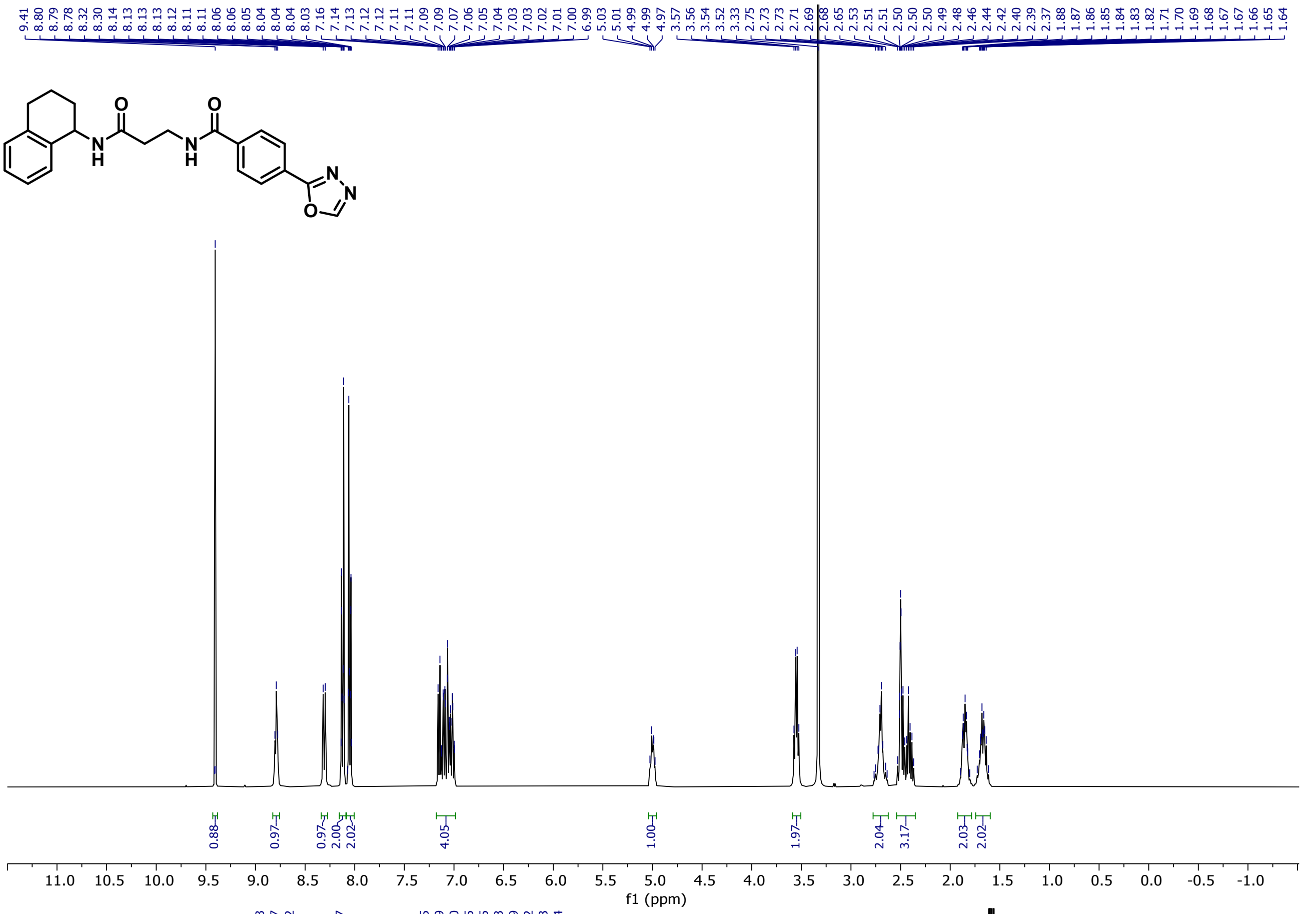

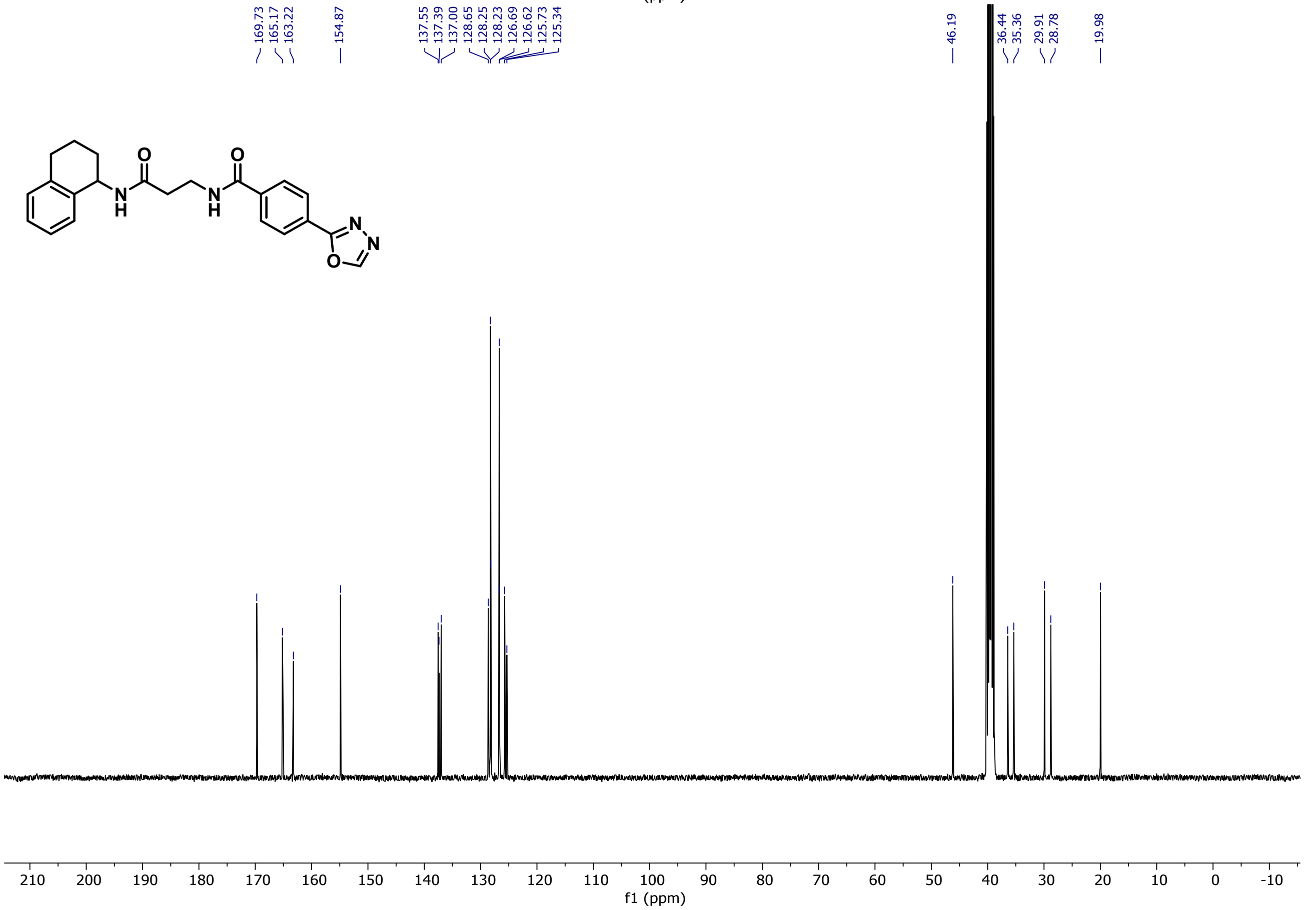

NMR spectra of 10k in DMSO-d_6.

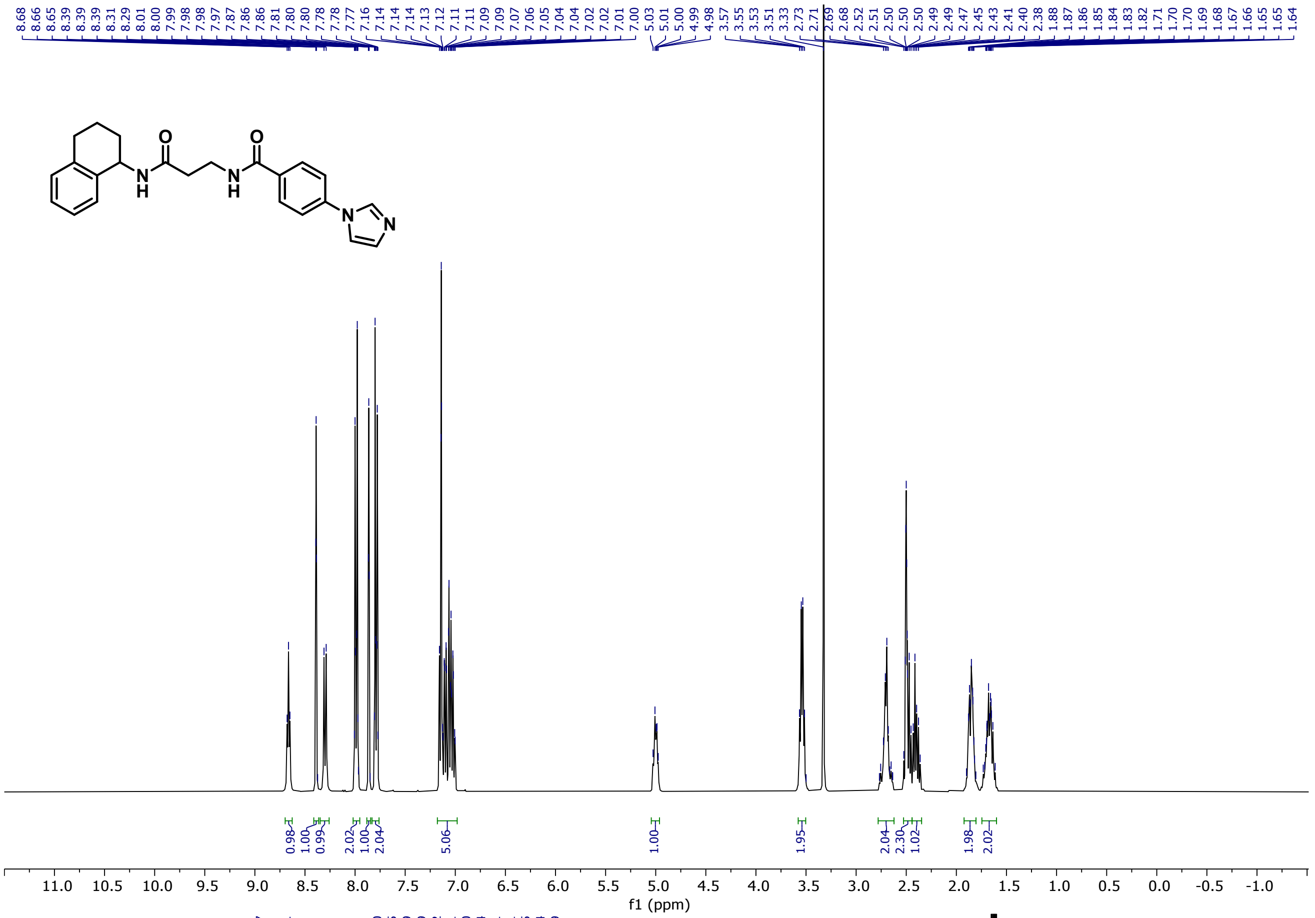

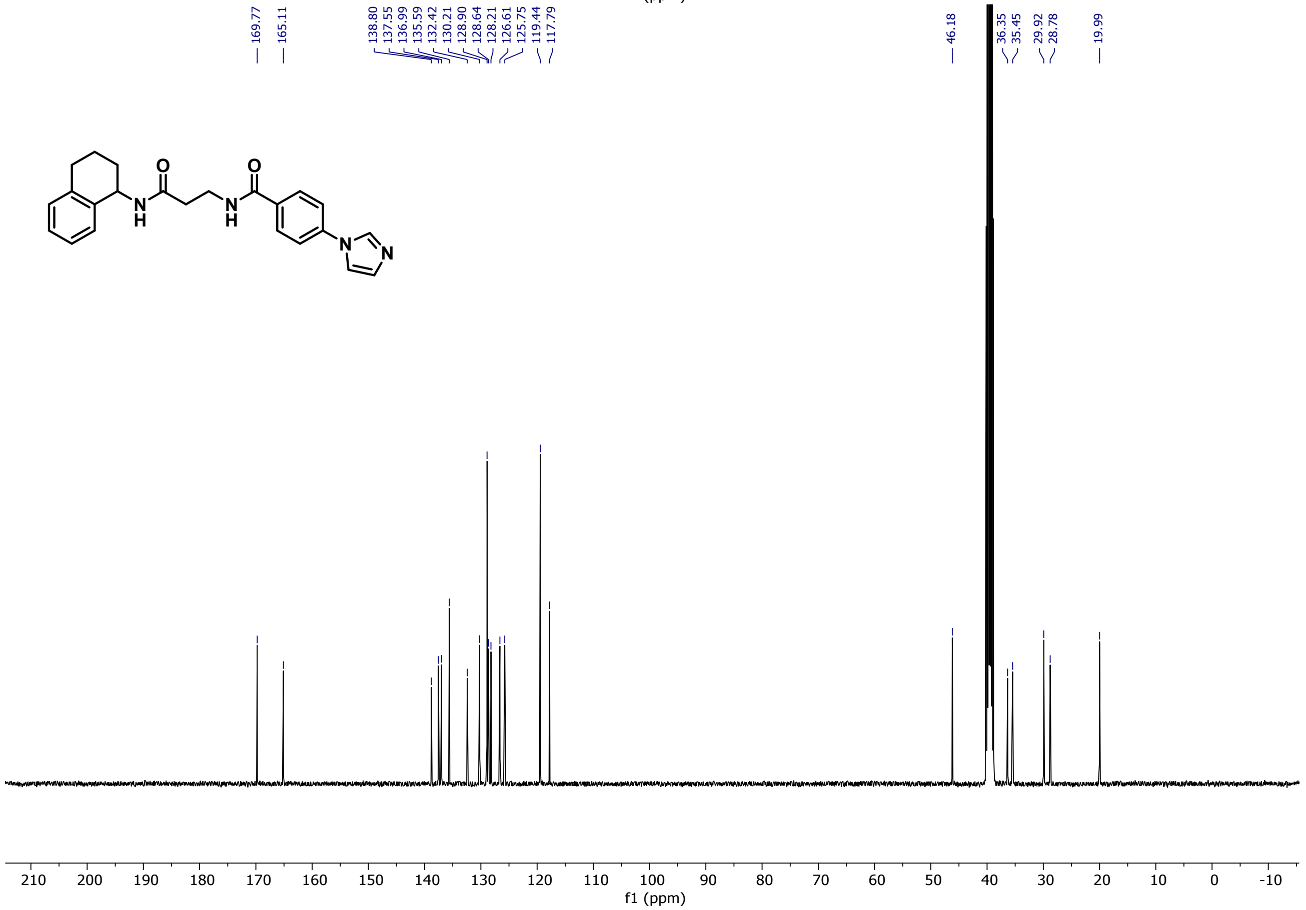

NMR spectra of 10l in DMSO-d_6.

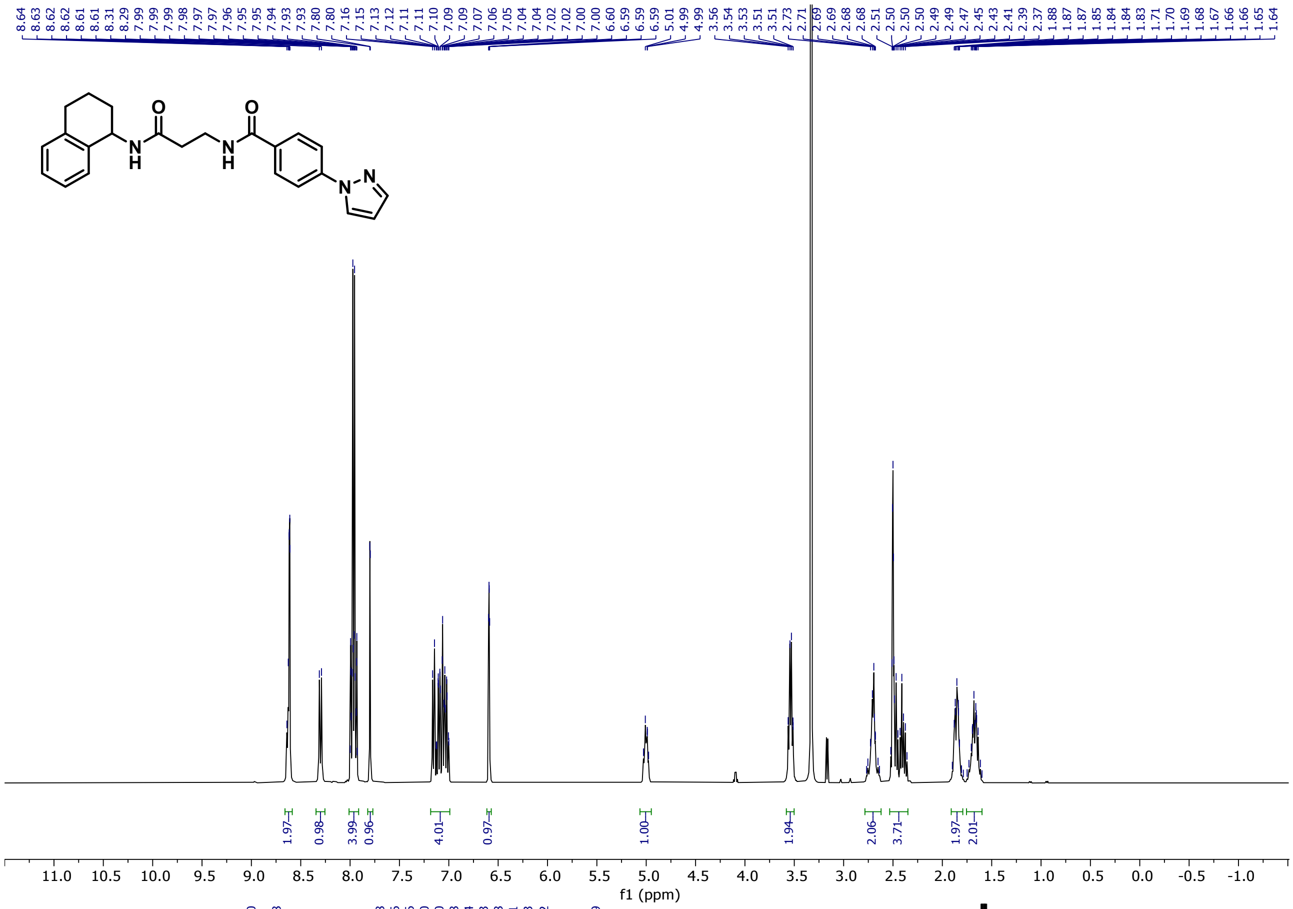

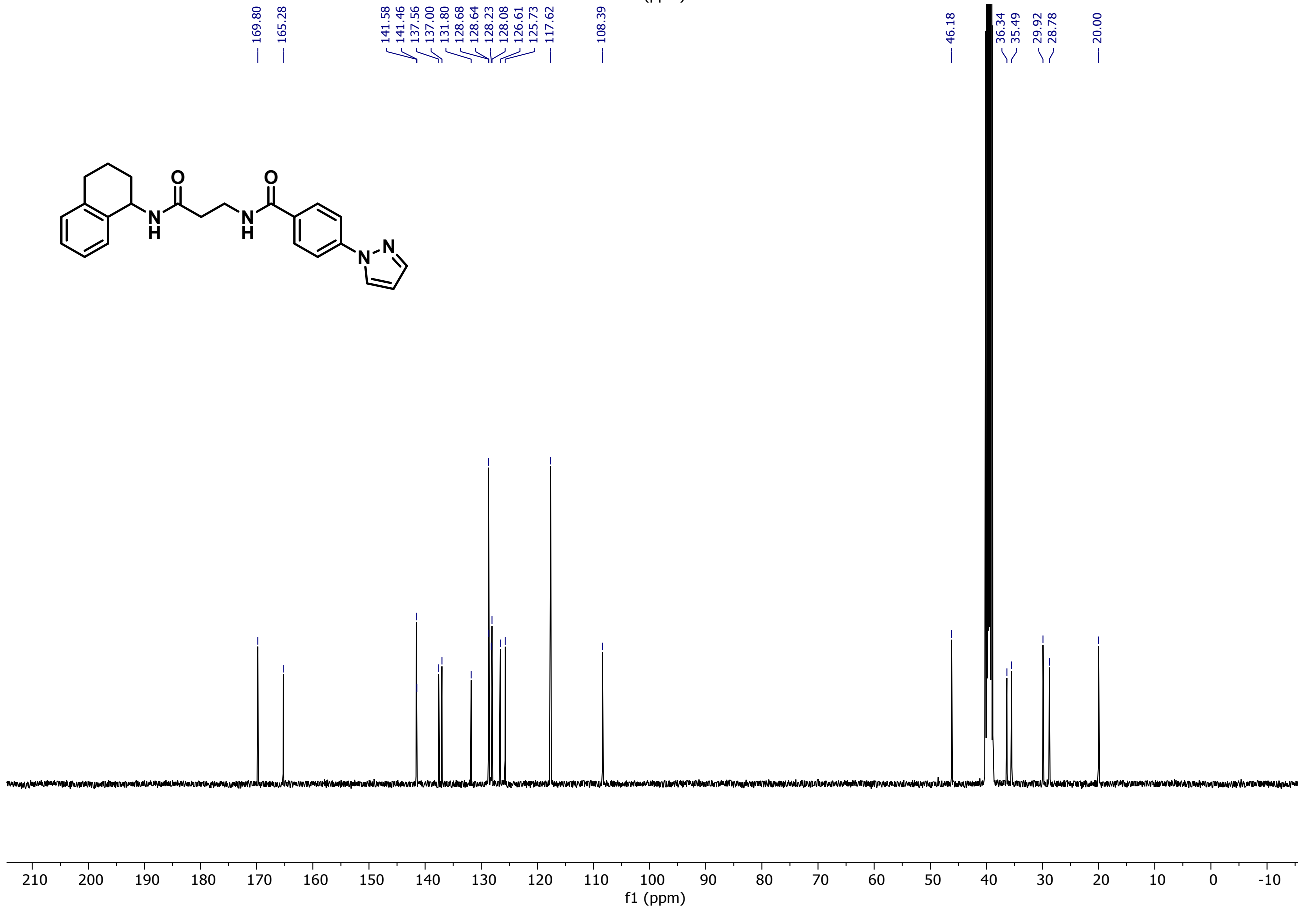

NMR spectra of 10m in DMSO-d_6.

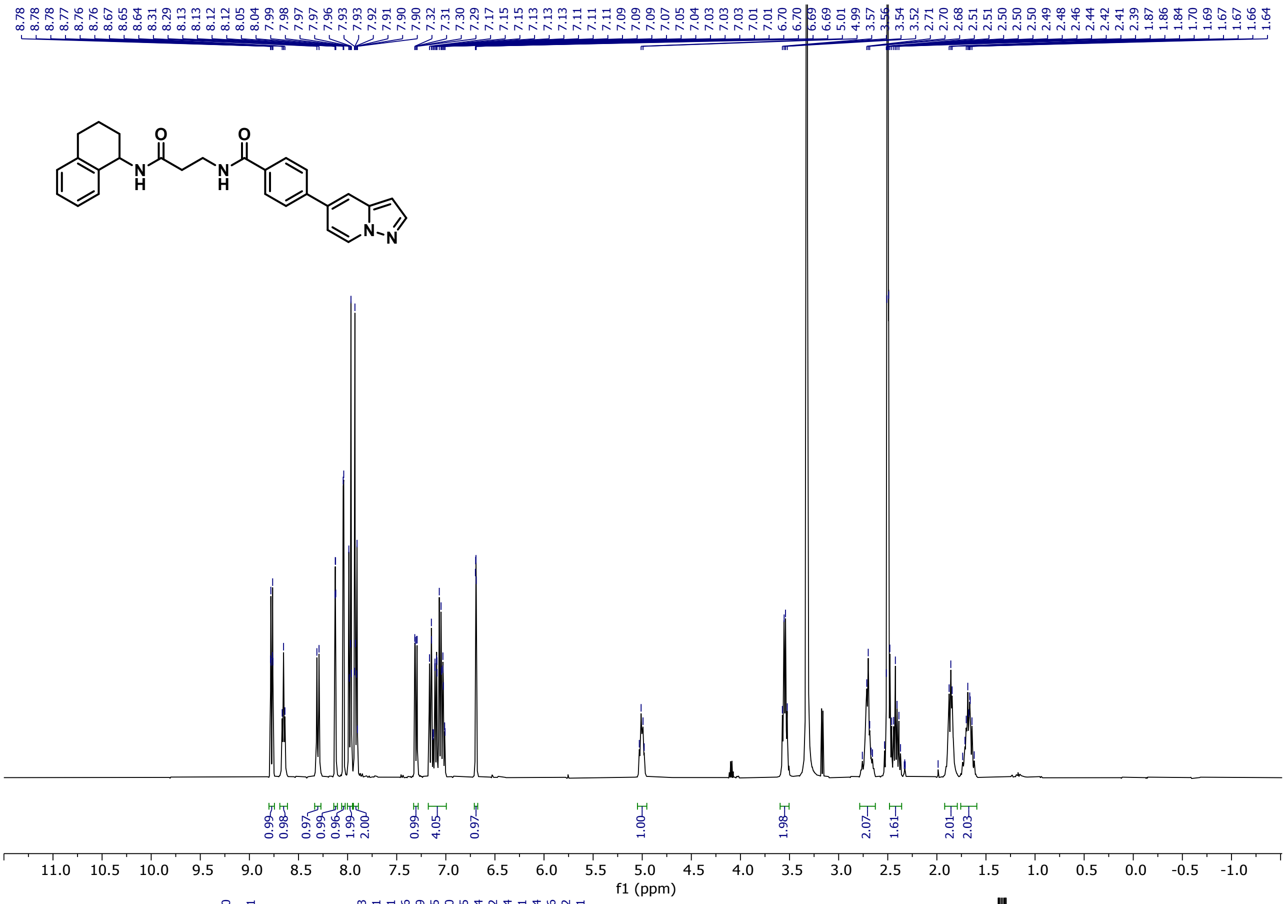

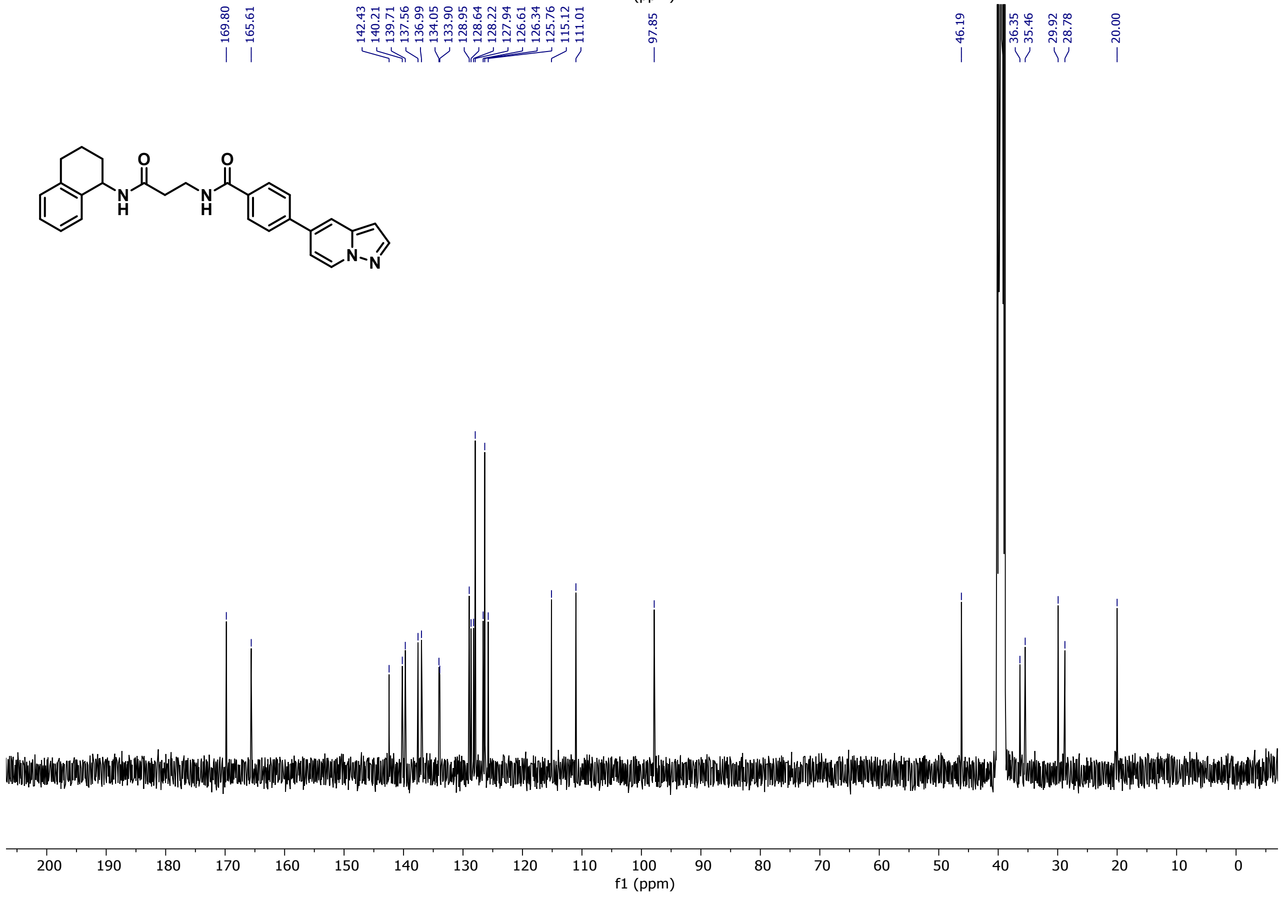

NMR spectra of 10n in DMSO-d_6.

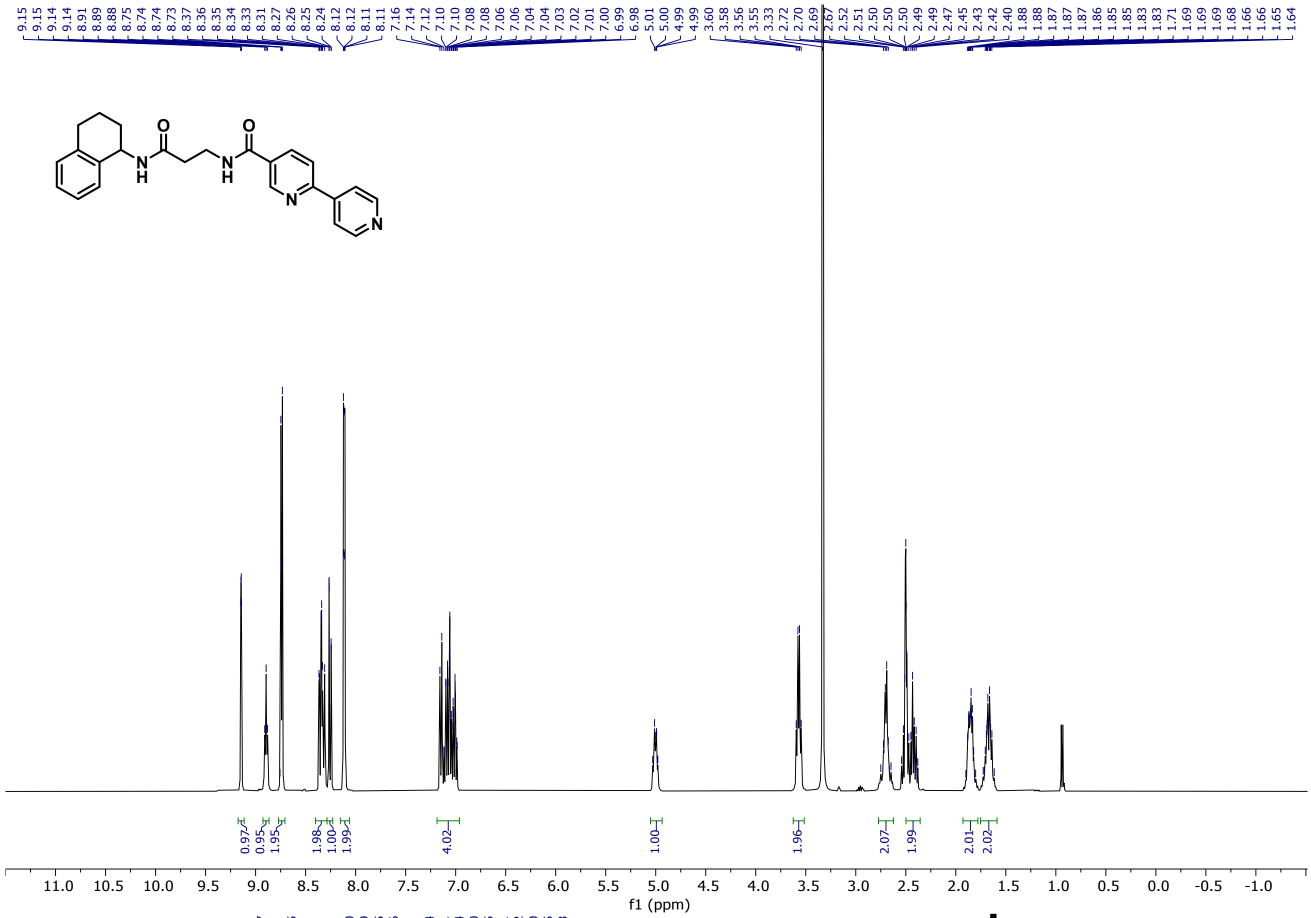

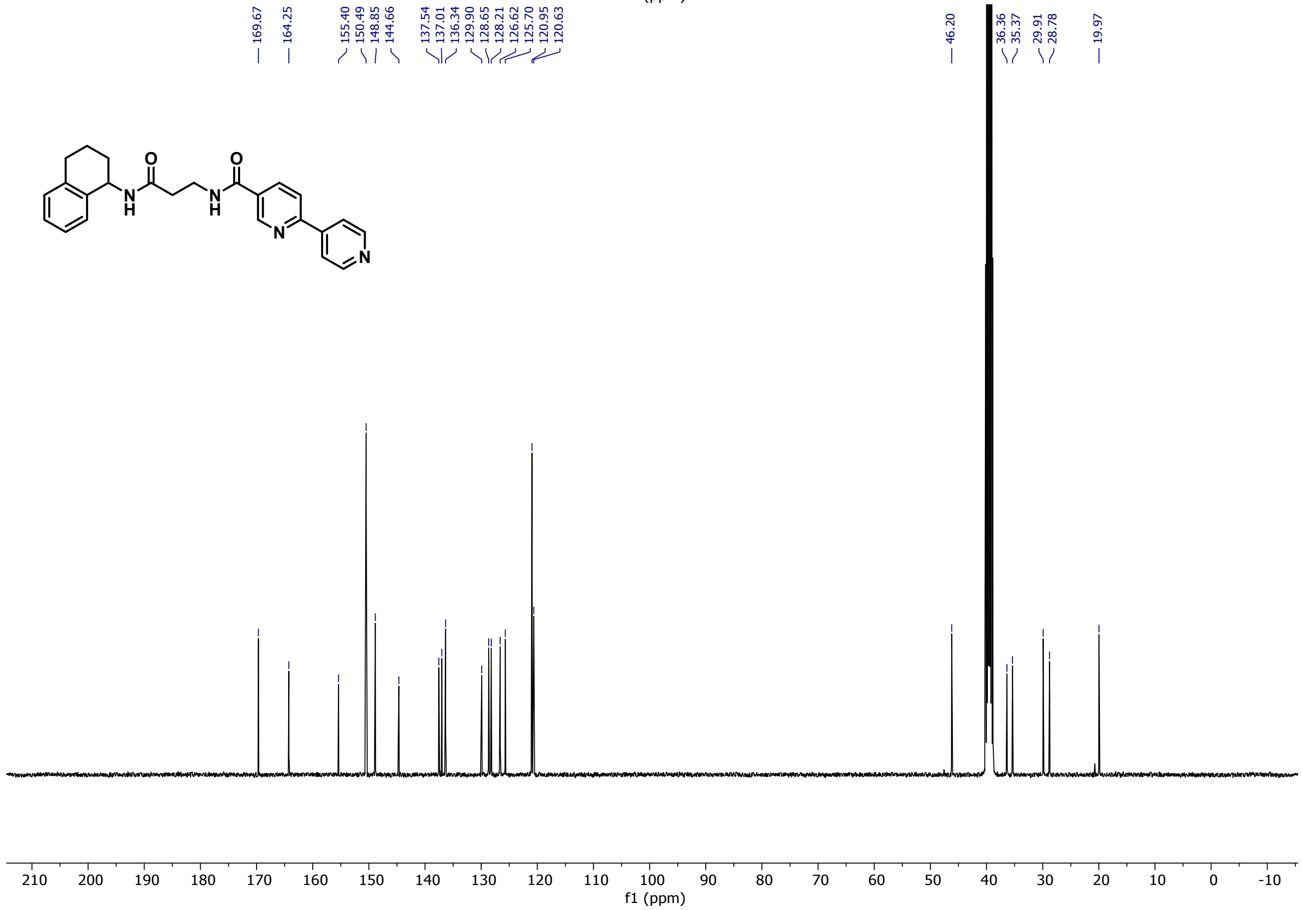

NMR spectra of 10o in DMSO-*d*₆.

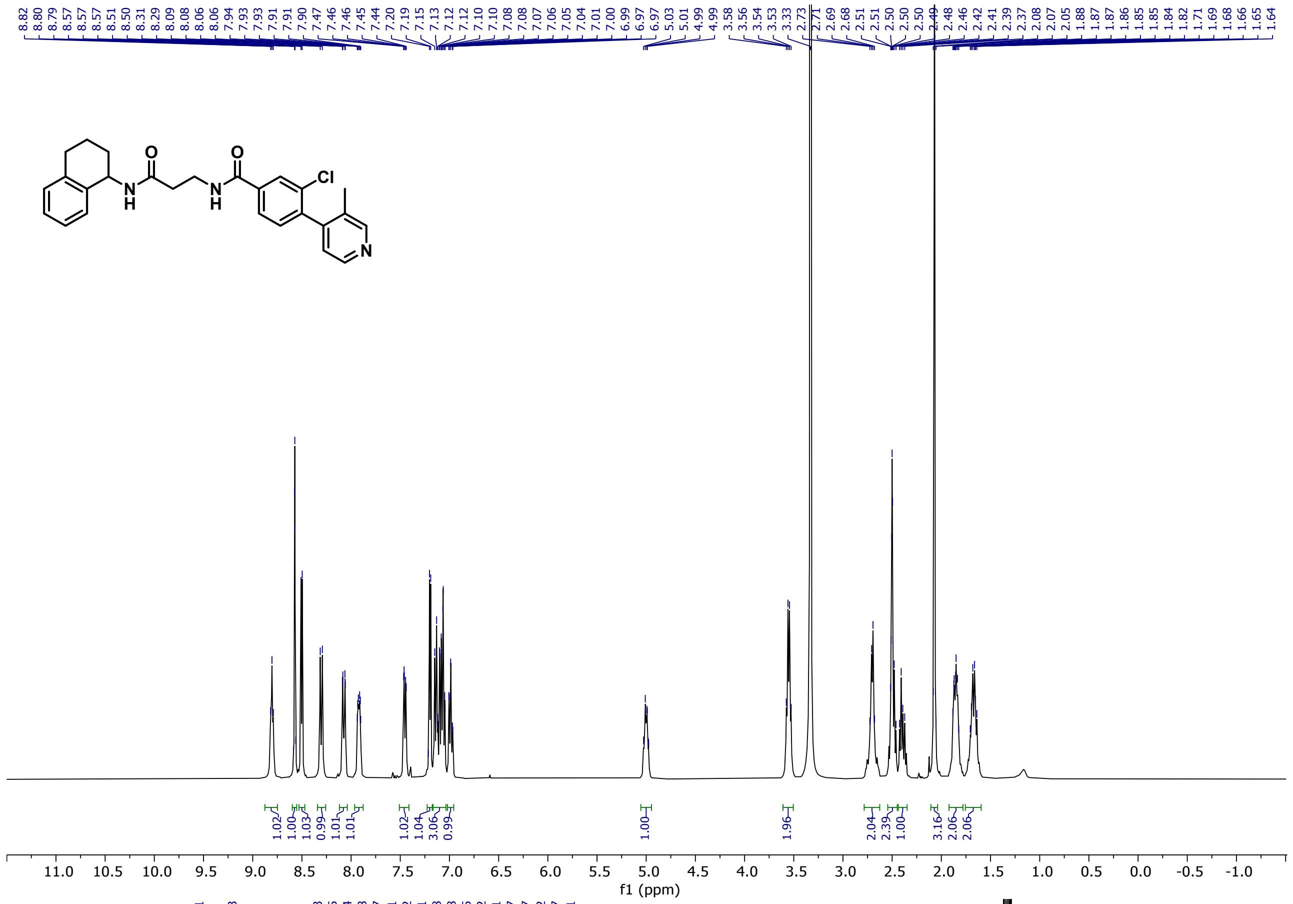

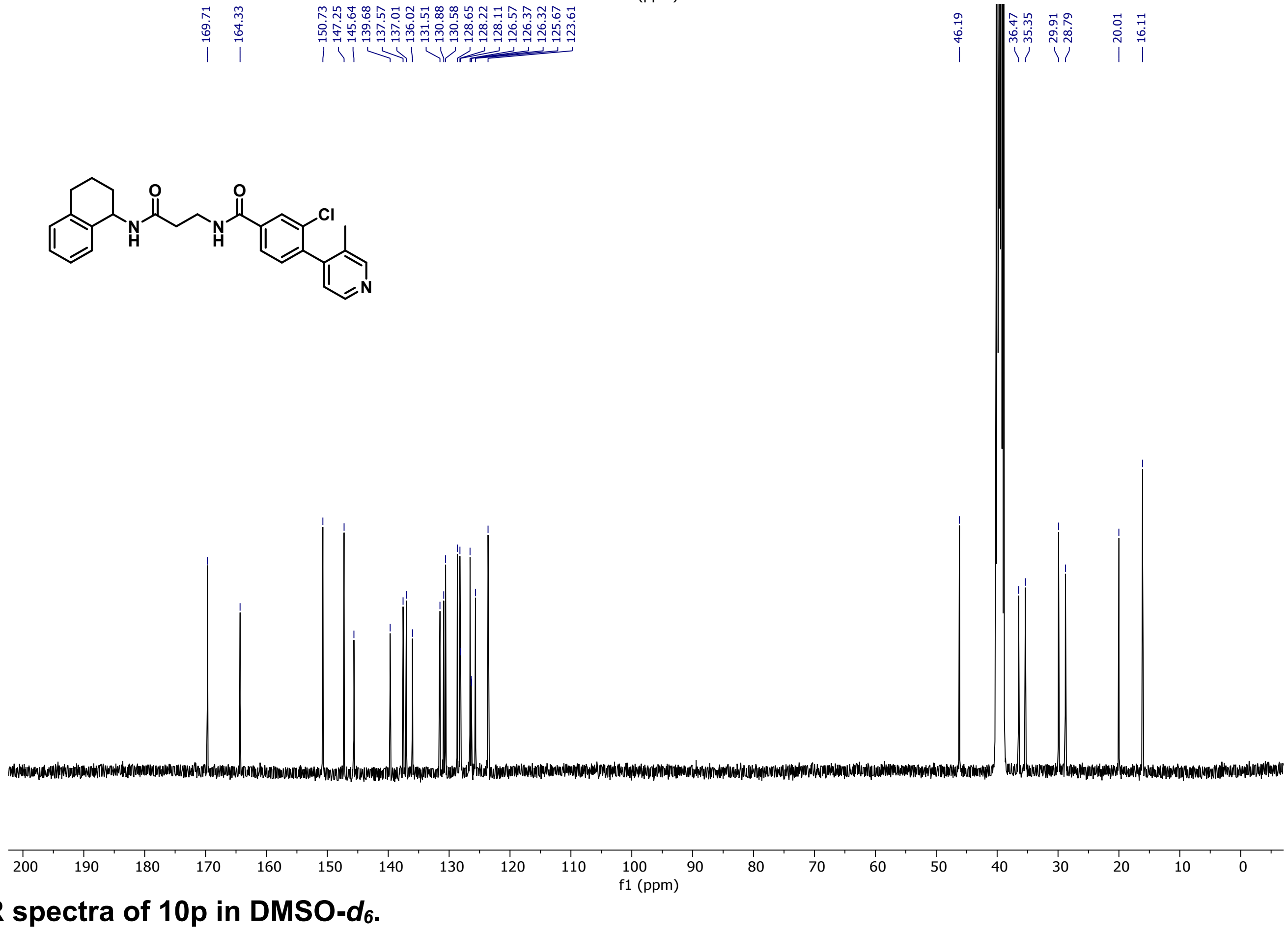

NMR spectra of 10p in DMSO-d_6.

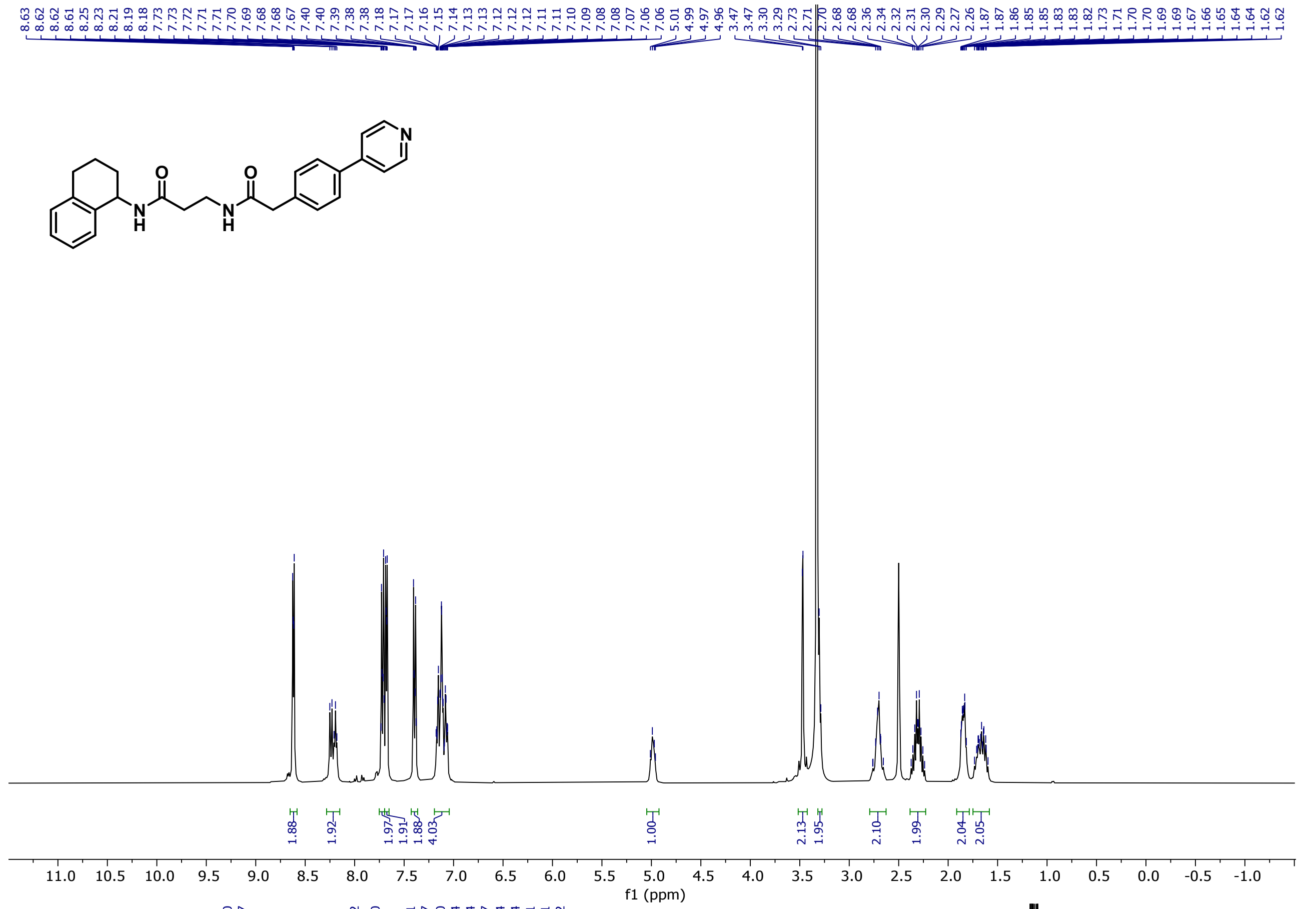

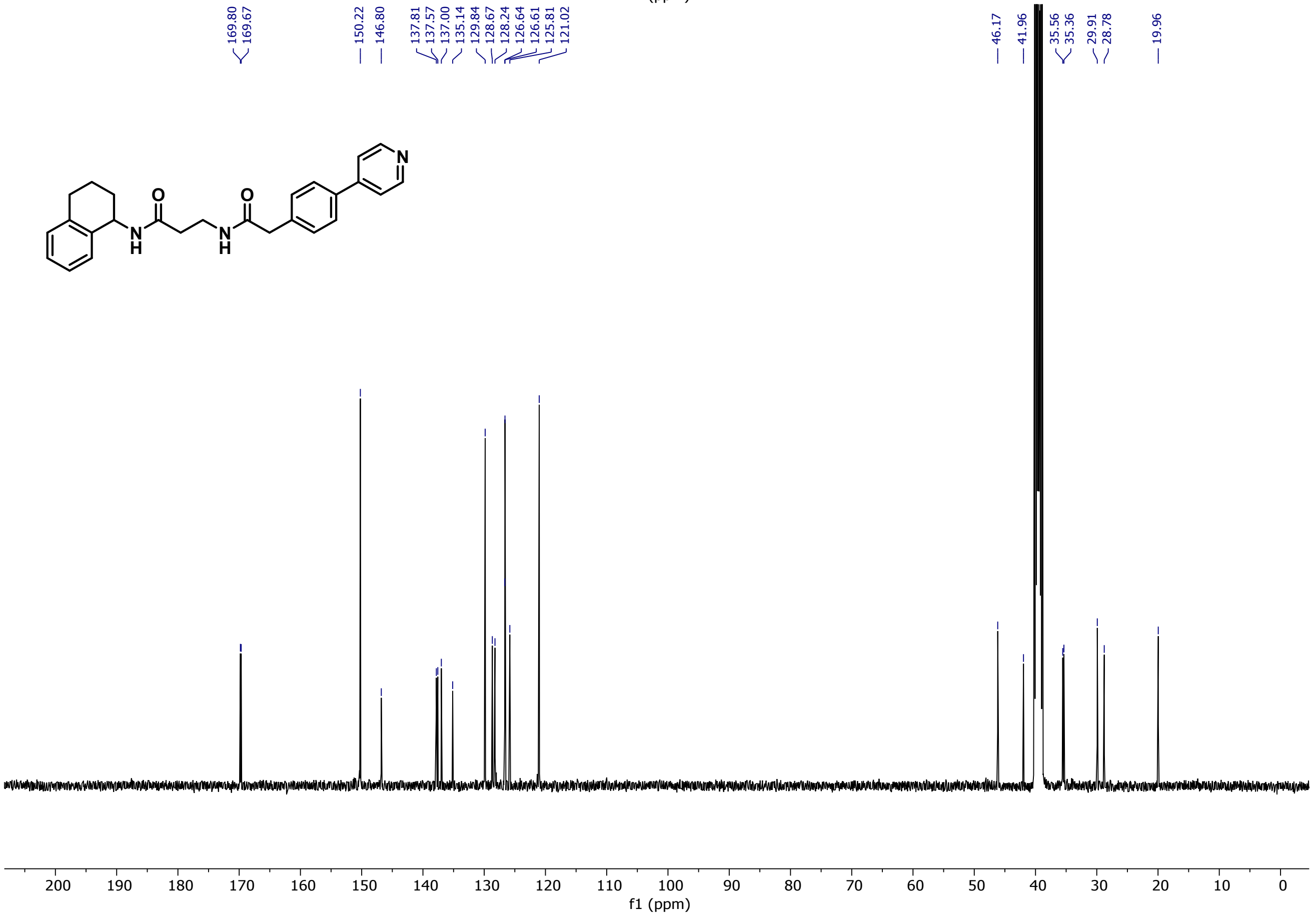

NMR spectra of 10q in DMSO-d_6.

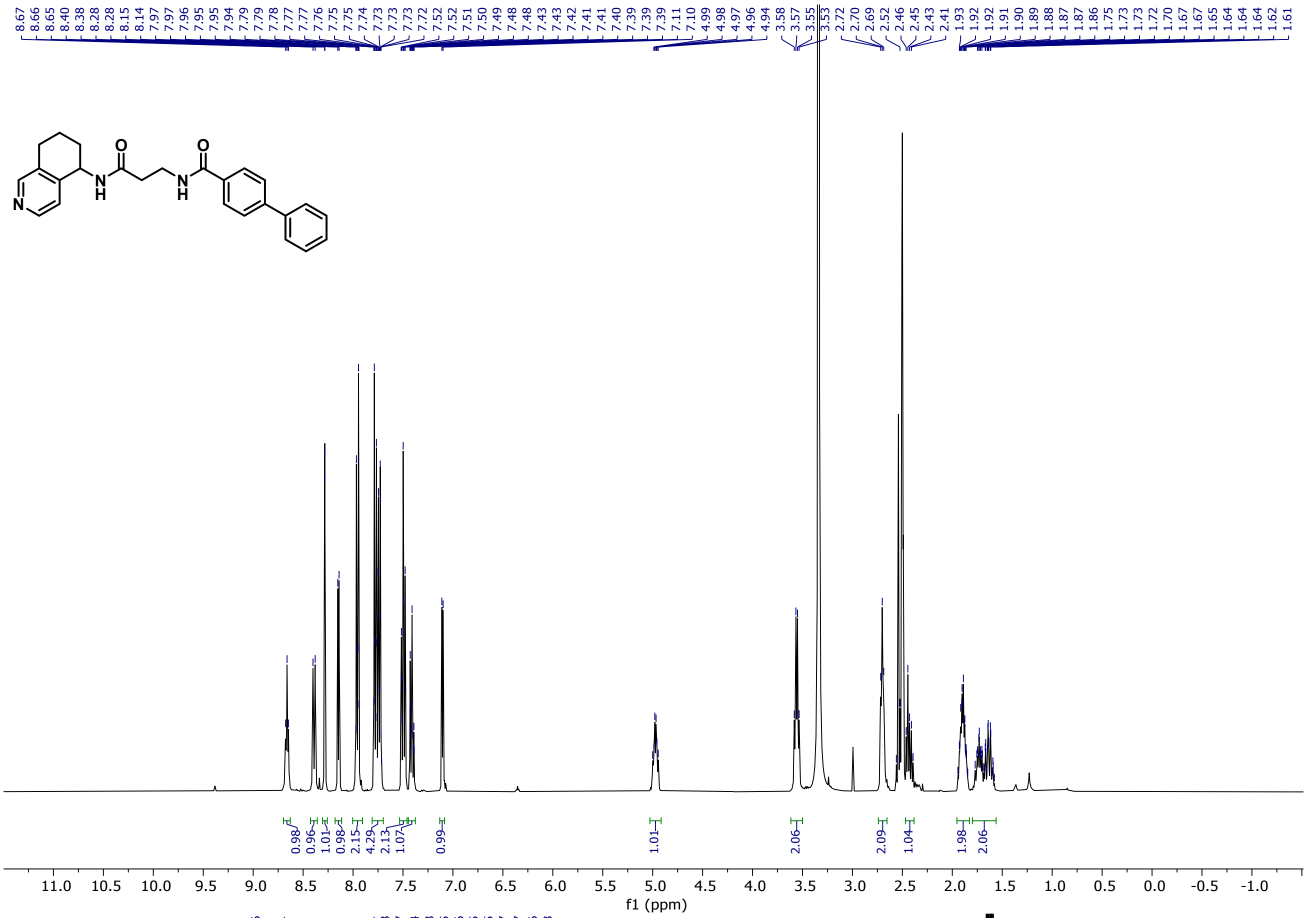

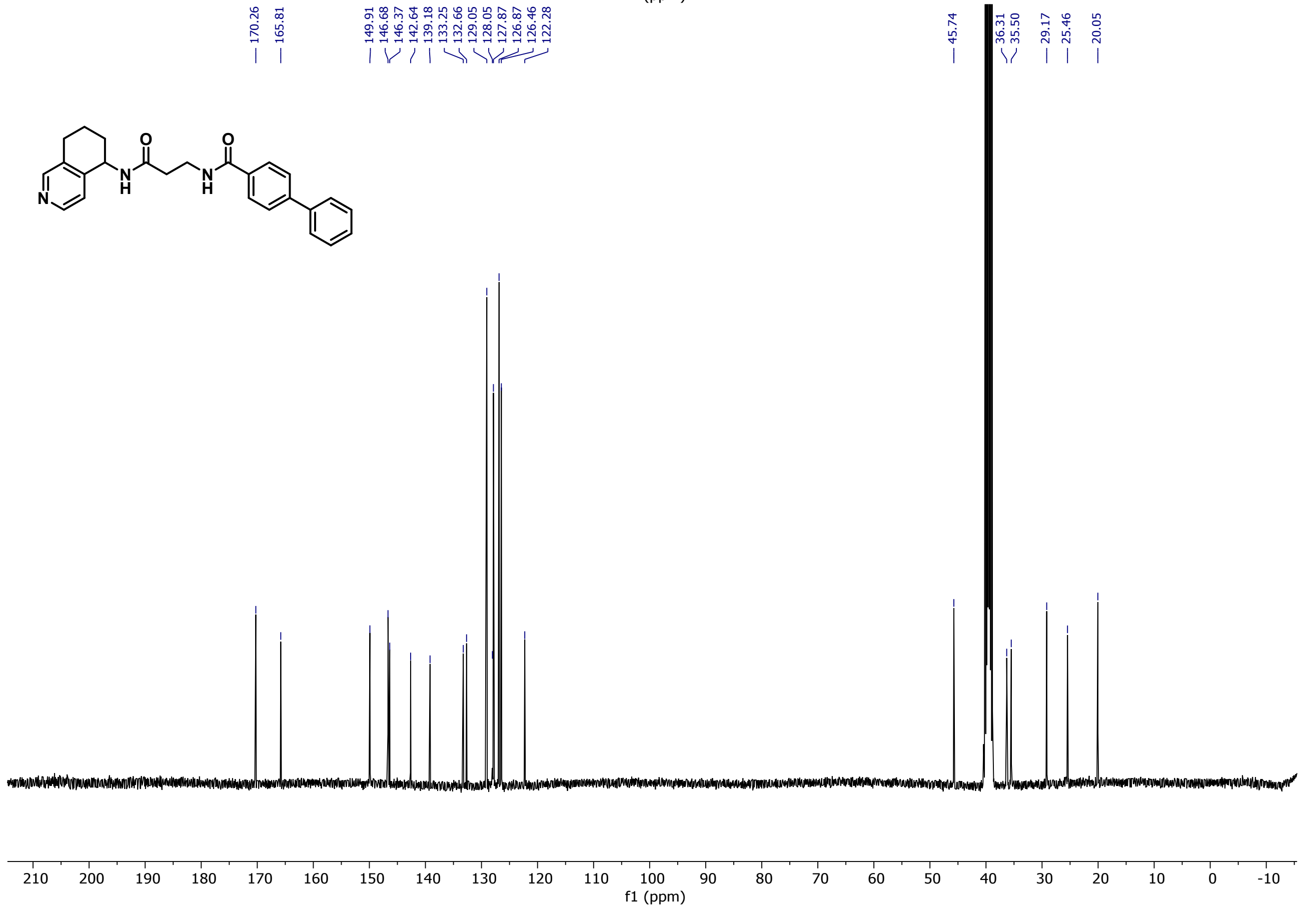

NMR spectra of 10r in DMSO-*d*$_6$.

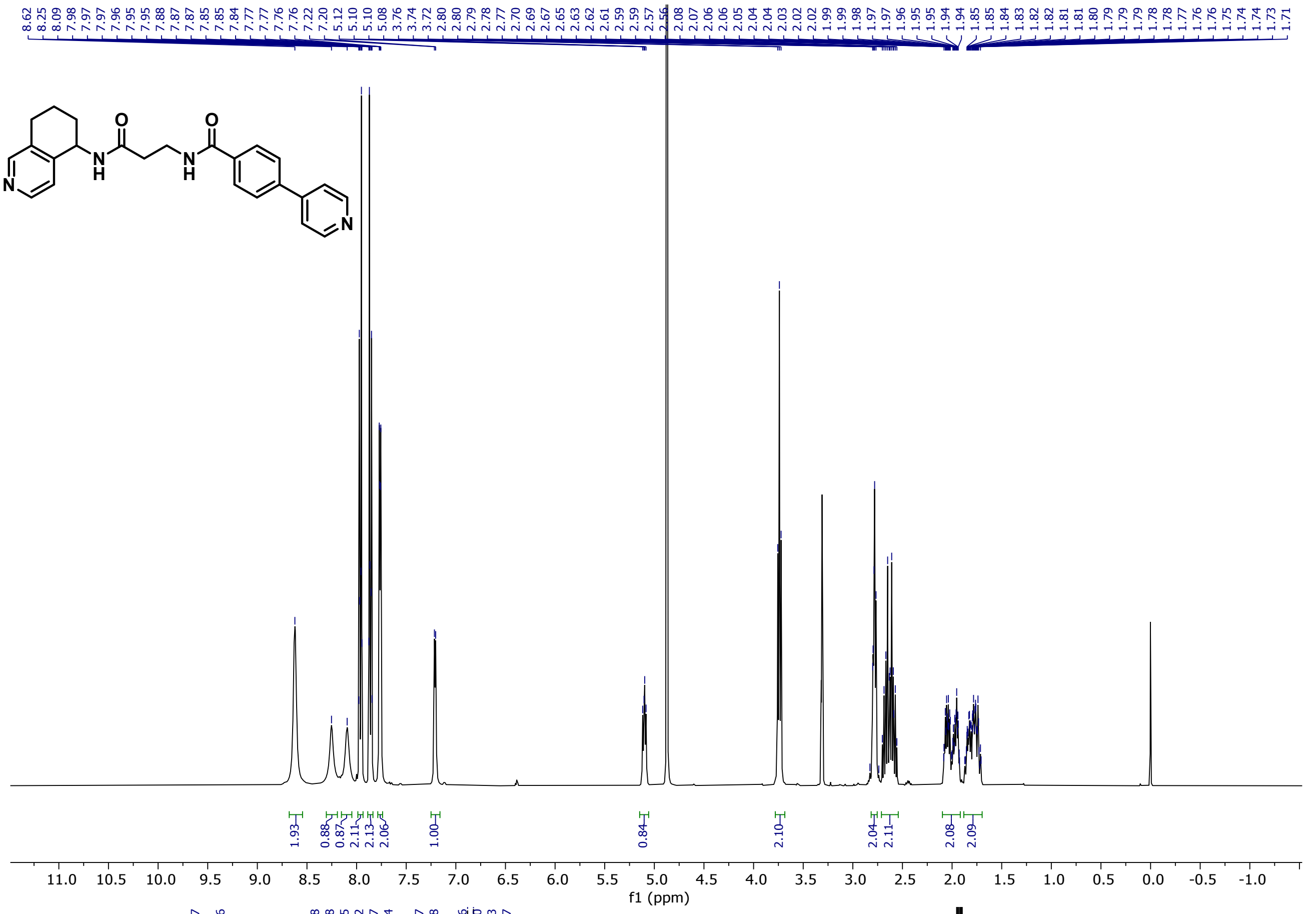

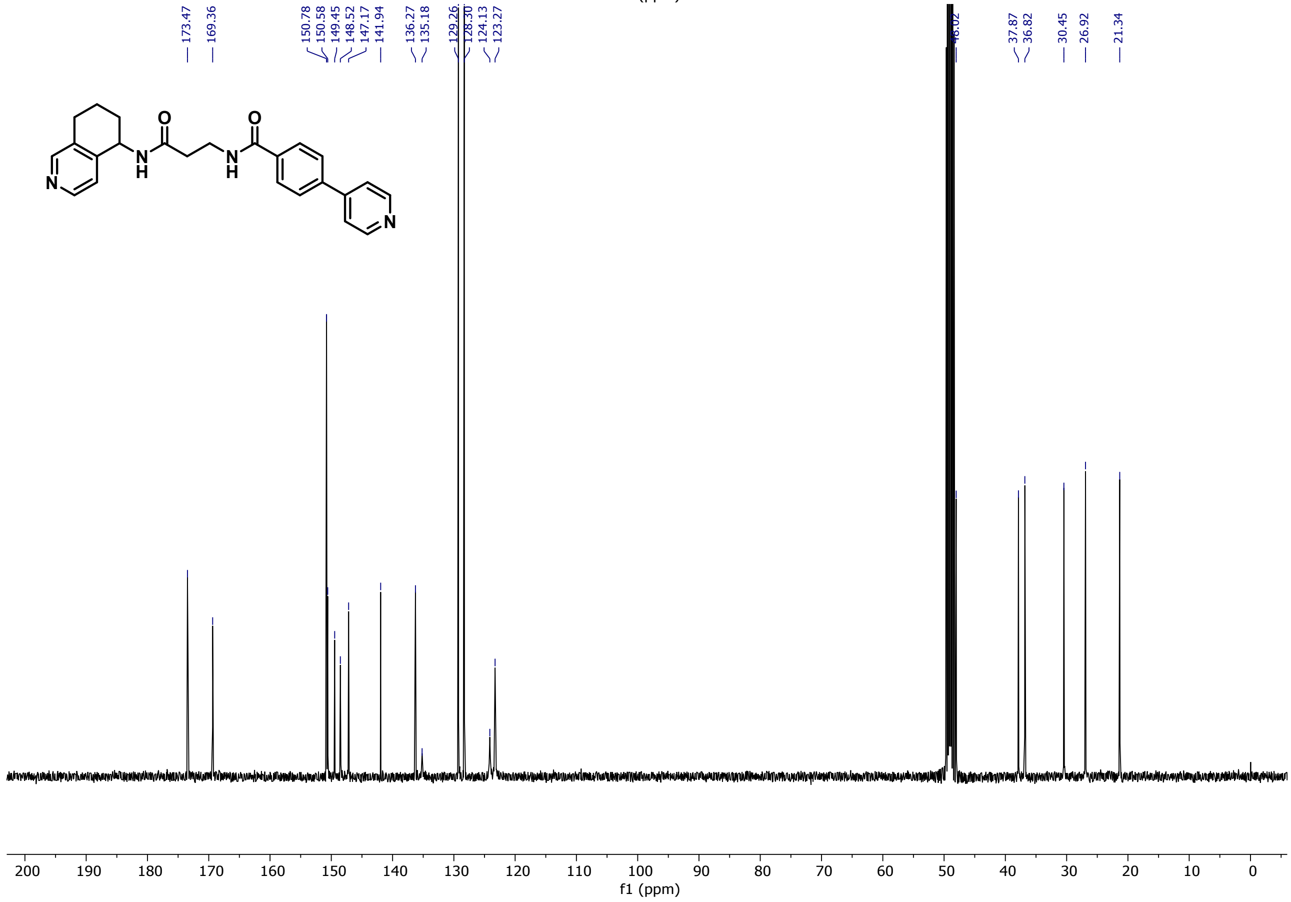

NMR spectra of 10s in MeOD-d_4.

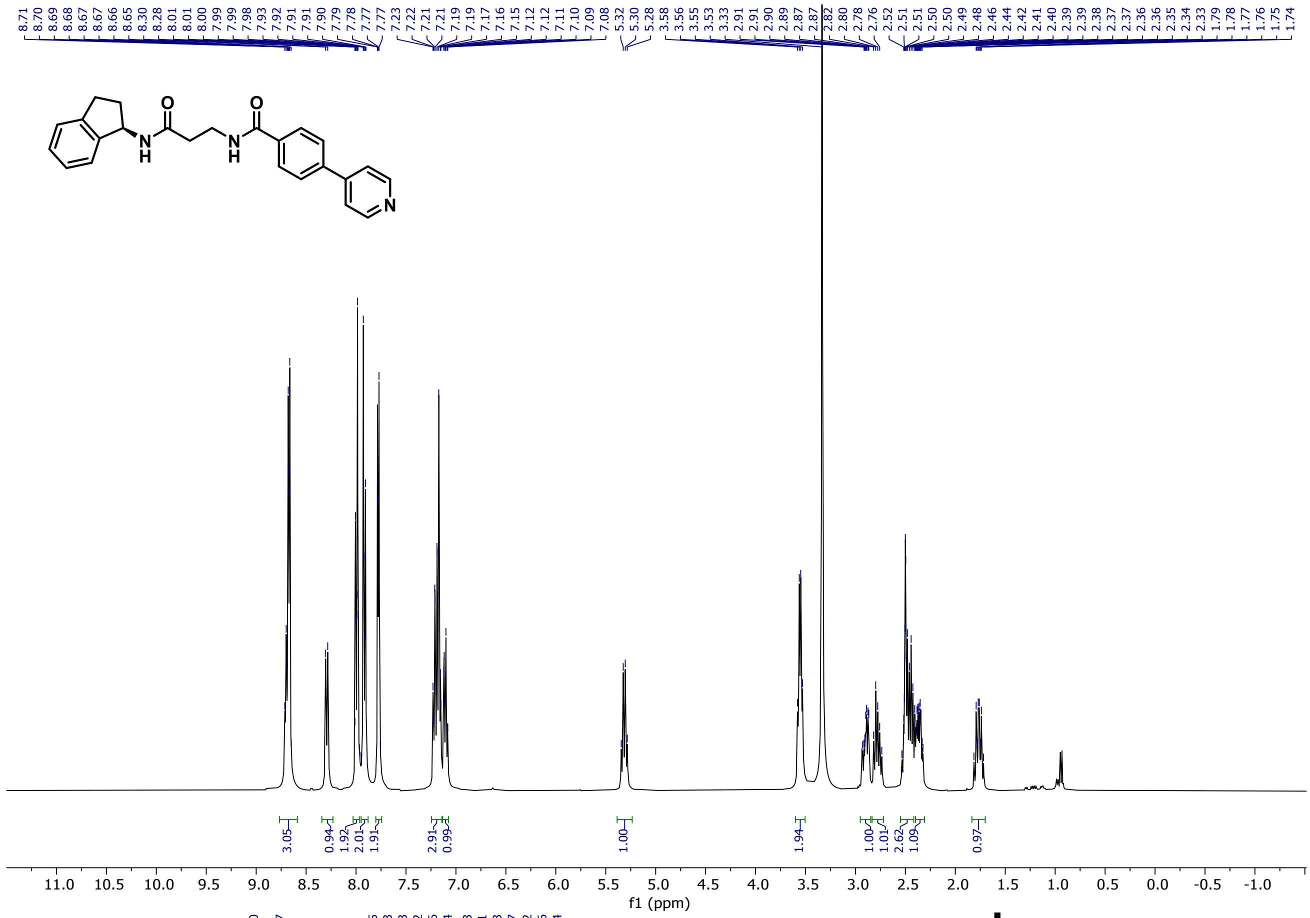

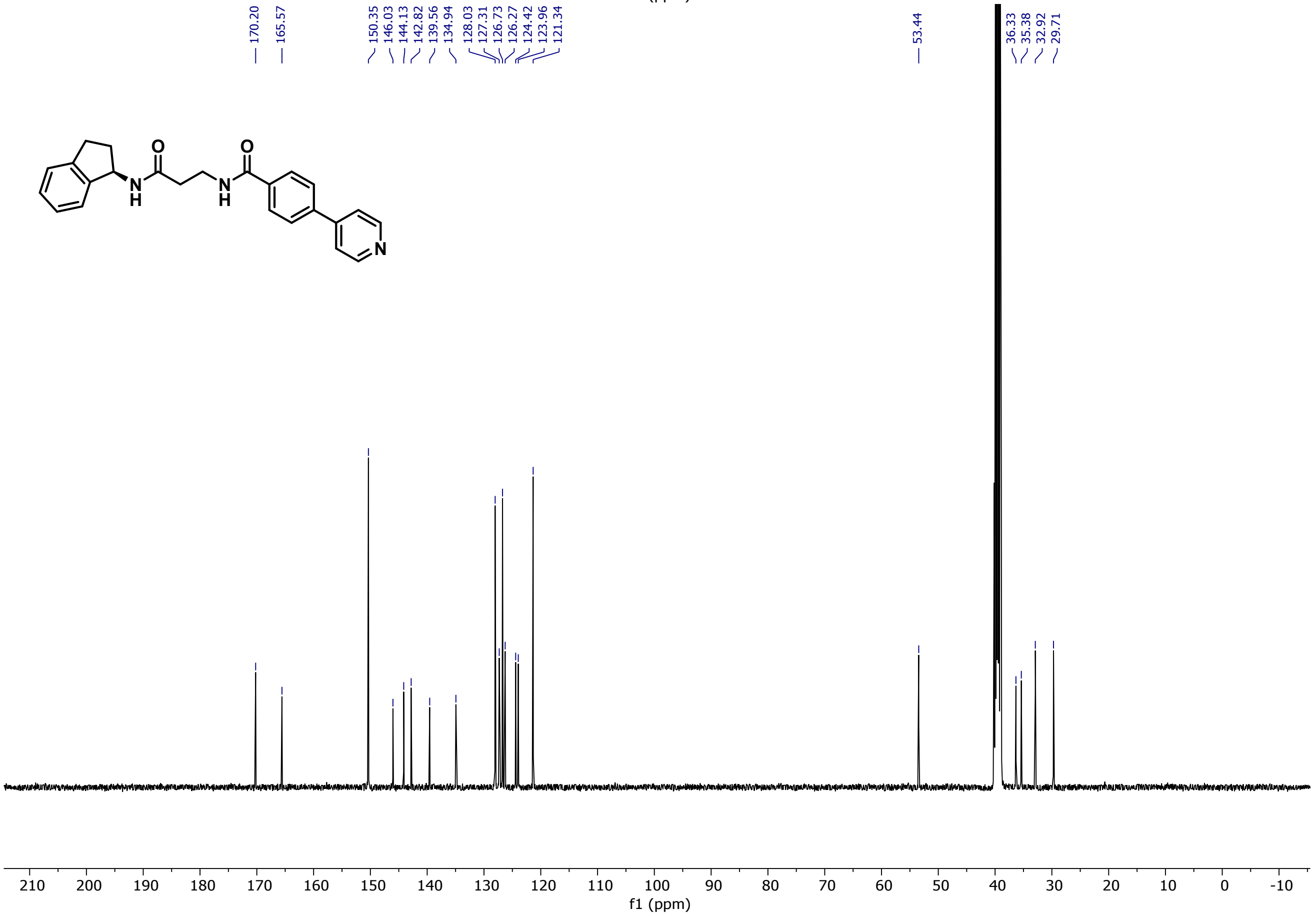

NMR spectra of 10t in DMSO-d_6.

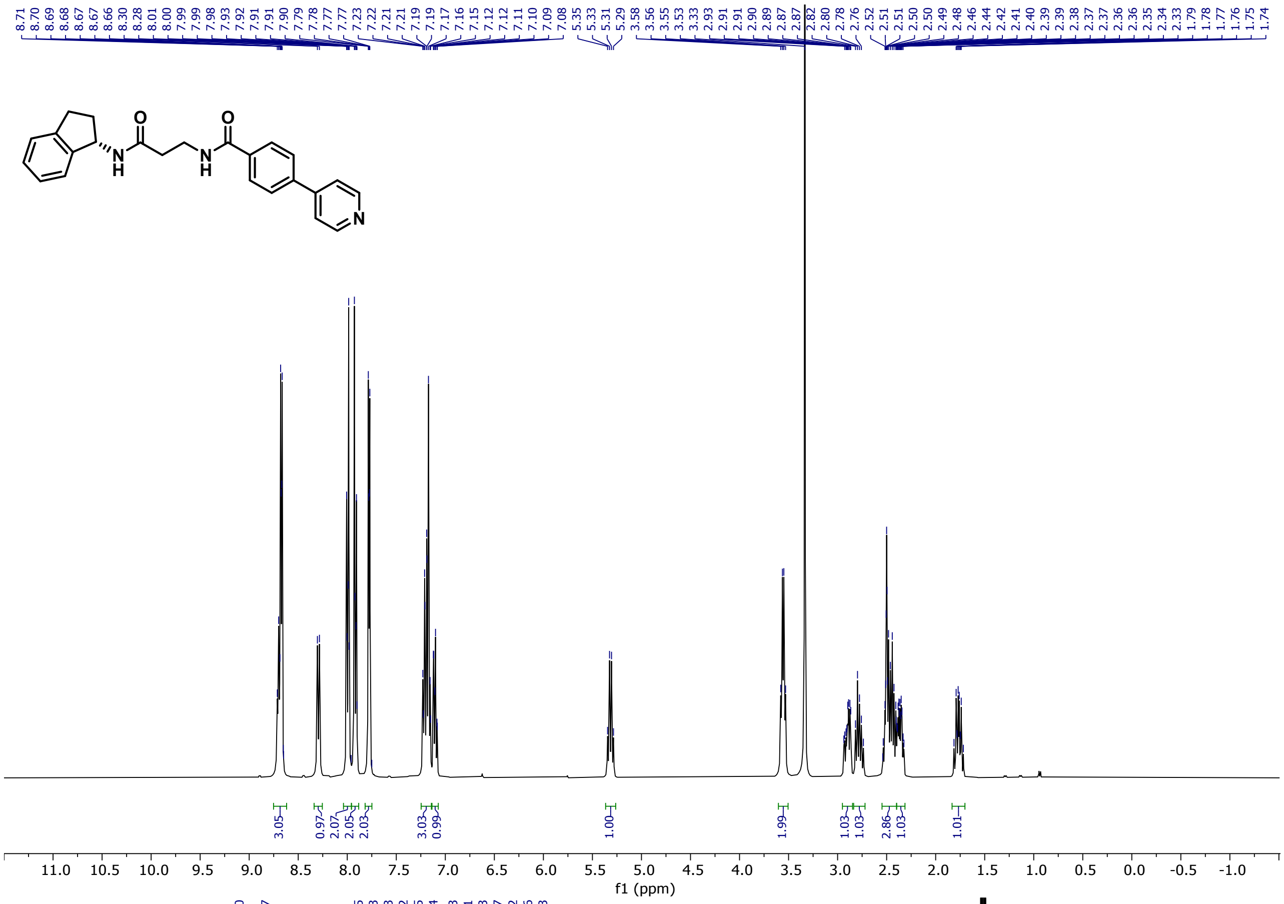

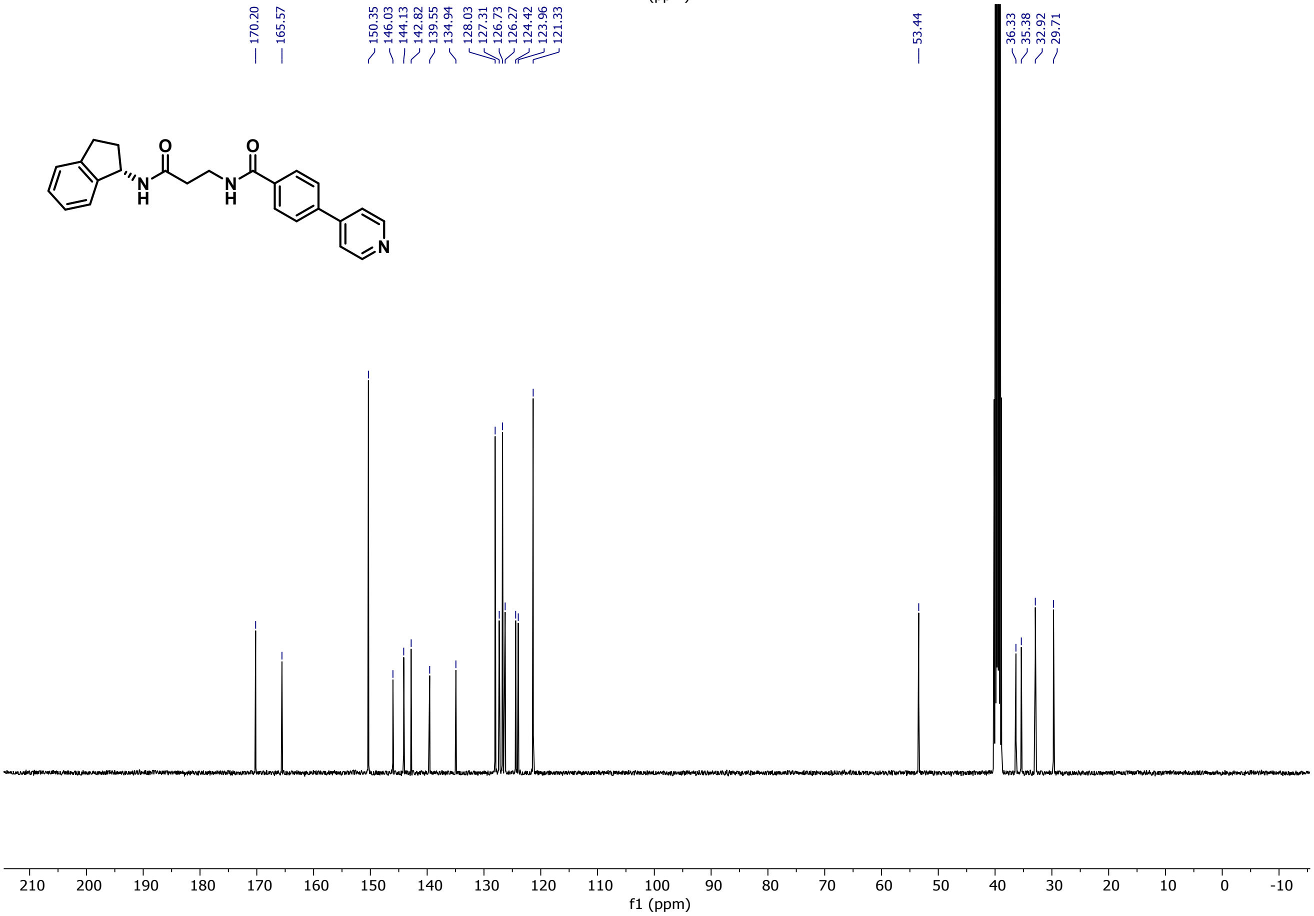

NMR spectra of 10u in DMSO-d_6.

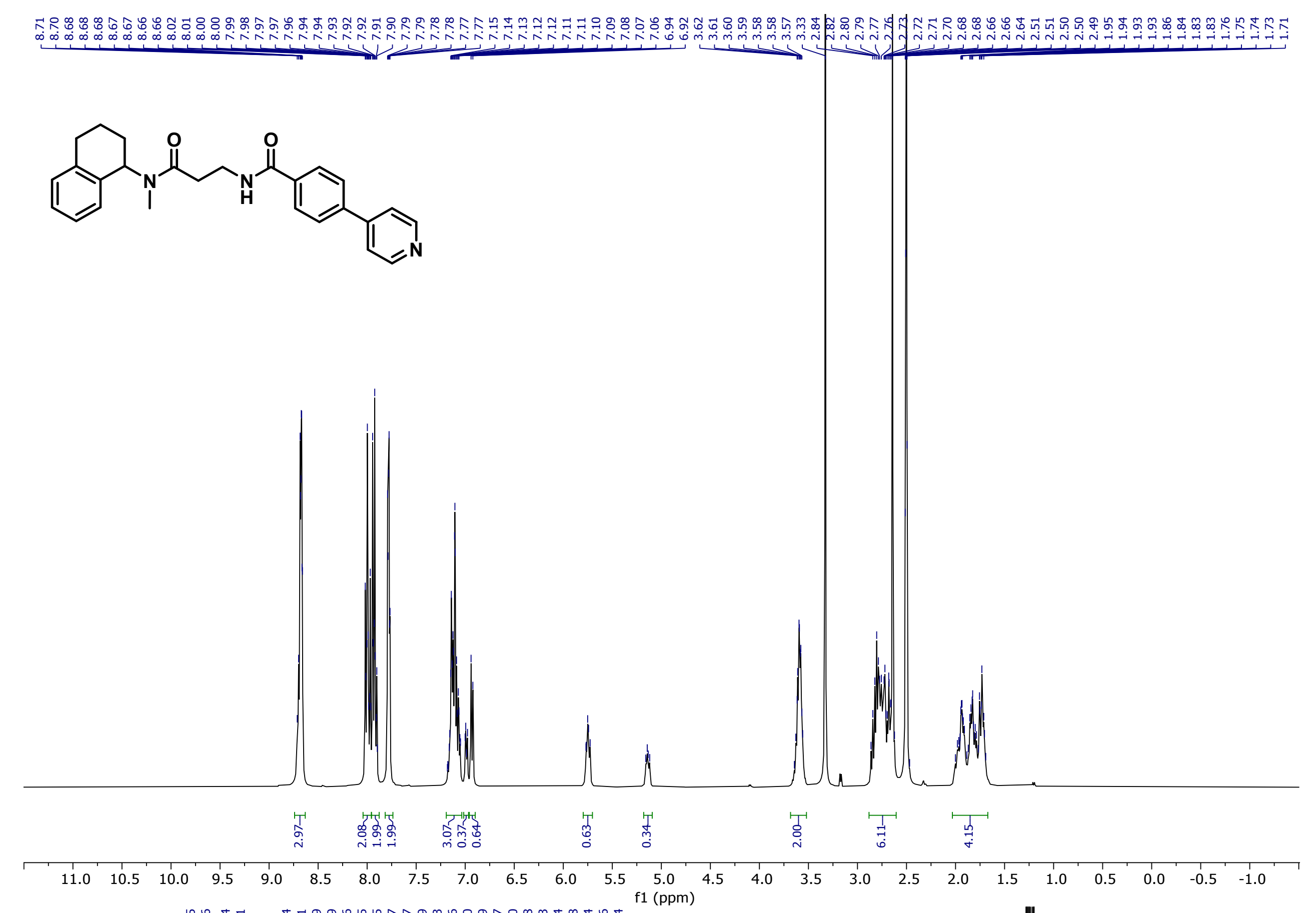

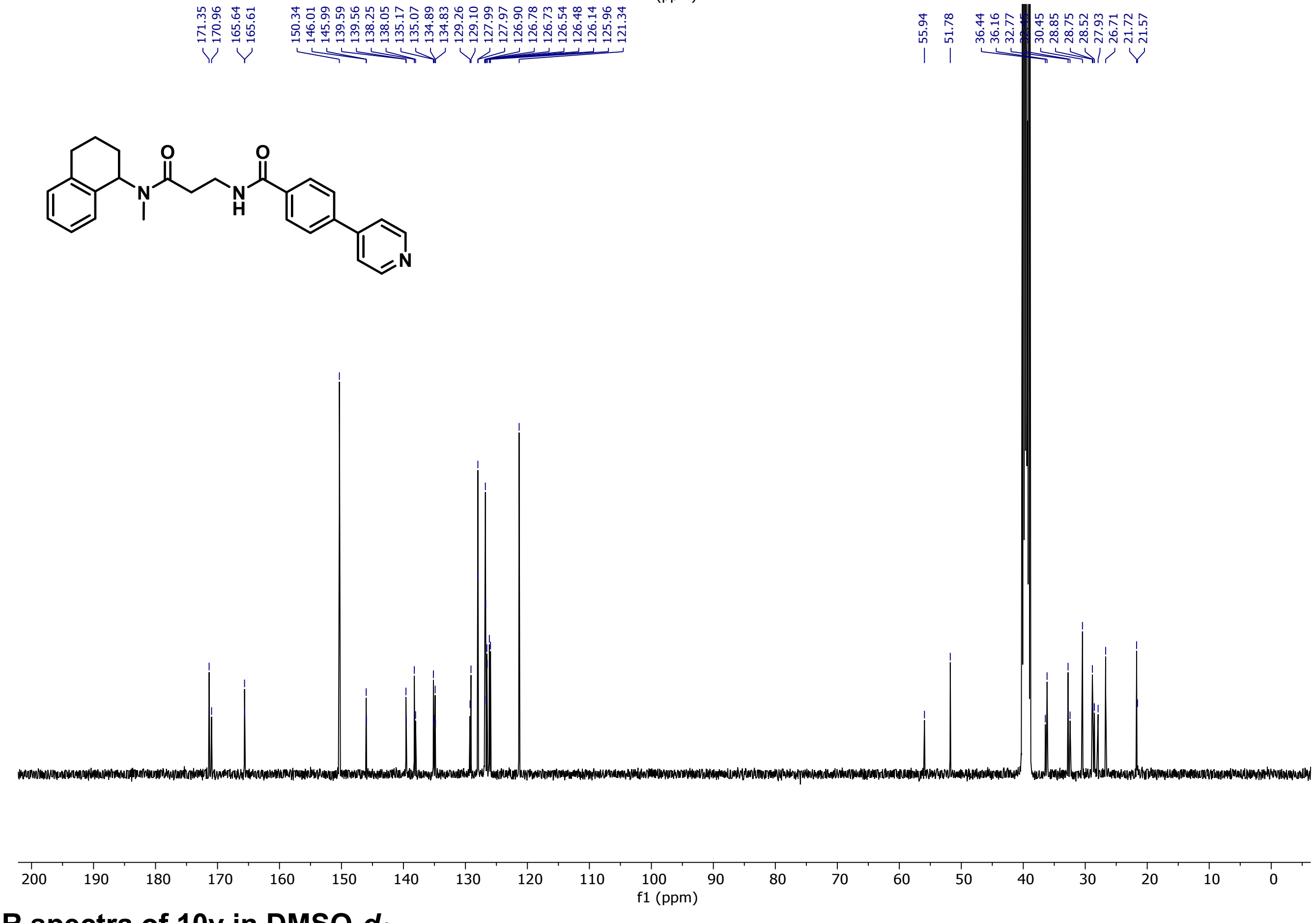

NMR spectra of 10v in DMSO-*d*$_6$.

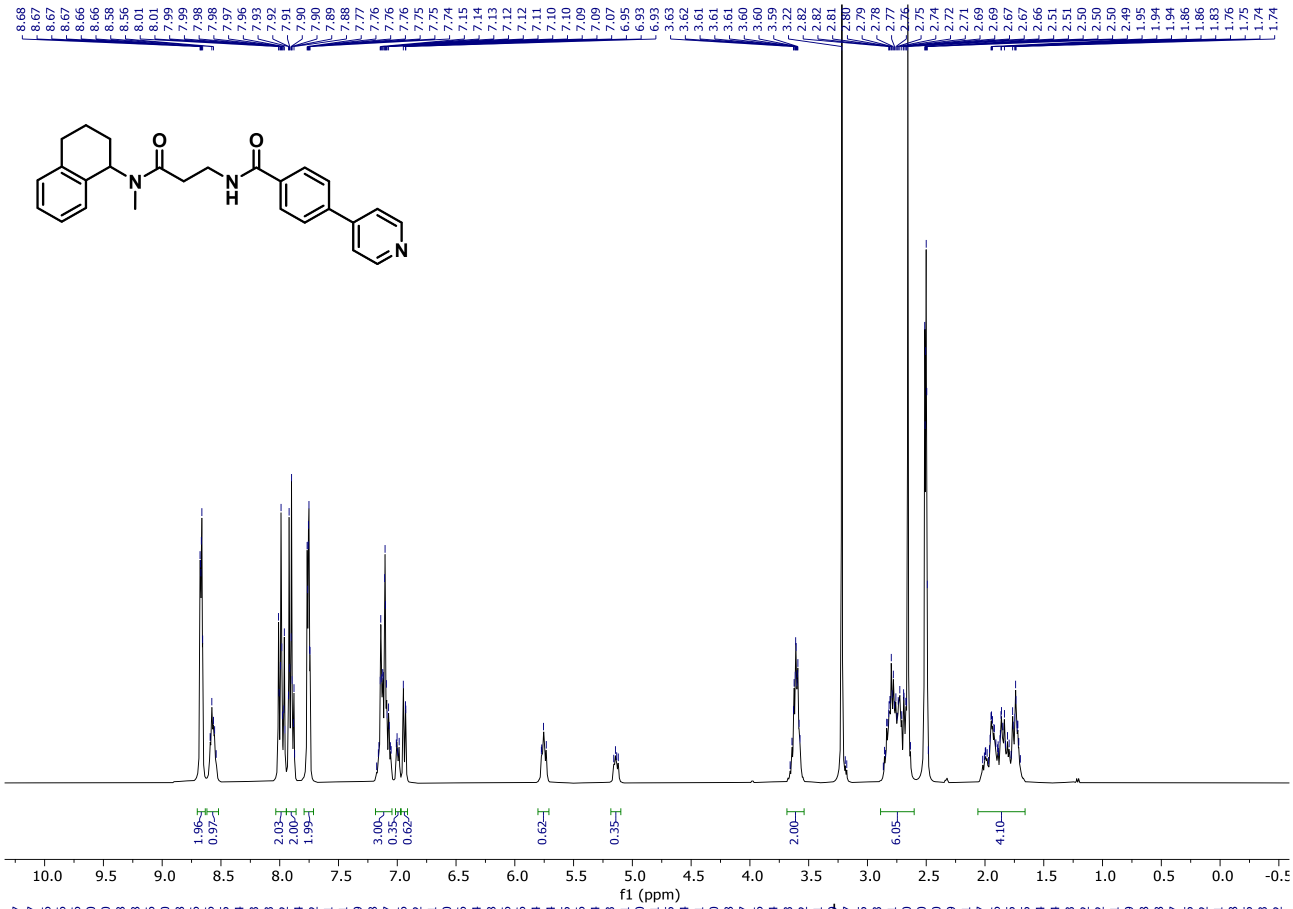

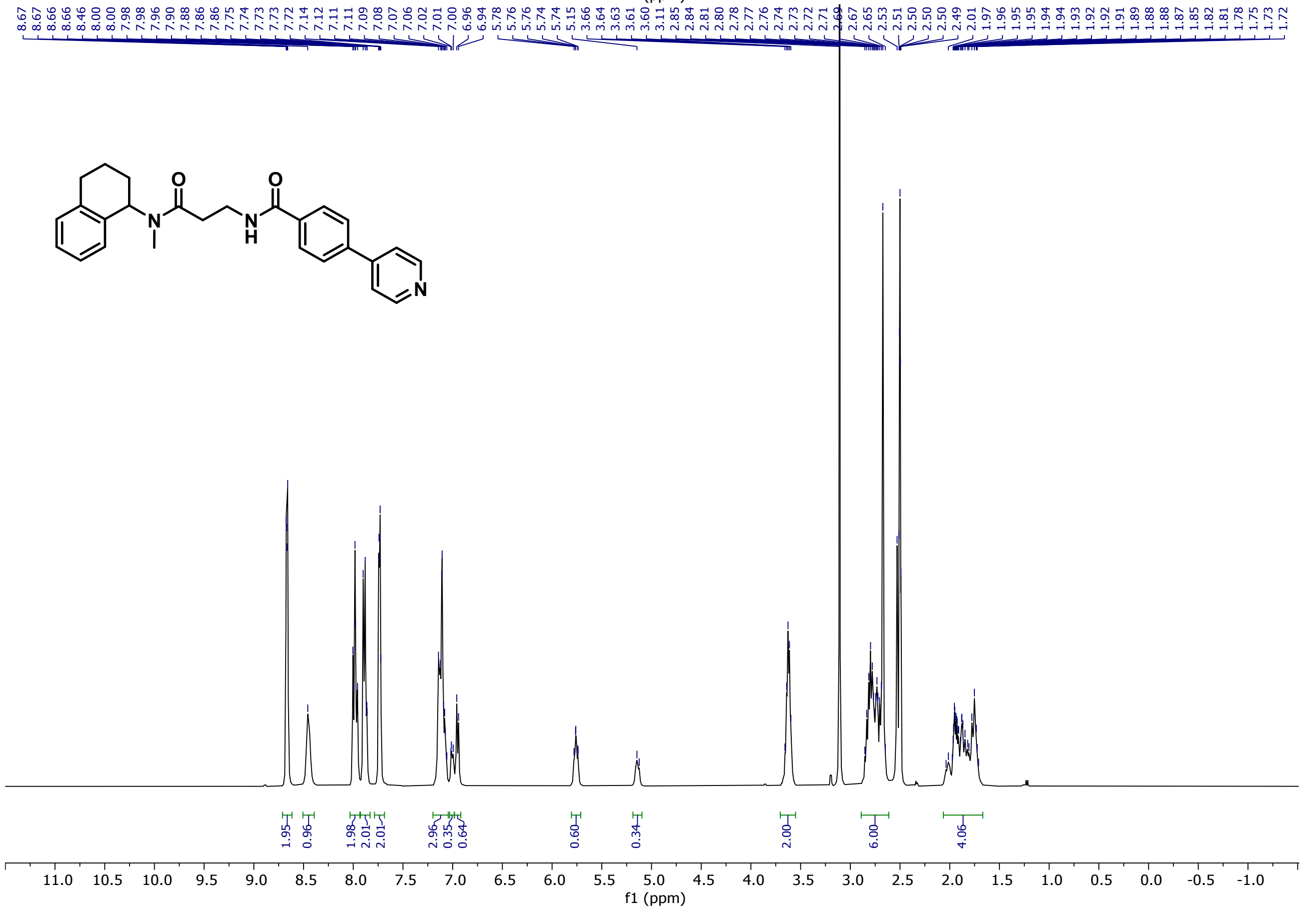

^{1}H NMR spectra of 10v in DMSO-d_6 at 49 °C (top) and 70 °C (bottom).

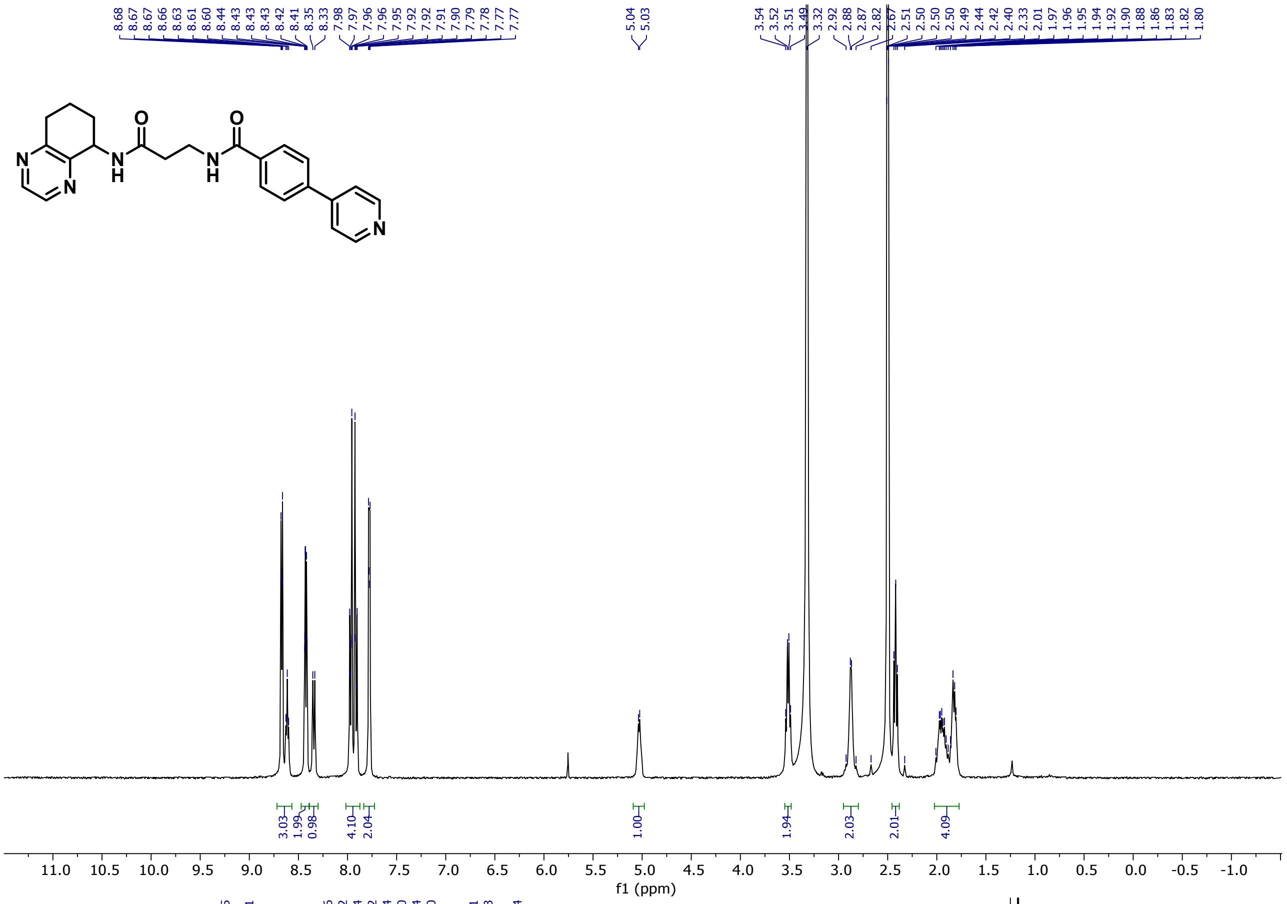

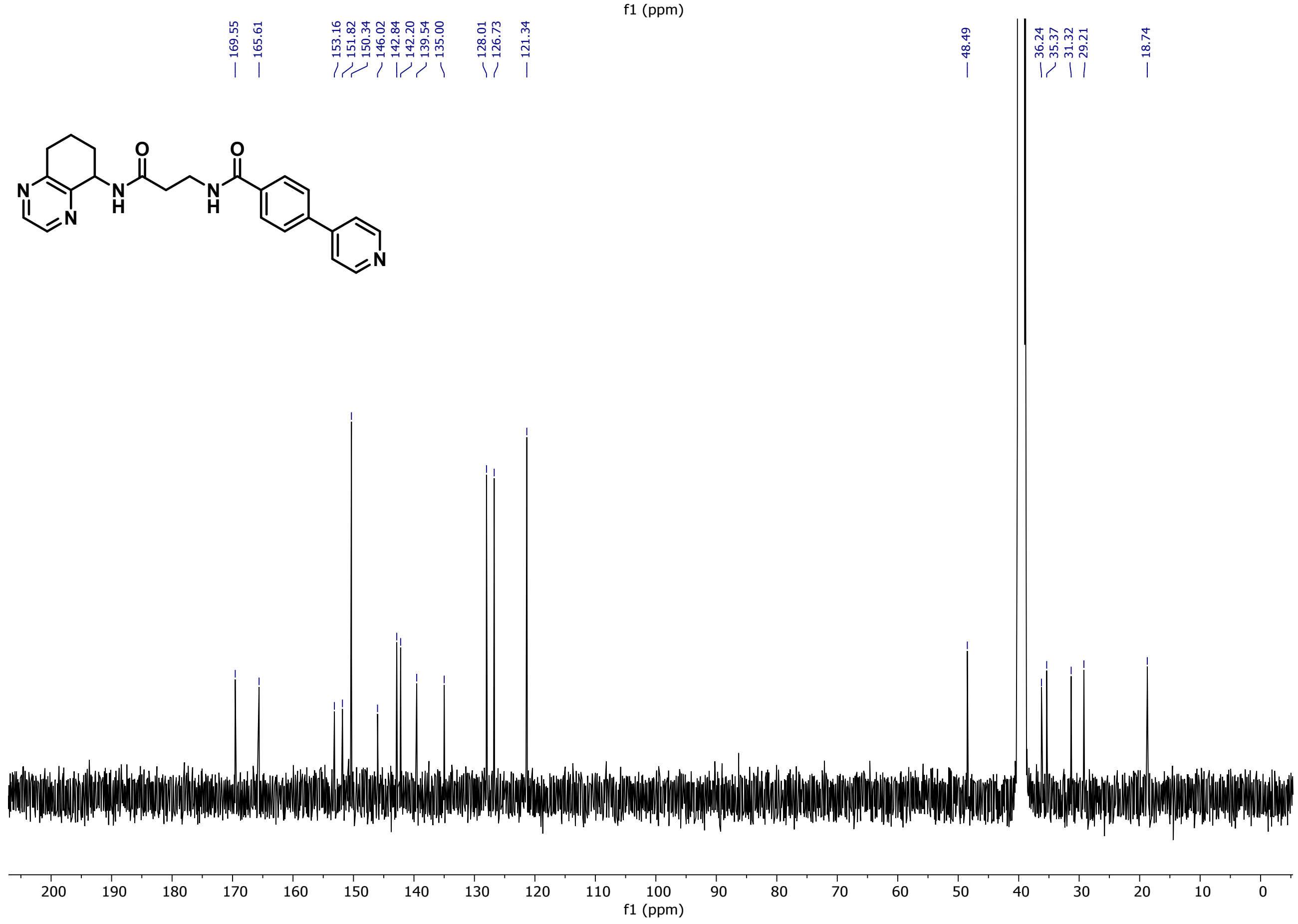

NMR spectra of 10w in DMSO-d_6.

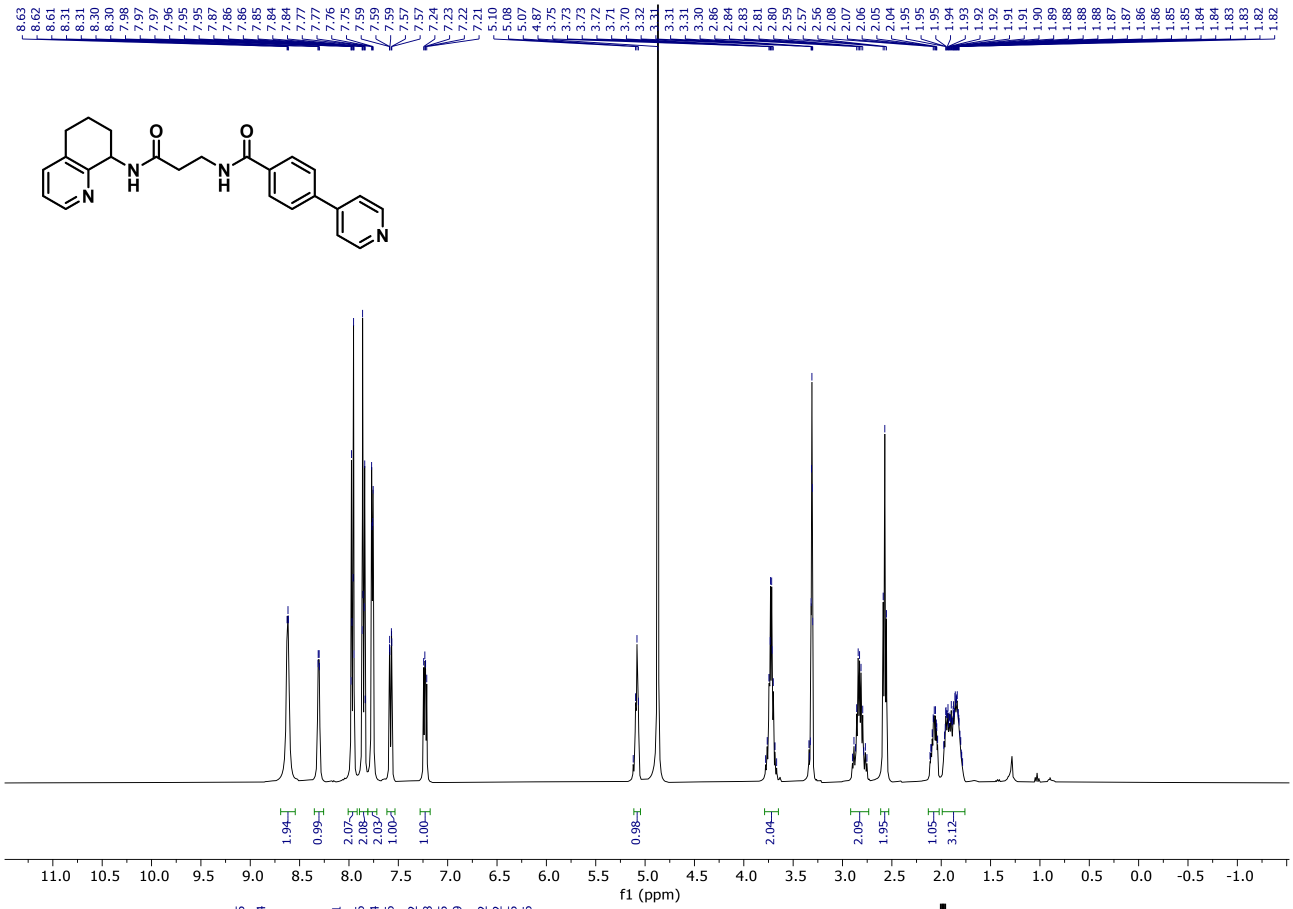

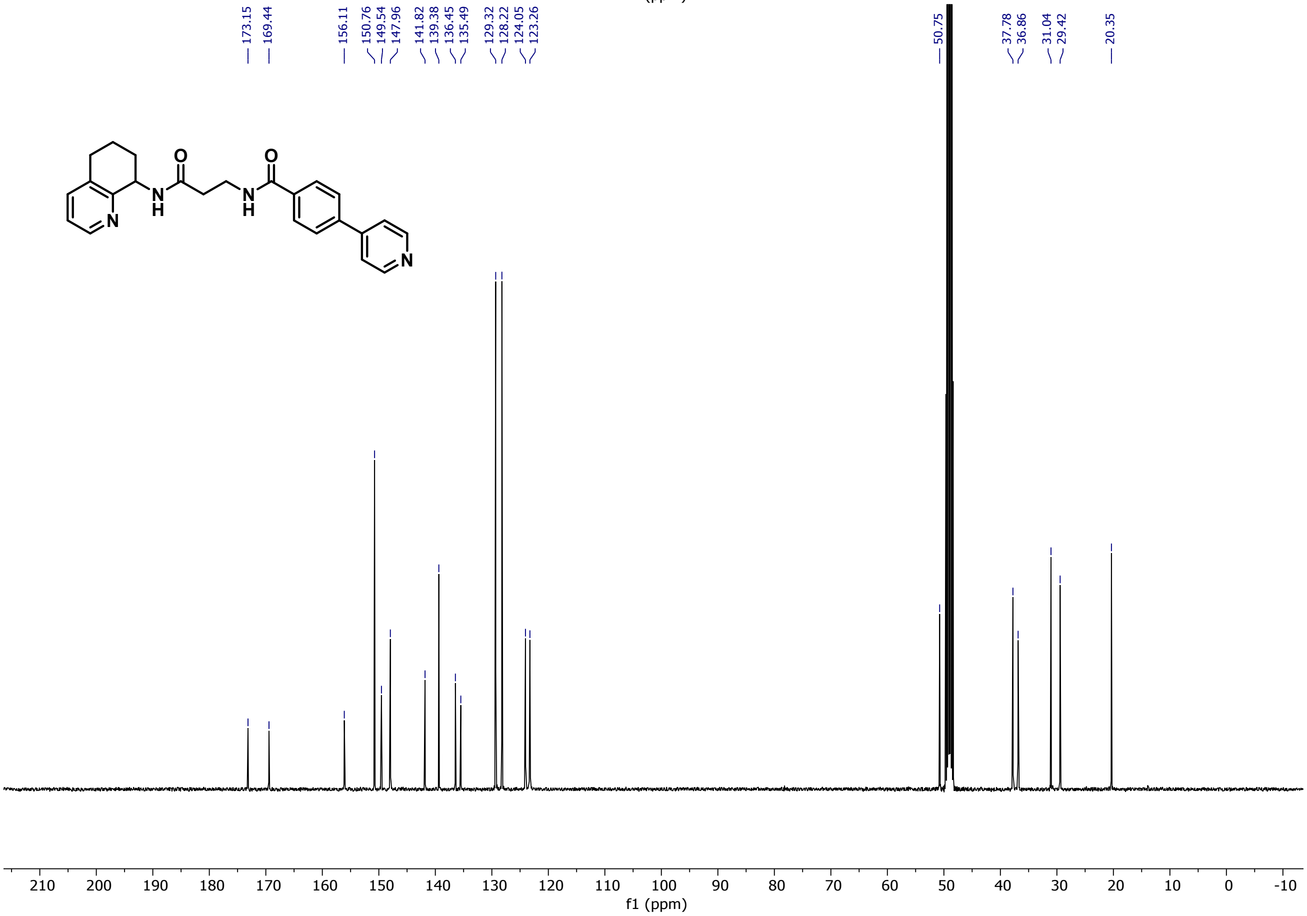

NMR spectra of 10x in DMSO-*d*$_6$.

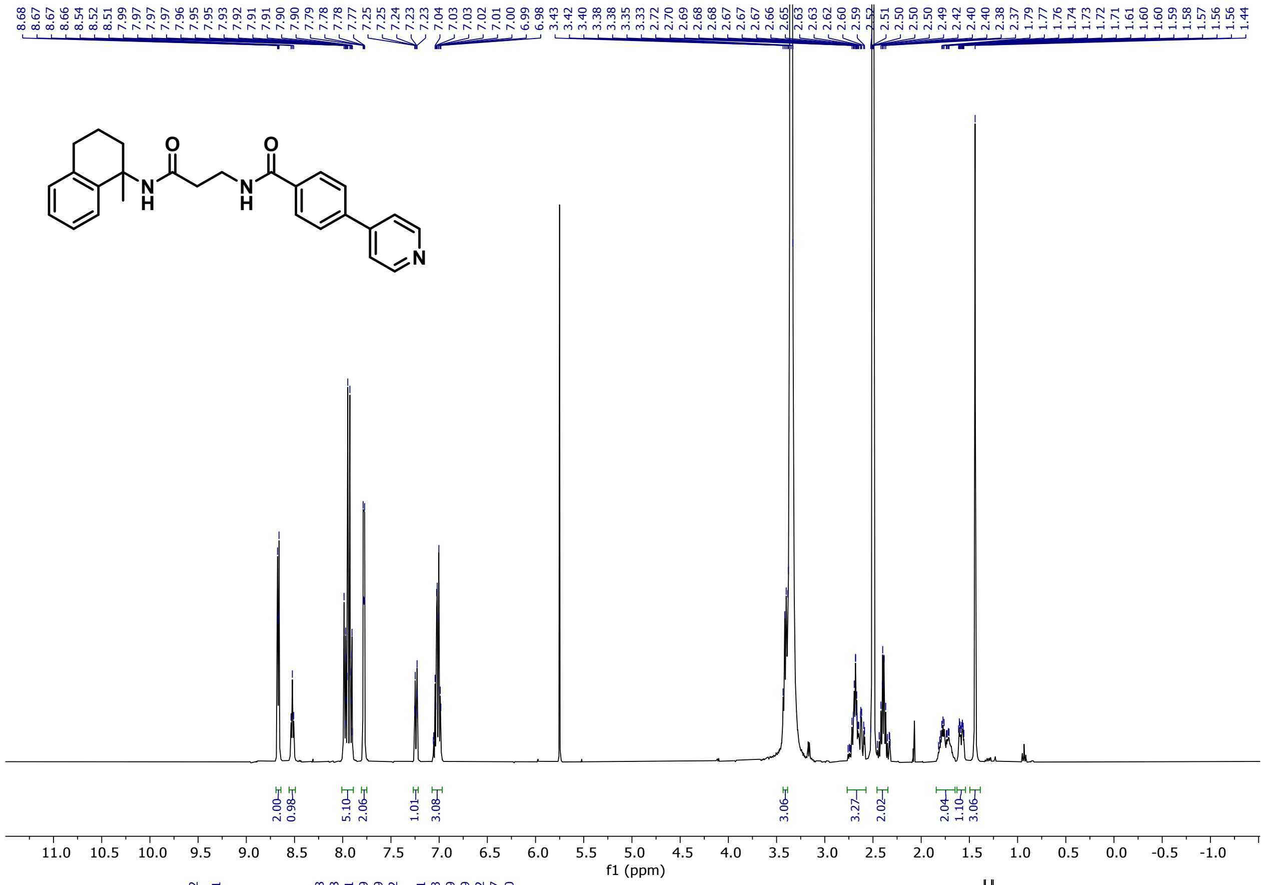

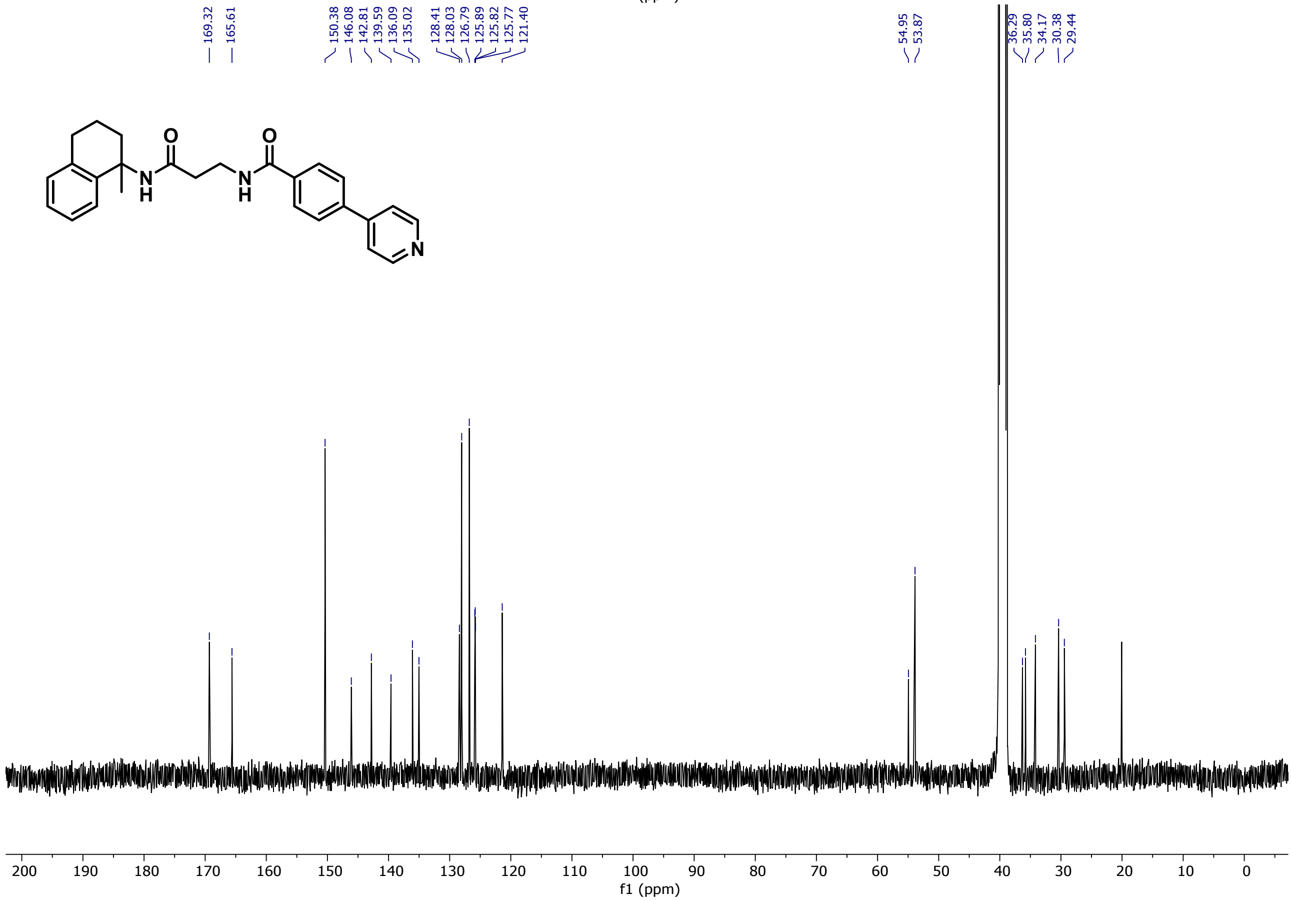

NMR spectra of 10y in DMSO-d_6.

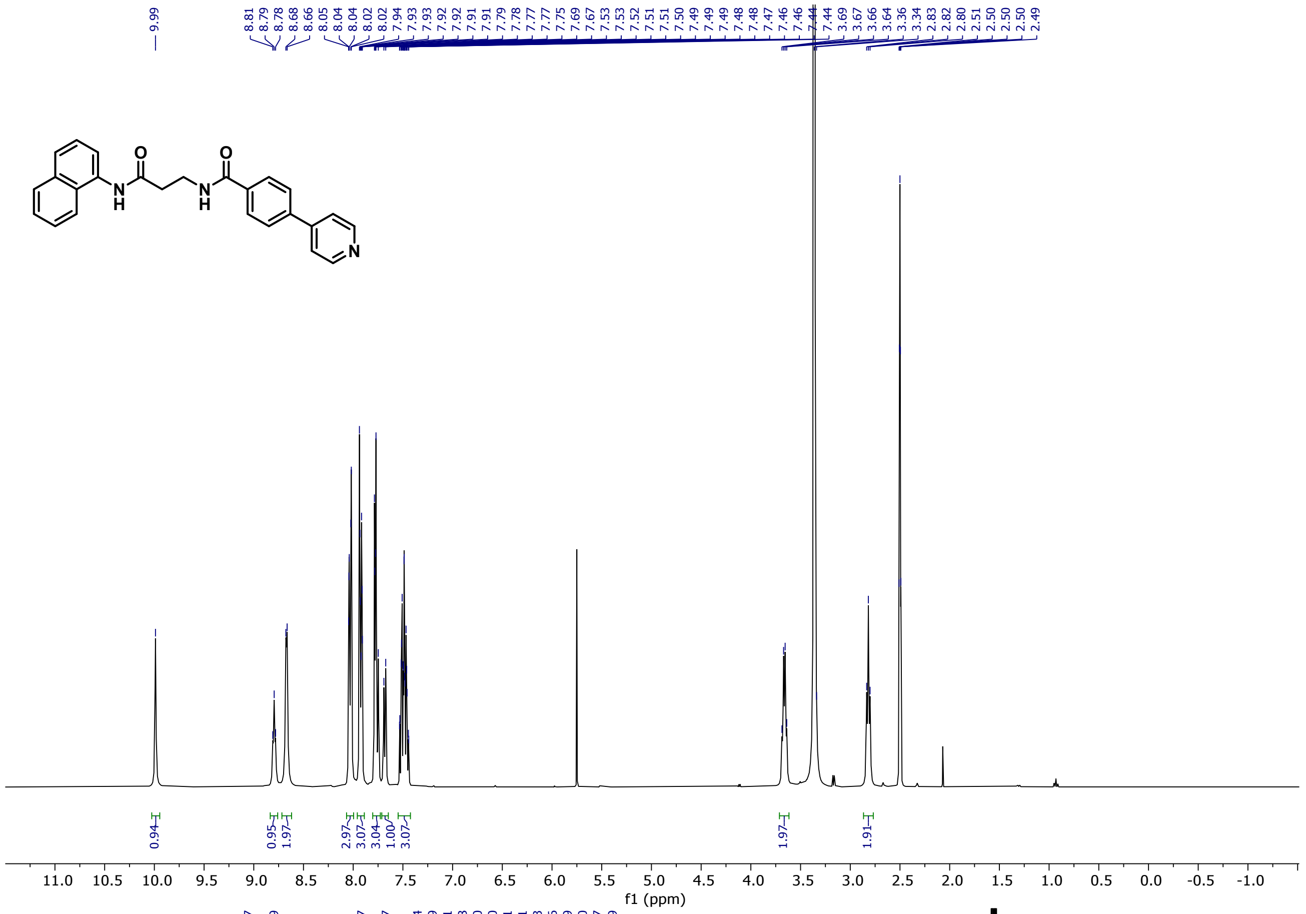

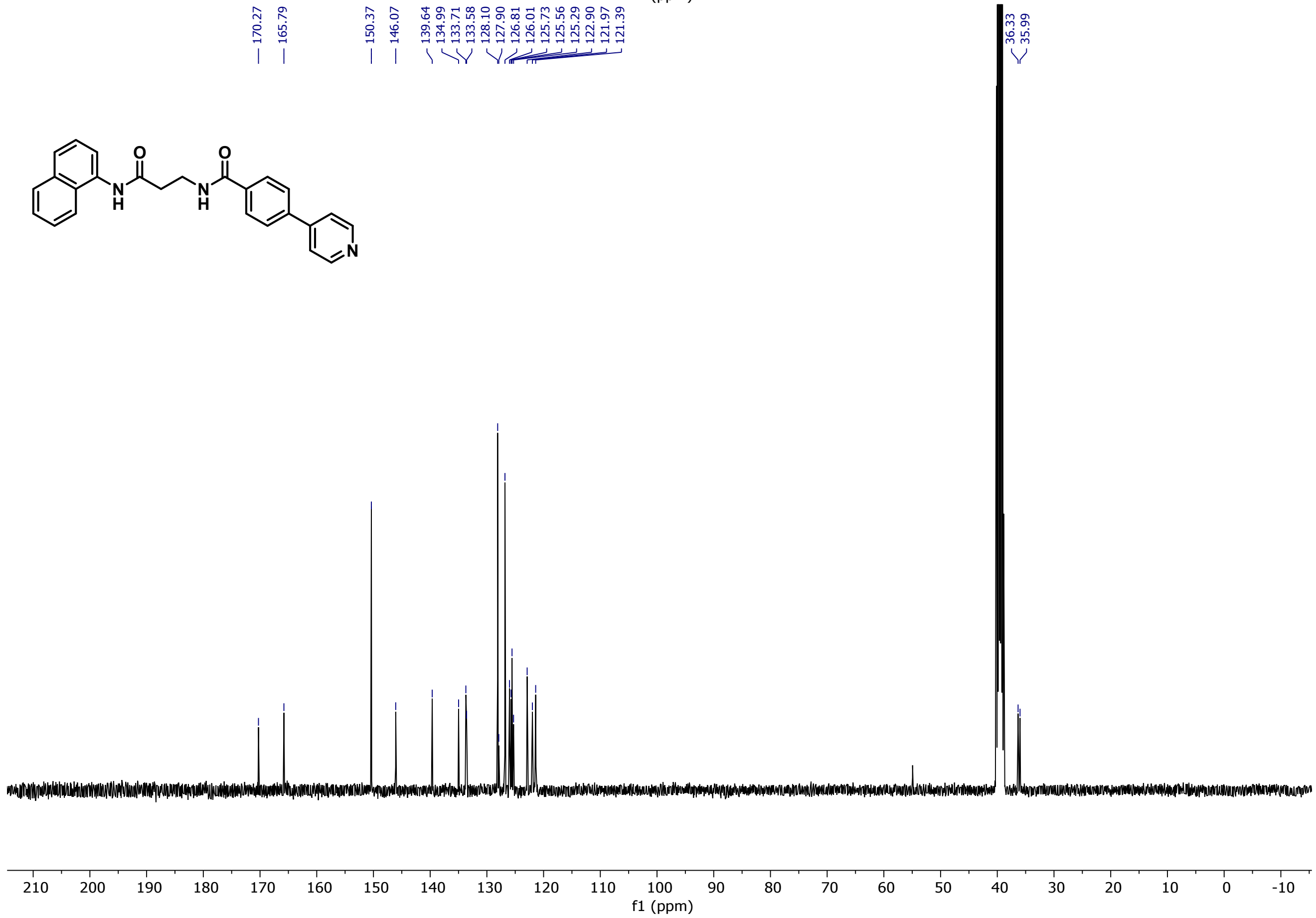

NMR spectra of 10z in DMSO-d_6.

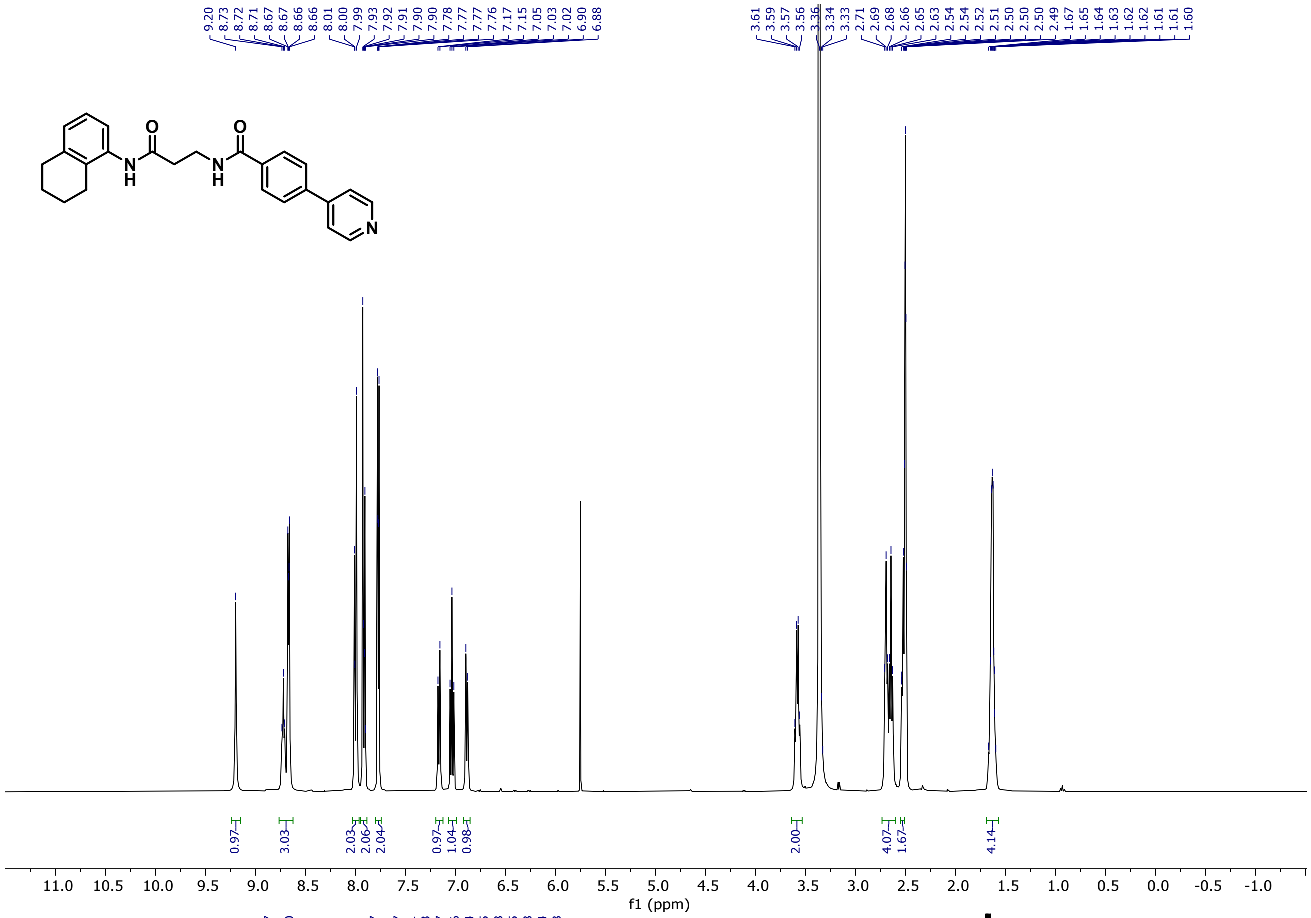

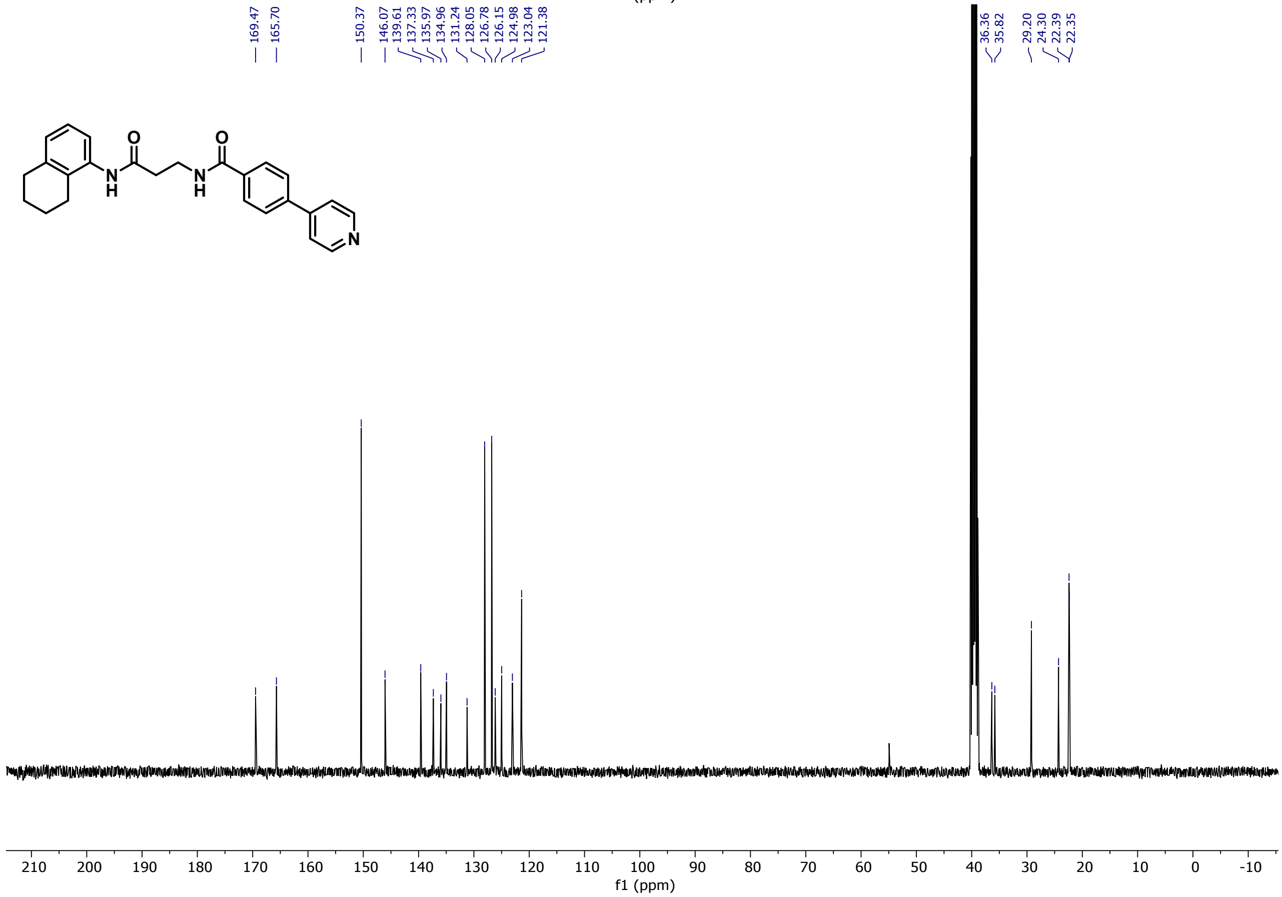

NMR spectra of 10aa in DMSO-d_6.

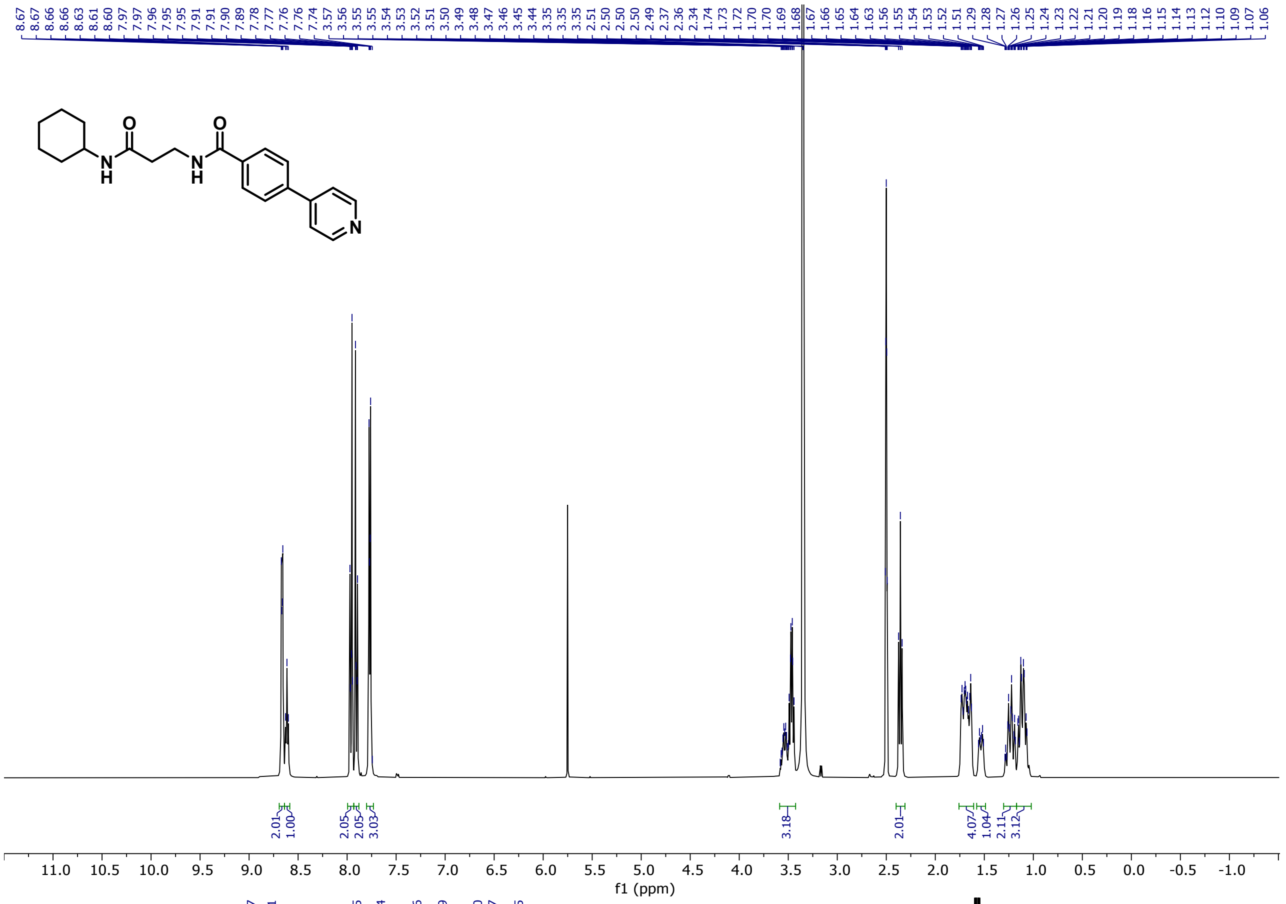

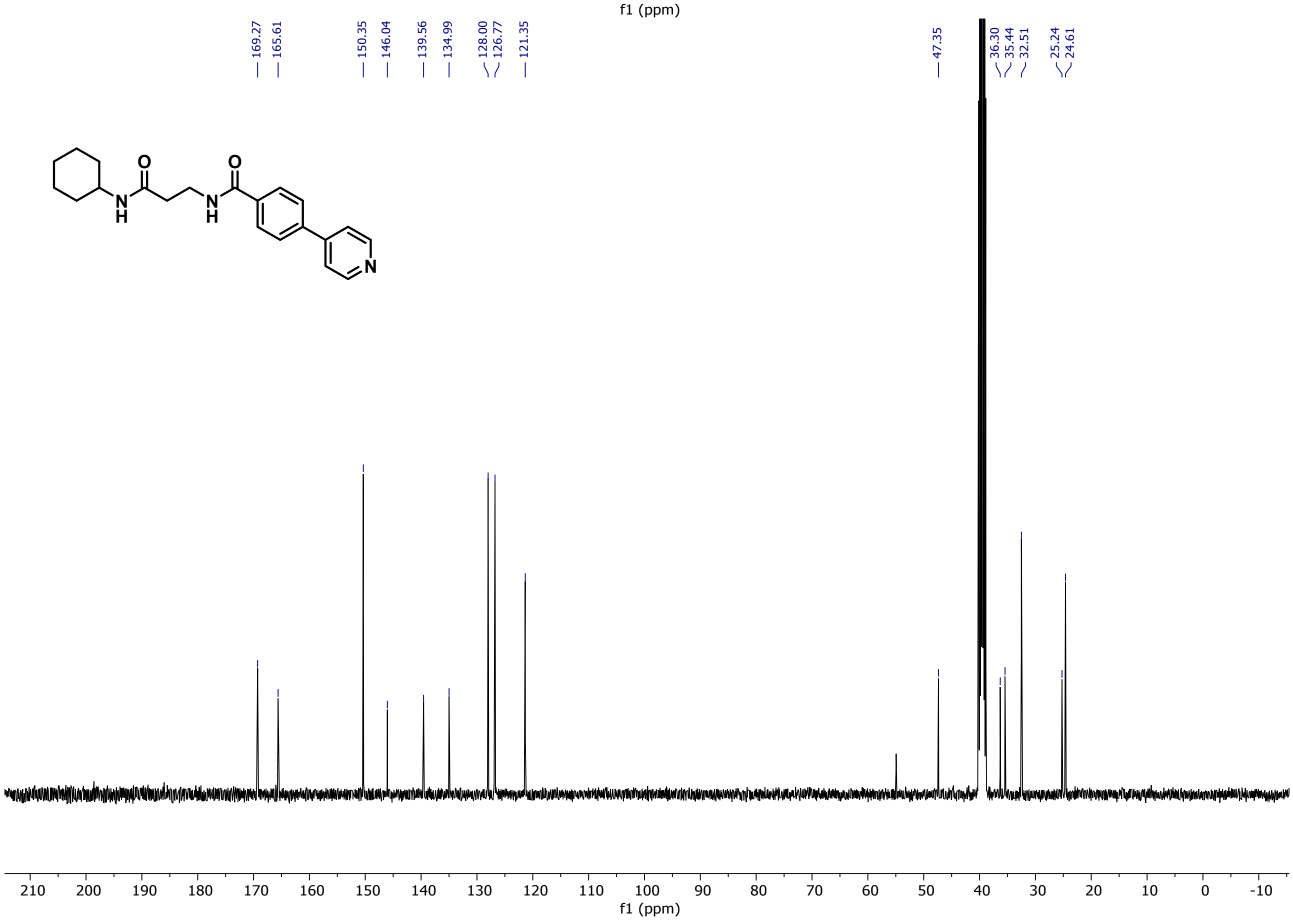

NMR spectra of 10ab in DMSO-d_6.

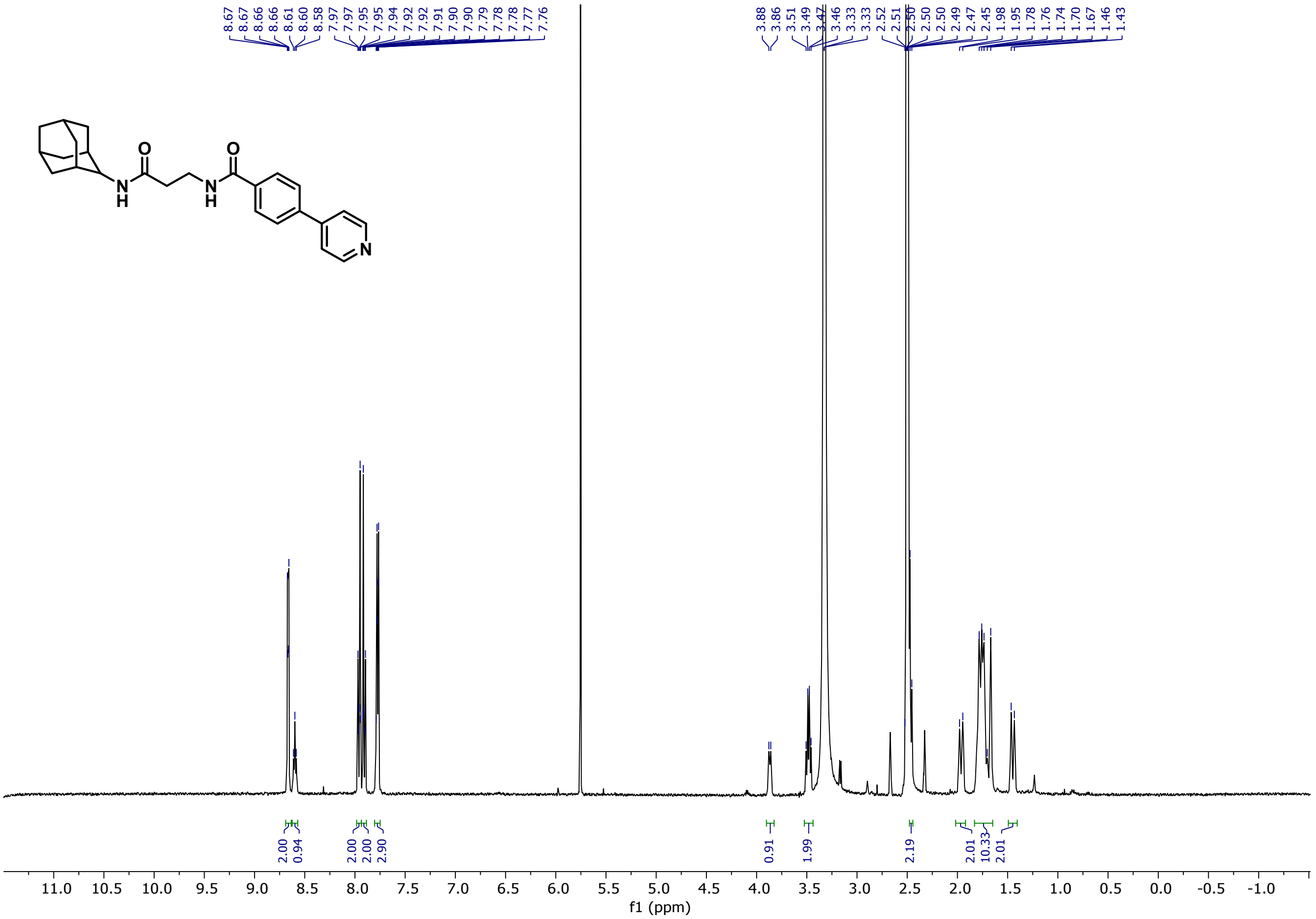

^{1}H NMR spectrum of 10ac in DMSO-d_6.

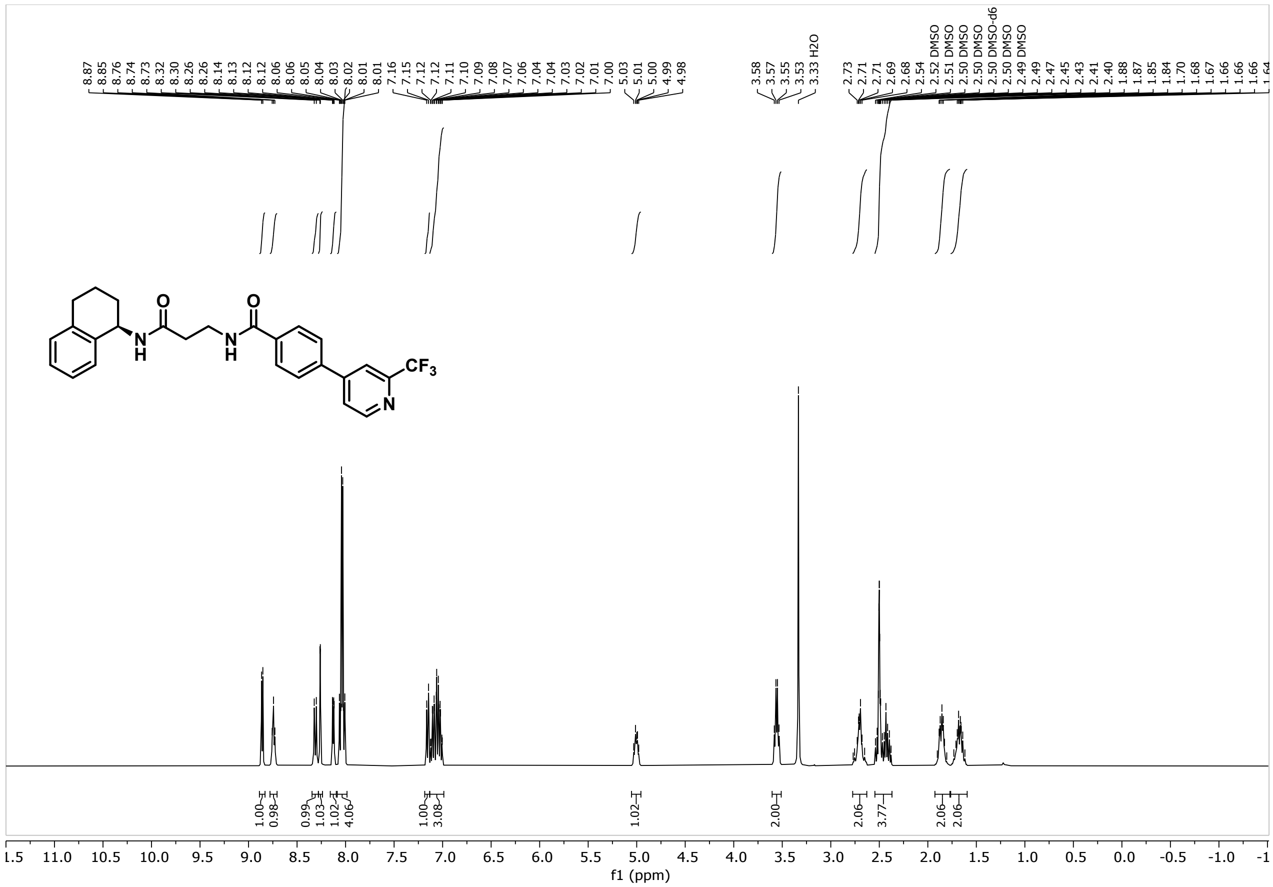

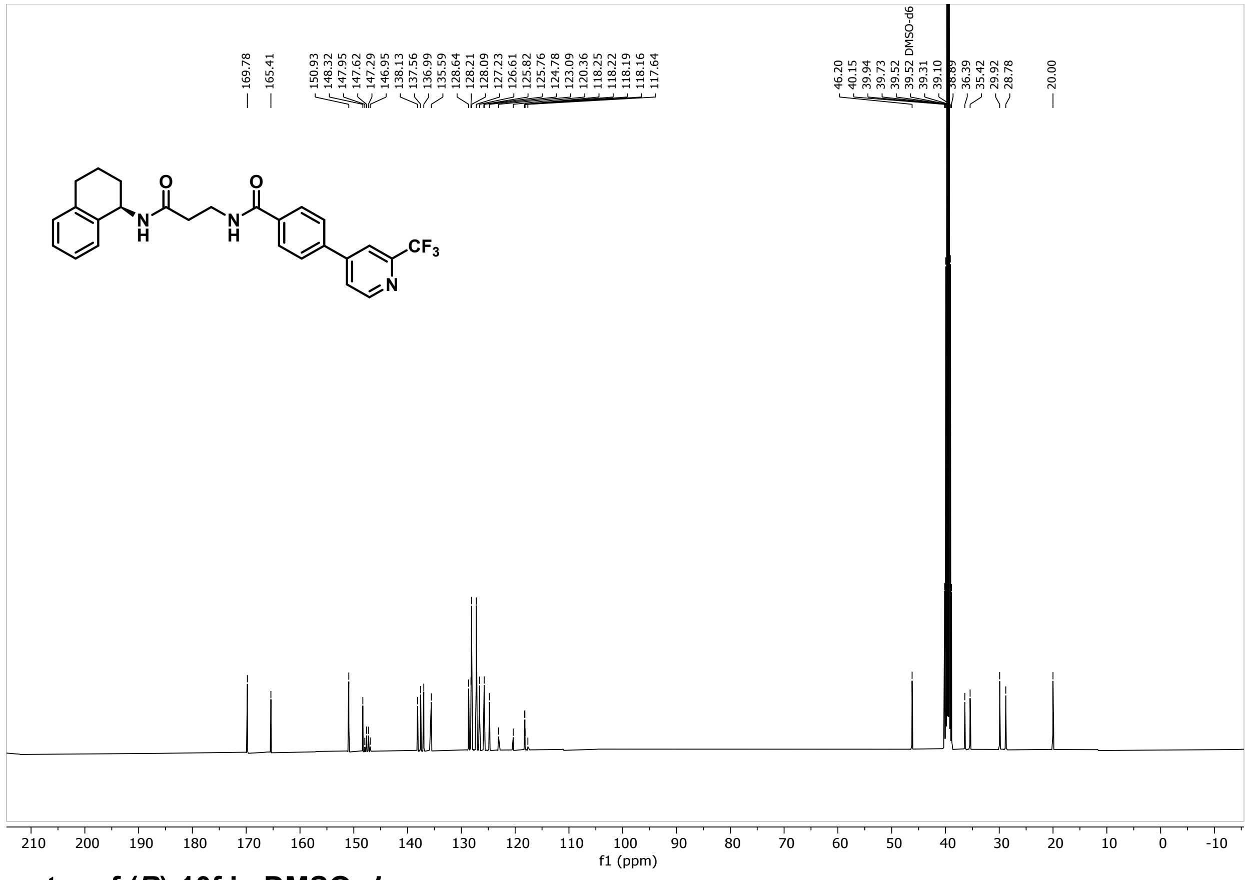

NMR spectra of (*R*)-10f in DMSO-*d*$_6$.

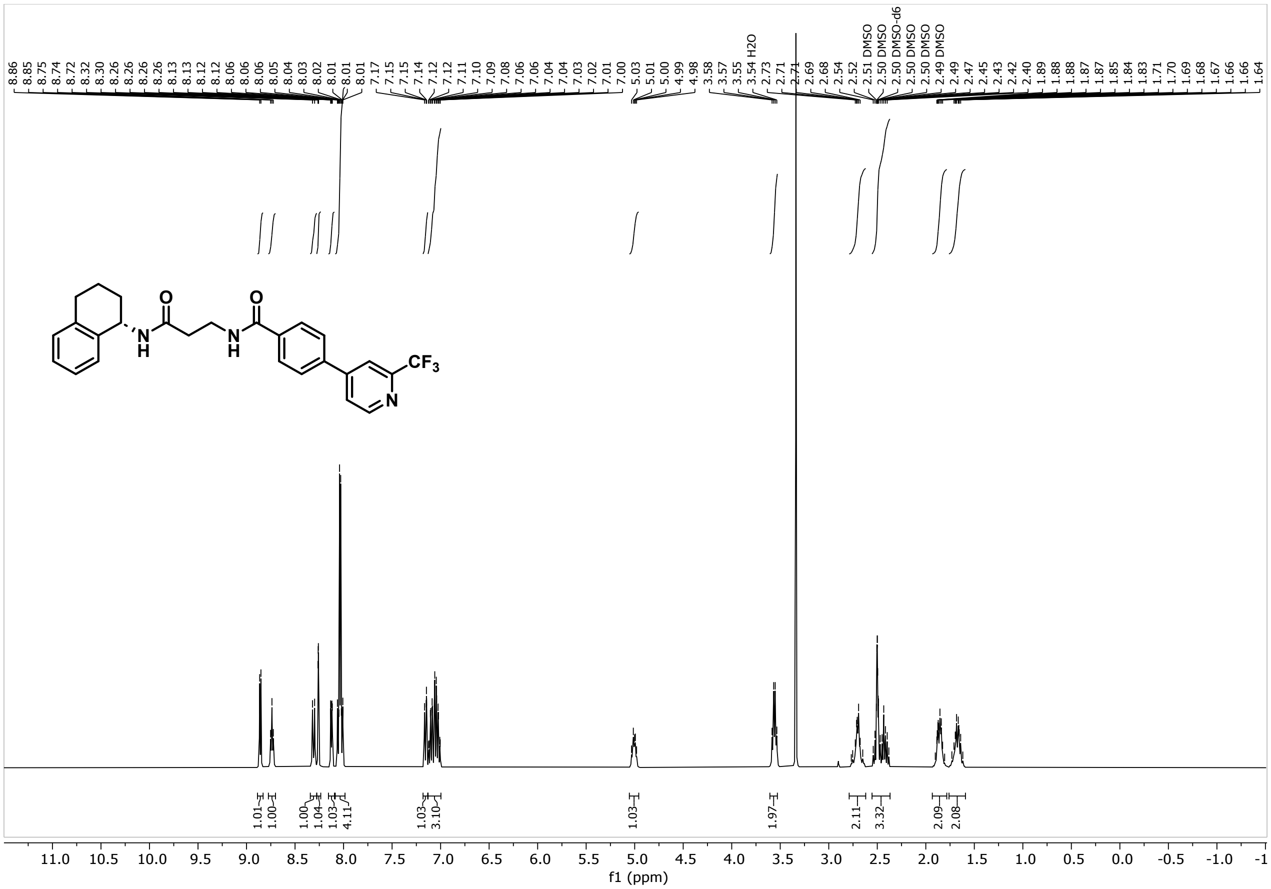

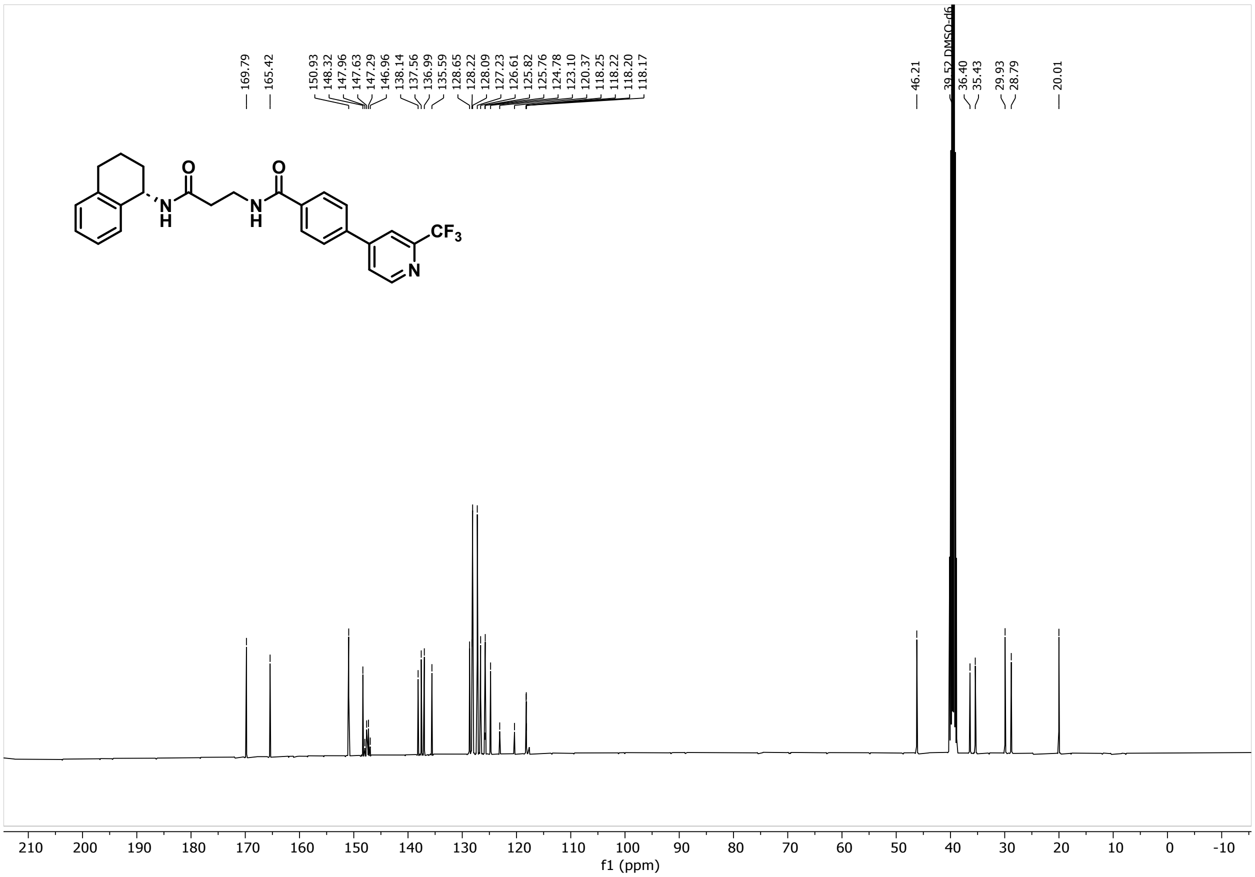

NMR spectra of (*S*)-10f in DMSO-*d*$_6$.

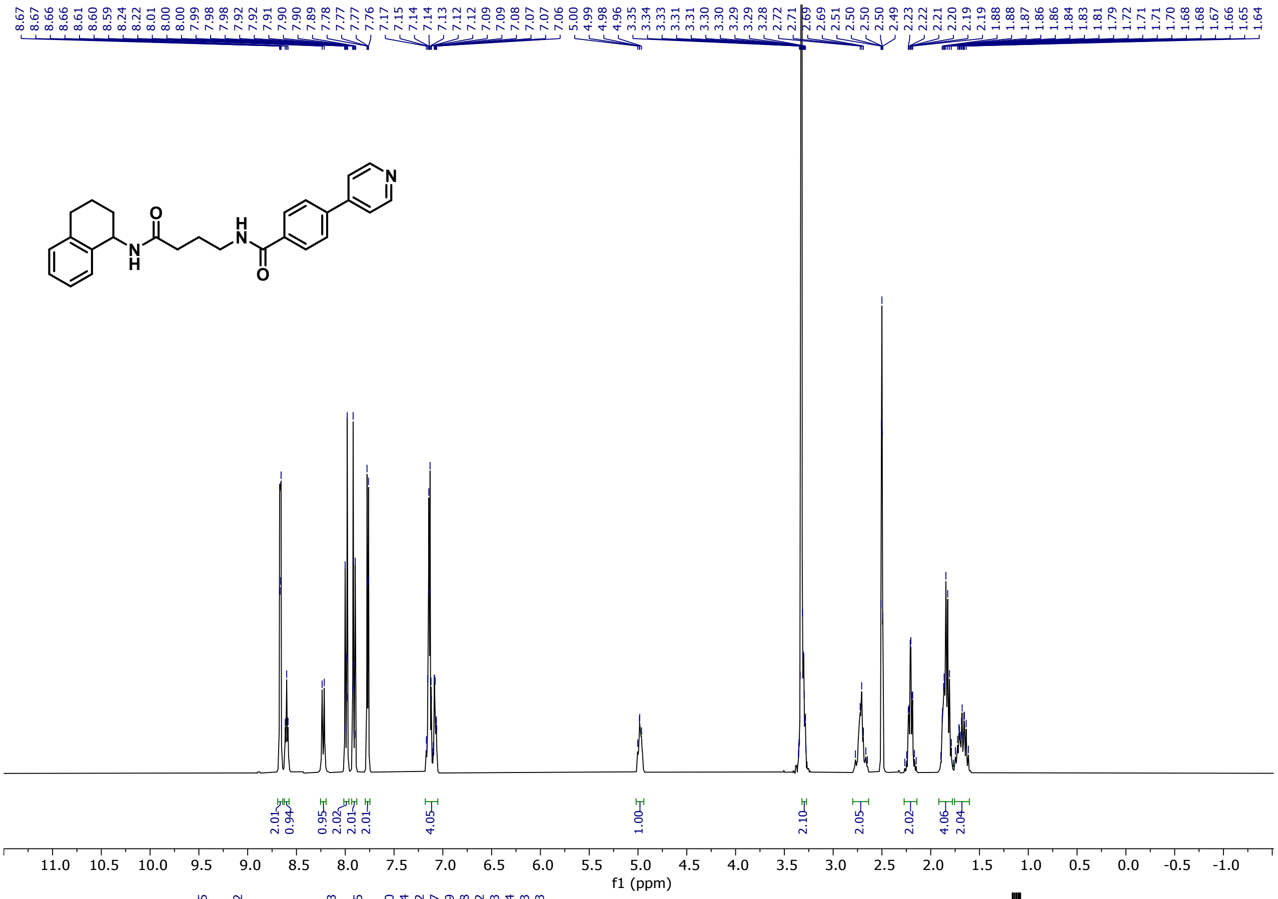

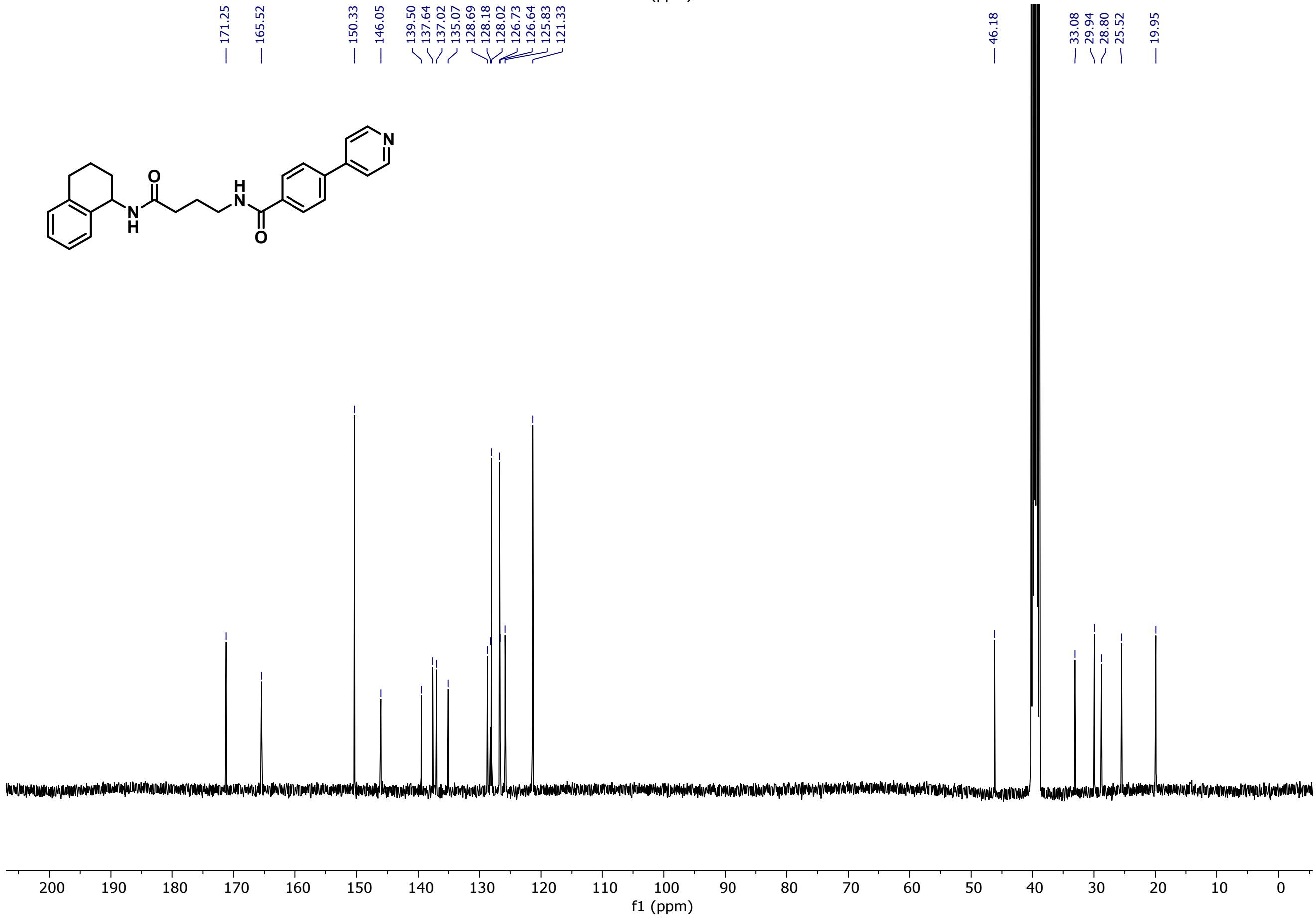

NMR spectra of 11a in DMSO-d_6.

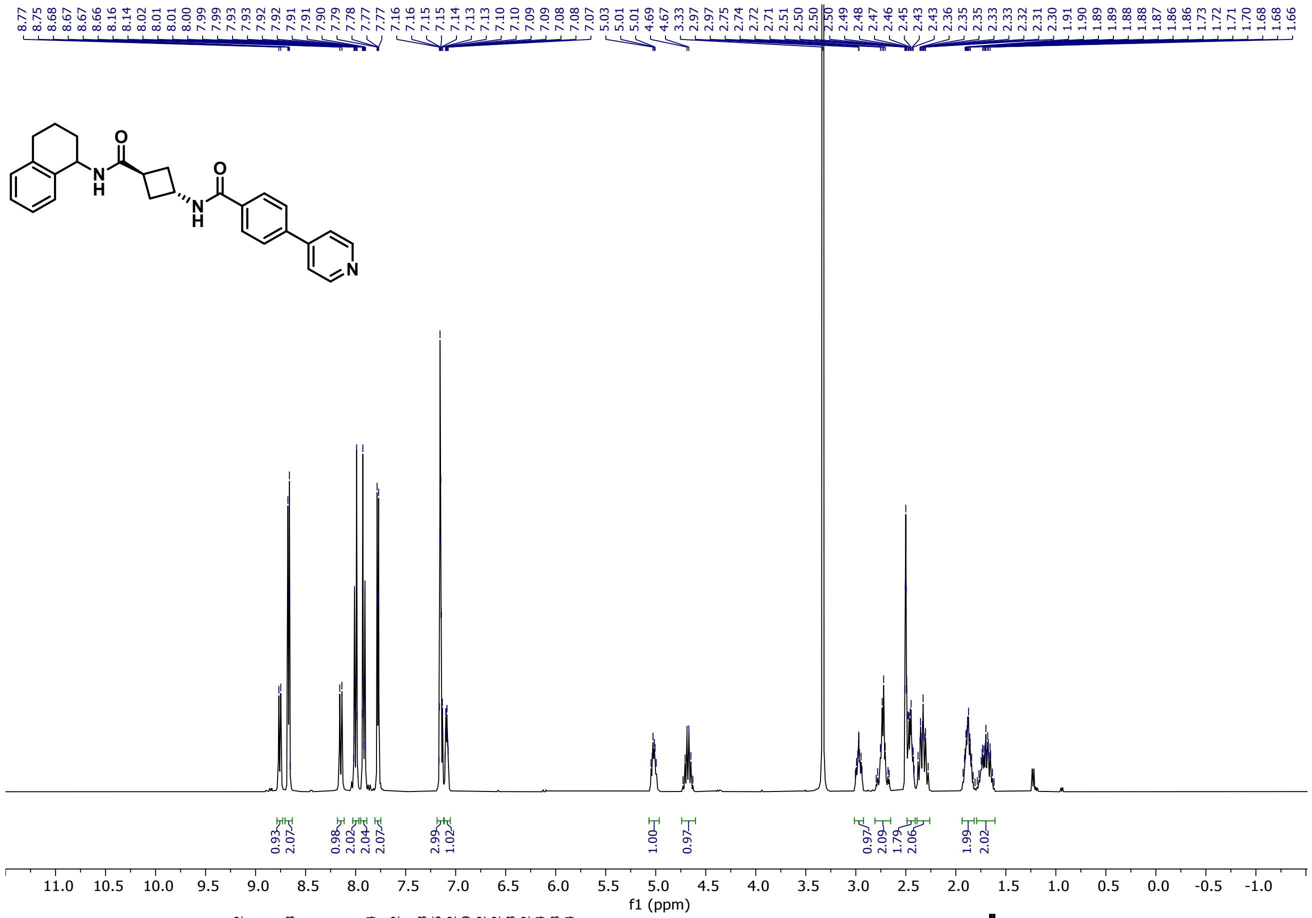

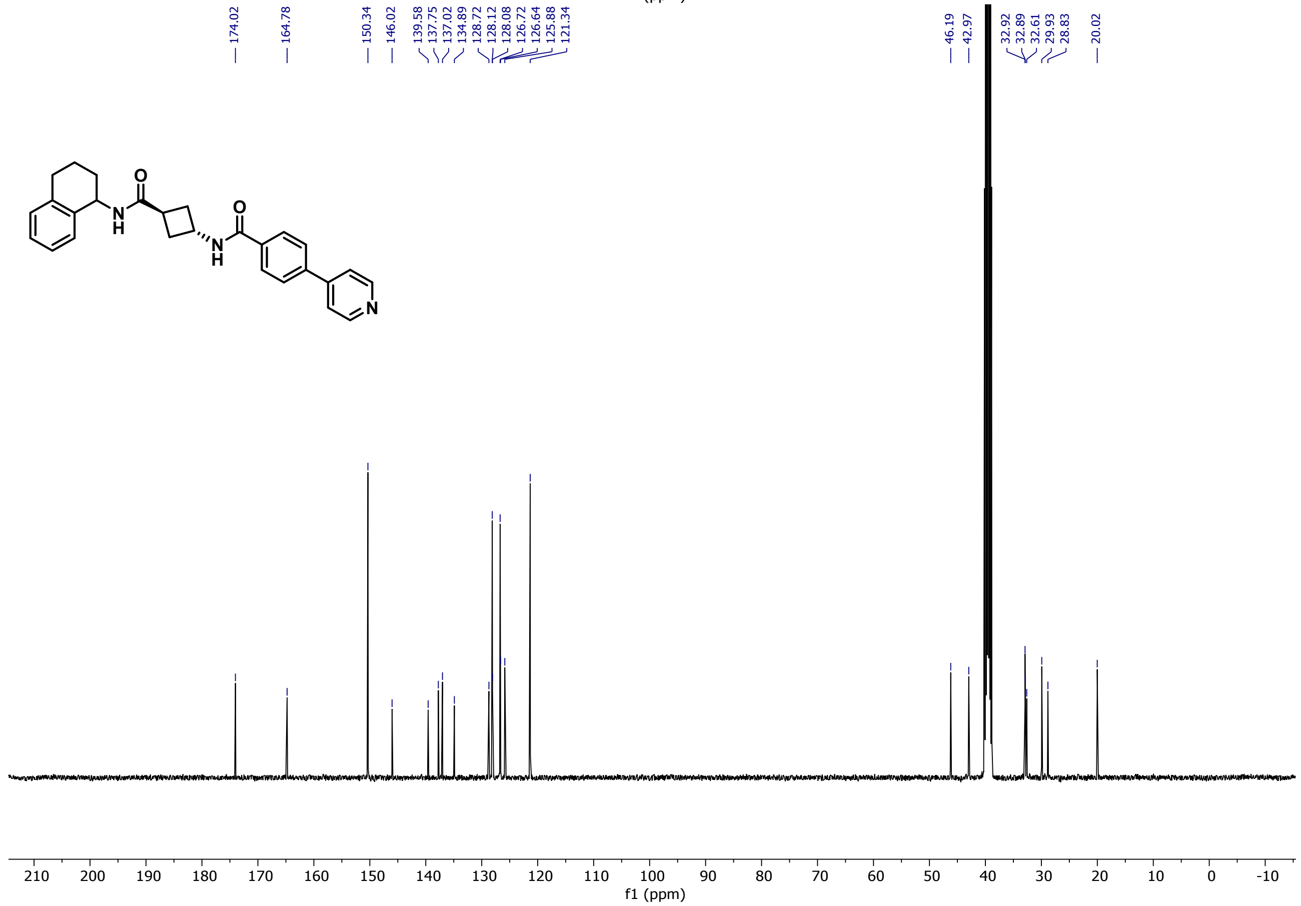

NMR spectra of 11b in DMSO-d_6.

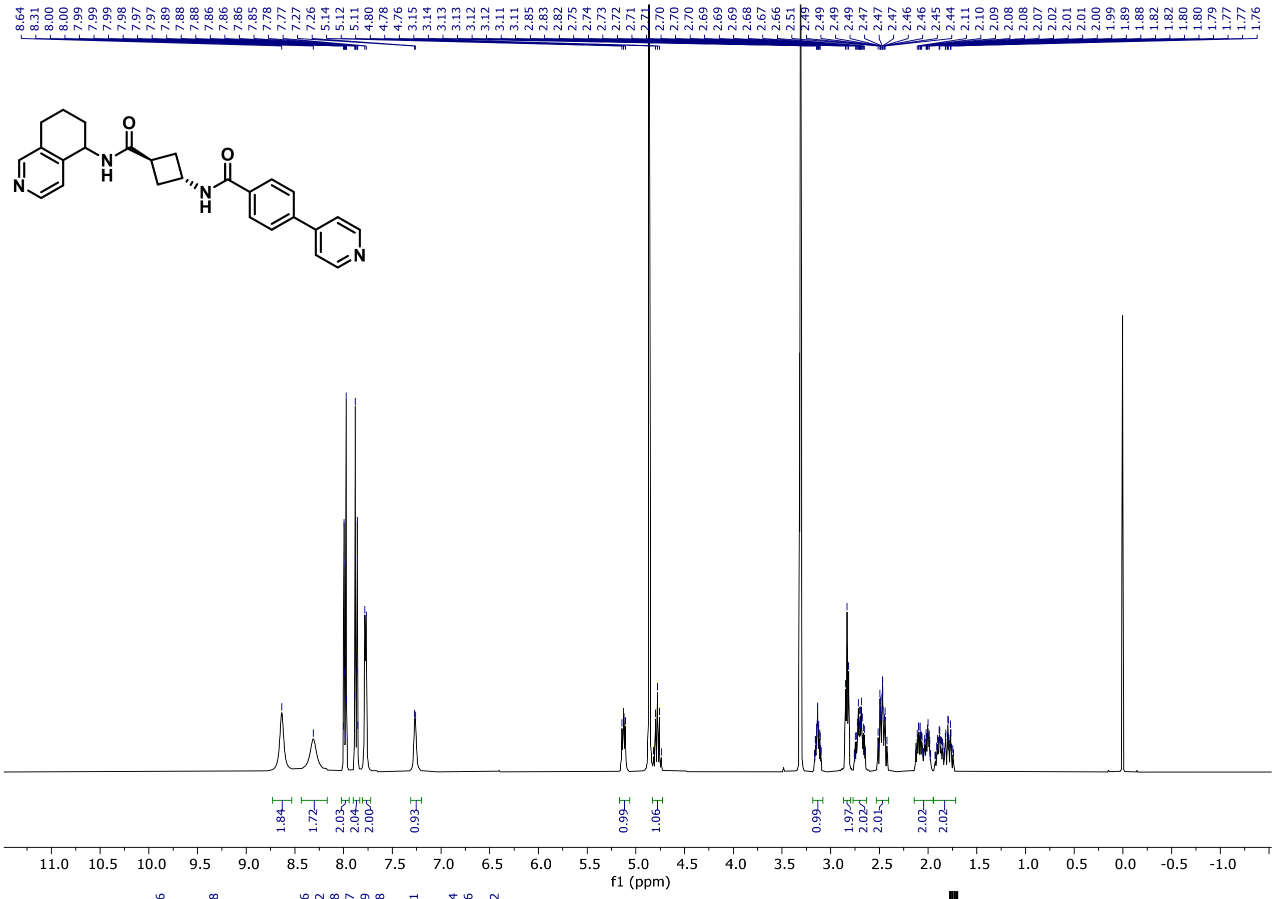

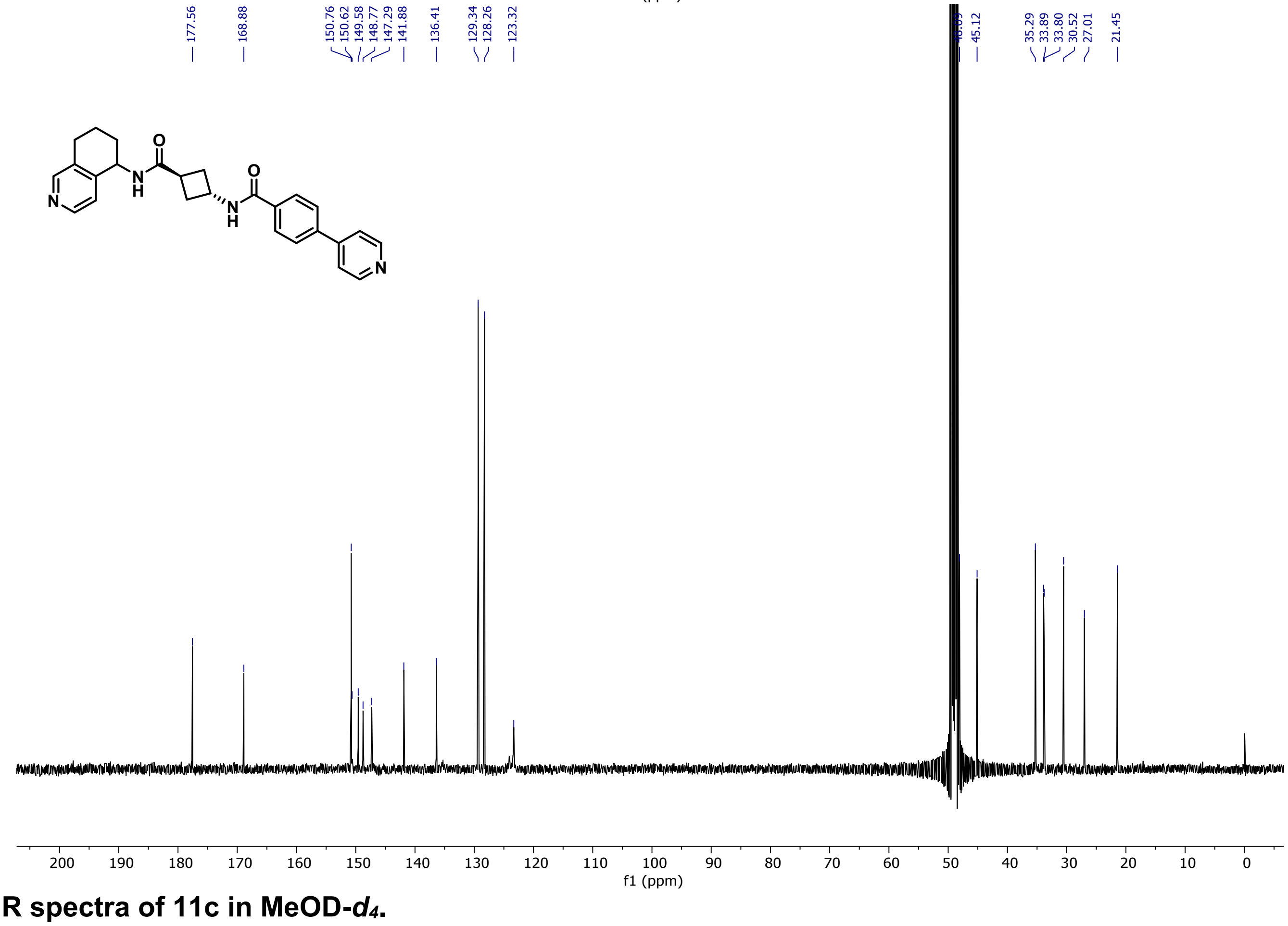

NMR spectra of 11c in MeOD-d_4.

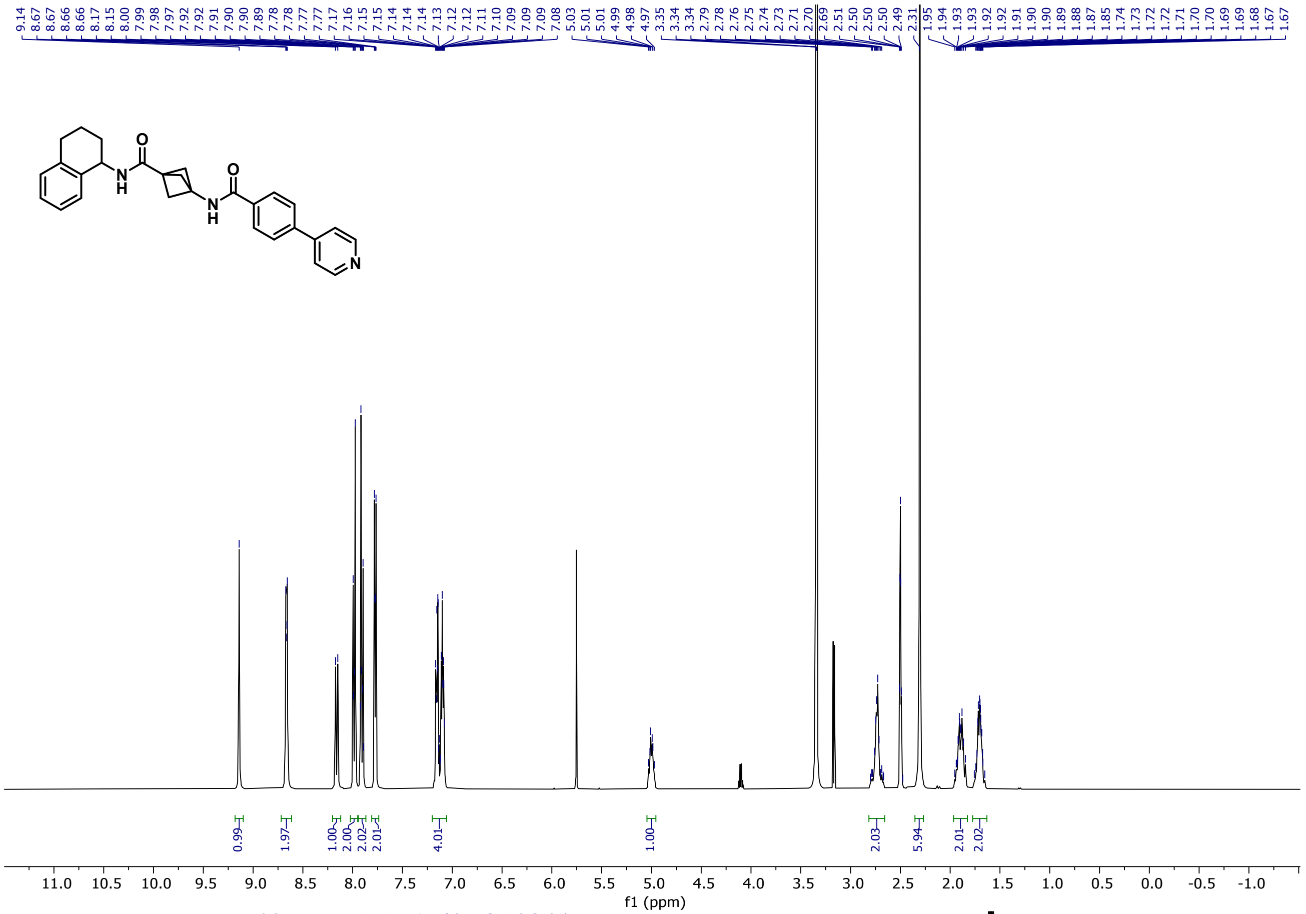

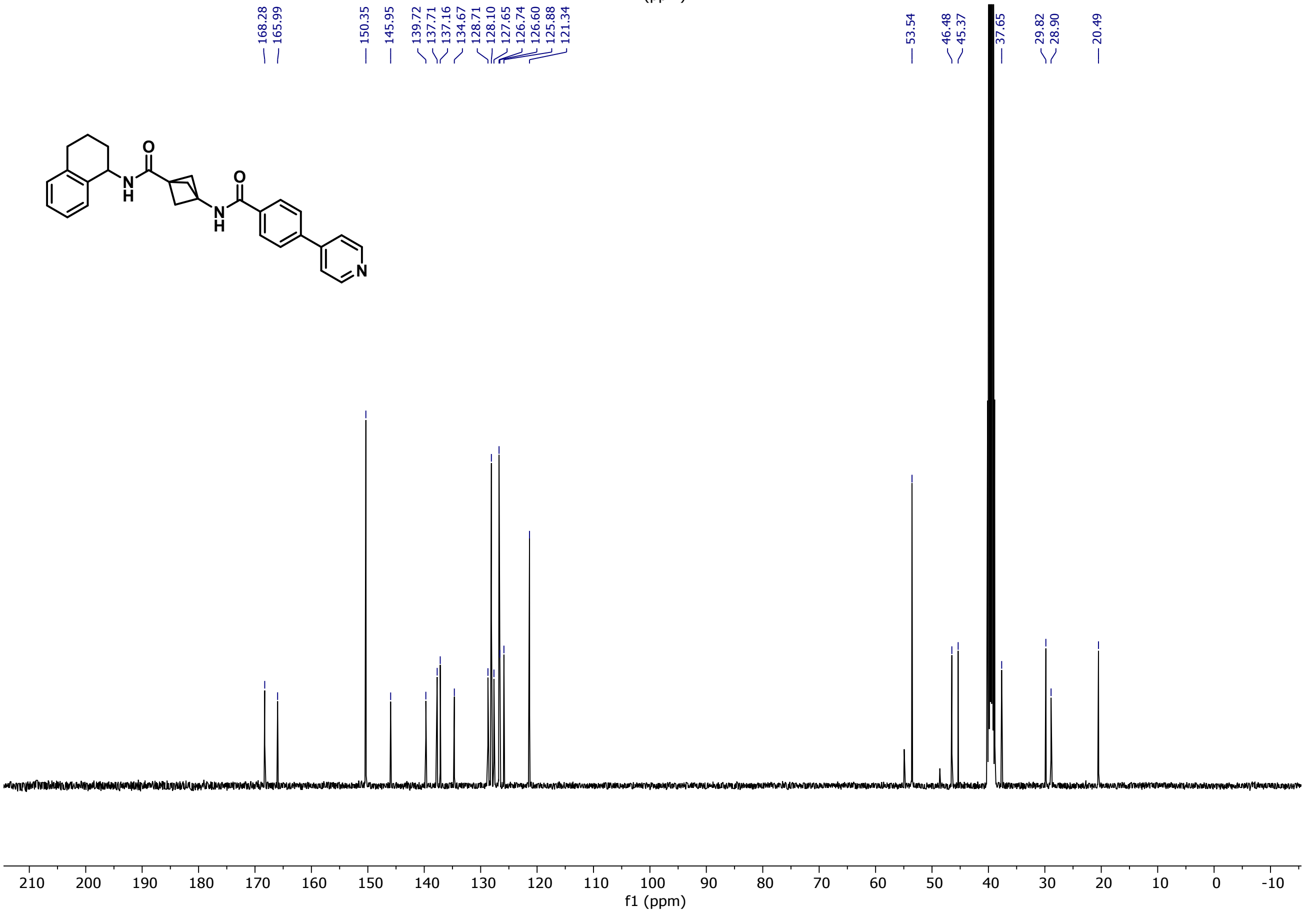

NMR spectra of 11d in DMSO-d_6.

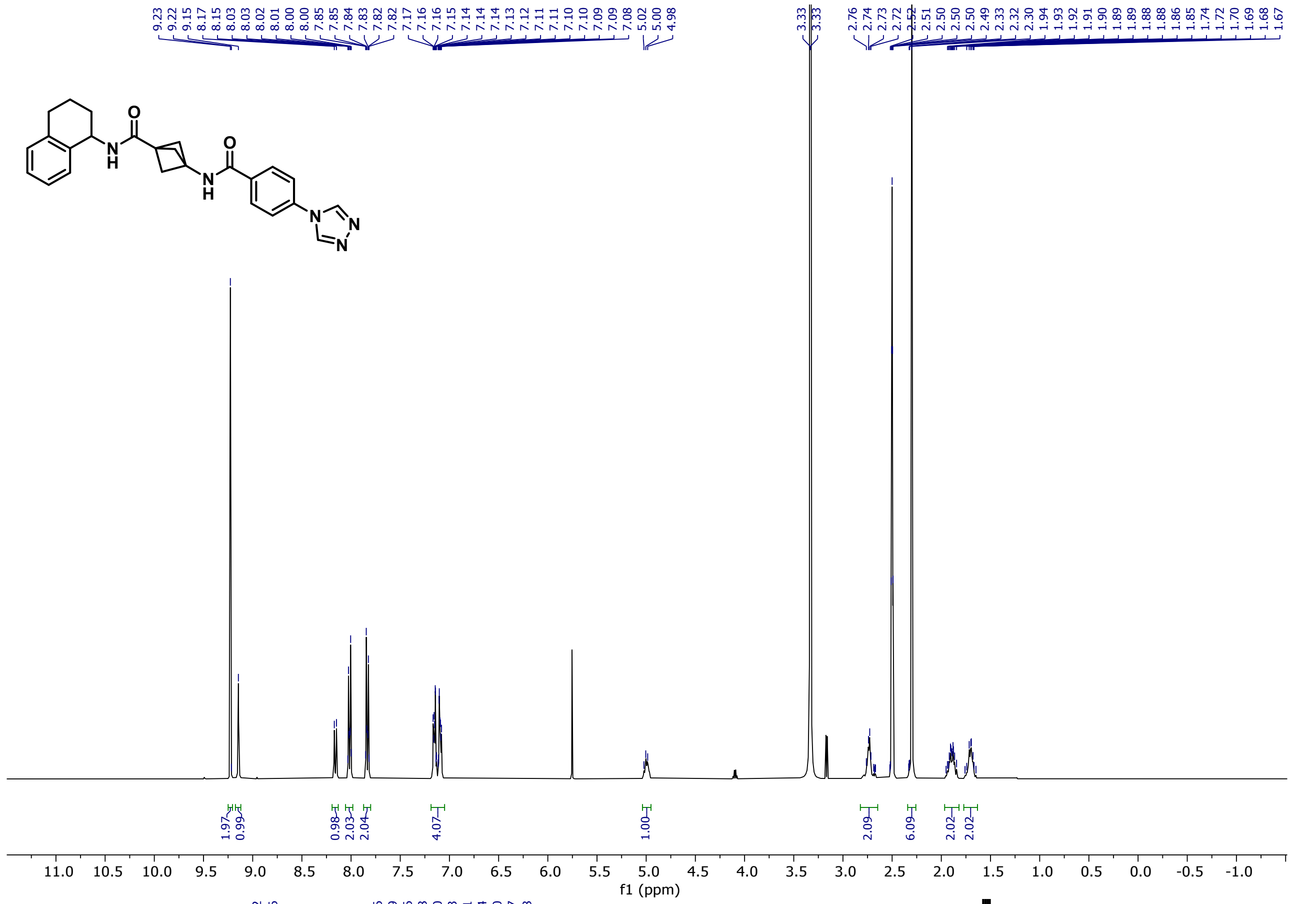

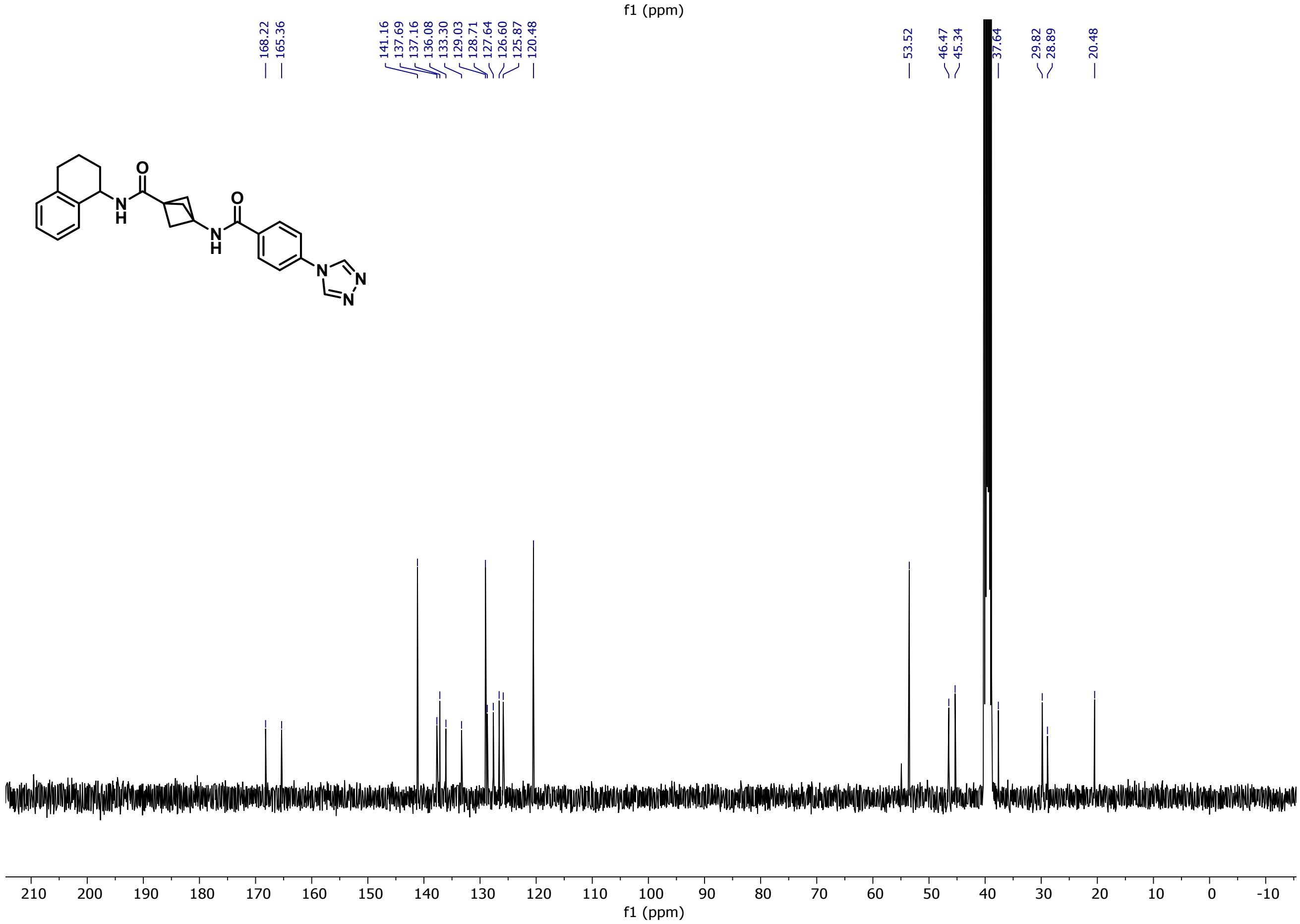

NMR spectra of 11e in DMSO-*d*$_6$.

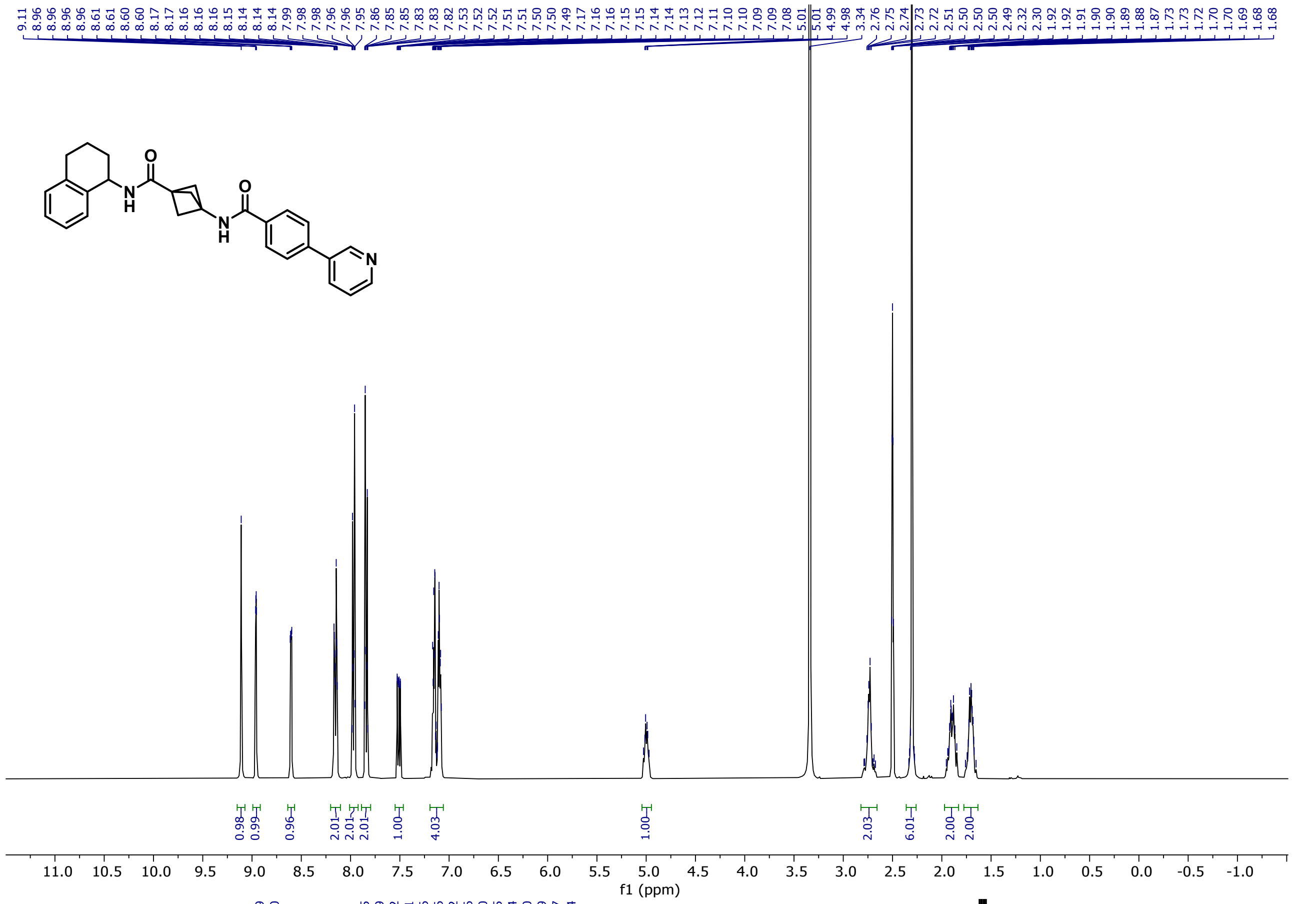

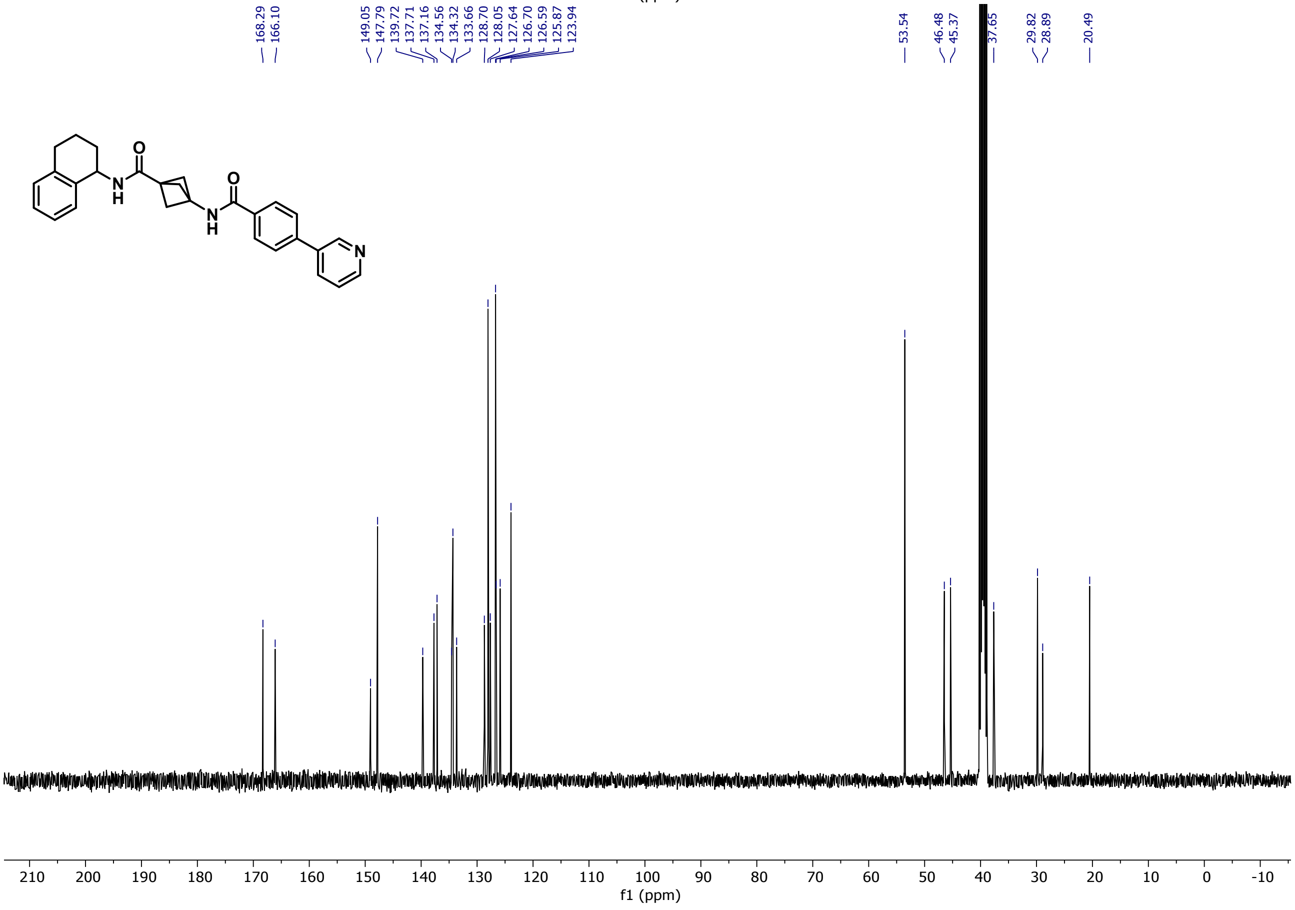

NMR spectra of 11f in DMSO-d_6.

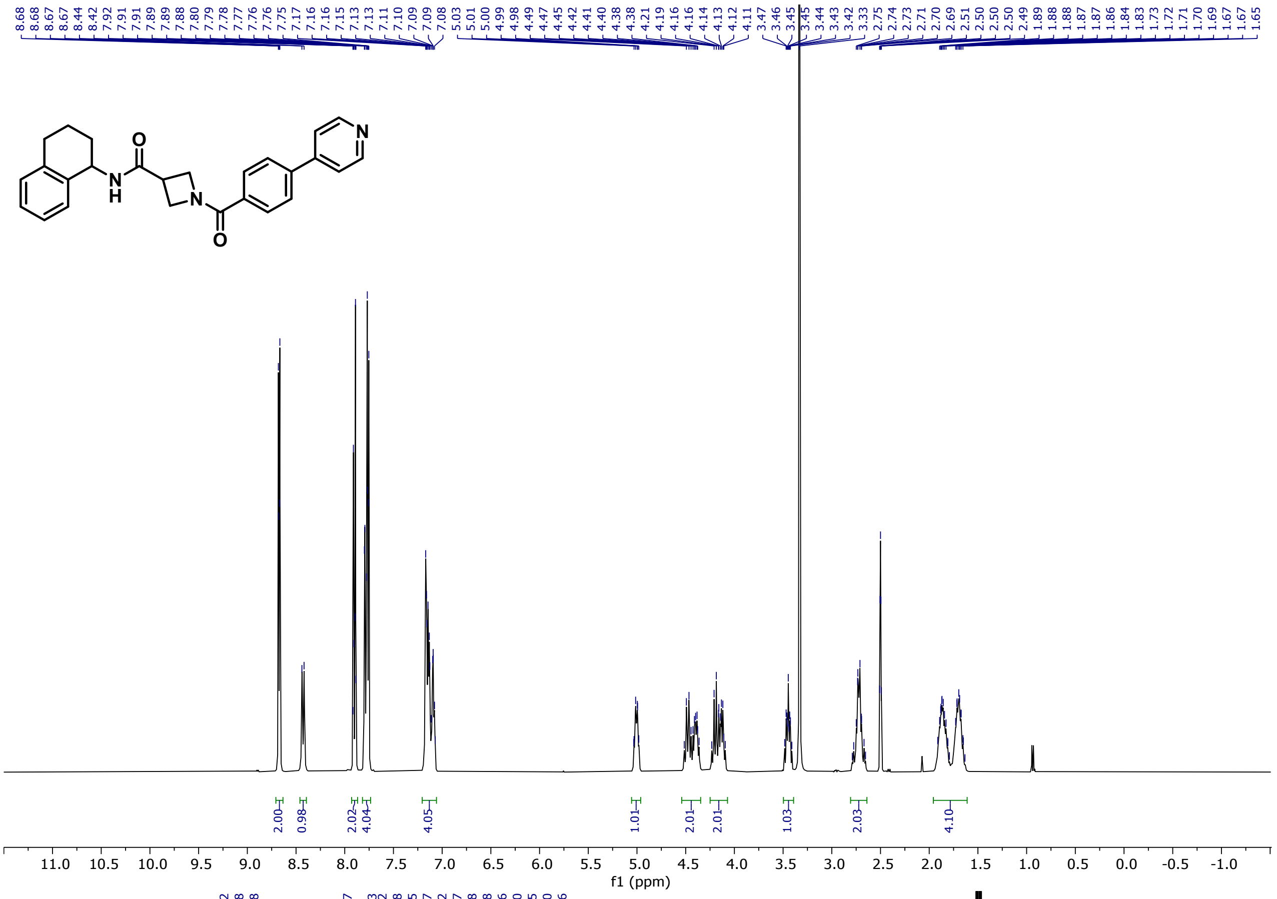

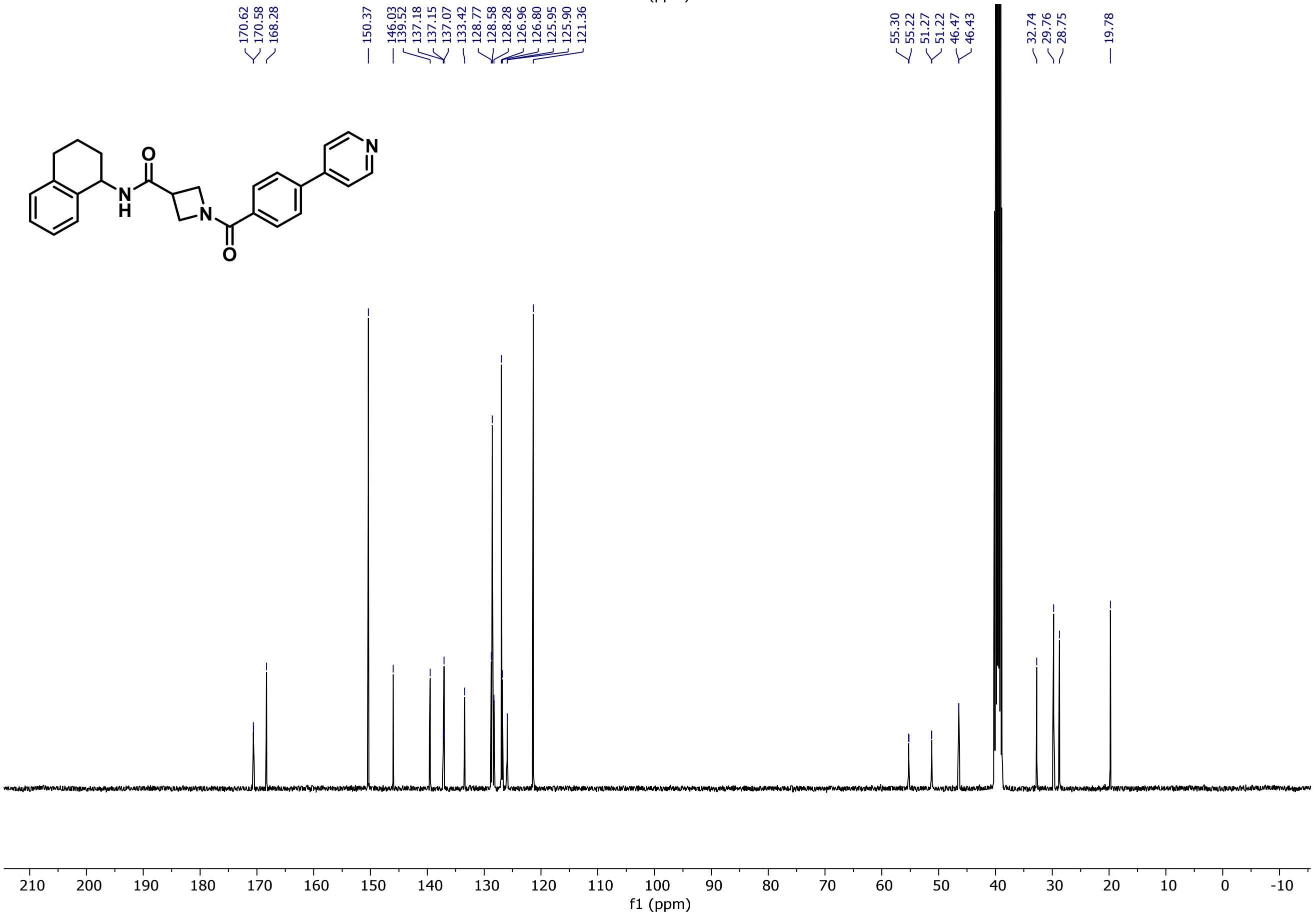

NMR spectra of 11g in DMSO-d_6.

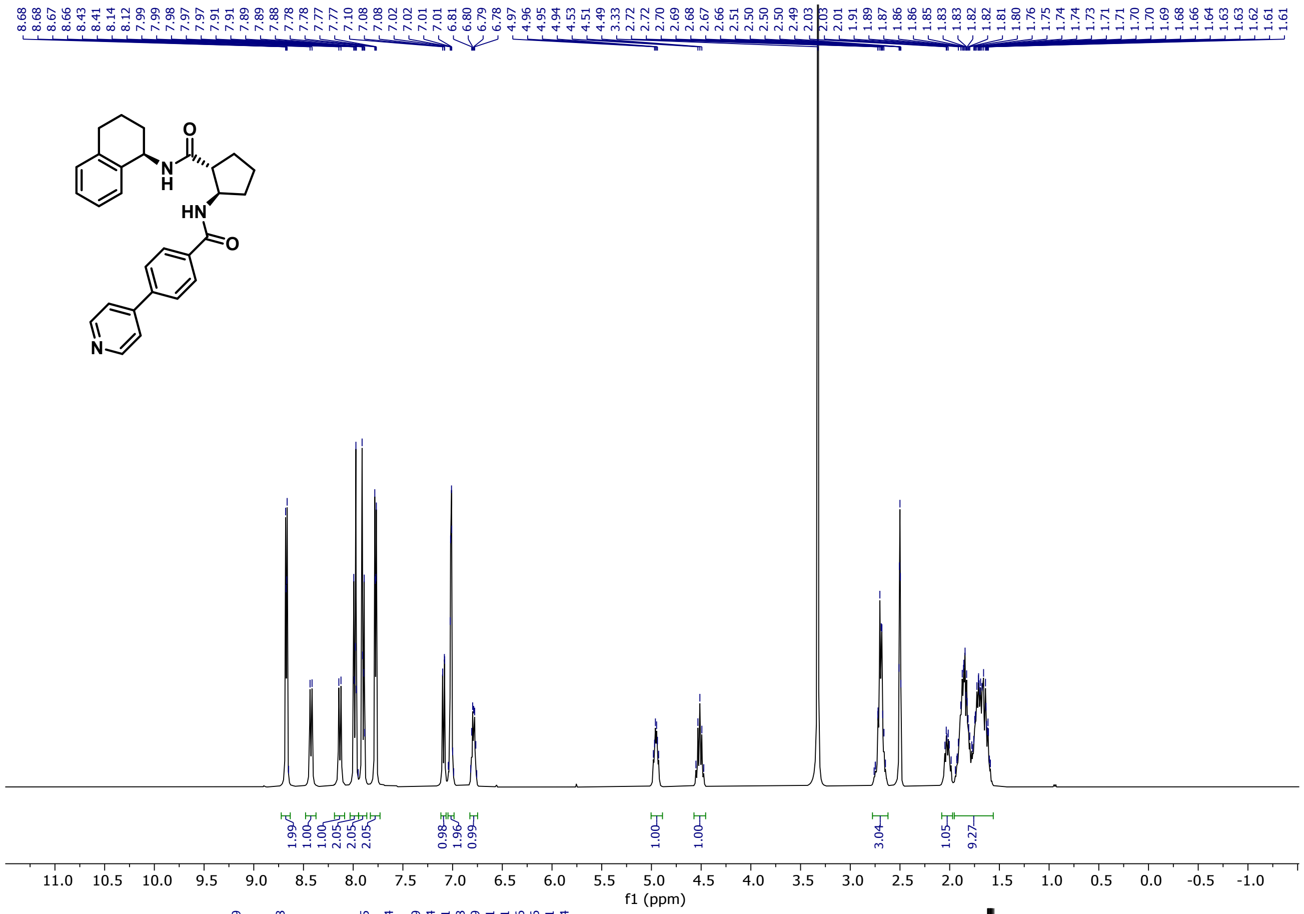

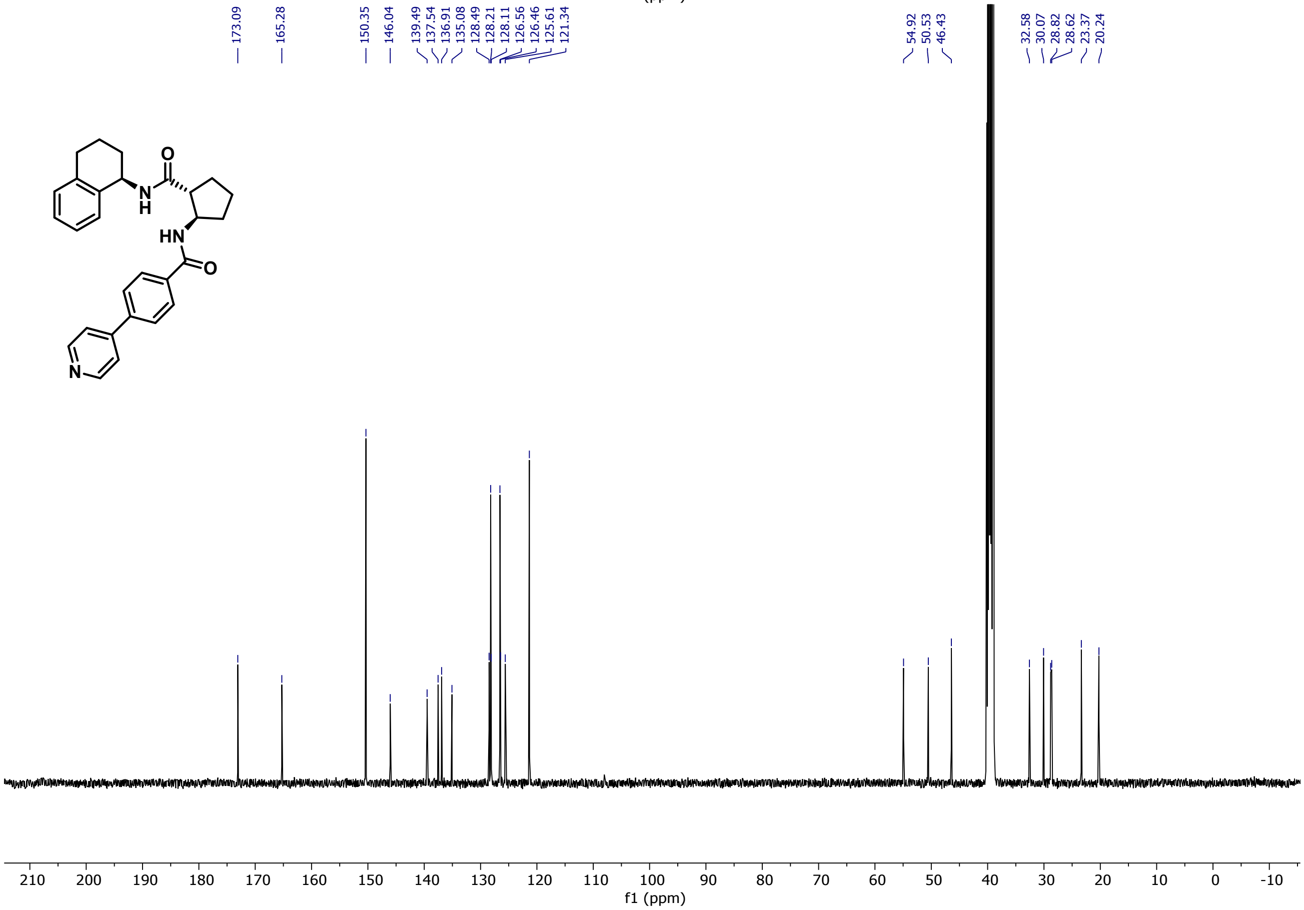

NMR spectra of 11h in DMSO-*d*$_6$.

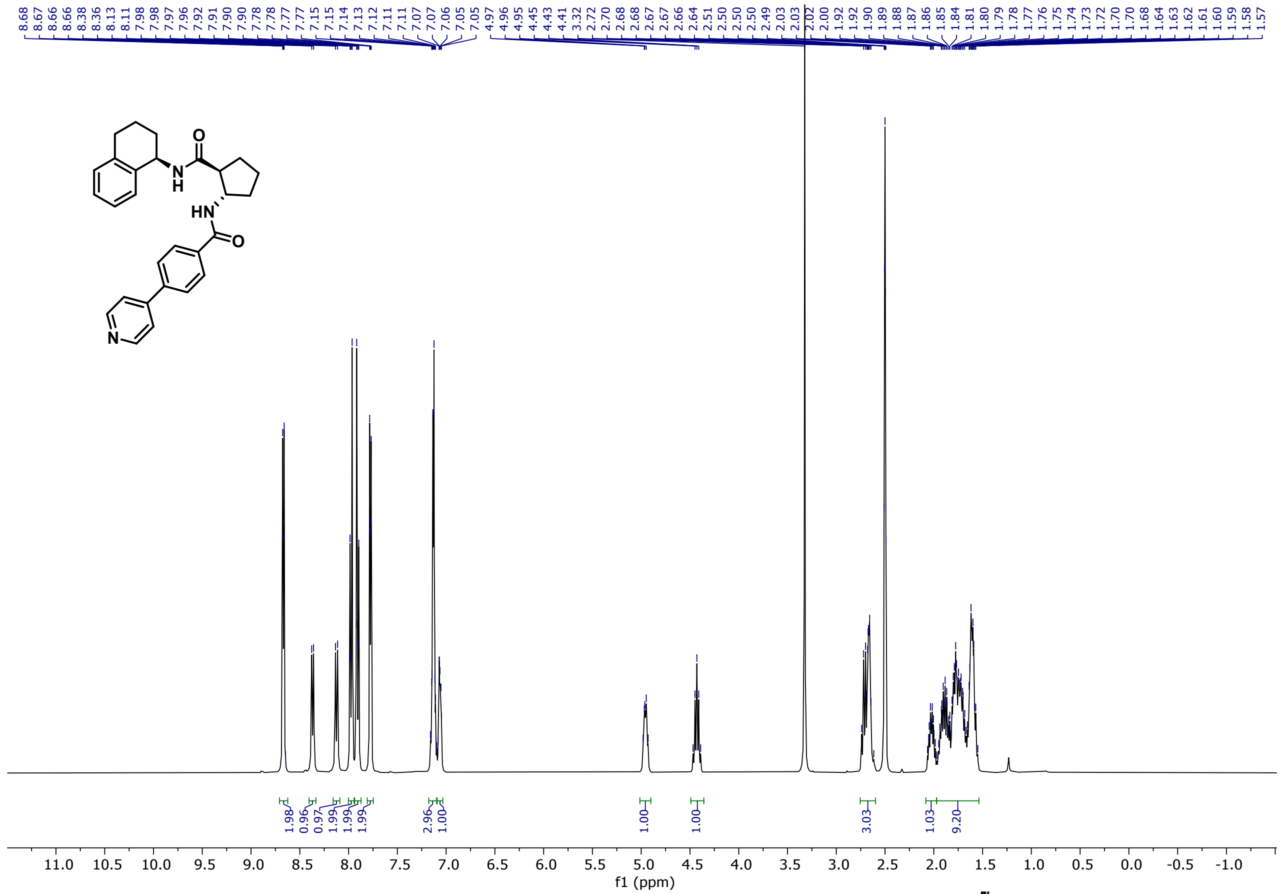

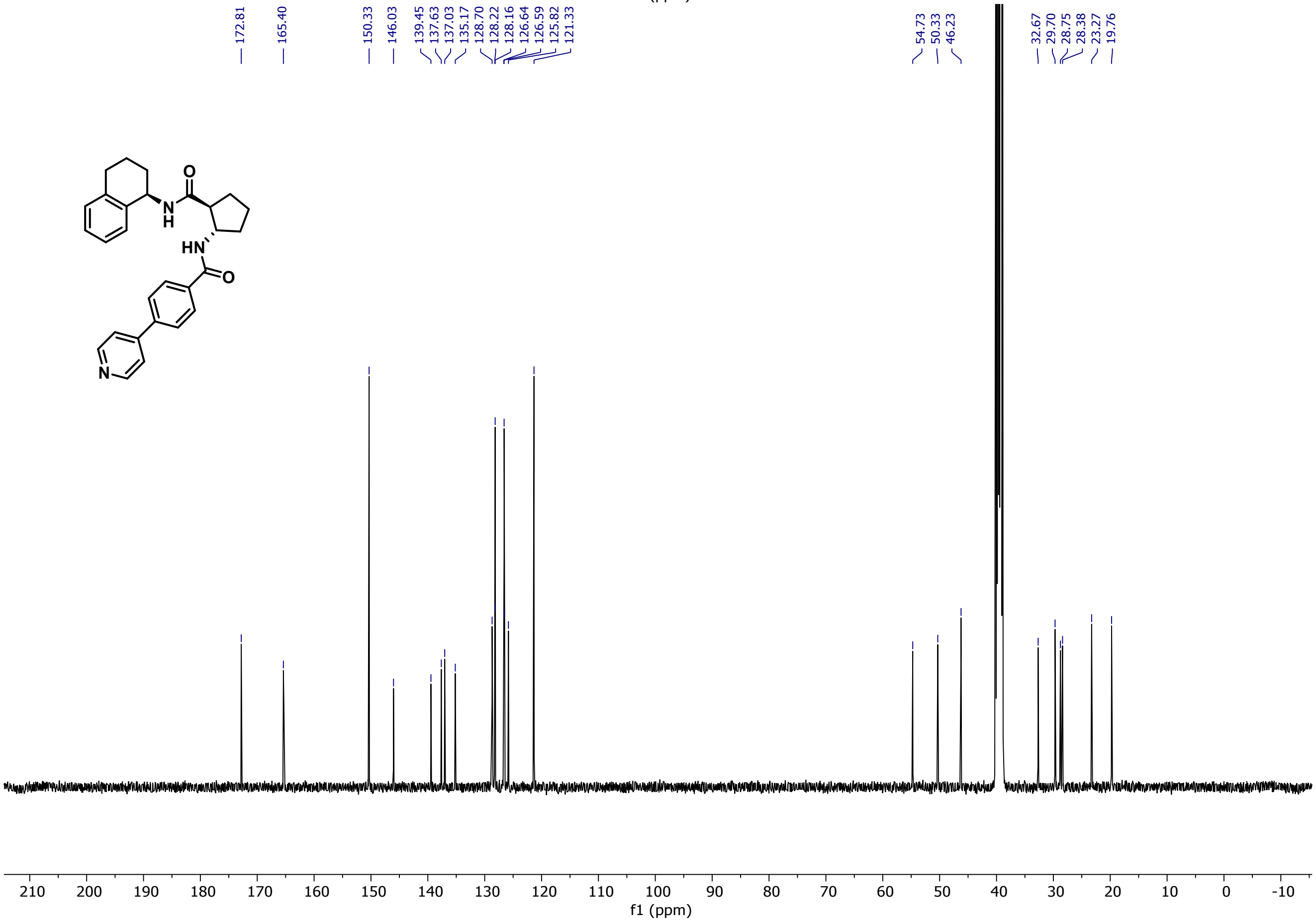

NMR spectra of 11i in DMSO-d_6.

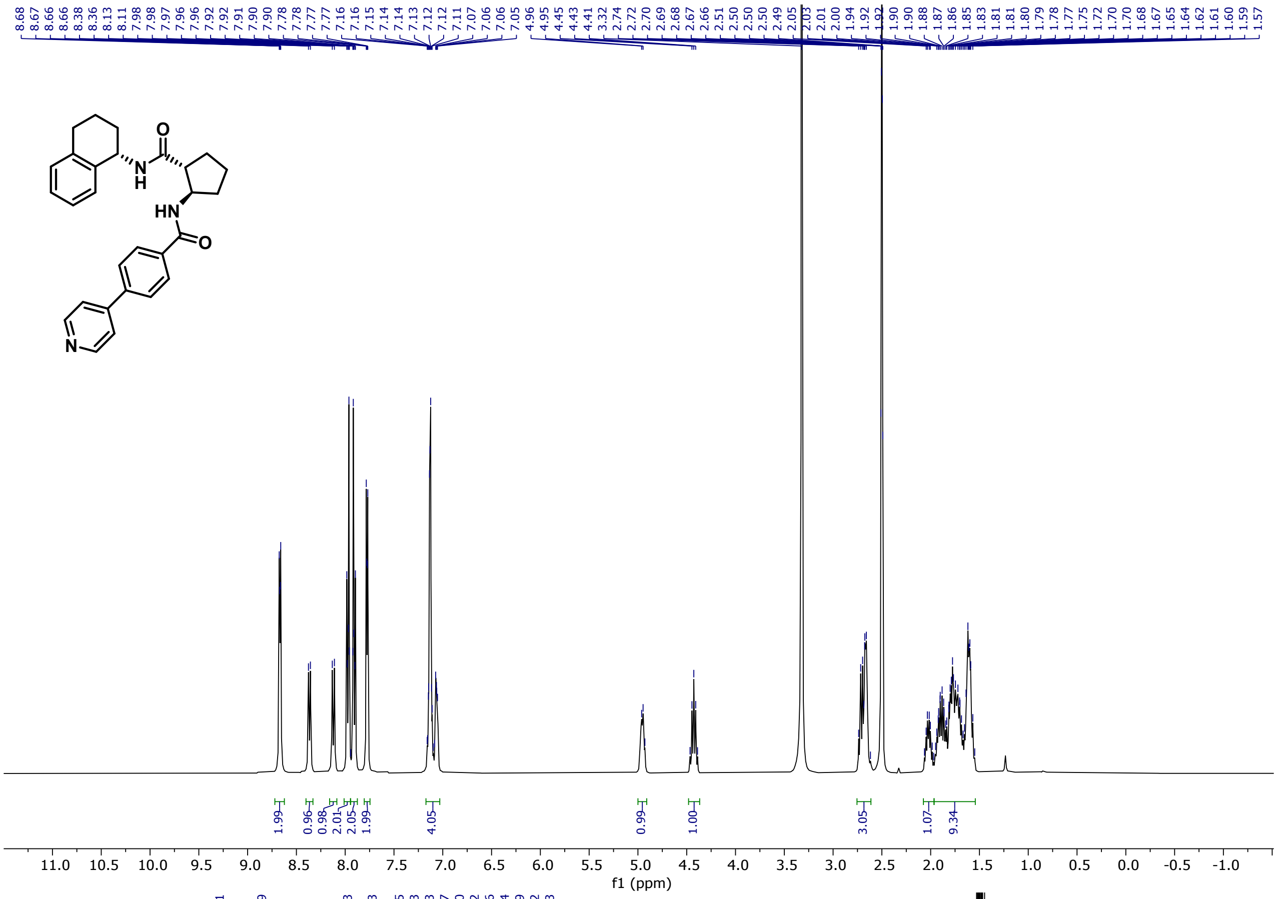

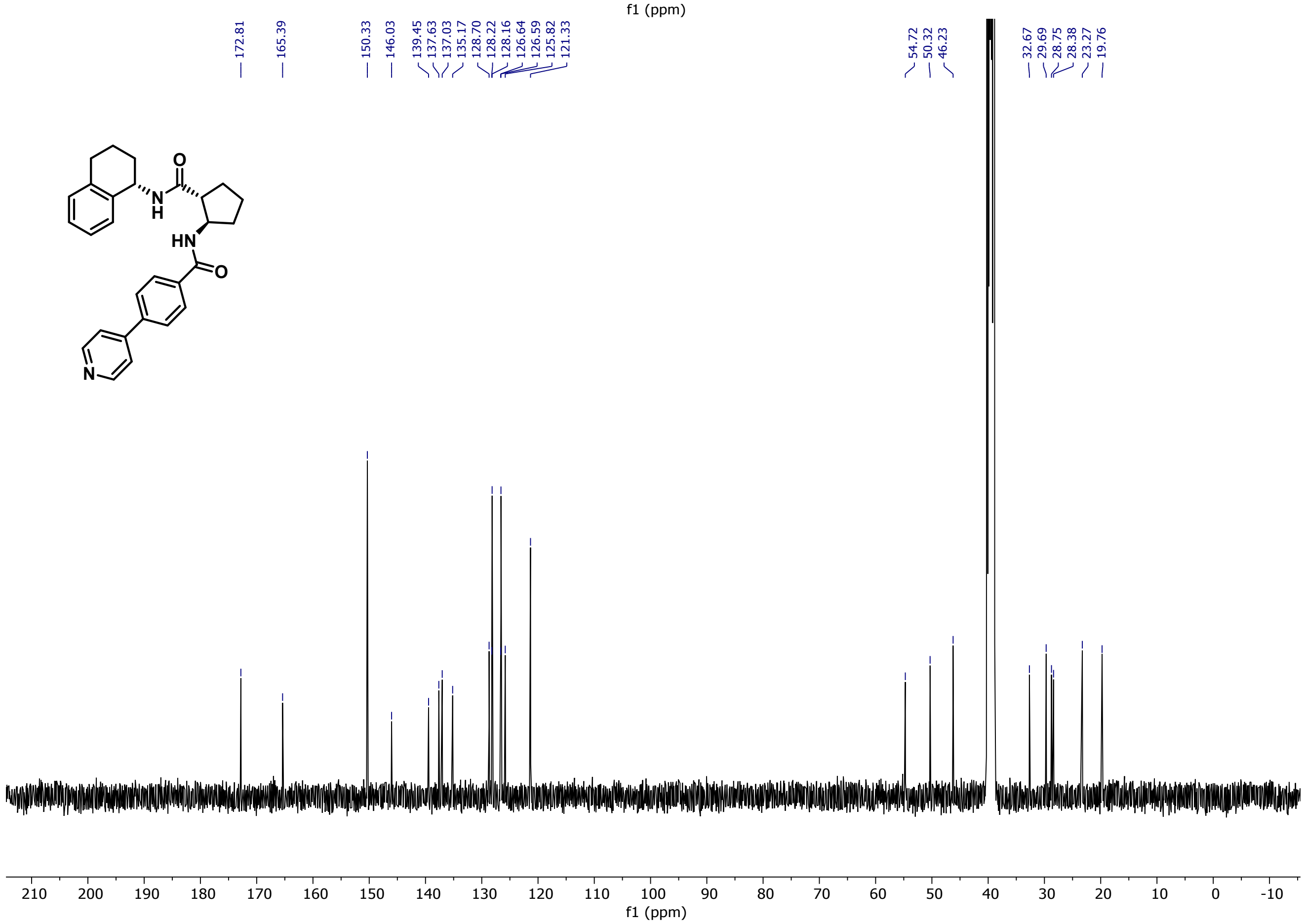

NMR spectra of 11j in DMSO-*d*$_6$.

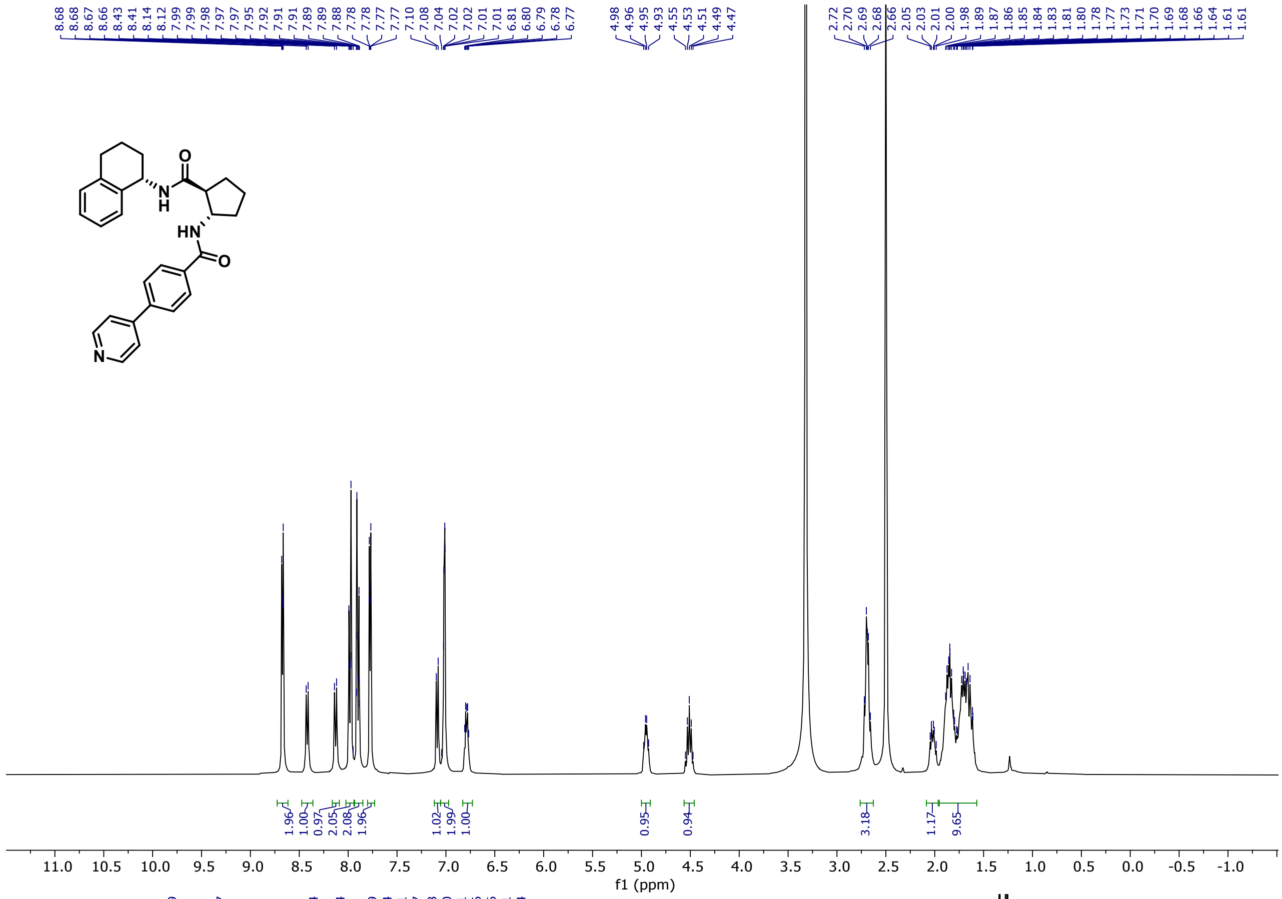

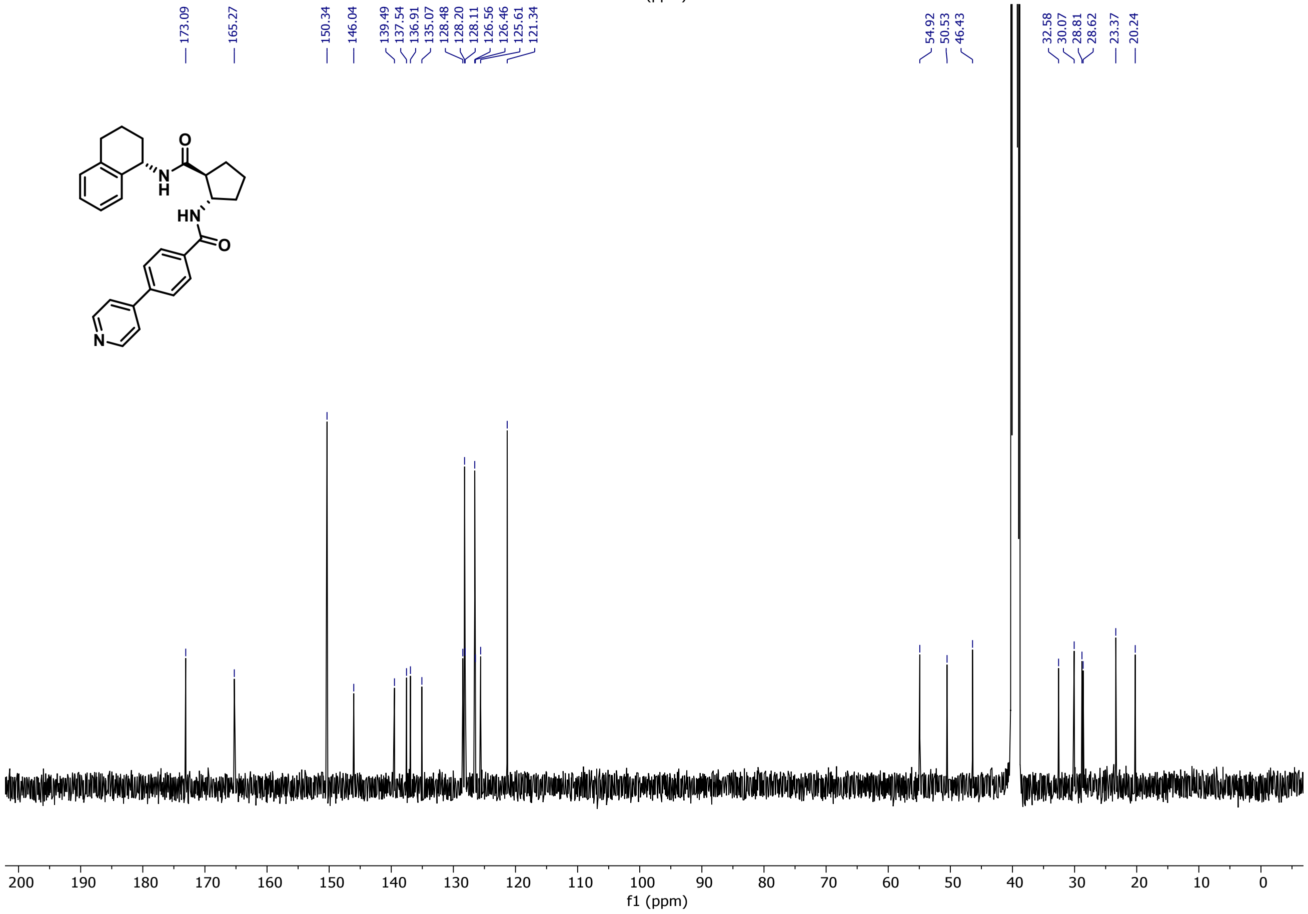

NMR spectra of 11k in DMSO-d_6.

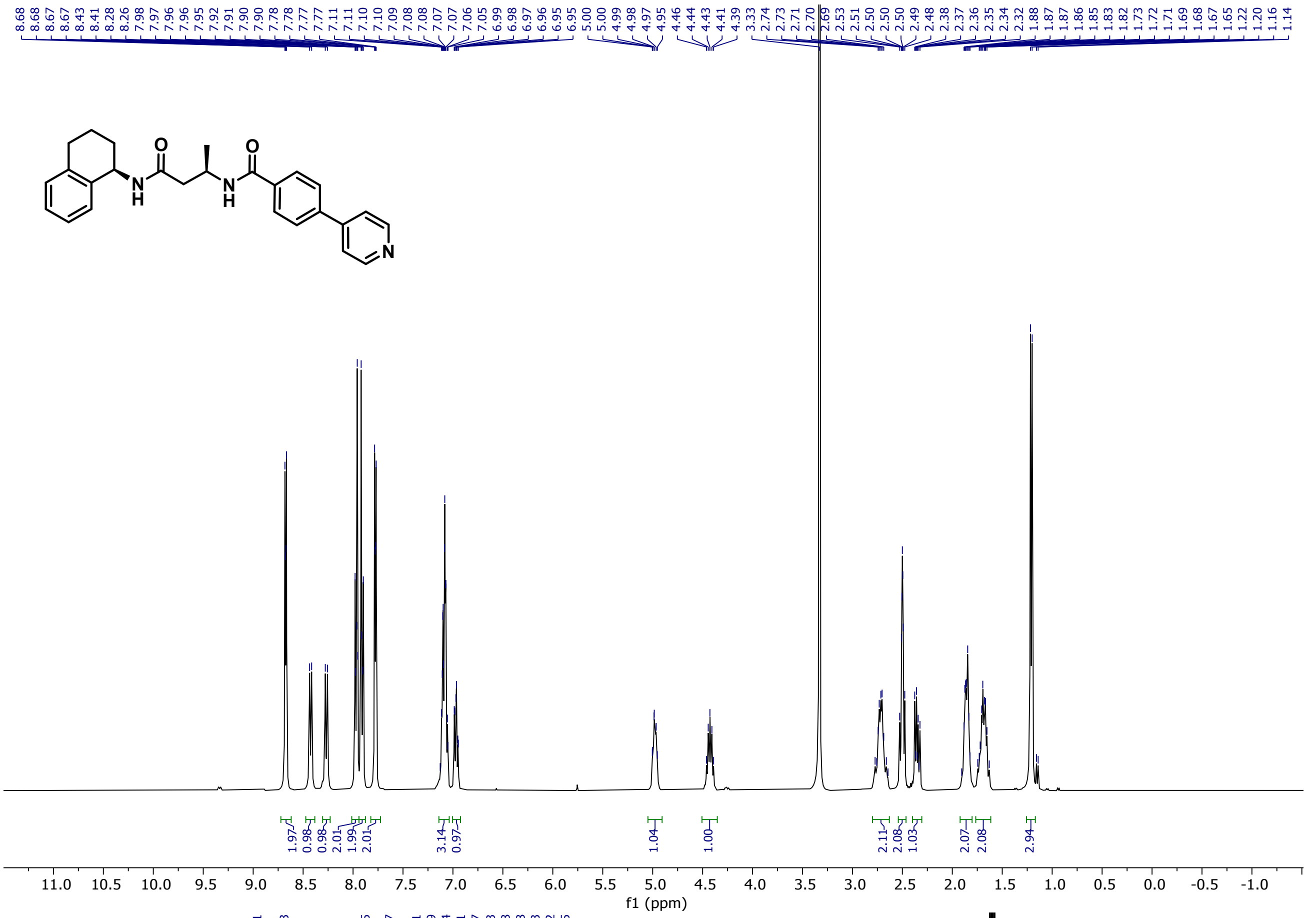

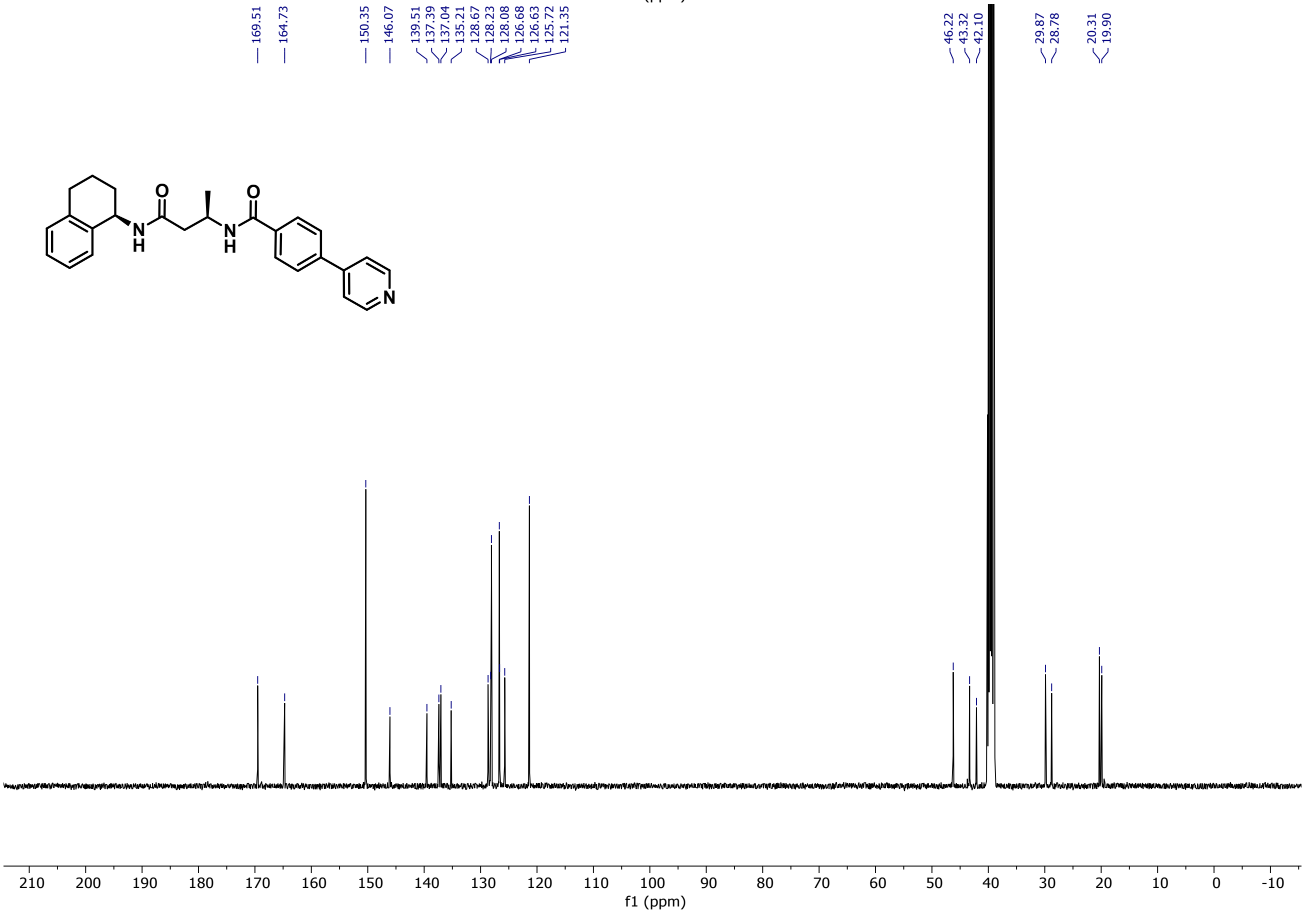

NMR spectra of 11l in DMSO-d_6.

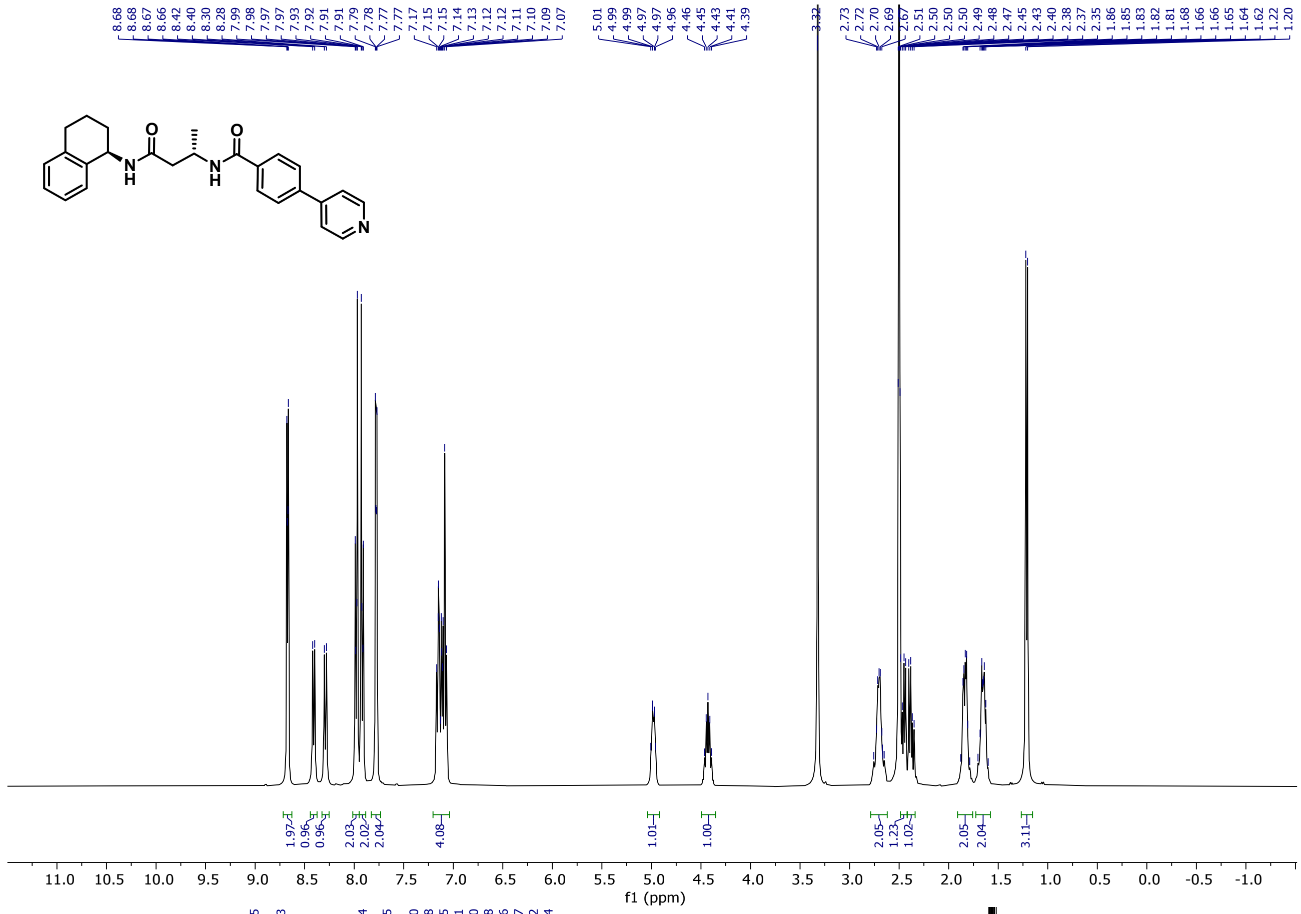

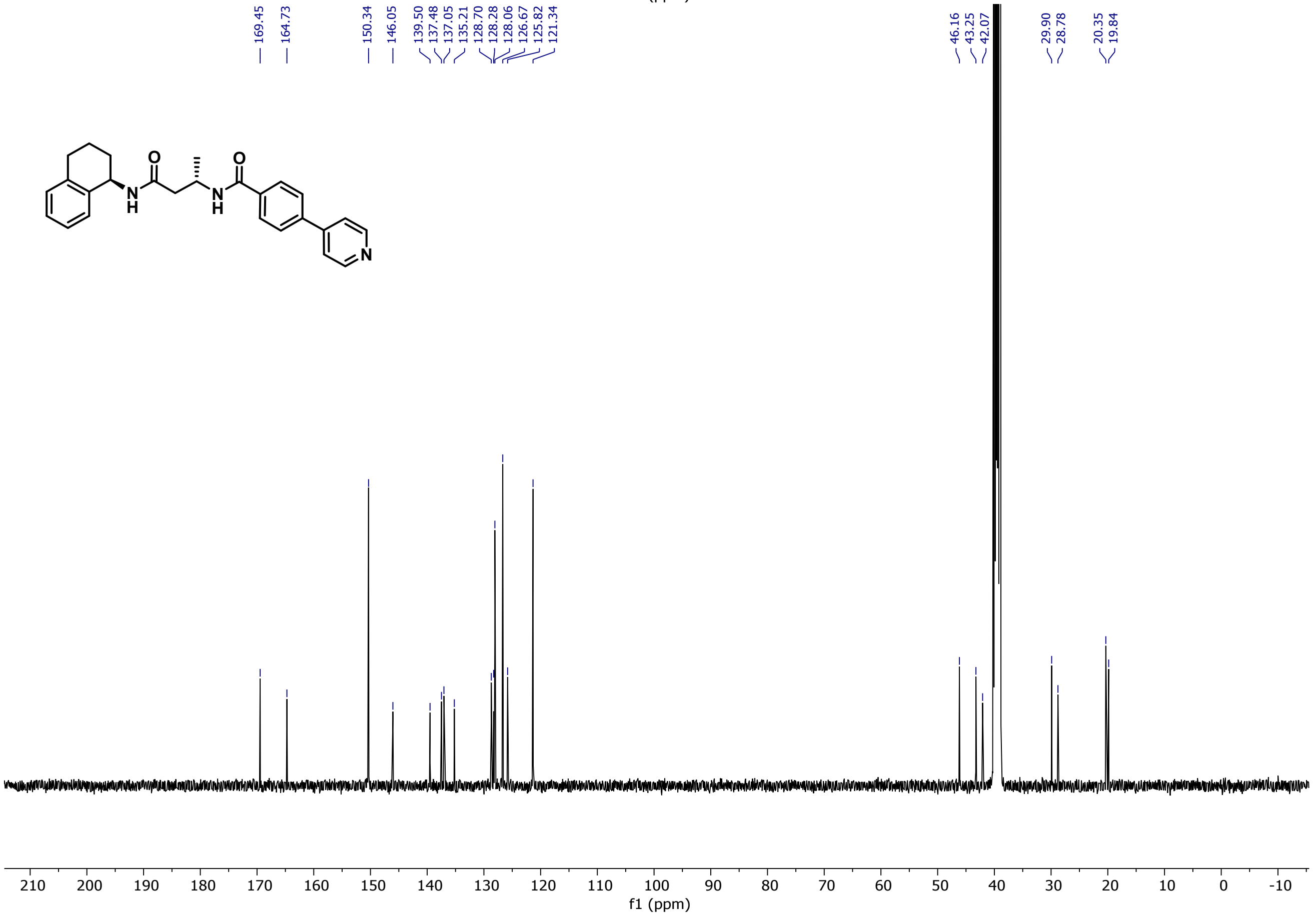

NMR spectra of 11m in DMSO-*d*$_6$.

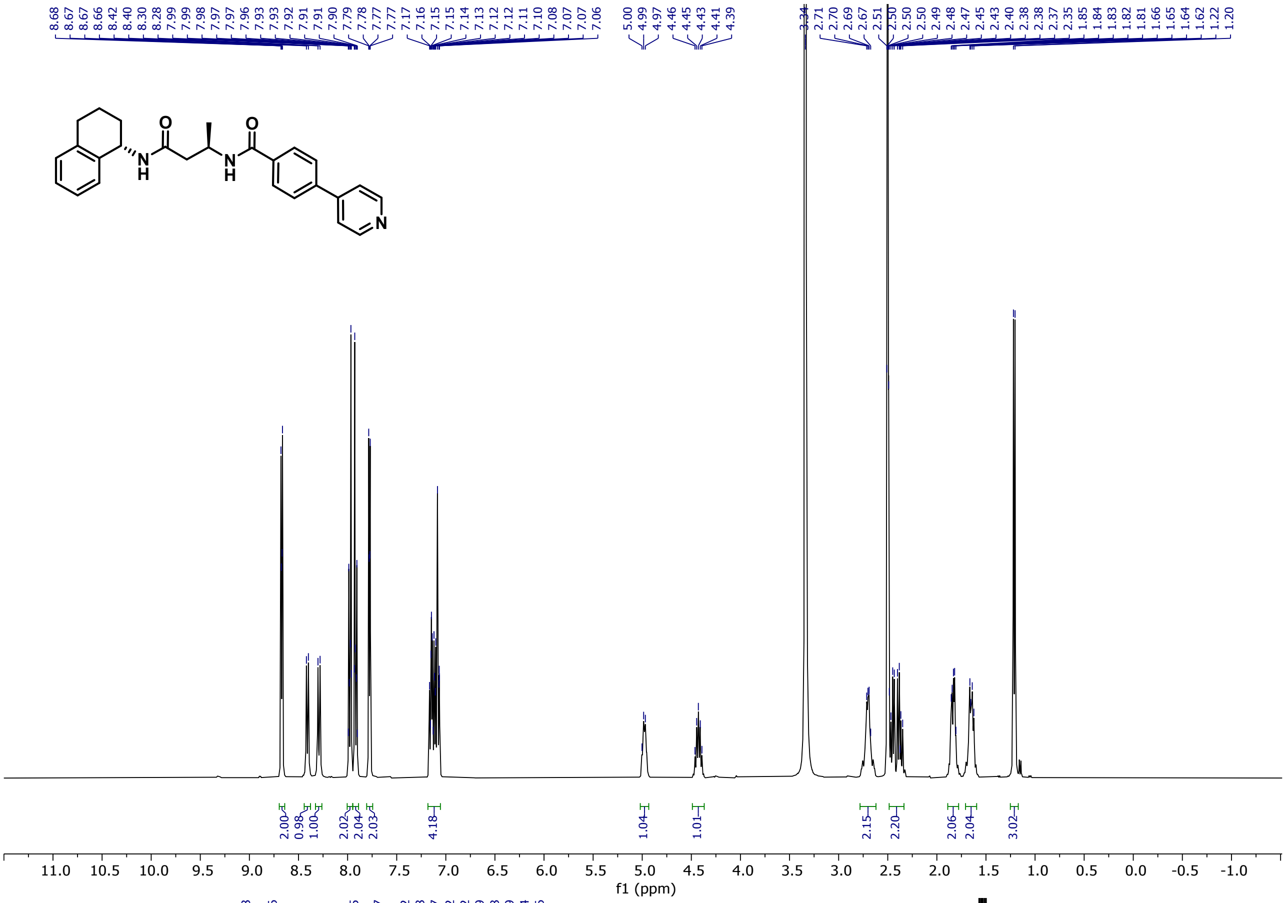

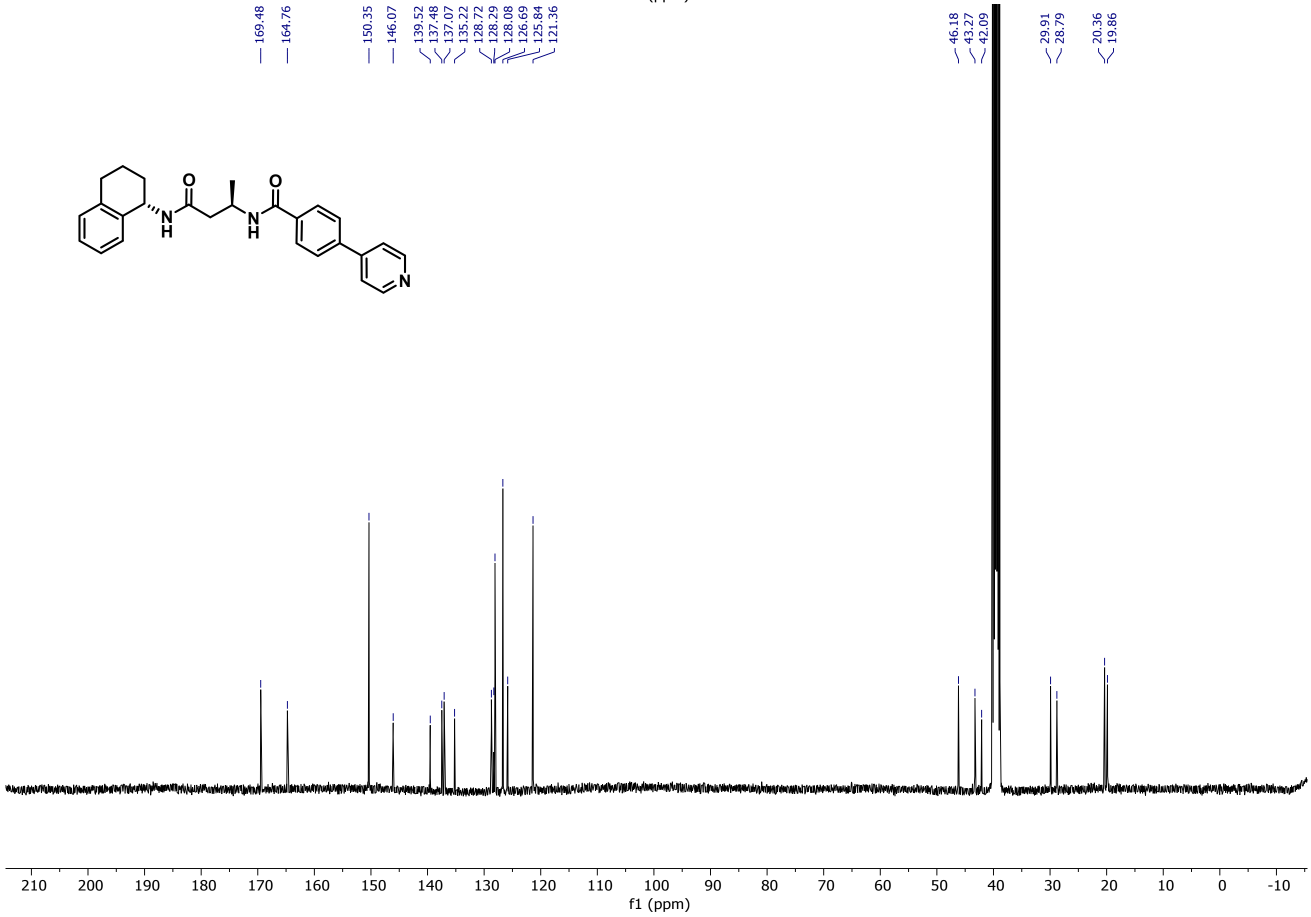

NMR spectra of 11n (VU6080195) in DMSO-d_6.

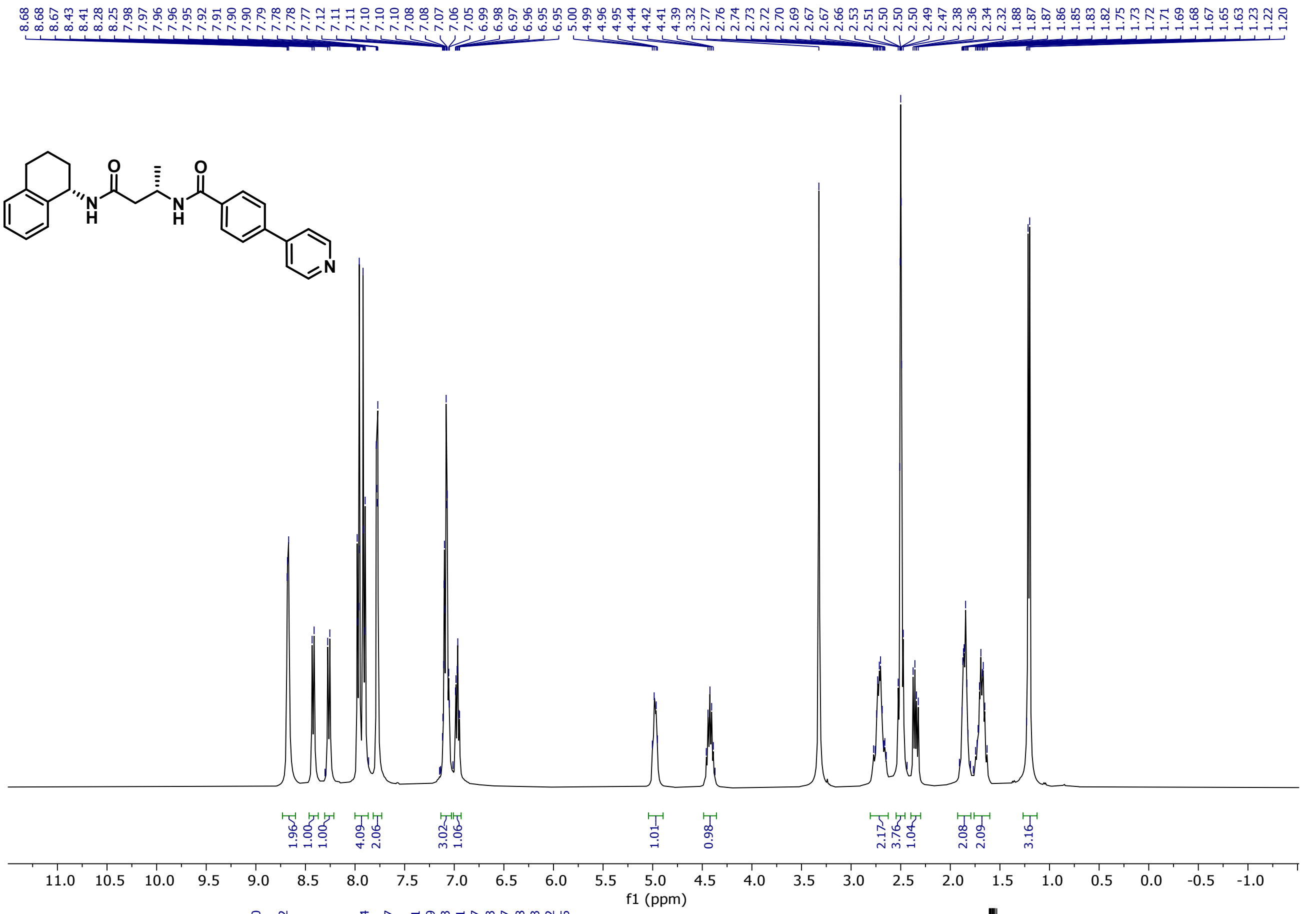

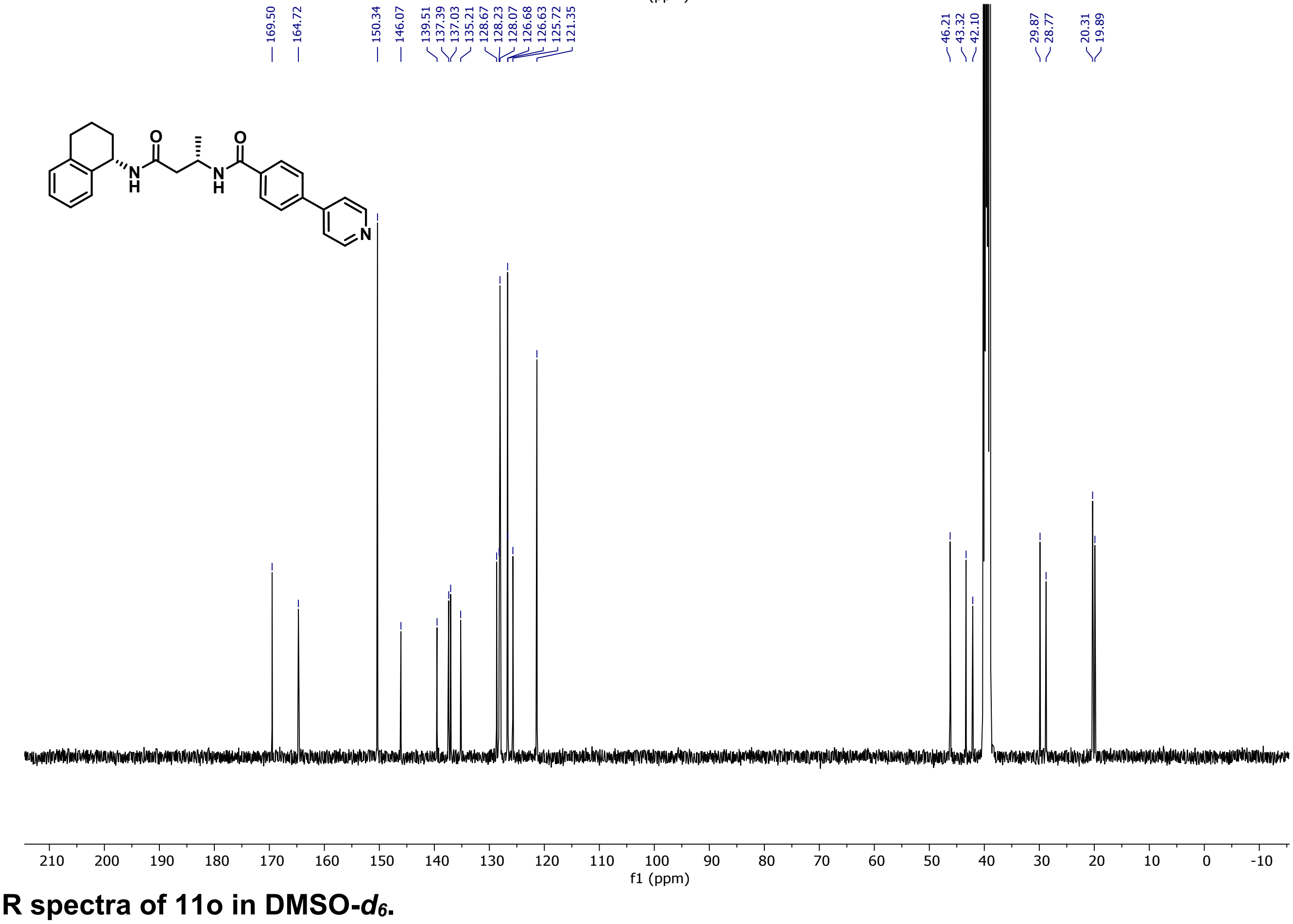

NMR spectra of 11o in DMSO-*d*$_6$.

TAOK1 and 2 Activities of Selected Compounds

Table S1. Activities of Analogues of Compound 43 (1).

Compound	Structure	Percent Enzyme Activity at 10 µM (Single Dose)		TAOK1 IC_{50} (nM)	TAOK2 IC_{50} (nM)	TAOK1 Selectivity
**9i**	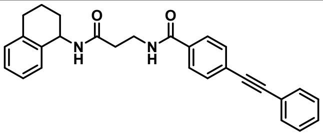	79.2	89.6	n.d.	n.d.	n.d.
**9j**	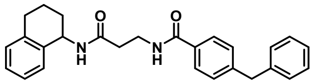	94.3	115.3	n.d.	n.d.	n.d.
**9k**	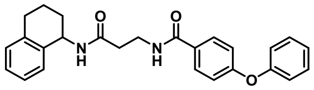	80.3	109.8	n.d.	n.d.	n.d.
**9l**	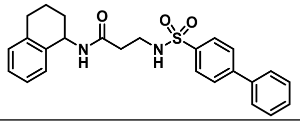	64.7	83.5	n.d.	n.d.	n.d.

Table S2. Activities of Analogues of Compound 9d (VU6071680).

Compound	Structure	Percent Enzyme Activity at 10 µM (Single Dose)		TAOK1 IC_{50} (nM)	TAOK2 IC_{50} (nM)	TAOK1 Selectivity
		TAOK1	TAOK2			
**10r**	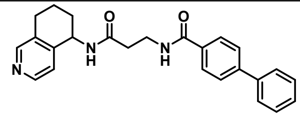	90.5	99.7	n.d.	n.d.	n.d.
**10s**	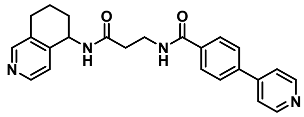	89.8	91.9	n.d.	n.d.	n.d.
**10t**	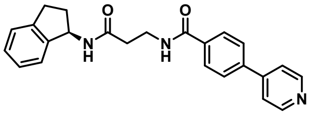	38.8	76.4	6,600	>10,000	>1.5
**10u**	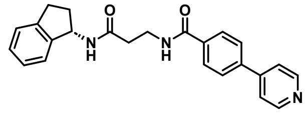	35.4	84.3	3,980	Inactive	>2.5
**10v**	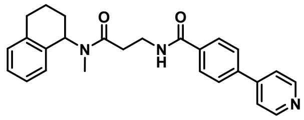	7.7	22.3	629	2,340	3.7
**10w**	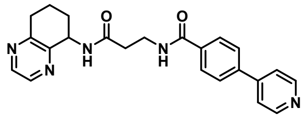	95.7	93.9	n.d.	n.d.	n.d.
**10x**	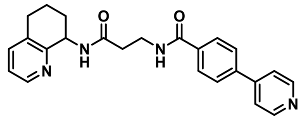	92.9	95.5	n.d.	n.d.	n.d.
**10y**	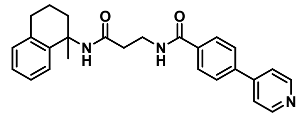	62.7	126.1	n.d.	n.d.	n.d.
**10z**	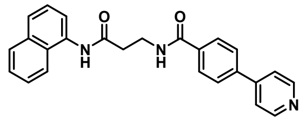	53.3	84.6	n.d.	n.d.	n.d.
**10aa**	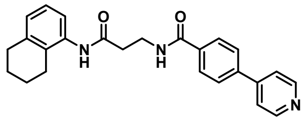	75.2	90.9	n.d.	n.d.	n.d.
**10ab**	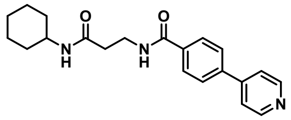	88.0	95.8	n.d.	n.d.	n.d.
**10ac**	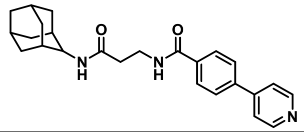	85.2	93.6	n.d.	n.d.	n.d.

DMPK

In vitro

Plasma protein binding and Brain homogenate binding: Determination of fraction unbound (f_u) in plasma was conducted in vitro via equilibrium dialysis using HTDialysis (HTD) membrane plates. The top half of the plate was filled with 100 uL of Dubelco's Phosphate Buffered Saline, pH 7.4 (DPBS). Compounds were diluted into plasma from each species (5 µM final concentration), which was aliquoted in triplicate to the 'bottom half' of the prepared HTD plate wells. The HTD plate was sealed and incubated for 6 hours at 37 ˚C. Following incubation, each well (both top and bottom halves) was transferred (20 µL) to the corresponding wells of a 96-shallow-well (V-bottom) plate. The daughter plates were then matrix-matched (DPBS side wells received equal volume of plasma, and plasma side wells received equal volume of DPBS), and extraction solution (120 µL; acetonitrile containing 50 nM carbamazepine as IS) was added to all wells of both daughter plates to precipitate protein and extract the test article. The plates were then sealed and centrifuged (3500 rcf) for 10 minutes at ambient temperature. Supernatant (60 µL) from each well of the daughter plates was then transferred to the corresponding wells of new daughter plates (96-shallow-well, V bottom) containing water (Milli-Q, 60 µL/well), and the plates were sealed in preparation for LC-MS/MS analysis (see LC-MS/MS analysis method below).

The unbound fraction (f_u) was calculated following the equation below, and mean values for each species were calculated from 3 replicates.

A similar approach was used to determine the degree of brain homogenate binding, which employed the same methodology and procedure with the following modifications: 1) a final compound concentration of 1 µM was used, 2) naïve rat brains were homogenized in DPBS (1:3 composition of brain: DPBS, w/w) using a Mini-Bead Beater™ machine in order to obtain brain homogenate, which was then treated in the same manner as the plasma samples in the previously described plasma protein binding assay. Fraction unbound for both plasma and brain samples was determined using Equation 4.

$$f_u = \frac{Conc_{buffer}}{Conc_{plasma}}$$

Equation 4 Determination of fraction unbound in plasma.

The diluted fraction unbound (f_u2) in brain was calculated in the same manner by using brain homogenate rather than plasma. Undiluted fraction unbound for the brain was calculated using Equation 5

$$f_u = \frac{1/4}{\left\{\left(\frac{1}{fu2}\right) - 1\right\} + 1/4}$$

Equation 5 Determination of fraction unbound in brain. F_u2 represents the diluted fraction unbound.

Intrinsic clearance: Human or rat hepatic microsomes (0.5 mg/mL) and 1 µM test compound were incubated in 100 mM potassium phosphate pH 7.4 buffer with 3 mM $MgCl_2$ at 37 °C with constant shaking. After a 5 min preincubation, the reaction was initiated by the addition of NADPH (1 mM). At selected time intervals (0, 3, 7,

15, 25, and 45 min), aliquots were taken and subsequently placed into a 96-well plate containing cold acetonitrile with internal standard (50 ng/mL carbamazepine). Plates were then centrifuged at 3000 rcf (4 °C) for 10 min, and the supernatant was transferred to a separate 96-well plate and diluted 1:1 with water for LC/MS/MS analysis. The *in vitro* half-life ($_{1/2}$, min, Eq. 1), intrinsic clearance (CL_{int}, mL/min/kg, Eq. 2), and subsequent predicted hepatic clearance (CL_{hep}, mL/min/kg, Eq. 3) was determined employing the following equations:

(1) $T_{1/2} = \frac{Ln(2)}{K}$

where k represents the slope from linear regression analysis of the natural log percent remaining of a test compound as a function of incubation time

(2) $CL_{int} = \frac{0.693}{in\ vitro T_{1/2}} x \frac{mL\ incubation}{mg\ microsomes} x \frac{45\ mg\ microsomes}{gram\ liver} x \frac{20^{a}\ gram\ liver}{kg\ body\ wt}$

[a]scale-up factors: of 20 (human) or 45 (rat)

(3) $CL_{hep} = \frac{Q_h \cdot CL_{int}}{Q_h + CL_{int}}$

where Q_h (hepatic blood flow, mL/min/kg) is 21 (human) or 70 (rat).

LC/MS/MS Bioanalysis of Samples from Plasma Protein Binding and Intrinsic Clearance Assays:
Samples were analyzed on a Thermo Electron TSQ Quantum Ultra triple quad mass spectrometer (San Jose, CA) via electrospray ionization (ESI) with two Themo Electron Accella pumps (San Jose, CA), and a Leap Technologies CTC PAL autosampler (Carrboro, NC). Analytes were separated by gradient elution on a dual column system with two Thermo Hypersil Gold (2.1 x 30 mm, 1.9 µm) columns (San Jose, CA) thermostated at 40 °C. HPLC mobile phase A was 0.1% formic acid in water and mobile phase B was 0.1% formic acid in acetonitrile. The gradient started at 10% B after a 0.2 min hold and was linearly increased to 95% B over 0.8 min; hold at 95% B for 0.2 min; returned to 10% B in 0.1 min. The total run time was 1.3 min and the HPLC flow rate was 0.8 mL/min. While pump 1 ran the gradient method, pump 2 equilibrated the alternate column isocratically at 10% B. Compound optimization, data collection, and processing were performed using Thermo Electron's QuickQuan software (v2.3) and Xcalibur (v2.0.7 SP1).

***In vivo* DMPK experimental:**
Determination of brain to plasma ratio:
Animal care and use

All animal study procedures were approved by the Institutional Animal Care and Use Committee and were conducted in accordance with the National Institutes of Health regulations of animal care covered in Principles of Laboratory Animal Care (National Institutes of Health). All rats were fasted overnight prior to testing.

In-life phase

For determination of the brain over plasma ratio (K_p), compounds were formulated in 8% ethanol, 32% PEG400 and 60% DMSO (*v/v/v*) and administered as a single 0.2 mg/kg IV dose (1 mL/kg) to male, Sprague Dawley rats (*n* = 1) via injection into a surgically-implanted jugular vein catheter. At 15 min post dosing, blood sample was collected into chilled, K_2EDTA anticoagulant-fortified tube and immediately placed on wet ice. The blood sample was then centrifuged (1700 rcf, 5 minutes, 4 ˚C) to obtain plasma sample. At the same post-administration time point, whole brain sample was obtained by rapid dissection, rinsed with PBS, and immediately frozen in individual tissue collection box (dry ice). All brain and plasma samples were stored at -80 ˚C until analysis by LC-MS/MS.

Sample Analysis: Concentrations in plasma and brain homogenates were quantified by liquid chromatography tandem mass spectrometry (LC-MS/MS). Whole brains were homogenized in 3 mL of 70:30 IPA:water in a mini bead beater for 3 min, and centrifuged at 3,500 g for 5 min. 5 uL of the supernatant was diluted in 15 uL of blank plasma for quantification of the analytes. Plasma samples were centrifuged at 3,500 g for 5 min. A standard curve was generated by diluting the analytes DMSO stocks with blank plasma to obtain a final concentration of 10,000 ng/ml followed by a serial dilution down to 0.5 ng/ml. Quality controls were generated by a serial dilution of the 5,000 ng/ml standard curve solution in blank plasma to obtain 3 concentrations of 500, 50, and 5 ng/ml. 20 uL of brain diluted in plasma, plasma, blank plasma, standard curve and QC samples were loaded in a V-bottom 96-well plate. 120 uL of acetonitrile containing 0.05 uM carbamazepine (internal standard) was added to each well and the plate was centrifuged at 3,500 g for 5 min. 60 uL of the supernatant of each well (protein free) was transferred to a new 96-well plate containing 60 uL of water. The plates were sealed for analysis by LC-MS/MS.

Plasma and brain tissue samples originating from *in vivo* studies were analyzed by electrospray ionization using an AB Sciex Q-TRAP 5500 (Foster City, CA) that was coupled to a Shimadzu LC-20AD pump (Columbia, MD) and a Leap Technologies CTC PAL auto-sampler (Carrboro, NC). Analytes were separated by gradient elution using a C18 column (3 x 50 mm, 3 mm; Fortis Technologies Ltd, Cheshire, UK) that was thermostated at 40 °C. HPLC mobile phase A was 0.1% formic acid in water (pH unadjusted); mobile phase B was 0.1% formic acid in acetonitrile (pH unadjusted). A 10% B gradient was held for 0.2 min and was linearly increased to 90% B over 0.8 min, with an isocratic hold for 0.5 min, before transitioning to 10% B over 0.05 min. The column was re-equilibrated (1 min) before the next sample injection. The total run time was 2.55 min, and the HPLC flow rate was 0.5 ml/min. The source temperature was set at 500 °C, and mass spectral analyses were performed using a Turbo-Ion spray source in positive ionization mode (5.0-kV spray voltage) and using multiple-reaction monitoring of transitions specific for the analytes. All data were analyzed using AB Sciex Analyst 1.5.1 software.

Brain plasma concentration ratio (K_p) was calculated by dividing brain concentration by plasma concentration for each animal. Unbound brain to unbound plasma concentration ratio ($K_{p,uu}$) is calculated using the following formula: $K_{p,uu}$ = (Brain ng/g x brain fu)/(plasma ng/ml x plasma fu).

Pharmacokinetic profiles in rats following oral single escalating doses

Single escalating oral dosing in Sprague-Dawley rats was performed at Frontage Laboratories according to their non-GLP Standard Operating Procedure and IACUC protocols. In short, compounds were formulated in 10% Tween 80 in water and dosed at 10 mg/kg. At different times,
arterial blood was collected from a femoral artery catheter, and compound concentration was determined in plasma by LC-MS/MS following their non-GLP protocol. PK parameters were determined using Phoenix WinNonlin software (version 6.3).

General HotSpot Kinase Assay Protocol (provided by Reaction Biology)

A. **Assay principle**

Compounds are pre-incubated with kinase and substrate (and cofactors if needed) mixtures at room temperature, and then the reaction is initiated by the addition of radioisotopically-labeled ATP (33P-ɣ-ATP). The reaction mixtures are then spotted onto filter paper, that binds the radioisotope-labeled catalytic product (33P-substrate). Unreacted 33P-ATP is removed via washing of the filter paper.

B. **Reagent**

Base Reaction buffer; 20 mM Hepes (pH 7.5), 10 mM $MgCl_2$, 1 mM EGTA, 0.01% Brij35, 0.02 mg/mL BSA, 0.1 mM Na_3VO_4, 2 mM DTT, 1% DMSO Required cofactors are added individually to each kinase reaction.

C. **Reaction Procedure**

1. Prepare substrate in freshly prepared Reaction Buffer
2. Deliver any required cofactors to the substrate solution above
3. Deliver kinase into the substrate solution and gently mix
4. Deliver compounds in 100% DMSO into the kinase reaction mixture by Acoustic technology (Echo550 or equivalent; nanoliter range), incubate for 20 min at room temperature
5. Deliver 33P-ATP into the reaction mixture to initiate the reaction
6. Incubate for 2 hours at room temperature
7. Detect kinase activity by P81 filter-binding method

D. **Data Analysis**

Kinase activity data were expressed as the percent remaining kinase activity in test samples compared to vehicle (dimethyl sulfoxide) reactions. IC50 values and curve fits were obtained using GraphPad Prism software.

E. **Enzyme Source and Substrates**

TAOK1

Enzyme Source: SignalChem, Cat# T24-11G-10

Km ATP: 30μM

Substrate: MBP, SignalChem, Cat# M42-51N

TAOK2

Enzyme Source: SignalChem, Cat# PV3760

Km ATP: 100μM

Substrate: MBP, SignalChem, Cat# M42-51N

TAOK3

Enzyme Source: SignalChem, Cat# PR6434A

Km ATP: 20μM

Substrate: MBP, SignalChem, Cat# M42-51N

F. Reference

Anastassiadis T, et al. Comprehensive assay of kinase catalytic activity reveals features of kinase inhibitor selectivity. Nat Biotechnol. 2011 Oct 30;29(11):1039-45. doi: 10.1038/nbt.2017.

Compound 43 (VU6063661) Kinase Selectivity Panel (Eurofins)

Methods: For most assays, kinase-tagged T7 phage strains were prepared in an *E. coli* host derived from the BL21 strain. *E. coli* were grown to log-phase and infected with T7 phage and incubated with shaking at 32°C until lysis. The lysates were centrifuged and filtered to remove cell debris. The remaining kinases were produced in HEK-293 cells and subsequently tagged with DNA for qPCR detection. Streptavidin-coated magnetic beads were treated with biotinylated small molecule ligands for 30 minutes at room temperature to generate affinity resins for kinase assays. The liganded beads were blocked with excess biotin and washed with blocking buffer (SeaBlock (Pierce), 1% BSA, 0.05% Tween 20, 1 mM DTT) to remove unbound ligand and to reduce non-specific binding. Binding reactions were assembled by combining kinases, liganded affinity beads, and test compounds in 1x binding buffer (20% SeaBlock, 0.17x PBS, 0.05% Tween 20, 6 mM DTT). Test compounds were prepared as 111X stocks in 100% DMSO. Kds were determined using an 11-point 3-fold compound dilution series with three DMSO control points. All compounds for Kd measurements are distributed by acoustic transfer (non-contact dispensing) in 100% DMSO. The compounds were then diluted directly into the assays such that the final concentration of DMSO was 0.9%. All reactions performed in polypropylene 384-well plate. Each was a final volume of 0.02 ml. The assay plates were incubated at room temperature with shaking for 1 hour and the affinity beads were washed with wash buffer (1x PBS, 0.05% Tween 20). The beads were then re-suspended in elution buffer (1x PBS, 0.05% Tween 20, 0.5 µM nonbiotinylated affinity ligand) and incubated at room temperature with shaking for 30 minutes. The kinase concentration in the eluates was measured by qPCR.

Binding Constants (Kds)

Binding constants (Kds) were calculated with a standard dose-response curve using the Hill equation:

$$Response = Background + \frac{Signal - Background}{1 + \left(\frac{Kd^{Hill\,Slope}}{Dose^{Hill\,Slope}}\right)}$$

The Hill Slope was set to -1.

Curves were fitted using a non-linear least square fit with the Levenberg-Marquardt algorithm.

References

KINOME*scan*™ and BROMO*scan*™ use the same assay technology. For a more detailed description of this assay technology, see:

Fabian, M.A. *et al*. A small molecule-kinase interaction map for clinical kinase inhibitors. *Nat. Biotechnol.* **23**, 329-336 (2005).

Kinase	Percent Activity at 10 μM
AAK1(h)	95
Abl(h)	97
ACK1(h)	111
ACTR2(h)	98
ALK(h)	98
ALK1(h)	91
ALK2(h)	85
ALK4(h)	100
ALK6(h)	100
Arg(h)	105
AMPKα1(h)	100
AMPKα2(h)	98
A-Raf(h)	104
ARK5(h)	105
ASK1(h)	96
Aurora-A(h)	106
Aurora-B(h)	98
Aurora-C(h)	95
Axl(h)	102
BIKe(h)	87
Blk(h)	99
BMPR2(h)	104
Bmx(h)	96
BRK(h)	106
BrSK1(h)	95
BrSK2(h)	101
BTK(h)	108
B-Raf(h)	111
CaMKI(h)	110
CaMKIβ(h)	101
CaMKIγ(h)	105
CaMKIIα(h)	96
CaMKIIβ(h)	100
CaMKIIγ(h)	94
CaMKIδ(h)	103
CaMKIIδ(h)	96
CaMKIV(h)	105
CaMKK1(h)	103
CaMKK2(h)	97
Cdc7/cyclinB1(h)	80
CDK1/cyclinB(h)	92
CDK2/cyclinA(h)	97
CDK2/cyclinE(h)	88
CDK3/cyclinE(h)	93
CDK4/cyclinD3(h)	87
CDK5/p25(h)	105
CDK5/p35(h)	101
CDK6/cyclinD3(h)	106
CDK7/cyclinH/MAT1(h)	109
CDK9/cyclin T1(h)	109

Kinase	Percent Activity at 10 μM
CDK14/cyclinY(h)	98
CDK16/cyclinY(h)	111
CDK17/cyclinY(h)	128
CDK18/cyclinY(h)	108
CDKL1(h)	90
CDKL2(h)	93
CDKL3(h)	95
CDKL4(h)	98
ChaK1(h)	103
CHK1(h)	101
CHK2(h)	103
CK1α(h)	95
CK1ε(h)	93
CK1γ1(h)	102
CK1γ2(h)	100
CK1γ3(h)	92
CK1δ(h)	103
CK2(h)	100
CK2α1(h)	96
CK2α2(h)	100
CLIK1(h)	100
CLK1(h)	96
CLK2(h)	103
CLK3(h)	99
CLK4(h)	102
cKit(h)	105
COT(h)	83
CRIK(h)	93
CSK(h)	119
c-RAF(h)	108
cSRC(h)	109
DAPK1(h)	106
DAPK2(h)	95
DCAMKL1(h)	110
DCAMKL2(h)	131
DCAMKL3(h)	97
DDR1(h)	102
DDR2(h)	108
DMPK(h)	84
DRAK1(h)	75
DRAK2(h)	94
DYRK1A(h)	97
DYRK1B(h)	100
DYRK2(h)	100
DYRK3(h)	92
eEF-2K(h)	113
EGFR(h)	101
EphA1(h)	95
EphA2(h)	111

Kinase	Percent Activity at 10 µM
EphA3(h)	109
EphA4(h)	102
EphA5(h)	104
EphA7(h)	111
EphA8(h)	99
EphB2(h)	92
EphB1(h)	110
EphB3(h)	93
EphB4(h)	111
ErbB2(h)	93
ErbB4(h)	103
FAK(h)	86
Fer(h)	82
Fes(h)	101
FGFR1(h)	103
FGFR2(h)	103
FGFR3(h)	105
FGFR4(h)	99
Fgr(h)	99
Flt1(h)	93
Flt3(h)	85
Flt4(h)	91
Fms(h)	100
Fyn(h)	99
GAK(h)	102
GCK(h)	87
GCN2(h)	87
GRK1(h)	99
GRK2(h)	88
GRK3(h)	103
GRK4(h)	94
GRK5(h)	98
GRK6(h)	99
GRK7(h)	98
GSK3α(h)	101
GSK3β(h)	105
Haspin(h)	87
Hck(h)	87
Hck(h) activated	108
HIPK1(h)	114
HIPK2(h)	96
HIPK3(h)	101
HIPK4(h)	93
HPK1(h)	96
HRI(h)	90
ICK(h)	113
IGF-1R(h)	110
IGF-1R(h), activated	104
IKKα(h)	106
IKKβ(h)	105

Kinase	Percent Activity at 10 µM
IKKε(h)	100
IR(h)	97
IR(h), activated	95
IRE1(h)	93
IRR(h)	112
IRAK1(h)	105
IRAK4(h)	90
Itk(h)	104
JAK1(h)	102
JAK2(h)	106
JAK3(h)	92
JNK1α1(h)	104
JNK2α2(h)	104
JNK3(h)	102
KDR(h)	105
LATS1(h)	93
LATS2(h)	87
Lck(h)	82
Lck(h) activated	104
LIMK1(h)	106
LIMK2(h)	93
LKB1(h)	91
LOK(h)	109
Lyn(h)	115
LRRK2(h)	96
LTK(h)	89
MAK(h)	99
MAPK1(h)	101
MAPK2(h)	104
MAP4K3(h)	120
MAP4K4(h)	87
MAP4K5(h)	98
MAPKAP-K2(h)	86
MAPKAP-K3(h)	86
MATK(h)	102
MEK1(h)	88
MEK2(h)	99
MARK1(h)	95
MARK3(h)	96
MARK4(h)	101
MEKK2(h)	97
MEKK3(h)	107
MELK(h)	97
Mer(h)	103
Met(h)	116
MINK(h)	92
MKK3(h)	113
MKK6(h)	99
MLCK(h)	97
MLK1(h)	94

Kinase	Percent Activity at 10 μM
MLK2(h)	99
MLK3(h)	98
MLK4(h)	98
Mnk1(h)	112
Mnk2(h)	95
MOK(h)	101
MRCKα(h)	102
MRCKβ(h)	96
MRCKγ(h)	84
MSK1(h)	90
MSK2(h)	108
MSSK1(h)	119
MST1(h)	106
MST2(h)	111
MST3(h)	95
MST4(h)	78
mTOR(h)	97
mTOR/FKBP12(h)	101
MuSK(h)	82
MYLK2(h)	103
MYO3B(h)	95
NDR1(h)	108
NDR2(h)	98
NEK1(h)	102
NEK2(h)	108
NEK3(h)	79
NEK4(h)	99
NEK5(h)	108
NEK6(h)	109
NEK7(h)	97
NEK9(h)	101
NIM1(h)	95
NEK11(h)	92
NLK(h)	95
NUAK2(h)	95
OSR1(h)	105
p70S6K(h)	105
p70S6Kβ(h)	118
PAK1(h)	103
PAK2(h)	99
PAK4(h)	96
PAK3(h)	91
PAK5(h)	83
PAK6(h)	97
PAR-1Bα(h)	99
PASK(h)	105
PEK(h)	101
PDGFRα(h)	93
PDGFRβ(h)	120
PDHK2(h)	91

Kinase	Percent Activity at 10 μM
PDHK4(h)	102
PDK1(h)	87
PhKγ1(h)	97
PhKγ2(h)	93
Pim-1(h)	93
Pim-2(h)	105
Pim-3(h)	95
PKA(h)	100
PKAcβ(h)	93
PKBα(h)	107
PKBβ(h)	95
PKBγ(h)	98
PKCα(h)	105
PKCβI(h)	94
PKCβII(h)	111
PKCγ(h)	107
PKCδ(h)	100
PKCε(h)	105
PKCη(h)	98
PKCι(h)	99
PKCμ(h)	102
PKCθ(h)	107
PKCζ(h)	100
PKD2(h)	100
PKD3(h)	102
PKG1α(h)	108
PKG1β(h)	105
PKR(h)	104
Plk1(h)	98
Plk3(h)	99
Plk4(h)	95
PRAK(h)	78
PRKG2(h)	96
PRK1(h)	96
PRK2(h)	105
PrKX(h)	85
PRP4(h)	92
PTK5(h)	98
Pyk2(h)	98
Ret(h)	107
RIPK1(h)	93
RIPK2(h)	97
ROCK-I(h)	115
ROCK-II(h)	108
Ron(h)	98
Ros(h)	89
Rse(h)	100
Rsk1(h)	73
Rsk2(h)	97
Rsk3(h)	100

Kinase	Percent Activity at 10 μM
Rsk4(h)	101
SAPK2a(h)	96
SAPK2b(h)	88
SAPK3(h)	112
SAPK4(h)	105
SBK1(h)	117
SGK(h)	101
SGK2(h)	102
SGK3(h)	91
SIK(h)	102
SIK2(h)	100
SIK3(h)	101
SLK(h)	118
Snk(h)	101
SNRK(h)	111
SRMS(h)	86
SRPK1(h)	98
SRPK2(h)	102
STK16(h)	93
STK25(h)	101
STK32A(h)	99
STK32B(h)	99
STK32C(h)	98
STK33(h)	107
STK39(h)	100
Syk(h)	89
TAF1L(h)	94
TAK1(h)	107
TAO1(h)	1
TAO2(h)	2
TAO3(h)	5
TBK1(h)	98
Tec(h) activated	83
TGFBR1(h)	98
TGFBR2(h)	103
Tie2 (h)	107
TLK1(h)	92
TLK2(h)	95
TNIK(h)	129
TRB2(h)	91
TrkA(h)	83
TrkB(h)	104
TrkC(h)	100
TSSK1(h)	101
TSSK2(h)	96
TSSK3(h)	107
TSSK4(h)	106
TTBK1(h)	96
TTBK2(h)	93

Kinase	Percent Activity at 10 μM
TTK(h)	105
Txk(h)	87
TYK2(h)	97
ULK1(h)	96
ULK2(h)	102
ULK3(h)	95
VRK1(h)	109
VRK2(h)	90
Wee1(h)	103
Wee1B(h)	97
WNK1(h)	130
WNK2(h)	93
WNK3(h)	98
WNK4(h)	93
Yes(h)	101
ZAK(h)	104
ZAP-70(h)	92
ZIPK(h)	108
ATM(h)	98
ATR/ATRIP(h)	100
DNA-PK(h)	101
PI3 Kinase (p110b/p85a)(h)	97
PI3 Kinase (p120g)(h)	76
PI3 Kinase (p110d/p85a)(h)	80
PI3 Kinase (p110a/p85a)(h)	99
PI3KC2a(h)	106
PI3KC2g(h)	96
PIP4K2a(h)	106
PIP5K1a(h)	108
PIP5K1g(h)	106

Compound 43 (VU6063661) Lead Profiling Radioligand Binding Studies (Eurofins)

Radioligand Binding Assay Results

Assay Name	Species	Rep.	Conc.	% Inh.
Adenosine A1	hum	2	10 µM	22
Adenosine A2A	hum	2	10 µM	0
Adenosine A3	hum	2	10 µM	55
Adrenergic α1A	hum	2	10 µM	26
Adrenergic α1B	hum	2	10 µM	42
Adrenergic α1D	hum	2	10 µM	25
Adrenergic α2A	hum	2	10 µM	42
Adrenergic β1	hum	2	10 µM	25
Adrenergic β2	hum	2	10 µM	100
Androgen (Testosterone)	hum	2	10 µM	8
Bradykinin B1	hum	2	10 µM	-1
Bradykinin B2	hum	2	10 µM	20
Calcium Channel L-Type, Benzothiazepine	rat	2	10 µM	13
Calcium Channel L-Type, Dihydropyridine	rat	2	10 µM	55
Calcium Channel N-Type	rat	2	10 µM	8
Cannabinoid CB1	hum	2	10 µM	12
Dopamine D1	hum	2	10 µM	28
Dopamine D2S	hum	2	10 µM	12
Dopamine D3	hum	2	10 µM	25
Dopamine D4.4	hum	2	10 µM	72
Endothelin ETA	hum	2	10 µM	1
Endothelin ETB	hum	2	10 µM	7
Epidermal Growth Factor	hum	2	10 µM	19
Estrogen ERα	hum	2	10 µM	-3
GABAA, Flunitrazepam, Central	rat	2	10 µM	9
GABAA, Muscimol, Central	rat	2	10 µM	3
GABAB1A	hum	2	10 µM	6
Glucocorticoid	hum	2	10 µM	23
Glutamate, Kainate	rat	2	10 µM	-14
GABAA, Flunitrazepam, Central	rat	2	10 µM	19
Glutamate, NMDA, Glycine	rat	2	10 µM	2
Glutamate, NMDA, Phencyclidine	rat	2	10 µM	3
Histamine H1	hum	2	10 µM	21
Histamine H2	hum	2	10 µM	-22
Histamine H3	hum	2	10 µM	14
Imidazoline I2, Central	rat	2	10 µM	19
Interleukin IL-1 R1	hum	2	10 µM	4
Leukotriene, Cysteinyl CysLT1	hum	2	10 µM	27
Melatonin MT1	hum	2	10 µM	34
Muscarinic M1	hum	2	10 µM	2
Muscarinic M2	hum	2	10 µM	-13
Muscarinic M3	hum	2	10 µM	4

Neuropeptide Y Y1	hum	2	10 µM	-6
Neuropeptide Y Y2	hum	2	10 µM	10
Nicotinic Acetylcholine α1, Bungarotoxin	hum	2	10 µM	2
Nicotinic Acetylcholine α3β4	hum	2	10 µM	-6
Opiate δ1 (OP1, DOP)	hum	2	10 µM	1
Opiate κ (OP2, KOP)	hum	2	10 µM	17
Opiate µ (OP3, MOP)	hum	2	10 µM	14
Phorbol Ester	mouse	2	10 µM	-3
Platelet Activating Factor (PAF)	hum	2	10 µM	4
Potassium Channel [KATP]	ham	2	10 µM	14
Potassium Channel hERG, [3H]Dofetilide	hum	2	10 µM	48
Prostanoid EP4	hum	2	10 µM	37
Purinergic P2X	rat	2	10 µM	-15
Purinergic P2Y, Non-Selective	rat	2	10 µM	1
Rolipram	rat	2	10 µM	10
Serotonin (5-Hydroxytryptamine) 5-HT1A	hum	2	10 µM	42
Serotonin (5-Hydroxytryptamine) 5-HT2B	hum	2	10 µM	91
Serotonin (5-Hydroxytryptamine) 5-HT3	hum	2	10 µM	-26
Sigma σ1	hum	2	10 µM	33
Sodium Channel, Site	rat	2	10 µM	48
Tachykinin NK1	hum	2	10 µM	19
Thyroid Hormone	rat	2	10 µM	34
Transporter, Dopamine (DAT)	hum	2	10 µM	29
Transporter, GABA	rat	2	10 µM	-3
Transporter, Norepinephrine (NET)	hum	2	10 µM	58
274030 Transporter, Serotonin (5-Hydroxytryptamine) (SERT)	hum	2	10 µM	29

VU6071680 Eurofins Off-Target Kinase Panel

Methods: Methods are the same as for Compound 43 (VU6063661).

Kinases	% Enzyme Activity (relative to DMSO controls) VU6071680 Data 1	Data 2	IC_{50} (M) Control Cmpd	Control Cmpd ID
ABL1	97.97	96.61	3.28E-08	STAUROSPORINE
AKT1	94.24	93.99	4.95E-09	STAUROSPORINE
ALK4/ACVR1B	107.50	102.01	7.94E-08	LDN193189
ARK5/NUAK1	96.01	95.73	8.49E-10	STAUROSPORINE
Aurora A	80.53	75.57	2.27E-09	STAUROSPORINE
BRAF	109.37	109.15	1.39E-08	GW5074
BTK	103.97	103.56	1.87E-08	STAUROSPORINE
c-Kit	96.93	96.54	8.03E-10	STAUROSPORINE
c-MET	97.33	93.80	2.13E-08	STAUROSPORINE
c-Src	103.61	102.66	2.56E-09	STAUROSPORINE
CAMK1a	101.24	100.40	3.14E-09	STAUROSPORINE
CDK1/cyclin B	96.49	94.47	1.14E-09	STAUROSPORINE
CDK4/cyclin D1	102.47	99.39	1.04E-08	STAUROSPORINE
CDK7/cyclin H	76.45	75.41	6.55E-08	STAUROSPORINE
CDK9/cyclin T2	83.65	82.72	3.43E-09	STAUROSPORINE
CHK1	100.22	99.38	1.14E-10	STAUROSPORINE
CK1a1	94.31	92.28	3.83E-06	STAUROSPORINE
CK1g1	91.96	90.58	5.09E-06	STAUROSPORINE
CK2a	105.70	104.73	9.66E-08	GW5074
CLK2	30.07	29.14	4.11E-09	STAUROSPORINE
DAPK2	99.44	96.96	5.91E-09	STAUROSPORINE
DCAMKL1	95.65	95.11	9.75E-08	STAUROSPORINE
DYRK1/DYRK1A	21.31	20.85	2.17E-09	STAUROSPORINE
EGFR	99.33	98.78	7.19E-08	STAUROSPORINE
EPHA5	98.55	97.97	1.60E-08	STAUROSPORINE
EPHB2	98.87	97.69	6.50E-08	STAUROSPORINE
ERK1	107.67	104.39	5.65E-09	SCH772984
FGFR2	106.10	100.17	2.05E-09	STAUROSPORINE
FLT1/VEGFR1	86.87	86.19	5.55E-09	STAUROSPORINE
FLT3	69.29	68.25	6.36E-10	STAUROSPORINE
GSK3b	92.78	92.45	3.38E-09	STAUROSPORINE
HIPK2	65.27	64.75	2.84E-07	STAUROSPORINE
IGF1R	101.86	99.13	3.67E-08	STAUROSPORINE
IKKe/IKBKE	105.61	103.27	1.88E-10	STAUROSPORINE
IRAK4	104.95	101.73	1.10E-08	STAUROSPORINE
JAK2	101.95	100.78	1.45E-10	STAUROSPORINE
JNK3	87.52	87.25	3.00E-08	JNKI VIII
LIMK1	93.20	92.53	5.98E-10	STAUROSPORINE
LOK/STK10	94.19	91.62	4.59E-08	RO-31-8220

Kinases	% Enzyme Activity (relative to DMSO controls) VU6071680 Data 1	Data 2	IC$_{50}$ (M) Control Cmpd	Control Cmpd ID
LYN	98.61	96.84	5.40E-10	STAUROSPORINE
MARK2/PAR-1Ba	92.29	90.53	4.93E-11	STAUROSPORINE
MAST3	107.25	100.16	8.88E-07	STAUROSPORINE
MEK1	100.08	99.96	3.78E-08	STAUROSPORINE
MEKK1	96.87	96.56	1.05E-06	STAUROSPORINE
MLK1/MAP3K9	93.83	93.77	1.26E-09	STAUROSPORINE
MNK2	67.73	66.66	3.20E-08	STAUROSPORINE
MSK2/RPS6KA4	89.61	89.52	2.16E-09	STAUROSPORINE
MST1/STK4	93.06	91.02	8.13E-10	STAUROSPORINE
MYLK3	106.72	100.52	6.66E-08	STAUROSPORINE
NEK1	85.24	84.56	8.29E-09	STAUROSPORINE
P38a/MAPK14	99.67	99.17	1.96E-08	SB202190
p70S6K/RPS6KB1	86.54	86.41	3.80E-10	STAUROSPORINE
PAK4	99.78	99.70	2.97E-09	STAUROSPORINE
PIM3	78.75	77.27	7.38E-11	STAUROSPORINE
PKA	74.52	74.38	2.51E-09	STAUROSPORINE
PKCa	98.60	95.86	1.17E-10	STAUROSPORINE
PKCtheta	95.67	94.83	2.28E-10	STAUROSPORINE
PLK1	107.68	104.60	1.22E-07	STAUROSPORINE
RET	90.41	89.28	3.45E-09	STAUROSPORINE
ROCK1	57.19	56.84	5.96E-10	STAUROSPORINE
RSK1	109.07	104.16	1.62E-10	STAUROSPORINE
STK22D/TSSK1	87.41	86.50	4.58E-11	STAUROSPORINE
TEC	105.06	102.39	4.83E-08	STAUROSPORINE
TGFBR2	99.68	99.02	9.72E-08	LDN193189
TRKA	96.94	95.64	3.00E-09	STAUROSPORINE
ULK1	101.75	100.04	1.56E-08	STAUROSPORINE
PI3Ka (p110a/p85a)	97.16	93.74	3.49E-09	PI-103
PI3Kb (p110b/p85a)	91.17	90.60	9.10E-09	PI-103
PI3Kd (p110d/p85a)	103.77	102.42	1.11E-08	PI-103
PI3Kg (p110g)	99.48	98.41	2.88E-08	PI-103
